# Expedient access to saturated nitrogen heterocycles by photoredox cyclization of imino-tethered dihydropyridines†
†Electronic supplementary information (ESI) available: Supplementary substrate scope. Supplementary optimizations table. BDFE calculations. Detailed experimental procedures and compound characterization. See DOI: 10.1039/c9sc03429c


**DOI:** 10.1039/c9sc03429c

**Published:** 2019-08-28

**Authors:** Noah B. Bissonnette, J. Michael Ellis, Lawrence G. Hamann, Fedor Romanov-Michailidis

**Affiliations:** a Celgene Corporation , 200 Cambridge Park Drive, Suite 3000 , Cambridge , MA 02140 , USA . Email: fmichailidis@celgene.com ; Email: fedorromanov87@gmail.com

## Abstract

A large proportion of medicinally relevant molecules bear nitrogen and sp^3^-hybridized carbon functionalities. Overwhelmingly, these atoms are found as part of (hetero)cyclic structures. Despite their importance, synthetic approaches to saturated nitrogen heterocycles are limited to several established stoichiometric alkylation techniques, as well as a few methods involving C–H bond activation. The synthetic community remains interested in more general, mild, and sustainable ways to access these motifs. Here we describe a dual-catalyst system composed of an iridium photocatalyst and a lithium phosphate base that is capable of selectively homolyzing the N–H bond of 4-alkyl-1,4-dihydropyridines, presumably by proton-coupled-electron-transfer (PCET), and mediating efficient cyclization of the resultant carbon-centered radicals with tethered imines. The outcome of this transformation is access to a broad range of structurally complex nitrogen heterocycles obtainable from simple aldehyde starting materials in a highly chemoselective manner.

## Abstract 2

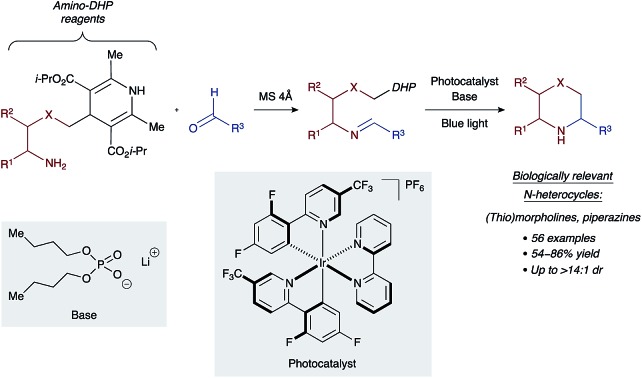
This work describes an expedient access to sp^3^-rich nitrogen heterocycles *via* mild photoredox cleavage of 4-alkyl-1,4-dihydropyridines followed by cyclization of the resultant carbon-centered radicals with tethered imines.

## 

Therapeutic drugs display a wide range of structural diversity, dense arrays of functional groups, stereochemical complexity, and a large number of sp^3^-hybridized carbon centers.^1^ When combined, these features lead to a spectrum of specific three-dimensional molecular signatures for each compound ultimately influencing biological activity and physicochemical properties.^2^ Key structural elements are responsible for the “drug-likeness” of organic molecules. In particular, nitrogen heterocycles,^3^ as well as high-fraction sp^3^ (Fsp^3^) scaffolds^4^ are among the most impactful structural components of pharmaceuticals. Indeed, although these molecular components represent a small part of the entire drug molecule, they can significantly influence on-target potency, absorption–distribution–metabolism–excretion (ADME) properties, bioavailability, and pharmacokinetics.^5^ As a result, the synthetic community has invested considerable time and effort devising methods for the efficient assembly of sp^3^-rich nitrogen heterocycles such as piperidines, morpholines, thiomorpholines, and piperazines.3b,c,^6^

Typical access to these heterocycles relies on several established stoichiometric techniques,^7^ each with its own strengths and shortcomings. Various alkylations afford morpholines, piperidines, and piperazines—although substitution can be limited. In addition, catalytic C–H activation chemistry has been recently used to prepare piperidine derivatives,^8^ but these methods often require involved substrates and expensive transition-metal catalysts. The limitations imposed by current synthetic methods can increase the difficulty of accessing polysubstituted derivatives of these heterocycles. There remains value in more general, mild, and sustainable approaches to saturated nitrogen heterocycles.

Visible light constitutes an environmentally benign and sustainable source of energy to effect chemical reactivity.^9^ Photocatalysts can remove an electron from organic substrates by harnessing over 60 kcal mol^–1^ of energy from visible light. In addition, Brønsted bases can catalyze the photo-oxidation of weakly acidic substrates by a process known as proton-coupled-electron-transfer (PCET). PCET involves the simultaneous removal of an electron and a proton from an organic molecule in a single concerted step.^10^

Work by Nishibayashi and Molander demonstrated 4-alkyl-1,4-dihydropyridines (DHPs) as latent radical precursors accessed from various aldehydes.^11^,^12^ The Hantzsch ester moiety can undergo single-electron-transfer (SET) oxidation to the corresponding radical cation **I.** followed by homolysis of the C–C bond, releasing an alkyl radical **II.** and a pyridine byproduct (Scheme 1). Given the potent hydrogen-bonding ability of Hantzsch esters,^13^ we envisioned oxidation of a DHP moiety under mild conditions by a PCET mechanism combining an iridium photocatalyst and a Brønsted basic counterion (X^–^).^14^

**Scheme 1:**
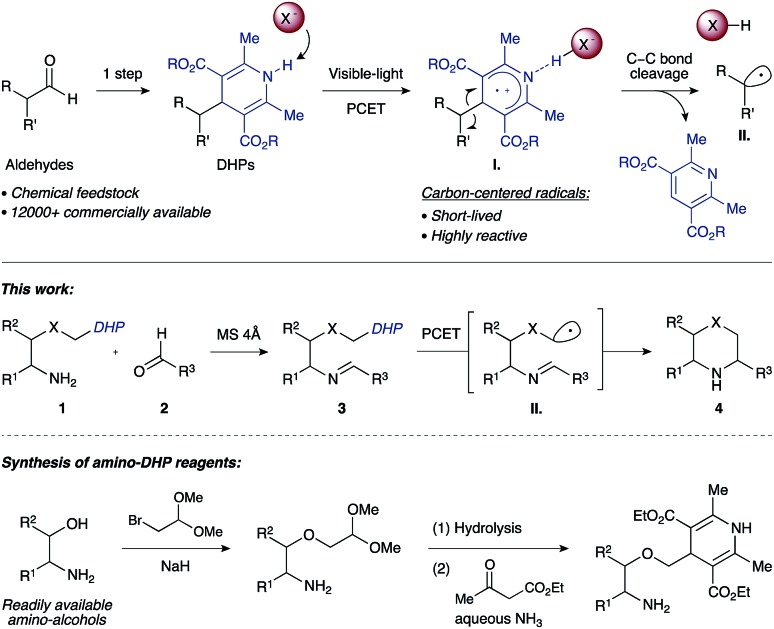
Synthesis of saturated N-heterocycles by photoredox cyclization of imino-tethered dihydropyridines.

We propose that condensation of an amino-DHP reagent **1** with an aldehyde **2** would give an imino-tethered DHP intermediate **3** that could subsequently photocyclize by a radical PCET mechanism, providing entry to medicinally relevant nitrogen heterocycles such as (thio)morpholines and piperazines **4** (Scheme 1). Earlier reports with tin (SnAP)^15^ and silicon (SLAP)^16^ radical precursors require toxic reagents, tolerate a narrow substrate scope, or implement harsh reaction conditions.^17^ In contrast, the concerted PCET pathway would allow the use of photocatalysts and bases with redox potentials (*E*^ox^) and p*K*_a_ values far removed from those of the DHP substrate being activated.^18^ Since radicals may be formed under mild reaction conditions, this was anticipated to afford wider functional group compatibility and overall synthetic utility. Herein, we describe our findings in this endeavour.

At the outset of our studies with the morpholine-forming imino-DHP substrate **3aa**, we examined photoredox catalysts that provide higher oxidation potentials in their photoexcited states (E*^ox^) (Table 1). For example, the excited state species of the cationic iridium photocatalyst [Ir(dF(CF_3_)ppy)_2_(dtbbpy)]PF_6_ (**PC1**, E*^ox^ +1.21 V *vs.* SCE;^19^,^20^ where dF(CF_3_)ppy = 2-(2,4-difluorophenyl)-5-(trifluoromethyl)pyridine, dtbbpy = 4,4′-di-*tert*-butyl-2,2′-bipyridine) should oxidize the DHP moiety (*E*^ox^ ranging from +1.01 to +1.23 V *vs.* SCE).^12^,^21^ However, attempted cyclization with this catalyst led to no desired product, unless co-catalytic base was used as an additive.^22^ Among the various bases initially tested, tetrabutylammonium acetate (p*K*_a_ of conjugate acid 12 in DMSO)^23^ gave the most promising results (25% yield of product **4aa**, entry 1). We reasoned that the low yield was the result of insufficient ability of the reduced form of photocatalyst **PC1** (*E*^red^ –1.37 V *vs.* SCE)^19^,^20^ to reduce the nitrogen-centered radical and complete the catalytic cycle.^24^ Indeed, switching to a related iridium photocatalyst lacking the *t*-Bu groups on the bipyridyl ligand [Ir(dF(CF_3_)ppy)_2_(bpy)]PF_6_ (**PC2**, E*^ox^ +0.97 V, *E*^red^ –1.23 V *vs.* SCE; where bpy = 2,2′-bipyridine)^14^,^20^ led to an improved reaction yield (33%, entry 2). Neutral iridium photocatalysts with even lower excited-state oxidation potentials (**PC3** and **PC4**, E*^ox^ +0.31 and +0.36 V *vs.* SCE)^20^ were also investigated. Moderate yields of the desired morpholine **4aa** were observed (18% and 20%, respectively, entries 3 and 4), indicating that strongly oxidizing photocatalysts are not required for successful cyclization given that co-catalytic tetrabutylammonium acetate is present.

**Table 1:** Reaction development^*a*^

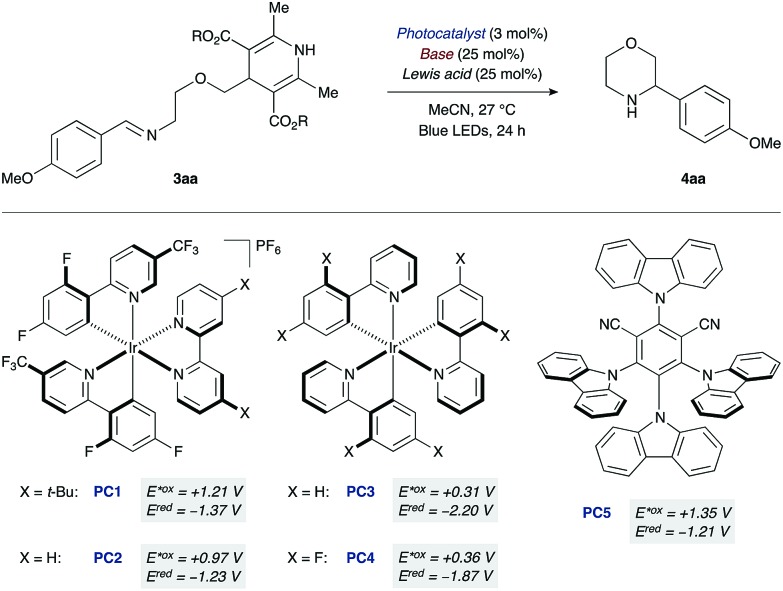				
Entry	Photocat.	Base	Effective BDFE^*b*^	Yield^*c*^ (%)
1	**PC1**	[AcO]NBu_4_	99	25
2	**PC2**	[AcO]NBu_4_	94	33
3	**PC3**	[AcO]NBu_4_	78	18
4	**PC4**	[AcO]NBu_4_	80	20
5	**PC5**	[AcO]NBu_4_	102	21
6^*f*^	**PC2**	[AcO]NBu_4_	94	39
7	**PC2**	[TFA]NBu_4_	87	36
8	**PC2**	[DBP]NBu_4_	84	41
9^*d*^	**PC2**	[DBP]NBu_4_	84	39
10^*e*^	**PC2**	[DBP]NBu_4_	84	45
11^*f*^	**PC2**	[DBP]NBu_4_	84	49
12	**PC2**	[DBP]_2_Mg	84	48
13	**PC2**	[DBP]Li	84	51
14^*g*^	**PC2**	[DBP]Li	84	58
15^*h*^	**PC2**	[DBP]Li	84	67
16^*h*^ ^,^^*i*^	**PC2**	[DBP]Li	84	80

^*a*^R = Et, for entries 1–15.

^*b*^BDFE in kcal mol^–1^.

^*c*^Isolated yield of purified product.

^*d*^With 25 mol% of Bi(OTf)_3_.

^*e*^With 25 mol% of Mg(NTf_2_)_2_.

^*f*^With 25 mol% of LiNTf_2_.

^*g*^With CH_2_Cl_2_ as solvent.

^*h*^With CH_2_Cl_2_/TFE 4 : 1 mixture as solvent.

^*i*^R = i-Pr. Ac, acetyl. TFE, 2,2,2-trifluoroethanol. TFA, 2,2,2-trifluoroacetate. DBP, di(*n*-butyl)phosphate.

To further evaluate effective photocatalyst/base combinations for oxidative DHP cleavage, we made use of a thermodynamic formalism introduced by Mayer^25^ and further elaborated by Knowles^14^ that defines an effective bond strength (“BDFE”) for any given oxidant/base pair as a function of the redox potential and p*K*_a_ value of its constituents. We tested the validity of this approach by evaluating the combinations of five photocatalysts **PC1–5** and three Brønsted bases of decreasing p*K*_a_ of their conjugate acids (AcO^–^ > CF_3_CO_2_^–^ > (*n*-BuO)_2_PO_2_^–^).^22^ We observed that combinations with “BDFE” values significantly lower than the strength of the N–H bond of the Hantzsch ester in substrate **3aa** (*ca.* 90 kcal mol^–1^)13*b* are not effective catalysts for cyclization. For example, the “BDFE” values for **PC3** or **PC4** in combination with tetrabutylammonium acetate are 78 and 80 kcal mol^–1^, respectively,^22^ and the corresponding yields of **4aa** are 18% and 20% (entries 3 and 4). Similarly, for combinations with very high “BDFE” values (>100 kcal mol^–1^), low yields of morpholine **4aa** are obtained. An organic photocatalyst with high oxidation ability, 4CzIPN (**PC5**, E*^ox^ +1.35 V *vs.* SCE),^26^ when used in conjunction with tetrabutylammonium acetate leads to only 21% yield of **4aa** despite a “BDFE” value of 102 kcal mol^–1^ (entry 5).

However, photocatalyst/base combinations with “BDFE” values approaching or slightly exceeding the N–H BDFE of **3aa** result in efficient generation of product **4aa**. For example, the “BDFE” values for **PC2** in conjunction with either trifluoroacetate [CF_3_CO_2_]NBu_4_ (p*K*_a_ of conjugate acid 7 in DMSO)^23^ or dibutylphosphate [(BuO)_2_PO_2_]NBu_4_ (p*K*_a_ of conjugate acid 5 in DMSO)^23^ are 87 and 84 kcal mol^–1^, respectively. This means that PCET activation of DHP substrate **3aa** is thermodynamically feasible with these photocatalyst/base combinations. In accord with this, a slight increase in yield is observed experimentally (36% and 41%, entries 7 and 8). Notably, iridium complex **PC2** (E*^ox^ +0.97 V *vs.* SCE) and dibutylphosphate [(BuO)_2_PO_2_]NBu_4_ (p*K*_a_ of conjugate acid 5 in DMSO) have an oxidation potential and p*K*_a_ value far removed from those of the DHP substrate (*E*^ox^ +1.23 V *vs.* SCE, p*K*_a_ 14 in DMSO).^21^,^23^ These results are consistent with a PCET mechanism for the oxidation of the Hantzsch ester moiety.

Voltammetric oxidation of 4-alkyl-1,4-dihydropyridines has been described in aprotic21*a* and protic21b,c media. While at pH < 4 the oxidation process is independent of the pH of the medium, at pH > 4 the process becomes markedly pH-dependent with deprotonation of the N–H bond of the Hantzsch ester now being the rate-determining step. Additionally, in the pH-dependent regime, the Hantzsch esters are more easily oxidized than at lower pH values. Conversely, a *N*-ethyl Hantzsch ester derivative shows a completely pH-independent oxidation behaviour. To place these observations in the context of our photocatalytic cyclization reaction and further probe the PCET mechanism for DHP cleavage, we ran additional control experiments (Table 2).

**Table 2:** Control experiments to probe the PCET mechanism for DHP cleavage. PMP, *para*-methoxy phenyl

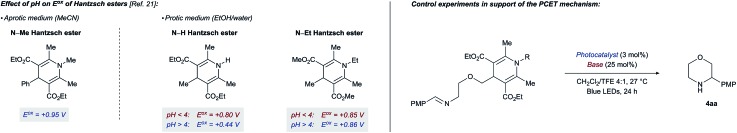							
Entry	R	Photocat.	E*^ox,^^*a*^	Base	p*K*_a_	Effective BDFE^*b*^	Yield^*c*^ (%)
1	H	**PC2**	+0.97	[AcO]NBu_4_	12	94	33
2	H	**PC2**	+0.97	[TFA]NBu_4_	7	87	36
3	H	**PC2**	+0.97	[DBP]NBu_4_	5	84	41
4	H	**PC2**	+0.97	[BAr^F^_4_]Na	<1	—	<5
5	H	**PC2**	+0.97	None	—	—	<5
6	H	**PC5**	+1.35	None	—	—	14
7	H	**PC5**	+1.35	[AcO]NBu_4_	12	102	21
8	Me	**PC2**	+0.97	[AcO]NBu_4_	12	94	<5
9	Me	**PC5**	+1.35	[AcO]NBu_4_	12	102	17

^*a*^ E*^ox^ in V.

^*b*^BDFE in kcal mol^–1^.

^*c*^Isolated yield of purified product. Ac, acetyl. TFA, 2,2,2-trifluoroacetate. DBP, di(*n*-butyl)phosphate. BAr^F^_4_, tetrakis(3,5-bis(trifluoromethyl)phenyl)borate.

Consistent with a PCET mechanism, moderate yields of cyclization product **4aa** (33–41%, entries 1–3) are witnessed for combinations of **PC2** and various bases with effective BDFE values in the range of the DHP N–H bond strength (*ca.* 90 kcal mol^–1^). In contrast, almost no product **4aa** is formed when (i) the base is replaced by a non-coordinating BAr^F^_4_ anion (entry 4), (ii) the base is omitted (entry 5), or (iii) a *N*-methyl Hantzsch ester derivative is used as substrate (entry 8). These results suggest that a base and an intact N–H bond are both required for efficient photoredox cleavage of the DHP moiety by photocatalyst **PC2** (E*^ox^ +0.97 V *vs.* SCE). On the other hand, a stronger photooxidant **PC5** (E*^ox^ +1.35 V *vs.* SCE) that does not necessarily depend on a PCET mechanism to oxidize the Hantzsch ester requires neither a base nor an intact N–H bond to promote cyclization (14% and 17% yield, entries 6 and 9). Taken together, the results of these control experiments and the published data on the pH dependency of DHP *E*^ox^ potentials are most consistent with a PCET mechanism for the cleavage of the Hantzsch ester in the title reaction. Interestingly, the cyclization with **PC5** is more efficient when performed with tetrabutylammonium acetate and an intact N–H bond both present (21% yield, entry 7). This result implies that hydrogen bonding between the Hantzsch ester and the base is important not only for efficient oxidation of the DHP to its corresponding radical cation but also for subsequent C–C bond cleavage (Scheme 1).

From the successful photocatalyst/base combinations tested, we elected to further study the **PC2**/dibutylphosphate pair. Control reactions omitting either the iridium photocatalyst or visible-light irradiation provided none of the desired cyclization product.^22^ Similarly, reactions run in the absence of the phosphate base resulted in <5% conversion of the starting DHP substrate **3aa**. Alternatively, adding Lewis acids to the reaction mixture led to interesting results (Table 1). While strong Lewis acids such as Bi(OTf)_3_ did not have much effect (39% yield, entry 9), milder Lewis acids such as Mg(NTf_2_)_2_ or LiNTf_2_ were found to promote the cyclization (45% and 49% yield, entries 10 and 11). Most reproducible results are obtained when pre-forming the dibutylphosphate with the Lewis-acidic cation. Indeed, when using 25 mol% of the [(*n*-BuO)_2_PO_2_]Li additive, the desired morpholine **4aa** is recovered in 51% yield (entry 13). Furthermore, we found that the reaction affords better results when (i) carried out in a 4 : 1 v/v mixture of dichloromethane and 2,2,2-trifluoroethanol (67% yield, entry 15), and (ii) the substrate contains bulky iso-propyl groups on the Hantzsch ester moiety (80% yield, entry 16).

To demonstrate the versatility of our one-pot protocol, various aldehydes **2a–z**, **2α**, and **2β** (blue colour) were condensed with either morpholine-, thiomorpholine-, or piperazine-forming amino-DHP reagents **1a–j** (red colour), and then subjected to the photoredox cyclization with visible light irradiation (Scheme 2).

**Scheme 2:**
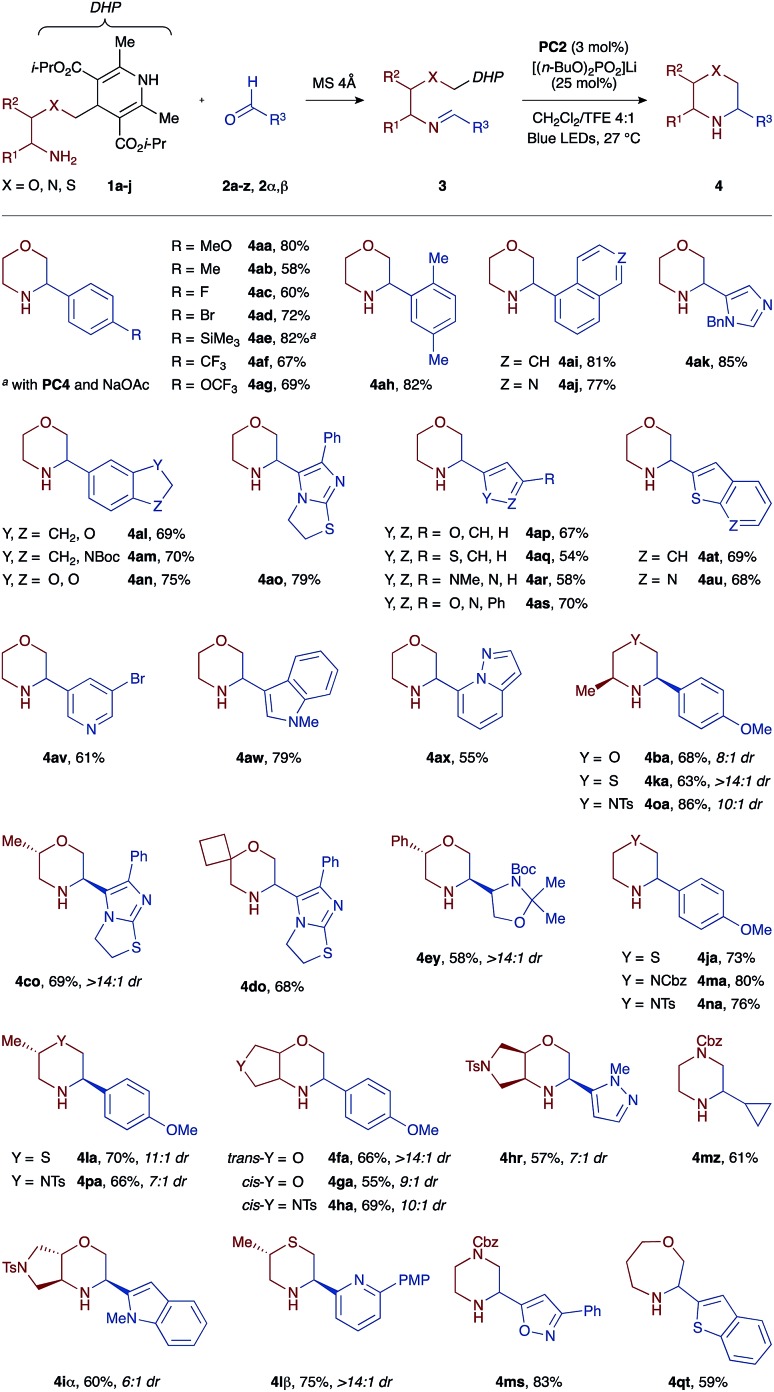
A representative substrate scope.

The cyclization tolerates a broad spectrum of substituents and functional groups. For example, benzaldehyde derivatives bearing either electron-withdrawing (CF_3_, OCF_3_, and F) or -releasing (Me, MeO, and SiMe_3_) substituents at the *para* position all cyclize well with the morpholine-forming amino-DHP reagent **1a**, affording the corresponding heterocyclic products **4aa–4ag** in high yields (58–82%). We were pleased to find that imines **3** derived from heteroaromatic aldehydes also work well in our reaction. These substrates are particularly interesting from a drug discovery perspective, but are challenging to incorporate into redox reactions due to competing off-target oxidation events and non-specific binding of the basic nitrogen atoms to Lewis acids. However, under our mild PCET conditions, a wide array of heteroaromatic substrates are tolerated, ranging from electron-deficient pyridines (**3av** and **3lβ**), thienopyridines (**3au**), isoquinolines (**3aj**), imidazoles (**3ak**), pyrazolopyridines (**3ax**), oxazoles (**3as** and **3ms**), and pyrazoles (**3ar** and **3hr**) to electron-rich furans (**3ap**), thiophenes (**3aq**), benzothiophenes (**3at** and **3jt**), indoles (**3aw** and **3iα**), indolines (**3am**), and dihydroimidazothiazoles (**3co** and **3do**). Lastly, imines derived from aliphatic aldehydes cyclize equally well, giving access to pharmacologically relevant products such as chiral glyceraldehyde-derived morpholine **4ey** (58% yield) or cyclopropyl-substituted piperazine **4mz** (61% yield). Importantly, the necessity of hydrogen-bonding between the Hantzsch ester moiety and the phosphate base enables chemoselective PCET activation even in the presence of more readily oxidizable groups such as indole (*e.g.*, imines **3aw** or **3iα** with *E*^ox^ +1.21 V *vs.* SCE).

With respect to the amino-DHP component, various branched tethers are accommodated in the cyclization reaction, including those bearing methyl (**1b** and **1c**) or phenyl groups (**1e**), spirocycles (**1d**), and *trans*- (**1f** and **1i**) or *cis*-fused bicycles (**1g** and **1h**). The yields of the corresponding morpholine products are mostly high (55–69%), and excellent diastereoselectivities (up to >14 : 1 dr) are observed in cases where multiple stereogenic centers are created such as in products **4ba**, **4co**, **4fa**, **4ga**, **4ha**, **4hr**, and **4iα**. Moreover, the reaction outcome is not restricted to six-membered products, and moderately sized ring systems such as **4qt** can be accessed with equally high efficiency (59% yield). A more exhaustive list of obtainable heterocyclic products (>50 examples) is compiled in the ESI.† 22

The generality of our approach was further evaluated by investigating access to saturated nitrogen heterocycles other than morpholines. To this end, we devised a set of thiomorpholine- (**1j**, **1k**, and **1l**) and piperazine-forming (**1m**, **1n**, **1o**, and **1p**) amino-DHP reagents that were first condensed with aldehydes **2a**, **2s**, **2z**, and **2β** and then photocyclized with blue light (Table 1). To our great delight, all of the substrate combinations examined were found to give the expected cyclized products in high yields (63–86%) and with excellent levels of diastereocontrol (up to >14 : 1 dr). This is an important finding as it points to the utility of this approach *versus* previously described methods where the redox potentials of the tin or silicon reagents shift greatly as a function of the heteroatom present in the substrate: *E*^ox^(O) > *E*^ox^(S) > *E*^ox^(N).^15^–^17^ Conversely, because the same DHP moiety is used in all our substrate types, its oxidation potential (*E*^ox^ +1.23 V *vs.* SCE) remains unaffected by the nature of the tethering heteroatom and permits the synthesis of morpholines, thiomorpholines, and piperazines under a single set of catalytic conditions.

As shown in Scheme 3, a plausible redox-neutral photocatalytic cycle involves: (1) PCET quenching of the photoexcited Ir(iii)* species (E*^ox^ +0.97 V) by imino-DHP **3** (*E*^ox^ +1.23 V) with help from the Brønsted-basic phosphate anion (X^–^, p*K*_a_ of conjugate acid 5); (2) β-scission of the resultant radical-cationic intermediate **I.** that affords α-heteroatom stabilized C-centered radical **II.**, conjugate acid XH, and pyridine byproduct **5**; (3) Baldwin-allowed 6-*endo*-trig cyclization of **II.** that delivers N-centered radical **III.**; and (4) SET-reduction of **III.** by the Ir(ii) photocatalyst (*E*^red^ –1.23 V) followed by proton transfer from acid XH that furnishes morpholine **4** and regenerates the ground-state Ir(iii) photocatalyst as well as the X^–^ counterion.

**Scheme 3:**
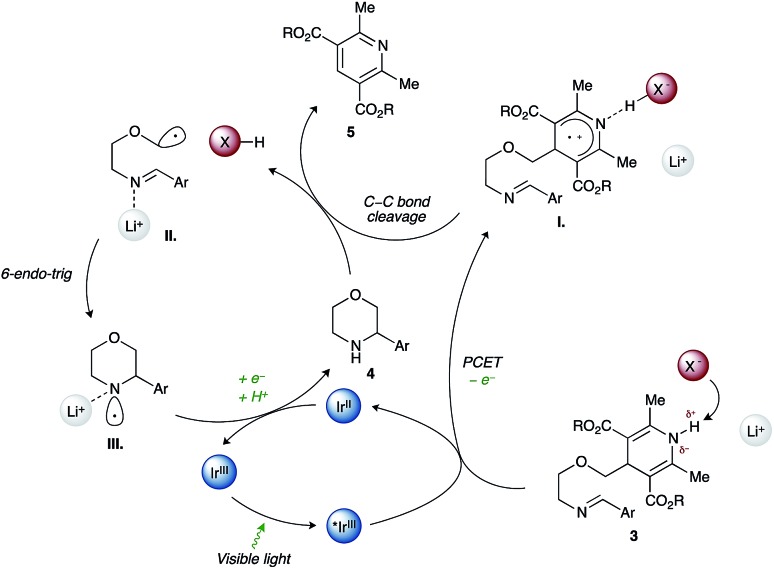
Postulated catalytic cycle.

We reason that the Hantzsch ester moiety in imino-DHP **3** (p*K*_a_ 14) can form a strong H-bond with dibutylphosphate (p*K*_a_ of conjugate acid 5). Although H-bonding of the N–H bond is anticipated to lower the potential required for photooxidation of the Hantzsch ester, it will not fully deprotonate the resultant radical cationic intermediate **I.** Consequently, the nascent radical cation will only be generated when the substrate is associated with the anion. This explains the observed high chemoselectivity of our method. Indeed, the employed photocatalyst/base dual-catalyst system is reluctant to oxidizing other redox-labile functional groups present in substrates given that they do not have N–H bonds of sufficient acidity. Furthermore, we believe that the Lewis-acidic lithium cation coordinates to the N-centered radical **III.** leading to its stabilization and a concomitant decrease of its reduction potential relative to the uncoordinated N-centered radical.^27^ This in turn renders reduction of **III.** by the Ir(ii) photocatalyst (*E*^red^ –1.23 V) more facile.

Nitrogen heterocycles are among the most abundant structural components of pharmaceuticals. For example, of the top 200 brand name commercial medicines in 2014, 116 contain a nitrogen heterocycle.3*a* We have developed a one-pot approach to saturated nitrogen heterocycles that harnesses energy from visible light to cleave 4-alkyl-1,4-dihydropyridines by a unique PCET mechanism. Cleavage is followed by cyclization of the resultant carbon-centered radical with a tethered imine, giving heterocyclic products. Our mild protocol is characterized by a remarkably broad substrate scope and high functional group tolerance. The concise assembly of a variety of these heterocycles should allow desirable properties such as solubility, permeability, and metabolic stability, to be built into lead series, ultimately increasing the pace of drug discovery.

## Author contributions

This project was conceived and initiated by FRM. Optimization of the reaction conditions, the synthesis and characterization of starting materials and products was performed by NBB with assistance from FRM. The manuscript was drafted by FRM and proofread by FRM, NBB, LGH, and JME. The ESI was drafted by NBB and proofread by FRM, NBB, LGH, and JME.

## Conflicts of interest

There are no conflicts to declare.

## Supplementary Material

Supplementary information

## References

[cit1] Butler M. S., Robertson A. A., Cooper M. A. (2014). Nat. Prod. Rep..

[cit2] Clemons P. A., Bodycombe N. E., Carrinski H. A., Wilson J. A., Shamii A. F., Wagner B. K., Koethler A. N., Schreiber S. L. (2010). Proc. Natl. Acad. Sci. U. S. A..

[cit3] Vitaku E., Smith D. T., Njardarson J. T. (2014). J. Med. Chem..

[cit4] Lovering F., Bikker J., Humblet C. J. (2009). J. Med. Chem..

[cit5] Brown D. G., Boström J. (2016). J. Med. Chem..

[cit6] Cernak T., Dykstra K. D., Tyagarajan S., Vachal P., Krska S. W. (2016). Chem. Soc. Rev..

[cit7] Worlikar S. A., Kesharwani T., Yao T., Larock R. C. (2007). J. Org. Chem..

[cit8] Romanov-Michailidis F., Sedillo K. F., Neely J. M., Rovis T. (2015). J. Am. Chem. Soc..

[cit9] Twilton J., Le C., Zhang P., Shaw M. H., Evans R. W., MacMillan D. W. C. (2017). Nat. Rev..

[cit10] Mayer J. M. (2004). Annu. Rev. Phys. Chem..

[cit11] Nakajima K., Nojima S., Sakata K., Nishibayashi Y. (2016). ChemCatChem.

[cit12] Gutiérrez-Bonet A., Remeur C., Matsui J. K., Molander G. A. (2017). J. Am. Chem. Soc..

[cit13] Eisner U., Kuthan J. (1972). Chem. Rev..

[cit14] Choi G. J., Knowles R. R. (2015). J. Am. Chem. Soc..

[cit15] Vo C.-V. T., Mikutis G., Bode J. W. (2013). Angew. Chem., Int. Ed..

[cit16] Hsieh S.-Y., Bode J. W. (2017). ACS Cent. Sci..

[cit17] Luescher M. U., Vo C.-V. T., Bode J. W. (2014). Org. Lett..

[cit18] Gentry E. C., Knowles R. R. (2016). Acc. Chem. Res..

[cit19] Slinker J. D., Gorodetsky A. A., Lowry M. S., Wang J., Parker S., Rohl R., Bernhard S., Malliaras G. G. (2004). J. Am. Chem. Soc..

[cit20] Teegardin K., Day J. I., Chan J., Weaver J. (2016). Org. Process Res. Dev..

[cit21] Stradins J., Ogle J., Kadysh V., Baumane L., Gavars R., Duburs G. (1987). J. Electroanal. Chem..

[cit22] See the ESI for details

[cit23] Bordwell F. G. (1988). Acc. Chem. Res..

[cit24] Uoyama H., Goushi K., Shizu K., Nomura H., Adachi C. (2012). Nature.

[cit25] Warren J. J., Tronic T. A., Mayer J. M. (2010). Chem. Rev..

[cit26] Romero N. A., Nicewicz D. A. (2016). Chem. Rev..

[cit27] Jonsson M., Wayner D. D. M., Lusztyk J. (1996). J. Phys. Chem..

