# Supplementary material for: Expedient access to saturated nitrogen heterocycles by photoredox cyclization of imino-tethered dihydropyridines
Source: Chem Sci. 2019 Aug 28;10(41):9591–6. doi: 10.1039/c9sc03429c (PMC6993613; doi:10.1039/c9sc03429c)
Supplement: Supplementary file 1 [file SC-010-C9SC03429C-s001.pdf]

Electronic Supplementary Material (ESI) for Chemical Science.
This journal is © The Royal Society of Chemistry 2019

Supporting Information

Expedient Access to Saturated Nitrogen Heterocycles by Photoredox Cyclization of Imino-Tethered Dihydropyridines

*Noah B. Bissonnette, J. Michael Ellis, Lawrence G. Hamann, Fedor Romanov-Michailidis**

Table of Contents

General Information 3

Photoredox Reactor Set-Up 4

Experimental Procedures 5

Photoredox Cyclization Procedures 5

General Procedure A 5

General Procedure B 5

General Procedure C 5

Formal Bond Dissociation Free Energy Calculations 6

Full Optimizations Table 6

Complete List of N-Heterocyclic Products 8

Starting Material Synthesis Procedures 9

General Procedure A: Morpholine-Forming Amino-DHP Reagents (Step 1) 9

General Procedure B: Morpholine-Forming Amino-DHP Reagents (Step 2) 9

General Procedure C: Morpholine-Forming Amino-DHP Reagents (Step 3) 10

General Procedure D: Morpholine-Forming Amino-DHP Reagents (Step 4): Ethyl-ester and Nitrile Hantzsch esters 10

General Procedure E: Morpholine-Forming Amino-DHP Reagents (Step 4): iso-Propyl and tert-Butyl Hantzsch esters 10

General Procedure F: Morpholine-Forming Amino-DHP Reagents (Step 5) 11

General Procedure G: Thiomorpholine- and Piperazine-Forming Amino-DHP Reagents (Step 1) 11

General Procedure H: Morpholine-Forming Amino-DHP Reagents: Pyrrolidino Amines 12

General Procedure I: Thiomorpholine-Forming Amino-DHP Reagents (Step 2) 12

General Procedure J: Piperazine-Forming Amino-DHP Reagents (Step 2) 12

General Procedure K: Piperazine-Forming Amino-DHP Reagents (Step 3): p-Tosyl Piperazines 13

General Procedure L: Piperazine-Forming Amino-DHP Reagents (Step 3): Cbz Piperazines 13

General Procedure M: Thiomorpholine- and Piperazine-Forming Amino-DHP Reagents (Step 4) 14

General Procedure N: Thiomorpholine- and Piperazine-Forming Amino-DHP Reagents (Step 5) 14

General Procedure O: Thiomorpholine- and Piperazine-Forming Amino-DHP Reagents (Step 6) 15

General Procedure P: Metal Phosphate Salts....15

Characterization of Starting Materials 16

Complete List of Synthesized Amino-DHP Reagents....16

Morpholine-Forming Amino-DHP Reagents 16

Thiomorpholine-Forming Amino-DHP Reagents 27

Piperazine-Forming Amino-DHP Reagents 30

Complete List of Commercially-Available Aldehydes 36

Product Characterization 36

NMR and Mass Spectra....51

Starting Materials 51

Morpholine-Forming Amino-DHP Reagents 51

Thiomorpholine-Forming Amino-DHP Reagents 96

Piperazine-Forming Amino-DHP Reagents 108

N-Heterocyclic Products....126

General Information

Unless otherwise noted, all reactions were performed in oven-dried glassware and carried out under an atmosphere of argon or nitrogen with magnetic stirring. All column chromatography was performed using a CombiFlash Rf+ Automated Flash Chromatography System with a 200 mL/min max flow rate, 200 psi, and 200–400 nm UV variable wavelength detector, and Silica RediSep Rf gold columns (Normal Phase) or C18 Silica RediSep Rf gold columns (Reversed Phase). Thin layer chromatography was performed on SiliCycle® 250 µm 60 Å plates. Visualization was accomplished with 254 nm UV light, $KMnO_4$ stain, or I_2.

NMR spectra were recorded on Bruker Ascend 400 MHz spectrometer at ambient temperature. Chemical shifts (δ) are reported in parts per million (ppm) from $CDCl_3$ (^{1}H: 7.26 ppm, ^{13}C: 77.16 ppm), CD_2Cl_2 (^{1}H: 5.32 ppm, ^{13}C: 54.0 ppm), or CD_3CN (^{1}H: 1.94 ppm, ^{13}C: 1.32 ppm) with multiplicity (*s* = singlet, br. *s* = broad singlet, *d* = doublet, *t* = triplet, *q* = quartet, and *m* = multiplet) and coupling constants (in Hz).

Low-resolution mass spectra were recorded on a Waters Acquity BEH uPLC-MS with C18 column (50 mm × 2.1 mm, 1.7) at 40 °C. The solvents employed were: A = 0.1% *v/v* solution of ammonium hydroxide in water; B = 0.1% *v/v* solution of ammonium hydroxide in acetonitrile. The gradient employed was as follows:

Time Flow Rate (minutes) (mL/min) % A % B 0 0.8 95 5 0.1 0.8 95 5 1.5 0.8 5 95 2.0 0.8 5 95 2.1 0.8 95 5 3 0.8 95 5.

Unless otherwise stated, all starting materials were obtained from commercial sources including Sigma-Aldrich, TCI, Matrix, Alfa-Aesar, and Oakwood Scientific. Anhydrous $Cu(OAc)_2$ and CsOAd were obtained from Sigma-Aldrich and stored in an argon-filled glovebox.

Photoredox Reactor Set-Up

All photoredox reactions were irradiated by a blue Kessil PR160 440 nm lamp (max intensity), with cooling to *ca.* 27 °C with an internal fan, and magnetic stirring using a Corning PC-410D stir plate.

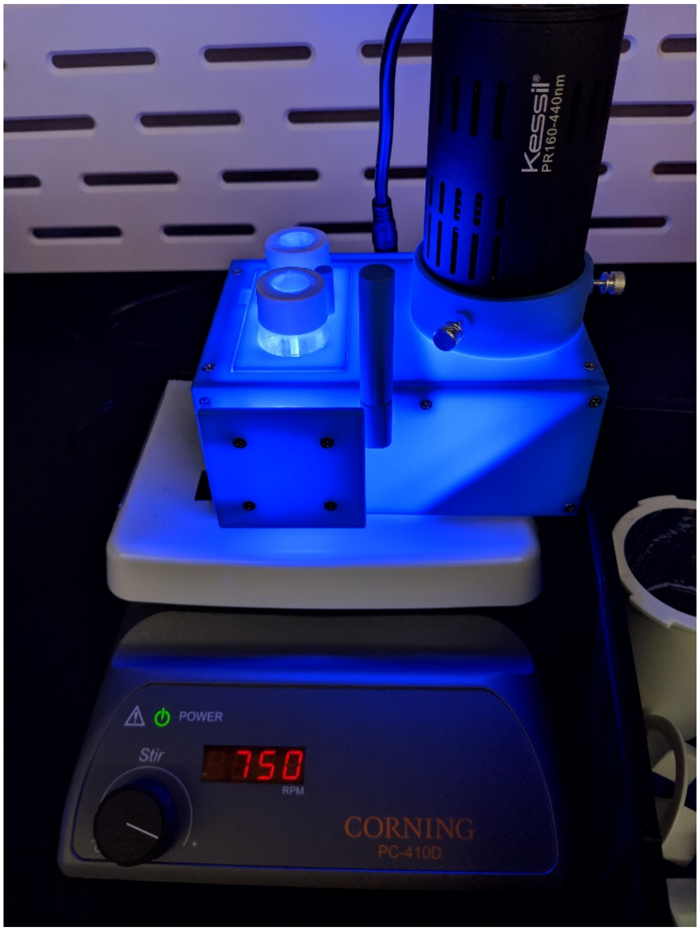

Experimental Procedures

Photoredox Cyclization Procedures

General Procedure A

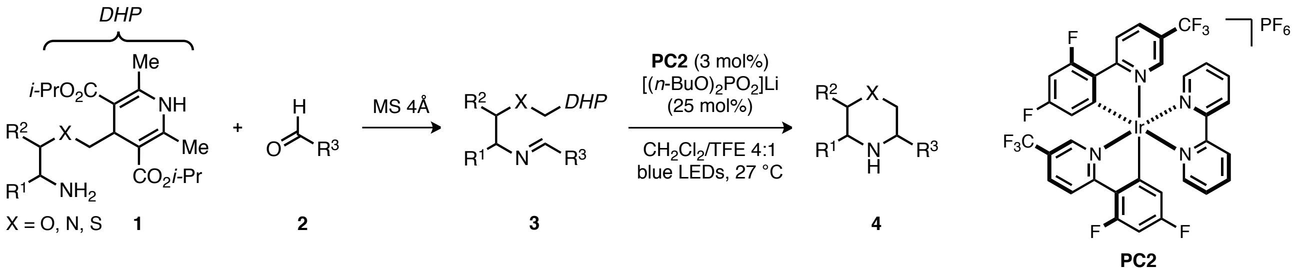

To an oven-dried scintillation vial, 3 Å molecular sieves (*ca.* 400 mg) were added to a solution of amino-DHP reagent **1** (1.0 equiv.), aldehyde **2** (0.95 equiv.) in anhydrous CH_2Cl_2 (0.1 M). The resultant mixture was then stirred for 12 h at room temperature. Next, the solution was filtered through a Celite plug and concentrated under reduced pressure yielding the corresponding imine **3**. Next, to a Schlenk pressure tube were added: the above-prepared imine **3**, iridium photocatalyst $[Ir(dF(CF_3)ppy)_2(bpy)]PF_6$ (**PC2**, 3 mol%), lithium dibutylphosphate $[(BuO)_2PO_2]Li$ (30 mol%), and CH_2Cl_2/TFE 4:1 *v/v* (0.075 M). The resultant solution was then purged of oxygen with two freeze-pump-thaw cycles. Afterwards, the tube was irradiated with blue LED light for 12-16 h, while the mixture was stirred at 500 rpm and cooled with an internal fan to *ca.* 27 °C. Upon completion, the reaction mixture was filtered through a Celite plug, the filtrate was concentrated under reduced pressure, and the crude residue was purified by reverse-phase flash chromatography (C18, eluent: water/MeCN with 0.1% TFA as modifier). The pure fractions were combined and concentrated under reduced pressure. Final lyophilization afforded pure, dry product **4** as the corresponding TFA salt.

General Procedure B

Same as General Procedure A except that 0.1% NH_4OH was used as modifier for the reverse-phase chromatographic purification.

General Procedure C

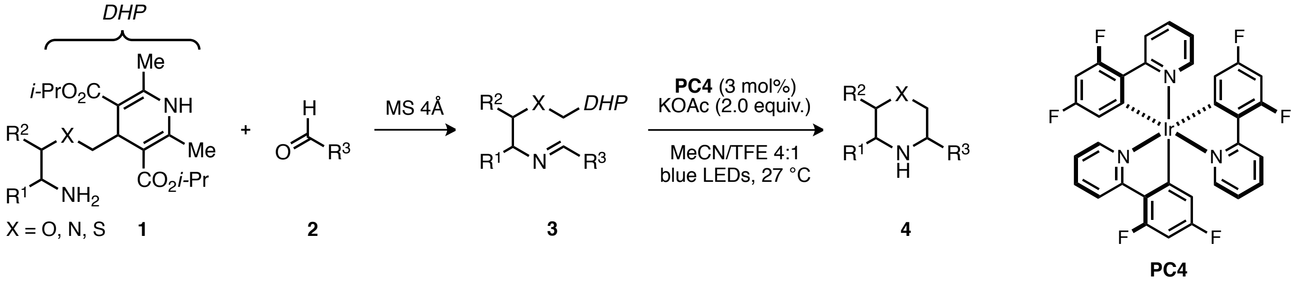

Imine **3** was prepared as described in General Procedure A. To a Schlenk pressure tube were added: the above-prepared imine **3**, iridium photocatalyst $Ir(dFppy)_3$ (**PC4**, 3 mol%), potassium acetate (2.0 equiv.), and MeCN/TFE 4:1 *v/v* (0.075 M). The resultant solution was then purged of oxygen with two freeze-pump-thaw

cycles. Afterwards, the tube was irradiated with blue LED light for 12-16 h, while the mixture was stirred at 500 rpm and cooled with an internal fan to *ca.* 27 °C. Upon completion, the reaction mixture was filtered through a Celite plug, the filtrate was concentrated under reduced pressure, and the crude residue was purified by reverse-phase flash chromatography (C18, eluent: water/MeCN with 0.1% TFA or NH_4OH as modifier). The pure fractions were combined and concentrated under reduced pressure. Final lyophilization afforded pure, dry product **4**.

Formal Bond Dissociation Free Energy Calculations

$$\text{"BDFE" (kcal/mol)} = 2.3RT\text{p}K_a + 23.06E^{*ox} + 54.9$$

Entry	Photooxidant	E^{*ox} (V vs SCE)	Base	pK_a (DMSO)	"BDFE"
1	$[Ir(dF(CF_3)ppy)_2(dtbbpy)]PF_6$ (**PC1**)	+1.21	$[CH_3CO_2]NBu_4$	12	99
2	$[Ir(dF(CF_3)ppy)_2(bpy)]PF_6$ (**PC2**)	+0.97	$[CH_3CO_2]NBu_4$	12	94
3	$Ir(ppy)_3$ (**PC3**)	+0.31	$[CH_3CO_2]NBu_4$	12	78
4	$Ir(dFppy)_3$ (**PC4**)	+0.36	$[CH_3CO_2]NBu_4$	12	80
5	4CzIPN (**PC5**)	+1.35	$[CH_3CO_2]NBu_4$	12	102
6	$[Ir(dF(CF_3)ppy)_2(bpy)]PF_6$ (**PC2**)	+0.97	$[CF_3CO_2]NBu_4$	7	87
7	$[Ir(dF(CF_3)ppy)_2(bpy)]PF_6$ (**PC2**)	+0.97	$[(BuO)_2PO_2]NBu_4$	5	84

Full Optimizations Table

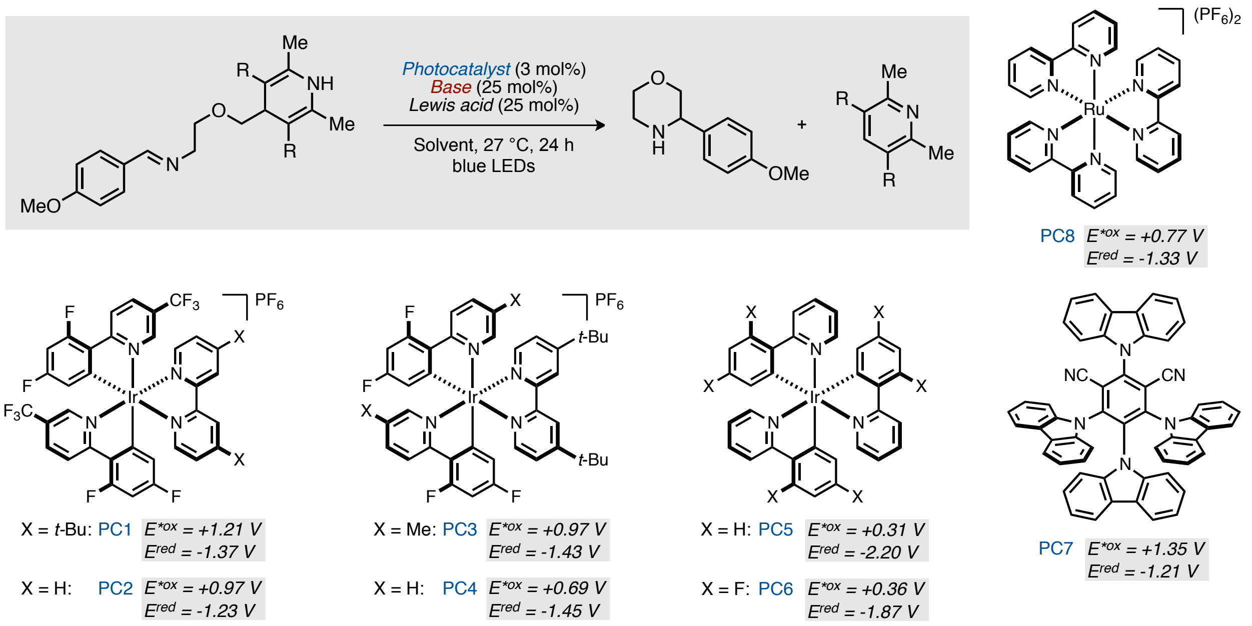

Entry	R	Photocatalyst	Base	Lewis acid	Solvent	Yield[a]
1	CO_2Et	$[Ir(dF(CF_3)ppy)_2(dtbbpy)]PF_6$ (**PC1**)	$[CH_3CO_2]NBu_4$	none	MeCN	25

2	CO_2Et	$[Ir(dF(CF_3)ppy)_2(bpy)]PF_6$ (**PC2**)	$[CH_3CO_2]NBu_4$	none	MeCN	33
3	CO_2Et	$[Ir(dF(Me)ppy)_2(dtbbpy)]PF_6$ (**PC3**)	$[CH_3CO_2]NBu_4$	none	MeCN	27
4	CO_2Et	$[Ir(dFppy)_2(dtbbpy)]PF_6$ (**PC4**)	$[CH_3CO_2]NBu_4$	none	MeCN	30
5	CO_2Et	$Ir(ppy)_3$ (**PC5**)	$[CH_3CO_2]NBu_4$	none	MeCN	18
6	CO_2Et	$Ir(dFppy)_3$ (**PC6**)	$[CH_3CO_2]NBu_4$	none	MeCN	20
7	CO_2Et	4CzIPN (**PC7**)	$[CH_3CO_2]NBu_4$	none	MeCN	16
8	CO_2Et	$[Ru(bpy)_3]PF_6$ (**PC8**)	$[CH_3CO_2]NBu_4$	none	MeCN	9
9	CO_2Et	$[Ir(dF(CF_3)ppy)_2(bpy)]PF_6$ (**PC2**)	2,6-Lutidine	none	MeCN	0
10	CO_2Et	$[Ir(dF(CF_3)ppy)_2(bpy)]PF_6$ (**PC2**)	$[PhCO_2]NBu_4$	none	MeCN	31
11	CO_2Et	$[Ir(dF(CF_3)ppy)_2(bpy)]PF_6$ (**PC2**)	$[CF_3CO_2]NBu_4$	none	MeCN	36
12	CO_2Et	$[Ir(dF(CF_3)ppy)_2(bpy)]PF_6$ (**PC2**)	$[(BuO)_2PO_2]NBu_4$	none	MeCN	41
13	CO_2Et	$[Ir(dF(CF_3)ppy)_2(bpy)]PF_6$ (**PC2**)	$[(PhO)_2PO_2]NBu_4$	none	MeCN	25
14	CO_2Et	$[Ir(dF(CF_3)ppy)_2(bpy)]PF_6$ (**PC2**)	$[(BuO)_2PO_2]NBu_4$	$Bi(OTf)_3$	MeCN	39
15	CO_2Et	$[Ir(dF(CF_3)ppy)_2(bpy)]PF_6$ (**PC2**)	$[(BuO)_2PO_2]NBu_4$	$Sc(OTf)_3$	MeCN	35
16	CO_2Et	$[Ir(dF(CF_3)ppy)_2(bpy)]PF_6$ (**PC2**)	$[(BuO)_2PO_2]NBu_4$	$Cu(OTf)_2$	MeCN	26
17	CO_2Et	$[Ir(dF(CF_3)ppy)_2(bpy)]PF_6$ (**PC2**)	$[(BuO)_2PO_2]NBu_4$	$Zn(OTf)_2$	MeCN	29
18	CO_2Et	$[Ir(dF(CF_3)ppy)_2(bpy)]PF_6$ (**PC2**)	$[(BuO)_2PO_2]NBu_4$	Me_3SiOTf	MeCN	10
19	CO_2Et	$[Ir(dF(CF_3)ppy)_2(bpy)]PF_6$ (**PC2**)	2,6-Lutidine	Me_3SiOTf	MeCN	40
20	CO_2Et	$[Ir(dF(CF_3)ppy)_2(bpy)]PF_6$ (**PC2**)	$[(BuO)_2PO_2]NBu_4$	$Mg(NTf_2)_2$	MeCN	45
21	CO_2Et	$[Ir(dF(CF_3)ppy)_2(bpy)]PF_6$ (**PC2**)	$[(BuO)_2PO_2]NBu_4$	$Ca(NTf_2)_2$	MeCN	47
22	CO_2Et	$[Ir(dF(CF_3)ppy)_2(bpy)]PF_6$ (**PC2**)	$[(BuO)_2PO_2]NBu_4$	$LiNTf_2$	MeCN	49
23	CO_2Et	$[Ir(dF(CF_3)ppy)_2(bpy)]PF_6$ (**PC2**)	$[(BuO)_2PO_2]_2Mg$	none	MeCN	48
24	CO_2Et	$[Ir(dF(CF_3)ppy)_2(bpy)]PF_6$ (**PC2**)	$[(BuO)_2PO_2]_2Ca$	none	MeCN	50
25	CO_2Et	$[Ir(dF(CF_3)ppy)_2(bpy)]PF_6$ (**PC2**)	$[(BuO)_2PO_2]Li$	none	MeCN	51
26	CO_2Et	$[Ir(dF(CF_3)ppy)_2(bpy)]PF_6$ (**PC2**)	$[(BuO)_2PO_2]Li$	none	CH_2Cl_2	58
27	CO_2Et	$[Ir(dF(CF_3)ppy)_2(bpy)]PF_6$ (**PC2**)	$[(BuO)_2PO_2]Li$	none	DMF	36
28	CO_2Et	$[Ir(dF(CF_3)ppy)_2(bpy)]PF_6$ (**PC2**)	$[(BuO)_2PO_2]Li$	none	MeCN/TFE 4:1	45
29	CO_2Et	$[Ir(dF(CF_3)ppy)_2(bpy)]PF_6$ (**PC2**)	$[(BuO)_2PO_2]Li$	none	CH_2Cl_2/TFE 4:1	67
30	CO_2*i*-Pr	$[Ir(dF(CF_3)ppy)_2(bpy)]PF_6$ (**PC2**)	$[(BuO)_2PO_2]Li$	none	CH_2Cl_2/TFE 4:1	80
31	CO_2*t*-Bu	$[Ir(dF(CF_3)ppy)_2(bpy)]PF_6$ (**PC2**)	$[(BuO)_2PO_2]Li$	none	CH_2Cl_2/TFE 4:1	77
32	CN	$[Ir(dF(CF_3)ppy)_2(bpy)]PF_6$ (**PC2**)	$[(BuO)_2PO_2]Li$	none	CH_2Cl_2/TFE 4:1	n.d.[b]
33	CO_2*i*-Pr	none	$[(BuO)_2PO_2]Li$	none	CH_2Cl_2/TFE 4:1	0
34	CO_2*i*-Pr	$[Ir(dF(CF_3)ppy)_2(bpy)]PF_6$ (**PC2**)	$[(BuO)_2PO_2]Li$	none	CH_2Cl_2/TFE 4:1	0[c]
35	CO_2*i*-Pr	$[Ir(dF(CF_3)ppy)_2(bpy)]PF_6$ (**PC2**)	none	none	CH_2Cl_2/TFE 4:1	< 5

[a] % yield of isolated product. [b] No desired product was observed. The reaction led to numerous side products. [c] Reaction performed in the absence of blue light irradiation.

Complete List of N-Heterocyclic Products

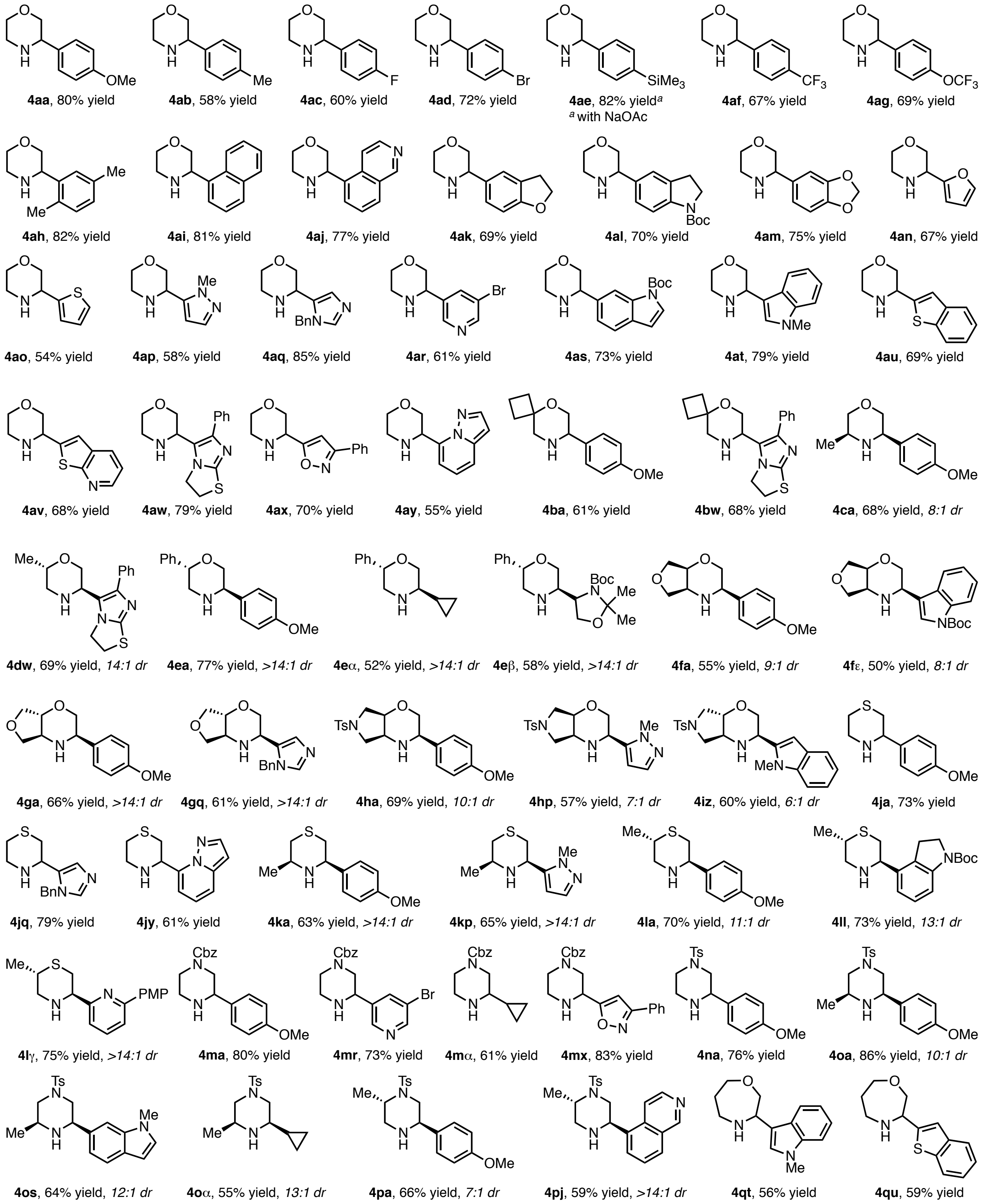

Scheme S1. List of *N*-heterocyclic products.

Starting Material Synthesis Procedures

General Procedure A: Morpholine-Forming Amino-DHP Reagents (Step 1)

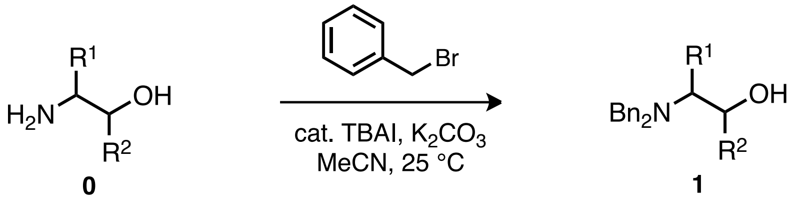

To a solution composed of amino-alcohol **0** (1.0 equiv.), tetrabutylammonium iodide (0.1 equiv.), and potassium carbonate (2.0 equiv.) in anhydrous MeCN (0.5 M) was added benzyl bromide (2.05 equiv.) dropwise at room temperature. The reaction mixture was stirred at room temperature for 18 h, and then concentrated under reduced pressure. The crude residue was partitioned between ethyl acetate and water. The aqueous layer was extracted with ethyl acetate, and the combined organic layers were dried over anhydrous Na_2SO_4, filtered, and concentrated under reduced pressure. The crude product was purified by silica gel flash chromatography (eluent: *n*-heptane/EtOAc 4:1 *v/v*). Pure fractions were combined and concentrated under reduced pressure, yielding dibenzyl-protected amino-alcohol **1**.

General Procedure B: Morpholine-Forming Amino-DHP Reagents (Step 2)

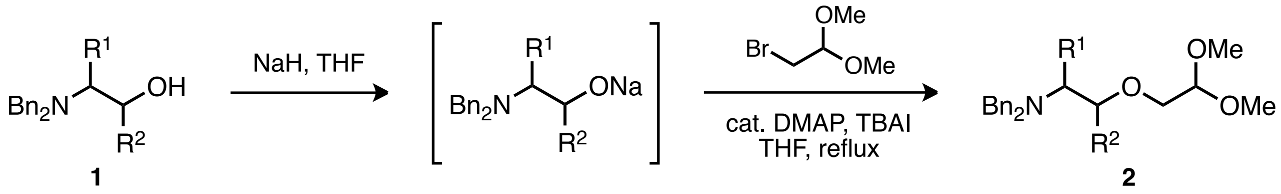

Sodium hydride (60% in mineral oils, pre-washed with pentane, 2.0 equiv.) was placed in an oven-dried two-necked flask, evacuated and placed under a nitrogen atmosphere. Anhydrous THF (0.3 M) was then added, and the solution was cooled down to 0 °C. Next, dibenzyl-protected amino-alcohol **1** in anhydrous THF was added dropwise, and the resultant solution was warmed up to room temperature and stirred for 3 h. The reaction mixture was cooled down to 0 °C, and DMAP (0.02 equiv.), tetrabutylammonium iodide (0.1 equiv.), and 2-bromo-1,1-dimethoxyethane (3.0 equiv.) were added. The mixture was stirred at 0 °C for 1 h followed by refluxing for 12-18 h. The reaction mixture was quenched with aqueous NH_4Cl at 0 °C, and ethyl acetate was added. The aqueous layer was extracted with ethyl acetate, and the organic layers were combined, dried over anhydrous Na_2SO_4, filtered, and concentrated under reduced pressure. The crude residue was purified by silica gel flash chromatography (eluent: *n*-heptane/EtOAc 4:1 *v/v*). Pure fractions were combined and concentrated under reduced pressure, yielding acetal product **2**.

General Procedure C: Morpholine-Forming Amino-DHP Reagents (Step 3)

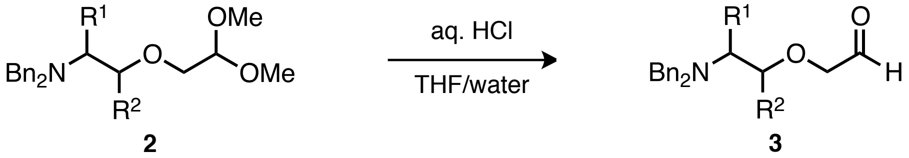

To a cooled (0 °C) solution of dibenzyl-protected acetal **2** (1.0 equiv.) in THF (0.3 M) was added aqueous 6 N HCl (10 equiv.). The reaction mixture was warmed up to room temperature and stirred for 7 h. Upon completion, the reaction was quenched with saturated aqueous $NaHCO_3$ at 0 °C, and methylene chloride was added. The aqueous layer was extracted with methylene chloride, dried over anhydrous Na_2SO_4, filtered, and concentrated under reduced pressure, yielding dibenzyl-protected aldehyde product **3**. The crude product was used in the next step as such, without further purification.

General Procedure D: Morpholine-Forming Amino-DHP Reagents (Step 4): Ethyl-ester and Nitrile Hantzsch esters

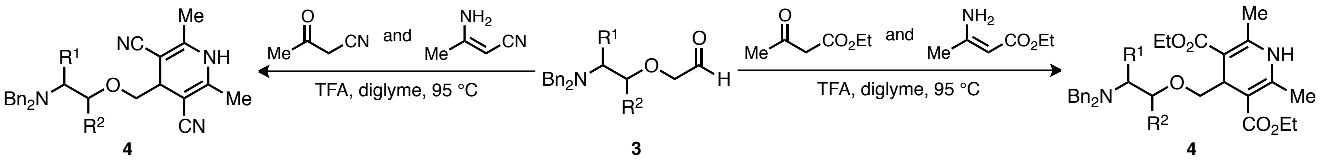

A mixture of aldehyde **3** (1.0 equiv.), ethyl acetoacetate or 3-oxobutanenitrile (1.0 equiv.), ethyl 3-aminocrotonate or 3-aminocrotononitrile (1.0 equiv.), and trifluoroacetic acid (1.1 equiv.) in diglyme (0.3 M) was heated to 95 °C and stirred overnight. Upon completion, the reaction was cooled down to room temperature, quenched with aqueous $NaHCO_3$, and ethyl acetate was added. Next, the aqueous layer was extracted with ethyl acetate. The combined organic layers were dried over anhydrous Na_2SO_4, filtered, and concentrated under reduced pressure. The crude residue was then purified by silica gel flash chromatography (eluent: *n*-heptane/EtOAc 4:1 *v/v*). Pure fractions were combined and concentrated under reduced pressure, yielding dibenzyl-protected DHP product **4**.

General Procedure E: Morpholine-Forming Amino-DHP Reagents (Step 4): iso-Propyl and tert-Butyl Hantzsch esters

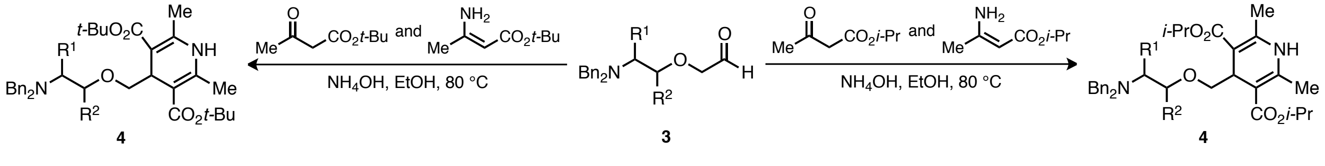

A mixture of aldehyde **3** (1.0 equiv.), *iso*-propyl acetoacetate (2.1 equiv.), and aqueous ammonium hydroxide (6.1 equiv.) in ethanol (0.3 M) was heated to 80 °C and stirred for 20 h. Upon completion, the reaction was cooled down to room temperature, quenched with aqueous $NaHCO_3$, and ethyl acetate was added. Next, the aqueous layer was extracted with ethyl acetate. The combined organic layers were dried over

anhydrous Na_2SO_4, filtered, and concentrated under reduced pressure. The crude residue was then purified by silica gel flash chromatography (eluent: *n*-heptane/EtOAc 4:1 *v/v*). Pure fractions were combined and concentrated under reduced pressure, yielding dibenzyl-protected DHP product **4**.

General Procedure F: Morpholine-Forming Amino-DHP Reagents (Step 5)

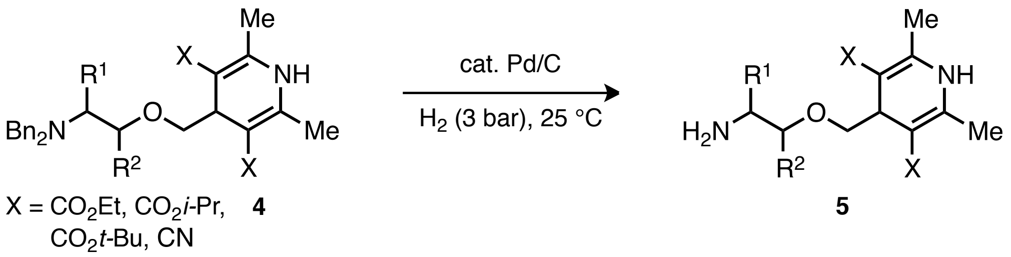

A suspension composed of dibenzyl-protected amino-DHP **4** (1.0 equiv.) and Pd/C (0.1 equiv.) in absolute ethanol (0.1 M) was evacuated and backfilled with hydrogen gas. The reaction mixture was then pressurized at 3 bar, and stirred at room temperature for 4-6 h. Upon completion, the mixture was filtered through a Celite plug, and the filtrate was concentrated under reduced pressure. Next, the crude product was purified by reverse-phase flash chromatography (C18, eluent: water/MeCN 3:2 *v/v* with 0.1% NH_4OH as modifier). Pure fractions were combined and evaporated under reduced pressure. Lyophilization yielded pure, dry amino-DHP reagent **5**.

General Procedure G: Thiomorpholine- and Piperazine-Forming Amino-DHP Reagents (Step 1)

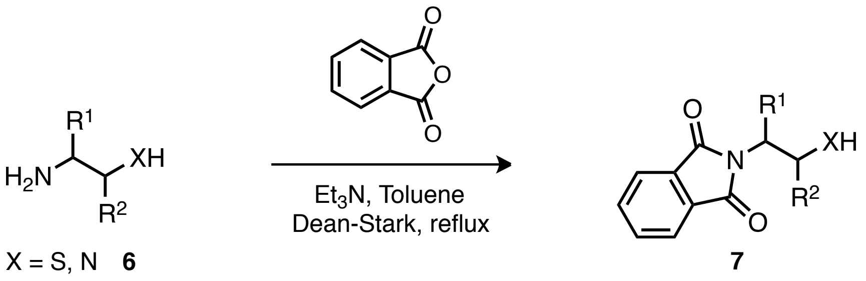

To a two-necked round-bottomed flask with a Dean-Stark apparatus attached were added: primary amine **6** (1.0 equiv.), Et_3N (3.5 equiv.), phthalic anhydride (1.0 equiv.), and anhydrous toluene (0.1 M). The reaction mixture was then refluxed for 2 days. Upon completion, the solution was cooled down to room temperature, and concentrated under reduced pressure. The crude residue was purified by silica gel flash chromatography (eluent: *n*-heptane/EtOAc 4:1 *v/v*). Pure fractions were combined and concentrated under reduced pressure, yielding phthalimide-protected amine **7**.

General Procedure H: Morpholine-Forming Amino-DHP Reagents: Pyrrolidino Amines

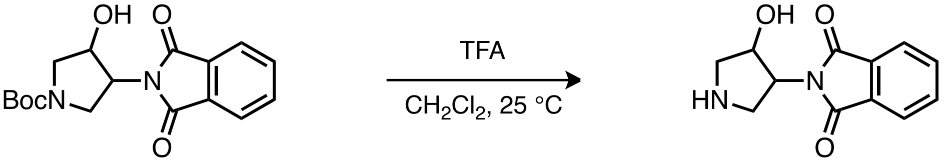

8 *cis* or *trans* → **9** *cis* or *trans*

To a cooled (0 °C) solution of Boc-protected pyrrolidino amine **8** (1.0 equiv.) in anhydrous CH_2Cl_2 (0.25 M) was added trifluoroacetic acid (50 equiv.) dropwise over 10 min. After an additional 15 min of stirring at 0 °C, the reaction mixture was warmed up to room temperature and stirred for 6 h. The mixture was then concentrated under reduced pressure and then lyophilized, yielding the trifluoroacetate salt of free pyrrolidine **9**.

General Procedure I: Thiomorpholine-Forming Amino-DHP Reagents (Step 2)

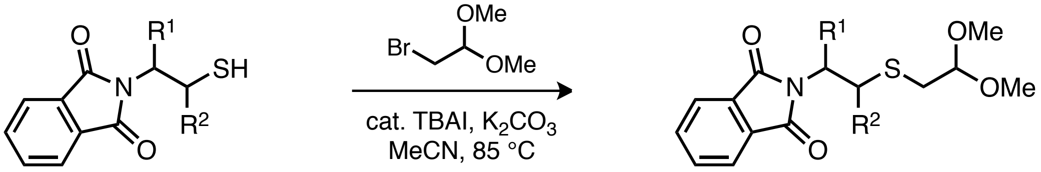

7 → **10**

To a solution composed of phthalimide-protected thiol **7** (1.0 equiv.), tetrabutylammonium iodide (0.1 equiv.), and potassium carbonate (2.05 equiv.) in anhydrous MeCN (0.5 M) was added 2-bromo-1,1-dimethoxyethane (2.05 equiv.). The reaction mixture was heated to reflux for 18-20 h, and then concentrated under reduced pressure. The crude residue was partitioned between ethyl acetate and water. The aqueous layer was extracted with ethyl acetate, and the combined organic layers were dried over anhydrous Na_2SO_4, filtered, and concentrated under reduced pressure. The crude product was purified by silica gel flash chromatography (eluent: *n*-heptane/EtOAc 4:1 *v/v*). Pure fractions were combined and concentrated under reduced pressure, yielding phthalimide-protected thioether acetal **10**.

General Procedure J: Piperazine-Forming Amino-DHP Reagents (Step 2)

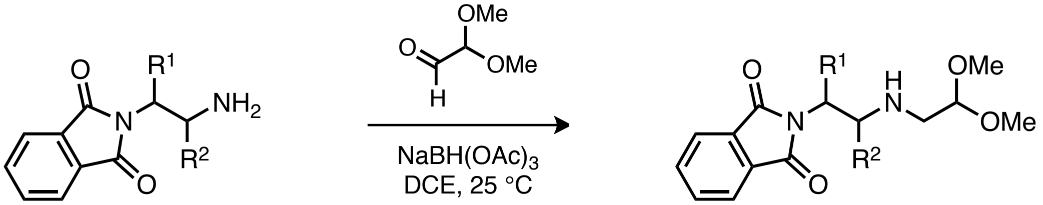

7 → **11**

To a suspension of phthalimide-protected primary amine **7** (1.0 equiv.) and sodium triacetoxyborohydride (2.1 equiv.) in 1,2-dichloroethane (0.3 M) was added 2,2-dimethoxyacetaldehyde (60% *w/w* aqueous solution, 1.05 equiv.). The resultant suspension was stirred vigorously at room temperature for 12-16 h. Upon completion, the reaction mixture was quenched with saturated aqueous $NaHCO_3$, and extracted with methylene

chloride. The combined organic layers were dried over anhydrous Na_2SO_4, filtered, and concentrated under reduced pressure. The crude residue was purified by silica gel flash chromatography (eluent: *n*-heptane/EtOAc 4:1 *v/v* first to remove the bis-alkylation byproduct, followed by CH_2Cl_2/MeOH 95:5 *v/v* to elute the desired mono-alkylation product). Pure fractions were combined and concentrated under reduced pressure, yielding phthalimide protected amino-acetal **11**.

General Procedure K: Piperazine-Forming Amino-DHP Reagents (Step 3): p-Tosyl Piperazines

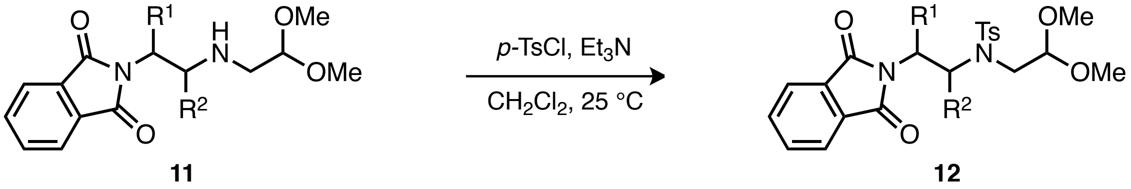

A solution of phthalimide protected amino-acetal **11** (1.0 equiv.) and Et_3N (1.3 equiv.) in anhydrous CH_2Cl_2 (0.3 M) was cooled down to 0 °C. Next, *p*-toluenesulfonyl chloride (1.3 equiv.) was added in one portion, and the resultant mixture was stirred at 0 °C for 15 min. The reaction mixture was then brought to room temperature and stirred for 14 h. The crude mixture was loaded directly onto silica gel and purified by silica gel flash chromatography (eluent: *n*-heptane/EtOAc 4:1 *v/v*). Pure fractions were combined and concentrated under reduced pressure, yielding *p*-tosyl protected amino-acetal **12**.

General Procedure L: Piperazine-Forming Amino-DHP Reagents (Step 3): Cbz Piperazines

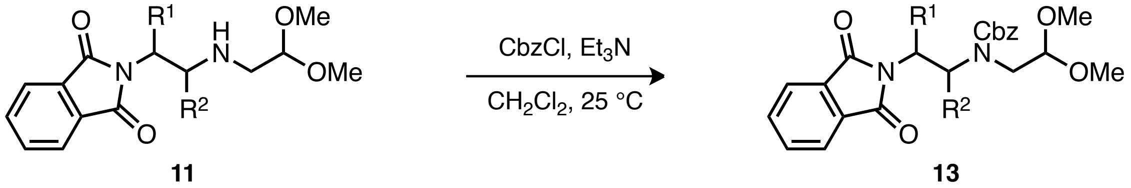

A solution of phthalimide protected amino-acetal **11** (1.0 equiv.) and Et_3N (2.1 equiv.) in anhydrous CH_2Cl_2 (0.6 M) was cooled down to −78 °C. Next, benzyl chloroformate (1.5 equiv.) was added dropwise over 10 min. The resultant mixture was stirred at −78 °C for 15 min, and then warmed up to room temperature. After stirring for a further 14 h, the solution was concentrated under reduced pressure, and the crude residue was loaded directly onto silica gel and purified by silica gel flash chromatography (eluent: *n*-heptane/EtOAc 3:2 *v/v*). Pure fractions were combined and concentrated under reduced pressure, yielding Cbz protected amino-acetal **13**.

General Procedure M: Thiomorpholine- and Piperazine-Forming Amino-DHP Reagents (Step 4)

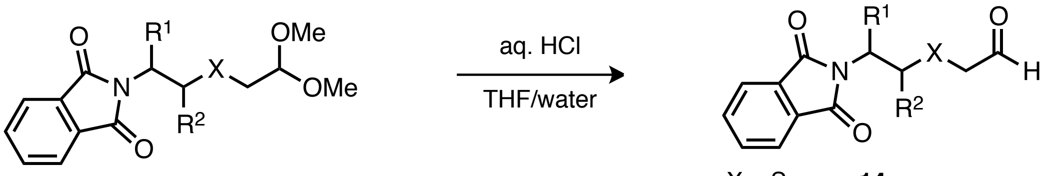

X = S **10**
X = NTs **12**
X = NCbz **13**

X = S **14**
X = NTs **15**
X = NCbz **16**

To a cooled (0 °C) solution of phthalimide-protected acetal **10**, **12**, or **13** (1.0 equiv.) in THF (0.3 M) was added aqueous 6 N HCl (10 equiv.). The reaction mixture was warmed up to room temperature and stirred for 7 h. Upon completion, the reaction was quenched with saturated aqueous $NaHCO_3$ at 0 °C, and methylene chloride was added. The aqueous layer was extracted with methylene chloride, dried over anhydrous Na_2SO_4, filtered, and concentrated under reduced pressure, yielding phthalimide-protected aldehyde product **14**, **15**, or **16**. The crude product was used in the next step as such, without further purification.

General Procedure N: Thiomorpholine- and Piperazine-Forming Amino-DHP Reagents (Step 5)

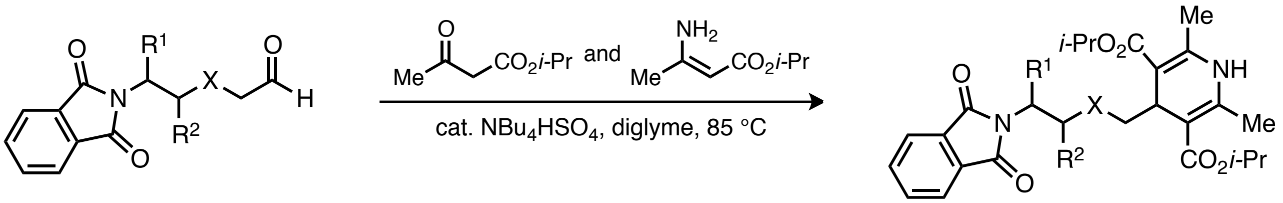

X = S **14**
X = NTs **15**
X = NCbz **16**

X = S **17**
X = NTs **18**
X = NCbz **19**

A mixture of phthalimide-protected aldehyde **14**, **15**, or **16** (1.0 equiv.), tetrabutylammonium hydrogensulfate (0.12 equiv.), *iso*-propyl acetoacetate (1.05 equiv.), *iso*-propyl 3-aminocrotonate (1.05 equiv.) in diglyme (0.3 M) was heated to 85 °C and stirred for 20 h. Upon completion, the reaction was cooled down to room temperature, quenched with aqueous $NaHCO_3$, and ethyl acetate was added. Next, the aqueous layer was extracted with ethyl acetate. The combined organic layers were dried over anhydrous Na_2SO_4, filtered, and concentrated under reduced pressure. The crude residue was then purified by silica gel flash chromatography (eluent: *n*-heptane/EtOAc 4:1 *v/v*). Pure fractions were combined and concentrated under reduced pressure, yielding phthalimide-protected DHP product **17**, **18**, or **19**.

General Procedure O: Thiomorpholine- and Piperazine-Forming Amino-DHP Reagents (Step 6)

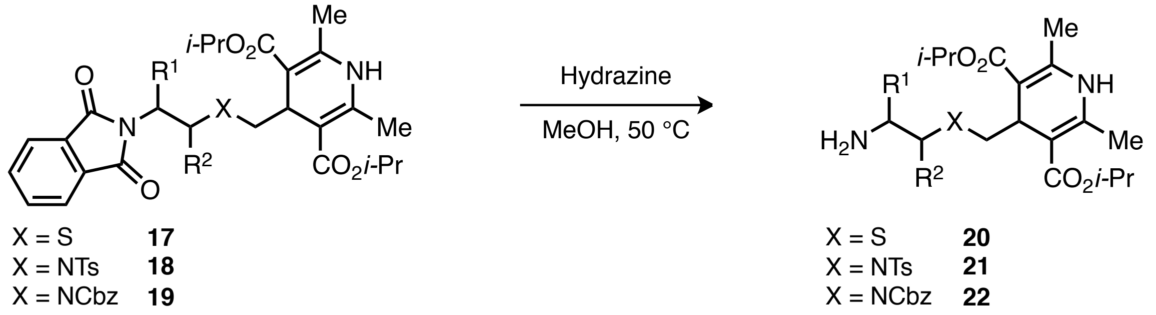

To a solution of phthalimide-protected DHP **17**, **18**, or **19** (1.0 equiv.) in absolute methanol (0.3 M) was added hydrazine (50% *w/w* aqueous solution, 10 equiv.). The reaction mixture was heated to 50 °C and stirred for 9-12 h. The mixture was then filtered through a Celite plug, and the filtrate was concentrated under reduced pressure. Next, the crude product was purified by reverse-phase flash chromatography (C18, eluent: water/MeCN 3:2 *v/v* with 0.1% NH_4OH as modifier). Pure fractions were combined and evaporated under reduced pressure. Lyophilization yielded pure, dry amino-DHP reagent **20**, **21**, or **22**.

General Procedure P: Metal Phosphate Salts

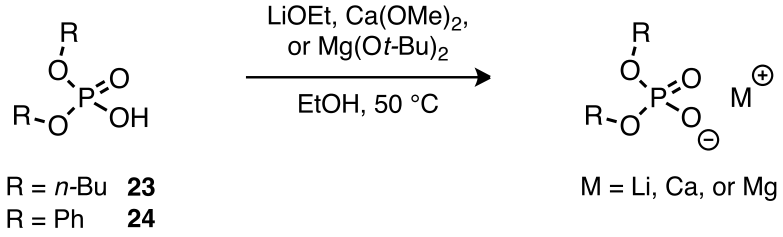

An oven-dried scintillation vial was charged with either dibutyl phosphate **23** or diphenyl phosphate **24** (1.0 equiv.) and absolute ethanol (0.5 M) under a nitrogen atmosphere. Next, either LiOEt (1.0 M solution in ethanol, 1.0 equiv.), $Ca(OMe)_2$ (1.0 equiv.), or $Mg(Ot\text{-}Bu)_2$ (1.0 equiv.) was added in one portion. The reaction mixture was then heated to 50 °C and stirred for 6 h. After cooling down to room temperature, the solution was concentrated under reduced pressure, yielding the various metal phosphate salts as off-white solids.

Characterization of Starting Materials

Complete List of Synthesized Amino-DHP Reagents

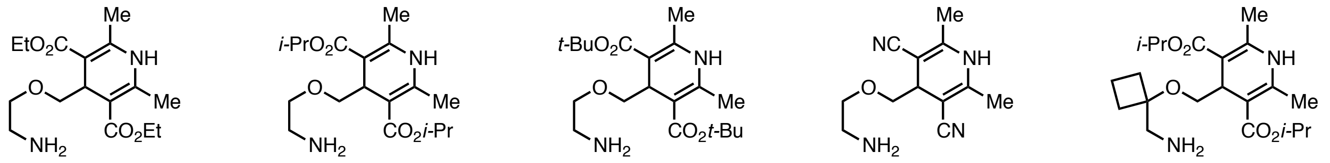

1a-CO_2Et **1a-CO_2*i*-Pr** **1a-CO_2*t*-Bu** **1a-CN** **1b**

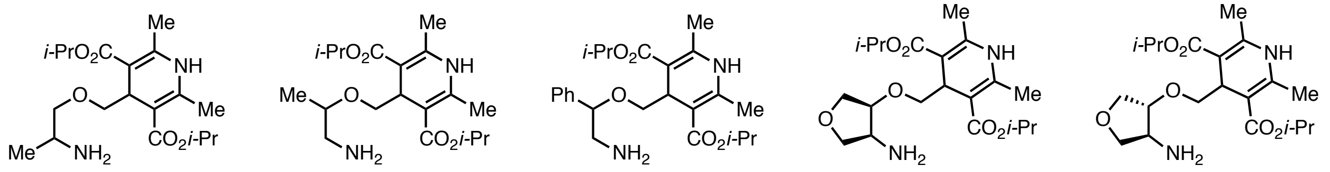

1c **1d** **1e** **1f** **1g**

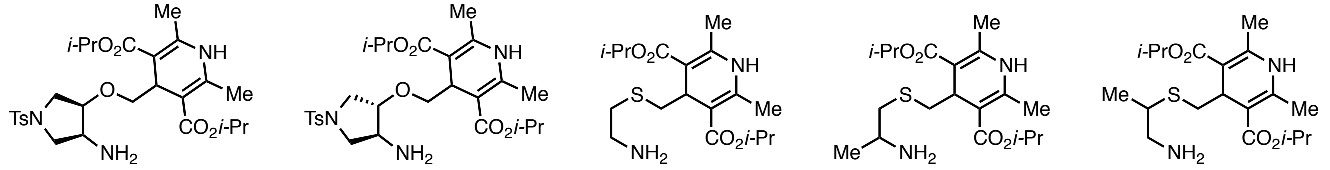

1h **1i** **1j** **1k** **1l**

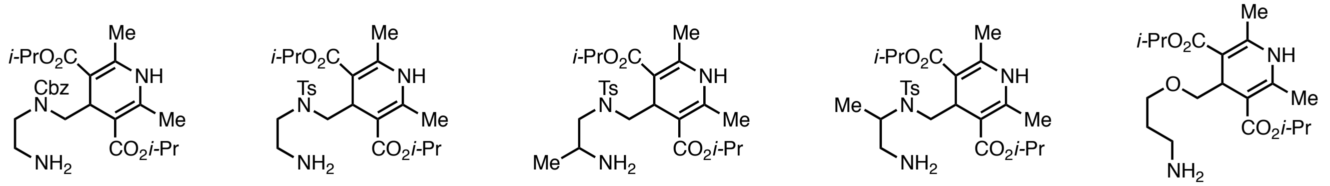

1m **1n** **1o** **1p** **1q**

Scheme S2. List of synthesized amino-DHP reagents.

Morpholine-Forming Amino-DHP Reagents

N,N-Dibenzyl-2-(2,2-dimethoxyethoxy)ethan-1-amine

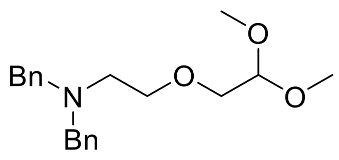

Synthesized according to General Procedure B. Purified by silica gel flash chromatography (eluent: *n*-heptane/EtOAc 4:1 *v/v*). Colorless oil. 95% yield. **^{1}H-NMR** (400 MHz, $CDCl_3$), δ, ppm: 2.71 (*t*, *J* 6.2 Hz, 2H), 3.38 (*s*, 6H), 3.44 (*d*, *J* 5.1 Hz, 2H), 3.58-3.62 (*m*, 2H), 3.65 (*s*, 4H), 4.47 (*t*, *J* 5.2 Hz, 1H), 7.20-7.26 (*m*, 2H), 7.28-7.34 (*m*, 4H), 7.36-7.40 (*m*, 4H).

2-(2-(Dibenzylamino)ethoxy)acetaldehyde

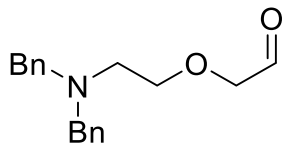

Synthesized according to General Procedure C. Used in the next step as such, without further purification. Colorless oil. 97% yield. **^{1}H-NMR** (400 MHz, $CDCl_3$), δ, ppm: 2.76 (*t*, *J* 5.9 Hz, 2H), 3.63 (*t*, *J* 5.9, 2H), 3.68 (*s*, 4H), 4.0 (*s*, 2H), 7.25 (*s*, 2H), 7.30-7.34 (*m*, 2H), 7.34-7.34 (*m*, 4H), 7.36-7.40 (*m*, 4H), 9.68 (*s*, 1H).

Diethyl 4-((2-(dibenzylamino)ethoxy)methyl)-2,6-dimethyl-1,4-dihydropyridine-3,5-dicarboxylate

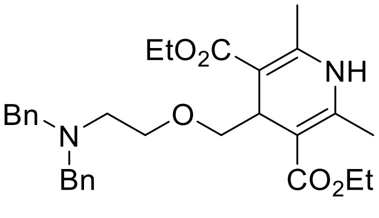

Synthesized according to General Procedure D. Purified by silica gel flash chromatography (eluent: *n*-heptane/EtOAc 3:2 *v/v*). Colorless oil. 75% yield. ^{1}**H-NMR** (400 MHz, $CDCl_3$), δ, ppm: 1.26 (*t*, *J* 7.1 Hz, 6H), 2.20 (*s*, 6H), 2.59 (*t*, *J* 5.9 Hz, 2H), 3.27 (*d*, *J* 5.0 Hz, 2H), 3.52 (*t*, *J* 5.8 Hz, 2H), 3.60 (*s*, 4H), 4.09-4.20 (*m*, 5H), 7.19-7.25 (*m*, 2H), 7.30 (*m*, 4H), 7.33-7.37 (*m*, 4H).

Diethyl 4-((2-aminoethoxy)methyl)-2,6-dimethyl-1,4-dihydropyridine-3,5-dicarboxylate (1a-CO$_2$Et)

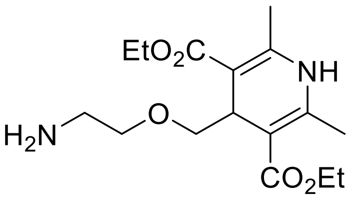

Synthesized according to General Procedure F. Purified by reverse-phase flash chromatography (C18, eluent: water/MeCN 3:2 *v/v* with 0.1% NH_4OH as modifier), then lyophilized. White fluffy solid. 86% yield. ^{1}**H-NMR** (400 MHz, $CDCl_3$), δ, ppm: 1.27 (*t*, *J* 7.1 Hz, 6H), 2.26 (*s*, 6H), 2.75 (*t*, *J* 5.0 Hz, 2H), 3.27 (*d*, *J* 5.8 Hz, 2H), 3.41 (*t*, *J* 5.0 Hz, 2H), 4.05-4.23 (*m*, 5H). ^{13}**C-NMR** (101 MHz, $CDCl_3$), δ, ppm: 14.4, 19.2, 34.2, 41.7, 59.7, 72.3, 74.2, 100.3, 145.6, 168.0. **MS**: *m/z* calcd. for $C_{16}H_{27}N_2O_5$ $[M+H]^+$ 327.2, found 327.4.

4-((2-Aminoethoxy)methyl)-2,6-dimethyl-1,4-dihydropyridine-3,5-dicarbonitrile (1a-CN)

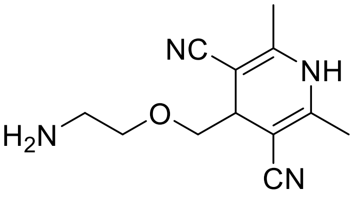

Synthesized according to General Procedures D and F. Purified by reverse-phase flash chromatography (C18, eluent: water/MeCN 4:1 *v/v* with 0.1% NH_4OH as modifier), then lyophilized. White fluffy solid. 94% yield (over two steps). ^{1}**H-NMR** (400 MHz, methanol-d_4), δ, ppm: 2.05 (*s*, 6H), 2.80 (*t*, *J* 5.1 Hz, 2H), 3.37-3.44 (*m*, 1 H), 3.47-3.57 (*m*, 4H). ^{13}**C-NMR** (101 MHz, methanol-d_4), δ, ppm: 18.2, 38.2, 42.2, 73.7, 75.1, 81.6, 120.6, 150.4. **MS**: *m/z* calcd. for $C_{12}H_{17}N_4O$ $[M+H]^+$ 233.1, found 233.2.

Diisopropyl 4-((2-aminoethoxy)methyl)-2,6-dimethyl-1,4-dihydropyridine-3,5-dicarboxylate (1a-CO$_2$i-Pr)

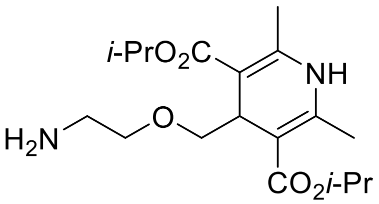

Synthesized according to General Procedures D and F. Purified by reverse-phase flash chromatography (C18, eluent: water/MeCN 1:1 *v/v* with 0.1% NH_4OH as modifier), then lyophilized. White fluffy solid. 87% yield (over two steps). ^{1}**H-NMR** (400 MHz, $CDCl_3$), δ, ppm: 1.27 (*d*, *J* 6.2 Hz, 12H), 2.26 (*s*, 6H), 2.75 (*t*, *J* 5.1 Hz, 2H), 3.29 (*d*, *J* 5.8 Hz, 2H), 3.42 (*t*, *J* 5.1 Hz, 2H), 4.18 (*t*, *J* 5.8 Hz, 1H), 5.06 (*sept.*, *J* 6.2 Hz, 2H). ^{13}**C-NMR** (101 MHz, $CDCl_3$), δ, ppm: 19.4, 22.0, 22.1, 34.3, 42.0, 66.9, 73.0, 74.2, 101.0, 144.9, 167.4. **MS**: *m/z* calcd. for $C_{18}H_{31}N_2O_5$ $[M+H]^+$ 355.2, found 355.4.

Di-tert-butyl 4-((2-aminoethoxy)methyl)-2,6-dimethyl-1,4-dihydropyridine-3,5-dicarboxylate (1a-CO$_2$t-Bu)

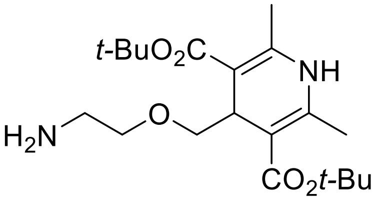

Synthesized according to General Procedures D and F. Purified by reverse-phase flash chromatography (C18, eluent: water/MeCN 1:1 *v/v* with 0.1%

NH_4OH as modifier), then lyophilized. White fluffy solid. 89% yield (over two steps). **^{1}H-NMR** (400 MHz, $CDCl_3$), δ, ppm: 1.51 (*s*, 18H), 2.25 (*s*, 6H), 2.79 (*t*, *J* 5.1 Hz, 2H), 3.28 (*d*, *J* 5.9 Hz, 2H), 3.46 (*t*, *J* 5.1 Hz, 2H), 4.14 (*t*, *J* 5.9 Hz, 1H). **^{13}C-NMR** (101 MHz, $CDCl_3$), δ, ppm: 19.5, 28.2, 28.4, 41.9, 72.9, 74.2, 79.6, 102.1, 144.1, 167.3. **MS**: *m/z* calcd. for $C_{20}H_{35}N_2O_5$ $[M+H]^+$ 383.3, found 383.6.

1-((Dibenzylamino)methyl)cyclobutan-1-ol

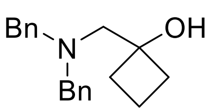

Synthesized according to General Procedure A. Purified by silica gel flash chromatography (eluent: *n*-heptane/EtOAc 4:1 *v/v*). Colorless oil. 88% yield. **^{1}H-NMR** (400 MHz, $CDCl_3$), δ, ppm: 1.44-1.57 (*m*, 1H), 1.75-1.87 (*m*, 1H), 2.0-2.08 (*m*, 2H), 2.10-2.19 (*m*, 2H), 2.71 (*s*, 2H), 3.66 (*s*, 4H), 4.04 (*s*, 1H), 7.27-7.38 (*m*, 10H).

N,N-Dibenzyl-1-(1-(2,2-dimethoxyethoxy)cyclobutyl)methanamine

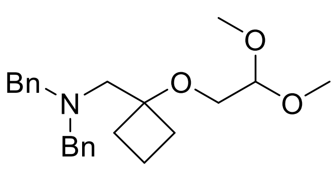

Synthesized according to General Procedure B. Purified by silica gel flash chromatography (eluent: *n*-heptane/EtOAc 4:1 *v/v*). Colorless oil. 75% yield. An inseparable mixture of product and starting material was obtained (*approx.* 1:1), separated in later steps. **^{1}H-NMR** (400 MHz, $CDCl_3$), δ, ppm: 1.11 (*dt*, *J* 11.1, 8.8 Hz, 1H), 1.58-1.64 (*m*, 1H), 2.06-2.18 (*m*, 4H), 2.62 (*s*, 2H), 3.37 (*s*, 6H), 3.41 (*d*, *J* 5.3 Hz, 2H), 3.67 (*d*, *J* 1.9 Hz, 6H), 4.44 (*t*, *J* 5.2 Hz, 1H), 7.33-7.44 (*m*, 10H).

Diisopropyl 4-((1-((dibenzylamino)methyl)cyclobutoxy)methyl)-2,6-dimethyl-1,4-dihydropyridine-3,5-dicarboxylate

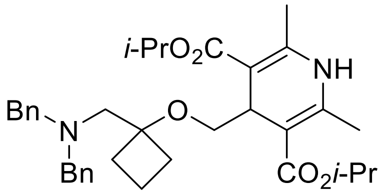

Synthesized according to General Procedures C and E. Purified by silica gel flash chromatography (eluent: *n*-heptane/EtOAc 4:1 *v/v*). Colorless oil. 30% yield (over two steps). **^{1}H-NMR** (400 MHz, $CDCl_3$), δ, ppm: 0.96-1.06 (*m*, 1H), 1.25 (*t*, *J* 6.5 Hz, 12H), 1.40-1.55 (*m*, 1H), 1.80-1.97 (*m*, 4H), 2.12 (*s*, 6H), 2.50 (*s*, 2H), 3.25 (*d*, *J* 4.4 Hz, 2H), 3.60 (*s*, 4H), 4.05 (*t*, *J* 4.2 Hz, 1H), 5.07 (*sept.*, *J* 6.2 Hz, 2H), 5.23 (*s*, 1H), 7.19-7.25 (*m*, 2H), 7.28-7.33 (*m*, 4H), 7.35-7.39 (*m*, 4H).

Diisopropyl 4-((1-(aminomethyl)cyclobutoxy)methyl)-2,6-dimethyl-1,4-dihydropyridine-3,5-dicarboxylate (1b)

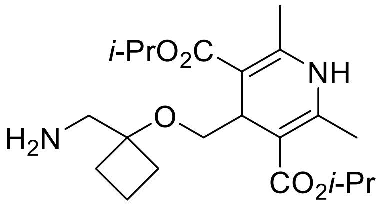

Synthesized according to General Procedure F. Purified by reverse-phase flash chromatography (C18, eluent: water/MeCN 1:1 *v/v* with 0.1% NH_4OH as modifier), then lyophilized. White fluffy solid. 65% yield. **^{1}H-NMR** (400 MHz, methanol-d_4), δ, ppm: 1.28 (*d*, *J* 6.2 Hz, 12H), 1.48-1.56 (*m*, 1H), 1.64-1.75 (*m*, 1H), 1.75-1.86 (*m*, 2H), 1.97-2.09 (*m*, 2H), 2.25 (*s*, 6H), 2.70 (*s*, 2H), 3.11 (*d*, *J* 5.8 Hz, 2H), 4.05 (*t*, *J* 5.8 Hz, 1H), 5.03 (*sept.*, *J* 6.2 Hz, 2H). **^{13}C-NMR** (101 MHz, methanol-d_4), δ, ppm: 13.2, 18.8, 22.5, 30.3, 36.3, 46.5, 68.5, 80.0, 100.9, 148.3, 169.9. **MS**: *m/z* calcd. for $C_{21}H_{35}N_2O_5$ $[M+H]^+$ 395.3, found 395.4.

(S)-N,N-Dibenzyl-1-(2,2-dimethoxyethoxy)propan-2-amine

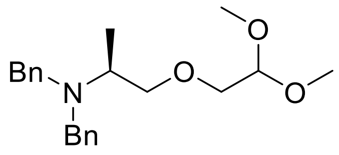

Synthesized according to General Procedures A and B. Purified by silica gel flash chromatography (eluent: *n*-heptane/EtOAc 4:1 *v/v*). Colorless oil. 64% yield (over two steps). ^{1}**H-NMR** (400 MHz, $CDCl_3$), *δ*, ppm: 1.08 (*d*, *J* 6.7 Hz, 3H), 2.99-3.09 (*m*, 1H), 3.39 (*s*, 6H), 3.41-3.47 (*m*, 3H), 3.56-3.62 (*m*, 2H), 3.63-3.69 (*m*, 1H), 3.70-3.76 (*m*, 2H), 4.49 (*t*, *J* 5.3 Hz, 1H), 7.18-7.24 (*m*, 2H), 7.27-7.32 (*m*, 4H), 7.39 (*d*, *J* 7.5 Hz, 4H).

(S)-2-(2-(Dibenzylamino)propoxy)acetaldehyde

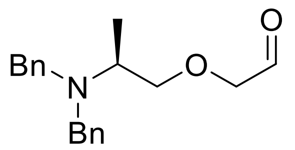

Synthesized according to General Procedure C. Used in the next step as such, without further purification. Colorless oil. 94% yield. ^{1}**H-NMR** (400 MHz, $CDCl_3$), *δ*, ppm: 1.12 (*d*, *J* 6.7 Hz, 3H), 3.05-3.13 (*m*, 1H), 3.48 (*dd*, *J* 9.7, 6.2 Hz, 1H), 3.60 (*d*, *J* 13.9 Hz, 2H), 3.69 (*dd*, *J* 9.7, 6.4 Hz, 1H), 3.72-3.78 (*m*, 2H), 3.99 (*s*, 2H), 7.20-7.24 (*m*, 2H), 7.30 (*t*, *J* 7.5 Hz, 4H), 7.38-7.41 (*m*, 4H), 9.72 (*s*, 1H).

Diisopropyl (S)-4-((2-(dibenzylamino)propoxy)methyl)-2,6-dimethyl-1,4-dihydropyridine-3,5-dicarboxylate

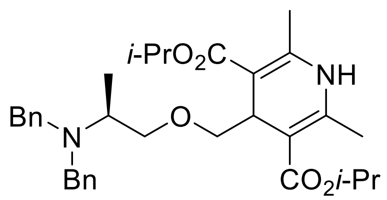

Synthesized according to General Procedure E. Purified by silica gel flash chromatography (eluent: *n*-heptane/EtOAc 4:1 *v/v*). Colorless oil. 43% yield. ^{1}**H-NMR** (400 MHz, $CDCl_3$), *δ*, ppm: 0.99-1.04 (*m*, 3H), 1.25 (*d*, *J* 6.2 Hz, 12H), 2.17-2.23 (*m*, 6H), 2.84-2.93 (*m*, 1H), 3.22-3.32 (*m*, 2H), 3.39-3.48 (*m*, 2H), 3.53-3.60 (*m*, 2H), 3.68 (*d*, *J* 13.9 Hz, 2H), 4.06-4.11 (*m*, 1H), 5.05 (*sept.*, *J* 6.2 Hz, 2H), 5.29-5.37 (*m*, 1H), 7.17-7.23 (*m*, 2H), 7.25-7.27 (*m*, 2H), 7.28-7.30 (*m*, 2H), 7.33-7.38 (*m*, 4H).

Diisopropyl (S)-4-((2-aminopropoxy)methyl)-2,6-dimethyl-1,4-dihydropyridine-3,5-dicarboxylate (1c)

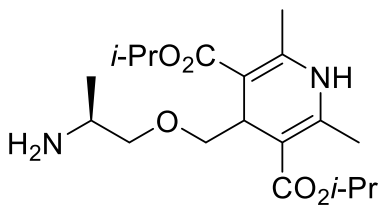

Synthesized according to General Procedure F. Purified by reverse-phase flash chromatography (C18, eluent: water/MeCN 1:1 *v/v* with 0.1% NH_4OH as modifier), then lyophilized. White fluffy solid. 97% yield. ^{1}**H-NMR** (400 MHz, $CDCl_3$), *δ*, ppm: 1.24 (*s*, 3H), 1.28 (*d*, *J* 6.2 Hz, 12H), 2.29 (*s*, 6H), 3.25-3.40 (*m*, 4H), 3.52 (*br. d*, *J* 6.4 Hz, 1H), 4.08 (*t*, *J* 5.1 Hz, 1H), 5.06 (*d*, *J* 6.2 Hz, 2H), 5.81-6.15 (*br. s*, 2H), 6.45 (*s*, 1H). ^{13}**C-NMR** (101 MHz, $CDCl_3$), *δ*, ppm: 16.6, 19.4, 22.1, 34.6, 47.7, 67.1, 73.8, 74.9, 100.3, 145.8, 167.8. **MS**: *m/z* calcd. for $C_{19}H_{33}N_2O_5$ $[M+H]^+$ 369.2, found 369.4.

(3R,4R)-4-(Dibenzylamino)tetrahydrofuran-3-ol

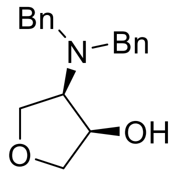

Synthesized according to General Procedure A (3 equiv. of K_2CO_3 were used). Purified by silica gel flash chromatography (eluent: *n*-heptane/EtOAc 4:1 *v/v*). Colorless oil. 85% yield.

^{1}**H-NMR** (400 MHz, $CDCl_3$), *δ*, ppm: 3.31 (*br. t*, *J* 9.8 Hz, 1H), 3.63-3.78 (*m*, 5H), 3.8 (*br. s*, 1H), 3.86-3.93 (*m*, 1H), 3.93-4.00 (*m*, 2H), 4.20 (*br. s*, 1H), 7.27-7.38 (*m*, 10H).

(3R,4R)-N,N-Dibenzyl-4-(2,2-dimethoxyethoxy)tetrahydrofuran-3-amine

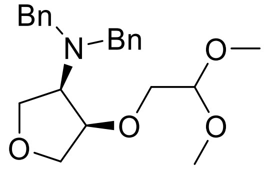

Synthesized according to General Procedure B. Purified by silica gel flash chromatography (eluent: *n*-heptane/EtOAc 4:1 *v/v*). Colorless oil. 96% yield. ^{1}**H-NMR** (400 MHz, $CDCl_3$), *δ*, ppm: 3.49 (*s*, 7H), 3.61 (*br. d*, *J* 5.1 Hz, 2H), 3.76-3.84 (*m*, 2H), 3.84 (*br. s*, 4H), 3.89-3.98 (*m*, 2H), 4.05-4.14 (*m*, 1H), 4.54 (*t*, *J* 5.1 Hz, 1H), 7.22-7.28 (*m*, 3H), 7.30-7.41 (*m*, 3H), 7.33 (*t*, *J* 7.5 Hz, 4H).

Diisopropyl 4-((((3R,4R)-4-(dibenzylamino)tetrahydrofuran-3-yl)oxy)methyl)-2,6-dimethyl-1,4-dihydropyridine-3,5-dicarboxylate

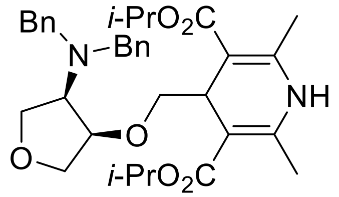

Synthesized according to General Procedures C and E (the aldehyde was not isolated due to instability). Purified by silica gel flash chromatography (eluent: *n*-heptane/EtOAc 4:1 *v/v*). Colorless oil. 37% yield (over two steps). ^{1}**H-NMR** (400 MHz, $CDCl_3$), *δ*, ppm: 1.24-1.29 (*m*, 12H), 2.19-2.26 (*m*, 6H), 3.20-3.29 (*m*, 1H), 3.30-3.36 (*m*, 1H), 3.51-3.57 (*m*, 1H), 3.68-3.77 (*m*, 3H), 3.78-3.81 (*m*, 4H), 3.88-4.00 (*m*, 1H), 4.10 (*t*, *J* 3.5 Hz, 1H), 5.09 (*sept.*, *J* 6.2 Hz, 2H), 5.32-5.39 (*m*, 1H), 7.18-7.25 (*m*, 2H), 7.27-7.32 (*m*, 4H), 7.32-7.37 (*m*, 4H).

Diisopropyl 4-((((3R,4R)-4-aminotetrahydrofuran-3-yl)oxy)methyl)-2,6-dimethyl-1,4-dihydropyridine-3,5-dicarboxylate (1f)

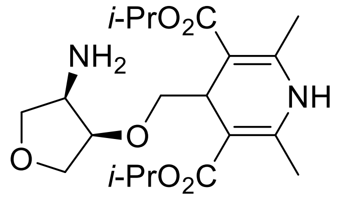

Synthesized according to General Procedure F. Purified by reverse-phase flash chromatography (C18, eluent: water/MeCN 3:2 *v/v* with 0.1% NH_4OH as modifier), then lyophilized. White fluffy solid. 95% yield. ^{1}**H-NMR** (400 MHz, $CDCl_3$), *δ*, ppm: 1.26-1.31 (*m*, 12H), 2.28 (*d*, *J* 3.3 Hz, 6H), 3.25-3.35 (*m*, 1H), 3.35-3.48 (*m*, 3H), 3.71-3.75 (*m*, 1H), 3.75-3.80 (*m*, 1H), 3.85-3.92 (*m*, 2H), 4.15 (*t*, *J* 5.0 Hz, 1H), 5.02-5.13 (*m*, 2H), 5.57 (*s*, 1H). ^{13}**C-NMR** (101 MHz, $CDCl_3$), *δ*, ppm: 19.4, 19.6, 22.1, 34.7, 54.0, 67.1, 71.4, 72.5, 72.9, 79.2, 100.6, 100.7, 145.2, 145.4, 167.3, 167.4. **MS**: *m/z* calcd. for $C_{20}H_{33}N_2O_6$ $[M+H]^+$ 397.2, found 397.3.

(R)-1-(Dibenzylamino)propan-2-ol

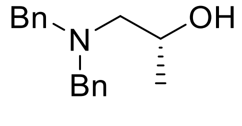

Synthesized according to General Procedure A. Purified by silica gel flash chromatography (eluent: *n*-heptane/EtOAc 4:1 *v/v*). Colorless oil. 92% yield. ^{1}**H-NMR** (400 MHz, $CDCl_3$), *δ*, ppm: 1.08 (*d*, *J* 6.1 Hz, 3H), 2.43 (*d*, *J* 6.6 Hz, 2H), 3.28 (*s*, 1H), 3.42 (*d*, *J* 13.5 Hz, 2H), 3.83-3.92 (*m*, 3H), 7.25-7.45 (*m*, 10H).

(R)-N,N-Dibenzyl-2-(2,2-dimethoxyethoxy)propan-1-amine

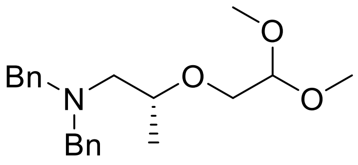

Synthesized according to General Procedure B. Purified by silica gel flash chromatography (eluent: *n*-heptane/EtOAc 4:1 *v/v*). Colorless oil. 67% yield. **^{1}H-NMR** (400 MHz, $CDCl_3$), *δ*, ppm: 1.12 (*d*, *J* 6.1 Hz, 3H), 2.44 (*dd*, *J* 13.2, 6.2 Hz, 1H), 2.61 (*dd*, *J* 13.2, 5.8 Hz, 1H), 3.37 (*s*, 6H), 3.43-3.64 (*m*, 5H), 3.65-3.74 (*m*, 2H), 4.45 (*t*, *J* 5.2 Hz, 1H), 7.20-7.26 (*m*, 2H), 7.28-7.34 (*m*, 4H), 7.34-7.39 (*m*, 4H).

(R)-2-((1-(Dibenzylamino)propan-2-yl)oxy)acetaldehyde

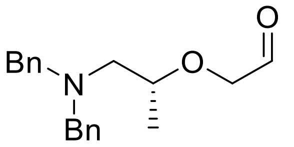

Synthesized according to General Procedure C. Used in the next step as such, without further purification. Colorless oil. 99% yield. **^{1}H-NMR** (400 MHz, $CDCl_3$), *δ*, ppm: 1.16 (*d*, *J* 6.2 Hz, 3H), 2.43-2.54 (*m*, 1H), 2.71 (*dd*, *J* 13.4, 6.2 Hz, 1H), 3.58-3.62 (*m*, 1H), 3.65 (*s*, 4H), 4.08 (*d*, *J* 3.2 Hz, 2H), 7.28-7.41 (*m*, 10H), 9.69 (*s*, 1H).

Diisopropyl (R)-4-(((1-(dibenzylamino)propan-2-yl)oxy)methyl)-2,6-dimethyl-1,4-dihydropyridine-3,5-dicarboxylate

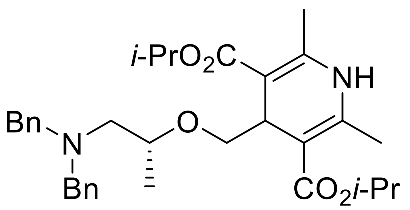

Synthesized according to General Procedure E. Purified by silica gel flash chromatography (eluent: *n*-heptane/EtOAc 4:1 *v/v*). Colorless oil. 71% yield. **^{1}H-NMR** (400 MHz, $CDCl_3$), *δ*, ppm: 0.95 (*d*, *J* 6.1 Hz, 3H), 1.14-1.18 (*m*, 12H), 2.10 (*s*, 6H), 2.25-2.39 (*m*, 2H), 3.24 (*d*, *J* 4.8 Hz, 2H), 3.37-3.42 (*m*, 1H), 3.42-3.54 (*m*, 4H), 3.97 (*t*, *J* 4.7 Hz, 1H), 4.91-5.02 (*m*, 2H), 5.19-5.23 (*m*, 1H), 7.11-7.16 (*m*, 2H), 7.19-7.22 (*m*, 4H), 7.24-7.29 (*m*, 4H).

Diisopropyl (R)-4-(((1-aminopropan-2-yl)oxy)methyl)-2,6-dimethyl-1,4-dihydropyridine-3,5-dicarboxylate (1d)

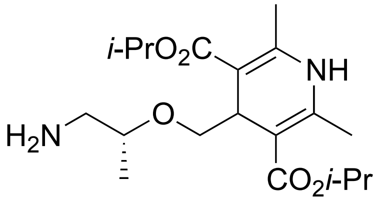

Synthesized according to General Procedure F. Purified by reverse-phase flash chromatography (C18, eluent: water/MeCN 1:1 *v/v* with 0.1% NH_4OH as modifier), then lyophilized. White fluffy solid. 66% yield. **^{1}H-NMR** (400 MHz, $CDCl_3$), *δ*, ppm: 1.04 (*d*, *J* 6.1 Hz, 3H), 1.29 (*br. d*, *J* 6.1 Hz, 12H), 2.28 (*s*, 6H), 2.50-2.58 (*m*, 1H), 2.66 (*dd*, *J* 13.2, 3.2 Hz, 1H), 3.19 (*dd*, *J* 9.1, 5.8 Hz, 1H), 3.26-3.36 (*m*, 1H), 3.43 (*dd*, *J* 9.1, 5.2 Hz, 1H), 4.15 (*t*, *J* 5.4 Hz, 1H), 5.09 (*dt*, *J* 12.2, 6.0 Hz, 2H), 5.64 (*br. s*, 1H). **^{13}C-NMR** (101 MHz, $CDCl_3$), *δ*, ppm: 16.9, 19.4, 19.5, 22.0, 22.1, 35.0, 47.6, 66.9, 71.6, 77.2, 100.9, 101.3, 144.6, 145.0, 167.4, 167.6. **MS**: *m/z* calcd. for $C_{19}H_{33}N_2O_5$ $[M+H]^+$ 369.2, found 369.3.

(3S,4R)-4-(Dibenzylamino)tetrahydrofuran-3-ol

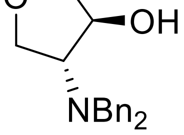

Synthesized according to General Procedure A. Purified by silica gel flash chromatography (eluent: *n*-heptane/EtOAc 4:1 *v/v*). Colorless oil. 94% yield. **^{1}H-NMR** (400 MHz, $CDCl_3$), *δ*,

ppm: 1.25-1.38 (*m*, 1H), 3.40 (*br. t*, *J* 7.6 Hz, 1H), 3.63-3.80 (*m*, 5H), 3.82-3.88 (*m*, 1H), 3.99-4.09 (*m*, 2H), 4.54 (*br. s*, 1H), 7.28-7.43 (*m*, 10H).

(3R,4S)-N,N-Dibenzyl-4-(2,2-dimethoxyethoxy)tetrahydrofuran-3-amine

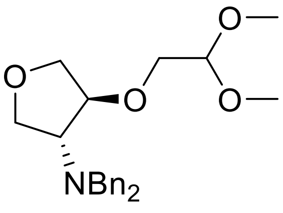

Synthesized according to General Procedure B. Purified by silica gel flash chromatography (eluent: *n*-heptane/EtOAc 4:1 *v/v*). Colorless oil. 86% yield. **^{1}H-NMR** (400 MHz, $CDCl_3$), *δ*, ppm: 3.36-3.44 (*m*, 6H), 3.45-3.52 (*m*, 3H), 3.68-3.73 (*m*, 5H), 3.90-3.97 (*m*, 2H), 4.08 (*dd*, *J* 9.9, 6.1 Hz, 1H), 4.23-4.28 (*m*, 1H), 4.47 (*t*, *J* 5.2 Hz, 1H), 7.28-7.44 (*m*, 10H).

2-(((3S,4R)-4-(Dibenzylamino)tetrahydrofuran-3-yl)oxy)acetaldehyde

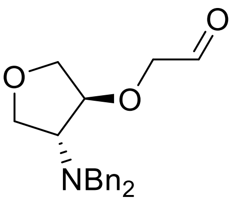

Synthesized according to General Procedure C. Used in the next step as such, without further purification. Colorless oil. 99% yield. **^{1}H-NMR** (400 MHz, $CDCl_3$), *δ*, ppm: 3.45-3.50 (*m*, 1H), 3.60-3.73 (*m*, 5H), 3.87-4.08 (*m*, 5H), 4.19-4.24 (*m*, 1H), 7.25-7.38 (*m*, 10H), 9.60 (*s*, 1H).

Diisopropyl 4-((((3S,4R)-4-(dibenzylamino)tetrahydrofuran-3-yl)oxy)methyl)-2,6-dimethyl-1,4-dihydropyridine-3,5-dicarboxylate

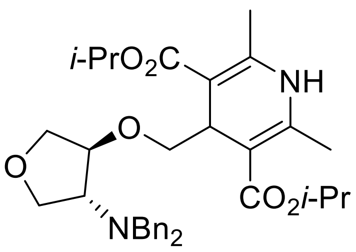

Synthesized according to General Procedure E. Purified by silica gel flash chromatography (eluent: *n*-heptane/EtOAc 4:1 *v/v*). Colorless oil. 53% yield. **^{1}H-NMR** (400 MHz, $CDCl_3$), *δ*, ppm: 1.26-1.36 (*m*, 12H), 2.12-2.35 (*m*, 6H), 3.15-3.27 (*m*, 1H), 3.30 (*br. dd*, *J* 9.2, 4.7 Hz, 2H), 3.56-3.77 (*m*, 2H), 3.80-3.97 (*m*, 2H), 4.02-4.09 (*m*, 1H), 4.11-4.22 (*m*, 2H), 5.10 (*dq*, *J* 15.9, 6.2 Hz, 2H), 5.30 (*s*, 1H), 7.26-7.41 (*m*, 10H).

Diisopropyl 4-((((3S,4R)-4-aminotetrahydrofuran-3-yl)oxy)methyl)-2,6-dimethyl-1,4-dihydropyridine-3,5-dicarboxylate (1g)

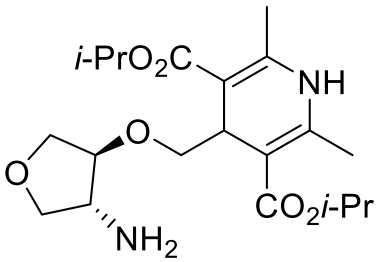

Synthesized according to General Procedure F. Purified by reverse-phase flash chromatography (C18, eluent: water/MeCN 1:1 *v/v* with 0.1% NH_4OH as modifier), then lyophilized. White fluffy solid. 96% yield. **^{1}H-NMR** (400 MHz, $CDCl_3$), *δ*, ppm: 1.25-1.30 (*m*, 12H), 2.26 (*s*, 6H), 3.27-3.31 (*m*, 2H), 3.31-3.35 (*m*, 1H), 3.51 (*dd*, *J* 8.9, 2.1 Hz, 1H), 3.62 (*dd*, *J* 9.9, 2.0 Hz, 1H), 3.7 (*br. d*, *J* 4.9 Hz, 1H), 3.83-3.91 (*m*, 1H), 3.96-4.05 (*m*, 1H), 4.17 (*t*, *J* 5.6 Hz, 1H), 5.06 (*dq*, *J* 12.2, 6.2 Hz, 2H), 5.57 (*s*, 1H). **^{13}C-NMR** (101 MHz, $CDCl_3$), *δ*, ppm: 19.5, 19.6, 22.1, 34.2, 57.0, 67.0, 66.9, 69.1, 71.9, 72.1, 74.1, 86.6, 100.76, 100.7, 144.9, 145.0, 167.3, 167.4. **MS**: *m/z* calcd. for $C_{20}H_{33}N_2O_6$ $[M+H]^+$ 397.2, found 397.3.

(S)-2-(2-(2,2-Dimethoxyethoxy)-2-phenylethyl)isoindoline-1,3-dione

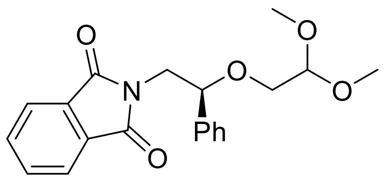

Synthesized according to General Procedures G and B. Purified by silica gel flash chromatography (eluent: *n*-heptane/EtOAc 4:1 *v/v*). Colorless oil. 44% yield (over two steps). **^{1}H-NMR** (400 MHz, $CDCl_3$), *δ*, ppm: 3.13-3.21 (*m*, 6H), 3.21-3.27 (*m*, 1H), 3.41-3.48 (*m*, 1H), 3.77 (*dd*, *J* 13.9, 4.8 Hz, 1H), 4.07 (*dd*, *J* 13.9, 9.1 Hz, 1H), 4.35 (*t*, *J* 5.3 Hz, 1H), 4.79 (*dd*, *J* 9.1, 4.7 Hz, 1H), 7.29-7.34 (*m*, 1H), 7.34-7.40 (*m*, 2H), 7.41-7.46 (*m*, 2H), 7.72 (*dd*, *J* 5.5, 3.1 Hz, 2H), 7.82-7.90 (*m*, 2H).

(S)-2-(2-(1,3-Dioxoisoindolin-2-yl)-1-phenylethoxy)acetaldehyde

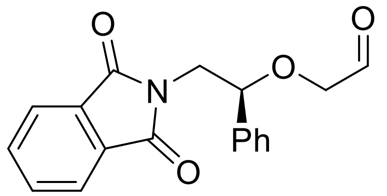

Synthesized according to General Procedure M. Used in the next step as such, without further purification. Colorless oil. 97% yield. **^{1}H-NMR** (400 MHz, $CDCl_3$), *δ*, ppm: 3.70-3.84 (*m*, 2H), 3.89-3.97 (*m*, 1H), 4.06-4.15 (*m*, 1H), 4.74 (*dd*, *J* 9.2, 4.3 Hz, 1H), 7.26-7.39 (*m*, 5H), 7.63-7.70 (*m*, 2H), 7.77-7.83 (*m*, 2H), 9.47 (*s*, 1H).

Diisopropyl (S)-4-((2-(1,3-dioxoisoindolin-2-yl)-1-phenylethoxy)methyl)-2,6-dimethyl-1,4-dihydropyridine-3,5-dicarboxylate

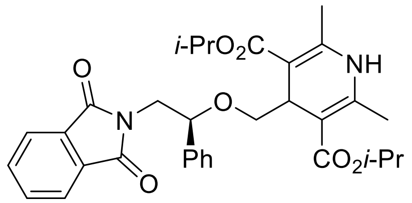

Synthesized according to General Procedure N. Purified by silica gel flash chromatography (eluent: *n*-heptane/EtOAc 4:1 *v/v*). Colorless oil. 59% yield. **^{1}H-NMR** (400 MHz, $CDCl_3$), *δ*, ppm: 1.03-1.20 (*m*, 12H), 2.06 (*s*, 3H), 2.33 (*s*, 3H), 3.09 (*dd*, *J* 9.1, 2.9 Hz, 1H), 3.22 (*dd*, *J* 9.1, 3.6 Hz, 1H), 3.62 (*dd*, *J* 13.8, 4.3 Hz, 1H), 3.87 (*t*, *J* 3.1 Hz, 1H), 3.95 (*dd*, *J* 13.8, 9.5 Hz, 1H), 4.66 (*dd*, *J* 9.5, 4.3 Hz, 1H), 4.83-5.00 (*m*, 2H), 5.47 (*s*, 1H), 7.28-7.34 (*m*, 5H), 7.74 (*dd*, *J* 5.4, 3.0 Hz, 2H), 7.85 (*dd*, *J* 5.4, 3.1 Hz, 2H).

Diisopropyl (S)-4-((2-amino-1-phenylethoxy)methyl)-2,6-dimethyl-1,4-dihydropyridine-3,5-dicarboxylate (1e)

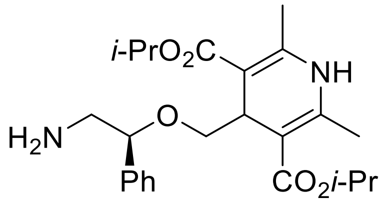

Synthesized according to General Procedure O. Purified by reverse-phase flash chromatography (C18, eluent: water/MeCN 1:1 *v/v* with 0.1% NH_4OH as modifier), then lyophilized. White fluffy solid. 73% yield. **^{1}H-NMR** (400 MHz, methanol-*d_4*), *δ*, ppm: 1.16-1.29 (*m*, 12H), 2.25 (*d*, *J* 4.8 Hz, 6H), 2.65 (*d*, *J* 6.2 Hz, 2H), 3.02-3.12 (*m*, 1H), 3.17 (*dd*, *J* 8.4, 5.8 Hz, 1H), 4.11 (*t*, *J* 5.6 Hz, 1H), 4.16 (*t*, *J* 6.2 Hz, 1H), 5.0 (*dsept.*, *J* 9.8, 6.2 Hz, 2H), 7.19-7.34 (*m*, 5H). **^{13}C-NMR** (101 MHz, methanol-*d_4*), *δ*, ppm: 18.7, 18.9, 22.46, 22.54, 36.1, 49.9, 68.3, 68.4, 73.3, 84.5, 100.7, 101.1, 127.8, 128.9, 129.6, 142.0, 148.2, 148.5, 169.76, 169.84. **MS**: *m/z* calcd. for $C_{24}H_{35}N_2O_5$ $[M+H]^+$ 431.3, found 431.4.

tert-Butyl (3R,4S)-3-(1,3-dioxoisoindolin-2-yl)-4-hydroxypyrrolidine-1-carboxylate

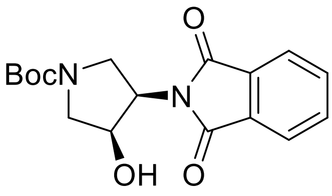

Synthesized according to General Procedure G. Purified by silica gel flash chromatography (eluent: *n*-heptane/EtOAc 3:2 *v/v*, followed by CH_2Cl_2/MeOH 95:5). White solid. 78% yield. **^{1}H-NMR** (400 MHz, $CDCl_3$), δ, ppm: 1.48 (*s*, 9H), 2.29 (*br. s*, 1H), 3.29 (*dd*, *J* 11.1, 7.3 Hz, 1H), 3.70 (*dd*, *J* 10.6, 9.4 Hz, 1H), 3.94 (*br. s*, 2H), 4.62 (*br. d*, *J* 8.3 Hz, 1H), 4.97-5.06 (*m*, 1H), 7.73-7.78 (*m*, 2H), 7.84-7.89 (*m*, 2H).

2-((3R,4S)-4-Hydroxypyrrolidin-3-yl)isoindoline-1,3-dione, TFA Salt

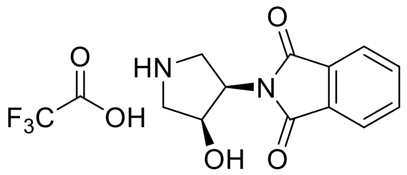

Synthesized according to General Procedure H. Used in the next step as such, without further purification. Colorless oil. 99% yield. **^{1}H-NMR** (400 MHz, methanol-d_4), δ, ppm: 3.36 (*br. d*, *J* 12.4 Hz, 1H), 3.70 (*dd*, *J* 12.2, 4.3 Hz, 1H), 3.80-3.92 (*m*, 2H), 4.51-4.56 (*m*, 1H), 4.83 (*dt*, *J* 3.9, 1.9 Hz, 1H), 7.82-7.87 (*m*, 2H), 7.87-7.92 (*m*, 2H).

2-((3R,4S)-4-(2,2-Dimethoxyethoxy)-1-tosylpyrrolidin-3-yl)isoindoline-1,3-dione

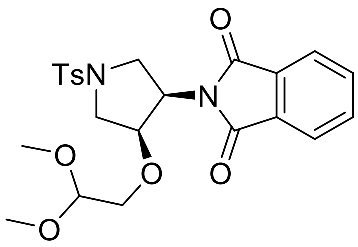

Synthesized according to General Procedures K and B. Purified by silica gel flash chromatography (eluent: *n*-heptane/EtOAc 3:2 *v/v*). Colorless oil. 28% yield (over two steps). **^{1}H-NMR** (400 MHz, $CDCl_3$), δ, ppm: 2.46 (*s*, 3H), 3.26 (*s*, 3H), 3.30 (*s*, 3H), 3.35 (*dd*, *J* 10.3, 4.9 Hz, 1H), 3.39-3.46 (*m*, 3H), 3.64-3.73 (*m*, 2H), 4.33 (*t*, *J* 5.1 Hz, 1H), 4.58-4.71 (*m*, 2H), 7.36 (*d*, *J* 8.0 Hz, 2H), 7.71-7.77 (*m*, 4H), 7.80-7.86 (*m*, 2H).

2-(((3S,4R)-4-(1,3-Dioxoisoindolin-2-yl)-1-tosylpyrrolidin-3-yl)oxy)acetaldehyde

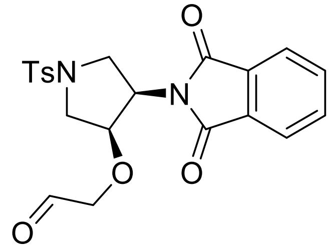

Synthesized according to General Procedure M. Used in the next step as such, without further purification. Colorless oil. 93% yield. **^{1}H-NMR** (400 MHz, $CDCl_3$), δ, ppm: 2.47 (*s*, 3H), 3.40-3.48 (*m*, 2H), 3.69-3.76 (*m*, 2H), 4.04 (*s*, 2H), 4.60 (*q*, *J* 6.2 Hz, 1H), 4.75 (*td*, *J* 8.7, 5.9 Hz, 1H), 7.37 (*d*, *J* 8.0 Hz, 2H), 7.72-7.78 (*m*, 4H), 7.81-7.87 (*m*, 2H), 9.54 (*s*, 1H).

Diisopropyl 4-((((3S,4R)-4-(1,3-dioxoisoindolin-2-yl)-1-tosylpyrrolidin-3-yl)oxy)methyl)-2,6-dimethyl-1,4-dihydropyridine-3,5-dicarboxylate

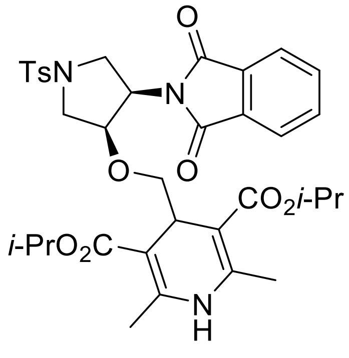

Synthesized according to General Procedure N. Purified by silica gel flash chromatography (eluent: *n*-heptane/EtOAc 3:2 *v/v*). Colorless oil. 66% yield. **^{1}H-NMR** (400 MHz, $CDCl_3$), δ, ppm: 1.09-1.26 (*m*, 12H), 2.05-2.30 (*m*, 6H), 2.44-2.48 (*m*, 3H), 3.18 (*dd*, *J* 9.4, 4.4 Hz, 1H), 3.26 (*td*, *J* 10.2, 4.5 Hz, 2H), 3.37 (*t*, *J* 9.1 Hz, 1H), 3.55-3.68 (*m*, 2H), 3.96 (*t*, *J* 4.4 Hz, 1H), 4.46-4.51 (*m*,

1H), 4.52-4.62 (*m*, 1H), 4.86 (*quint.*, *J* 6.3 Hz, 1H), 5.02 (*quint.*, *J* 6.2 Hz, 1H), 5.43 (*s*, 1H), 7.34 (*d*, *J* 8.1 Hz, 2H), 7.69-7.76 (*m*, 4H), 7.81 (*br. dd*, *J* 5.4, 3.1 Hz, 2H).

Diisopropyl 4-((((3S,4R)-4-amino-1-tosylpyrrolidin-3-yl)oxy)methyl)-2,6-dimethyl-1,4-dihydropyridine-3,5-dicarboxylate (1h)

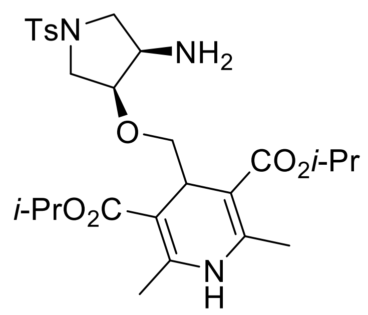

Synthesized according to General Procedure O. Purified by reverse-phase flash chromatography (C18, eluent: water/MeCN 1:1 *v/v* with 0.1% NH_4OH as modifier), then lyophilized. White fluffy solid. 76% yield. **^{1}H-NMR** (400 MHz, $CDCl_3$), *δ*, ppm: 1.25 (*t*, *J* 5.3 Hz, 12H), 2.23 (*s*, 6H), 2.43 (*s*, 3H), 2.99 (*dd*, *J* 10, 3.9 Hz, 1H), 3.10 (*dd*, *J* 10.5, 3.2 Hz, 1H), 3.15-3.27 (*m*, 3H), 3.39 (*dd*, *J* 10.0, 5.8 Hz, 1H), 3.49 (*dd*, *J* 10.5, 5.5 Hz, 1H), 3.54-3.59 (*m*, 1H), 4.07 (*t*, *J* 5.3 Hz, 1H), 4.98-5.08 (*m*, 2H), 5.53 (*s*, 1H), 7.32 (*d*, *J* 8.1 Hz, 2H), 7.70 (*d*, *J* 8.2 Hz, 2H). **^{13}C-NMR** (101 MHz, $CDCl_3$), *δ*, ppm: 19.4, 19.6, 21.5, 22.1, 34.3, 51.2, 53.5, 55.6, 67.00, 67.02, 72.4, 84.3, 100.4, 100.5, 127.5, 129.6, 133.9, 143.4, 145.1, 145.2, 167.2, 167.3. **MS**: *m/z* calcd, for $C_{27}H_{40}N_3O_7S$ $[M+H]^+$ 550.3, found 550.5.

tert-Butyl (3S,4S)-3-(1,3-dioxoisoindolin-2-yl)-4-hydroxypyrrolidine-1-carboxylate

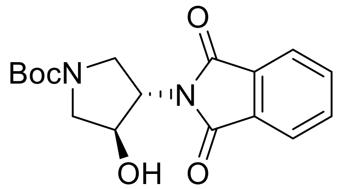

Synthesized according to General Procedure G. Purified by silica gel flash chromatography (eluent: *n*-heptane/EtOAc 3:2 *v/v*, followed by CH_2Cl_2/MeOH 95:5). White solid. 95% yield. **^{1}H-NMR** (400 MHz, methanol-*d_4*), *δ*, ppm: 1.49 (*d*, *J* 8.6 Hz, 9H), 3.18-3.25 (*m*, 1H), 3.68-3.85 (*m*, 3H), 4.60-4.69 (*m*, 1H), 4.75-4.85 (*m*, 1H), 7.80-7.85 (*m*, 2H), 7.85-7.90 (*m*, 2H).

2-((3S,4S)-4-Hydroxy-1-tosylpyrrolidin-3-yl)isoindoline-1,3-dione

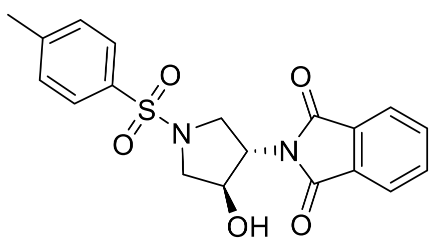

Synthesized according to General Procedures H and K. Purified by silica gel flash chromatography (eluent: *n*-heptane/EtOAc 3:2 *v/v*). Colorless oil. 64% yield (over two steps). **^{1}H-NMR** (400 MHz, $CDCl_3$), *δ*, ppm: 2.47 (*s*, 3H), 3.27 (*dd*, *J* 10.3, 5.9 Hz, 1H), 3.44-3.51 (*m*, 1H), 3.74 (*dd*, *J* 10.3, 7.0 Hz, 1H), 3.85 (*t*, *J* 9.4 Hz, 1H), 4.44-4.54 (*m*, 1H), 4.86 (*q*, *J* 6.6 Hz, 1H), 7.37 (*d*, *J* 8.1 Hz, 2H), 7.73-7.78 (*m*, 4H), 7.80-7.85 (*m*, 2H).

2-((3S,4S)-4-(2,2-Dimethoxyethoxy)-1-tosylpyrrolidin-3-yl)isoindoline-1,3-dione

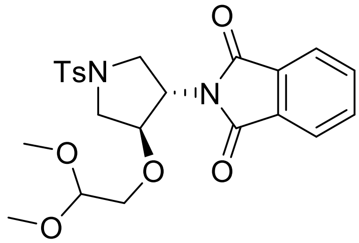

Synthesized according to General Procedure B. Purified by silica gel flash chromatography (eluent: *n*-heptane/EtOAc 3:2 *v/v*, followed by CH_2Cl_2/MeOH 95:5). White solid. 38% yield. **^{1}H-NMR** (400 MHz, $CDCl_3$), *δ*, ppm: 2.46 (*s*, 3H), 3.26 (*s*, 3H), 3.30 (*s*, 3H), 3.35 (*dd*, *J* 10.4, 4.9 Hz, 1H), 3.38-3.46 (*m*,

3H), 3.64-3.73 (*m*, 2H), 4.33 (*t*, *J* 5.1 Hz, 1H), 4.58-4.72 (*m*, 2H), 7.36 (*d*, *J* 8.1 Hz, 2H), 7.71-7.77 (*m*, 4H), 7.79-7.86 (*m*, 2H).

Diisopropyl 4-((((3S,4S)-4-(1,3-dioxoisoindolin-2-yl)-1-tosylpyrrolidin-3-yl)oxy)methyl)-2,6-dimethyl-1,4-dihydropyridine-3,5-dicarboxylate

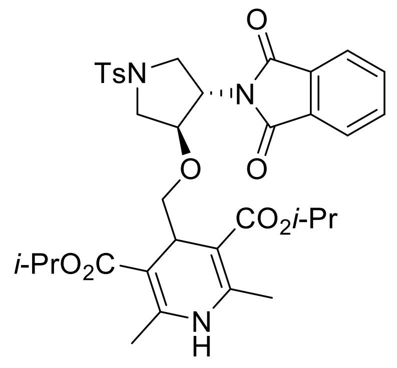

Synthesized according to General Procedures M and N. Purified by silica gel flash chromatography (eluent: *n*-heptane/EtOAc 3:2 *v/v*). Colorless oil. 68% yield (over two steps). **^{1}H-NMR** (400 MHz, $CDCl_3$), *δ*, ppm: 1.15 (*dd*, *J* 15.1, 6.2 Hz, 6H), 1.25 (*dd*, *J* 6.2, 2.5 Hz, 6H), 2.10 (*s*, 3H), 2.23 (*s*, 3H), 2.45 (*s*, 3H), 3.18 (*dd*, *J* 9.5, 4.5 Hz, 1H), 3.26 (*td*, *J* 10.3, 4.5 Hz, 2H), 3.37 (*t*, *J* 9.1 Hz, 1H), 3.56-3.66 (*m*, 2H), 3.96 (*t*, *J* 4.3 Hz, 1H), 4.46-4.52 (*m*, 1H), 4.53-4.61 (*m*, 1H), 4.86 (*dt*, *J* 12.4, 6.2 Hz, 1H), 5.02 (*dt*, *J* 12.5, 6.2 Hz, 1H), 5.43 (*s*, 1H), 7.34 (*d*, *J* 8.0 Hz, 2H), 7.69-7.76 (*m*, 4H), 7.81 (*br. dd*, *J* 5.50, 3.06 Hz, 2H).

Diisopropyl 4-((((3S,4S)-4-amino-1-tosylpyrrolidin-3-yl)oxy)methyl)-2,6-dimethyl-1,4-dihydropyridine-3,5-dicarboxylate (1i)

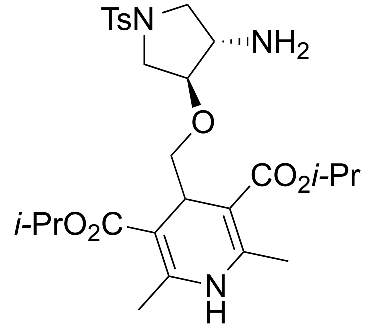

Synthesized according to General Procedure O. Purified by reverse-phase flash chromatography (C18, eluent: water/MeCN 1:1 *v/v* with 0.1% NH_4OH as modifier), then lyophilized. White fluffy solid. 62% yield. **^{1}H-NMR** (400 MHz, $CDCl_3$), *δ*, ppm: 1.10-1.14 (*m*, 12H), 2.11 (*s*, 6H), 2.30 (*s*, 3H), 2.87 (*dd*, *J* 10.0, 4.0 Hz, 1H), 2.97 (*dd*, *J* 10.5, 3.4 Hz, 1H), 3.03-3.14 (*m*, 3H), 3.23-3.32 (*m*, 1H), 3.36 (*dd*, *J* 10.5, 5.5 Hz, 1H), 3.39-3.51 (*m*, 1H), 3.95 (*t*, *J* 5.4 Hz, 1H), 4.90 (*dtt*, *J* 9.4, 6.2, 3.1 Hz, 2H), 5.41 (*s*, 1H), 7.19 (*d*, *J* 8.0 Hz, 2H), 7.57 (*d*, *J* 8.2 Hz, 2H). **^{13}C-NMR** (101 MHz, $CDCl_3$), *δ*, ppm: 18.8, 19.0, 21.3, 21.9, 33.8, 51.0, 53.2, 55.4, 66.5, 72.3, 83.9, 99.4, 99.5, 127.3, 129.4, 133.5, 143.2, 145.8, 145.9, 167.2, 167.3. **MS**: *m/z* calcd. for $C_{27}H_{40}N_3O_7S$ $[M+H]^+$ 550.3, found 550.5.

N,N-Dibenzyl-3-(2,2-dimethoxyethoxy)propan-1-amine

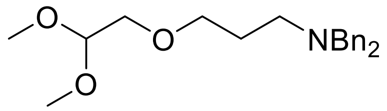

Synthesized according to General Procedure B. Purified by silica gel flash chromatography (eluent: *n*-heptane/EtOAc 4:1 *v/v*). Colorless oil. 70% yield. **^{1}H-NMR** (400 MHz, $CDCl_3$), *δ*, ppm: 1.81 (*quint.*, *J* 6.8 Hz, 2H), 2.50 (*t*, *J* 7.0 Hz, 2H), 3.36 (*s*, 6H), 3.39 (*d*, *J* 5.3 Hz, 2H), 3.49 (*t*, *J* 6.7 Hz, 2H), 3.55 (*s*, 4H), 4.40 (*t*, *J* 5.2 Hz, 1H), 7.21-7.26 (*m*, 2H), 7.28-7.37 (*m*, 8H).

2-(3-(Dibenzylamino)propoxy)acetaldehyde

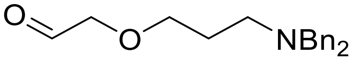

Synthesized according to General Procedure C. Used in the next step as such, without further purification. Colorless oil. 99% yield. **^{1}H-NMR** (400 MHz, $CDCl_3$), *δ*, ppm: 1.79-1.86 (*m*, 2H), 2.54 (*t*, *J* 6.7 Hz, 2H), 3.51-3.56 (*m*, 6H), 3.89 (*s*, 2H), 7.25 (*br. d*, *J* 7.1 Hz, 2H), 7.31-7.36 (*m*, 8H), 9.57 (*s*, 1H).

Diisopropyl 4-((3-(dibenzylamino)propoxy)methyl)-2,6-dimethyl-1,4-dihydropyridine-3,5-dicarboxylate

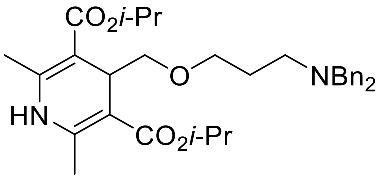

Synthesized according to General Procedure E. Purified by silica gel flash chromatography (eluent: *n*-heptane/EtOAc 4:1 *v/v*). Colorless oil. 61% yield. **^{1}H-NMR** (400 MHz, $CDCl_3$), *δ*, ppm: 1.25 (*d*, *J* 6.2 Hz, 12H), 1.67-1.77 (*m*, 2H), 2.17 (*s*, 6H), 2.42-2.49 (*m*, 2H), 3.21 (*d*, *J* 4.9 Hz, 2H), 3.38 (*t*, *J* 6.4 Hz, 2H), 3.55 (*s*, 4H), 4.05 (*t*, *J* 4.8 Hz, 1H), 5.04 (*quint.*, *J* 6.3 Hz, 2H), 5.14 (*s*, 1H), 7.20-7.25 (*m*, 2H), 7.31 (*t*, *J* 7.5 Hz, 4H), 7.35-7.39 (*m*, 4H).

Diisopropyl 4-((3-aminopropoxy)methyl)-2,6-dimethyl-1,4-dihydropyridine-3,5-dicarboxylate (1q)

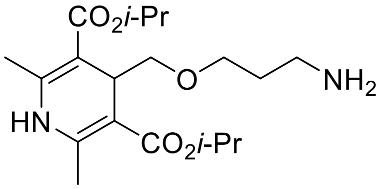

Synthesized according to General Procedure F. Purified by reverse-phase flash chromatography (C18, eluent: water/MeCN 1:1 *v/v* with 0.1% NH_4OH as modifier), then lyophilized. White fluffy solid. 92% yield. **^{1}H-NMR** (400 MHz, $CDCl_3$), *δ*, ppm: 1.29 (*d*, *J* 6.2 Hz, 12H), 1.67 (*quint.*, *J* 6.2 Hz, 2H), 2.30 (*s*, 6H), 2.78 (*t*, *J* 6.5 Hz, 2H), 3.30 (*d*, *J* 5.0 Hz, 2H), 3.50 (*t*, *J* 5.9 Hz, 2H), 4.15 (*t*, *J* 5.0 Hz, 1H), 5.09 (*quint.*, *J* 6.2 Hz, 2H), 5.77 (*s*, 1H). **^{13}C-NMR** (101 MHz, $CDCl_3$), *δ*, ppm: 19.5, 22.0, 22.1, 32.7, 34.5, 40.0, 66.9, 69.7, 74.4, 100.6, 145.4, 167.4. **MS**: *m/z* calcd. for $C_{19}H_{33}N_2O_5$ $[M+H]^+$ 369.2, found 369.6.

Thiomorpholine-Forming Amino-DHP Reagents

2-(2-Mercaptoethyl)isoindoline-1,3-dione

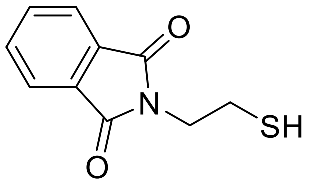

Synthesized according to General Procedure G. Purified by silica gel flash chromatography (eluent: *n*-heptane/EtOAc 3:2 *v/v*, followed by CH_2Cl_2/MeOH 95:5). White solid. 95% yield. **^{1}H-NMR** (400 MHz, $CDCl_3$), *δ*, ppm: 1.44 (*t*, *J* 8.6 Hz, 1H), 1.54 (*br. s*, 1H), 2.81-2.89 (*m*, 2H), 3.90 (*t*, *J* 7.2 Hz, 2H), 7.72-7.77 (*m*, 2H), 7.85-7.90 (*m*, 2H).

2-(2-((2,2-Dimethoxyethyl)thio)ethyl)isoindoline-1,3-dione

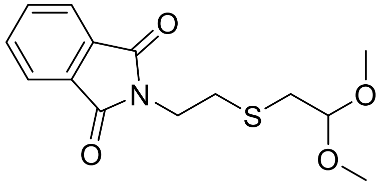

Synthesized according to General Procedure I. Purified by silica gel flash chromatography (eluent: *n*-heptane/EtOAc 3:2 *v/v*). White solid. 66% yield. **^{1}H-NMR** (400 MHz, $CDCl_3$), *δ*, ppm: 2.78 (*d*, *J* 5.4 Hz, 2H), 2.91 (*t*, *J* 7.0 Hz, 2H), 3.39 (*s*, 6H), 3.92 (*t*, *J* 6.9 Hz, 2H), 4.52 (*t*, *J* 5.4 Hz, 1H), 7.70-7.76

(*m*, 2H), 7.83-7.89 (*m*, 2H).

2-((2-(1,3-Dioxoisoindolin-2-yl)ethyl)thio)acetaldehyde

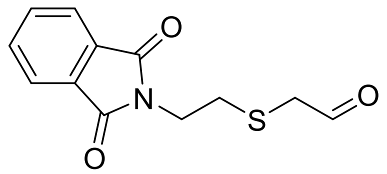

Synthesized according to General Procedure M. Used in the next step as such, without further purification. Colorless oil. 98% yield. **^{1}H-NMR** (400 MHz, $CDCl_3$), *δ*, ppm: 1.55 (*s*, 1H), 2.80 (*t*, *J* 6.5 Hz, 2H), 3.32 (*d*, *J* 3.4 Hz, 2H), 3.91 (*t*, *J* 6.5 Hz, 2H), 7.72-7.78 (*m*, 2H), 7.84-7.90 (*m*, 2H), 9.51 (*s*, 1H).

Diisopropyl 4-(((2-(1,3-dioxoisoindolin-2-yl)ethyl)thio)methyl)-2,6-dimethyl-1,4-dihydropyridine-3,5-dicarboxylate

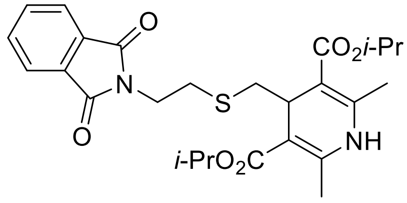

Synthesized according to General Procedure N. Purified by silica gel flash chromatography (eluent: *n*-heptane/EtOAc 3:2 *v/v*). Colorless oil. 52% yield. **^{1}H-NMR** (400 MHz, $CDCl_3$), *δ*, ppm: 1.25-1.33 (*m*, 12H), 2.30 (*s*, 6H), 2.58 (*d*, *J* 6.0 Hz, 2H), 2.83 (*t*, *J* 6.9 Hz, 2H), 3.89 (*t*, *J* 6.9 Hz, 2H), 4.19 (*t*, *J* 5.9 Hz, 1H), 5.08 (*sept.*, *J* 6.2 Hz, 2H), 5.62 (*s*, 1H), 7.68-7.75 (*m*, 2H), 7.81-7.86 (*m*, 2H).

Diisopropyl 4-(((2-aminoethyl)thio)methyl)-2,6-dimethyl-1,4-dihydropyridine-3,5-dicarboxylate (1j)

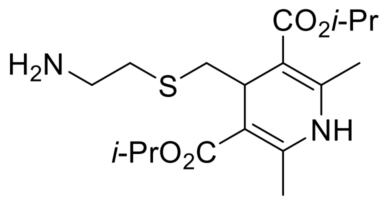

Synthesized according to General Procedure O. Purified by reverse-phase flash chromatography (C18, eluent: water/MeCN 1:1 *v/v* with 0.1% NH_4OH as modifier), then lyophilized. White fluffy solid. 45% yield. **^{1}H-NMR** (400 MHz, $CDCl_3$), *δ*, ppm: 1.29 (*dd*, *J* 6.1, 4.3 Hz, 12H), 2.30 (*s*, 6H), 2.48 (*d*, *J* 6.1 Hz, 2H), 2.61 (*t*, *J* 6.2 Hz, 2H), 2.81-2.89 (*m*, 2H), 4.16 (*t*, *J* 6.0 Hz, 1H), 5.08 (*sept.*, *J* 6.2 Hz, 2H), 5.75 (*s*, 1H). **^{13}C-NMR** (101 MHz, $CDCl_3$), *δ*, ppm: 19.5, 22.05, 22.06, 33.3, 36.5, 38.0, 40.7, 67.1, 102.6, 144.7, 167.3. **MS**: *m/z* calcd. for $C_{18}H_{31}N_2O_4S$ $[M+H]^+$ 371.2, found 371.2.

2-(2-((2,2-dimethoxyethyl)thio)propyl)isoindoline-1,3-dione

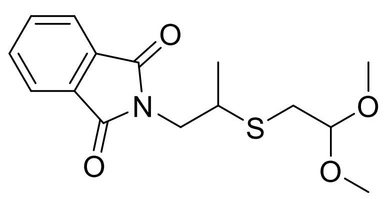

Synthesized according to General Procedures G and B. Purified by silica gel flash chromatography (eluent: *n*-heptane/EtOAc 3:2 *v/v*). Colorless oil. 52% yield (over two steps). **^{1}H-NMR** (400 MHz, $CDCl_3$), *δ*, ppm: 1.32 (*d*, *J* 7.0 Hz, 3H), 2.81 (*qd*, *J* 13.3, 5.6 Hz, 2H), 3.25-3.33 (*m*, 1H), 3.36 (*d*, *J* 5.3 Hz, 6H), 3.70-3.78 (*m*, 1H), 3.85 (*dd*, *J* 13.9, 7.0 Hz, 1H), 4.49 (*t*, *J* 5.6 Hz, 1H), 7.71-7.76 (*m*, 2H), 7.83-7.89 (*m*, 2H).

2-((1-(1,3-Dioxoisoindolin-2-yl)propan-2-yl)thio)acetaldehyde

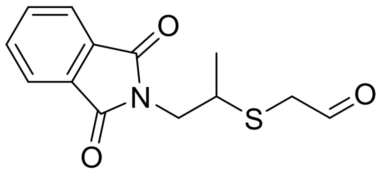

Synthesized according to General Procedure M. Used in the next step as such, without further purification. Colorless oil. 95% yield. **^{1}H-NMR** (400 MHz, $CDCl_3$), *δ*, ppm: 1.35 (*d*, *J* 6.9 Hz, 3H), 3.19 (*sext.*, *J* 7.0 Hz, 1H), 3.31-3.42 (*m*, 2H), 3.81 (*qd*, *J* 14.2, 7.1 Hz, 2H), 7.74-7.79 (*m*, 2H), 7.87-7.91 (*m*, 2H), 9.56 (*t*, *J* 3.2 Hz, 1H).

Diisopropyl 4-(((1-(1,3-dioxoisoindolin-2-yl)propan-2-yl)thio)methyl)-2,6-dimethyl-1,4-dihydropyridine-3,5-dicarboxylate

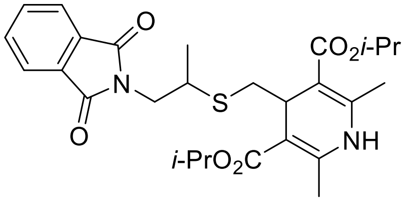

Synthesized according to General Procedure N. Purified by silica gel flash chromatography (eluent: *n*-heptane/EtOAc 3:2 *v/v*). Colorless oil. 61% yield. **^{1}H-NMR** (400 MHz, $CDCl_3$), *δ*, ppm: 1.23-1.31 (*m*, 15H), 2.27 (*d*, *J* 6.2 Hz, 6H), 2.66 (*d*, *J* 5.5 Hz, 2H), 3.12-3.20 (*m*, 1H), 3.64-3.71 (*m*, 1H), 3.75 (*d*, *J* 6.7 Hz, 1H), 4.19 (*t*, *J* 5.6 Hz, 1H), 4.99-5.11 (*m*, 2H), 5.55 (*s*, 1H), 7.72 (*dd*, *J* 5.4, 3.1 Hz, 2H), 7.83-7.87 (*m*, 2H).

Diisopropyl 4-(((1-aminopropan-2-yl)thio)methyl)-2,6-dimethyl-1,4-dihydropyridine-3,5-dicarboxylate (1l)

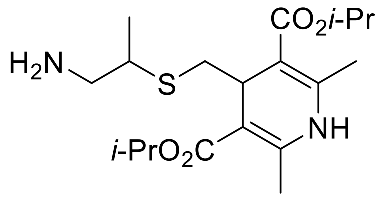

Synthesized according to General Procedure O. Purified by reverse-phase flash chromatography (C18, eluent: water/MeCN 1:1 *v/v* with 0.1% NH_4OH as modifier), then lyophilized. White fluffy solid. 66% yield. **^{1}H-NMR** (400 MHz, $CDCl_3$), *δ*, ppm: 1.19-1.24 (*m*, 3H), 1.25-1.31 (*m*, 12H), 2.29 (*d*, *J* 1.2 Hz, 6H), 2.49-2.55 (*m*, 2H), 2.62-2.69 (*m*, 1H), 2.73-2.84 (*m*, 2H), 4.16 (*t*, *J* 5.8 Hz, 1H), 5.02-5.13 (*m*, 2H), 5.79 (*s*, 1H). **^{13}C-NMR** (101 MHz, $CDCl_3$), *δ*, ppm: 18.9, 19.5, 19.6, 22.06, 22.07, 33.8, 36.3, 43.7, 47.0, 67.1, 102.5, 144.7, 167.3. **MS**: *m/z* calcd. for $C_{19}H_{33}N_2O_4S$ $[M+H]^+$ 385.2, found 385.2.

2-(1-Mercaptopropan-2-yl)isoindoline-1,3-dione

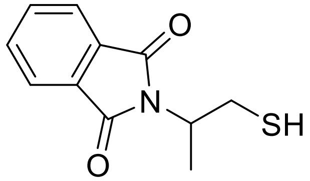

Synthesized according to General Procedure G. Purified by silica gel flash chromatography (eluent: *n*-heptane/EtOAc 3:2 *v/v*, followed by CH_2Cl_2/MeOH 95:5). White solid. 89% yield. **^{1}H-NMR** (400 MHz, $CDCl_3$), *δ*, ppm: 1.32-1.41 (*m*, 1H), 1.56 (*d*, *J* 7.0 Hz, 3H), 2.94 (*ddd*, *J* 13.7, 7.7, 5.9 Hz, 1H), 3.21 (*dt*, *J* 13.8, 9.7 Hz, 1H), 4.39 (*dquint.*, *J* 9.7, 6.7 Hz, 1H), 7.71-7.76 (*m*, 2H), 7.82-7.88 (*m*, 2H).

2-(1-((2,2-Dimethoxyethyl)thio)propan-2-yl)isoindoline-1,3-dione

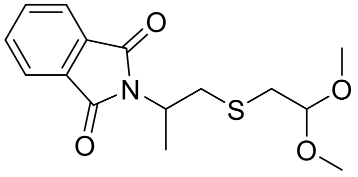

Synthesized according to General Procedure B. Purified by silica gel flash chromatography (eluent: *n*-heptane/EtOAc 1:1 *v/v*). Colorless oil. 69% yield.

^{1}**H-NMR** (400 MHz, $CDCl_3$), *δ*, ppm: 1.57 (*d*, *J* 7.0 Hz, 3H), 2.63-2.78 (*m*, 2H), 3.03 (*dd*, *J* 13.8, 5.4 Hz, 1H), 3.25 (*dd*, *J* 13.8, 10.2 Hz, 1H), 3.37 (*d*, *J* 3.2 Hz, 6H), 4.46-4.58 (*m*, 2H), 7.70-7.76 (*m*, 2H), 7.82-7.88 (*m*, 2H).

2-((2-(1,3-Dioxoisoindolin-2-yl)propyl)thio)acetaldehyde

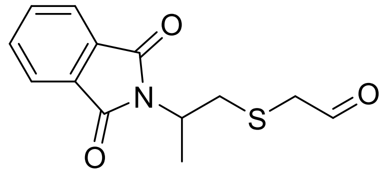

Synthesized according to General Procedure M. Used in the next step as such, without further purification. Colorless oil. 96% yield. ^{1}**H-NMR** (400 MHz, $CDCl_3$), *δ*, ppm: 1.55 (*d*, *J* 7.0 Hz, 3H), 2.76 (*dd*, *J* 14.1, 5.0 Hz, 1H), 3.12-3.24 (*m*, 2H), 3.26-3.37 (*m*, 1H), 4.43-4.54 (*m*, 1H), 7.70-7.77 (*m*, 2H), 7.81-7.88 (*m*, 2H), 9.46 (*dd*, *J* 4.3, 2.6 Hz, 1H).

Diisopropyl 4-(((2-(1,3-dioxoisoindolin-2-yl)propyl)thio)methyl)-2,6-dimethyl-1,4-dihydropyridine-3,5-dicarboxylate

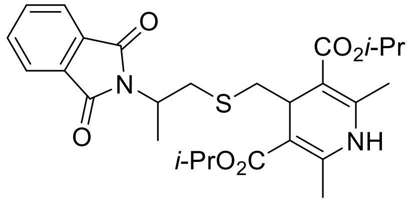

Synthesized according to General Procedure N. Purified by silica gel flash chromatography (eluent: *n*-heptane/EtOAc 3:2 *v/v*). Colorless oil. 73% yield. ^{1}**H-NMR** (400 MHz, $CDCl_3$), *δ*, ppm: 1.34 (*br. d*, *J* 6.2 Hz, 12H), 1.54-1.58 (*m*, 3H), 2.28 (*d*, *J* 3.3 Hz, 6H), 2.33-2.43 (*m*, 1H), 2.58-2.66 (*m*, 1H), 2.94-3.02 (*m*, 1H), 3.07-3.17 (*m*, 1H), 4.18 (*dd*, *J* 7.0, 5.0 Hz, 1H), 4.47-4.55 (*m*, 1H), 5.09 (sept., *J* 6.2 Hz, 2H), 5.60 (*s*, 1H), 7.69-7.73 (*m*, 2H), 7.82 (*dd*, *J* 5.4, 3.1 Hz, 2H).

Diisopropyl 4-(((2-aminopropyl)thio)methyl)-2,6-dimethyl-1,4-dihydropyridine-3,5-dicarboxylate (1k)

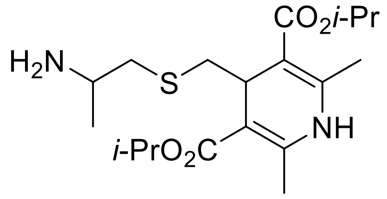

Synthesized according to General Procedure O. Purified by reverse-phase flash chromatography (C18, eluent: water/MeCN 1:2 *v/v* with 0.1% NH_4OH as modifier), then lyophilized. White fluffy solid. 42% yield. ^{1}**H-NMR** (400 MHz, $CDCl_3$), *δ*, ppm: 1.12 (*d*, *J* 6.4 Hz, 3H), 1.24-1.32 (*m*, 12H), 2.30 (*s*, 6H), 2.41-2.48 (*m*, 1H), 2.51-2.58 (*m*, 1H), 2.61-2.68 (*m*, 1H), 2.95-3.07 (*m*, 1H), 4.17 (*t*, *J* 6.0 Hz, 1H), 5.08 (*sept.*, *J* 6.2 Hz, 2H), 5.72 (*s*, 1H). ^{13}**C-NMR** (101 MHz, $CDCl_3$), *δ*, ppm: 19.5, 22.1, 23.0, 33.4, 38.7, 43.1, 45.9, 67.0, 67.0, 102.5, 102.6, 144.5, 144.8, 167.2, 167.3. **MS**: *m/z* calcd. for $C_{19}H_{33}N_2O_4S$ $[M+H]^+$ 385.2, found 385.3.

Piperazine-Forming Amino-DHP Reagents

2-(2-((2,2-Dimethoxyethyl)amino)ethyl)isoindoline-1,3-dione

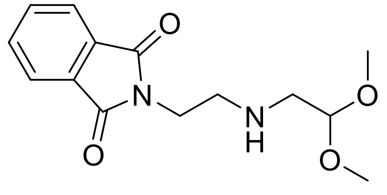

Synthesized according to General Procedure J. Purified by silica gel flash chromatography (eluent: *n*-heptane/EtOAc 3:2 *v/v*, followed by CH_2Cl_2/MeOH 95:5). White solid. 57% yield. ^{1}**H-NMR** (400 MHz, $CDCl_3$), *δ*, ppm: 2.80 (*d*, *J* 5.5 Hz, 2H), 2.96 (*t*, *J* 6.5 Hz, 2H), 3.39 (*s*, 6H), 3.83 (*t*, *J* 6.4 Hz, 2H), 4.39-4.48 (*m*, 1H), 7.71-7.77 (*m*, 2H), 7.83-7.91 (*m*, 2H).

N-(2,2-Dimethoxyethyl)-N-(2-(1,3-dioxoisoindolin-2-yl)ethyl)-4-methylbenzenesulfonamide

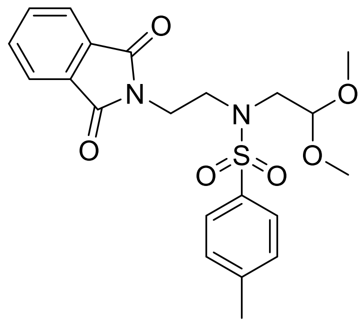

Synthesized according to General Procedure K. Purified by silica gel flash chromatography (eluent: *n*-heptane/EtOAc 3:2 *v/v*). Colorless oil. 92% yield. **^{1}H-NMR** (400 MHz, $CDCl_3$), *δ*, ppm: 2.33 (*s*, 3H), 3.41-3.46 (*m*, 8H), 3.54 (*t*, *J* 6.1 Hz, 2H), 3.91 (*t*, *J* 6.0 Hz, 2H), 4.57 (*t*, *J* 5.2 Hz, 1H), 7.17 (*d*, *J* 8.1 Hz, 2H), 7.64 (*d*, *J* 8.2 Hz, 2H), 7.70-7.75 (*m*, 2H), 7.80-7.85 (*m*, 2H).

N-(2-(1,3-Dioxoisoindolin-2-yl)ethyl)-4-methyl-N-(2-oxoethyl)benzenesulfonamide

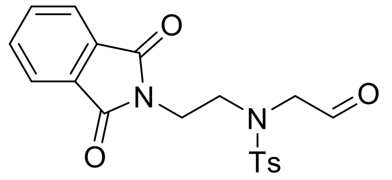

Synthesized according to General Procedure M. Used in the next step as such, without further purification. White solid. 99% yield. **^{1}H-NMR** (400 MHz, $CDCl_3$), *δ*, ppm: 2.31-2.35 (*m*, 3H), 3.47-3.51 (*m*, 2H), 3.78-3.85 (*m*, 2H), 4.04-4.10 (*m*, 2H), 7.16-7.21 (*m*, 2H), 7.59-7.63 (*m*, 2H), 7.71-7.77 (*m*, 2H), 7.82-7.87 (*m*, 2H), 9.65 (*s*, 1H).

Diisopropyl 4-(((N-(2-(1,3-dioxoisoindolin-2-yl)ethyl)-4-methylphenyl)sulfonamido)methyl)-2,6-dimethyl-1,4-dihydropyridine-3,5-dicarboxylate

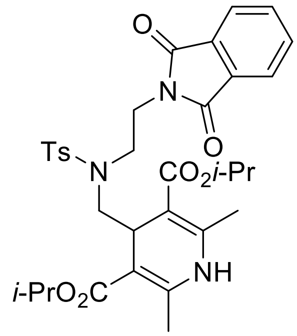

Synthesized according to General Procedure N. Purified by silica gel flash chromatography (eluent: *n*-heptane/EtOAc 3:2 *v/v*). White oil. 58% yield. **^{1}H-NMR** (400 MHz, $CDCl_3$), *δ*, ppm: 1.31 (*d*, *J* 6.2 Hz, 12H), 2.12-2.19 (*m*, 3H), 2.29 (*s*, 6H), 3.34 (*d*, *J* 6.9 Hz, 2H), 3.86 (*br. dd*, *J* 13.7, 4.9 Hz, 4H), 4.26 (*t*, *J* 6.9 Hz, 1H), 5.07 (*dt*, *J* 12.5, 6.2 Hz, 2H), 5.82 (*s*, 1H), 6.94 (*d*, *J* 8.1 Hz, 2H), 7.48 (*d*, *J* 8.2 Hz, 2H), 7.64-7.69 (*m*, 2H), 7.72-7.76 (*m*, 2H).

Diisopropyl 4-(((N-(2-aminoethyl)-4-methylphenyl)sulfonamido)methyl)-2,6-dimethyl-1,4-dihydropyridine-3,5-dicarboxylate (1n)

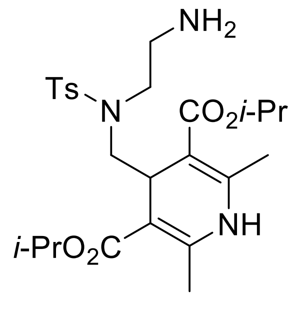

Synthesized according to General Procedure O. Purified by reverse-phase flash chromatography (C18, eluent: water/MeCN 1:2 *v/v* with 0.1% NH_4OH as modifier), then lyophilized. White fluffy solid. 30% yield. **^{1}H-NMR** (400 MHz, $CDCl_3$), *δ*, ppm: 1.29 (*t*, *J* 5.6 Hz, 12H), 2.28 (*s*, 6H), 2.40 (*s*, 3H), 2.86 (*t*, *J* 6.5 Hz, 2H), 2.97 (*d*, *J* 6.7 Hz, 2H), 3.30 (*t*, *J* 6.5 Hz, 2H), 4.22 (*t*, *J* 6.7 Hz, 1H), 5.00-5.12 (*m*, 2H), 5.90 (*s*, 1H), 7.25 (*d*, *J* 8.1 Hz, 2H), 7.61 (*d*, *J* 8.1 Hz, 2H). **^{13}C-NMR** (101 MHz, $CDCl_3$), *δ*, ppm: 19.7, 21.5, 22.10, 22.14, 32.4, 39.3, 50.4, 51.4, 67.3, 100.9, 127.2, 129.5, 137.4, 142.8, 145.6, 166.9. **MS**: *m/z* calcd. for $C_{25}H_{38}N_3O_6S$ $[M+H]^+$ 508.2, found 508.5.

Benzyl (2,2-dimethoxyethyl)(2-(1,3-dioxoisoindolin-2-yl)ethyl)carbamate

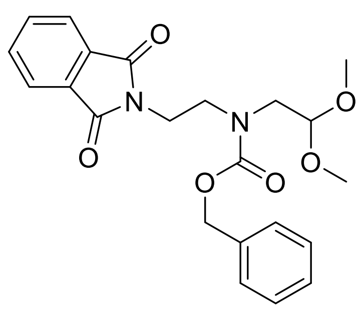

Synthesized according to General Procedure L. Purified by silica gel flash chromatography (eluent: *n*-heptane/EtOAc 1:1 *v/v*), followed by reverse-phase flash chromatography (C18, eluent: water/MeCN 1:1 *v/v* with 0.1% TFA as modifier), then lyophilized. White fluffy oil. 61% yield. Mixture of hydrolyzed product and the desired acetal (*approx.* 1:4). The product is rotameric. **^{1}H-NMR** (400 MHz, $CDCl_3$), *δ*, ppm: 3.32-3.44 (*m*, 8H), 3.64-3.73 (*m*, 2H), 3.84-3.95 (*m*, 2H), 4.36-4.58 (*m*, 1H), 4.94-5.05 (*m*, 2H), 7.24-7.37 (*m*, 5H), 7.70-7.84 (*m*, 4H).

Diisopropyl 4-((((benzyloxy)carbonyl)(2-(1,3-dioxoisoindolin-2-yl)ethyl)amino)methyl)-2,6-dimethyl-1,4-dihydropyridine-3,5-dicarboxylate

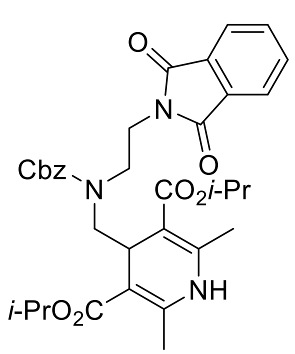

Synthesized according to General Procedures M and N. Purified by silica gel flash chromatography (eluent: *n*-heptane/EtOAc 3:2 *v/v*). White solid. 58% yield (over two steps). The product is rotameric. **^{1}H-NMR** (400 MHz, $CDCl_3$), *δ*, ppm: 1.19-1.27 (*m*, 12H), 2.10-2.35 (*m*, 6H), 3.20 (*br. d*, *J* 6.5 Hz, 2H), 3.60-3.69 (*m*, 2H), 3.79-3.94 (*m*, 2H), 4.10-4.19 (*m*, 1H), 4.84 (*d*, *J* 18.5 Hz, 2H), 4.93-5.05 (*m*, 2H), 5.46-5.79 (*m*, 1H), 7.17-7.26 (*m*, 3H), 7.27-7.31 (*m*, 2H), 7.64-7.80 (*m*, 4H).

Diisopropyl 4-(((2-aminoethyl)((benzyloxy)carbonyl)amino)methyl)-2,6-dimethyl-1,4-dihydropyridine-3,5-dicarboxylate (1m)

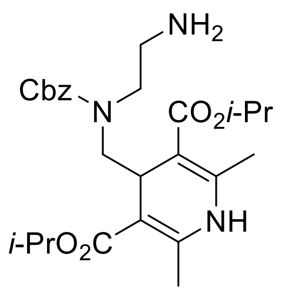

Synthesized according to General Procedure O. Purified by reverse-phase flash chromatography (C18, eluent: water/MeCN 1:2 *v/v* with 0.1% NH_4OH as modifier), then lyophilized. White fluffy solid. 40% yield. **^{1}H-NMR** (400 MHz, $CDCl_3$), *δ*, ppm: 1.25 (*br. d*, *J* 5.9 Hz, 12H), 2.10-2.32 (*m*, 6H), 2.83 (*dt*, *J* 19.5, 6.1 Hz, 2H), 3.12-3.27 (*m*, 2H), 3.34-3.44 (*m*, 2H), 4.12-4.21 (*m*, 1H), 4.97-5.10 (*m*, 4H), 5.64-6.22 (*m*, 1H), 7.28-7.37 (*m*, 5H). **MS**: *m/z* calcd. for $C_{26}H_{38}N_3O_6$ $[M+H]^+$ 488.3, found 488.7.

tert-Butyl (S)-(2-(1,3-dioxoisoindolin-2-yl)propyl)carbamate

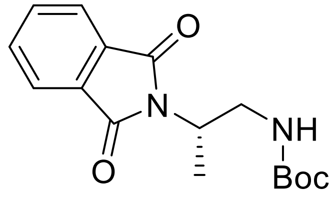

Synthesized according to General Procedure G. Purified by silica gel flash chromatography (eluent: *n*-heptane/EtOAc 3:2 *v/v*, followed by CH_2Cl_2/MeOH 95:5). White solid. 92% yield. **^{1}H-NMR** (400 MHz, $CDCl_3$), *δ*, ppm: 1.26-1.44 (*m*, 9H), 1.47 (*br. d*, *J* 7.0 Hz, 3H), 3.37 (*dt*, *J* 14.2, 4.9 Hz, 1H), 3.65-3.85 (*m*, 1H), 4.47-4.58 (*m*, 1H), 4.84 (*br. s*, 1H), 7.69-7.75 (*m*, 2H), 7.81-7.86 (*m*, 2H).

(S)-2-(1-Aminopropan-2-yl)isoindoline-1,3-dione, TFA Salt

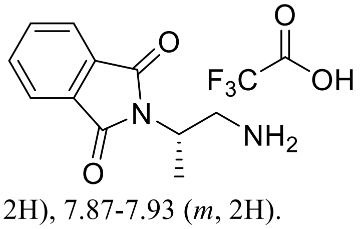

Synthesized according to General Procedure H. Used in the next step as such, without further purification. Colorless oil. 96% yield. **^{1}H-NMR** (400 MHz, methanol-*d_4*), *δ*, ppm: 1.53 (*d*, *J* 7.0 Hz, 3H), 3.26 (*dd*, *J* 13.3, 4.0 Hz, 1H), 3.69 (*dd*, *J* 13.2, 10.4 Hz, 1H), 4.63 (*ddd*, *J* 10.6, 6.9, 3.9 Hz, 1H), 7.81-7.86 (*m*, 2H), 7.87-7.93 (*m*, 2H).

(S)-2-(1-((2,2-Dimethoxyethyl)amino)propan-2-yl)isoindoline-1,3-dione

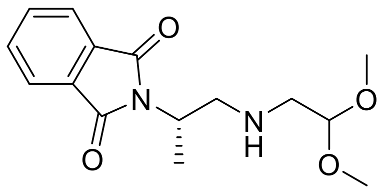

Synthesized according to General Procedure J. Purified by silica gel flash chromatography (eluent: *n*-heptane/EtOAc 3:2 *v/v*, followed by CH_2Cl_2/MeOH 95:5). White solid. 56% yield. **^{1}H-NMR** (400 MHz, $CDCl_3$), *δ*, ppm: 1.47 (*d*, *J* 7.0 Hz, 3H), 2.66-2.72 (*m*, 1H), 2.75-2.82 (*m*, 1H), 2.91 (*dd*, *J* 12.5, 5.5 Hz, 1H), 3.25-3.30 (*m*, 1H), 3.32 (*d*, *J* 1.0 Hz, 6H), 4.36 (*t*, *J* 5.4 Hz, 1H), 4.41-4.51 (*m*, 1H), 7.68-7.74 (*m*, 2H), 7.79-7.86 (*m*, 2H).

(S)-N-(2,2-Dimethoxyethyl)-N-(2-(1,3-dioxoisoindolin-2-yl)propyl)-4-methylbenzenesulfonamide

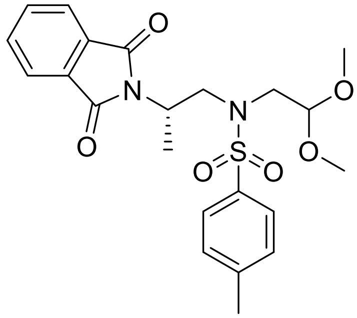

Synthesized according to General Procedure K. Purified by silica gel flash chromatography (eluent: *n*-heptane/EtOAc 3:2 *v/v*). Colorless oil. 94% yield. **^{1}H-NMR** (400 MHz, $CDCl_3$), *δ*, ppm: 1.46 (*d*, *J* 7.2 Hz, 3H), 2.33 (*s*, 3H), 3.23-3.31 (*m*, 2H), 3.35 (*s*, 3H), 3.39-3.45 (*m*, 4H), 4.00 (*dd*, *J* 14.7, 9.7 Hz, 1H), 4.49 (*t*, *J* 5.1 Hz, 1H), 4.66-4.76 (*m*, 1H), 7.17 (*d*, *J* 8.2 Hz, 2H), 7.61 (*d*, *J* 8.2 Hz, 2H), 7.67-7.72 (*m*, 2H), 7.76-7.83 (*m*, 2H).

(S)-N-(2-(1,3-Dioxoisoindolin-2-yl)propyl)-4-methyl-N-(2-oxoethyl)benzenesulfonamide

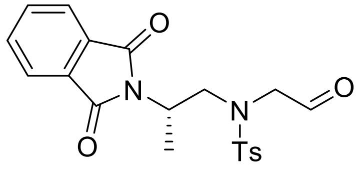

Synthesized according to General Procedure M. Used in the next step as such, without further purification. White solid. 73% yield. **^{1}H-NMR** (400 MHz, $CDCl_3$), *δ*, ppm: 1.50 (*d*, *J* 7.1 Hz, 3H), 2.38 (*s*, 3H), 3.06 (*dd*, *J* 14.6, 3.9 Hz, 1H), 3.94 (*s*, 2H), 4.03-4.11 (*m*, 1H), 4.52 (*ddt*, *J* 10.7, 7.1, 3.4 Hz, 1H), 7.23 (*d*, *J* 8.1 Hz, 2H), 7.62 (*d*, *J* 8.2 Hz, 2H), 7.71-7.77 (*m*, 2H), 7.83 (*dd*, *J* 5.4, 3.1 Hz, 2H), 9.54 (*s*, 1H).

Diisopropyl (S)-4-(((N-(2-(1,3-dioxoisoindolin-2-yl)propyl)-4-methylphenyl)sulfonamido)methyl)-2,6-dimethyl-1,4-dihydropyridine-3,5-dicarboxylate

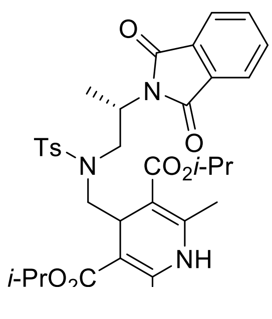

Synthesized according to General Procedures M and N. Purified by silica gel flash chromatography (eluent: *n*-heptane/EtOAc 3:2 *v/v*). White solid. 54% yield. **^{1}H-NMR** (400 MHz, $CDCl_3$), *δ*, ppm: 1.28-1.40 (*m*, 12H), 1.52 (*d*, *J* 7.1 Hz, 3H), 2.12-2.19 (*m*,

6H), 2.34 (*s*, 3H), 3.20 (*dd*, *J* 14.2, 3.7 Hz, 1H), 3.39 (*dd*, *J* 13.8, 9.8 Hz, 1H), 3.77 (*dd*, *J* 15.3, 2.7 Hz, 1H), 4.20-4.30 (*m*, 2H), 4.74-4.85 (*m*, 1H), 4.98-5.08 (*m*, 1H), 5.09-5.20 (*m*, 1H), 5.79 (*s*, 1H), 6.89 (*d*, *J* 8.1 Hz, 2H), 7.42 (*d*, *J* 8.2 Hz, 2H), 7.63-7.67 (*m*, 2H), 7.67-7.71 (*m*, 2H).

Diisopropyl (S)-4-(((N-(2-aminopropyl)-4-methylphenyl)sulfonamido)methyl)-2,6-dimethyl-1,4-dihydropyridine-3,5-dicarboxylate (1o)

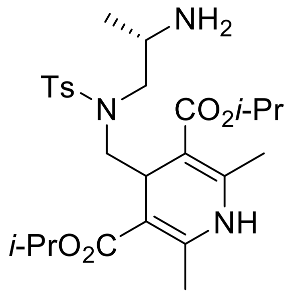

Synthesized according to General Procedure O. Purified by reverse-phase flash chromatography (C18, eluent: water/MeCN 1:2 *v/v* with 0.1% NH_4OH as modifier), then lyophilized. White fluffy solid. 77% yield. **^{1}H-NMR** (400 MHz, $CDCl_3$), *δ*, ppm: 1.01 (*d*, *J* 6.4 Hz, 3H), 1.29 (*td*, *J* 6.4, 4.7 Hz, 12H), 2.24 (*s*, 3H), 2.31 (*s*, 3H), 2.39 (*s*, 3H), 2.91-2.99 (*m*, 1H), 3.00-3.08 (*m*, 1H), 3.09-3.20 (*m*, 2H), 3.22-3.32 (*m*, 1H), 4.24 (*t*, *J* 6.8 Hz, 1H), 4.97-5.15 (*m*, 2H), 5.93 (*s*, 1H), 7.25 (*d*, *J* 8.1 Hz, 2H), 7.62 (*d*, *J* 8.2 Hz, 2H). **^{13}C-NMR** (101 MHz, $CDCl_3$), *δ*, ppm: 19.7, 19.8, 21.3, 21.4, 22.09, 22.14, 32.2, 44.2, 50.5, 57.1, 67.28, 67.34, 100.8, 101.0, 127.2, 129.5, 137.6, 142.7, 145.7, 145.8, 166.8, 166.9. **MS**: *m/z* calcd. for $C_{26}H_{40}N_3O_6S$ $[M+H]^+$ 522.3, found 522.4.

tert-Butyl (1-(1,3-dioxoisoindolin-2-yl)propan-2-yl)carbamate

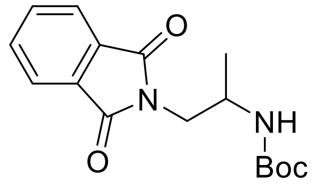

Synthesized according to General Procedure G. Purified by silica gel flash chromatography (eluent: *n*-heptane/EtOAc 3:2 *v/v*, followed by CH_2Cl_2/MeOH 95:5). White solid. 78% yield. **^{1}H-NMR** (400 MHz, $CDCl_3$), *δ*, ppm: 1.21 (*d*, *J* 6.72 Hz, 3H), 1.26 (*s*, 9H), 3.63-3.75 (*m*, 2H), 3.96-4.19 (*m*, 1H), 4.66 (*br. d*, *J* 7.5 Hz, 1H), 7.68-7.76 (*m*, 2H), 7.86 (*dd*, *J* 5.3, 3.1 Hz, 2H).

2-(2-Aminopropyl)isoindoline-1,3-dione, TFA Salt

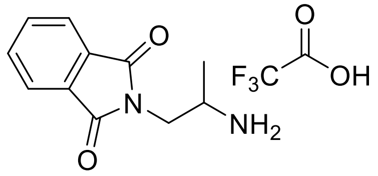

Synthesized according to General Procedure H. Used in the next step as such, without further purification. Colorless oil. 99% yield. **^{1}H-NMR** (400 MHz, methanol-*d_4*), *δ*, ppm: 1.35 (*d*, *J* 6.72 Hz, 3H), 3.59-3.71 (*m*, 1H), 3.85-3.93 (*m*, 2H), 7.83-7.88 (*m*, 2H), 7.89-7.95 (*m*, 2H).

2-(2-((2,2-Dimethoxyethyl)amino)propyl)isoindoline-1,3-dione

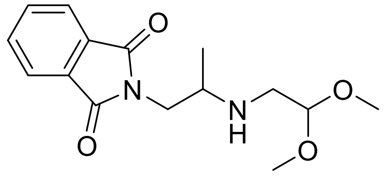

Synthesized according to General Procedure J. Purified by silica gel flash chromatography (eluent: *n*-heptane/EtOAc 3:2 *v/v*, followed by CH_2Cl_2/MeOH 95:5). White solid. 63% yield. **^{1}H-NMR** (400 MHz, methanol-*d_4*), *δ*, ppm: 1.10 (*d*, *J* 6.5 Hz, 3H), 2.75 (*qd*, *J* 12.5, 5.4 Hz, 2H), 3.04-3.12 (*m*, 1H), 3.33 (*d*, *J* 4.8 Hz, 6H), 3.58-3.65 (*m*, 1H), 3.66-3.74 (*m*, 1H), 4.40 (*t*, *J* 5.4 Hz, 1H), 7.79-7.84 (*m*, 2H), 7.84-7.90 (*m*, 2H).

***N*-(2,2-Dimethoxyethyl)-*N*-(1-(1,3-dioxoisoindolin-2-yl)propan-2-yl)-4-methylbenzenesulfonamide**

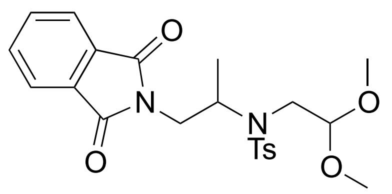

Synthesized according to General Procedure K. Purified by silica gel flash chromatography (eluent: *n*-heptane/EtOAc 3:2 *v/v*). Colorless oil. 92% yield. **^{1}H-NMR** (400 MHz, $CDCl_3$), *δ*, ppm: 1.11 (*d*, *J* 7.0 Hz, 3H), 2.30 (*s*, 3H), 3.26-3.33 (*m*, 1H), 3.39-3.46 (*m*, 4H), 3.49-3.57 (*m*, 4H), 3.93 (*dd*, *J* 14.1, 8.9 Hz, 1H), 4.30 (*ddd*, *J* 8.8, 7.0, 5.1 Hz, 1H), 4.66 (*t*, *J* 4.9 Hz, 1H), 7.14 (*d*, *J* 8.1 Hz, 2H), 7.63 (*d*, *J* 8.3 Hz, 2H), 7.68-7.73 (*m*, 2H), 7.79-7.85 (*m*, 2H).

***N*-(1-(1,3-dioxoisoindolin-2-yl)propan-2-yl)-4-methyl-*N*-(2-oxoethyl)benzenesulfonamide**

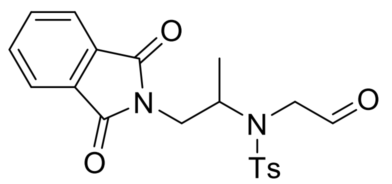

Synthesized according to General Procedure M. Used in the next step as such, without further purification. White solid. 87% yield. **^{1}H-NMR** (400 MHz, $CDCl_3$), *δ*, ppm: 1.04 (*d*, *J* 6.9 Hz, 3H), 2.25 (*s*, 3H), 3.47 (*dd*, *J* 14.5, 4.0 Hz, 1H), 3.67 (*dd*, *J* 14.5, 10.9 Hz, 1H), 3.77-3.84 (*m*, 1H), 3.95 (*d*, *J* 18.6 Hz, 1H), 4.27-4.38 (*m*, 1H), 7.06 (*d*, *J* 8.0 Hz, 2H), 7.58 (*d*, *J* 8.3 Hz, 2H), 7.70-7.76 (*m*, 2H), 7.76-7.83 (*m*, 2H), 9.65 (*d*, *J* 1.7 Hz, 1H).

Diisopropyl 4-(((N-(1-(1,3-dioxoisoindolin-2-yl)propan-2-yl)-4-methylphenyl)sulfonamido)methyl)-2,6-dimethyl-1,4-dihydropyridine-3,5-dicarboxylate

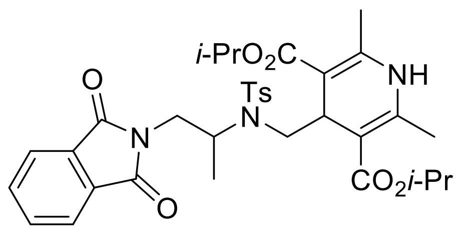

Synthesized according to General Procedure N. Purified by silica gel flash chromatography (eluent: *n*-heptane/EtOAc 3:2 *v/v*). White solid. 45% yield. The product is rotameric. **^{1}H-NMR** (400 MHz, $CDCl_3$), *δ*, ppm: 1.10 (*br. d*, *J* 6.7 Hz, 3H), 1.35 (*ddd*, *J* 12.0, 6.3, 3.1 Hz, 12H), 2.26 (*s*, 3H), 2.37 (*s*, 6H), 3.11 (*br. d*, *J* 6.6 Hz, 2H), 3.63-3.70 (*m*, 1H), 3.76 (*t*, *J* 2.9 Hz, 1H), 4.20-4.27 (*m*, 1H), 4.44 (*t*, *J* 6.9 Hz, 1H), 5.12 (*dq*, *J* 13.1, 6.4 Hz, 2H), 5.85 (*s*, 1H), 7.11 (*d*, *J* 8.1 Hz, 2H), 7.65-7.71 (*m*, 4H), 7.76-7.81 (*m*, 2H).

Diisopropyl 4-(((N-(1-aminopropan-2-yl)-4-methylphenyl)sulfonamido)methyl)-2,6-dimethyl-1,4-dihydropyridine-3,5-dicarboxylate (1p)

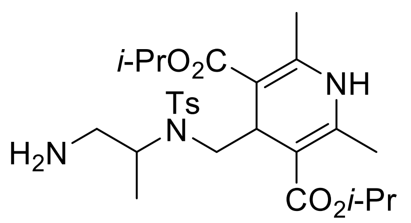

Synthesized according to General Procedure O. Purified by reverse-phase flash chromatography (C18, eluent: water/MeCN 1:2 *v/v* with 0.1% NH_4OH as modifier), then lyophilized. White fluffy solid. 70% yield. **^{1}H-NMR** (400 MHz, $CDCl_3$), *δ*, ppm: 0.71 (*d*, *J* 6.7 Hz, 3H), 1.28-1.37 (*m*, 12H), 2.34 (*d*, *J* 8.0 Hz, 6H), 2.39 (*s*, 3H), 2.60 (*dd*, *J* 13.5, 5.0 Hz, 1H), 2.68 (*dd*, *J* 14.6, 5.8 Hz, 1H), 2.88 (*dd*, *J* 13.5, 8.8 Hz, 1H), 3.05 (*dd*, *J* 14.6, 7.6 Hz, 1H), 3.57-3.68 (*m*, 1H), 4.28 (*dd*, *J* 7.3, 5.9 Hz, 1H), 5.04-5.19 (*m*, 2H), 6.03 (*s*, 1H), 7.24 (*d*, *J* 8.1 Hz, 2H), 7.64 (*d*, *J* 8.2 Hz, 2H). **^{13}C-NMR** (101 MHz, $CDCl_3$), *δ*, ppm: 14.7, 19.5, 20.1, 21.5, 22.13, 22.15, 22.20, 22.24, 34.8, 46.4, 46.6, 58.1, 67.4,

67.5, 101.1, 101.9, 127.3, 129.5, 137.9, 142.8, 145.2, 145.5, 167.1, 167.5. **MS**: *m/z* calcd. for $C_{26}H_{40}N_3O_6S$ $[M+H]^+$ 522.3, found 522.4.

Complete List of Commercially-Available Aldehydes

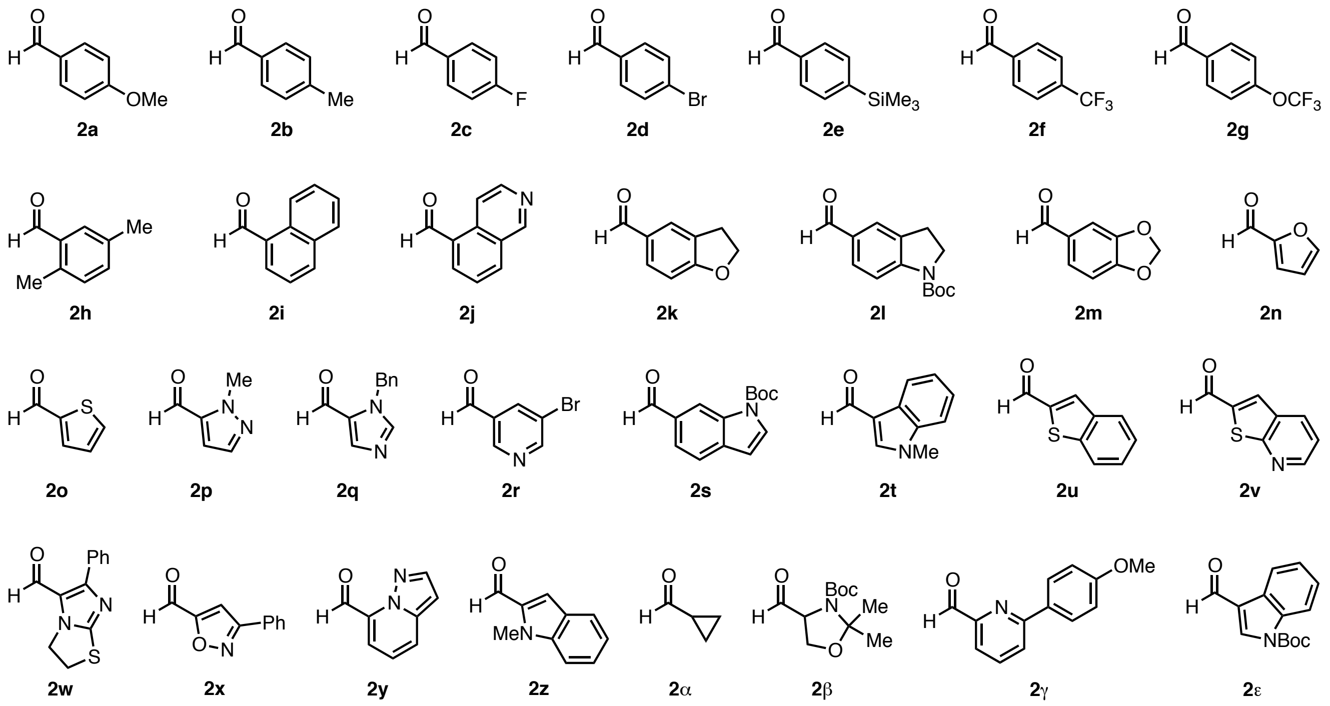

Scheme S3. List of commercially-available aldehydes.

Product Characterization

3-(4-Methoxyphenyl)morpholine, TFA salt (4aa)

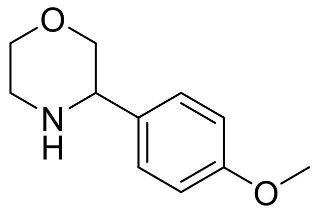

Synthesized according to General Procedure A, using 100 mg (0.23 mmol) of amino-DHP reagent **1a**. White fluffy solid. 80% yield. ^{1}**H-NMR** (400 MHz, methanol-d_4), δ, ppm: 3.35-3.44 (*m*, 2H), 3.81 (*s*, 3H), 3.82-3.98 (*m*, 2H), 4.00-4.15 (*m*, 2H), 4.41 (*dd*, *J* 10.8, 3.61 Hz, 1H), 7.02 (*d*, *J* 8.8 Hz, 2H), 7.43 (*d*, *J* 8.9 Hz, 2H). ^{13}**C-NMR** (101 MHz, methanol-d_4), δ, ppm: 45.3, 56.0, 59.8, 64.5, 70.0, 115.9, 125.9, 130.4, 162.6. ^{19}**F-NMR** (376 MHz, methanol-d_4), δ, ppm: −77.02 (*s*, 3F). **MS**: *m/z* calcd. for $C_{11}H_{16}NO_2$ $[M+H]^+$ 194.1, found 194.3.

3-(4-Bromophenyl)morpholine, TFA salt (4ad)

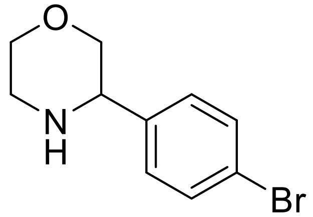

Synthesized according to General Procedure A, using 112 mg (0.23 mmol) of amino-DHP reagent **1a**. White fluffy solid. 72% yield. ^{1}**H-NMR** (400 MHz, methanol-d_4), δ, ppm: 3.38-3.47 (*m*, 2H), 3.81-3.97 (*m*, 2H), 4.07-4.16 (*m*, 2H), 4.50 (*dd*, *J* 10.8, 3.6 Hz, 1H), 7.43 (*d*, *J* 8.4 Hz, 2H), 7.62-7.71 (*m*, 2H). ^{13}**C-NMR**

(101 MHz, methanol-d_4), δ, ppm: 45.3, 59.5, 64.5, 69.7, 125.4, 130.9, 133.3, 133.9. **^{19}F-NMR** (376 MHz, methanol-d_4), δ, ppm: −77.04 (*s*, 3F). **MS**: *m/z* calcd. for $C_{10}H_{13}BrNO$ $[M+H]^+$ 242.0, found 242.1.

3-(4-Fluorophenyl)morpholine, TFA salt (4ac)

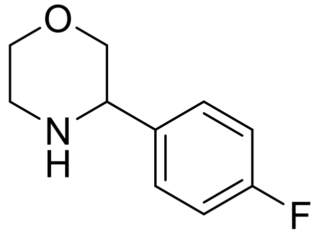

Synthesized according to General Procedure A, using 100 mg (0.23 mmol) of amino-DHP reagent **1a**. White fluffy solid. 60% yield. **^{1}H-NMR** (400 MHz, methanol-d_4), δ, ppm: 3.37-3.46 (*m*, 2H), 3.83-4.01 (*m*, 2H), 4.04-4.15 (*m*, 2H), 4.50 (*dd*, *J* 10.8, 3.6 Hz, 1H), 7.18-7.27 (*m*, 2H), 7.56 (*m*, 2H). **^{13}C-NMR** (101 MHz, methanol-d_4), δ, ppm: 45.3, 59.4, 64.5, 69.8, 117.4, 117.6, 130.3, 131.35, 131.44, 163.9, 166.3. **^{19}F-NMR** (376 MHz, methanol-d_4), δ, ppm: −112.96 (*s*, 3F), −77.02 (*s*, 1F). **MS**: *m/z* calcd. for $C_{10}H_{13}FNO$ $[M+H]^+$ 182.1, found 182.3.

3-(p-Tolyl)morpholine, TFA salt (4ab)

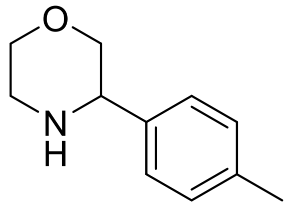

Synthesized according to General Procedure A, using 100 mg (0.23 mmol) of amino-DHP reagent **1a**. White fluffy solid. 58% yield. **^{1}H-NMR** (400 MHz, methanol-d_4), δ, ppm: 2.37 (*s*, 3H), 3.35-3.49 (*m*, 2H), 3.82-3.96 (*m*, 2H), 4.02-4.16 (*m*, 2H), 4.43 (*dd*, *J* 10.8, 3.6 Hz, 1H), 7.30 (*d*, *J* 8.1 Hz, 2H), 7.37 (*d*, *J* 8.2 Hz, 2H). **^{13}C-NMR** (101 MHz, methanol-d_4), δ, ppm: 21.3, 45.4, 60.0, 64.6, 70.1, 128.8, 131.1, 131.2, 141.7. **^{19}F-NMR** (376 MHz, methanol-d_4), δ, ppm: −76.93 (*s*, 3F). **MS**: *m/z* calcd. for $C_{11}H_{16}NO$ $[M+H]^+$ 178.1, found 178.3.

3-(Thieno[2,3-b]pyridin-2-yl)morpholine, TFA salt (4av)

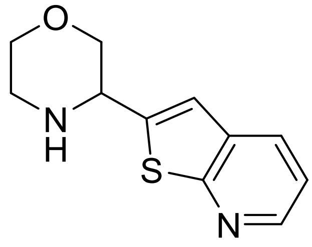

Synthesized according to General Procedure A, using 117 mg (0.25 mmol) of amino-DHP reagent **1a**. White fluffy solid. 68% yield. **^{1}H-NMR** (400 MHz, methanol-d_4), δ, ppm: 3.41-3.52 (*m*, 2H), 3.94 (*ddd*, *J* 13.1, 9.6, 3.7 Hz, 1H), 4.03-4.17 (*m*, 2H), 4.31 (*dd*, *J* 12.7, 3.6 Hz, 1H), 4.95-5.02 (*m*, 1H), 7.51 (*dd*, *J* 8.1, 4.7 Hz, 1H), 7.63 (*s*, 1H), 8.31 (*dd*, *J* 8.1, 1.4 Hz, 1H), 8.61 (*br. d*, *J* 4.0 Hz, 1H). **^{13}C-NMR** (101 MHz, methanol-d_4), δ, ppm: 44.8, 55.3, 64.8, 69.7, 122.1, 125.1, 134.1, 134.3, 136.3, 149.1, 162.4. **^{19}F-NMR** (376 MHz, methanol-d_4), δ, ppm: −77.25 (*s*, 3F). **MS**: *m/z* calcd. for $C_{11}H_{13}N_2OS$ $[M+H]^+$ 221.1, found 221.3.

3-(4-(Trifluoromethoxy)phenyl)morpholine, TFA salt (4ag)

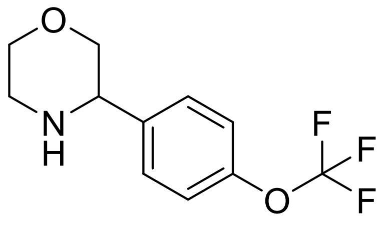

Synthesized according to General Procedure A, using 106 mg (0.20 mmol) of amino-DHP reagent **1a**. White fluffy solid. 69% yield. **^{1}H-NMR** (400 MHz, methanol-d_4), δ, ppm: 3.39-3.46 (*m*, 2H), 3.84-4.00 (*m*, 2H), 4.08-4.16 (*m*, 2H), 4.56 (*dd*, *J* 10.7, 3.6 Hz, 1H), 7.41 (*d*, *J* 8.3 Hz, 2H), 7.63 (*d*, *J* 8.8 Hz,

2H). **^{13}C-NMR** (101 MHz, methanol-*d_4*), *δ*, ppm: 45.3, 59.3, 64.6, 69.8, 118.1, 125.8 (*q*, J^{CF} 256 Hz), 123.1, 131.1, 133.2, 151.7. **^{19}F-NMR** (376 MHz, methanol-*d_4*), *δ*, ppm: −77.13 (*s*, 3F), −59.54 (*s*, 3F). **MS**: *m/z* calcd. for $C_{11}H_{13}F_3NO_2$ $[M+H]^+$ 248.1, found 248.41.

3-(4-(Trimethylsilyl)phenyl)morpholine, TFA salt (4ae)

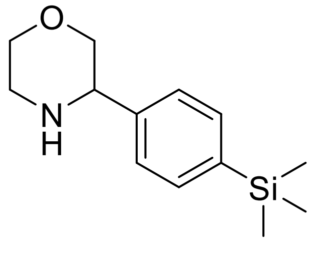

Synthesized according to General Procedure C, using 108 mg (0.21 mmol) of amino-DHP reagent **1a**. White fluffy solid. 82% yield. **^{1}H-NMR** (400 MHz, methanol-*d_4*), *δ*, ppm: 0.27 (*s*, 9H), 3.37-3.48 (*m*, 2H), 3.83-3.97 (*m*, 2H), 4.05-4.16 (*m*, 2H), 4.47 (*dd*, *J* 10.9, 3.6 Hz, 1H), 7.47 (*d*, *J* 8.0 Hz, 2H), 7.64 (*d*, *J* 8.0 Hz, 2H). **^{13}C-NMR** (101 MHz, methanol-*d_4*), *δ*, ppm: −1.2, 45.4, 60.2, 64.6, 70.0, 128.1, 134.5, 135.5, 144.3. **^{19}F-NMR** (376 MHz, methanol-*d_4*), *δ*, ppm: −76.90 (*s*, 3F). **MS**: *m/z* calcd. for $C_{13}H_{22}NOSi$ $[M+H]^+$ 236.1, found 236.2.

3-(4-(Trifluoromethyl)phenyl)morpholine, TFA salt (4af)

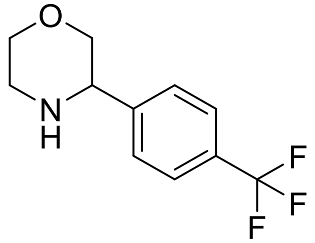

Synthesized according to General Procedure A, using 106 mg (0.21 mmol) of amino-DHP reagent **1a**. White fluffy solid. 67% yield. **^{1}H-NMR** (400 MHz, methanol-*d_4*), *δ*, ppm: 3.45 (*dd*, *J* 6.9, 2.3 Hz, 2H), 3.87-3.99 (*m*, 2H), 4.09-4.20 (*m*, 2H), 4.63 (*dd*, *J* 10.6, 3.6 Hz, 1H), 7.72 (*m*, *J* 8.3 Hz, 2H), 7.78-7.84 (*m*, 2H). **^{13}C-NMR** (101 MHz, methanol-*d_4*), *δ*, ppm: 45.4, 59.6, 64.6, 69.7, 125.4 (*q*, J^{CF} 271 Hz), 127.5 (*q*, J^{CF} 3.7 Hz), 129.8, 133.2 (*q*, J^{CF} 32.3 Hz), 138.3. **^{19}F-NMR** (376 MHz, methanol-*d_4*), *δ*, ppm: −77.10 (*s*, 3F), −64.49 (*s*, 3F). **MS**: *m/z* calcd. for $C_{11}H_{13}F_3NO$ $[M+H]^+$ 232.1, found 232.3.

3-(Benzo[b]thiophen-2-yl)morpholine, TFA salt (4au)

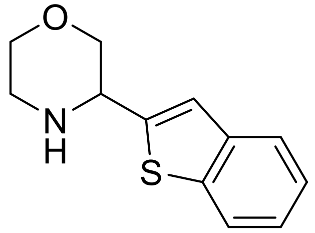

Synthesized according to General Procedure A, using 84 mg (0.17 mmol) of amino-DHP reagent **1a**. White fluffy solid. 69% yield. **^{1}H-NMR** (400 MHz, methanol-*d_4*), *δ*, ppm: 3.38-3.52 (*m*, 2H), 3.85-4.00 (*m*, 1H), 4.00-4.16 (*m*, 2H), 4.29 (*dd*, *J* 12.6, 3.6 Hz, 1H), 4.90-4.95 (*m*, 1H), 7.39-7.47 (*m*, 2H), 7.60 (*s*, 1H), 7.85-7.96 (*m*, 2H). **^{13}C-NMR** (101 MHz, methanol-*d_4*), *δ*, ppm: 44.9, 55.3, 64.7, 69.9, 123.6, 125.5, 126.4, 126.98, 127.04, 135.6, 140.5, 141.3. **^{19}F-NMR** (376 MHz, methanol-*d_4*), *δ*, ppm: −77.06 (*s*, 3F). **MS**: *m/z* calcd. for $C_{12}H_{14}NOS$ $[M+H]^+$ 220.1, found 220.2.

3-(2,3-Dihydrobenzofuran-5-yl)morpholine, TFA salt (4ak)

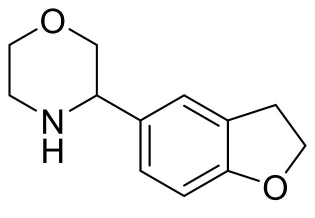

Synthesized according to General Procedure A, using 116 mg (0.24 mmol) of amino-DHP reagent **1a**. White fluffy solid. 69% yield. **^{1}H-NMR** (400 MHz, methanol-*d_4*), *δ*, ppm: 3.23 (*t*, *J* 8.7 Hz, 2H), 3.34-3.43 (*m*, 2H), 3.81-3.96 (*m*, 2H),

4.01-4.13 (*m*, 2H), 4.38 (*dd*, *J* 10.9, 3.7 Hz, 1H), 4.58 (*t*, *J* 8.7 Hz, 2H), 6.80 (*d*, *J* 8.3 Hz, 1H), 7.18-7.24 (*m*, 1H), 7.34 (*s*, 1H). **^{13}C-NMR** (101 MHz, methanol-*d_4*), *δ*, ppm: 30.4, 45.3, 60.1, 64.5, 70.1, 72.9, 110.9, 125.8, 129.3, 130.3, 163.1. **^{19}F-NMR** (376 MHz, methanol-*d_4*), *δ*, ppm: −76.97 (*s*, 3F). **MS**: *m/z* calcd. for $C_{12}H_{16}NO_2$ $[M+H]^+$ 206.1, found 206.2.

3-(2,5-Dimethylphenyl)morpholine, TFA salt (4ah)

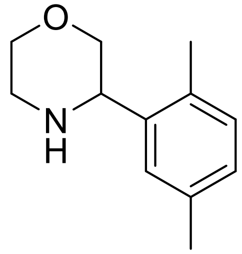

Synthesized according to General Procedure A, using 96 mg (0.20 mmol) of amino-DHP reagent **1a**. White fluffy solid. 82% yield. **^{1}H-NMR** (400 MHz, methanol-*d_4*), *δ*, ppm: 2.33-2.36 (*m*, 3H), 2.40 (*s*, 3H), 3.38-3.45 (*m*, 1H), 3.45-3.56 (*m*, 1H), 3.78-3.95 (*m*, 2H), 4.02 (*dd*, *J* 13.0, 3.6 Hz, 1H), 4.13 (*dd*, *J* 12.9, 3.6 Hz, 1H), 4.66 (*dd*, *J* 10.9, 3.6 Hz, 1H), 7.18 (*q*, *J* 7.8 Hz, 2H), 7.29 (*s*, 1H). **^{13}C-NMR** (101 MHz, methanol-*d_4*), *δ*, ppm: 18.9, 21.2, 45.7, 57.0, 64.6, 70.0, 127.5, 131.6, 132.1, 132.6, 135.0, 138.1. **^{19}F-NMR** (376 MHz, methanol-*d_4*), *δ*, ppm: −76.93 (*s*, 3F). **MS**: *m/z* calcd. for $C_{12}H_{18}NO$ $[M+H]^+$ 192.1, found 192.2.

3-(5-Bromopyridin-3-yl)morpholine, TFA salt (4ar)

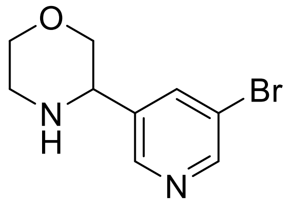

Synthesized according to General Procedure A, using 106 mg (0.20 mmol) of amino-DHP reagent **1a**. White fluffy solid. 61% yield. **^{1}H-NMR** (400 MHz, methanol-*d_4*), *δ*, ppm: 3.39-3.50 (*m*, 2H), 3.86-3.95 (*m*, 1H), 4.00 (*dd*, *J* 12.8, 10.5 Hz, 1H), 4.09-4.19 (*m*, 2H), 4.63 (*dd*, *J* 10.3, 3.6 Hz, 1H), 8.24 (*t*, *J* 1.8 Hz, 1H), 8.64-8.73 (*m*, 1H), 8.73-8.83 (*m*, 1H). **^{13}C-NMR** (101 MHz, methanol-*d_4*), *δ*, ppm: 45.2, 57.1, 64.6, 69.1, 122.5, 132.3, 140.2, 148.7, 153.2. **^{19}F-NMR** (376 MHz, methanol-*d_4*), *δ*, ppm: −77.45 (*s*, 3F). **MS**: *m/z* calcd. for $C_9H_{12}BrN_2O$ $[M+H]^+$ 243.0, found 243.1.

3-(1-Methyl-1H-indol-3-yl)morpholine, TFA salt (4at)

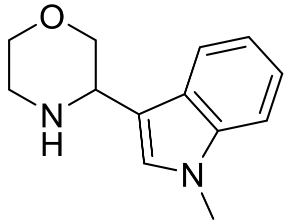

Synthesized according to General Procedure A, using 108 mg (0.22 mmol) of amino-DHP reagent **1a**. White fluffy solid. 79% yield. **^{1}H-NMR** (400 MHz, methanol-*d_4*), *δ*, ppm: 3.33-3.40 (*m*, 1H), 3.40-3.51 (*m*, 1H), 3.83 (*s*, 3H), 3.87-3.96 (*m*, 1H), 4.03-4.20 (*m*, 3H), 4.82 (*dd*, *J* 10.2, 3.8 Hz, 1H), 7.12-7.23 (*m*, 1H), 7.23-7.31 (*m*, 1H), 7.40-7.45 (*m*, 1H), 7.46 (*s*, 1H), 7.74 (*d*, *J* 8.0 Hz, 1H). **^{13}C-NMR** (101 MHz, methanol-*d_4*), *δ*, ppm: 33.3, 44.8, 52.6, 64.7, 69.9, 107.1, 111.1, 119.5, 121.4, 123.9, 127.7, 129.9, 138.5. **^{19}F-NMR** (376 MHz, methanol-*d_4*), *δ*, ppm: −77.03 (*s*, 3F). **MS**: *m/z* calcd. for $C_{13}H_{17}N_2O$ $[M+H]^+$ 217.1, found 217.2.

3-(Thiophen-2-yl)morpholine, TFA salt (4ao)

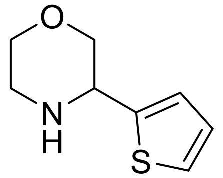

Synthesized according to General Procedure A, using 97 mg (0.22 mmol) of amino-DHP reagent **1a**. White fluffy solid. 54% yield. **^{1}H-NMR** (400 MHz, methanol-*d_4*), *δ*, ppm: 3.36-

3.42 (*m*, 2H), 3.83-3.92 (*m*, 1H), 3.93-4.01 (*m*, 1H), 4.05-4.12 (*m*, 1H), 4.15-4.22 (*m*, 1H), 4.83 (*dd*, *J* 10.0, 3.7 Hz, 1H), 7.14 (*dd*, *J* 5.0, 3.7 Hz, 1H), 7.34 (*d*, *J* 3.4 Hz, 1H), 7.56-7.63 (*m*, 1H). **^{13}C-NMR** (101 MHz, methanol-d_4), *δ*, ppm: 44.7, 54.7, 64.6, 70.1, 128.8, 129.2, 130.1, 135.0. **^{19}F-NMR** (376 MHz, methanol-d_4), *δ*, ppm: −76.99 (*s*, 3F). **MS**: *m/z* calcd. for $C_8H_{11}NOS$ $[M+H]^+$ 170.1, found 170.1.

3-(1-Methyl-1H-pyrazol-5-yl)morpholine, TFA salt (4ap)

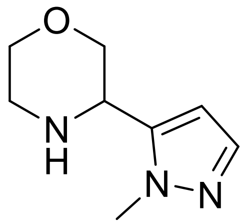

Synthesized according to General Procedure A, using 96 mg (0.22 mmol) of amino-DHP reagent **1a**. White fluffy solid. 58% yield. **^{1}H-NMR** (400 MHz, methanol-d_4), *δ*, ppm: 3.35-3.53 (*m*, 2H), 3.83-3.94 (*m*, 2H), 3.95-3.98 (*m*, 3H), 4.06-4.16 (*m*, 2H), 4.83 (*dd*, *J* 10.0, 3.7 Hz, 1H), 6.60 (*d*, *J* 2.0 Hz, 1H), 7.52 (*d*, *J* 1.7 Hz, 1H). **^{13}C-NMR** (101 MHz, methanol-d_4), *δ*, ppm: 37.2, 44.7, 50.8, 64.6, 68.9, 107.3, 136.1, 140.0. **^{19}F-NMR** (376 MHz, methanol-d_4), *δ*, ppm: −77.13 (*s*, 3F). **MS**: *m/z* calcd. for $C_8H_{14}N_3O$ $[M+H]^+$ 168.1, found 168.4.

3-(Furan-2-yl)morpholine, TFA salt (4an)

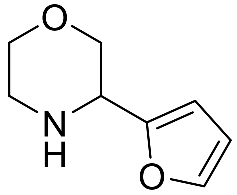

Synthesized according to General Procedure A, using 89 mg (0.21 mmol) of amino-DHP reagent **1a**. White fluffy solid. 67% yield. **^{1}H-NMR** (400 MHz, methanol-d_4), *δ*, ppm: 3.40 (*br. d*, *J* 3.4 Hz, 2H), 3.77-3.89 (*m*, 1H), 3.96-4.03 (*m*, 1H), 4.03-4.10 (*m*, 1H), 4.14-4.21 (*m*, 1H), 4.65 (*dd*, *J* 10.0, 3.7 Hz, 1H), 6.52 (*dd*, *J* 3.2, 1.8 Hz, 1H), 6.65 (*d*, *J* 3.3 Hz, 1H), 7.62-7.67 (*m*, 1H). **^{13}C-NMR** (101 MHz, methanol-d_4), *δ*, ppm: 44.4, 52.8, 64.7, 67.9, 112.1, 112.2, 145.8, 147.4. **^{19}F-NMR** (376 MHz, methanol-d_4), *δ*, ppm: −76.98 (*s*, 3F). **MS**: *m/z* calcd. for $C_8H_{12}NO_2$ $[M+H]^+$ 154.1, found 154.1.

tert-Butyl 6-(morpholin-3-yl)-indole-1-carboxylate, Free base (4as)

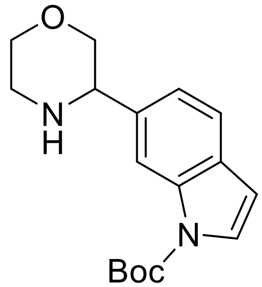

Synthesized according to General Procedure B, using 106 mg (0.18 mmol) of amino-DHP reagent **1a**. White fluffy solid. 73% yield. **^{1}H-NMR** (400 MHz, methanol-d_4), *δ*, ppm: 1.68 (*s*, 9H), 2.92-3.01 (*m*, 1H), 3.01-3.12 (*m*, 1H), 3.50 (*t*, *J* 10.8 Hz, 1H), 3.64 (*td*, *J* 11.4, 2.7 Hz, 1H), 3.86 (*dd*, *J* 11.4, 2.9 Hz, 2H), 3.97 (*dd*, *J* 10.3, 3.1 Hz, 1H), 6.58 (*d*, *J* 3.7 Hz, 1H), 7.23-7.28 (*m*, 1H), 7.51-7.55 (*m*, 1H), 7.57-7.61 (*m*, 1H), 8.22 (*s*, 1H). **^{13}C-NMR** (101 MHz, methanol-d_4), *δ*, ppm: 28.5, 47.4, 62.0, 68.0, 74.3, 85.1, 108.3, 115.0, 122.1, 123.3, 127.4, 131.8, 136.9, 137.5, 151.1. **MS**: *m/z* calcd. for $C_{17}H_{23}N_2O_3$ $[M+H]^+$ 303.2, found 303.58.

3-(Benzo[d][1,3]dioxol-5-yl)morpholine, TFA salt (4am)

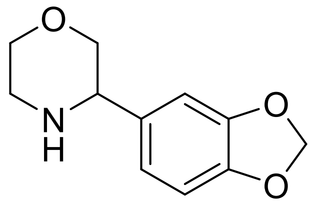

Synthesized according to General Procedure A, using 88 mg (0.18 mmol) of amino-DHP reagent **1a**. White fluffy solid. 75% yield. **^{1}H-NMR** (400 MHz, methanol-d_4), *δ*, ppm: 3.34-3.44 (*m*, 2H), 3.80-3.93 (*m*, 2H), 4.03-4.16 (*m*, 2H),

4.39 (*dd*, *J* 10.8, 3.6 Hz, 1H), 6.01 (*s*, 2H), 6.88-6.94 (*m*, 1H), 6.94-7.01 (*m*, 2H). **^{13}C-NMR** (101 MHz, methanol-*d$_4$*), *δ*, ppm: 45.4, 60.0, 64.5, 70.0, 103.3, 109.0, 110.1, 123.2, 127.5, 150.1, 150.7. **^{19}F-NMR** (376 MHz, methanol-*d$_4$*), *δ*, ppm: −76.96 (*s*, 3F). **MS**: *m/z* calcd. for $C_{11}H_{13}NO_3$ $[M+H]^+$ 208.1, found 208.3.

3-(Isoquinolin-5-yl)morpholine, TFA salt (4aj)

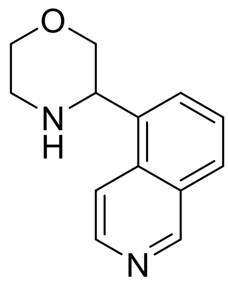

Synthesized according to General Procedure A, using 86 mg (0.17 mmol) of amino-DHP reagent **1a**. White fluffy solid. 77% yield. **^{1}H-NMR** (400 MHz, methanol-*d$_4$*), *δ*, ppm: 3.07 (*br. d*, *J* 12.4 Hz, 1H), 3.22 (*td*, *J* 12.0, 3.4 Hz, 1H), 3.43-3.51 (*m*, 1H), 3.70 (*br. d*, *J* 2.6 Hz, 1H), 3.98 (*br. d*, *J* 2.9 Hz, 2H), 4.67-4.74 (*m*, 1H), 7.72 (*t*, *J* 7.8 Hz, 1H), 8.01 (*d*, *J* 7.2 Hz, 1H), 8.06 (*d*, *J* 8.2 Hz, 1H), 8.16 (*d*, *J* 6.2 Hz, 1H), 8.50 (*d*, *J* 6.1 Hz, 1H), 9.25 (*s*, 1H). **^{19}F-NMR** (376 MHz, methanol-*d$_4$*), *δ*, ppm: −77.07 (*s*, 3F). **MS**: *m/z* calcd. for $C_{13}H_{15}N_2O$ $[M+H]^+$ 215.1, found 215.38.

3-(1-Benzyl-imidazol-5-yl)morpholine, TFA salt (4aq)

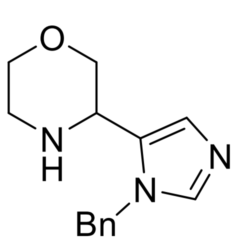

Synthesized according to General Procedure A, using 89 mg (0.17 mmol) of amino-DHP reagent **1a**. White fluffy solid. 85% yield. **^{1}H-NMR** (400 MHz, methanol-*d$_4$*), *δ*, ppm: 3.37-3.52 (*m*, 2H), 3.69 (*dd*, *J* 12.7, 3.6 Hz, 1H), 3.80-3.96 (*m*, 2H), 3.98-4.08 (*m*, 1H), 4.78 (*dd*, *J* 9.0, 3.6 Hz, 1H), 5.48-5.60 (*m*, 1H), 5.60-5.69 (*m*, 1H), 7.34-7.39 (*m*, 2H), 7.43-7.51 (*m*, 3H), 7.98 (*s*, 1H), 8.90 (*s*, 1H). **^{13}C-NMR** (101 MHz, methanol-*d$_4$*), *δ*, ppm: 44.2, 48.9, 52.0, 64.7, 68.6, 123.9, 128.5, 129.4, 130.7, 130.9, 134.6, 138.8. **^{19}F-NMR** (376 MHz, methanol-*d$_4$*), *δ*, ppm: −77.06 (*s*, 3F). **MS**: *m/z* calcd. for $C_{14}H_{18}N_3O$ $[M+H]^+$ 244.1, found 244.2.

2-(Naphthalen-2-yl)morpholine, TFA salt (4ai)

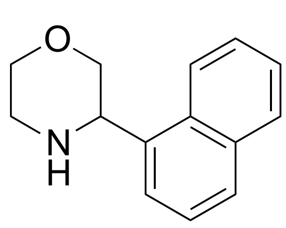

Synthesized according to General Procedure A, using 91 mg (0.18 mmol) of amino-DHP reagent **1a**. White fluffy solid. 81% yield. **^{1}H-NMR** (400 MHz, methanol-*d$_4$*), *δ*, ppm: 3.46-3.54 (*m*, 1H), 3.61-3.71 (*m*, 1H), 3.93-4.04 (*m*, 2H), 4.22 (*ddd*, *J* 12.7, 8.8, 3.5 Hz, 2H), 5.43 (*dd*, *J* 10.6, 3.4 Hz, 1H), 7.57-7.64 (*m*, 2H), 7.66-7.73 (*m*, 2H), 8.00 (*t*, *J* 8.6 Hz, 2H), 8.25 (*d*, *J* 8.6 Hz, 1H). **^{13}C-NMR** (101 MHz, methanol-*d$_4$*), *δ*, ppm: 45.8, 56.0, 64.8, 70.6, 123.3, 125.4, 126.5, 127.9, 128.8, 130.0, 130.5, 131.7, 132.0, 135.7. **^{19}F-NMR** (376 MHz, methanol-*d$_4$*), *δ*, ppm: −76.98 (*s*, 3F). **MS**: *m/z* calcd. for $C_{14}H_{16}NO$ $[M+H]^+$ 214.1, found 214.3.

tert-Butyl 4-(morpholin-3-yl)indoline-1-carboxylate, Free base (4al)

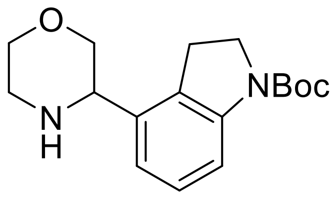

Synthesized according to General Procedure B, using 99 mg (0.17 mmol) of amino-DHP reagent **1a**. White fluffy solid. 70% yield. **^{1}H-NMR** (400 MHz, methanol-*d$_4$*), *δ*, ppm: 1.56 (*s*, 9H), 2.92-2.99 (*m*, 1H), 2.99-3.08 (*m*, 1H), 3.09-3.24 (*m*, 2H), 3.36-3.44 (*m*, 1H), 3.58-3.67 (*m*, 1H), 3.74-3.80 (*m*, 1H), 3.81-3.87

(*m*, 1H), 3.88-3.93 (*m*, 1H), 3.94-4.03 (*m*, 2H), 7.07 (*d*, *J* 7.7 Hz, 1H), 7.17 (*t*, *J* 8.0 Hz, 1H), 7.36-7.80 (*m*, 1H). **^{13}C-NMR** (101 MHz, methanol-d_4), δ, ppm: 26.7, 28.8, 47.4, 58.5, 68.1, 72.8, 82.0, 115.1, 121.2, 129.1, 131.2, 137.4, 144.1, 154.3. **MS**: *m/z* calcd. for $C_{17}H_{25}N_2O_3$ $[M+H]^+$ 305.2, found 305.3.

3-(6-Phenyl-2,3-dihydroimidazo[2,1-b]thiazol-5-yl)morpholine, Free base (4aw)

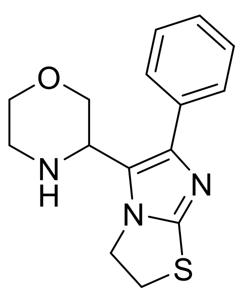

Synthesized according to General Procedure B, using 113 mg (0.20 mmol) of amino-DHP reagent **1a**. White fluffy solid. 79% yield. **^{1}H-NMR** (400 MHz, methanol-d_4), δ, ppm: 2.85-2.96 (*m*, 2H), 3.56-3.70 (*m*, 2H), 3.76-3.83 (*m*, 2H), 3.85-3.90 (*m*, 2H), 4.18 (*dd*, *J* 10.6, 3.3 Hz, 1H), 4.38 (*dt*, *J* 11.3, 7.3 Hz, 1H), 4.63 (*dt*, *J* 11.3, 7.2 Hz, 1H), 7.28-7.34 (*m*, 1H), 7.39 (*t*, *J* 7.5 Hz, 2H), 7.43-7.51 (*m*, 2H). **MS**: *m/z* calcd. for $C_{15}H_{18}N_3OS$ $[M+H]^+$ 288.1, found 288.1.

3-(3-Phenylisoxazol-5-yl)morpholine, Free base (4ax)

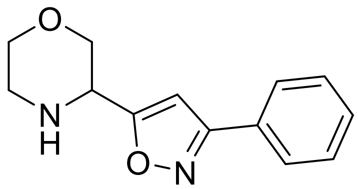

Synthesized according to General Procedure B, using 125 mg (0.25 mmol) of amino-DHP reagent **1a**. White fluffy solid. 70% yield. **^{1}H-NMR** (400 MHz, methanol-d_4), δ, ppm: 3.38-3.48 (*m*, 2H), 3.86-3.97 (*m*, 1H), 4.03-4.14 (*m*, 2H), 4.33 (*dd*, *J* 12.7, 3.7 Hz, 1H), 4.94 (*br. d*, *J* 3.7 Hz, 1H), 7.13 (*s*, 1H), 7.47-7.54 (*m*, 3H), 7.83-7.90 (*m*, 2H). **^{13}C-NMR** (101 MHz, methanol-d_4), δ, ppm: 44.3, 51.4, 64.9, 67.7, 104.1, 128.0, 129.5, 130.4, 131.9, 164.5, 165.8. **MS**: *m/z* calcd. for $C_{13}H_{15}N_2O_2$ $[M+H]^+$ 231.1, found 231.1.

3-(Pyrazolo[1,5-a]pyridin-7-yl)morpholine, Free base (4ay)

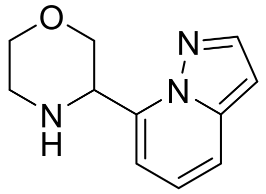

Synthesized according to General Procedure B, using 82 mg (0.17 mmol) of amino-DHP reagent **1a**. White fluffy solid. 55% yield. **^{1}H-NMR** (400 MHz, methanol-d_4), δ, ppm: 2.97-3.04 (*m*, 1H), 3.05-3.14 (*m*, 1H), 3.60-3.74 (*m*, 2H), 3.89 (*s*, 1H), 4.28 (*dd*, *J* 11.0, 3.1 Hz, 1H), 4.76 (*dd*, *J* 8.7, 3.0 Hz, 1H), 6.63 (*d*, *J* 2.2 Hz, 1H), 6.97 (*d*, *J* 7.0 Hz, 1H), 7.21 (*dd*, *J* 8.9, 7.0 Hz, 1H), 7.61 (*d*, *J* 8.8 Hz, 1H), 7.98 (*d*, *J* 2.2 Hz, 1H). **MS**: *m/z* calcd. for $C_{11}H_{14}N_3O$ $[M+H]^+$ 204.1, found 204.2.

7-(4-Methoxyphenyl)-5-oxa-8-azaspiro[3.5]nonane, TFA salt (4ba)

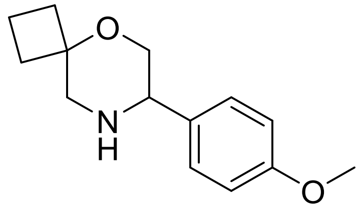

Synthesized according to General Procedure A, using 90 mg (0.18 mmol) of amino-DHP reagent **1b**. White fluffy solid. 61% yield. **^{1}H-NMR** (400 MHz, methanol-d_4), δ, ppm: 1.72-1.85 (*m*, 1H), 1.88-1.99 (*m*, 1H), 2.07-2.14 (*m*, 1H), 2.18 (*br. s*, 2H), 2.45 (*ddt*, *J* 12.1, 8.3, 4.0 Hz, 1H), 3.20 (*d*, *J* 12.1 Hz, 1H), 3.57 (*d*, *J* 12.8 Hz, 1H), 3.81 (*s*, 3H), 3.88-4.03 (*m*, 2H), 4.35 (*dd*, *J* 10.4, 3.9

Hz, 1H), 7.02 (*d*, *J* 8.8 Hz, 2H), 7.41 (*d*, *J* 8.8 Hz, 2H). **^{13}C-NMR** (101 MHz, methanol-d_4), *δ*, ppm: 13.1, 30.8, 32.6, 56.0, 58.7, 64.3, 75.2, 115.9, 125.4, 130.7, 162.6. **^{19}F-NMR** (376 MHz, methanol-d_4), *δ*, ppm: −76.93 (*s*, 3F). **MS**: *m/z* calcd. for $C_{14}H_{20}NO_2$ $[M+H]^+$ 234.1, found 234.2.

7-(6-Phenyl-2,3-dihydroimidazo[2,1-b]thiazol-5-yl)-5-oxa-8-azaspiro[3.5]nonane, Free base (4bw)

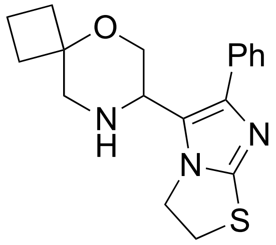

Synthesized according to General Procedure B, using 127 mg (0.21 mmol) of amino-DHP reagent **1b**. White fluffy solid. 68% yield. **^{1}H-NMR** (400 MHz, methanol-d_4), *δ*, ppm: 1.59-1.74 (*m*, 1H), 1.76-1.92 (*m*, 2H), 1.97-2.11 (*m*, 2H), 2.32-2.49 (*m*, 1 H), 2.70 (*dd*, *J* 11.6, 1.7 Hz, 1H), 3.07 (*br. d*, *J* 11.6 Hz, 1H), 3.60 (*br. dd*, *J* 11.3, 3.5 Hz, 1H), 3.66-3.74 (*m*, 1H), 3.90 (*br. t*, *J* 7.2 Hz, 2H), 4.14 (*dd*, *J* 10.8, 3.4 Hz, 1H), 4.34 (*dt*, *J* 11.3, 7.2 Hz, 1H), 4.64 (*dt*, *J* 11.3, 7.2 Hz, 1H), 7.28-7.35 (*m*, 1H), 7.41 (*br. t*, *J* 7.6 Hz, 2H), 7.45-7.55 (*m*, 2 H). **^{13}C-NMR** (101 MHz, methanol-d_4), *δ*, ppm: 13.8, 31.4, 32.9, 36.2, 39.7, 52.6, 53.9, 65.9, 76.8, 127.0, 128.6, 129.2, 129.6, 135.7, 145.2, 151.7. **MS**: *m/z* calcd. for $C_{18}H_{22}N_3OS$ $[M+H]^+$ 328.1, found 328.2.

(S)-3-(4-Methoxyphenyl)-5-methylmorpholine, TFA salt (4ca)

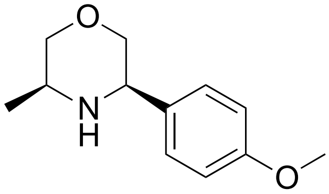

Synthesized according to General Procedure A, using 72 mg (0.15 mmol) of amino-DHP reagent **1c**. White fluffy solid. 68% yield, 8:1 dr. **^{1}H-NMR** (400 MHz, methanol-d_4), *δ*, ppm: 1.29 (*d*, *J* 6.5 Hz, 3H), 3.48-3.56 (*m*, 1H), 3.60 (*dt*, *J* 6.8, 3.5 Hz, 1H), 3.82 (*s*, 3H), 3.83-3.89 (*m*, 1H), 4.07 (*dd*, *J* 12.5, 3.2 Hz, 2H), 4.44 (*dd*, *J* 11.3, 3.4 Hz, 1H), 7.03 (*d*, *J* 8.7 Hz, 2H), 7.43 (*d*, *J* 8.80 Hz, 2H). **^{13}C-NMR** (101 MHz, methanol-d_4), *δ*, ppm: 14.2, 54.0, 56.0, 60.7, 69.5, 70.2, 115.9, 125.5, 130.5, 162.6. **^{19}F-NMR** (376 MHz, methanol-d_4), *δ*, ppm: −76.94 (*s*, 3F). **MS**: *m/z* calcd. for $C_{12}H_{18}NO_2$ $[M+H]^+$ 208.1, found 208.2.

(2S)-2-Methyl-5-(6-phenyl-2,3-dihydroimidazo[2,1-b]thiazol-5-yl)morpholine, Free base (4dw)

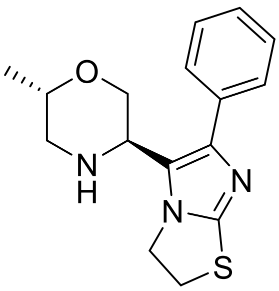

Synthesized according to General Procedure B, using 115 mg (0.20 mmol) of amino-DHP reagent **1d**. White fluffy solid. 69% yield, 14:1 dr. **^{1}H-NMR** (400 MHz, methanol-d_4), *δ*, ppm: 1.24 (*d*, *J* 6.2 Hz, 3H), 3.06 (*dd*, *J* 12.4, 11.2 Hz, 1H), 3.36 (*dd*, *J* 12.7, 1.9 Hz, 1H), 3.85-3.97 (*m*, 1H), 4.02-4.12 (*m*, 3H), 4.14-4.23 (*m*, 1H), 4.44-4.51 (*m*, 1H), 4.64 (*dd*, *J* 11.2, 3.7 Hz, 1H), 4.78 (*dt*, *J* 10.6, 6.7 Hz, 1H), 7.47-7.56 (*m*, 5H). **^{13}C-NMR** (101 MHz, methanol-d_4), *δ*, ppm: 18.5, 36.7, 50.2, 51.0, 51.7, 67.8, 71.2, 120.4, 129.2, 130.1, 130.3, 132.2, 146.7, 154.9. **MS**: *m/z* calcd. for $C_{16}H_{20}N_3OS$ $[M+H]^+$ 302.1, found 302.2.

(2S)-5-(4-Methoxyphenyl)-2-phenylmorpholine, Free base (4ea)

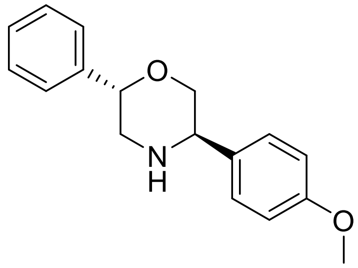

Synthesized according to General Procedure B, using 100 mg (0.18 mmol) of amino-DHP reagent **1e**. White fluffy solid. 77% yield, >14:1 dr. **^{1}H-NMR** (400 MHz, methanol-d_4), δ, ppm: 2.87 (*dd*, *J* 12.5, 10.6 Hz, 1H), 3.13 (*dd*, *J* 12.5, 2.5 Hz, 1H), 3.59-3.66 (*m*, 1H), 3.78 (*s*, 3H), 3.90 (*dd*, *J* 10.5, 3.2 Hz, 1H), 3.98 (*dd*, *J* 11.1, 3.3 Hz, 1H), 4.57 (*dd*, *J* 10.6, 2.4 Hz, 1H), 6.86-6.98 (*m*, 2H), 7.26-7.31 (*m*, 1H), 7.32-7.37 (*m*, 4H), 7.38-7.43 (*m*, 2H). **^{13}C-NMR** (101 MHz, methanol-d_4), δ, ppm: 54.4, 55.8, 60.3, 74.6, 79.6, 115.1, 127.4, 129.0, 129.45, 129.51, 133.0, 141.7, 161.0. **MS**: *m/z* calcd. for $C_{17}H_{20}NO_2$ $[M+H]^+$ 270.1, found 270.4.

tert-Butyl (S)-2,2-dimethyl-4-((3R,6S)-6-phenylmorpholin-3-yl)oxazolidine-3-carboxylate, Free base (4eβ)

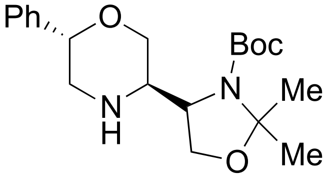

Synthesized according to General Procedure B, using 112 mg (0.17 mmol) of amino-DHP reagent **1e**. White fluffy solid. 58% yield, >14:1 dr. **^{1}H-NMR** (400 MHz, methanol-d_4), δ, ppm: 1.46-1.50 (*m*, 3H), 1.52 (*s*, 9H), 1.59 (*s*, 3H) 2.74 (*br. t*, *J* 11.7 Hz, 1H), 3.06 (*br. dd*, *J* 12.5, 2.1 Hz, 1H), 3.21-3.28 (*m*, 1H), 3.51-3.59 (*m*, 1H), 3.77-4.03 (*m*, 4 H), 4.15 (*br. s*, 1H), 4.42 (*dd*, *J* 10.6, 2.3 Hz, 1H), 7.30-7.37 (*m*, 5H). **^{13}C-NMR** (101 MHz, methanol-d_4), δ, ppm: 22.7, 24.1, 26.4, 27.4, 28.8, 54.1, 54.5, 56.7, 57.2, 58.3, 60.0, 60.2, 64.5, 64.7, 65.8, 70.2, 71.7, 72.0, 80.1, 81.9, 82.1, 95.2, 95.4, 127.3, 129.0, 129.5, 141.6, 154.0, 154.6. **MS**: *m/z* calcd. for $C_{20}H_{31}N_2O_4$ $[M+H]^+$ 363.2, found 363.4.

rac-(3R,4S,7S)-3-(4-Methoxyphenyl)hexahydro-2H-furo[3,4-b][1,4]oxazine, TFA salt (4fa)

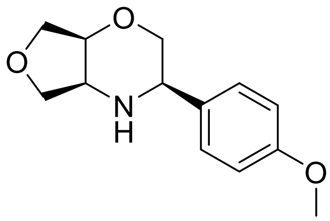

Synthesized according to General Procedure A, using 97 mg (0.19 mmol) of amino-DHP reagent **1f**. White fluffy solid. 55% yield, 9:1 dr. **^{1}H-NMR** (400 MHz, methanol-d_4), δ, ppm: 3.82 (*s*, 3H), 3.83-3.92 (*m*, 2H), 4.00-4.18 (*m*, 5H), 4.39-4.55 (*m*, 2H), 7.03 (*d*, *J* 8.7 Hz, 2H), 7.47 (*d*, *J* 8.8 Hz, 2H). **^{13}C-NMR** (101 MHz, methanol-d_4), δ, ppm: 55.9, 57.56, 57.59, 68.1, 69.7, 73.1, 75.5, 115.4, 129.9, 132.2, 161.4. **^{19}F-NMR** (376 MHz, methanol-d_4), δ, ppm: −76.93 (*s*, 3F). **MS**: *m/z* calcd. for $C_{13}H_{18}NO_3$ $[M+H]^+$ 236.1, found 236.3.

rac-tert-Butyl 3-((3R,4S,7S)-hexahydro-2H-furo[3,4-b][1,4]oxazin-3-yl)-1H-indole-1-carboxylate, Free base (4fε)

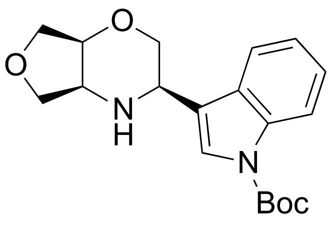

Synthesized according to General Procedure B, using 173 mg (0.28 mmol) of amino-DHP reagent **1f**. White fluffy solid. 50% yield, 8:1 dr. **^{1}H-NMR** (400 MHz, methanol-d_4), δ, ppm: 1.68 (*s*, 9H), 3.53 (*td*, *J* 5.0, 1.8 Hz, 1H), 3.71-3.86 (*m*, 4H) 3.91 (*dd*, *J* 9.4, 4.8 Hz, 1H), 4.10 (*dd*, *J* 8.9, 6.7 Hz, 1H), 4.21 (*dd*, *J* 8.6, 4.2 Hz, 1H), 4.36-4.42 (*m*, 1H), 4.57 (*s*, 2H), 7.23-7.28 (*m*, 1H), 7.30-7.35 (*m*,

1H), 7.67-7.72 (*m*, 2H), 8.13 (*d*, *J* 8.2 Hz, 1H). ^{13}**C-NMR** (101 MHz, methanol-*d₄*), *δ*, ppm: 28.6, 50.8, 56.6, 67.8, 68.2, 73.9, 75.6, 85.2, 116.4, 120.6, 122.0, 123.9, 124.2, 125.8, 130.4, 137.1, 151.1. **MS**: *m/z* calcd. for $C_{19}H_{25}N_2O_4$ $[M+H]^+$ 345.2, found 345.3.

rac-(3R,4S,7R)-3-(4-Methoxyphenyl)hexahydro-2H-furo[3,4-b][1,4]oxazine, Free base (4ga)

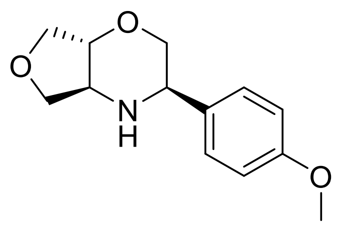

Synthesized according to General Procedure B, using 89 mg (0.17 mmol) of amino-DHP reagent **1g**. White fluffy solid. 66% yield, >14:1 dr. ^{1}**H-NMR** (400 MHz, methanol-d_4), *δ*, ppm: 3.16 (*ddd*, *J* 10.7, 9.2, 7.3 Hz, 1H), 3.50-3.65 (*m*, 3H), 3.68-3.75 (*m*, 1H), 3.78 (*s*, 3H), 3.90 (*dd*, *J* 11.5, 3.6 Hz, 1H), 3.93-4.01 (*m*, 3H), 6.90 (*d*, *J* 8.8 Hz, 2H), 7.35 (*d*, *J* 8.7 Hz, 2H). ^{13}**C-NMR** (101 MHz, methanol-d_4), *δ*, ppm: 55.8, 61.4, 62.4, 68.0, 69.3, 74.9, 81.4, 115.1, 129.8, 132.5, 161.1. **MS**: *m/z* calcd. for $C_{13}H_{18}NO_3$ $[M+H]^+$ 236.1, found 236.5.

rac-(3R,4S,7R)-3-(4-Methoxyphenyl)-6-tosyloctahydropyrrolo[3,4-b][1,4]oxazine, Free base (4ha)

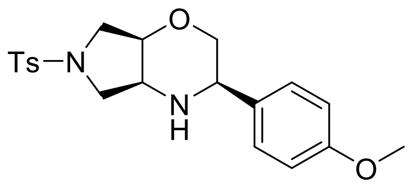

Synthesized according to General Procedure B, using 100 mg (0.15 mmol) of amino-DHP reagent **1h**. White fluffy solid. 69% yield, 10:1 dr. ^{1}**H-NMR** (400 MHz, methanol-d_4), *δ*, ppm: 2.47 (*s*, 3H), 3.17-3.29 (*m*, 2H), 3.42-3.52 (*m*, 1H), 3.73-3.88 (*m*, 6H), 3.96-4.05 (*m*, 1H), 4.15 (*dd*, *J* 13.0, 3.9 Hz, 1H), 4.49 (*dd*, *J* 11.4, 3.8 Hz, 1H), 7.00 (*d*, *J* 8.8 Hz, 2H), 7.38 (*d*, *J* 8.7 Hz, 2H), 7.48 (*d*, *J* 8.1 Hz, 2H), 7.78 (*d*, *J* 8.2 Hz, 2H). ^{13}**C-NMR** (101 MHz, methanol-d_4), *δ*, ppm: 21.7, 46.7, 49.4, 56.0, 58.3, 61.0, 70.5, 76.5, 116.0, 124.7, 128.8, 130.6, 131.4, 135.1, 146.1, 162.7. **MS**: *m/z* calcd. for $C_{20}H_{25}N_2O_4S$ $[M+H]^+$ 389.2, found 389.3.

rac-(3R,4S,7R)-3-(1-Methyl-1H-pyrazol-5-yl)-6-tosyloctahydropyrrolo[3,4-b][1,4]oxazine, Free base (4hp)

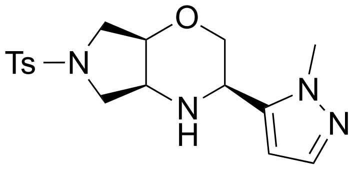

Synthesized according to General Procedure B, using 101 mg (0.16 mmol) of amino-DHP reagent **1h**. White fluffy solid. 57% yield, 7:1 dr. ^{1}**H-NMR** (400 MHz, methanol-d_4), *δ*, ppm: 2.48 (*s*, 3H), 2.80 (*ddd*, *J* 10.7, 9.5, 7.3 Hz, 1H), 2.96-3.02 (*m*, 1H), 3.04-3.11 (*m*, 1H), 3.32-3.39 (*m*, 1H), 3.45-3.54 (*m*, 1H), 3.59-3.71 (*m*, 2H), 3.87 (*s*, 3H), 3.93 (*dd*, *J* 11.5, 3.4 Hz, 1H), 4.14 (*dd*, *J* 10.7, 3.5 Hz, 1H), 6.28 (*d*, *J* 1.8 Hz, 1H), 7.39 (*d*, *J* 1.7 Hz, 1H), 7.48 (*d*, *J* 8.1 Hz, 2H), 7.78 (*d*, *J* 8.2 Hz, 2H). ^{13}**C-NMR** (101 MHz, methanol-d_4), *δ*, ppm: 21.8, 37.0, 39.8, 50.1, 52.6, 60.6, 72.0, 79.6, 105.7, 128.8, 131.3, 135.3, 139.3, 142.0, 145.6. **MS**: *m/z* calcd. for $C_{17}H_{23}N_4O_3S$ $[M+H]^+$ 363.1, found 363.2.

rac-(3R,4S,7S)-3-(1-Methyl-1H-indol-2-yl)-6-tosyloctahydropyrrolo[3,4-b][1,4]oxazine, Free base (4iz)

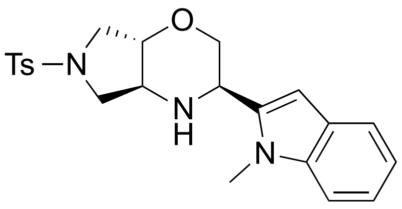

Synthesized according to General Procedure B, using 158 mg (0.23 mmol) of amino-DHP reagent **1i**. White fluffy solid. 60% yield, 6:1 dr. ^{1}**H-NMR** (400 MHz, methanol-d_4), δ, ppm: 2.53 (*s*, 3H), 2.86 (*td*, *J* 10.0, 7.4 Hz, 1H), 3.02-3.09 (*m*, 1H), 3.12 (*t*, *J* 9.7 Hz, 1H), 3.38-3.47 (*m*, 1H), 3.62 (*t*, *J* 11.0 Hz, 1H), 3.66-3.76 (*m*, 2H), 3.82 (*s*, 3H), 4.08 (*dd*, *J* 11.4, 3.4 Hz, 1H), 4.21 (*s*, 1H), 4.26 (*dd*, *J* 10.6, 3.2 Hz, 1H), 6.50 (*s*, 1H), 7.04-7.12 (*m*, 1H), 7.20 (*t*, *J* 7.3 Hz, 1H), 7.45 (*d*, *J* 8.2 Hz, 1H), 7.55 (*d*, *J* 8.0 Hz, 3H), 7.85 (*d*, *J* 8.2 Hz, 2H). ^{13}**C-NMR** (101 MHz, methanol-d_4), δ, ppm: 22.0, 30.7, 49.7, 50.3, 53.5, 60.8, 72.7, 79.6, 100.8, 110.7, 120.7, 121.6, 122.7, 128.8, 128.9, 131.4, 135.4, 139.0, 139.5, 145.5. **MS**: *m/z* calcd. for $C_{22}H_{25}N_3O_3S$ $[M+H]^+$ 412.2, found 412.4.

3-(4-Methoxyphenyl)thiomorpholine, TFA salt (4ja)

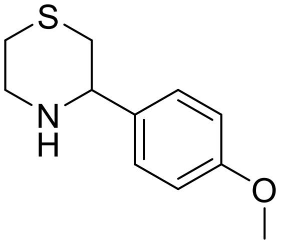

Synthesized according to General Procedure A, using 115 mg (0.24 mmol) of amino-DHP reagent **1j**. White fluffy solid. 73% yield. ^{1}**H-NMR** (400 MHz, methanol-d_4), δ, ppm: 2.76-2.87 (*m*, 2H), 3.11-3.20 (*m*, 1H), 3.32-3.40 (*m*, 1H), 3.42-3.52 (*m*, 1H), 3.70-3.76 (*m*, 1H), 3.83 (*br. s*, 3H), 4.44 (*dd*, *J* 11.7, 2.3 Hz, 1H), 4.89 (*s*, 5H), 7.0 (*d*, *J* 8.8 Hz, 2H), 7.42 (*d*, *J* 8.8 Hz, 2H). ^{13}**C-NMR** (101 MHz, methanol-d_4), δ, ppm: 24.7, 31.6, 48.6, 56.0, 62.8, 115.9, 129.81, 129.84, 162.4. ^{19}**F-NMR** (376 MHz, methanol-d_4), δ, ppm: −76.92 (*s*, 3F). **MS**: *m/z* calcd. for $C_{11}H_{16}NOS$ $[M+H]^+$ 210.1, found 210.3.

3-(1-Benzyl-1H-imidazol-5-yl)thiomorpholine, Free base (4jq)

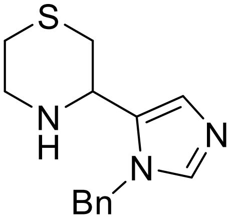

Synthesized according to General Procedure B, using 99 mg (0.18 mmol) of amino-DHP reagent **1j**. White fluffy solid. 79% yield. ^{1}**H-NMR** (400 MHz, methanol-d_4), δ, ppm: 2.21 (*br. d*, *J* 13.3 Hz, 1H), 2.32 (*br. dd*, *J* 13.5, 1.8 Hz, 1H), 2.66-2.78 (*m*, 2H), 2.87-2.96 (*m*, 1H), 3.27 (*dt*, *J* 12.8, 2.8 Hz, 1H), 3.85 (*dd*, *J* 10.5, 2.2 Hz, 1H), 5.32 (*s*, 2H), 7.00 (*s*, 1H), 7.18 (*d*, *J* 7.2 Hz, 2H), 7.31-7.40 (*m*, 3H), 7.73 (*s*, 1H). ^{13}**C-NMR** (101 MHz, methanol-d_4), δ, ppm: 27.6, 33.0, 49.5, 49.9, 53.9, 126.9, 128.3, 129.4, 130.3, 135.6, 138.4, 139.5. **MS**: *m/z* calcd. for $C_{14}H_{18}N_3S$ $[M+H]^+$: 260.1, found 260.1.

3-(Pyrazolo[1,5-a]pyridin-7-yl)thiomorpholine, Free base (4jy)

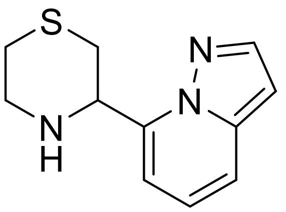

Synthesized according to General Procedure B, using 80 mg (0.16 mmol) of amino-DHP reagent **1j**. White fluffy solid. 61% yield. ^{1}**H-NMR** (400 MHz, methanol-d_4), δ, ppm: 2.50 (*br. d*, *J* 13.6 Hz, 1H), 2.87-3.00 (*m*, 3H), 3.20 (*td*, *J* 12.3, 2.5 Hz, 1H), 3.47 (*dt*, *J* 12.8, 3.0 Hz, 1H), 4.76 (*dd*, *J* 8.6, 4.1 Hz, 1H), 6.63 (*d*, *J* 2.2 Hz, 1H), 6.92 (*d*, *J* 7.0 Hz, 1H), 7.21 (*dd*, *J* 8.8, 7.1 Hz, 1H), 7.60 (*d*, *J* 8.9 Hz, 1H), 7.98 (*d*, *J* 2.3

Hz, 1H). **^{13}C-NMR** (101 MHz, methanol-d_4), δ, ppm: 25.2, 28.2, 48.4, 59.1, 99.6, 113.3, 121.0, 124.7, 135.2, 142.4, 143.1. **MS**: *m/z* calcd. for $C_{11}H_{14}N_3S$ $[M+H]^+$ 220.1, found 220.0.

rac-3-(4-Methoxyphenyl)-5-methylthiomorpholine, Free base (4ka)

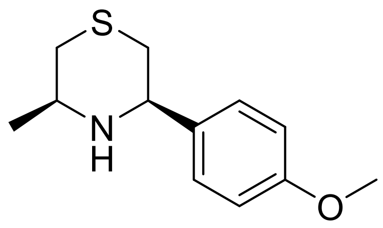

Synthesized according to General Procedure B, using 87 mg (0.17 mmol) of amino-DHP reagent **1k**. White fluffy solid. 63% yield, >14:1 dr. **^{1}H-NMR** (400 MHz, methanol-d_4), δ, ppm: 1.16 (*d*, *J* 6.4 Hz, 3H), 2.36-2.43 (*m*, 2H), 2.45-2.53 (*m*, 1H), 2.73 (*dd*, *J* 13.3, 11.0 Hz, 1H), 3.04-3.14 (*m*, 1H), 3.77 (*s*, 3H), 3.91 (*dd*, *J* 10.9, 2.1 Hz, 1H), 6.89 (*d*, *J* 8.7 Hz, 2H), 7.30 (*d*, *J* 8.6 Hz, 2H). **^{13}C-NMR** (101 MHz, methanol-d_4), δ, ppm: 21.2, 32.4, 32.7, 54.3, 54.6, 63.1, 113.5, 127.5, 135.8, 159.2. **MS**: *m/z* calcd. for $C_{12}H_{18}NOS$ $[M+H]^+$ 224.1, found 224.1.

rac-(3S,5R)-3-Methyl-5-(1-methyl-1H-pyrazol-5-yl)thiomorpholine, Free base (4kp)

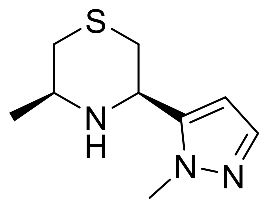

Synthesized according to General Procedure B, using 86 mg (0.18 mmol) of amino-DHP reagent **1k**. White fluffy solid. 65% yield, >14:1 dr. **^{1}H-NMR** (400 MHz, methanol-d_4), δ, ppm: 1.42 (*d*, *J* 6.6 Hz, 3H), 2.80-2.87 (*m*, 1H), 2.90-3.00 (*m*, 2H), 3.21 (*dd*, *J* 14.6, 11.6 Hz, 1H), 3.72-3.83 (*m*, 1H), 3.96 (*s*, 3H), 4.82 (*dd*, *J* 11.6, 2.6 Hz, 1H), 6.56 (*d*, *J* 2.0 Hz, 1H), 7.52 (*d*, *J* 1.8 Hz, 1H). **^{13}C-NMR** (101 MHz, methanol-d_4), δ, ppm: 19.6, 30.4, 31.1, 37.2, 55.2, 57.7, 106.4, 139.5, 140.0. **MS**: *m/z* calcd. for $C_9H_{16}N_3S$ $[M+H]^+$ 198.1, found 198.1.

rac-5-(4-Methoxyphenyl)-2-methylthiomorpholine, Free base (4la)

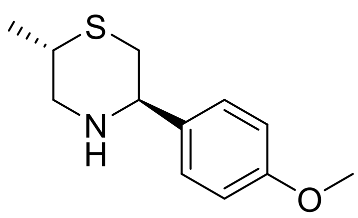

Synthesized according to General Procedure B, using 93 mg (0.19 mmol) of amino-DHP reagent **1l**. White fluffy solid. 70% yield, 11:1 dr. **^{1}H-NMR** (400 MHz, methanol-d_4), δ, ppm: 1.13 (*d*, *J* 6.7 Hz, 3H), 2.47 (*dd*, *J* 13.4, 2.1 Hz, 1H), 2.71 (*dd*, *J* 12.4, 10.9 Hz, 1H), 2.85-2.92 (*m*, 1H), 2.93-3.02 (*m*, 1H), 3.31-3.36 (*m*, 1H), 3.72-3.76 (*m*, 1H), 3.77 (*s*, 3H), 6.85-6.91 (*m*, 2H), 7.28 (*d*, *J* 8.7 Hz, 2H). **^{13}C-NMR** (101 MHz, methanol-d_4), δ, ppm: 18.8, 35.8, 36.0, 55.8, 57.9, 62.8, 115.1, 128.9, 136.9, 160.8. **MS**: *m/z* calcd. for $C_{12}H_{18}NOS$ $[M+H]^+$ 224.1, found 224.1.

rac-tert-butyl 4-((3R,6S)-6-Methylthiomorpholin-3-yl)indoline-1-carboxylate, Free base (4ll)

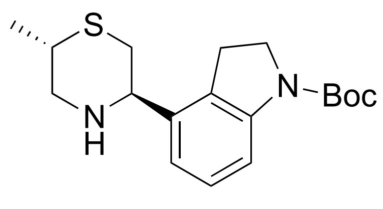

Synthesized according to General Procedure B, using 104 mg (0.17 mmol) of amino-DHP reagent **1l**. White fluffy solid. 73% yield, 13:1 dr. **^{1}H-NMR** (400 MHz, methanol-d_4), δ, ppm: 1.13 (*d*, *J* 6.7 Hz, 3H), 1.56 (*s*, 9H), 2.48 (*dd*, *J* 13.5, 2.0 Hz, 1H), 2.7 (*dd*, *J* 12.5, 10.9 Hz, 1H), 2.88 (*dd*, *J* 13.5, 10.9 Hz, 1H), 2.94-3.04 (*m*, 1H), 3.06-3.20 (*m*, 2H), 3.33 (*br. d*, *J* 2.7 Hz, 1H), 3.83 (*dd*, *J* 10.8, 2.1 Hz, 1H), 3.91-4.03 (*m*, 2H), 7.01 (*d*, *J* 7.7 Hz, 1H), 7.16 (*t*, *J* 7.9 Hz, 1H), 7.66 (*br. s*, 1H). **^{13}C-**

NMR (101 MHz, methanol-d_4), δ, ppm: 18.8, 26.8 (*br. s*), 28.8, 34.7, 35.9, 57.8, 60.1, 82.1 (*br. s*), 114.9 (*br. s*), 120.3, 129.2, 130.5 (*br. s*), 140.7 (*br. s*), 144.1 (*br. s*), 154.2 (*br. s*). **MS**: *m/z* calcd. for $C_{18}H_{27}N_2O_2S$ $[M+H]^+$ 335.2, found 335.3.

(2S,5R)-5-(6-(4-Methoxyphenyl)pyridin-2-yl)-2-methylthiomorpholine, Free base (4lγ)

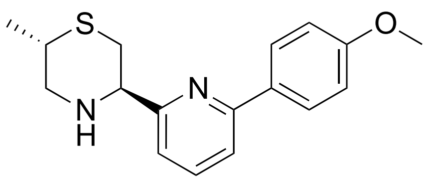

Synthesized according to General Procedure B, using 145 mg (0.25 mmol) of amino-DHP reagent **1l**. White fluffy solid. 75% yield, >14:1 dr. **^{1}H-NMR** (400 MHz, methanol-d_4), δ, ppm: 1.23 (*d*, *J* 6.4 Hz, 3H), 2.40-2.56 (*m*, 2H), 2.68-2.81 (*m*, 2H), 3.06-3.21 (*m*, 1H), 3.84 (*s*, 3H), 4.14 (*dd*, *J* 10.3, 3.2 Hz, 1H), 6.99-7.04 (*m*, 2H), 7.28 (*d*, *J* 7.6 Hz, 1H), 7.68 (*d*, *J* 7.8 Hz, 1H), 7.74-7.82 (*m*, 1H), 7.97-8.03 (*m*, 2H). **^{13}C-NMR** (101 MHz, methanol-d_4), δ, ppm: 22.8, 32.8, 34.0, 55.2, 55.9, 64.6, 115.2, 119.9, 129.5, 133.1, 139.2, 158.0, 162.1, 162.3. **MS**: *m/z* calcd. for $C_{17}H_{21}N_2OS$ $[M+H]^+$ 301.1, found 301.2.

Benzyl 3-(4-methoxyphenyl)piperazine-1-carboxylate, Free base (4ma)

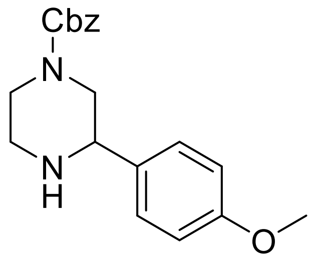

Synthesized according to General Procedure B, using 108 mg (0.18 mmol) of amino-DHP reagent **1m**. White fluffy solid. 80% yield. **^{1}H-NMR** (400 MHz, methanol-d_4), δ, ppm: 2.88 (*br. s*, 2H), 2.92-3.08 (*m*, 2H), 3.61 (*br. d*, *J* 9.1 Hz, 1H), 3.76 (*s*, 3H), 4.07 (*br. d*, *J* 10.0 Hz, 2H), 5.14 (*s*, 2H), 6.89 (*br. d*, *J* 8.6 Hz, 2H), 7.25-7.39 (*m*, 7H). **^{13}C-NMR** (101 MHz, methanol-d_4), δ, ppm: 44.9 (*br. d*, *J* 22 Hz), 46.8, 51.9 (*br. d*, *J* 19.8 Hz), 55.8, 60.7, 68.5, 115.1, 129.1, 129.25, 129.31, 129.7, 134.1, 138.2, 157.0, 161.0. **MS**: *m/z* calcd. for $C_{19}H_{23}N_2O_3$ $[M+H]^+$ 327.2, found 327.2.

Benzyl 3-(5-bromopyridin-3-yl)piperazine-1-carboxylate, Free base (4mr)

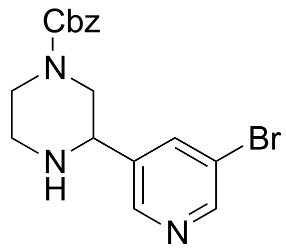

Synthesized according to General Procedure B, using 112 mg (0.17 mmol) of amino-DHP reagent **1m**. White fluffy solid. 73% yield. **^{1}H-NMR** (400 MHz, methanol-d_4), δ, ppm: 2.76-3.08 (*m*, 4H), 3.78 (*br. dd*, *J* 10.5, 2.5 Hz, 1H), 4.08 (*br. t*, *J* 12.5 Hz, 2H), 5.14 (*s*, 2H), 7.25-7.42 (*m*, 5H), 8.09 (*br. s*, 1H), 8.51-8.61 (*m*, 2H). **^{13}C-NMR** (101 MHz, methanol-d_4), δ, ppm: 45.1 (*br. s*), 46.4 (*br. s*), 51.2 (*br. s*), 58.1, 68.7, 122.1, 129.1, 129.4, 129.7, 138.1, 139.5, 140.5, 148.1, 151.0, 156.9. **MS**: *m/z* calcd. for $C_{17}H_{18}BrN_3O_2$ $[M+H]^+$ 376.1, found 376.3.

Benzyl 3-cyclopropylpiperazine-1-carboxylate, Free base (4mα)

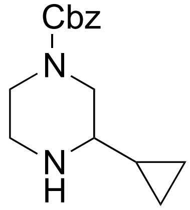

Synthesized according to General Procedure B, using 76 mg (0.14 mmol) of amino-DHP reagent **1m**. White fluffy solid. 61% yield. **^{1}H-NMR** (400 MHz, methanol-d_4), δ, ppm: 0.43 (*br. s*, 1H), 0.54 (*br. dd*, *J* 9.4, 4.5 Hz, 1H), 0.65-0.80 (*m*, 2H), 0.90-1.01 (*m*, 1H), 2.51 (*td*, *J* 10.2, 3.6 Hz, 1H), 3.06 (*td*, *J* 12.0, 3.4 Hz, 1H), 3.20-3.29 (*m*, 1H), 3.31-3.44 (*m*, 2H), 4.10-

4.32 (*m*, 2H), 5.10-5.20 (*m*, 2H), 7.30-7.40 (*m*, 5H). **^{13}C-NMR** (101 MHz, methanol-d_4), δ, ppm: 4.1, 11.7, 41.7 (*br. s*), 44.2 (*br. s*), 46.9 (*br. s*), 61.3, 69.1, 129.4, 129.5, 129.8, 137.8, 156.4. **MS**: *m/z* calcd. for $C_{15}H_{21}N_2O_2$ $[M+H]^+$ 261.2, found 261.2.

Benzyl 3-(3-phenylisoxazol-5-yl)piperazine-1-carboxylate, Free base (4mx)

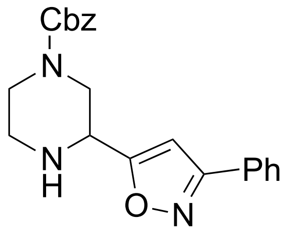

Synthesized according to General Procedure B, using 98 mg (0.15 mmol) of amino-DHP reagent **1m**. White fluffy solid. 83% yield. **^{1}H-NMR** (400 MHz, methanol-d_4), δ, ppm: 3.31-3.36 (*m*, 1H), 3.40-3.51 (*m*, 1H), 3.51-3.62 (*m*, 1H), 3.82 (*q*, *J* 6.5 Hz, 2H), 4.17 (*br. d*, *J* 14.4 Hz, 1H), 4.44 (*br. d*, *J* 13.8 Hz, 1H), 4.87 (*dd*, *J* 9.4, 3.7 Hz, 1H), 5.11-5.26 (*m*, 2H), 7.11 (*s*, 1H), 7.27-7.42 (*m*, 5H), 7.46-7.54 (*m*, 3H), 7.78-7.89 (*m*, 2H). **^{13}C-NMR** (101 MHz, methanol-d_4), δ, ppm: 41.8 (*br. s*), 44.0, 45.1 (*br. s*), 51.2 (*br. s*), 69.3, 104.0, 128.1, 129.4, 129.5, 129.6, 129.8, 130.4, 131.9, 137.7, 156.3, 164.5, 166.3. **MS**: *m/z* calcd. for $C_{21}H_{22}N_3O_3$ $[M+H]^+$ 364.2, found 364.3.

3-(4-Methoxyphenyl)-1-tosylpiperazine, Free base (4na)

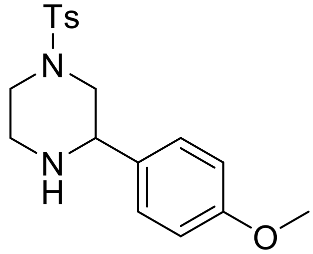

Synthesized according to General Procedure B, using 73 mg (0.12 mmol) of amino-DHP reagent **1n**. White fluffy solid. 76% yield. **^{1}H-NMR** (400 MHz, methanol-d_4), δ, ppm: 2.17 (*t*, *J* 10.9 Hz, 1H), 2.37 (*td*, *J* 11.5, 3.1 Hz, 1H), 2.44 (*s*, 3H), 2.94 (*td*, *J* 12.0, 3.1 Hz, 1H), 3.02-3.14 (*m*, 1H), 3.58-3.70 (*m*, 2H), 3.73-3.80 (*m*, 4H), 6.88 (*d*, *J* 8.7 Hz, 2H), 7.23 (*d*, *J* 8.7 Hz, 2H), 7.42 (*d*, *J* 8.1 Hz, 2H), 7.63 (*d*, *J* 8.2 Hz, 2H). **^{13}C-NMR** (101 MHz, methanol-d_4), δ, ppm: 21.6, 46.4, 47.1, 54.0, 55.8, 60.4, 115.2, 129.1, 129.3, 131.1, 133.8, 134.1, 145.6, 161.1. **MS**: *m/z* calcd. for $C_{18}H_{23}N_2O_3S$ $[M+H]^+$ 347.1, found 347.2.

(5S)-3-(4-Methoxyphenyl)-5-methyl-1-tosylpiperazine, Free base (4oa)

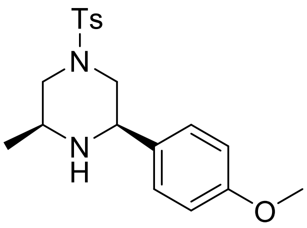

Synthesized according to General Procedure B, using 95 mg (0.15 mmol) of amino-DHP reagent **1o**. White fluffy solid. 86% yield, 10:1 dr. **^{1}H-NMR** (400 MHz, methanol-d_4), δ, ppm: 1.11 (*d*, *J* 6.2 Hz, 3H), 1.94 (*t*, *J* 10.8 Hz, 1H), 2.04-2.12 (*m*, 1H), 2.48 (*s*, 3H), 3.01 (*ddd*, *J* 9.8, 6.5, 2.9 Hz, 1H), 3.65 (*br. t*, *J* 11.4 Hz, 2H), 3.78-3.90 (*m*, 4H), 6.93 (*m*, *J* 8.6 Hz, 2H), 7.32 (*d*, *J* 8.6 Hz, 2H), 7.48 (*m*, *J* 8.0 Hz, 2H), 7.67 (*d*, *J* 8.1 Hz, 2H). **^{13}C-NMR** (101 MHz, methanol-d_4), δ, ppm: 19.4, 21.9, 52.0, 53.2, 53.7, 56.0, 60.5, 115.2, 129.1, 129.5, 131.2, 134.1, 134.3, 145.5, 160.8. **MS**: *m/z* calcd. for $C_{19}H_{25}N_2O_3S$ $[M+H]^+$ 361.2, found 361.4.

1-Methyl-6-((6S)-6-methyl-4-tosylpiperazin-2-yl)-1H-indole, TFA salt (4os)

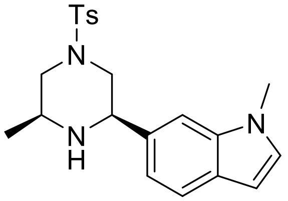

Synthesized according to General Procedure A, using 91 mg (0.14 mmol) of amino-DHP reagent **1o**. White fluffy solid. 64% yield, 12:1 dr. **^{1}H-NMR** (400 MHz, methanol-d_4), δ, ppm: 1.38 (*d*, *J* 6.6 Hz, 3H), 2.46 (*s*, 3H), 2.54 (*dd*, *J* 12.8, 11.6 Hz, 1H), 2.92 (*t*, *J* 12.4 Hz, 1H), 3.64-3.73 (*m*, 1H), 3.82 (*s*, 3H), 4.07 (*br. d*, *J* 13.1 Hz, 2H), 4.63 (*dd*, *J* 11.4, 3.1 Hz, 1H), 6.47 (*d*, *J* 2.8 Hz, 1H), 7.09 (*dd*, *J* 8.3, 1.3 Hz, 1H), 7.27 (*d*, *J* 3.2 Hz, 1H), 7.47 (*m*, *J* 8.1 Hz, 2H), 7.51-7.56 (*m*, 1H), 7.63 (*d*, *J* 8.2 Hz, 1H), 7.73 (*d*, *J* 8.2 Hz, 2H). **^{13}C-NMR** (101 MHz, methanol-d_4), δ, ppm: 15.9, 21.7, 33.1, 49.8, 50.1, 54.2, 62.1, 102.1, 110.1, 119.2, 122.8, 126.8, 129.1, 131.5, 131.5, 132.4, 134.0, 138.2, 146.3. **^{19}F-NMR** (376 MHz, methanol-d_4), δ, ppm: −76.88 (s, 3F). **MS**: *m/z* calcd. for $C_{21}H_{26}N_3O_2S$ $[M+H]^+$ 384.2, found 384.3.

(5S)-3-Cyclopropyl-5-methyl-1-tosylpiperazine, Free base (4oα)

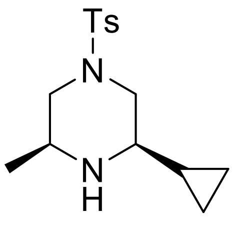

Synthesized according to General Procedure B, using 76 mg (0.13 mmol) of amino-DHP reagent **1o**. White fluffy solid. 55% yield, 13:1 dr. **^{1}H-NMR** (400 MHz, methanol-d_4), δ, ppm: 0.21-0.34 (*m*, 2H), 0.46-0.54 (*m*, 2H), 0.61 (*dt*, *J* 12.2, 4.4 Hz, 1H), 1.04 (*d*, *J* 6.4 Hz, 3H), 1.83 (*t*, *J* 10.9 Hz, 1H), 1.87-1.95 (*m*, 1H), 1.97-2.07 (*m*, 1H), 2.44 (*s*, 3H), 2.79 (*dqd*, *J* 10.0, 6.5, 3.0 Hz, 1H), 3.57 (*dt*, *J* 11.1, 2.2 Hz, 1H), 3.68 (*dd*, *J* 10.9, 2.2 Hz, 1H), 7.43 (*m*, *J* 8.1 Hz, 2H), 7.65 (*d*, *J* 8.2 Hz, 2H). **^{13}C-NMR** (101 MHz, methanol-d_4), δ, ppm: 2.8, 3.7, 14.8, 19.0, 21.6, 51.6, 51.8, 53.3, 62.0, 129.1, 131.0, 134.1, 145.6. **MS**: *m/z* calcd. for $C_{15}H_{23}N_2O_2S$ $[M+H]^+$ 295.1, found 295.1.

rac-5-(4-Methoxyphenyl)-2-methyl-1-tosylpiperazine, Free base (4pa)

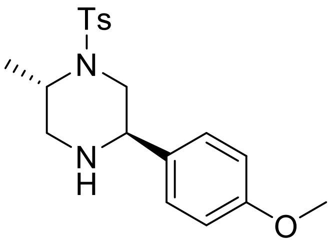

Synthesized according to General Procedure B, using 96 mg (0.15 mmol) of amino-DHP reagent **1p**. White fluffy solid. 66% yield, 7:1 dr. **^{1}H-NMR** (400 MHz, methanol-d_4), δ, ppm: 1.24 (*d*, *J* 7.2 Hz, 3H), 2.46 (*s*, 3H), 3.28 (*s*, 1H), 3.33-3.40 (*m*, 1H), 3.61 (*dd*, *J* 14.6, 11.9 Hz, 1H), 3.82 (*s*, 3H), 4.02 (*dd*, *J* 14.6, 3.6 Hz, 1H), 4.20 (*dd*, *J* 11.9, 3.7 Hz, 1H), 4.50 (*quint.*, *J* 6.0 Hz, 1H), 6.99-7.07 (*m*, 2H), 7.37-7.51 (*m*, 4H), 7.79 (*d*, *J* 8.3 Hz, 2H). **^{13}C-NMR** (101 MHz, methanol-d_4), δ, ppm: 14.6, 21.7, 43.2, 47.2, 48.9, 56.0, 59.7, 116.0, 126.0, 128.5, 130.5, 131.5, 138.4, 146.0, 162.8. **MS**: *m/z* calcd. for $C_{19}H_{25}N_2O_3S$ $[M+H]^+$ 361.2, found 361.2.

rac-5-(5-Methyl-4-tosylpiperazin-2-yl)isoquinoline, Free base (4pj)

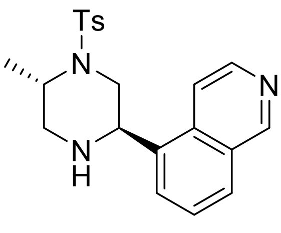

Synthesized according to General Procedure B, using 102 mg (0.15 mmol) of amino-DHP reagent **1p**. White fluffy solid. 59% yield, >14:1 dr. **^{1}H-NMR** (400 MHz, methanol-d_4), δ, ppm: 1.27 (*d*, *J* 6.9 Hz, 3H), 2.45 (*s*, 3H), 2.98-3.13 (*m*, 2H), 3.23 (*dd*, *J* 12.4, 3.9 Hz, 1H), 3.80 (*dd*, *J* 13.3, 2.9 Hz, 1H), 4.24-4.34 (*m*, 1H), 4.44 (*dd*, *J*

10.8, 2.9 Hz, 1H), 7.43 (*d*, *J* 8.1 Hz, 2H), 7.68-7.78 (*m*, 3H), 7.90 (*d*, *J* 6.1 Hz, 1H), 8.06 (*dd*, *J* 13.8, 7.70 Hz, 2H), 8.48 (*d*, *J* 6.1 Hz, 1H), 9.26 (*s*, 1H). **^{13}C-NMR** (101 MHz, methanol-d_4), *δ*, ppm: 15.1, 21.6, 47.3, 49.7, 52.0, 56.9, 117.7, 128.5, 128.9, 129.5, 130.2, 130.5, 131.2, 135.1, 136.9, 139.4, 143.4, 145.4, 154.35. **MS**: *m/z* calcd. for $C_{21}H_{24}N_3O_2S$ [M+H]$^+$ 382.2, found 382.2.

3-(Benzo[b]thiophen-2-yl)-1,4-oxazepane, Free base (4qu)

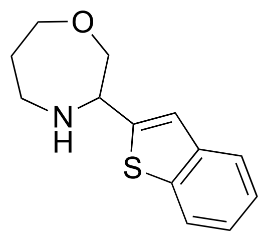

Synthesized according to General Procedure B, using 120 mg (0.23 mmol) of amino-DHP reagent **1q**. White fluffy solid. 59% yield. **^{1}H-NMR** (400 MHz, methanol-d_4), *δ*, ppm: 1.99-2.08 (*m*, 2H), 3.08 (*ddd*, *J* 13.6, 8.4, 5.0 Hz, 1H), 3.26 (*dt*, *J* 13.9, 5.0 Hz, 1H), 3.79-3.89 (*m*, 2H), 3.92-3.99 (*m*, 1H), 4.11 (*dd*, *J* 12.8, 3.8 Hz, 1H), 4.5 (*dd*, *J* 8.7, 3.7 Hz, 1H), 7.27-7.38 (*m*, 3H), 7.72-7.78 (*m*, 1H), 7.80-7.85 (*m*, 1H). **^{13}C-NMR** (101 MHz, methanol-d_4), *δ*, ppm: 32.7, 46.4, 62.0, 71.1, 77.1, 123.2, 123.4, 124.7, 125.6, 140.9, 141.1, 144.3. **MS**: *m/z* calcd. for $C_{13}H_{16}NOS$ [M+H]$^+$ 234.1, found 234.1.

NMR and Mass Spectra

Starting Materials

Morpholine-Forming Amino-DHP Reagents

N,N-Dibenzyl-2-(2,2-dimethoxyethoxy)ethan-1-amine

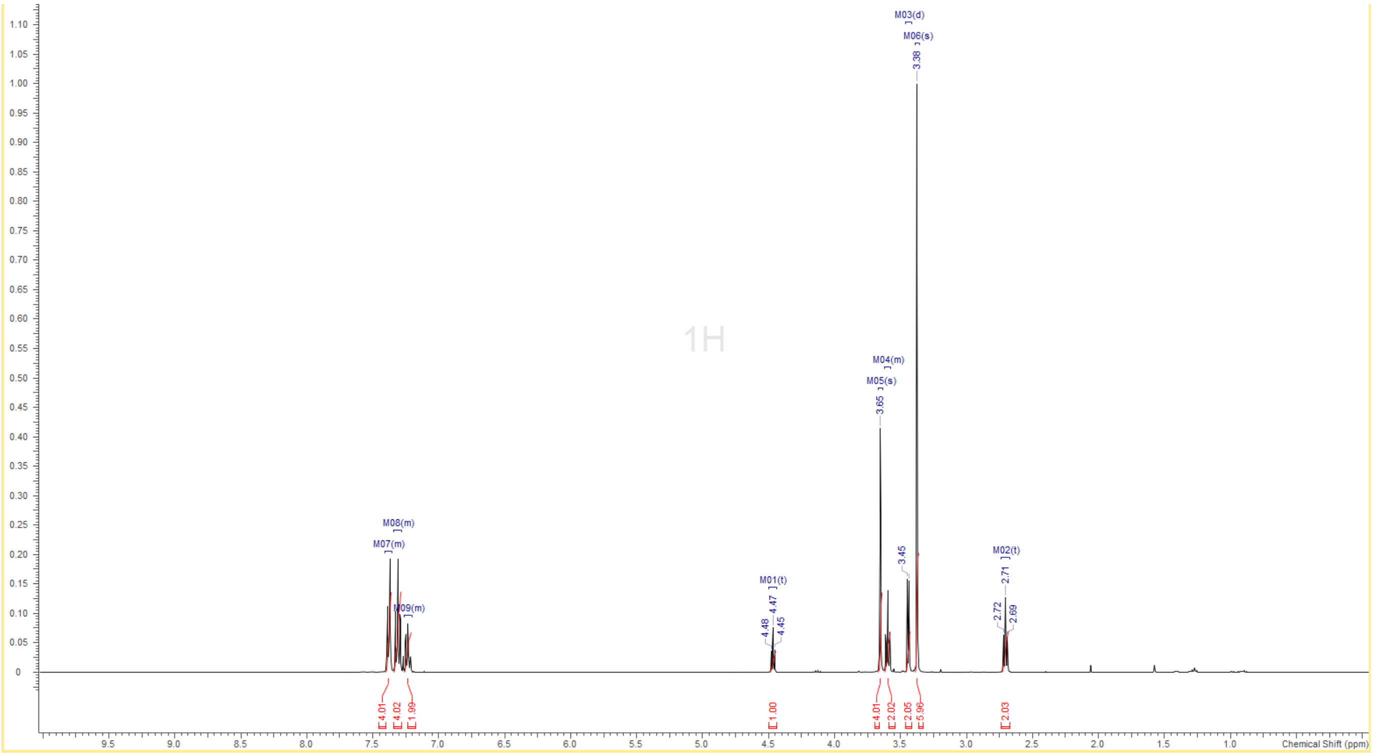

2-(2-(Dibenzylamino)ethoxy)acetaldehyde

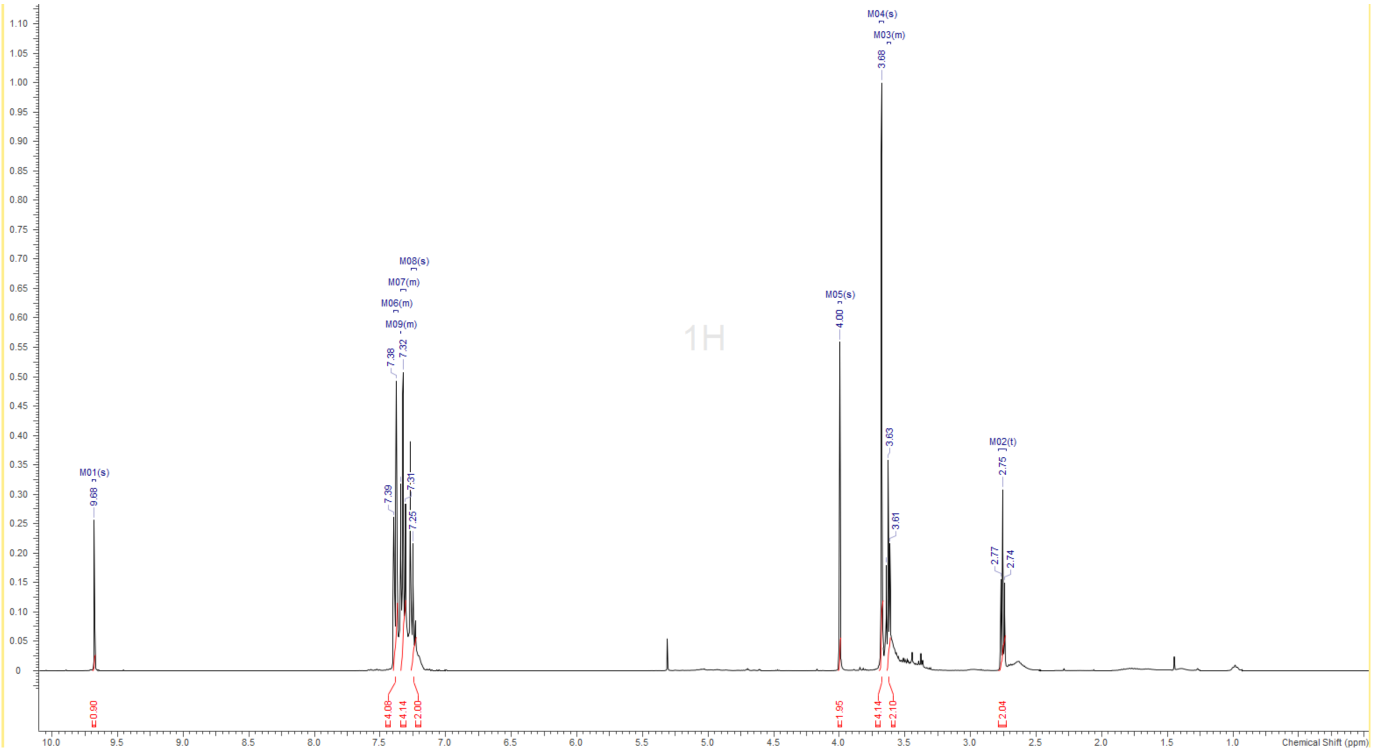

Diethyl 4-((2-(dibenzylamino)ethoxy)methyl)-2,6-dimethyl-1,4-dihydropyridine-3,5-dicarboxylate

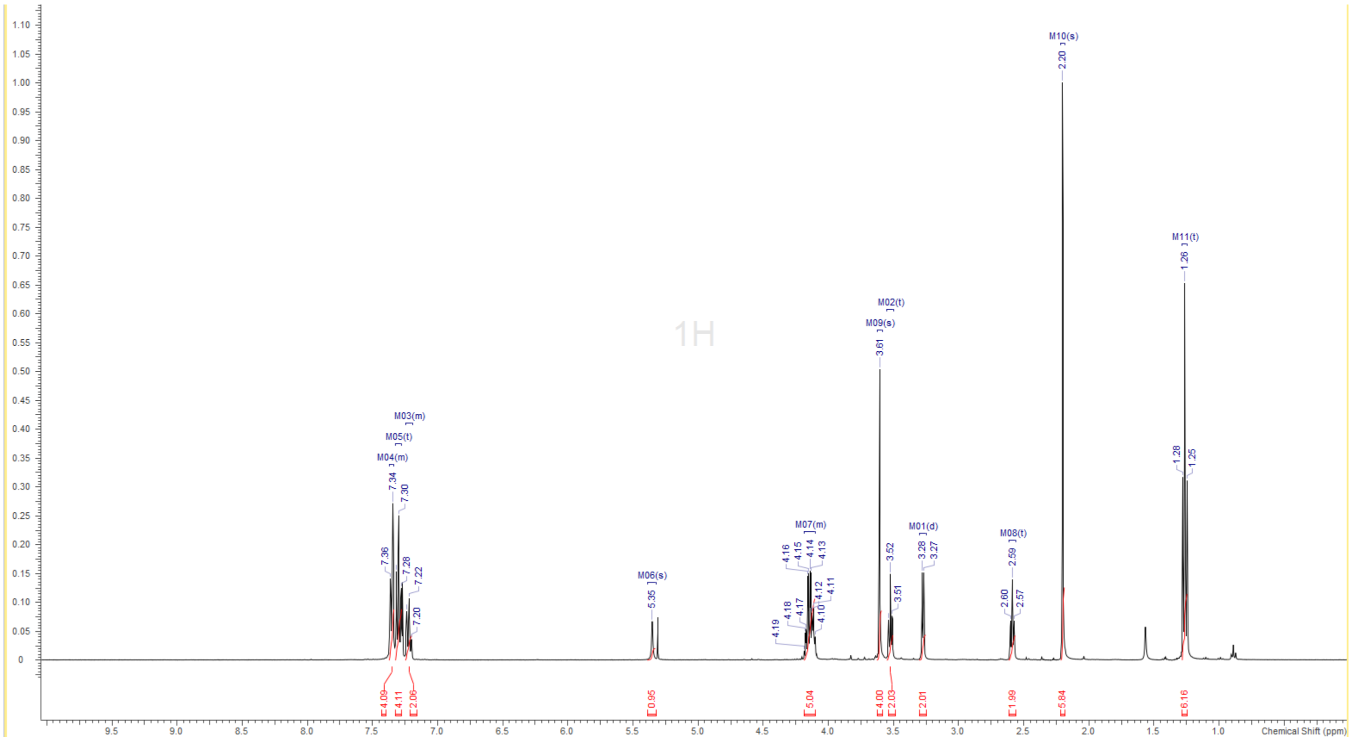

Diethyl 4-((2-aminoethoxy)methyl)-2,6-dimethyl-1,4-dihydropyridine-3,5-dicarboxylate (1a-CO_2Et)

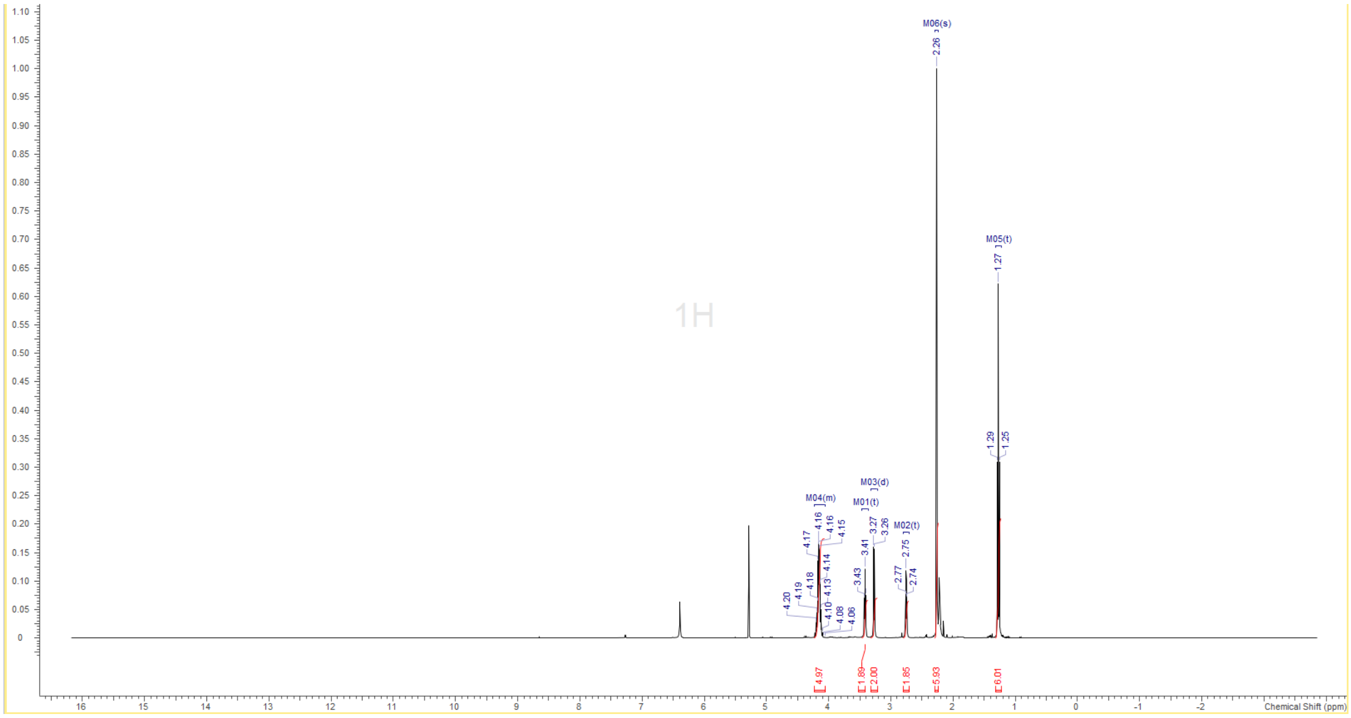
1H
M06(s)
2.26
M05(t)
1.27
1.29
1.25
M04(m)
M03(d)
M01(t)
M02(t)
4.97
1.89
2.00
1.85
5.93
6.01
Chemical Shift (ppm)

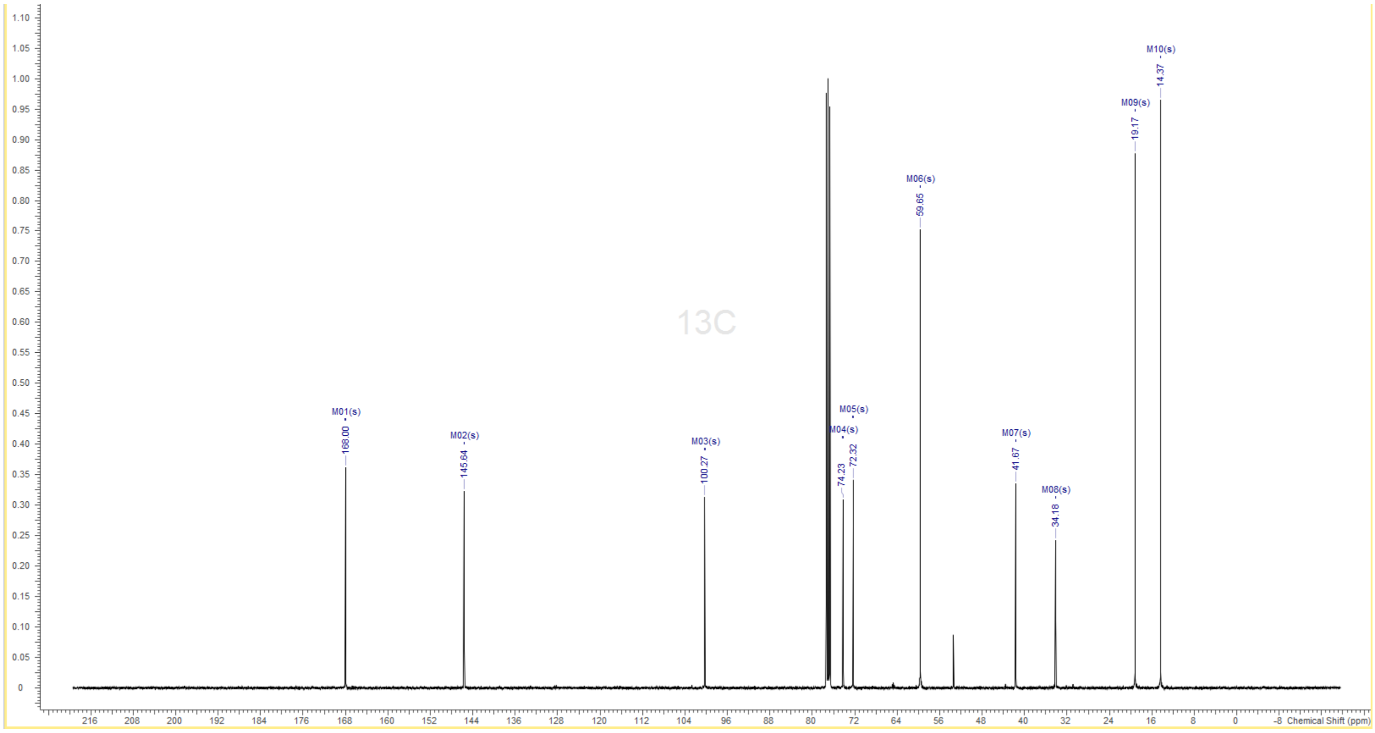
13C
M01(s)
168.00
M02(s)
145.64
M03(s)
100.27
M04(s)
74.23
M05(s)
72.32
M06(s)
59.65
M07(s)
41.67
M08(s)
34.18
M09(s)
19.17
M10(s)
14.37
Chemical Shift (ppm)

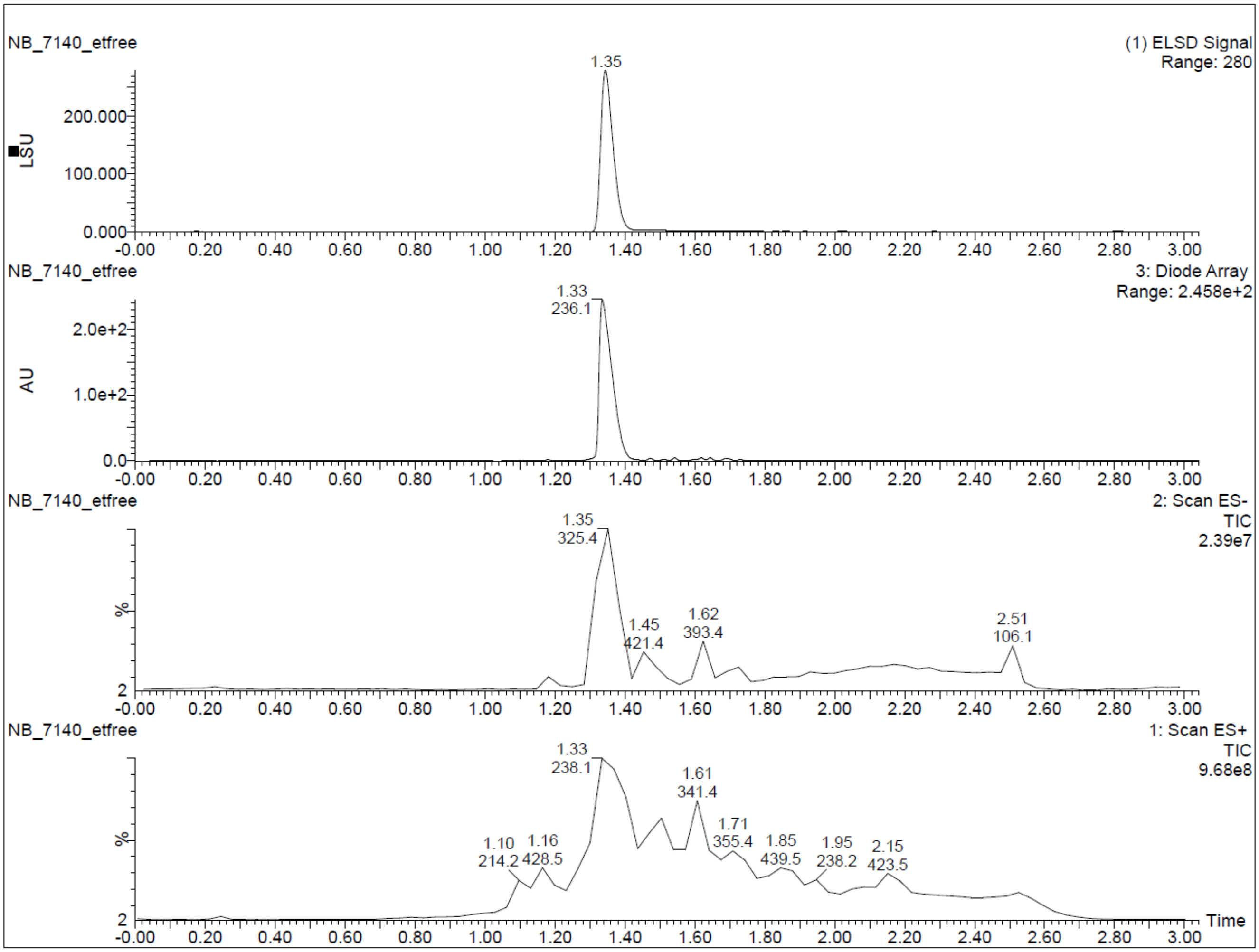

NB_7140_etfree
(1) ELSD Signal
Range: 280
1.35
LSU
200.000
100.000
0.000
-0.00
0.20
0.40
0.60
0.80
1.00
1.20
1.40
1.60
1.80
2.00
2.20
2.40
2.60
2.80
3.00
NB_7140_etfree
3: Diode Array
Range: 2.458e+2
1.33
236.1
AU
2.0e+2
1.0e+2
0.0
NB_7140_etfree
2: Scan ES-
TIC
2.39e7
1.35
325.4
1.45
421.4
1.62
393.4
2.51
106.1
%
2
NB_7140_etfree
1: Scan ES+
TIC
9.68e8
1.33
238.1
1.61
341.4
1.71
355.4
1.10
214.2
1.16
428.5
1.85
439.5
1.95
238.2
2.15
423.5
Time

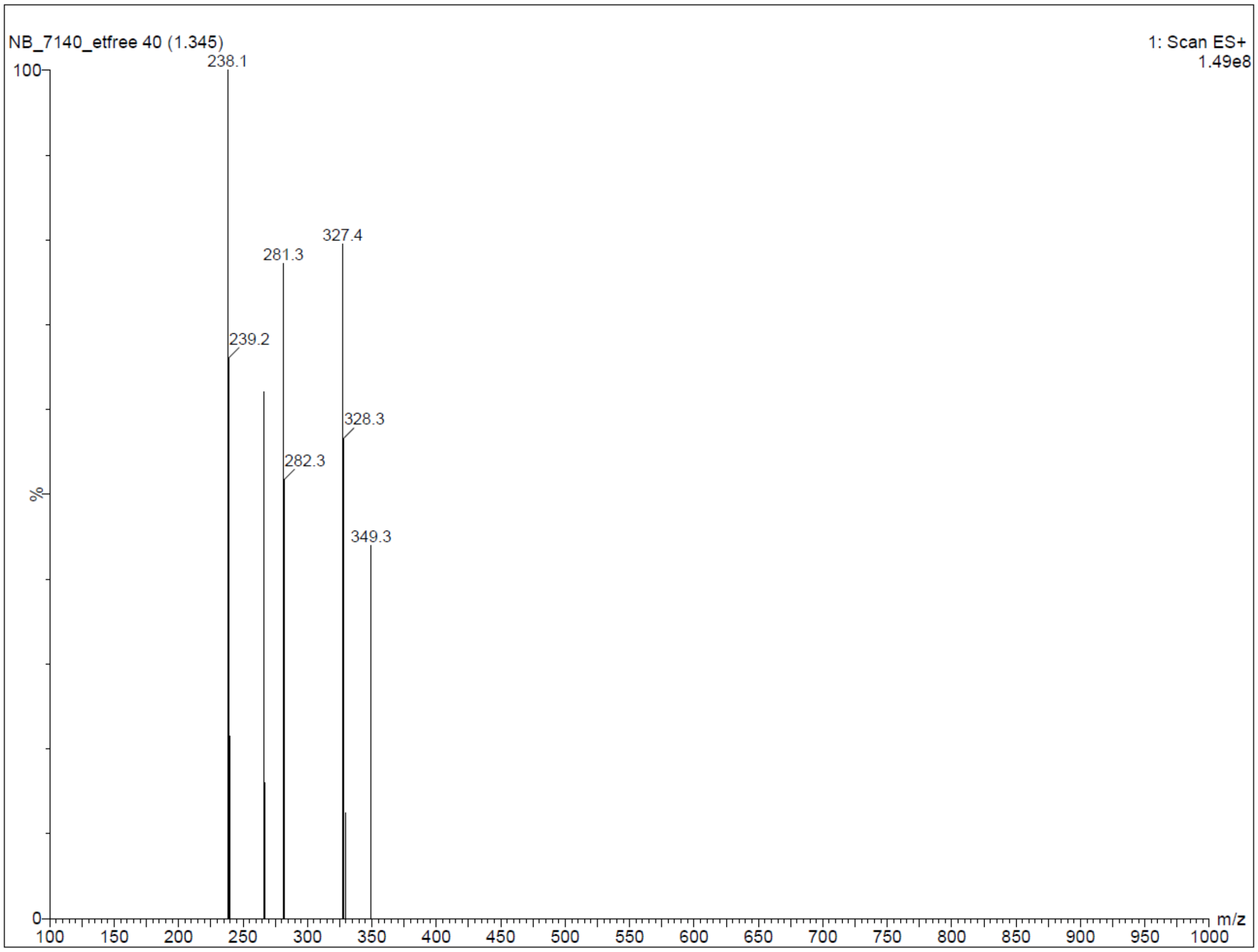

4-((2-Aminoethoxy)methyl)-2,6-dimethyl-1,4-dihydropyridine-3,5-dicarbonitrile (1a-CN)

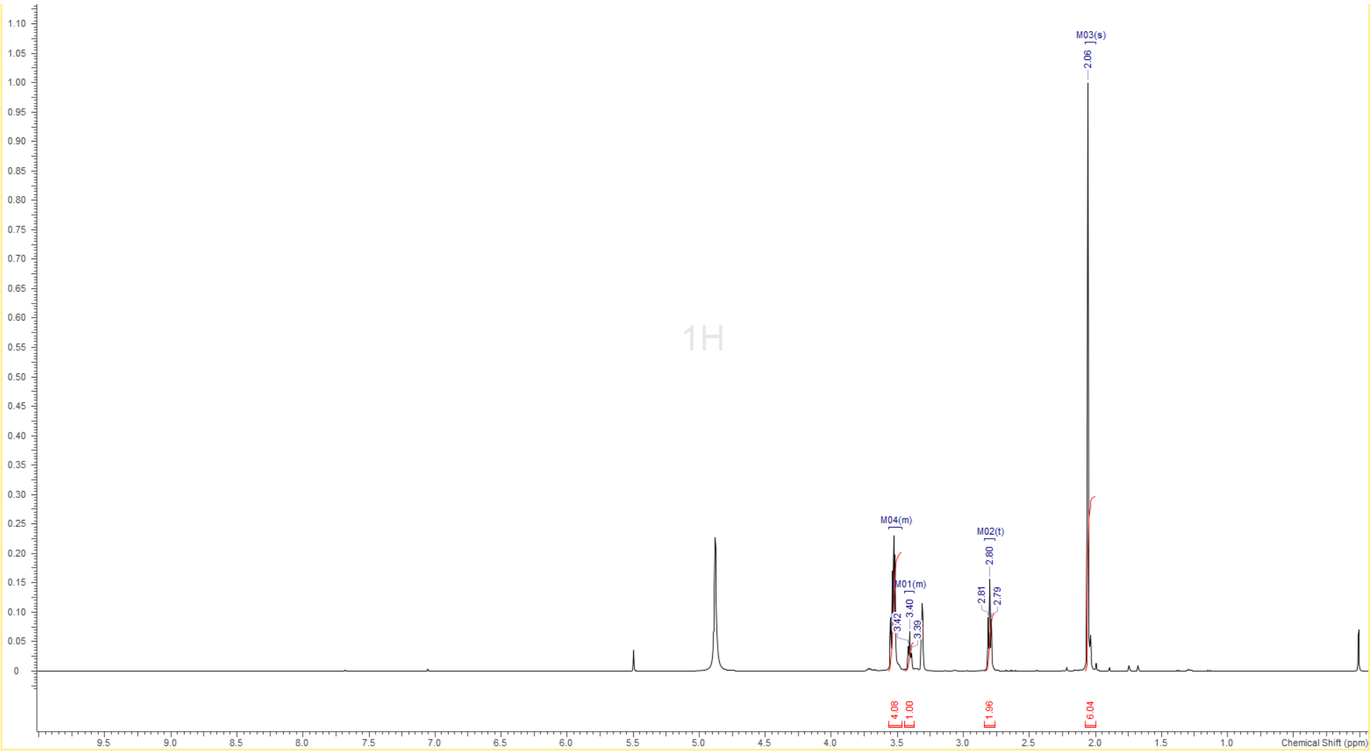

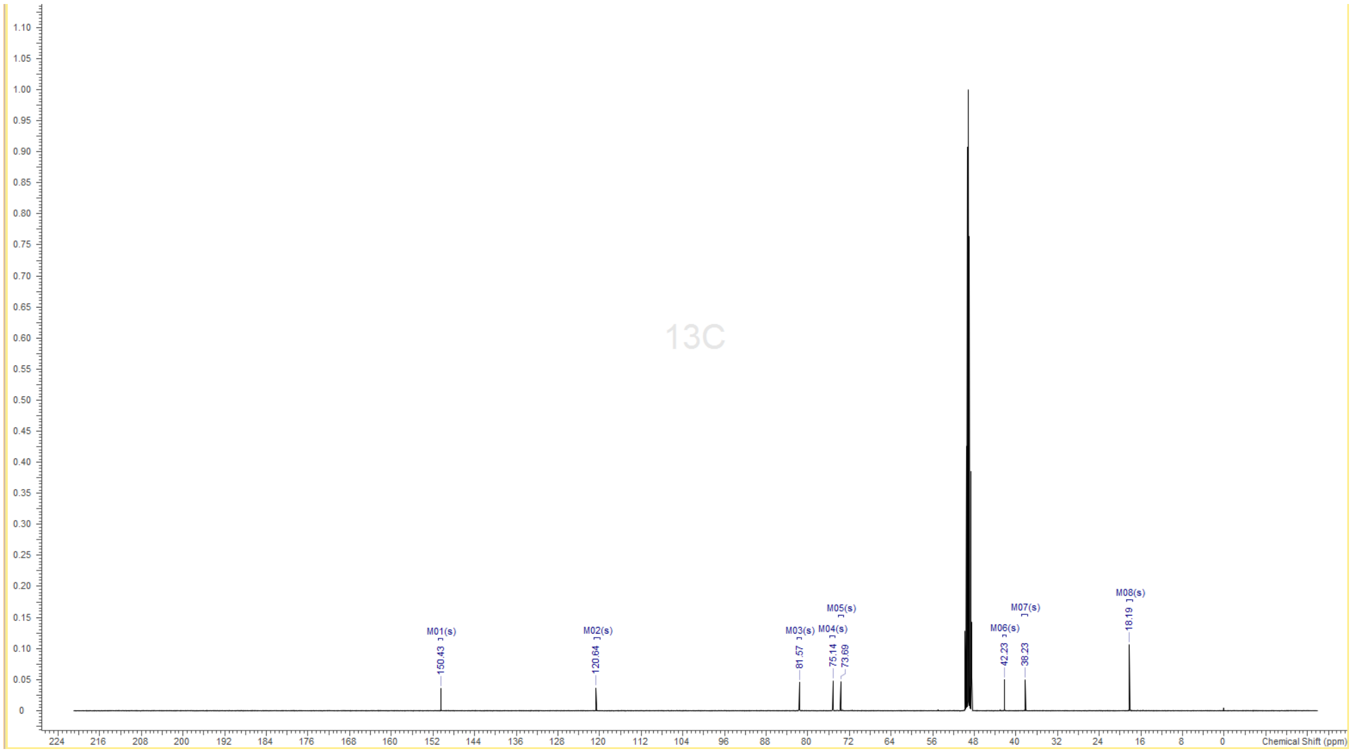
13C
M01(s)
150.43
M02(s)
120.64
M03(s)
81.57
M04(s)
M05(s)
75.14
73.69
M06(s)
42.23
M07(s)
38.23
M08(s)
18.19
Chemical Shift (ppm)

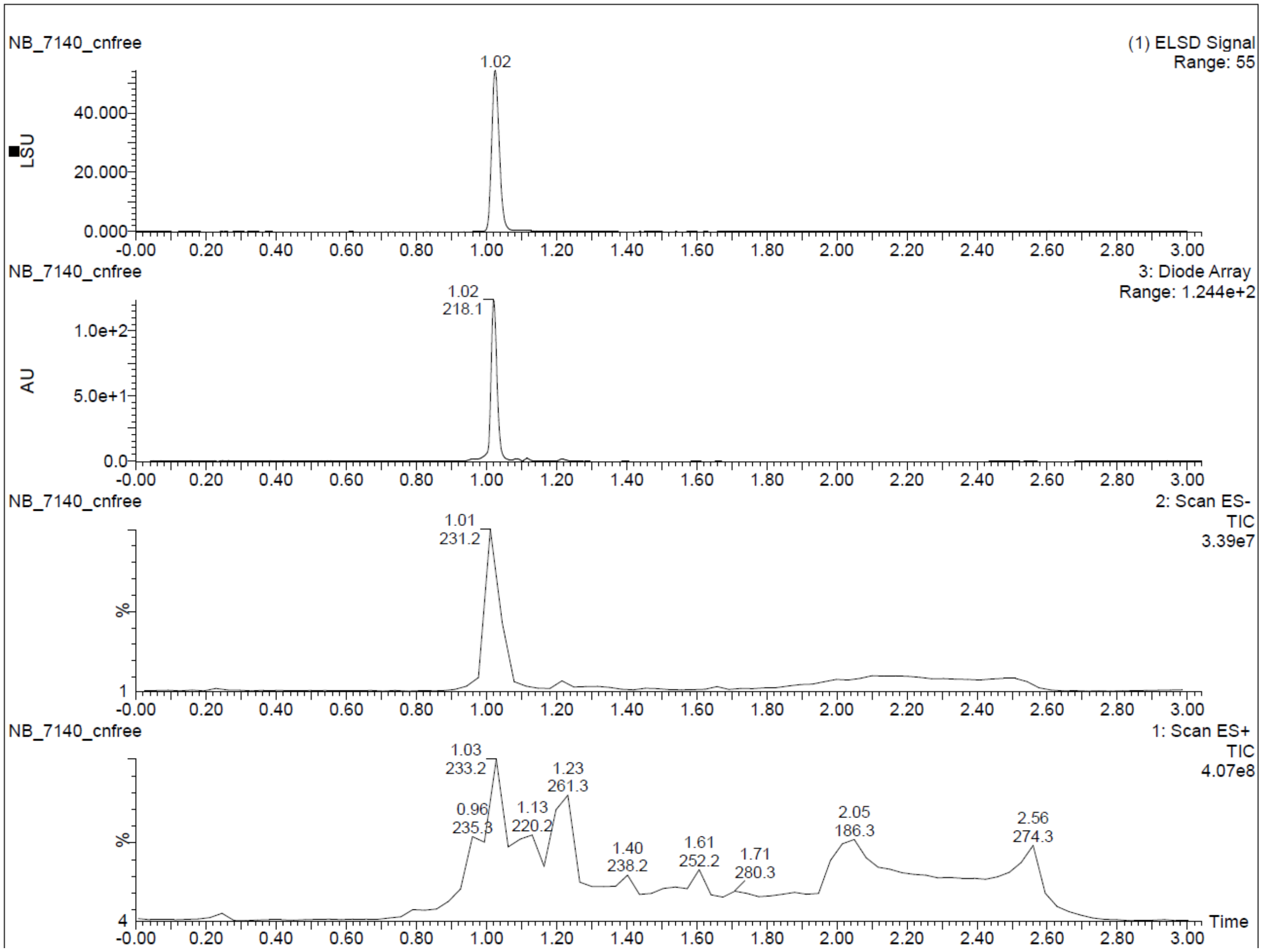
NB_7140_cnfree
(1) ELSD Signal
Range: 55
1.02
LSU
NB_7140_cnfree
3: Diode Array
Range: 1.244e+2
1.02
218.1
AU
NB_7140_cnfree
2: Scan ES-
TIC
3.39e7
1.01
231.2
%
NB_7140_cnfree
1: Scan ES+
TIC
4.07e8
1.03
233.2
1.23
261.3
0.96
235.3
1.13
220.2
1.40
238.2
1.61
252.2
1.71
280.3
2.05
186.3
2.56
274.3
%
Time

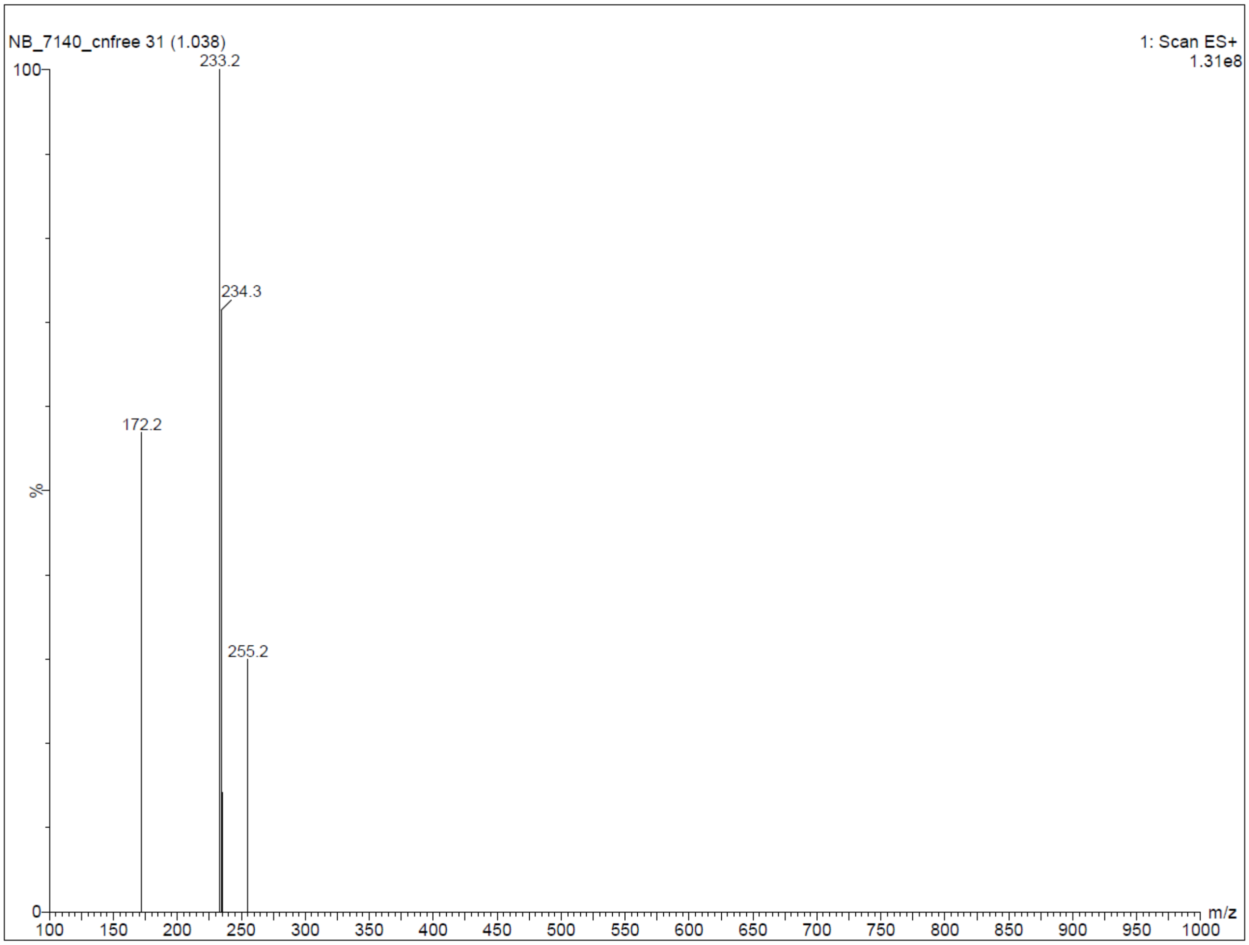

Diisopropyl 4-((2-aminoethoxy)methyl)-2,6-dimethyl-1,4-dihydropyridine-3,5-dicarboxylate (1a-CO_2i-Pr)

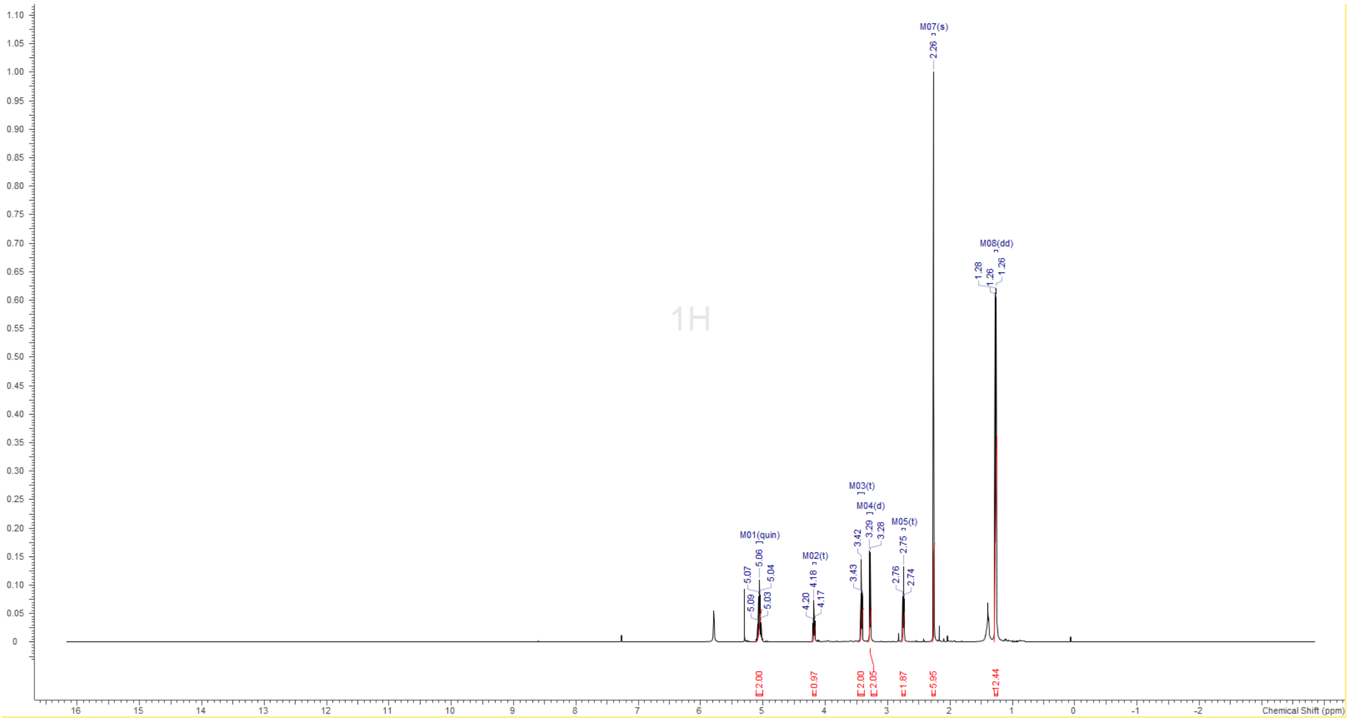

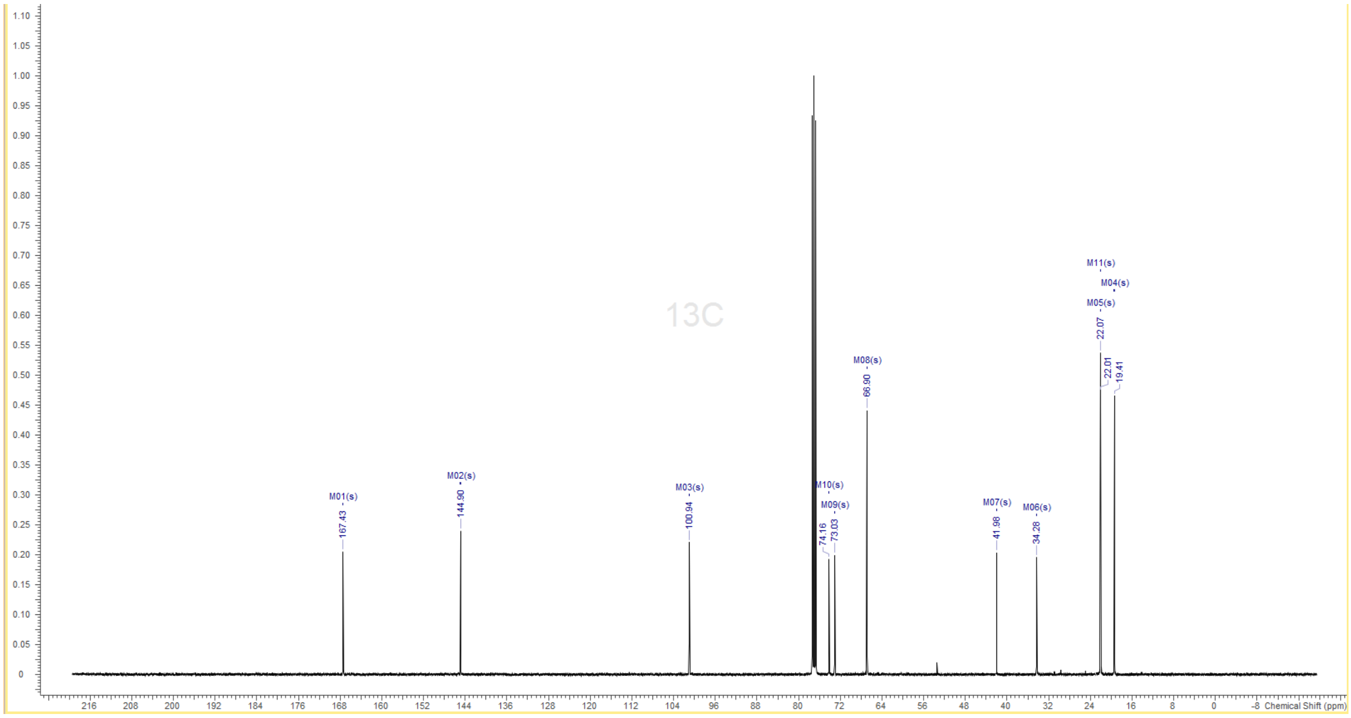
13C
M01(s)
167.43
M02(s)
144.90
M03(s)
100.94
M10(s)
74.16
M09(s)
73.03
M08(s)
66.90
M07(s)
41.98
M06(s)
34.28
M11(s)
M04(s)
M05(s)
22.07
22.01
19.41
Chemical Shift (ppm)

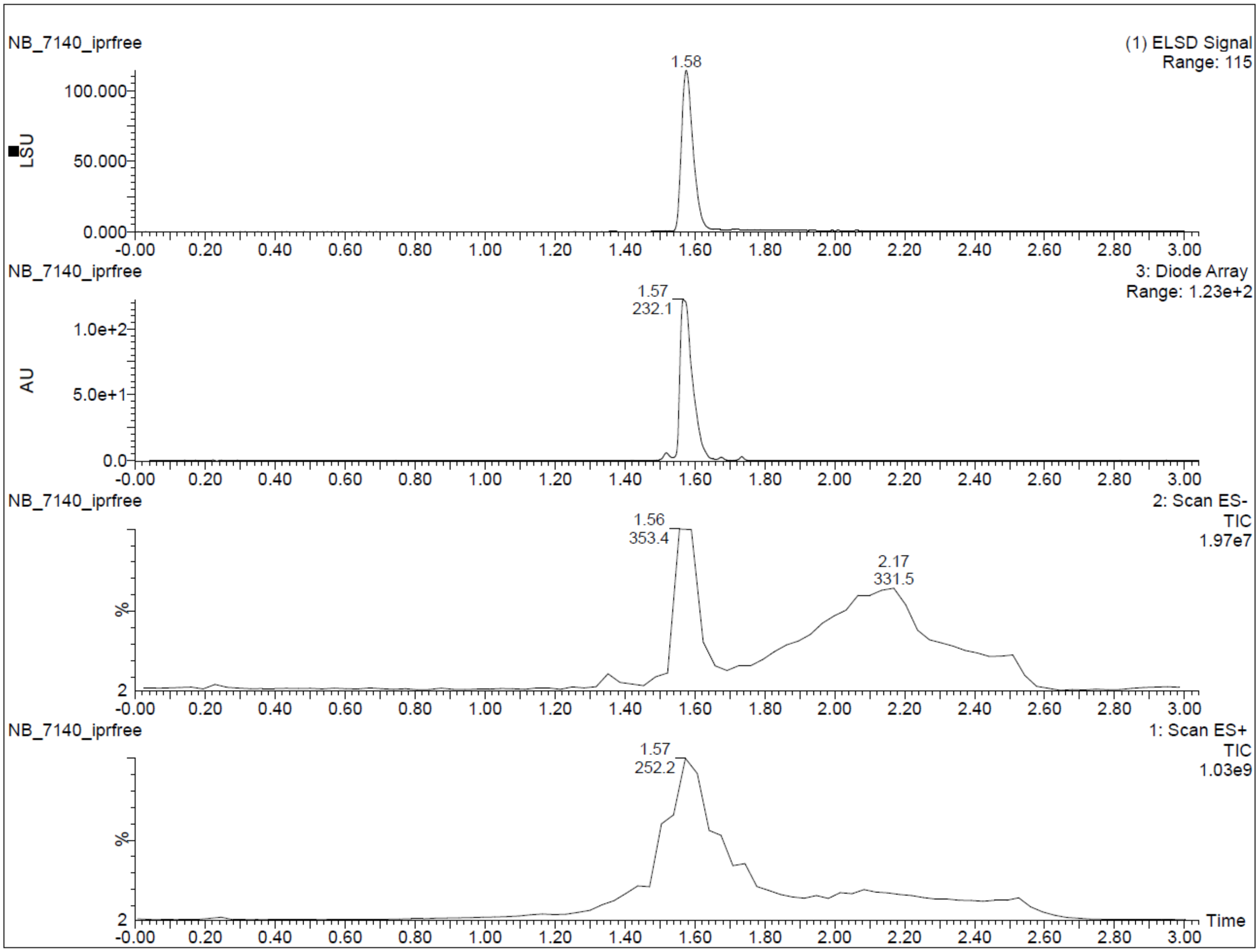
NB_7140_iprfree
(1) ELSD Signal
Range: 115
1.58
LSU
NB_7140_iprfree
3: Diode Array
Range: 1.23e+2
1.57
232.1
AU
NB_7140_iprfree
2: Scan ES-
TIC
1.97e7
1.56
353.4
2.17
331.5
%
NB_7140_iprfree
1: Scan ES+
TIC
1.03e9
1.57
252.2
%
Time

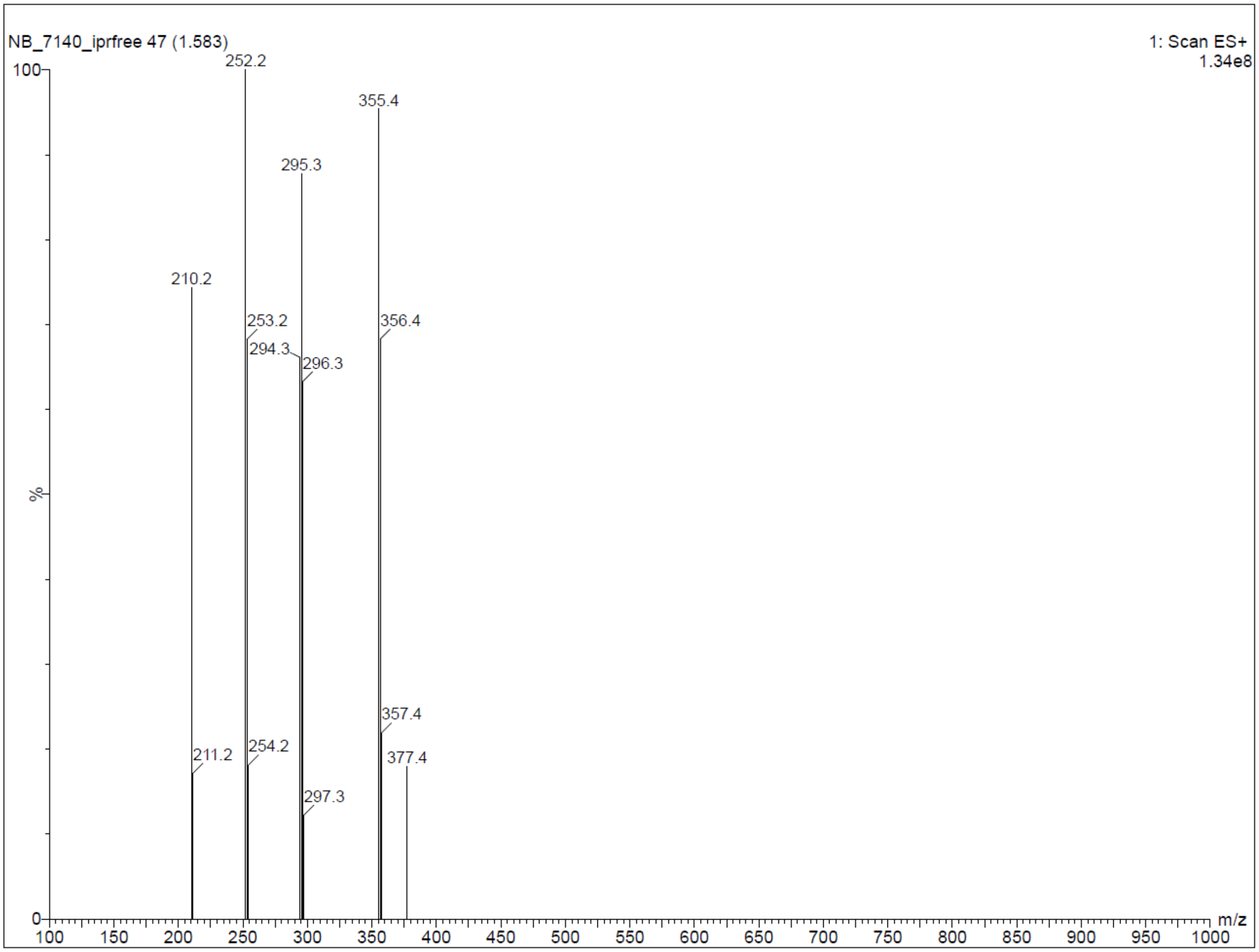

Di-tert-butyl 4-((2-aminoethoxy)methyl)-2,6-dimethyl-1,4-dihydropyridine-3,5-dicarboxylate (1a-CO_2t-Bu)

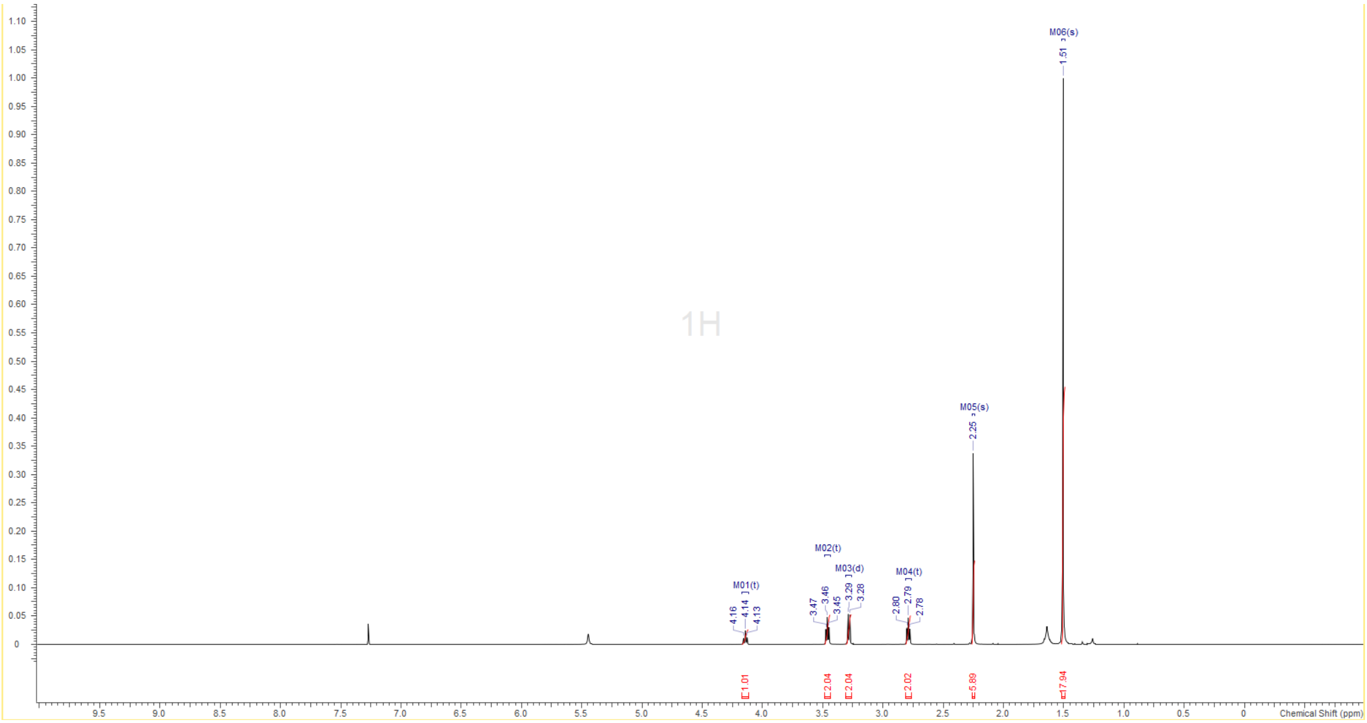

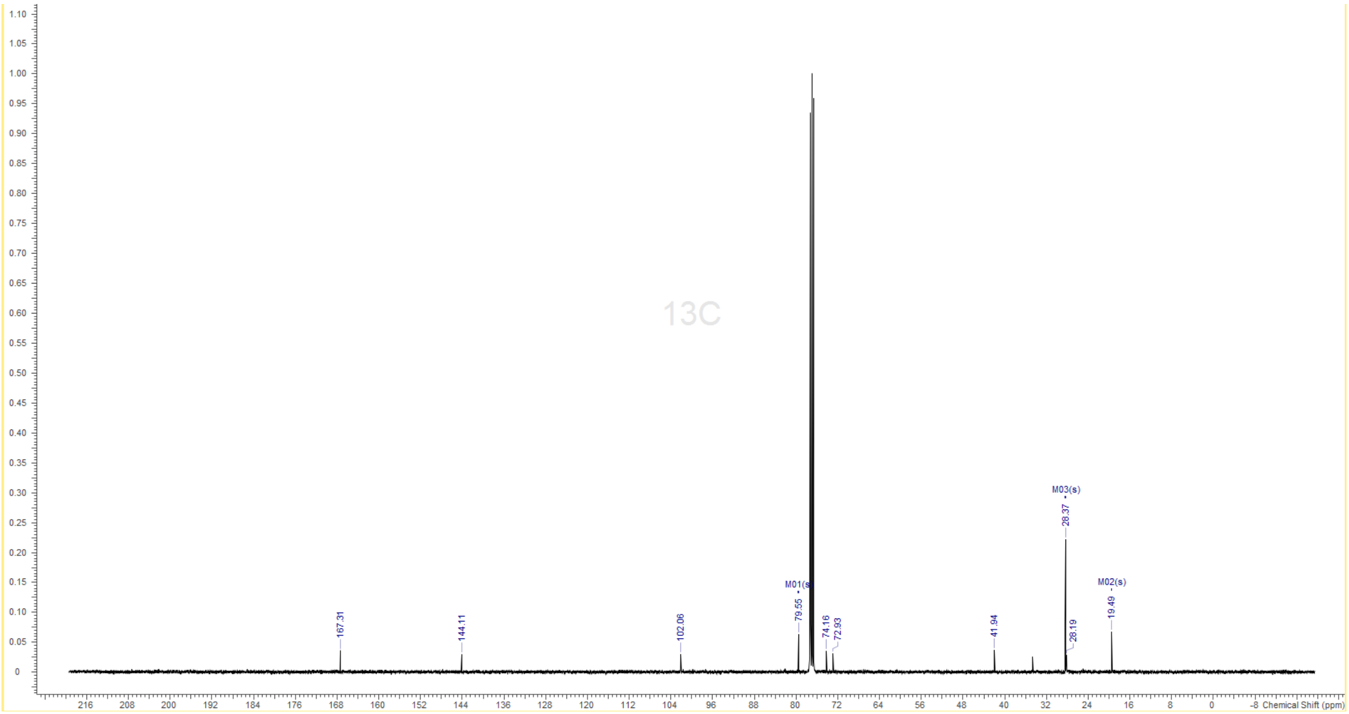
13C
M01(s)
M02(s)
M03(s)
167.31
144.11
102.06
79.55
74.16
72.93
41.94
28.37
28.19
19.49
Chemical Shift (ppm)

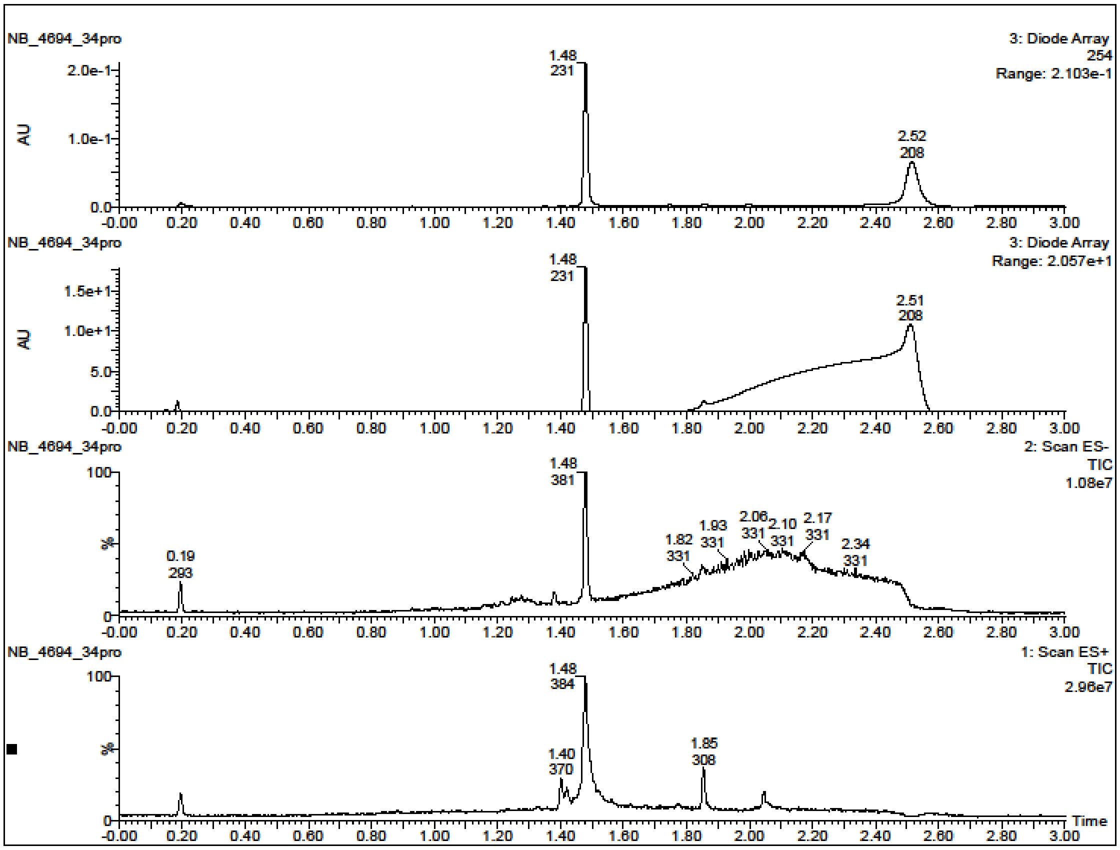
NB_4694_34pro
3: Diode Array
254
Range: 2.103e-1
3: Diode Array
Range: 2.057e+1
2: Scan ES-
TIC
1.08e7
1: Scan ES+
TIC
2.96e7
AU
%
Time

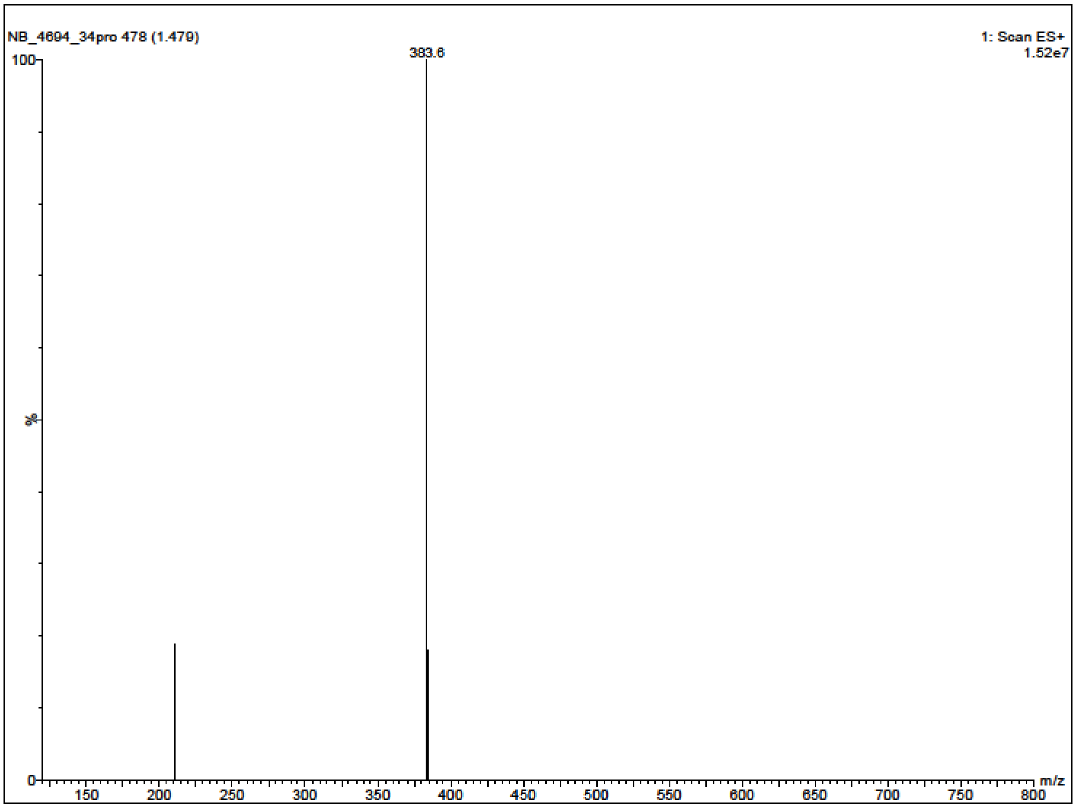
NB_4694_34pro 478 (1.479)
1: Scan ES+
1.52e7
383.6
%
m/z

1-((Dibenzylamino)methyl)cyclobutan-1-ol

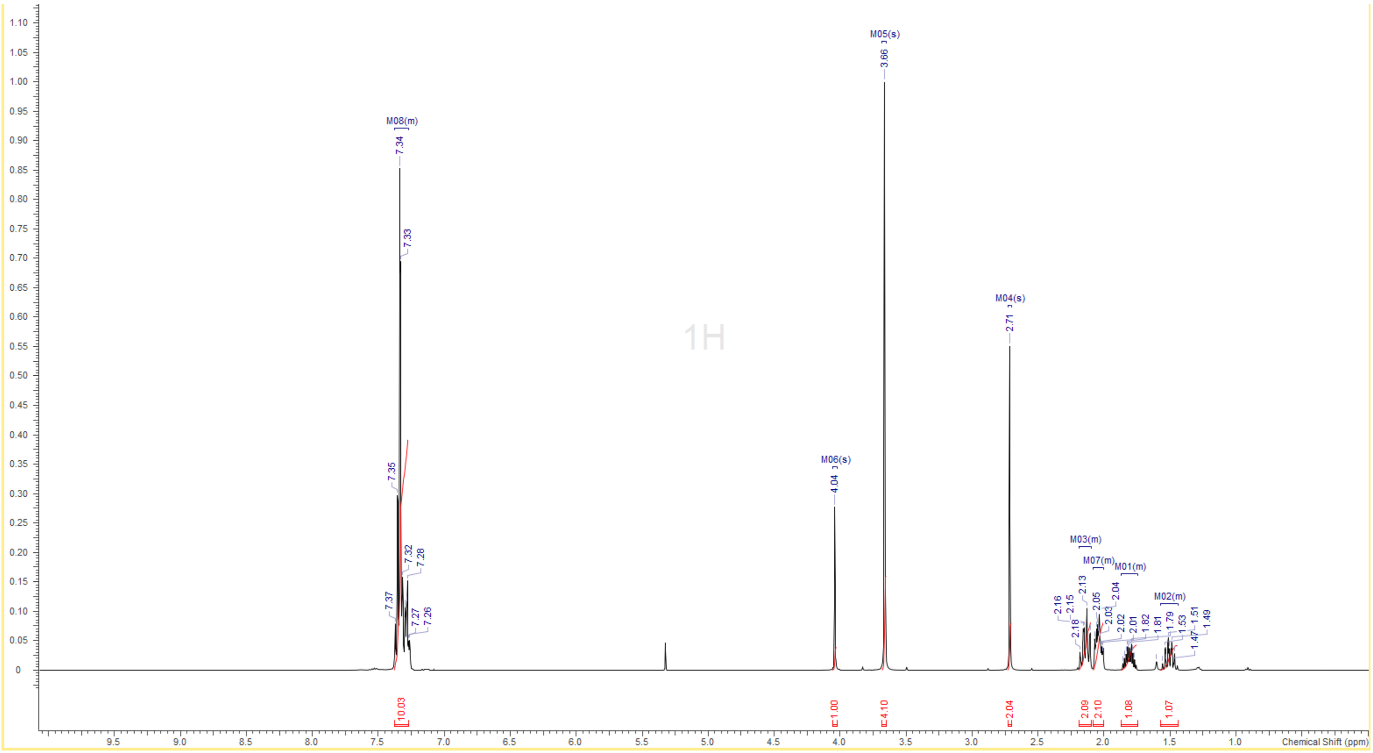

***N,N*-Dibenzyl-1-(1-(2,2-dimethoxyethoxy)cyclobutyl)methanamine**

(1:1 ratio with 1-((dibenzylamino)methyl)cyclobutan-1-ol)

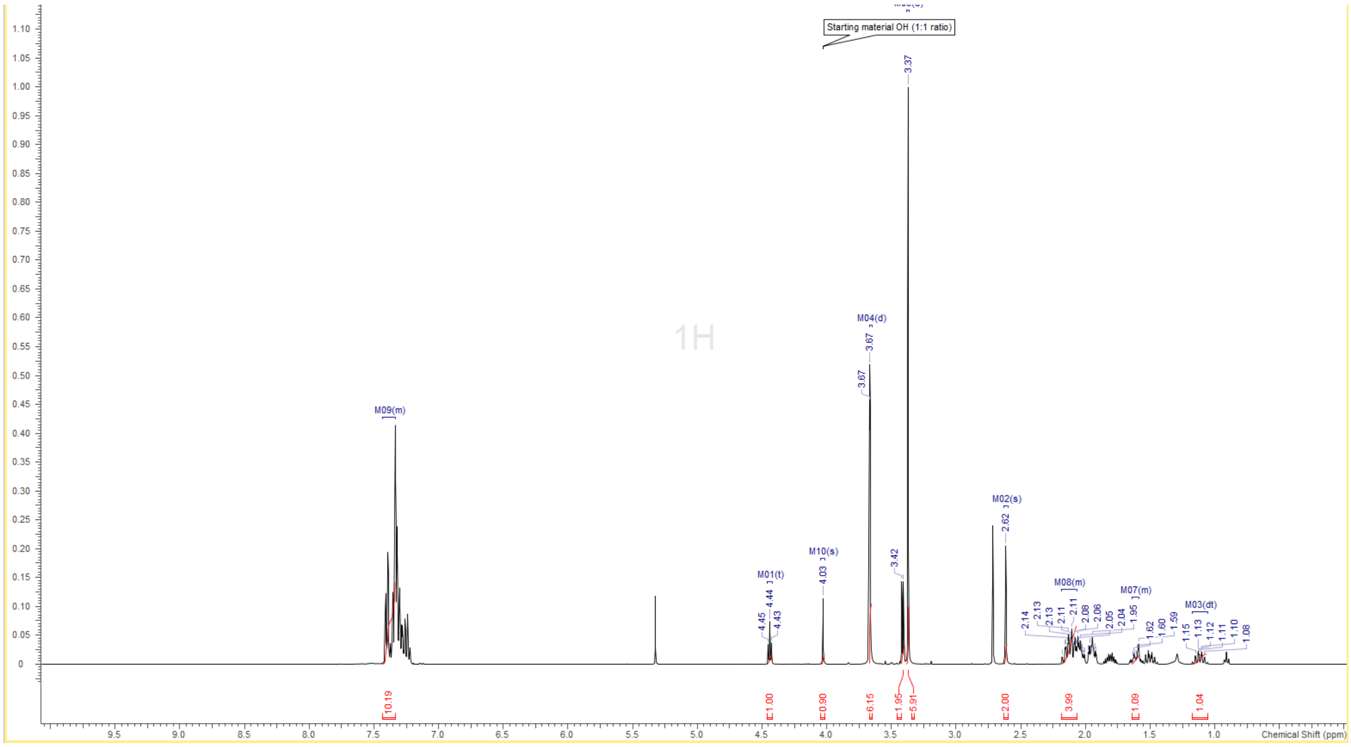

Diisopropyl 4-((1-((dibenzylamino)methyl)cyclobutoxy)methyl)-2,6-dimethyl-1,4-dihydropyridine-3,5-dicarboxylate

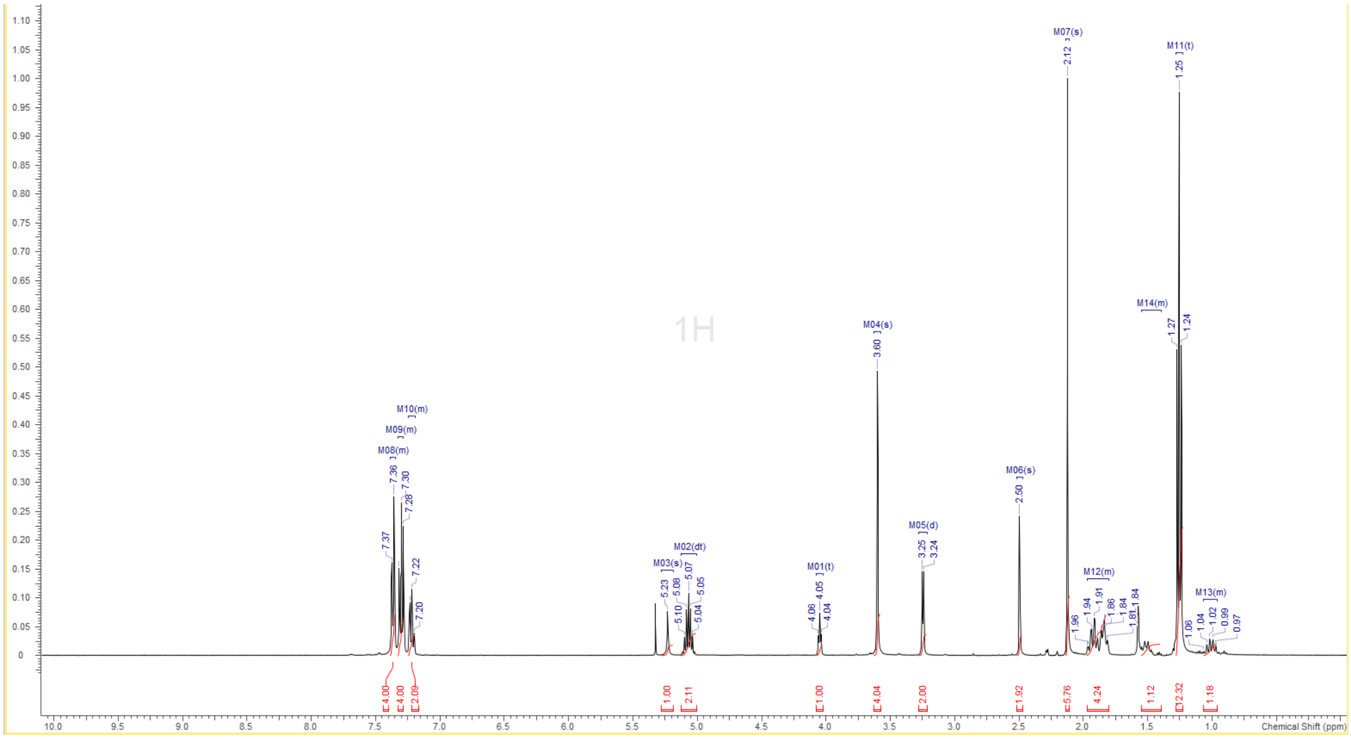

Diisopropyl 4-((1-(aminomethyl)cyclobutoxy)methyl)-2,6-dimethyl-1,4-dihydropyridine-3,5-dicarboxylate (1b)

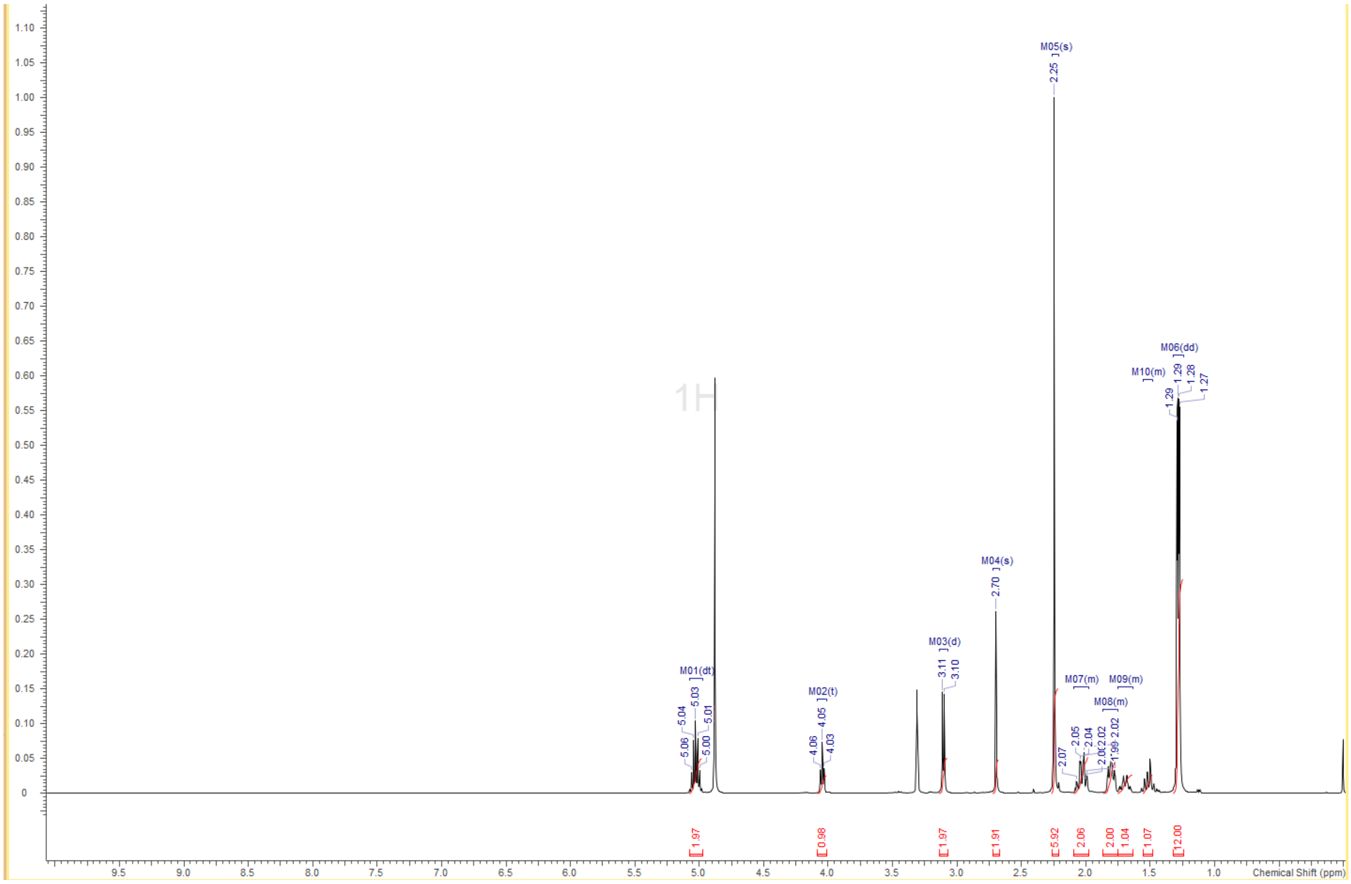

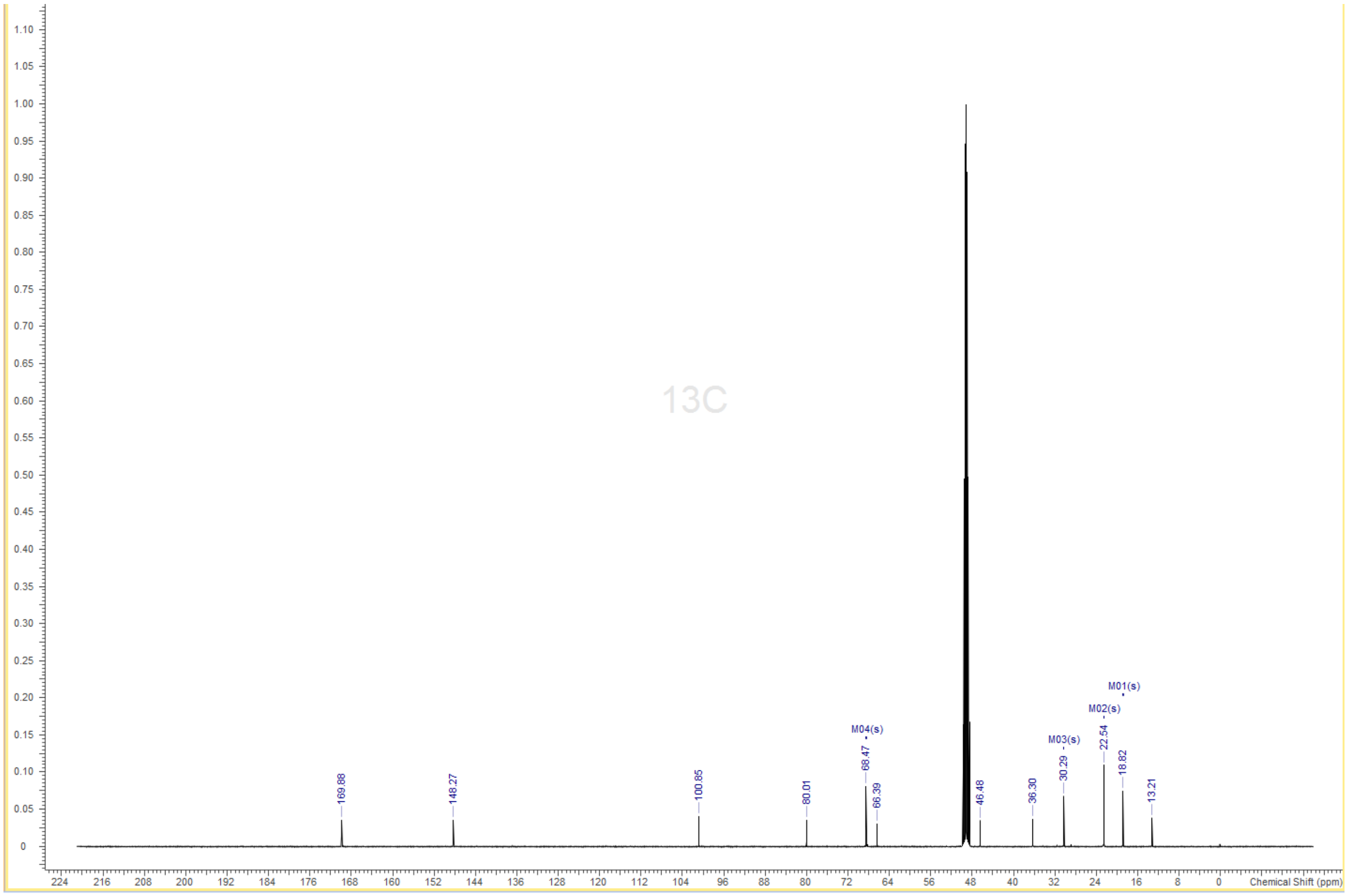
13C
169.88
148.27
100.85
80.01
68.47
66.39
46.48
36.30
30.29
22.54
18.82
13.21
M01(s)
M02(s)
M03(s)
M04(s)
Chemical Shift (ppm)

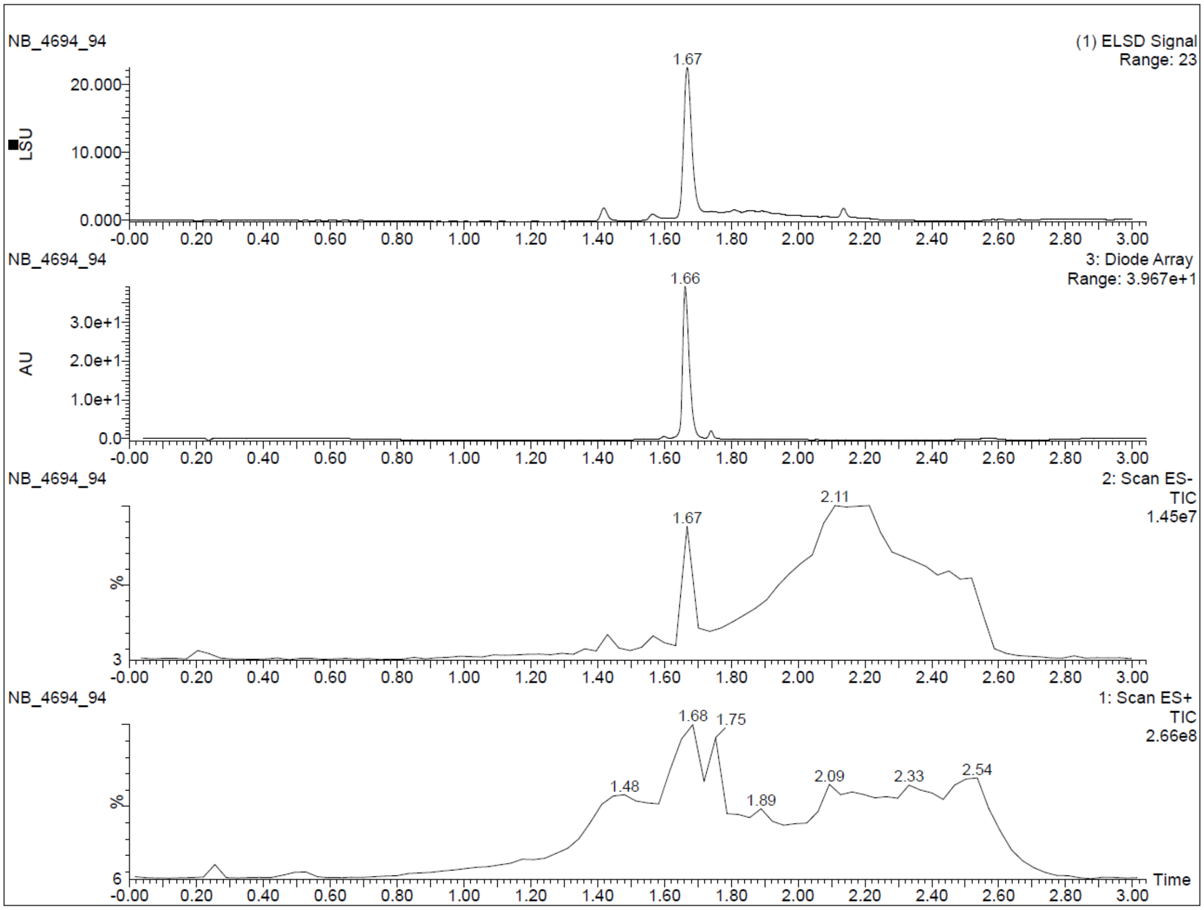
NB_4694_94
(1) ELSD Signal
Range: 23
1.67
LSU
NB_4694_94
3: Diode Array
Range: 3.967e+1
1.66
AU
NB_4694_94
2: Scan ES-
TIC
1.45e7
2.11
1.67
%
NB_4694_94
1: Scan ES+
TIC
2.66e8
1.68
1.75
1.48
1.89
2.09
2.33
2.54
%
Time

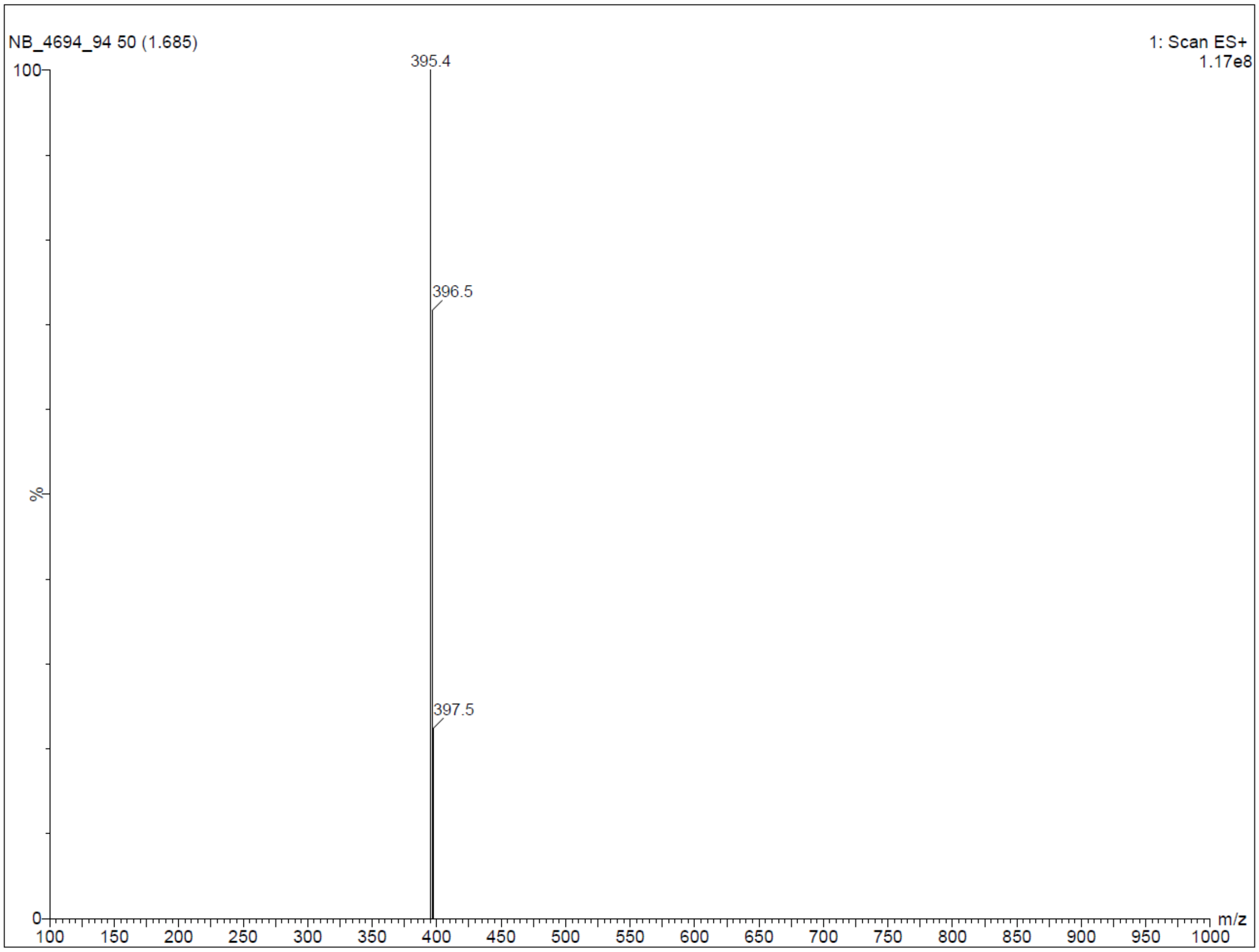

(S)-N,N-Dibenzyl-1-(2,2-dimethoxyethoxy)propan-2-amine

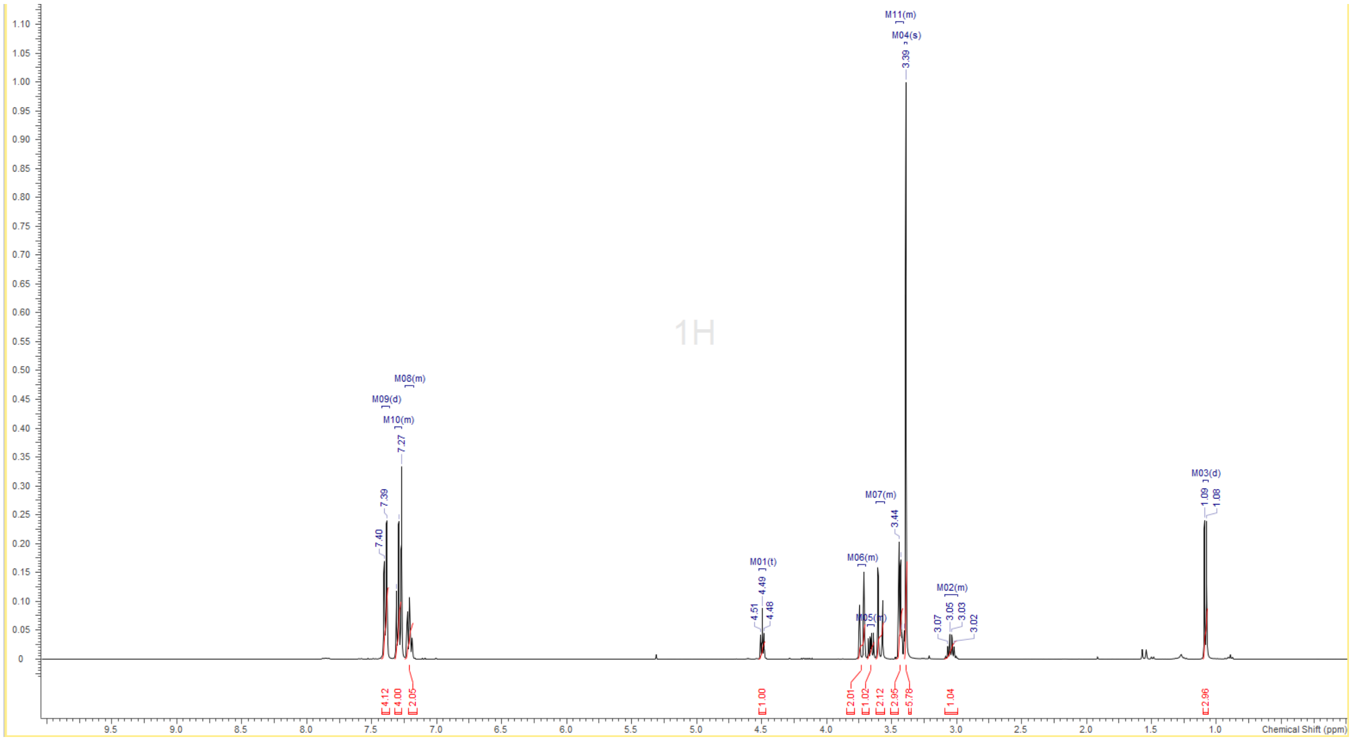

(S)-2-(2-(Dibenzylamino)propoxy)acetaldehyde

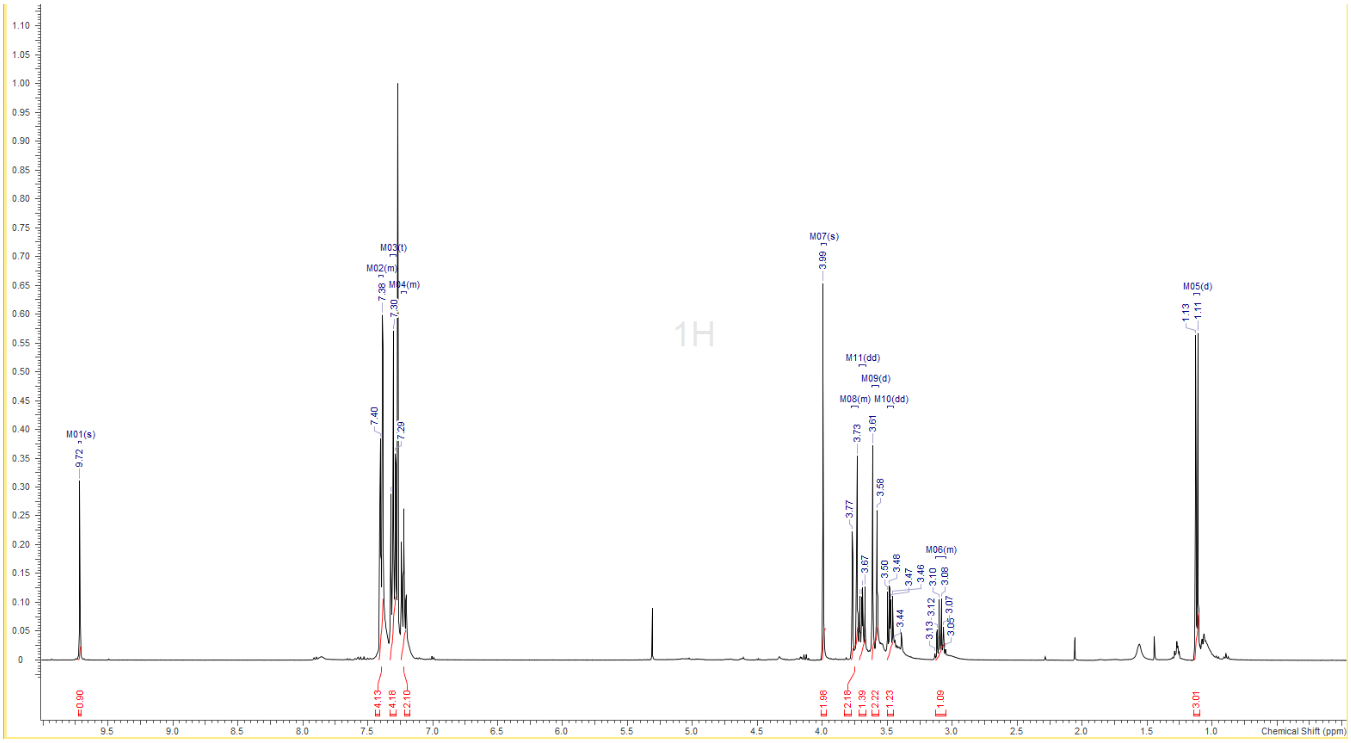

Diisopropyl (S)-4-((2-(dibenzylamino)propoxy)methyl)-2,6-dimethyl-1,4-dihydropyridine-3,5-dicarboxylate

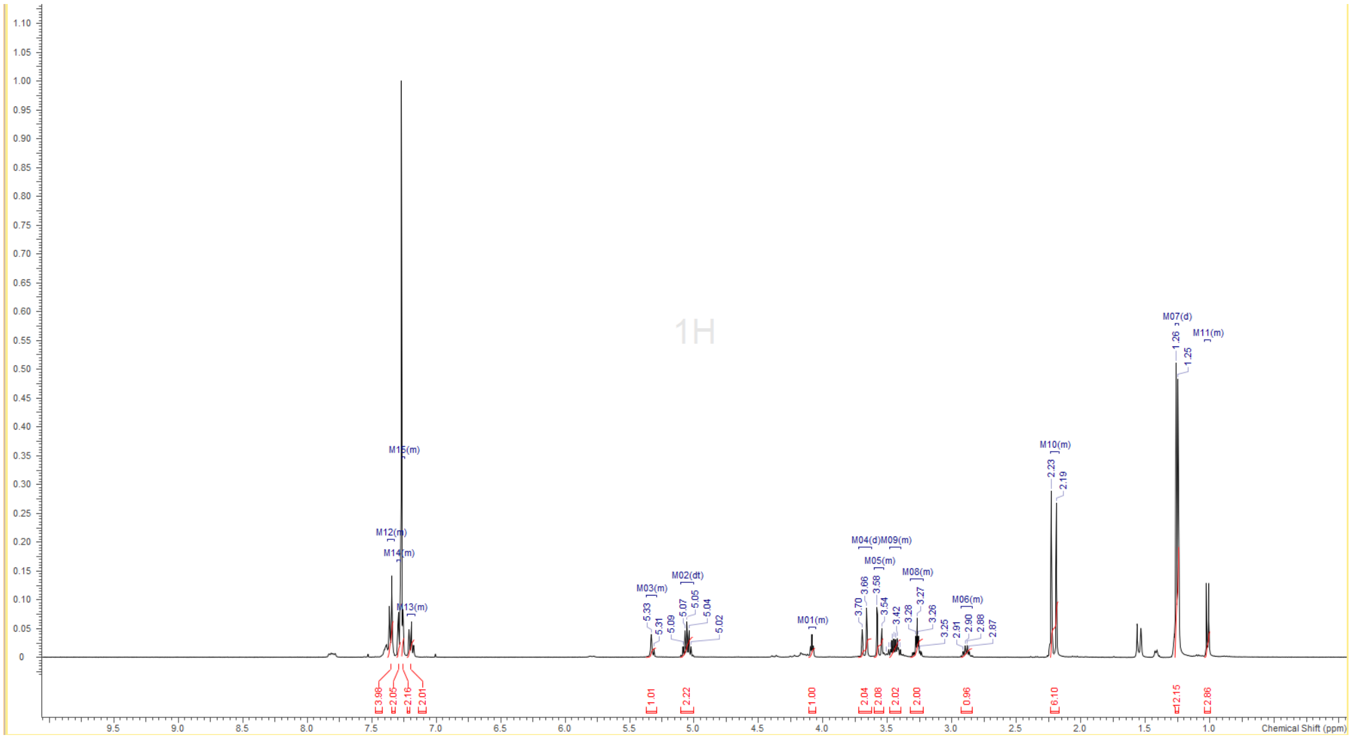

Diisopropyl (S)-4-((2-aminopropoxy)methyl)-2,6-dimethyl-1,4-dihydropyridine-3,5-dicarboxylate (1c)

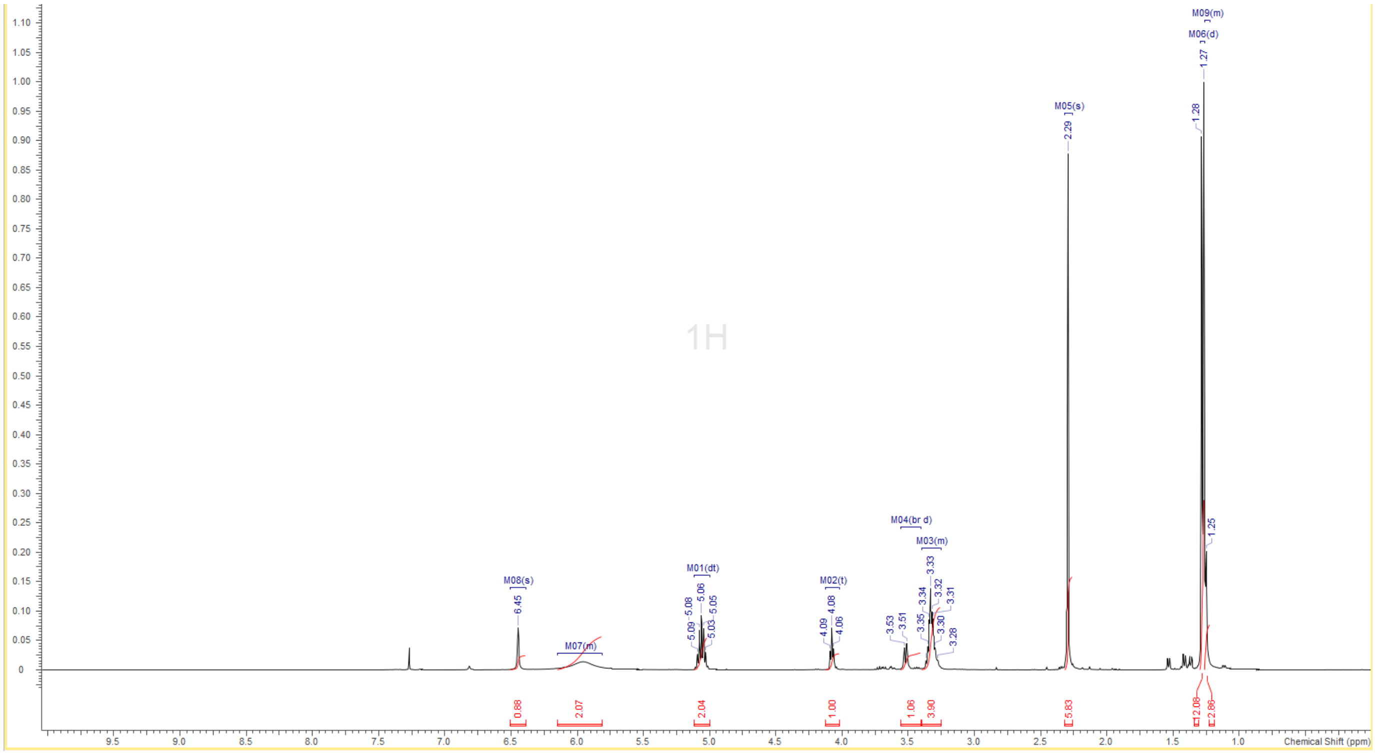
1H
M08(s)
6.45
M07(m)
M01(dt)
5.09
5.08
5.06
5.05
5.03
M02(t)
4.09
4.08
4.06
M04(br d)
3.53
3.51
M03(m)
3.35
3.34
3.33
3.32
3.31
3.30
3.28
M05(s)
2.29
M09(m)
M06(d)
1.28
1.27
1.25
0.88
2.07
2.04
1.00
1.06
3.90
5.83
12.08
2.86
Chemical Shift (ppm)

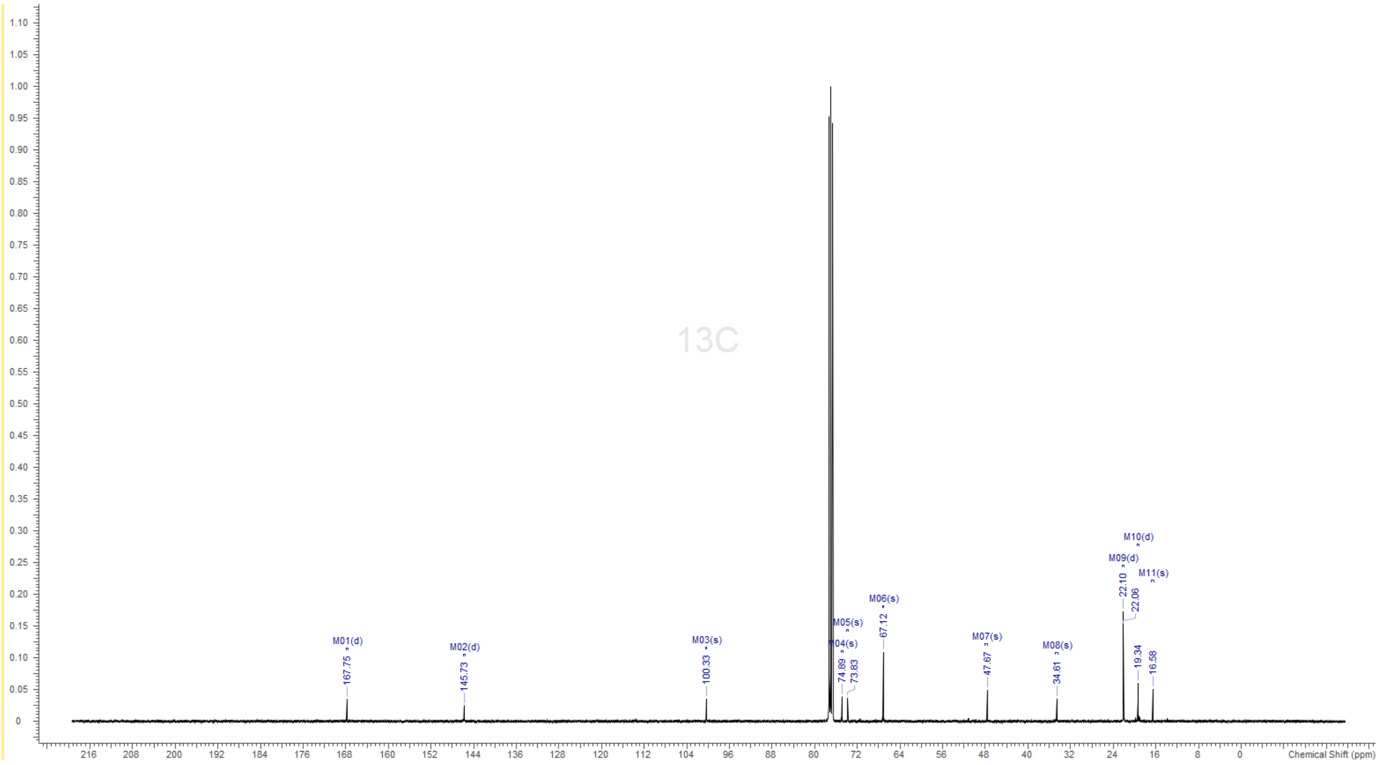
13C
M01(d)
167.75
M02(d)
145.73
M03(s)
100.33
M05(s)
M04(s)
74.89
73.83
M06(s)
67.12
M07(s)
47.67
M08(s)
34.61
M10(d)
M09(d)
22.10
22.06
M11(s)
19.34
16.58
Chemical Shift (ppm)

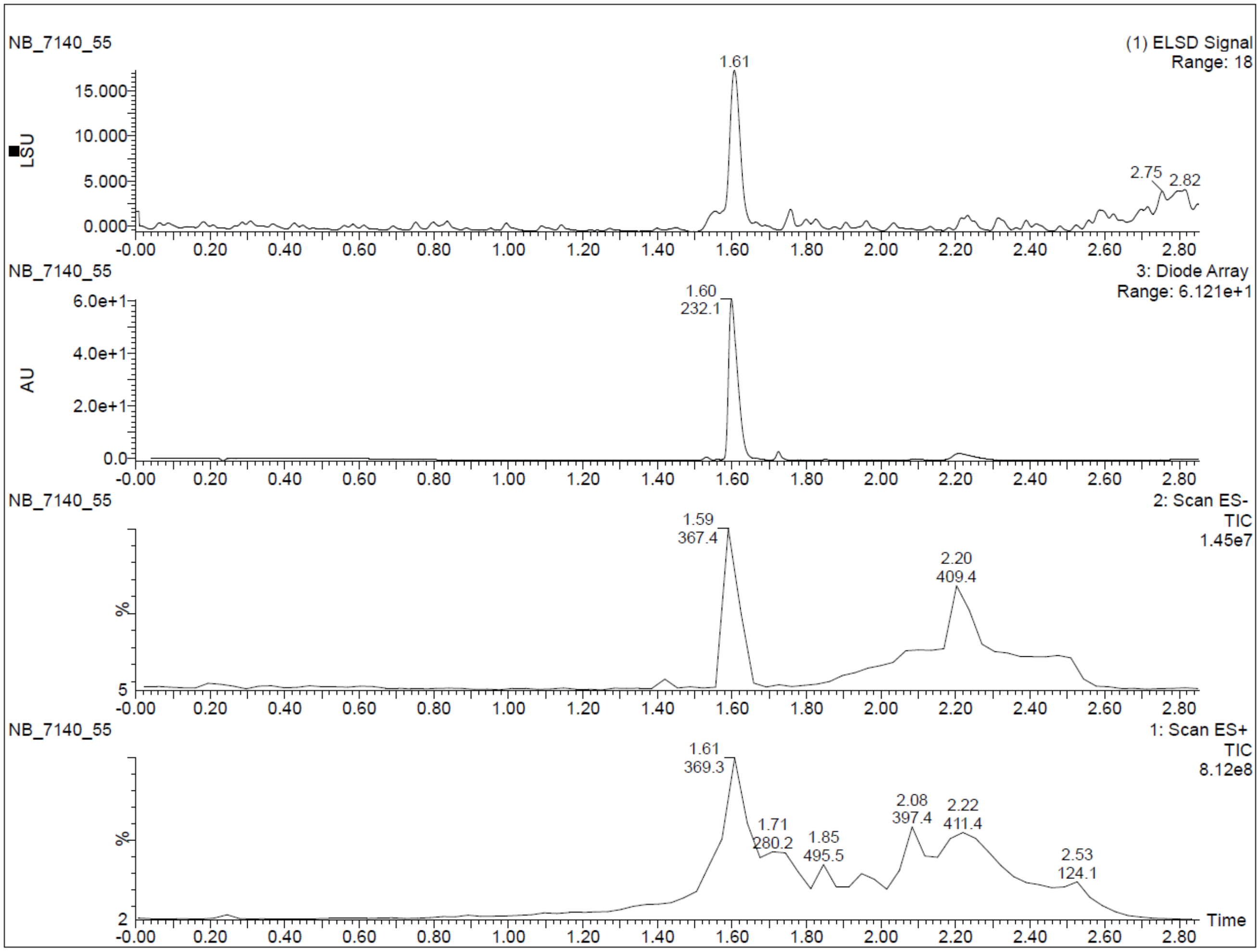

NB_7140_55
(1) ELSD Signal
Range: 18
LSU
1.61
2.75
2.82
NB_7140_55
3: Diode Array
Range: 6.121e+1
AU
1.60
232.1
NB_7140_55
2: Scan ES-
TIC
1.45e7
1.59
367.4
2.20
409.4
NB_7140_55
1: Scan ES+
TIC
8.12e8
1.61
369.3
1.71
280.2
1.85
495.5
2.08
397.4
2.22
411.4
2.53
124.1
Time

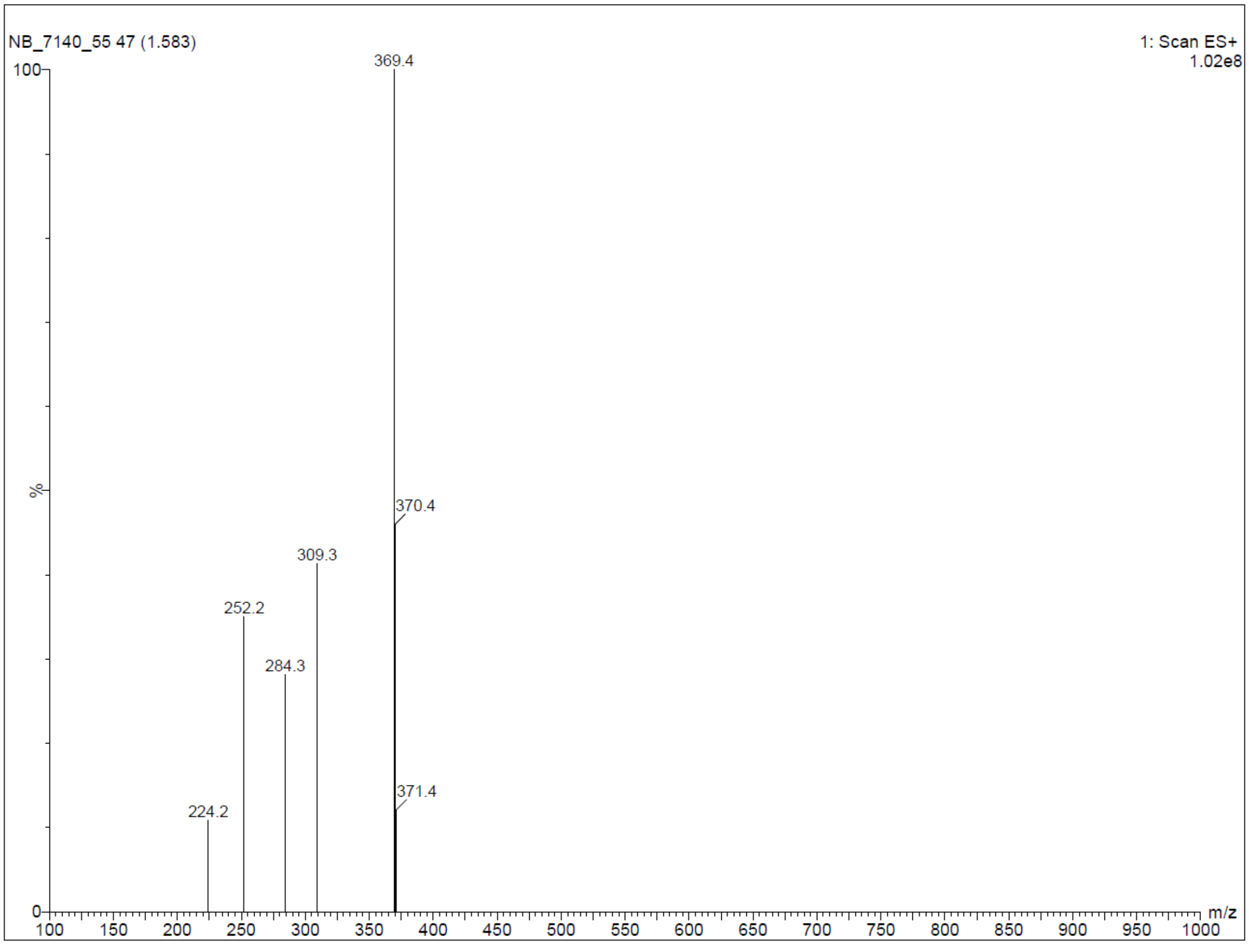

(3R,4R)-4-(Dibenzylamino)tetrahydrofuran-3-ol

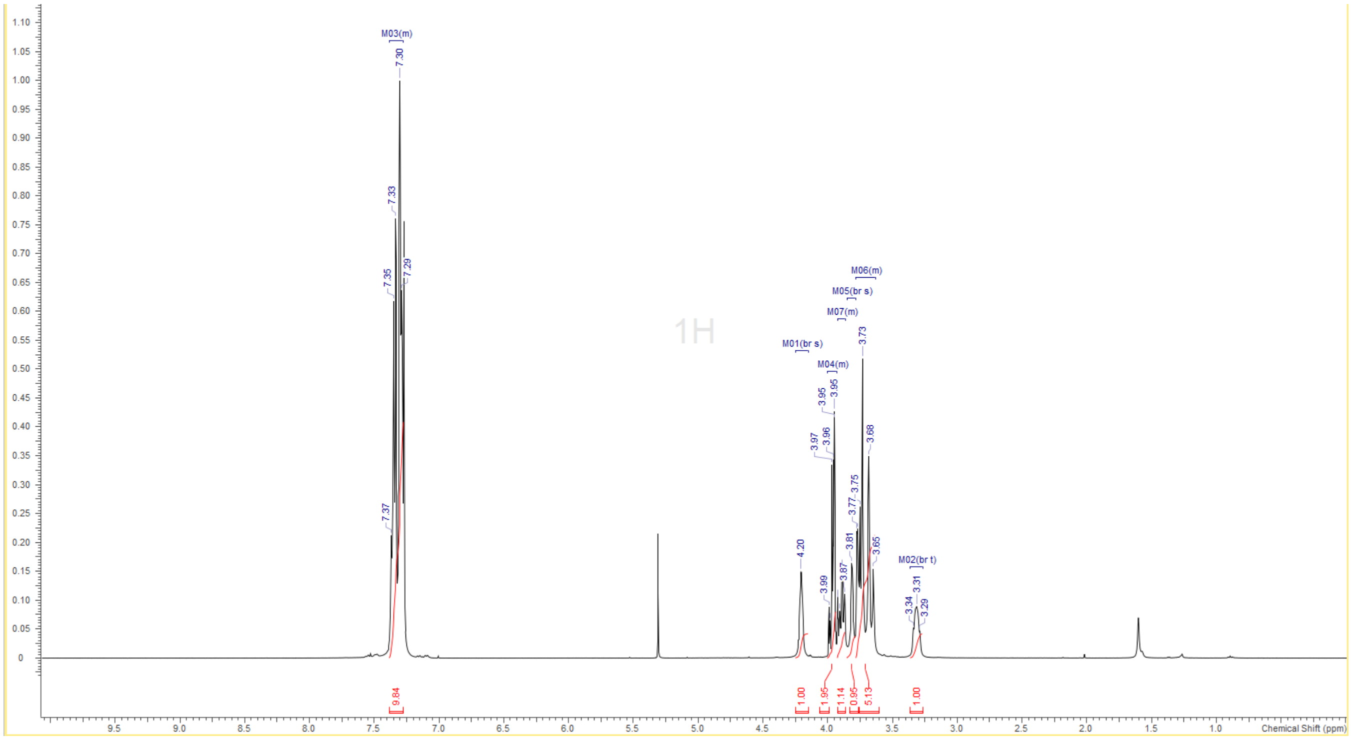

(3R,4R)-N,N-Dibenzyl-4-(2,2-dimethoxyethoxy)tetrahydrofuran-3-amine

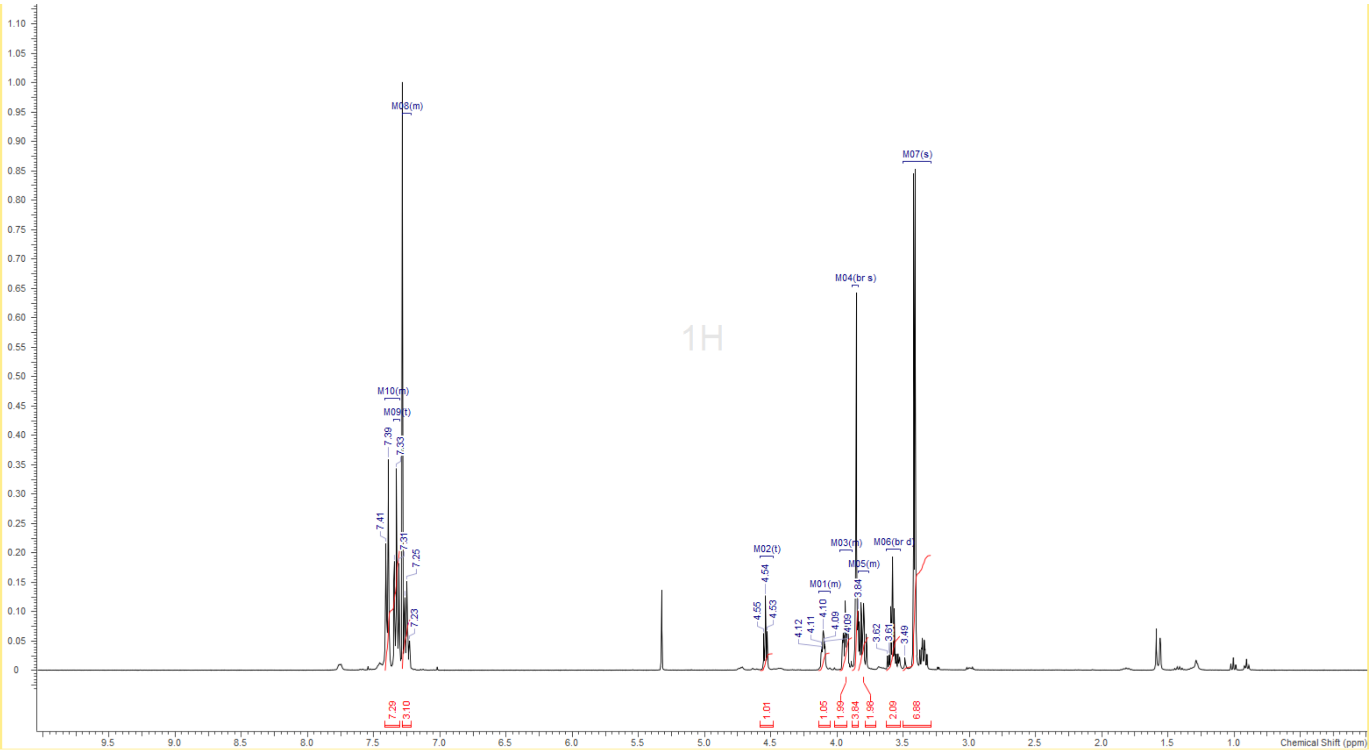

Diisopropyl 4-((((3R,4R)-4-(dibenzylamino)tetrahydrofuran-3-yl)oxy)methyl)-2,6-dimethyl-1,4-dihydropyridine-3,5-dicarboxylate

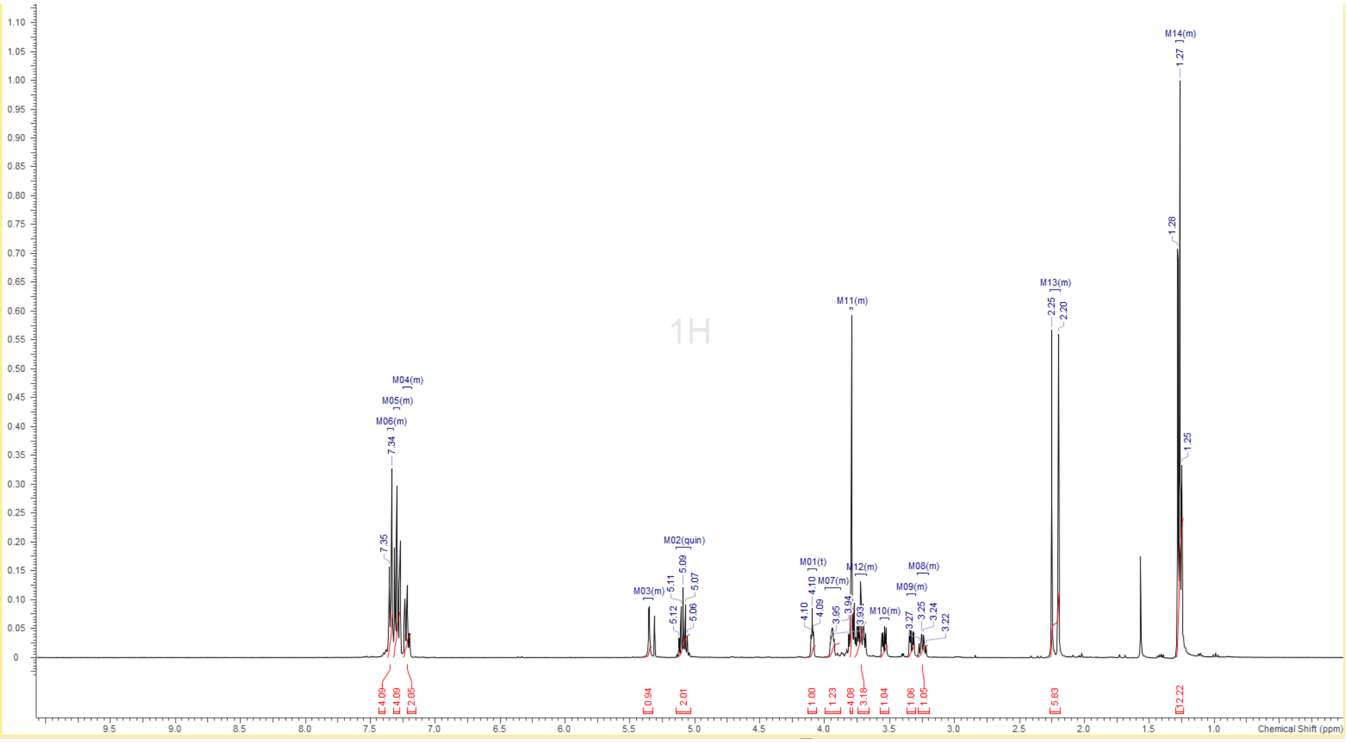

Diisopropyl 4-((((3R,4R)-4-aminotetrahydrofuran-3-yl)oxy)methyl)-2,6-dimethyl-1,4-dihydropyridine-3,5-dicarboxylate (1f)

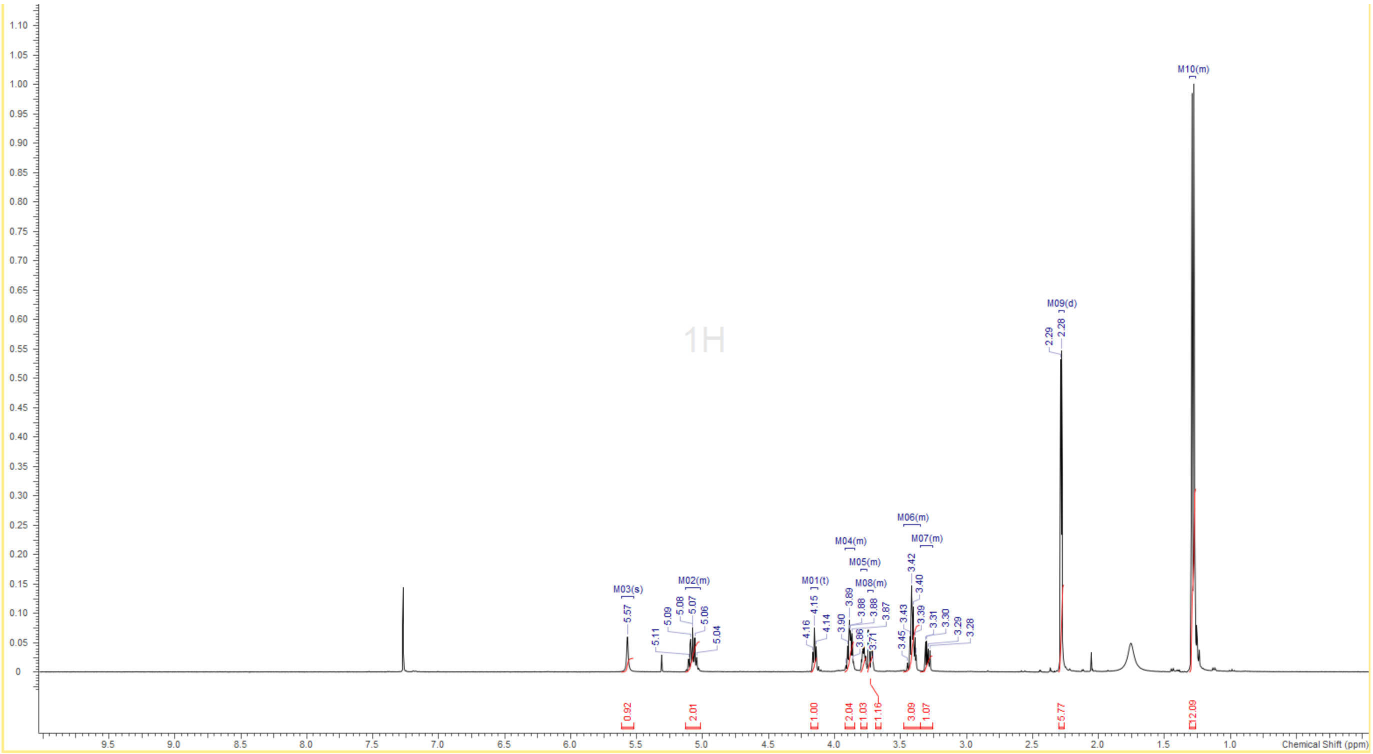
1H
Chemical Shift (ppm)

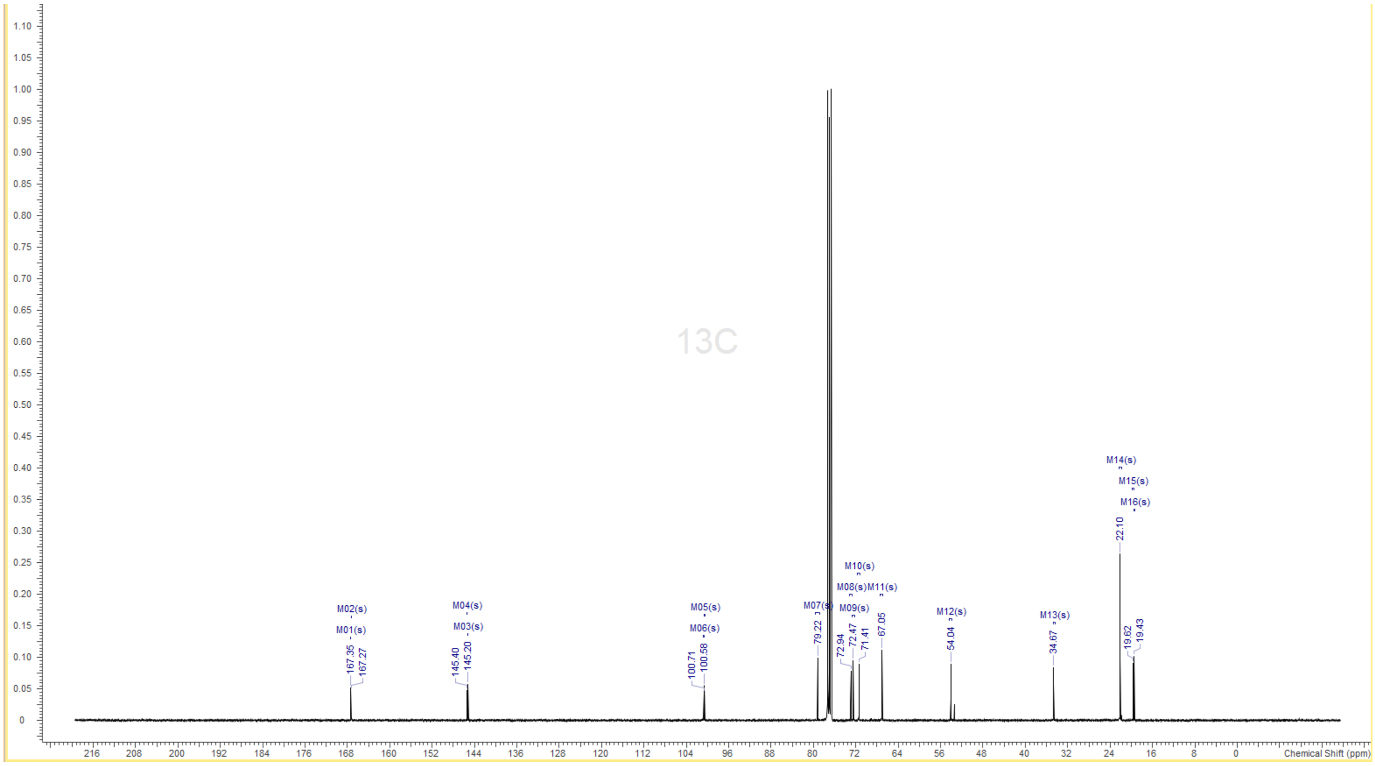
13C
Chemical Shift (ppm)

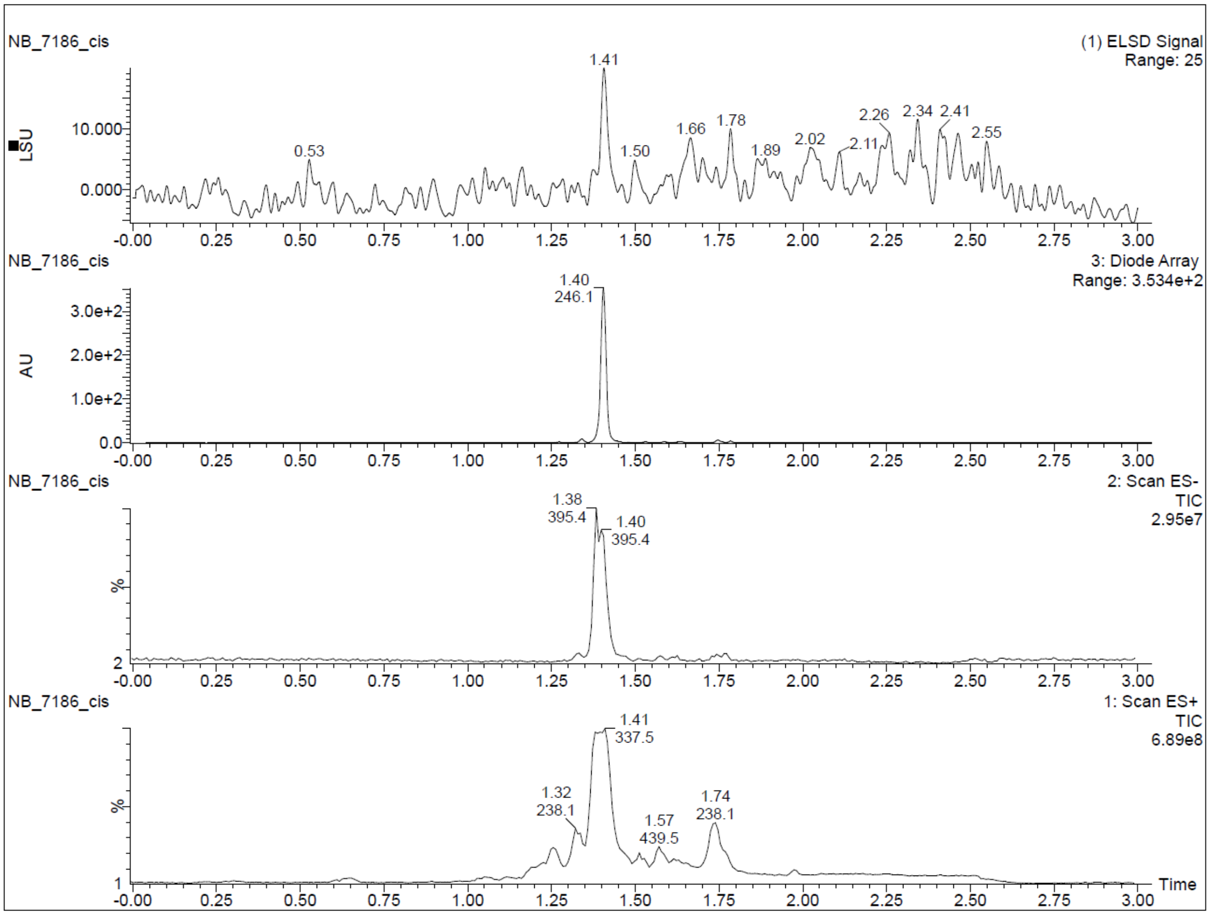
NB_7186_cis
(1) ELSD Signal
Range: 25
LSU
10.000
0.000
1.41
0.53
1.50
1.66
1.78
1.89
2.02
2.11
2.26
2.34
2.41
2.55
NB_7186_cis
3: Diode Array
Range: 3.534e+2
AU
3.0e+2
2.0e+2
1.0e+2
0.0
1.40
246.1
NB_7186_cis
2: Scan ES-
TIC
2.95e7
%
1.38
395.4
1.40
395.4
NB_7186_cis
1: Scan ES+
TIC
6.89e8
%
1.41
337.5
1.32
238.1
1.57
439.5
1.74
238.1
Time
-0.00
0.25
0.50
0.75
1.00
1.25
1.50
1.75
2.00
2.25
2.50
2.75
3.00

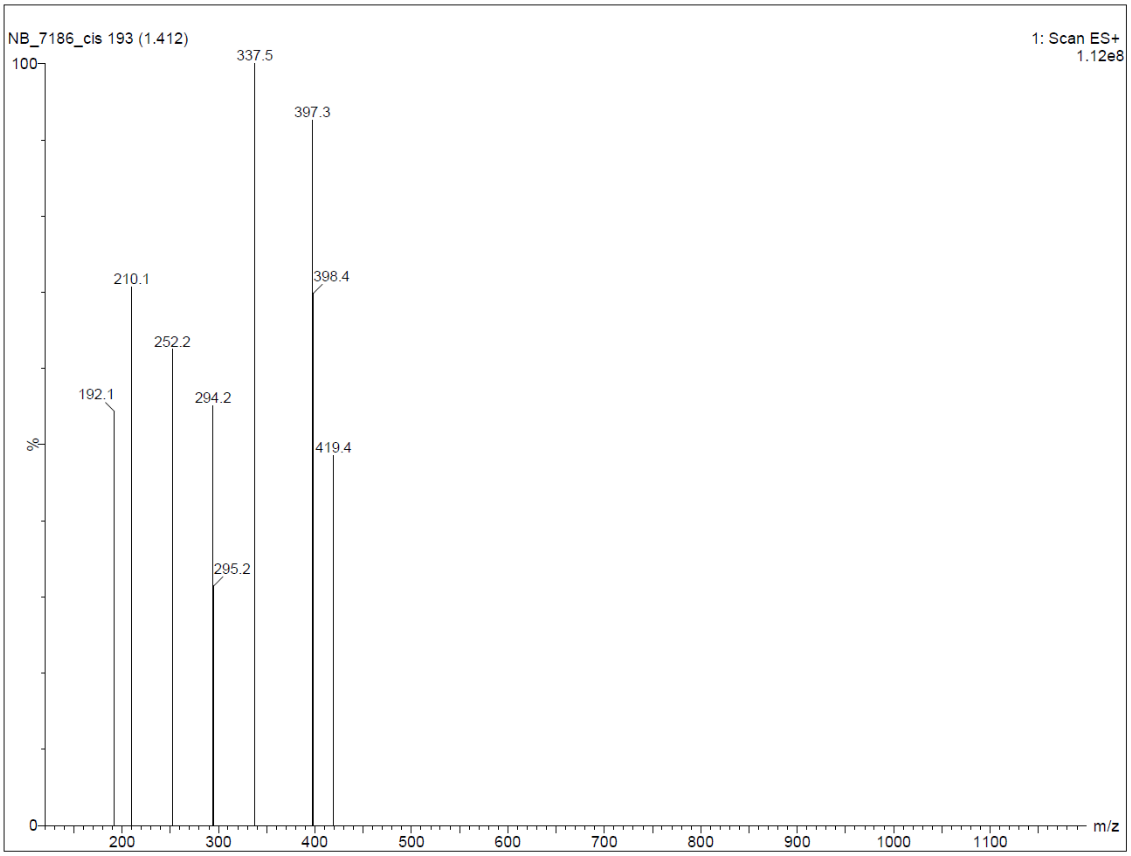
NB_7186_cis 193 (1.412)
1: Scan ES+
1.12e8
100
%
0
337.5
397.3
210.1
398.4
252.2
192.1
294.2
419.4
295.2
200
300
400
500
600
700
800
900
1000
1100
m/z

(R)-1-(Dibenzylamino)propan-2-ol

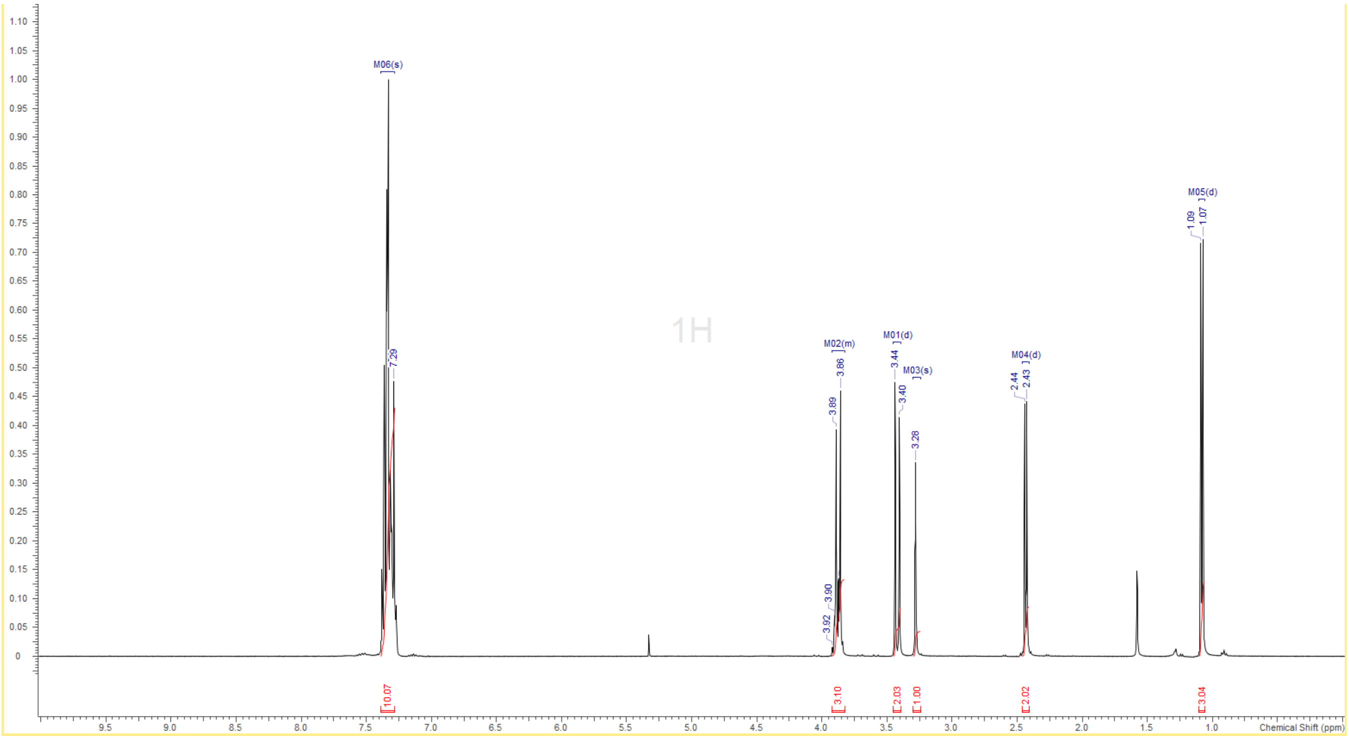

(R)-N,N-Dibenzyl-2-(2,2-dimethoxyethoxy)propan-1-amine

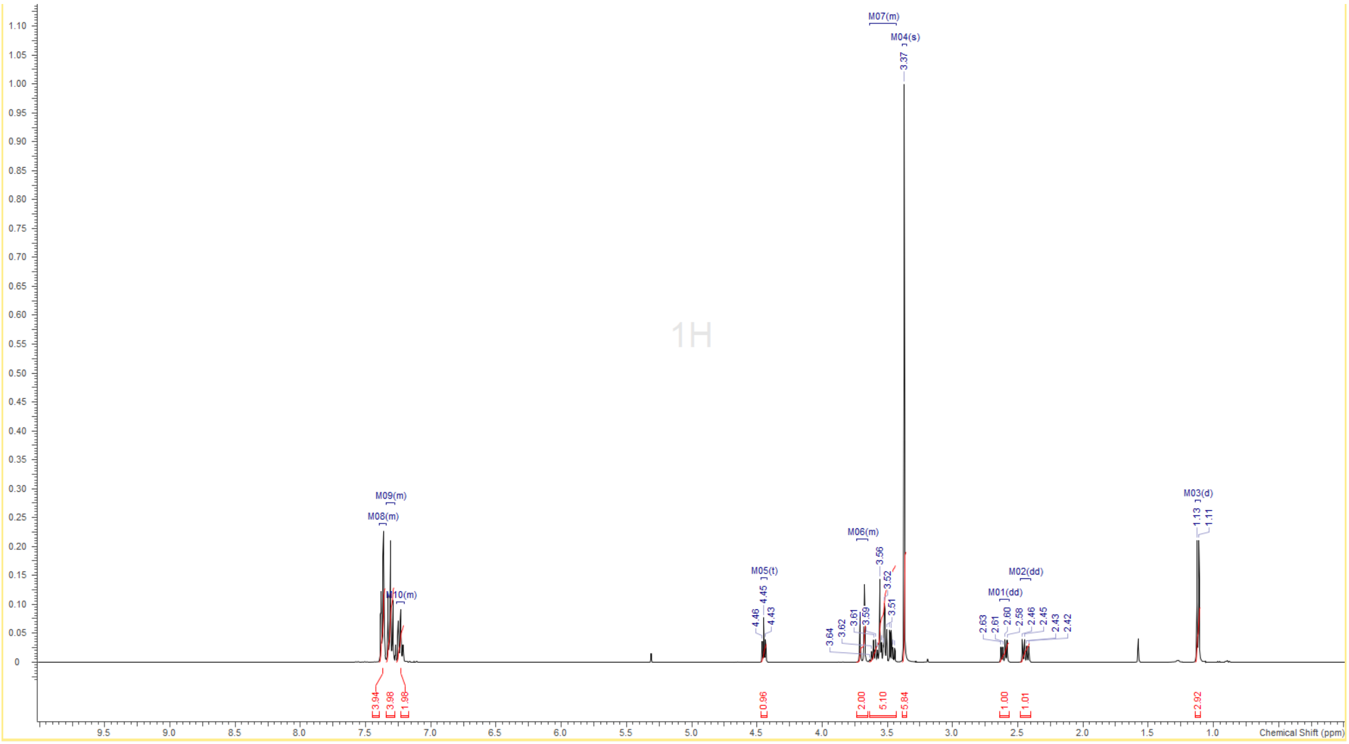

(R)-2-((1-(Dibenzylamino)propan-2-yl)oxy)acetaldehyde

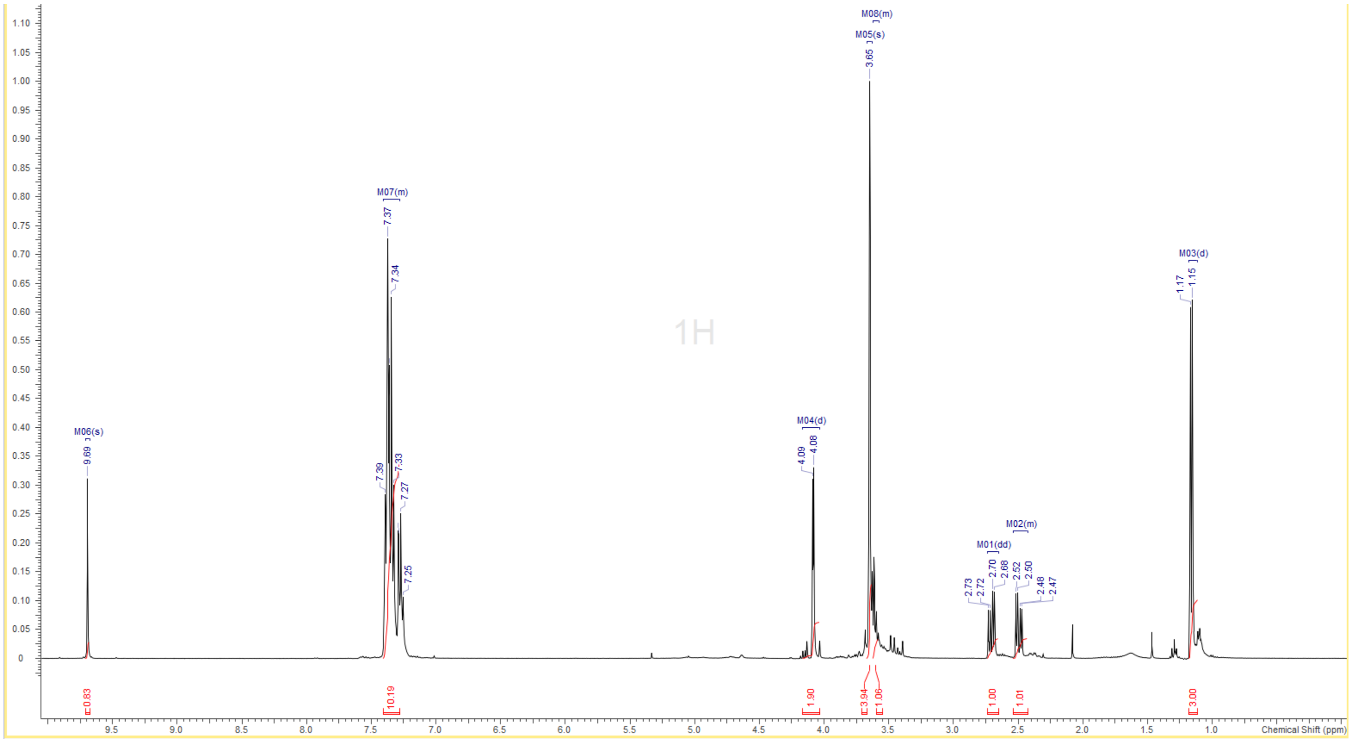

Diisopropyl (R)-4-(((1-(dibenzylamino)propan-2-yl)oxy)methyl)-2,6-dimethyl-1,4-dihydropyridine-3,5-dicarboxylate

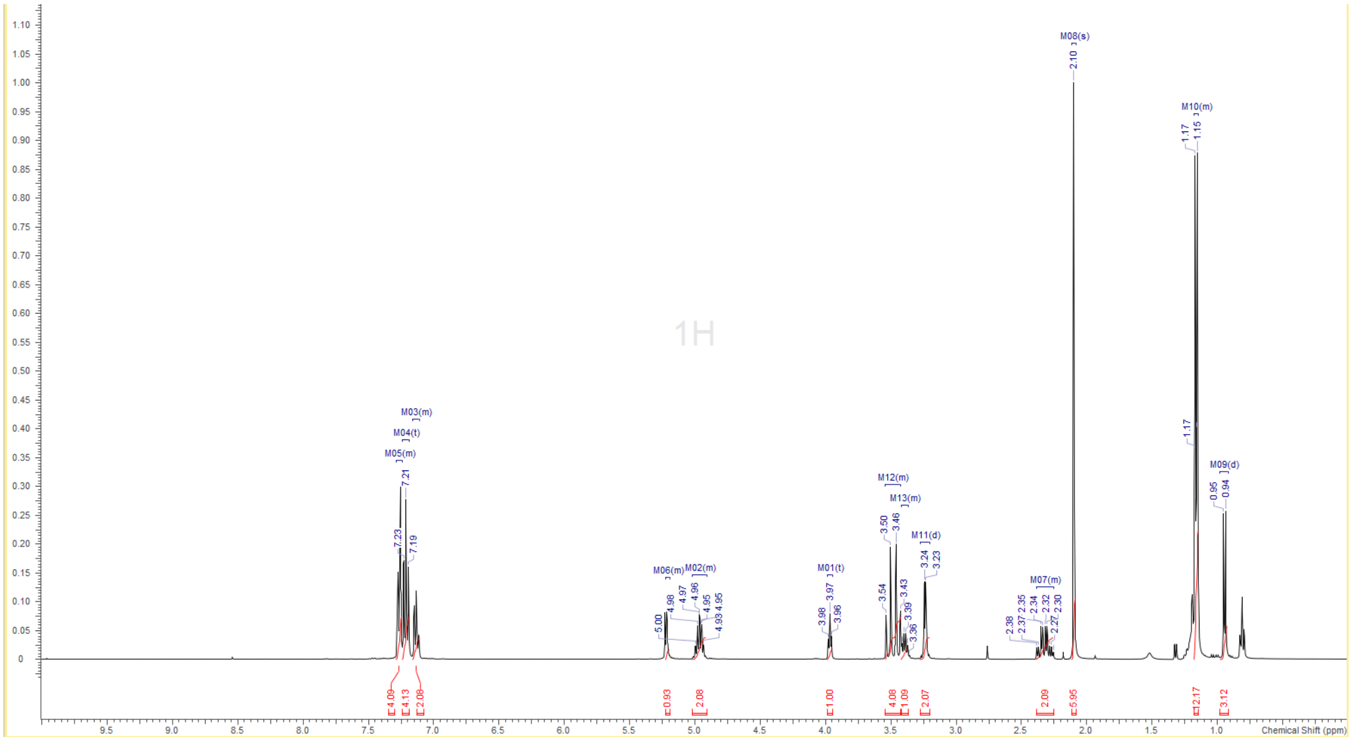

Diisopropyl (R)-4-(((1-aminopropan-2-yl)oxy)methyl)-2,6-dimethyl-1,4-dihydropyridine-3,5-dicarboxylate (1d)

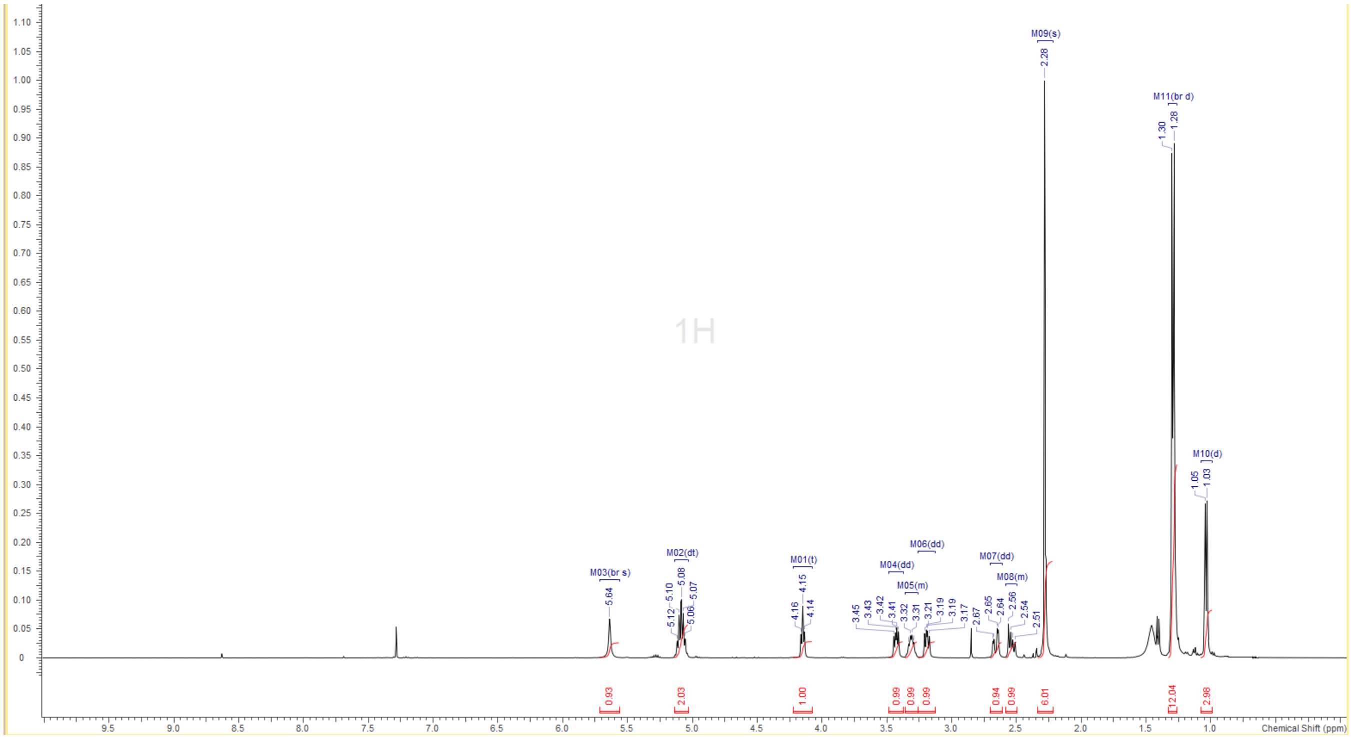

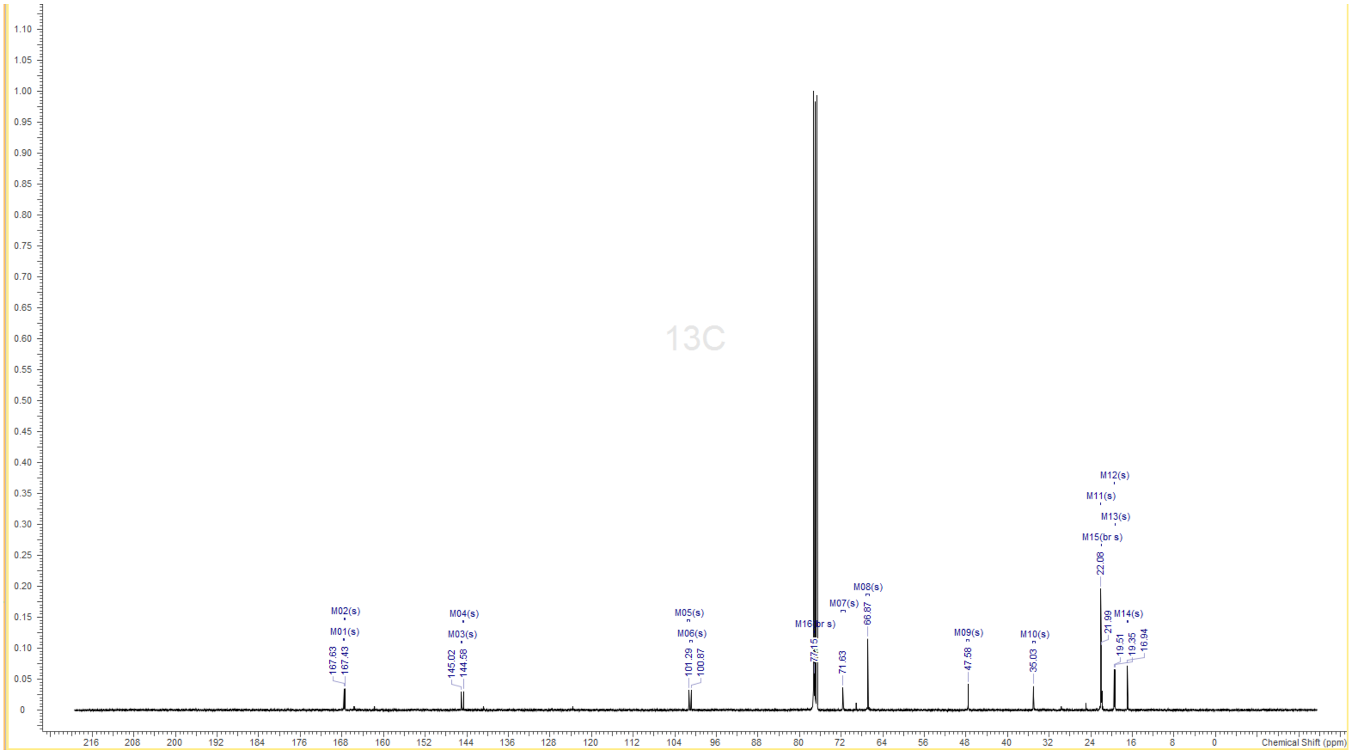

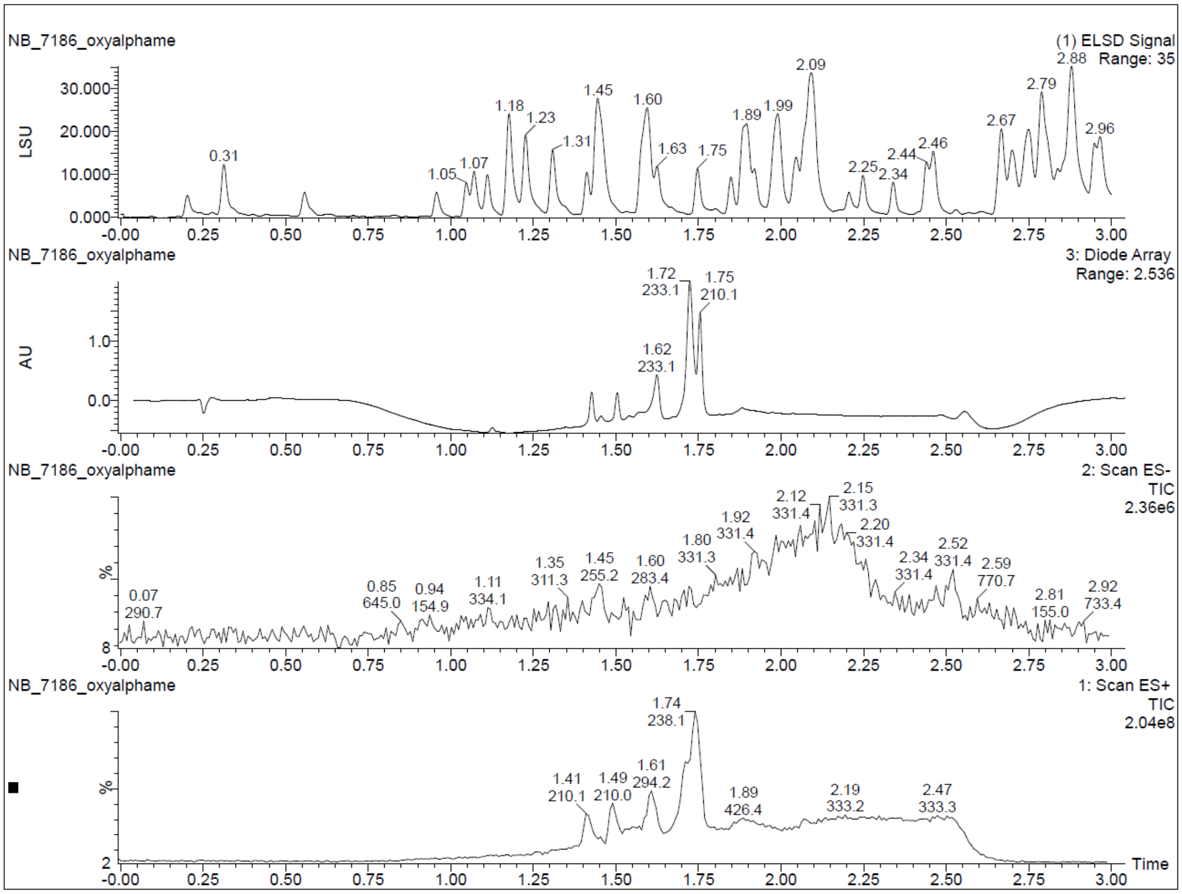
NB_7186_oxyalphame
(1) ELSD Signal
Range: 35
LSU
3: Diode Array
Range: 2.536
AU
2: Scan ES-
TIC
2.36e6
%
1: Scan ES+
TIC
2.04e8
Time

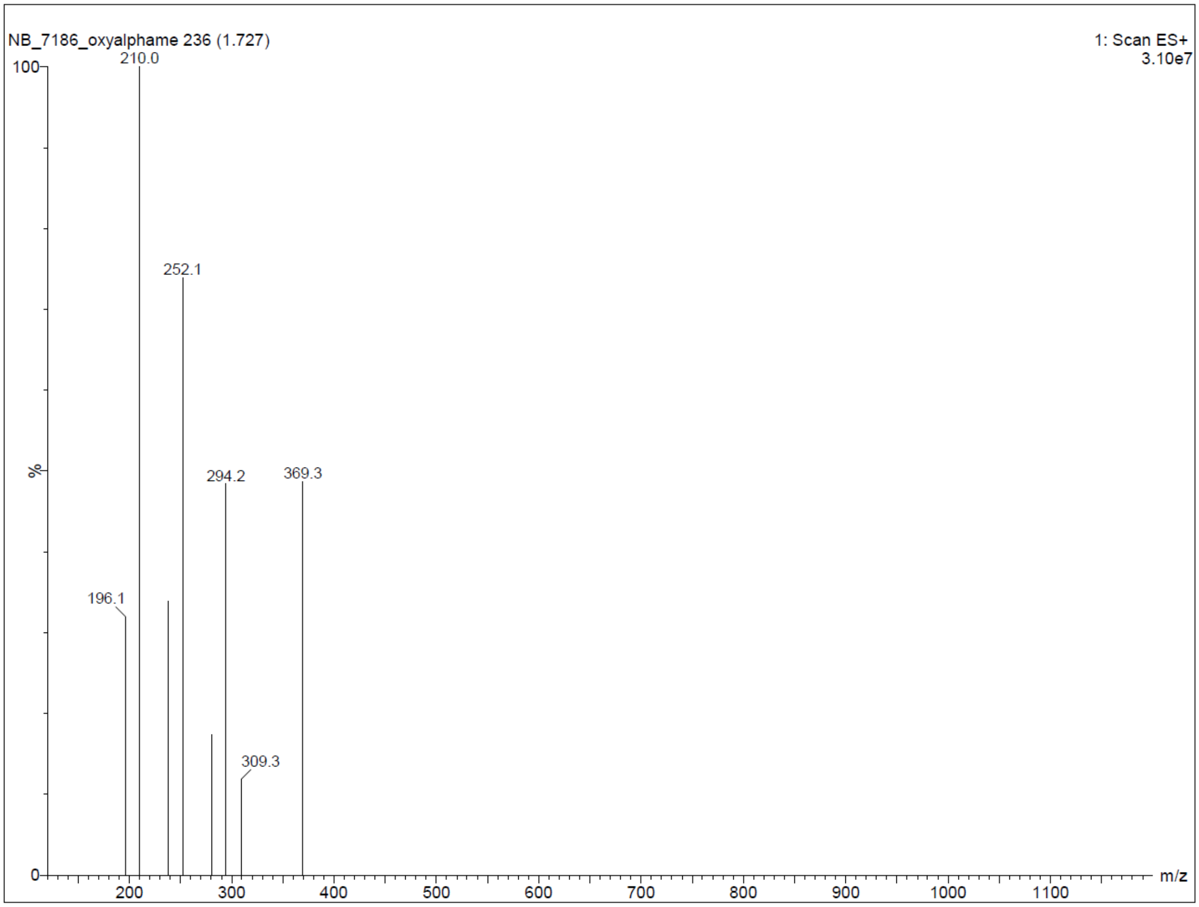
NB_7186_oxyalphame 236 (1.727)
1: Scan ES+
3.10e7
210.0
252.1
294.2
369.3
196.1
309.3
%
m/z

(3S,4R)-4-(Dibenzylamino)tetrahydrofuran-3-ol

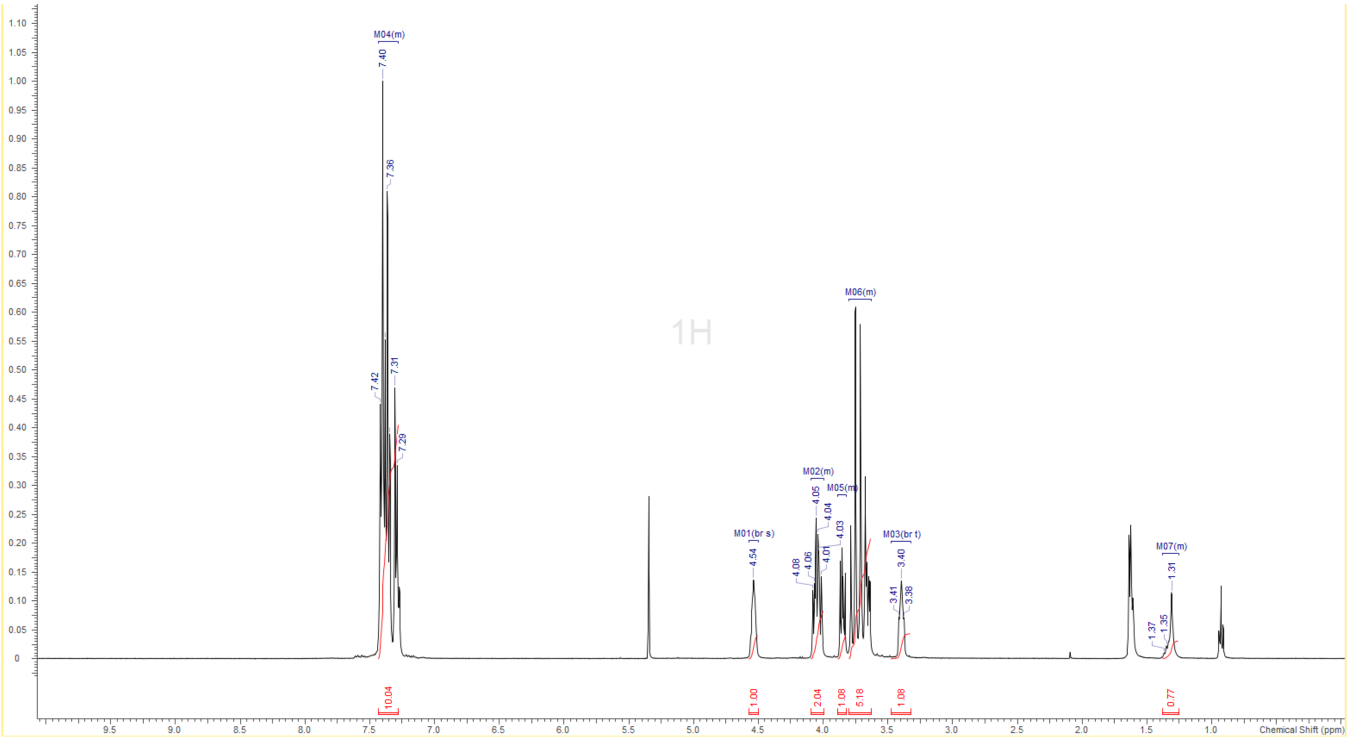

(3R,4S)-N,N-Dibenzyl-4-(2,2-dimethoxyethoxy)tetrahydrofuran-3-amine

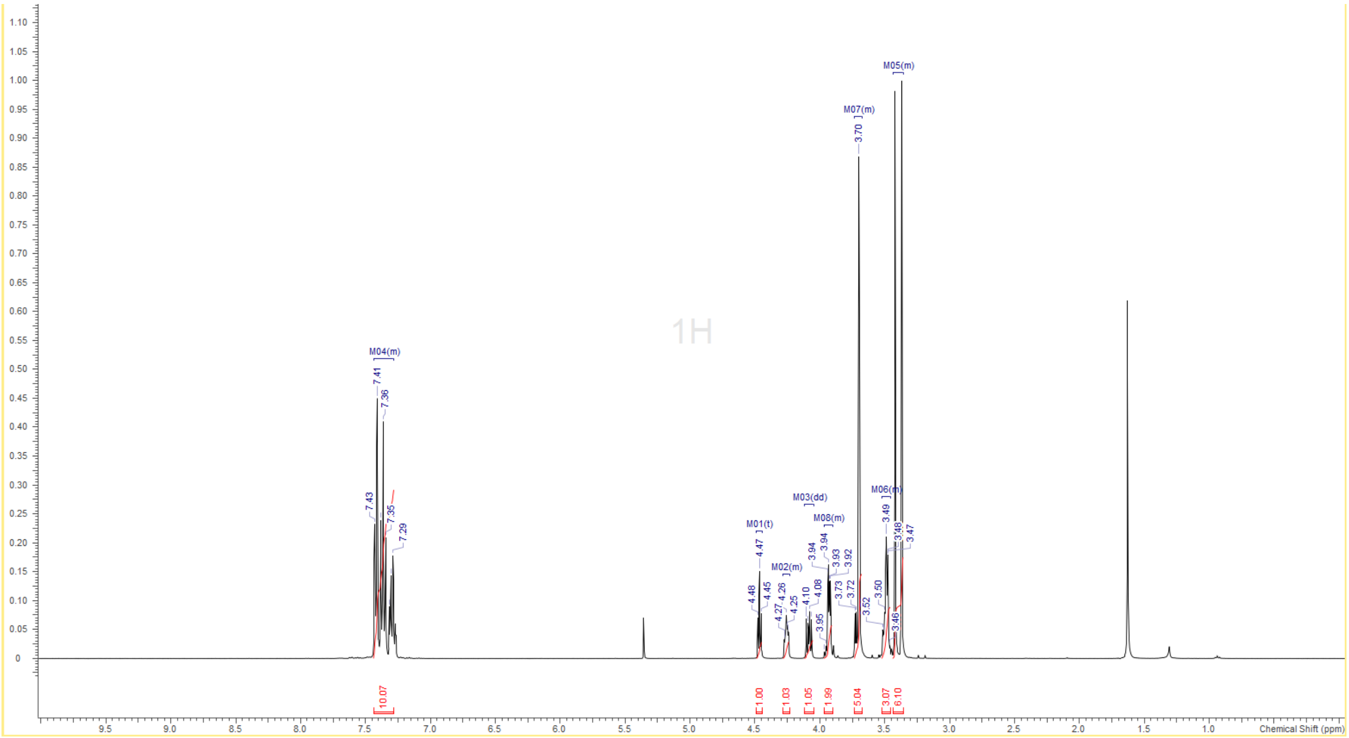

2-(((3S,4R)-4-(Dibenzylamino)tetrahydrofuran-3-yl)oxy)acetaldehyde

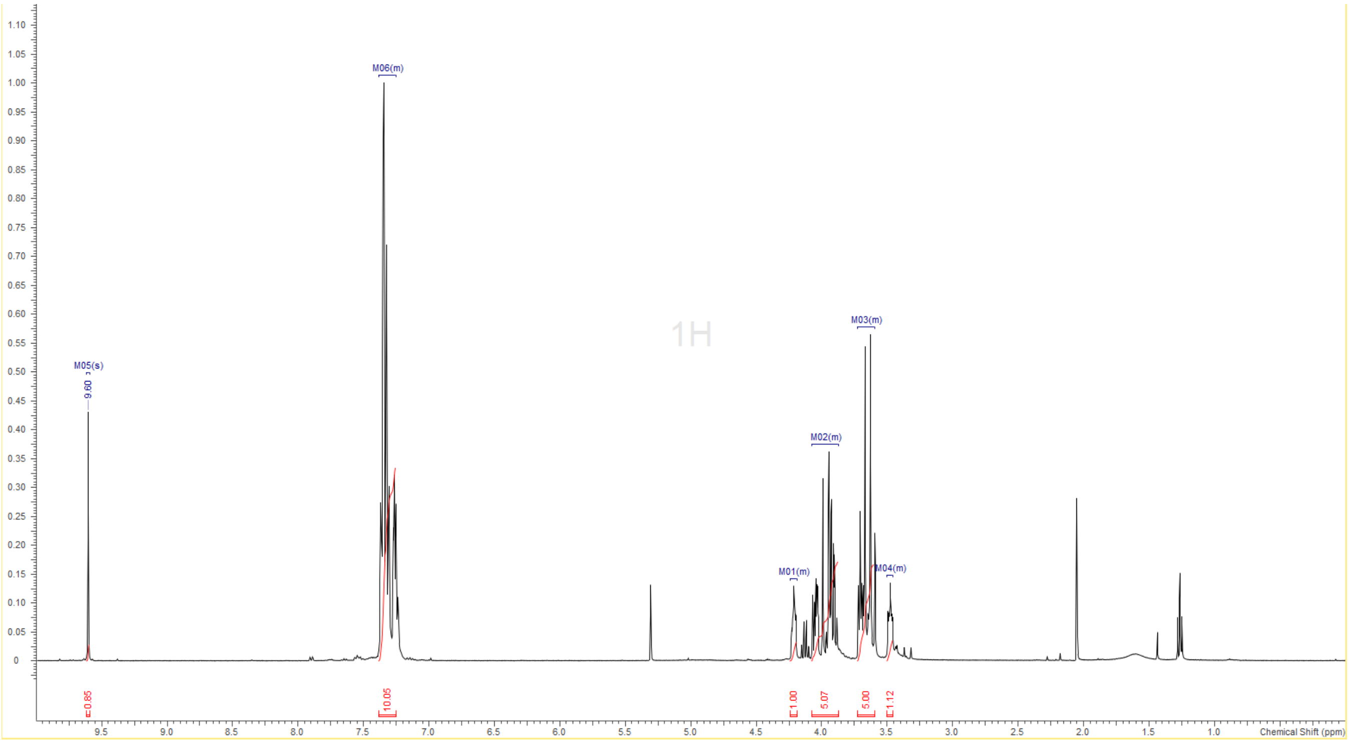

Diisopropyl 4-((((3S,4R)-4-(dibenzylamino)tetrahydrofuran-3-yl)oxy)methyl)-2,6-dimethyl-1,4-dihydropyridine-3,5-dicarboxylate

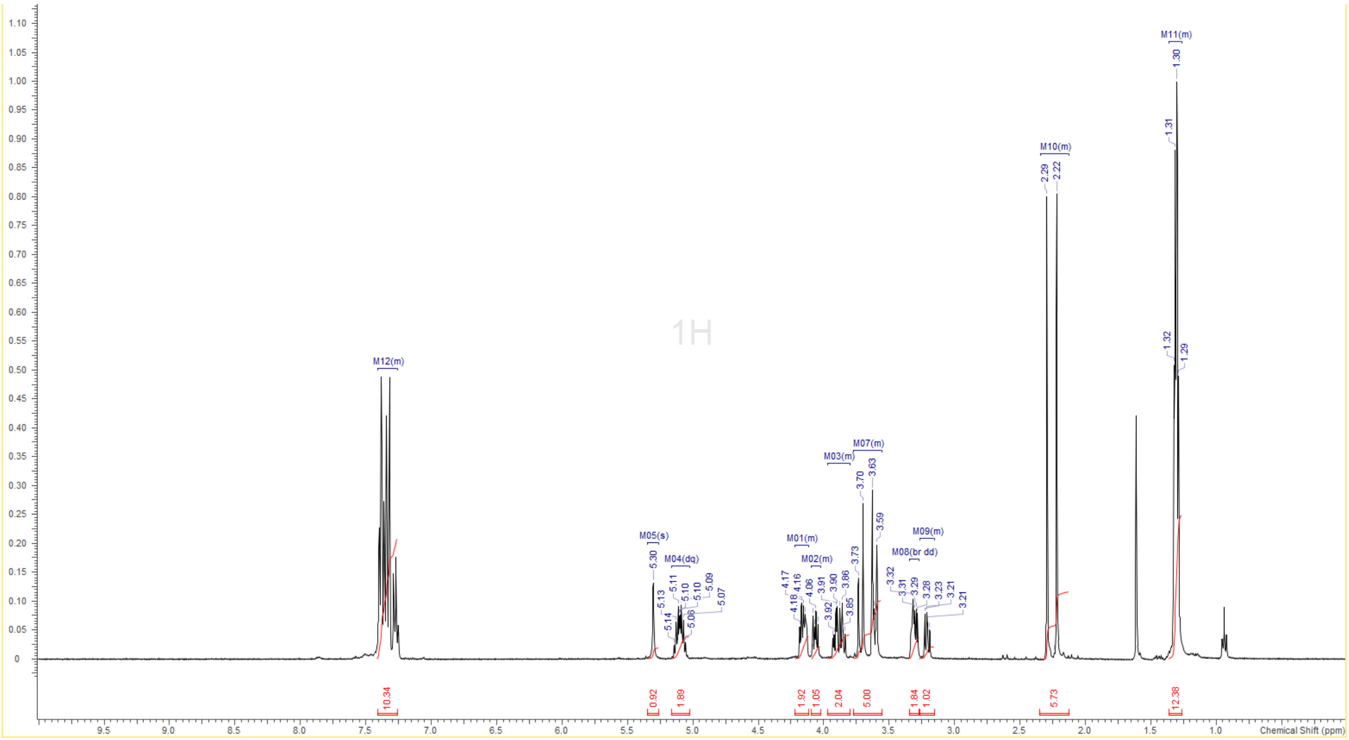

Diisopropyl 4-((((3S,4R)-4-aminotetrahydrofuran-3-yl)oxy)methyl)-2,6-dimethyl-1,4-dihydropyridine-3,5-dicarboxylate (1g)

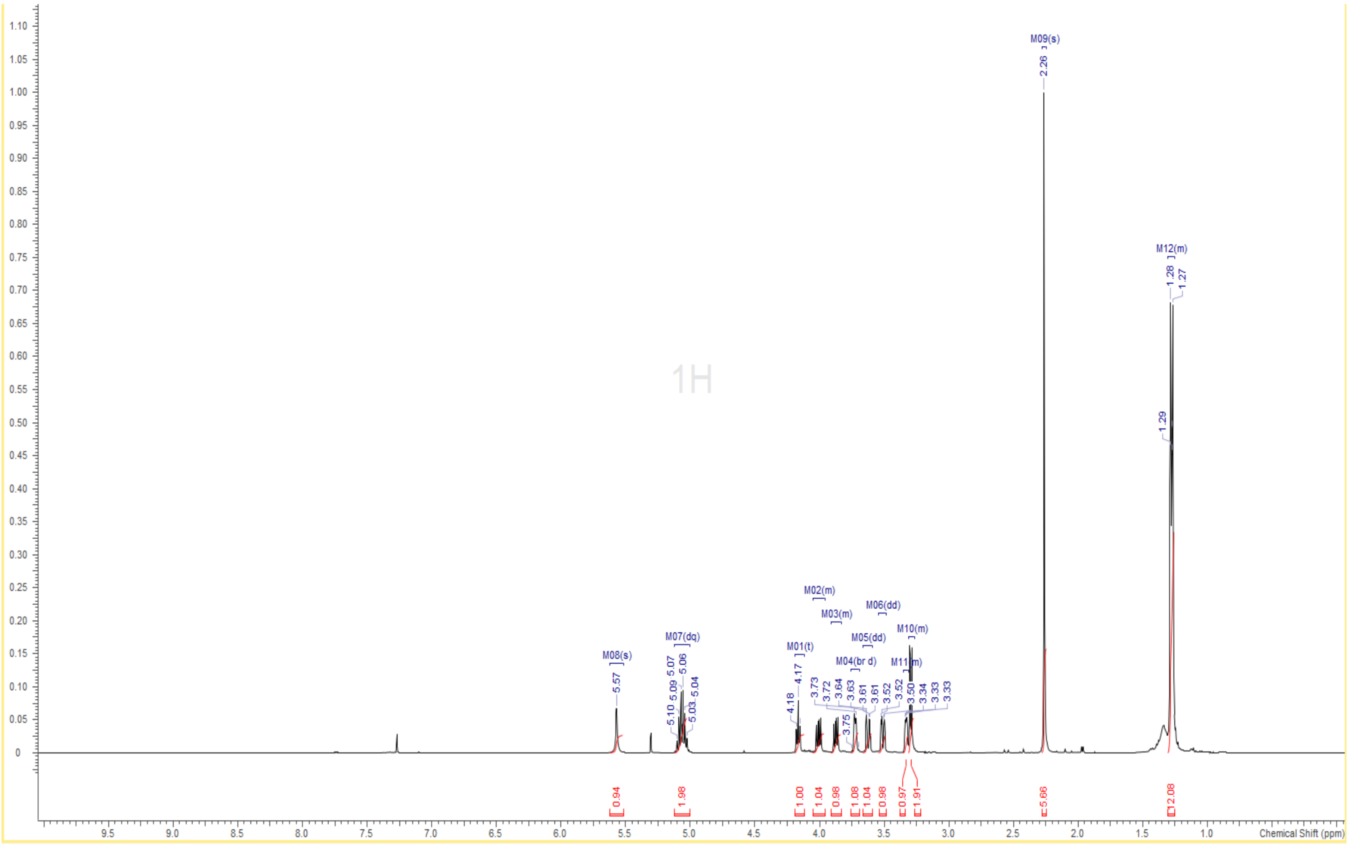
1H
M01(t)
M02(m)
M03(m)
M04(br d)
M05(dd)
M06(dd)
M07(dq)
M08(s)
M09(s)
M10(m)
M11(m)
M12(m)
Chemical Shift (ppm)

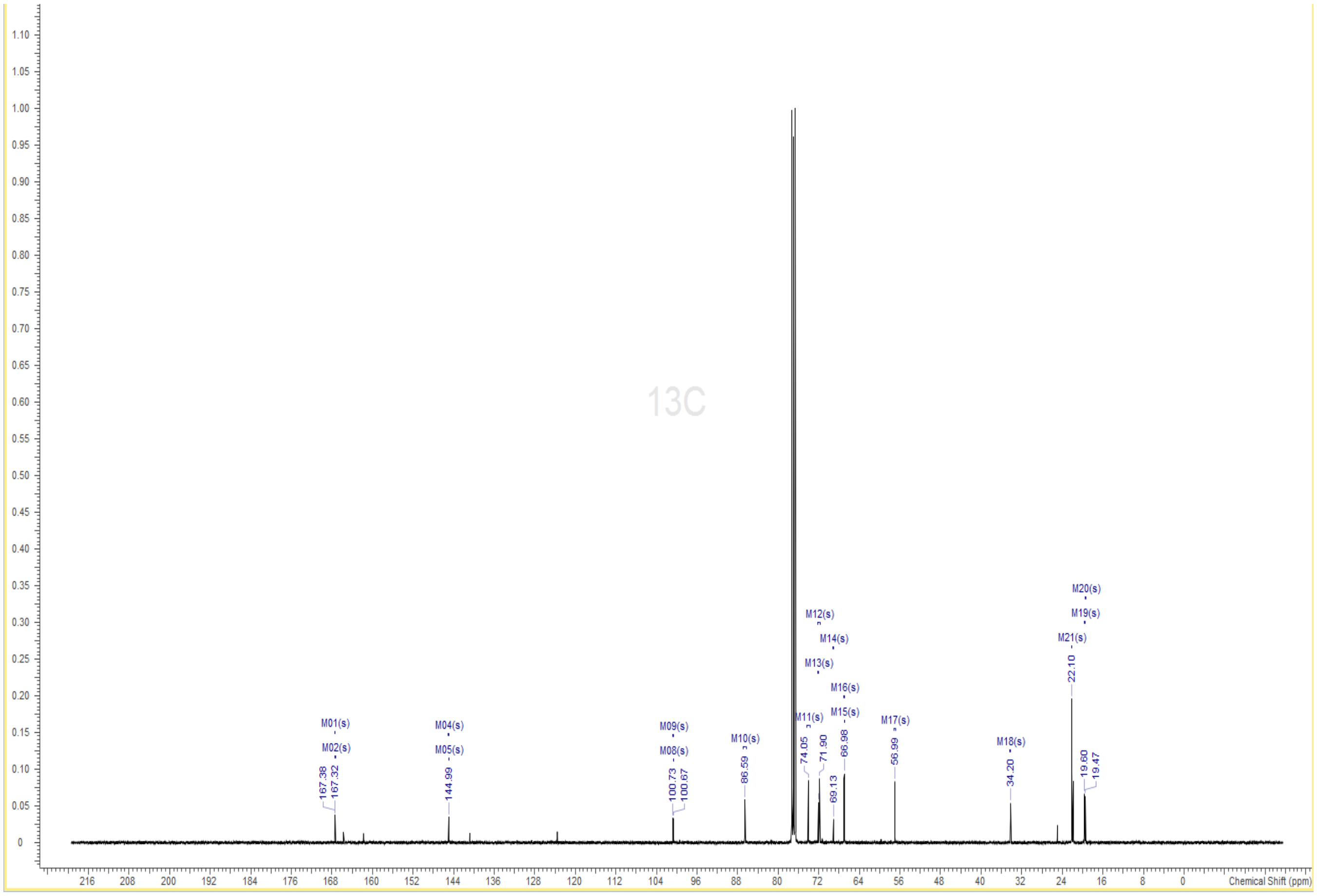
13C
167.38
167.32
144.99
100.73
100.67
86.59
74.05
71.90
69.13
66.98
56.99
34.20
22.10
19.60
19.47
Chemical Shift (ppm)

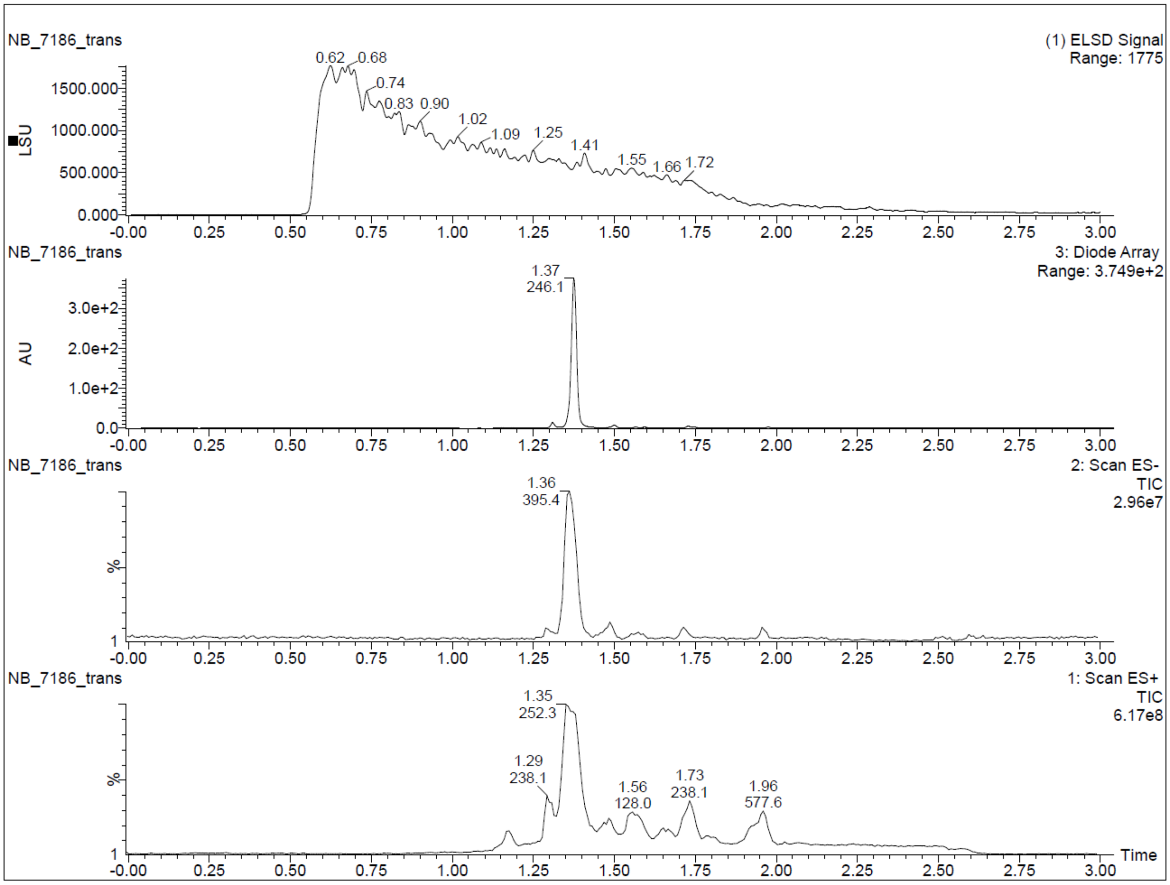
NB_7186_trans
(1) ELSD Signal
Range: 1775
LSU
3: Diode Array
Range: 3.749e+2
AU
2: Scan ES-
TIC
2.96e7
1: Scan ES+
TIC
6.17e8
Time

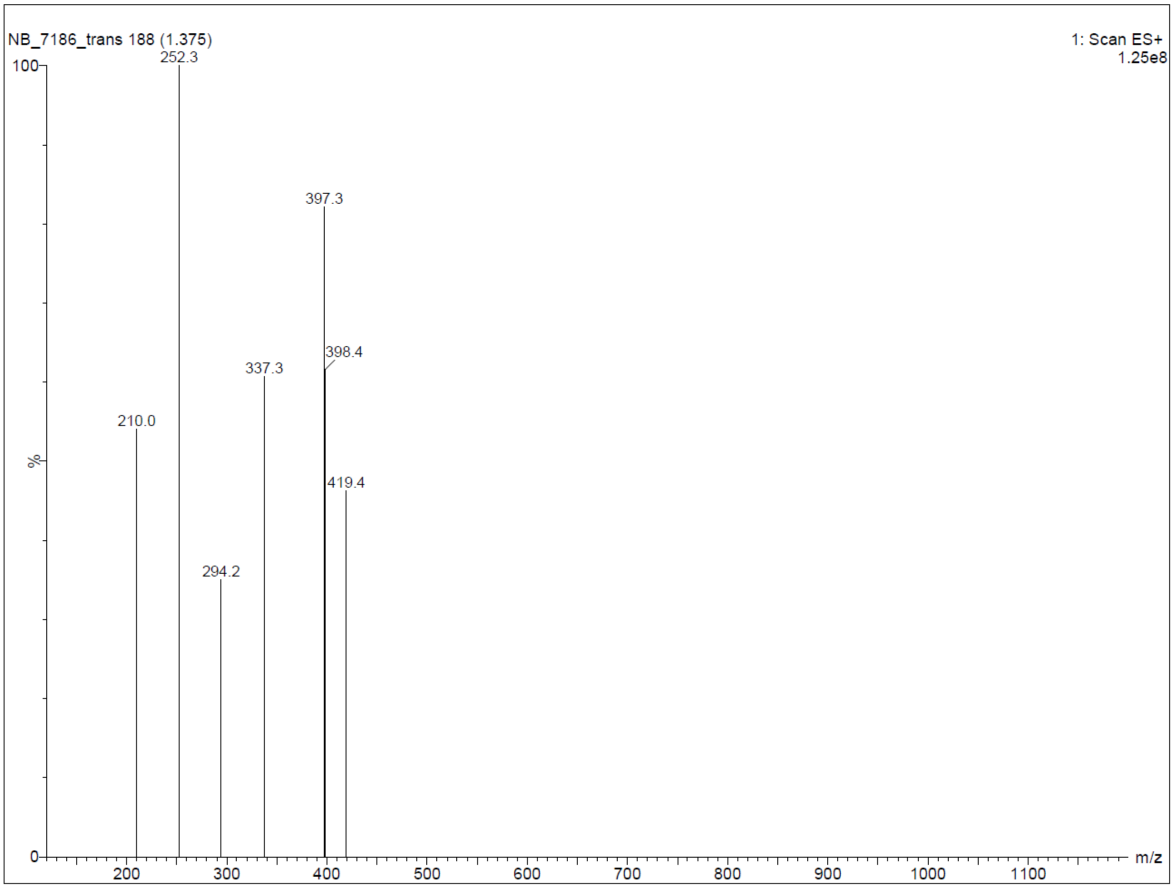
NB_7186_trans 188 (1.375)
1: Scan ES+
1.25e8
252.3
397.3
398.4
337.3
210.0
419.4
294.2
m/z

(S)-2-(2-(2,2-Dimethoxyethoxy)-2-phenylethyl)isoindoline-1,3-dione

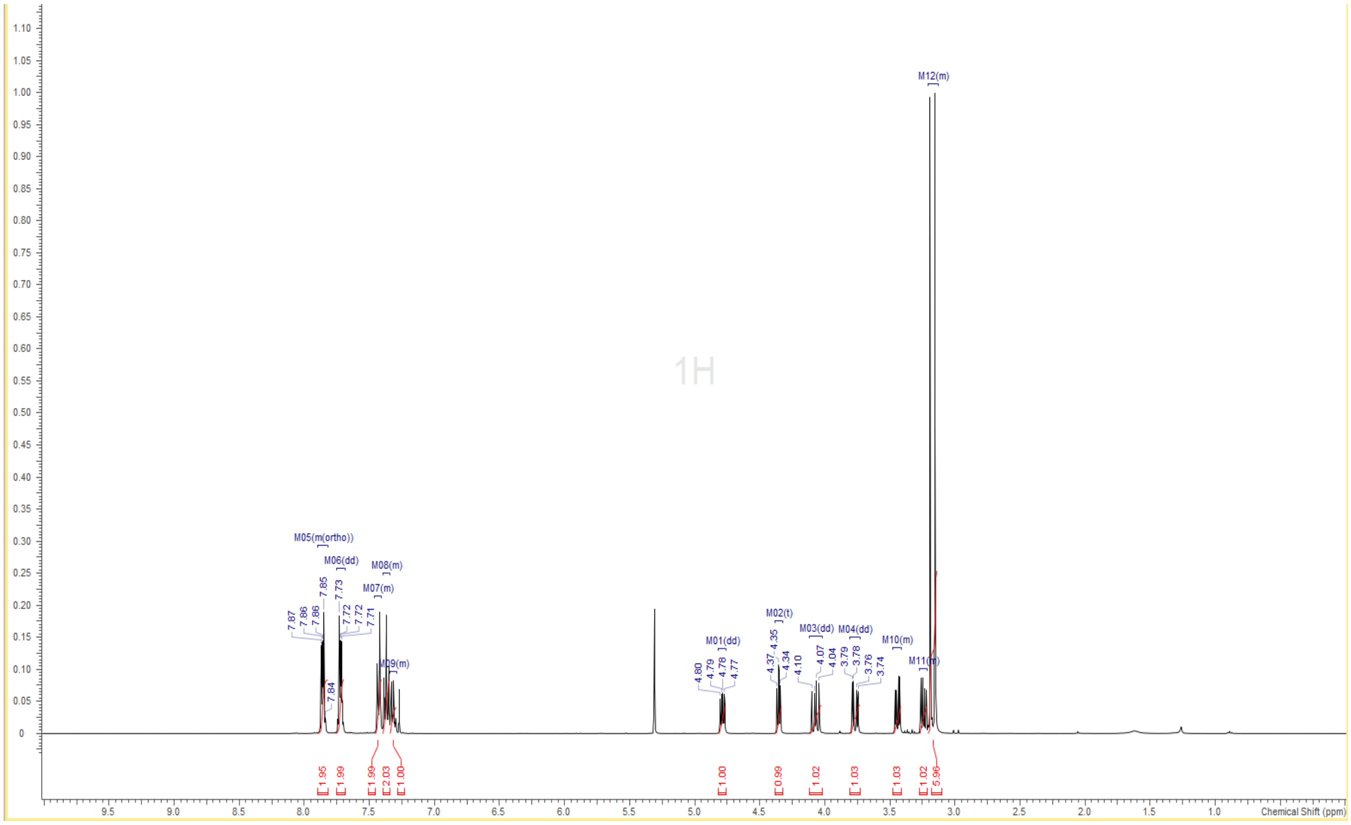

(S)-2-(2-(1,3-Dioxoisoindolin-2-yl)-1-phenylethoxy)acetaldehyde

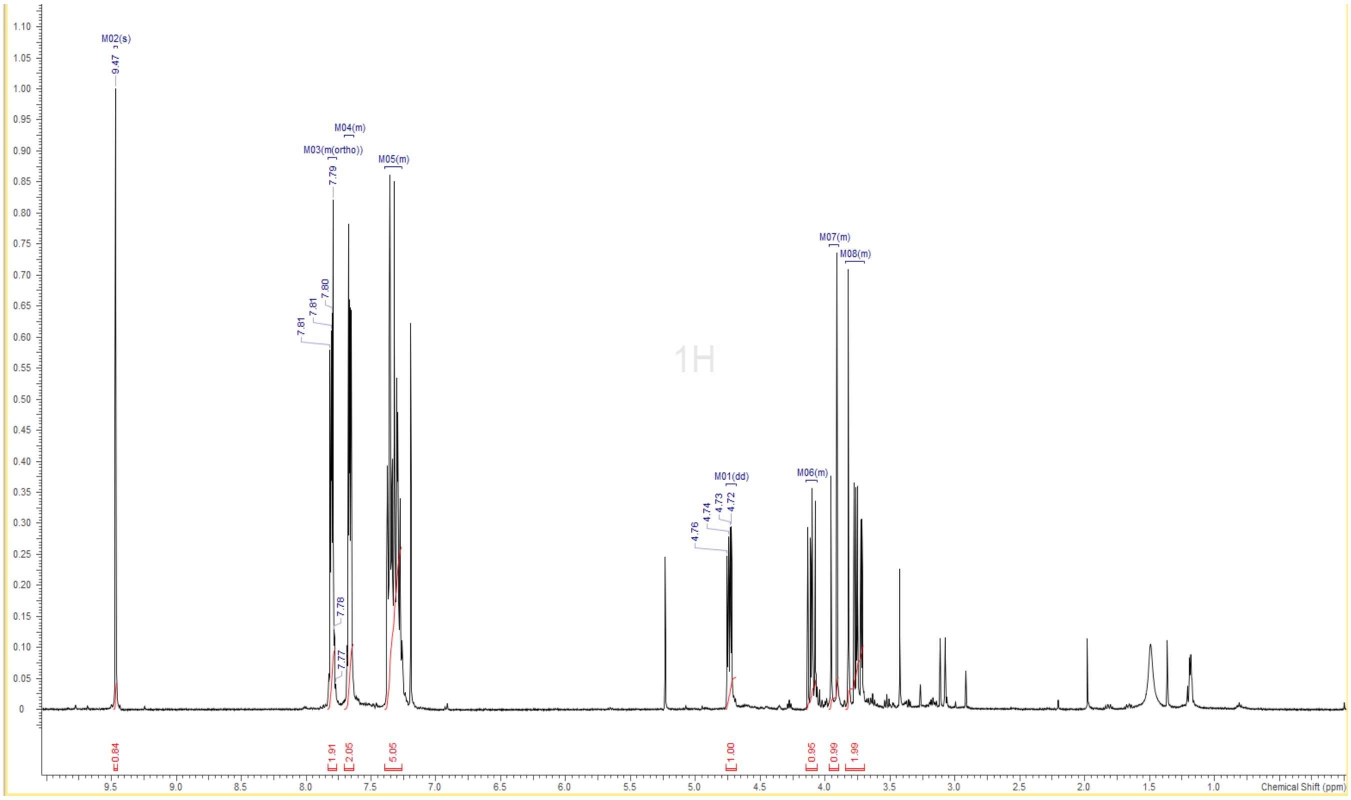

Diisopropyl (S)-4-((2-(1,3-dioxoisoindolin-2-yl)-1-phenylethoxy)methyl)-2,6-dimethyl-1,4-dihydropyridine-3,5-dicarboxylate

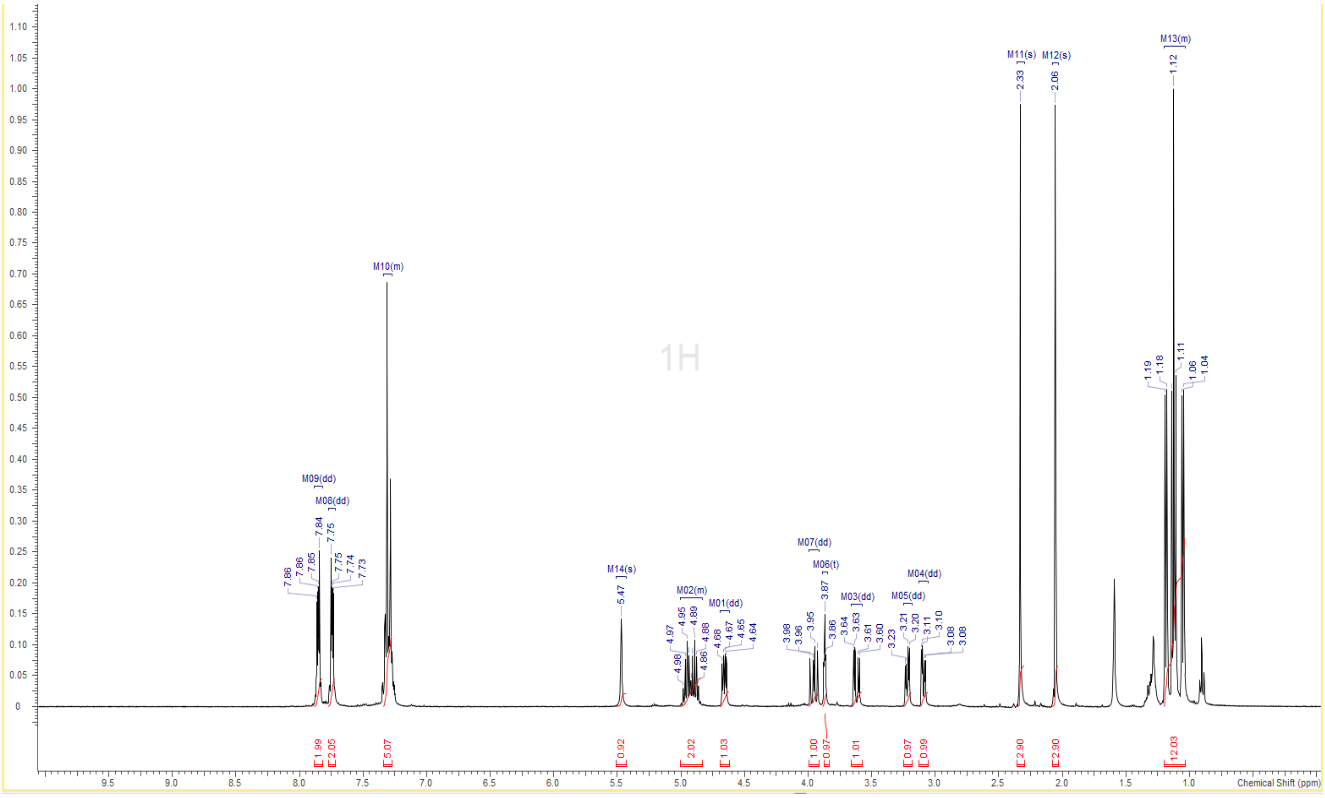

Diisopropyl (S)-4-((2-amino-1-phenylethoxy)methyl)-2,6-dimethyl-1,4-dihydropyridine-3,5-dicarboxylate (1e)

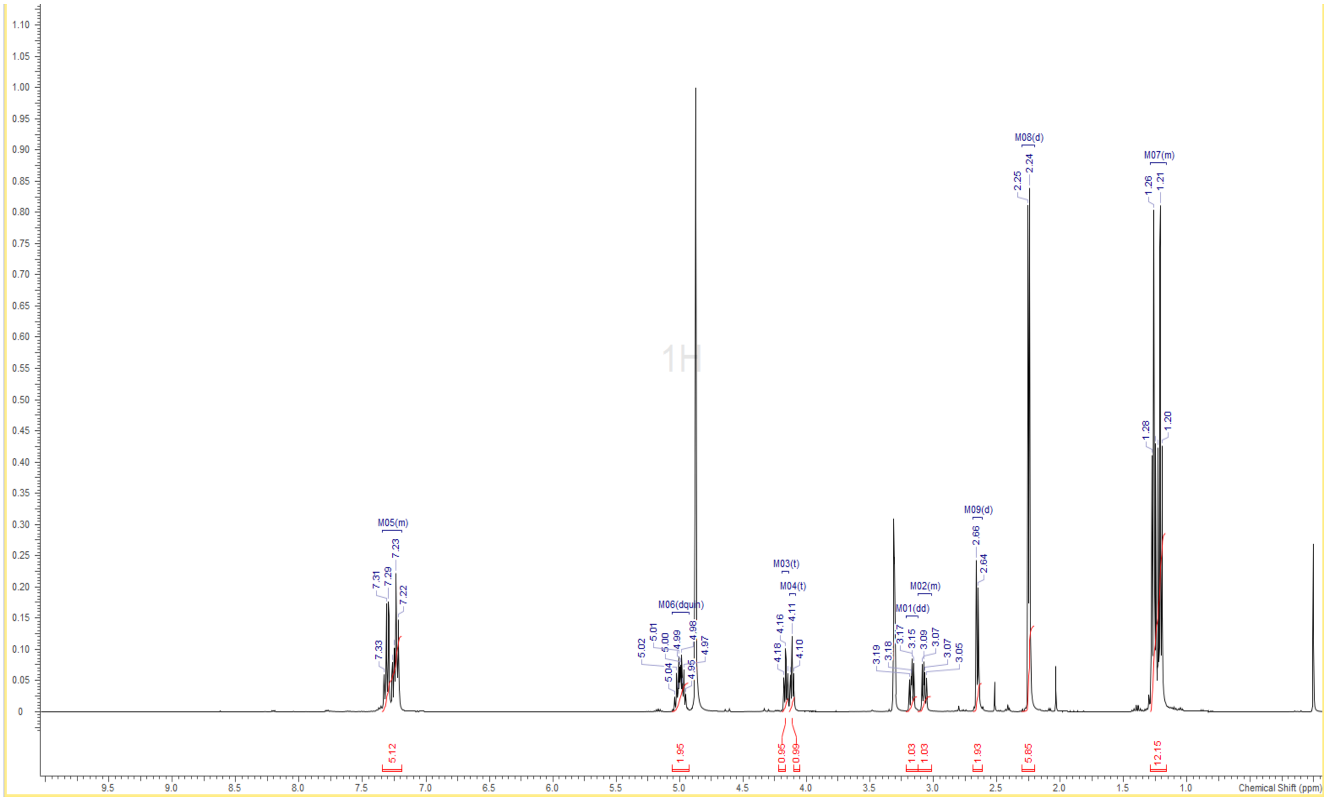

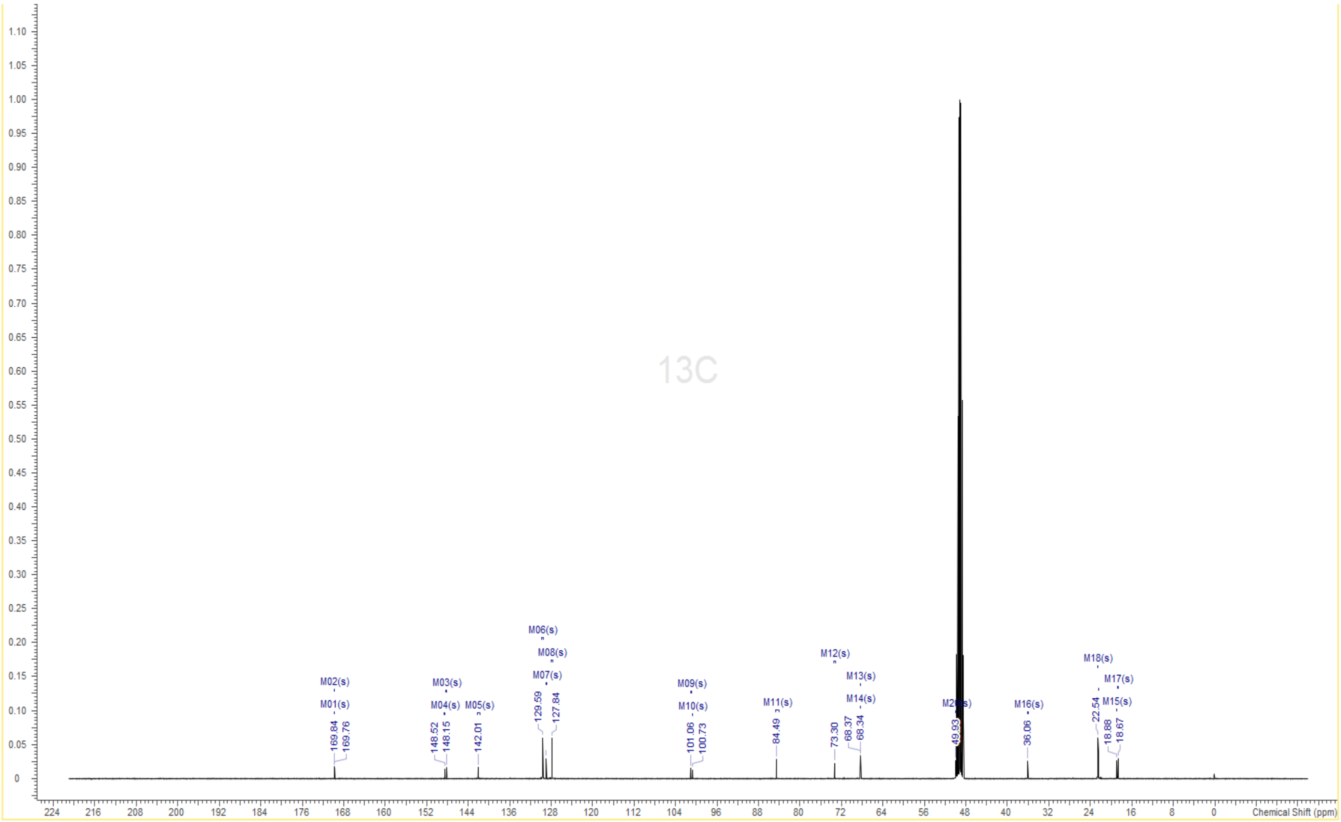
13C
169.84
169.76
148.52
148.15
142.01
129.59
127.84
101.06
100.73
84.49
73.30
68.37
68.34
49.93
36.06
22.54
18.88
18.67
Chemical Shift (ppm)

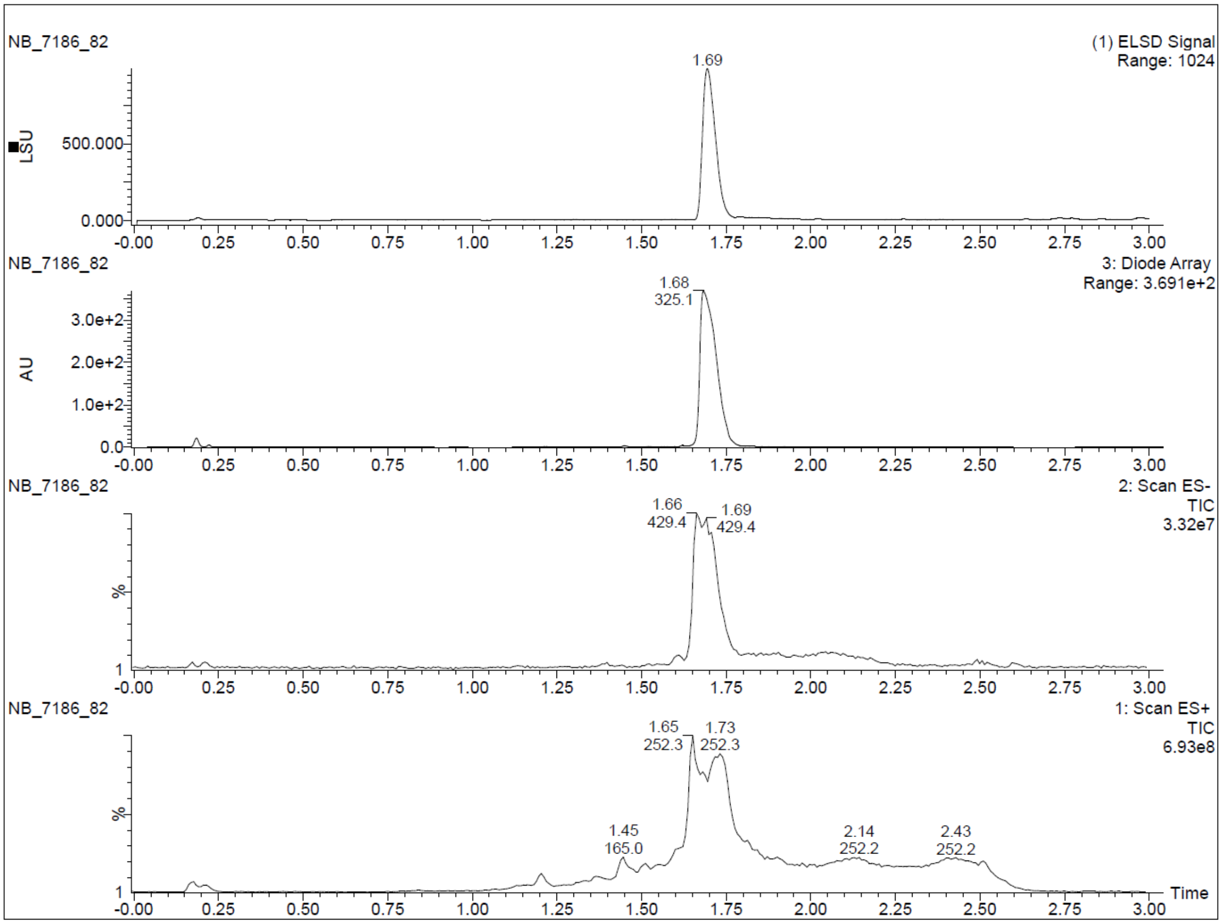
NB_7186_82
(1) ELSD Signal
Range: 1024
LSU
1.69
NB_7186_82
3: Diode Array
Range: 3.691e+2
AU
1.68
325.1
NB_7186_82
2: Scan ES-
TIC
3.32e7
1.66
429.4
1.69
429.4
NB_7186_82
1: Scan ES+
TIC
6.93e8
1.65
252.3
1.73
252.3
1.45
165.0
2.14
252.2
2.43
252.2
Time

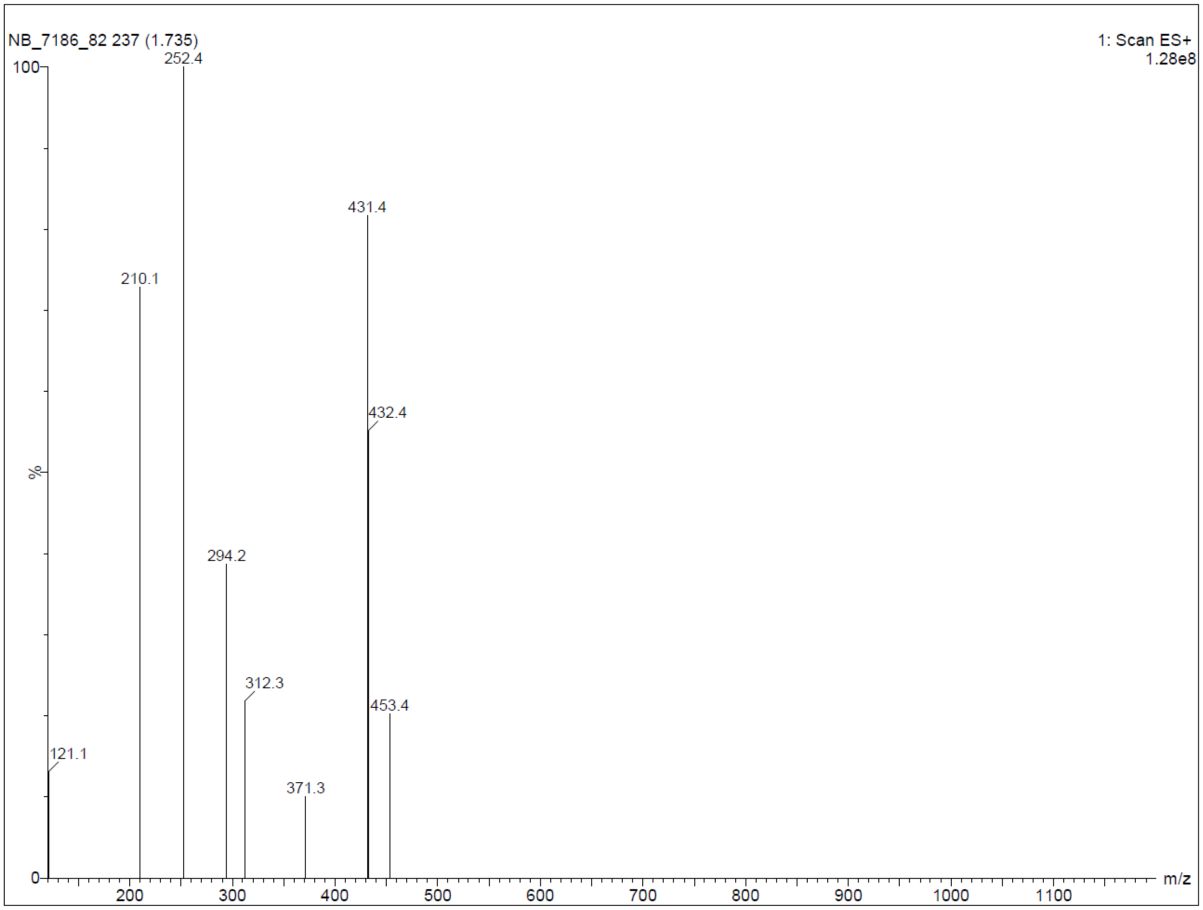

tert-Butyl (3R,4S)-3-(1,3-dioxoisoindolin-2-yl)-4-hydroxypyrrolidine-1-carboxylate

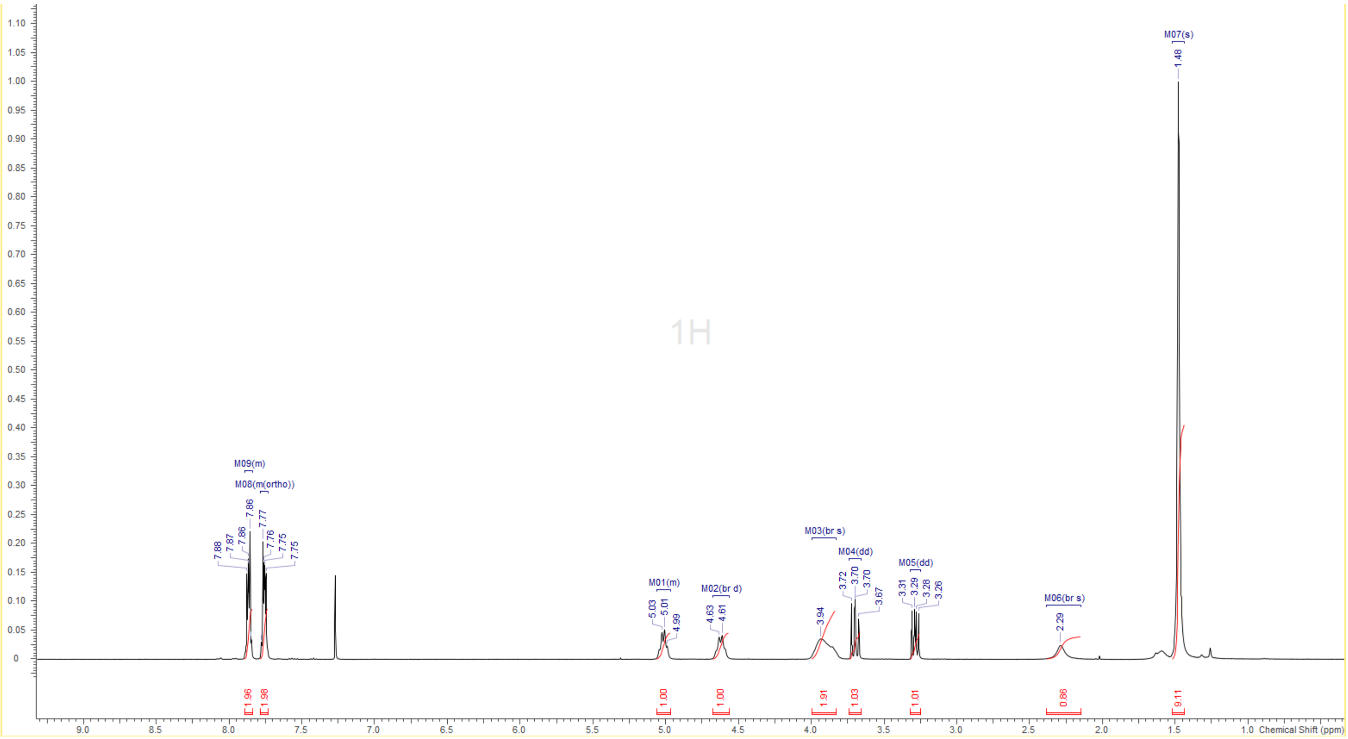

2-((3R,4S)-4-Hydroxypyrrolidin-3-yl)isoindoline-1,3-dione, TFA Salt

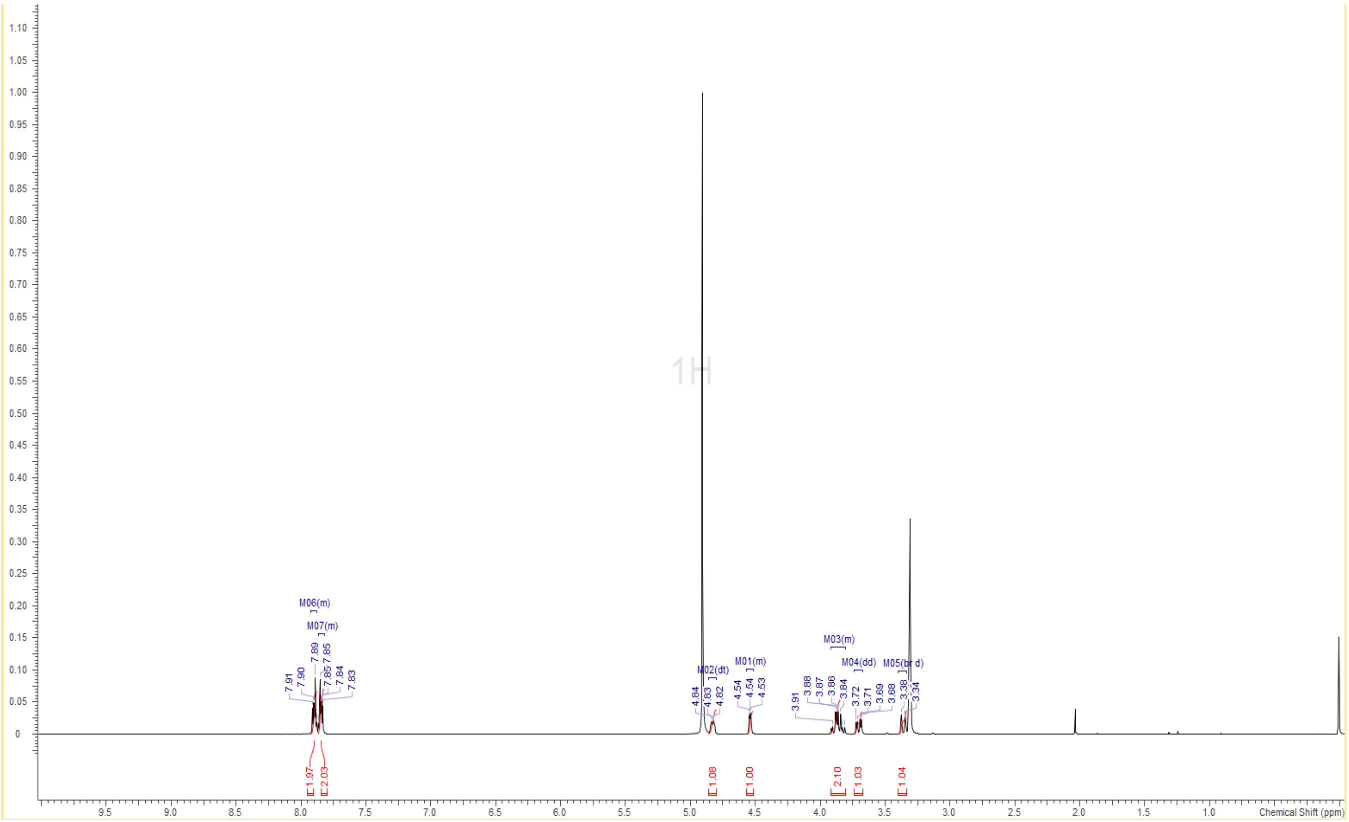

2-((3R,4S)-4-(2,2-Dimethoxyethoxy)-1-tosylpyrrolidin-3-yl)isoindoline-1,3-dione

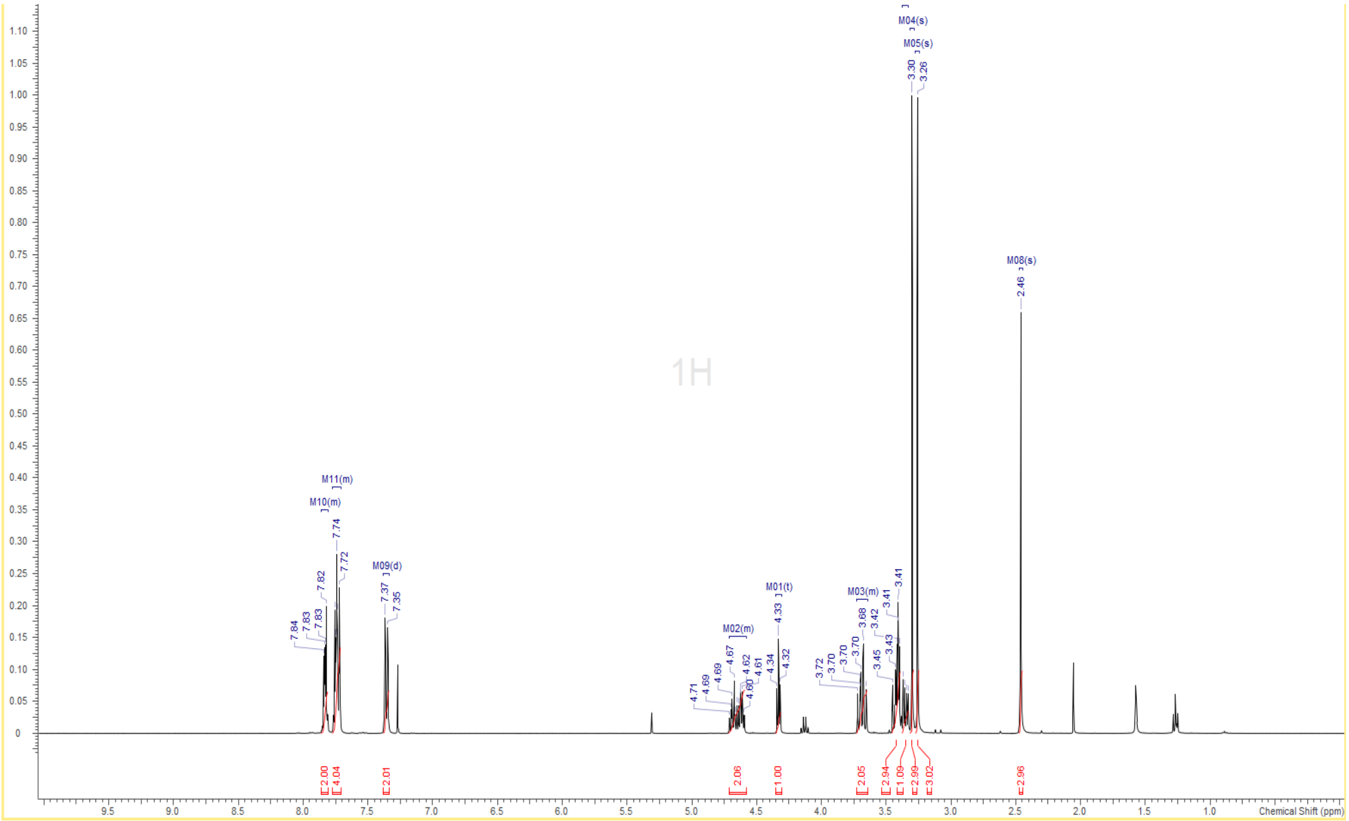

2-(((3S,4R)-4-(1,3-Dioxoisoindolin-2-yl)-1-tosylpyrrolidin-3-yl)oxy)acetaldehyde

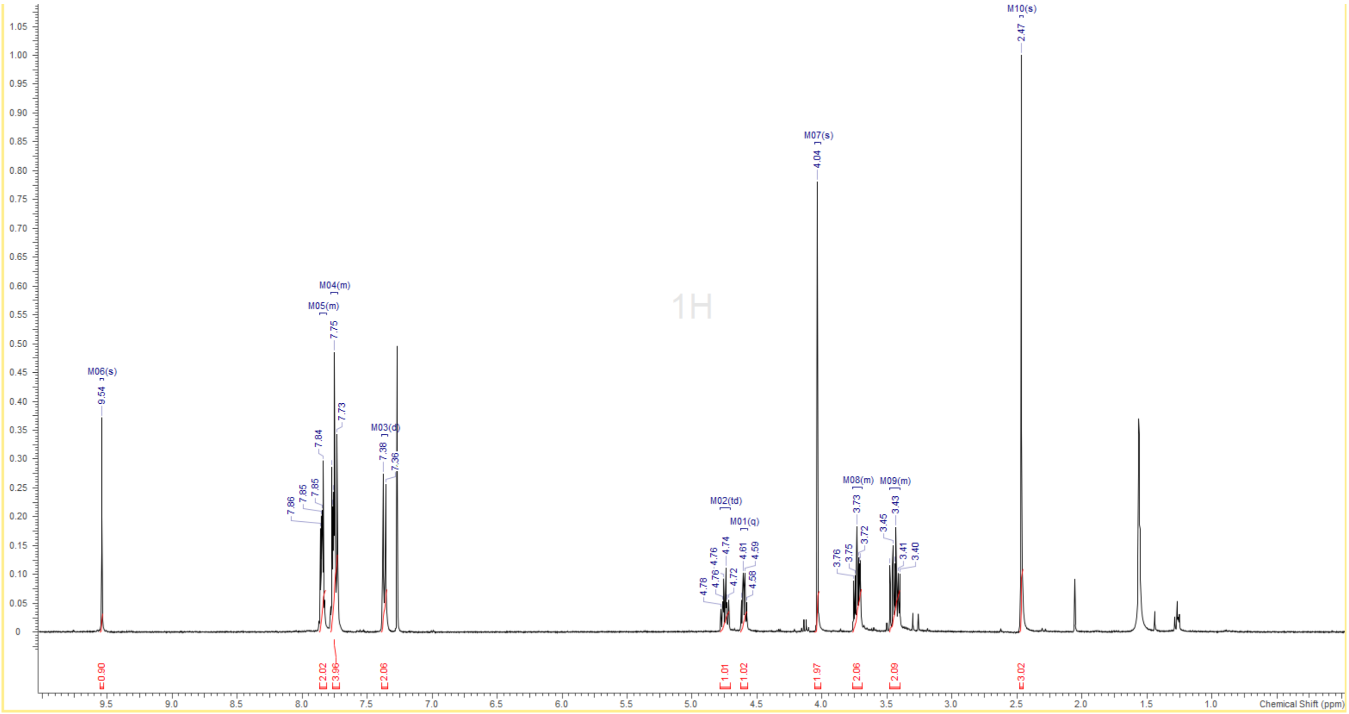

Diisopropyl 4-((((3S,4R)-4-(1,3-dioxoisoindolin-2-yl)-1-tosylpyrrolidin-3-yl)oxy)methyl)-2,6-dimethyl-1,4-dihydropyridine-3,5-dicarboxylate

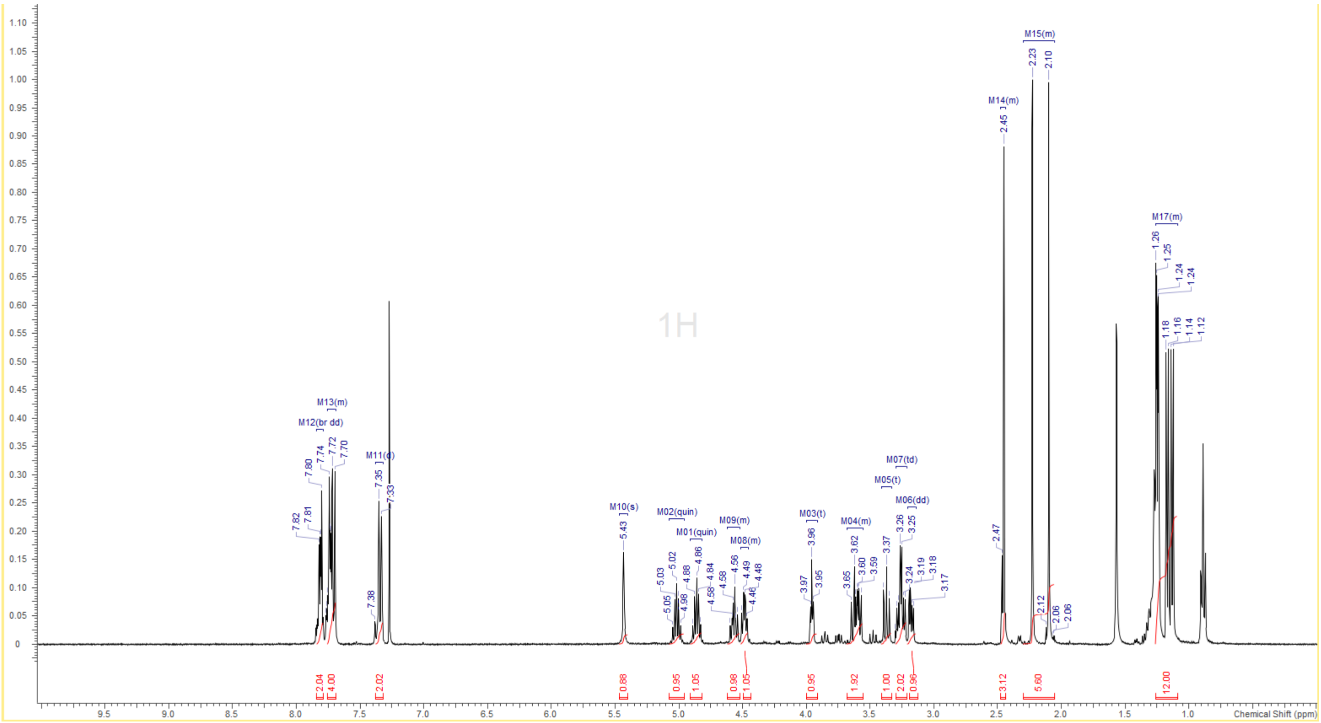

Diisopropyl 4-((((3S,4R)-4-amino-1-tosylpyrrolidin-3-yl)oxy)methyl)-2,6-dimethyl-1,4-dihydropyridine-3,5-dicarboxylate (1h)

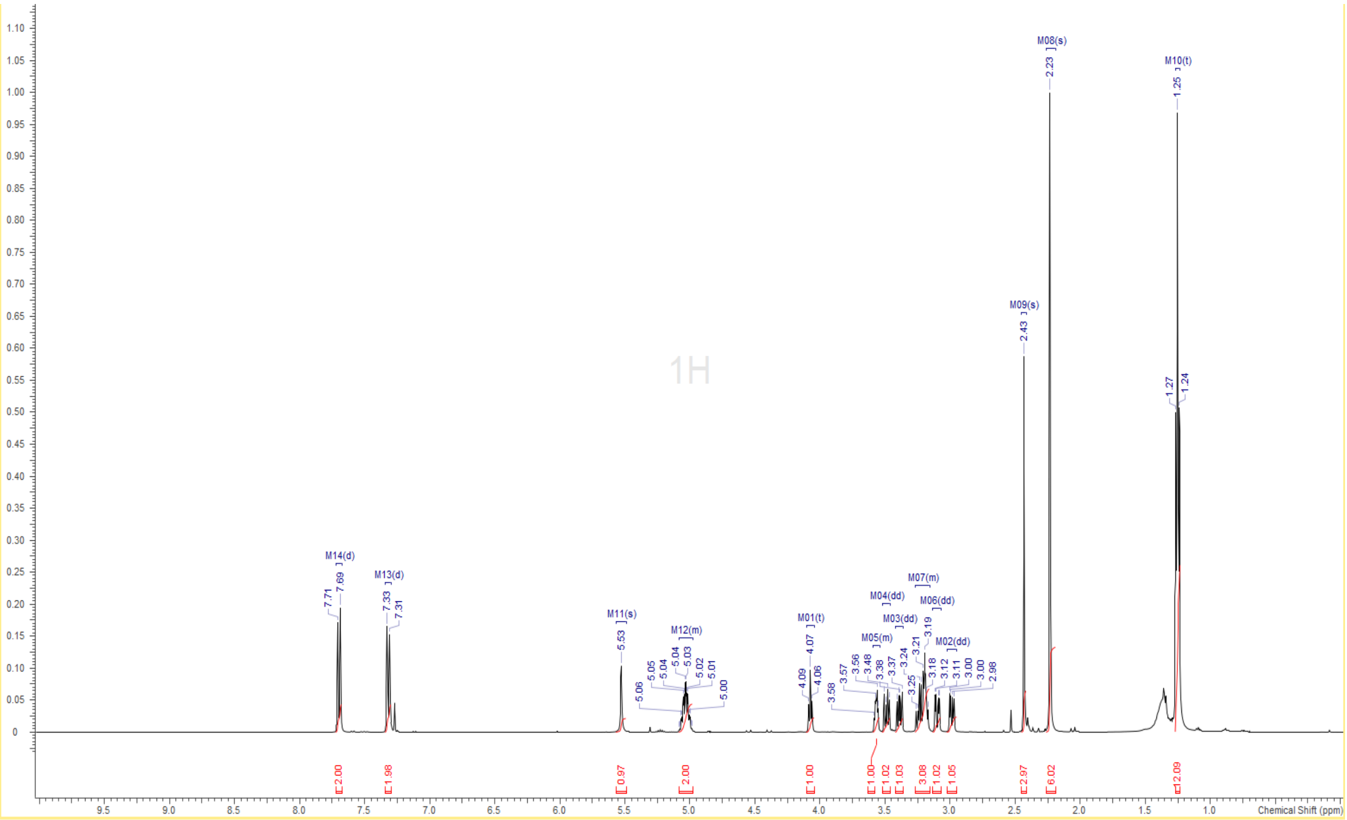
1H
M14(d) 7.71 7.69
M13(d) 7.33 7.31
M11(s) 5.53
M12(m) 5.06 5.05 5.04 5.04 5.03 5.02 5.01 5.00
M01(t) 4.09 4.07 4.06
M05(m) 3.58 3.57 3.56
M04(dd) 3.48 3.38
M03(dd) 3.37 3.24
M07(m) 3.25 3.21 3.19
M06(dd) 3.18 3.12
M02(dd) 3.11 3.00 3.00 2.98
M09(s) 2.43
M08(s) 2.23
M10(t) 1.27 1.25 1.24
2.00 1.98 0.97 2.00 1.00 1.00 1.02 1.03 3.08 1.02 1.05 2.97 6.02 12.09
Chemical Shift (ppm)

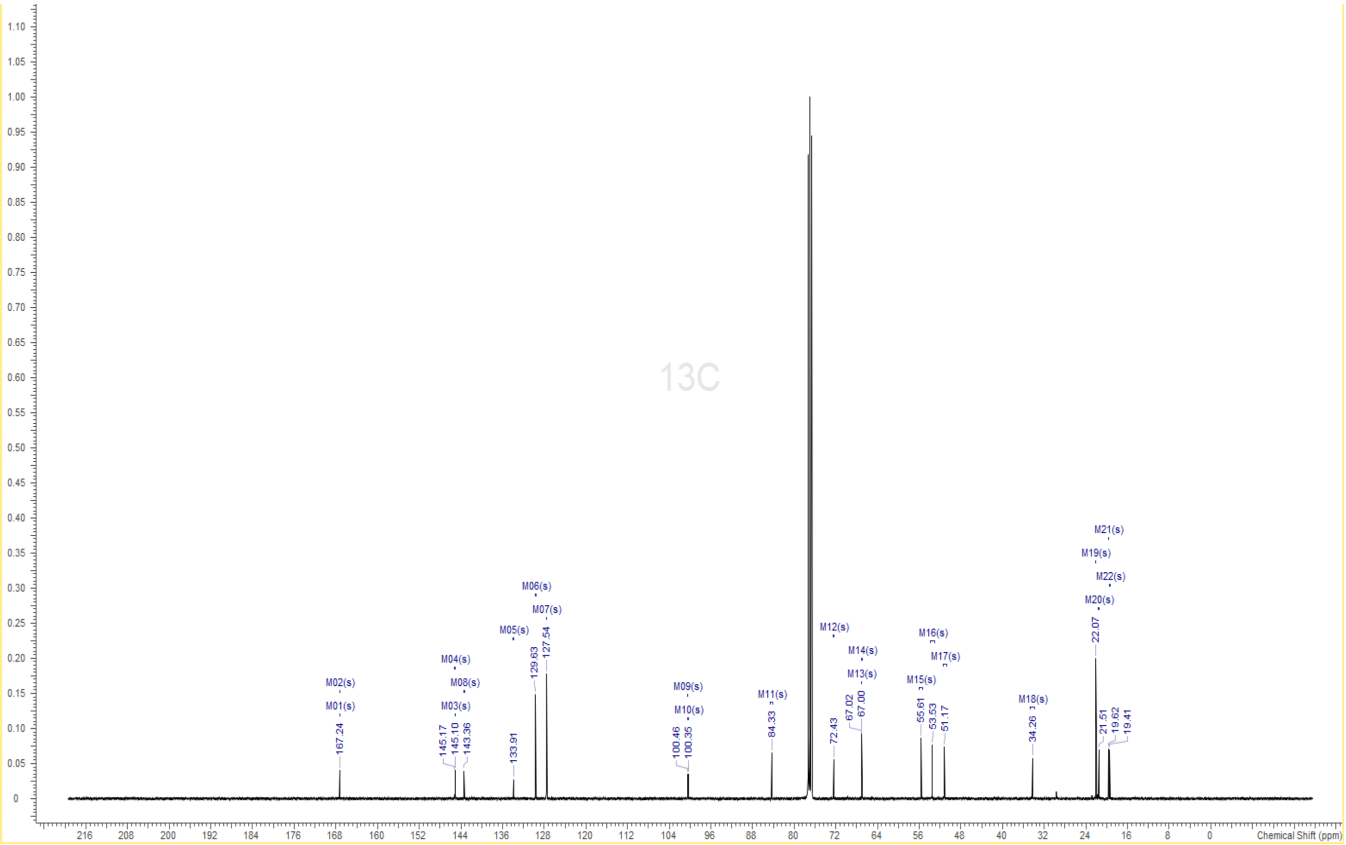
13C
M02(s) M01(s) 167.24
M04(s) M08(s) M03(s) 145.17 145.10 143.36
133.91
M05(s) M06(s) M07(s) 129.63 127.54
M09(s) M10(s) 100.46 100.35
M11(s) 84.33
M12(s) 72.43
M14(s) M13(s) 67.02 67.00
M15(s) M16(s) M17(s) 55.61 53.53 51.17
M18(s) 34.26
M21(s) M19(s) M22(s) M20(s) 22.07 21.51 19.62 19.41
Chemical Shift (ppm)

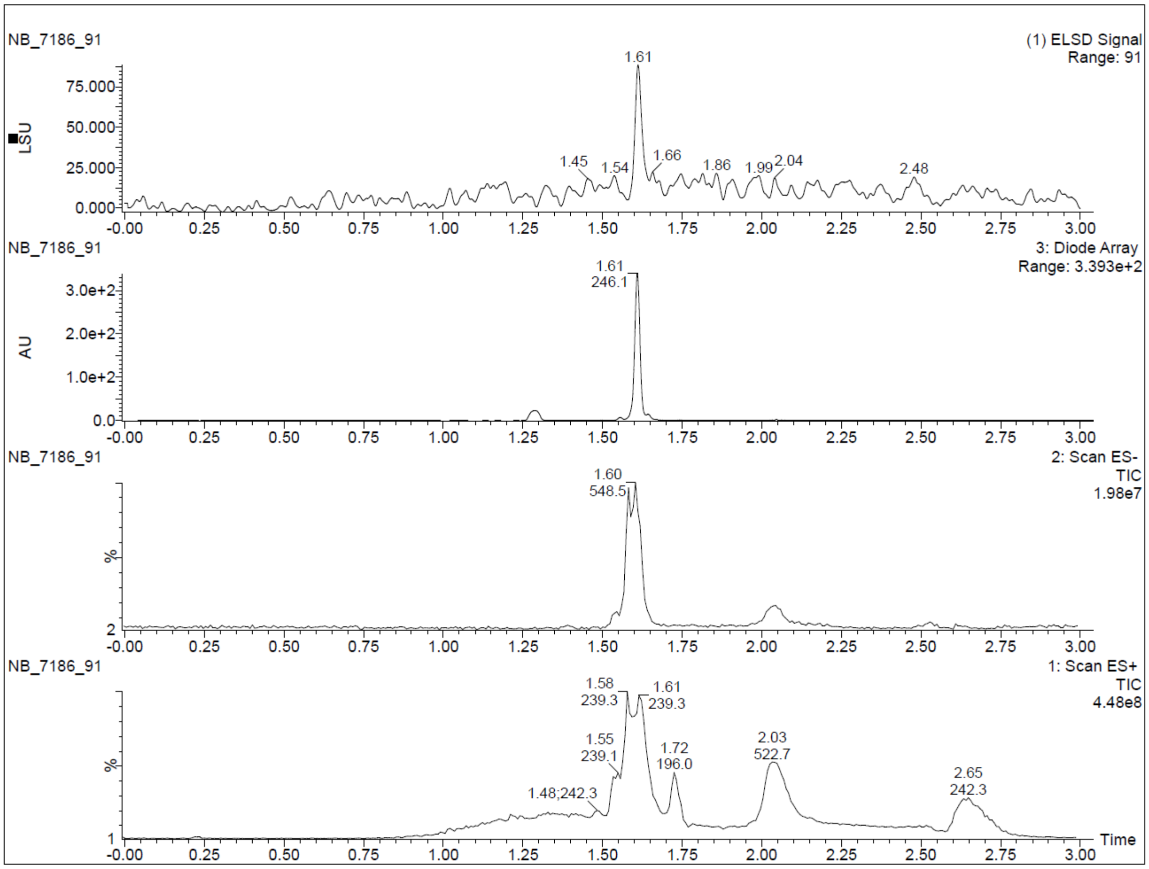
NB_7186_91
(1) ELSD Signal
Range: 91
LSU
1.61
1.45
1.54
1.66
1.86
1.99
2.04
2.48
3: Diode Array
Range: 3.393e+2
AU
246.1
2: Scan ES-
TIC
1.98e7
1.60
548.5
1: Scan ES+
TIC
4.48e8
1.58
239.3
1.55
239.1
1.48;242.3
1.72
196.0
2.03
522.7
2.65
242.3
Time

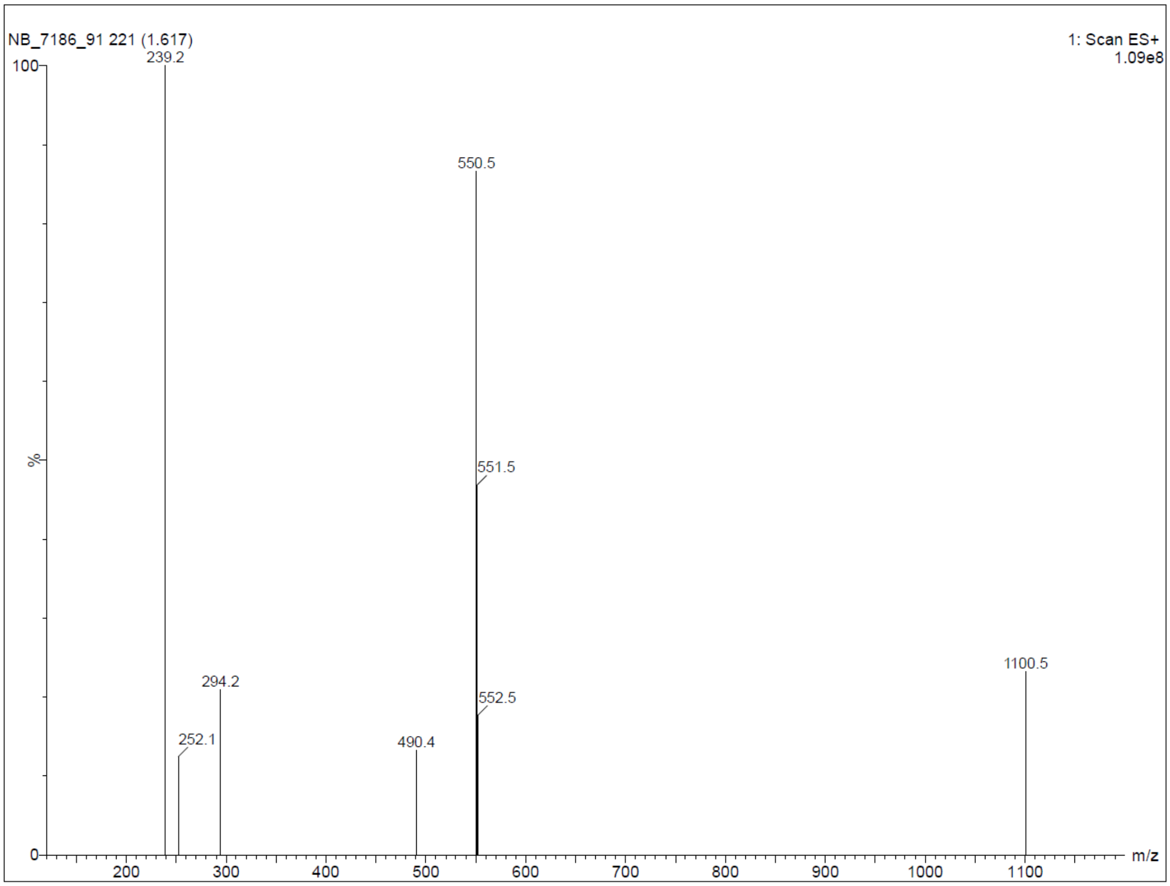
NB_7186_91 221 (1.617)
1: Scan ES+
1.09e8
239.2
550.5
551.5
552.5
294.2
252.1
490.4
1100.5
%
m/z

tert-Butyl (3S,4S)-3-(1,3-dioxoisoindolin-2-yl)-4-hydroxypyrrolidine-1-carboxylate

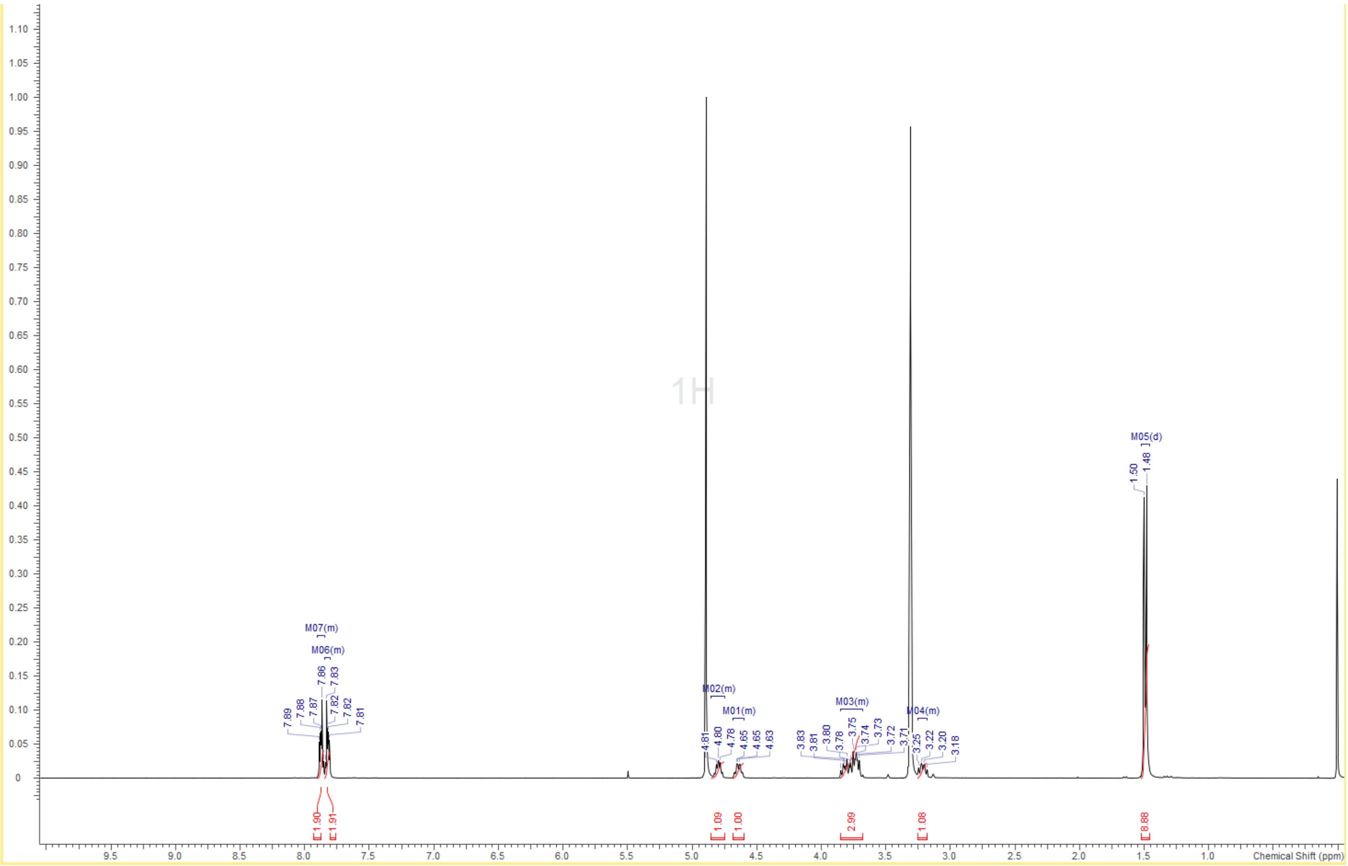

2-((3S,4S)-4-Hydroxy-1-tosylpyrrolidin-3-yl)isoindoline-1,3-dione

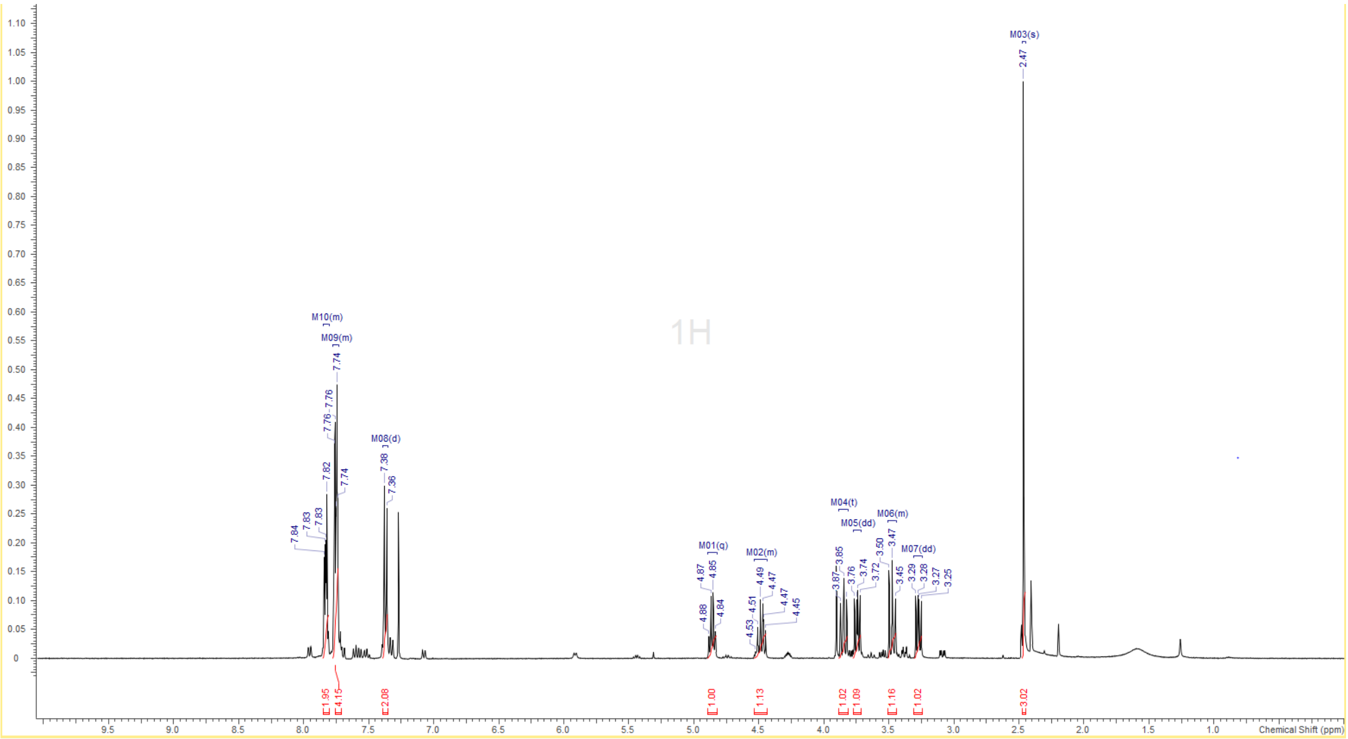

2-((3S,4S)-4-(2,2-Dimethoxyethoxy)-1-tosylpyrrolidin-3-yl)isoindoline-1,3-dione

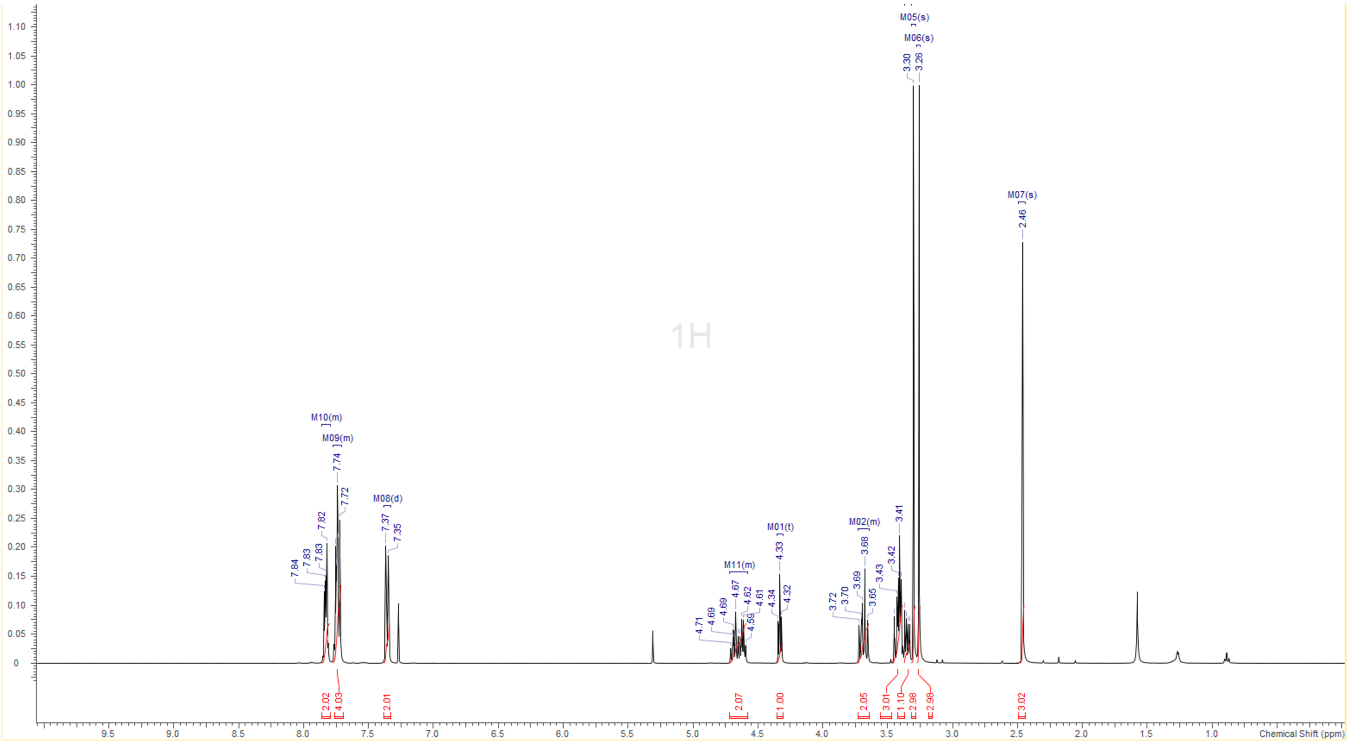

Diisopropyl 4-((((3S,4S)-4-(1,3-dioxoisoindolin-2-yl)-1-tosylpyrrolidin-3-yl)oxy)methyl)-2,6-dimethyl-1,4-dihydropyridine-3,5-dicarboxylate

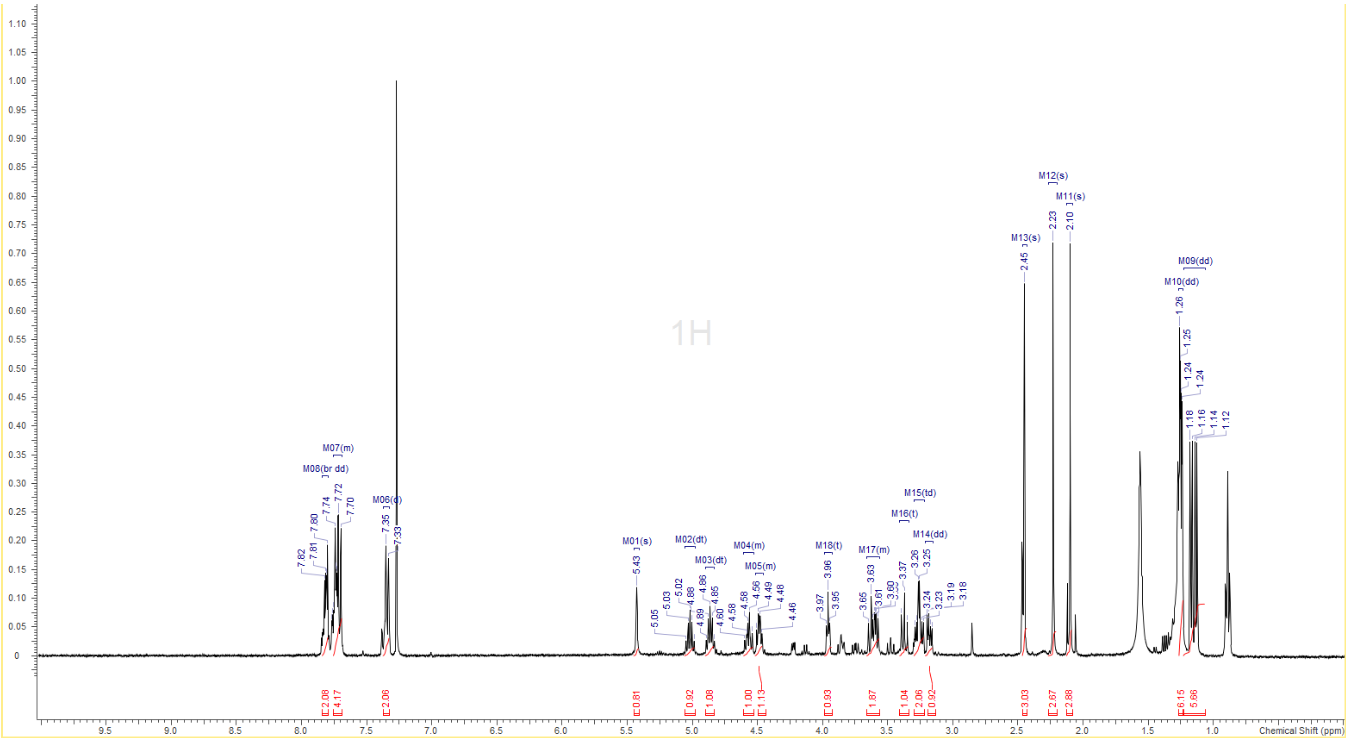

Diisopropyl 4-((((3S,4S)-4-amino-1-tosylpyrrolidin-3-yl)oxy)methyl)-2,6-dimethyl-1,4-dihydropyridine-3,5-dicarboxylate (1i)

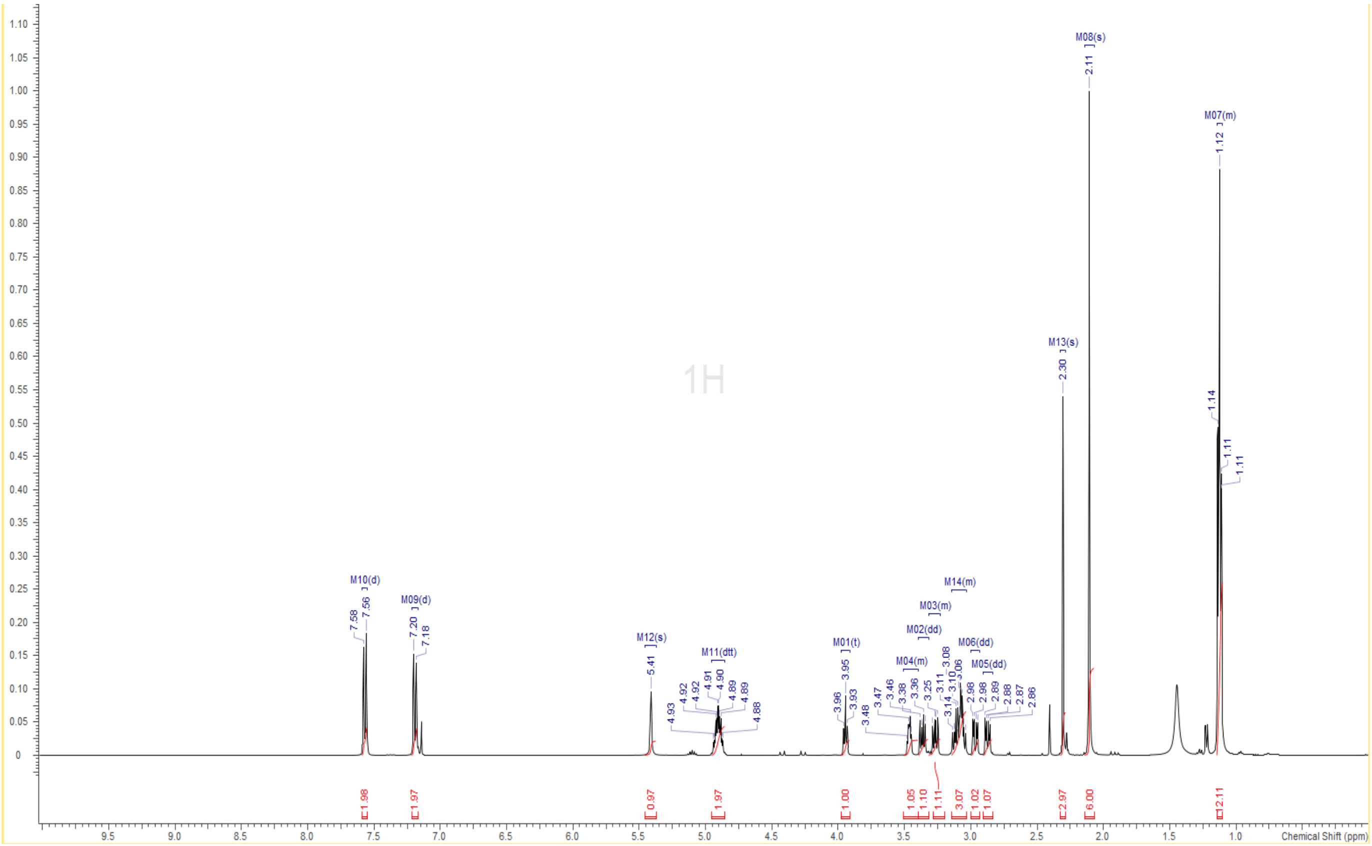
1H
M10(d)
M09(d)
M12(s)
M11(dtt)
M01(t)
M04(m)
M02(dd)
M03(m)
M14(m)
M06(dd)
M05(dd)
M13(s)
M08(s)
M07(m)
Chemical Shift (ppm)

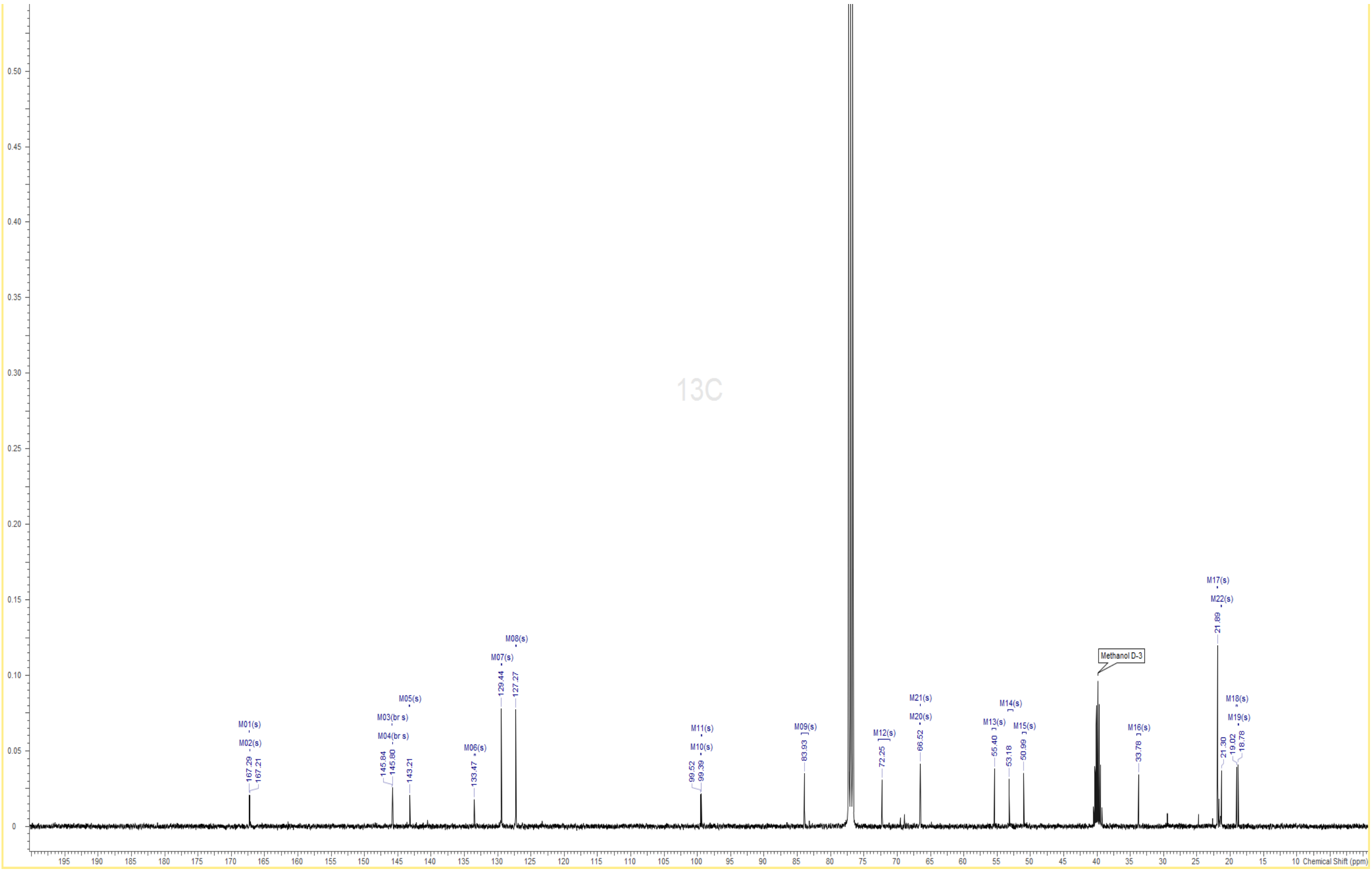
13C
Methanol D-3
Chemical Shift (ppm)

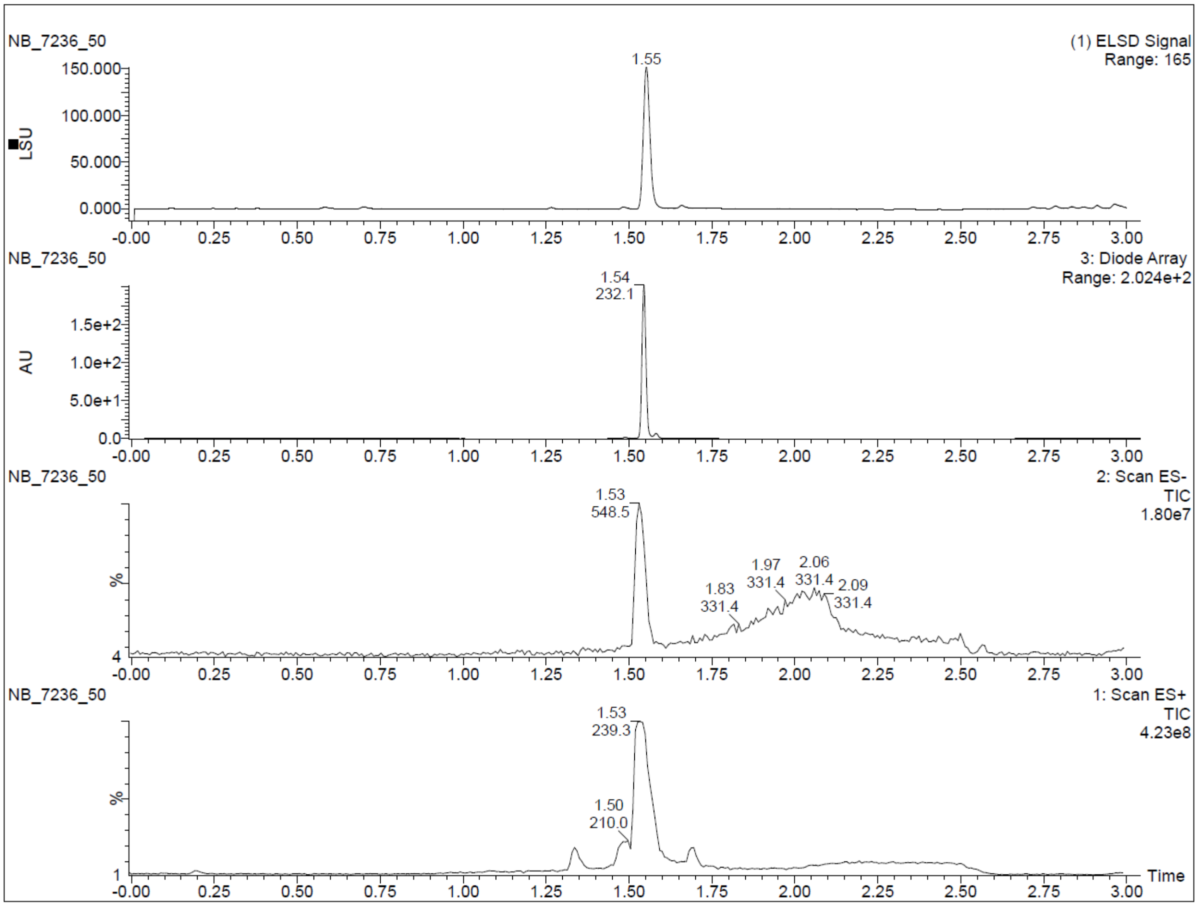
NB_7236_50
(1) ELSD Signal
Range: 165
LSU
1.55
3: Diode Array
Range: 2.024e+2
AU
1.54
232.1
2: Scan ES-
TIC
1.80e7
1.53
548.5
1.83
331.4
1.97
331.4
2.06
331.4
2.09
331.4
1: Scan ES+
TIC
4.23e8
1.53
239.3
1.50
210.0
Time

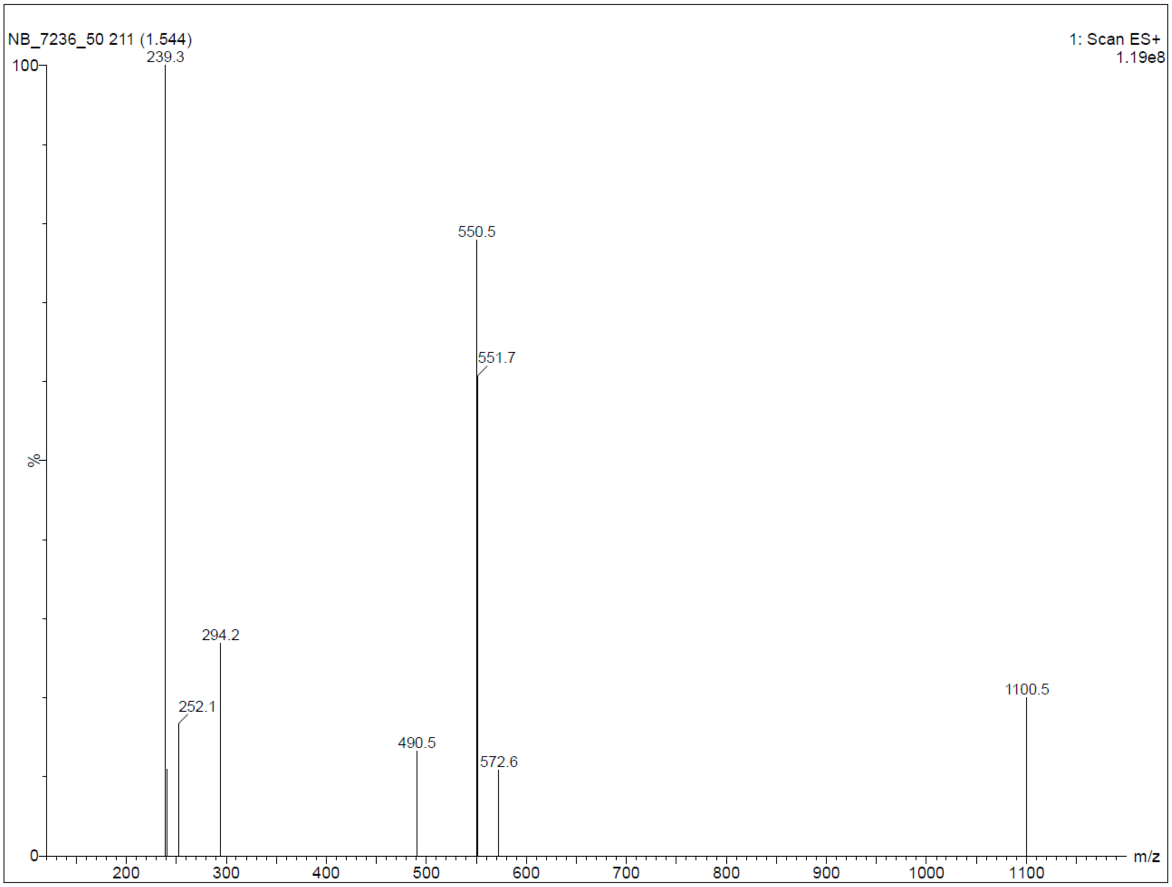
NB_7236_50 211 (1.544)
1: Scan ES+
1.19e8
239.3
550.5
551.7
294.2
252.1
490.5
572.6
1100.5
m/z

N,N-Dibenzyl-3-(2,2-dimethoxyethoxy)propan-1-amine

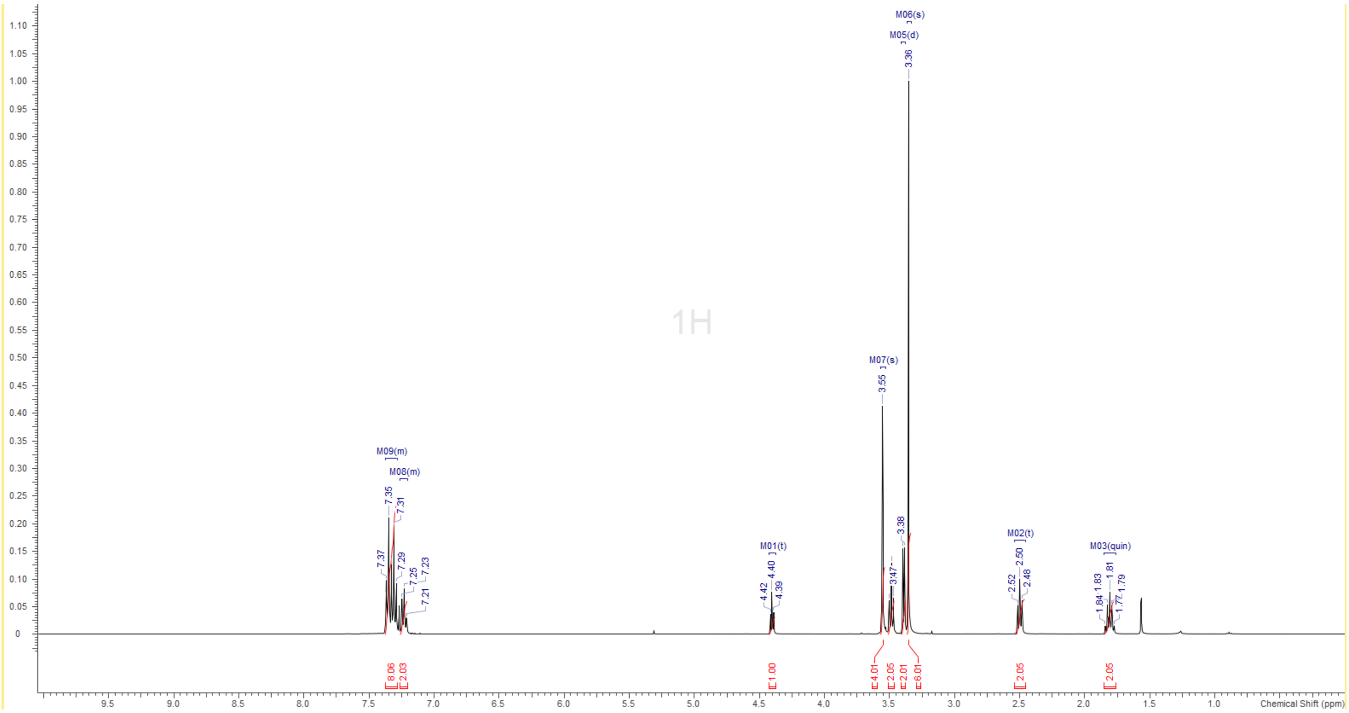

2-(3-(Dibenzylamino)propoxy)acetaldehyde

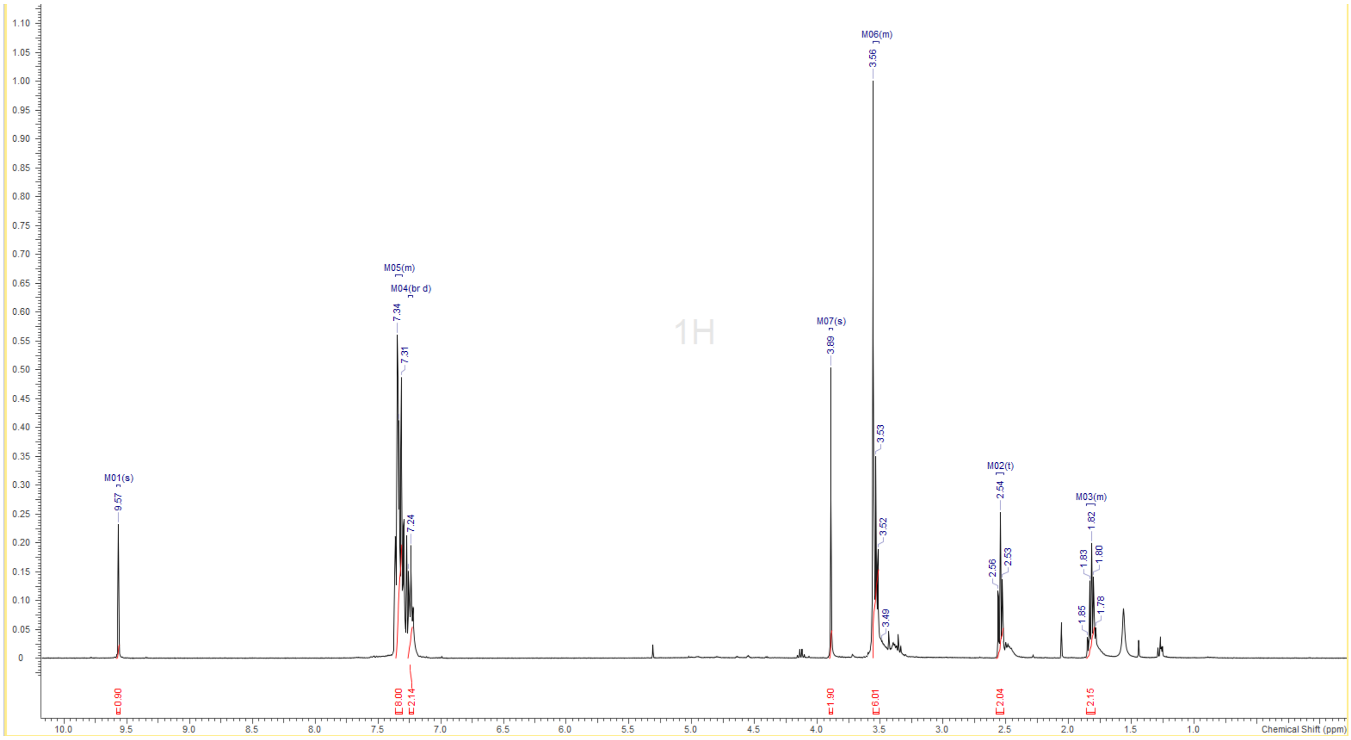

Diisopropyl 4-((3-(dibenzylamino)propoxy)methyl)-2,6-dimethyl-1,4-dihydropyridine-3,5-dicarboxylate

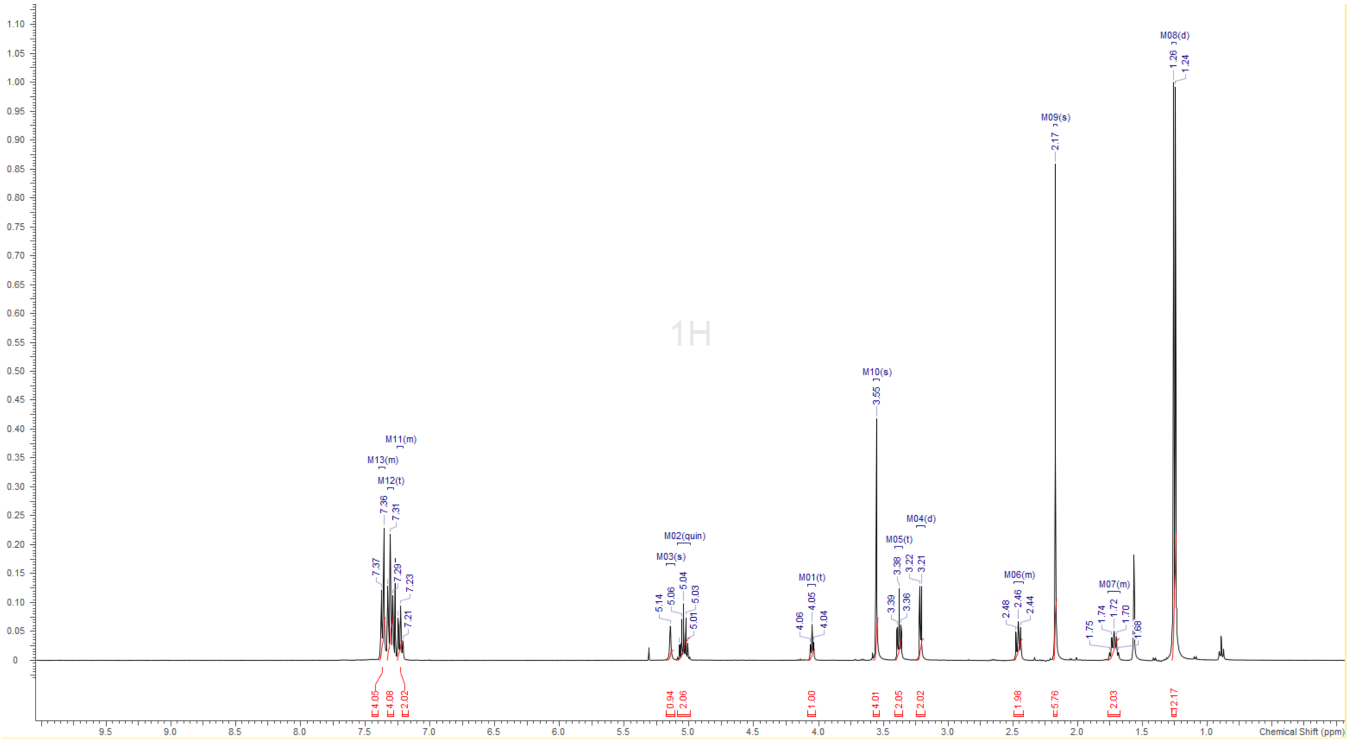

Diisopropyl 4-((3-aminopropoxy)methyl)-2,6-dimethyl-1,4-dihydropyridine-3,5-dicarboxylate (1q)

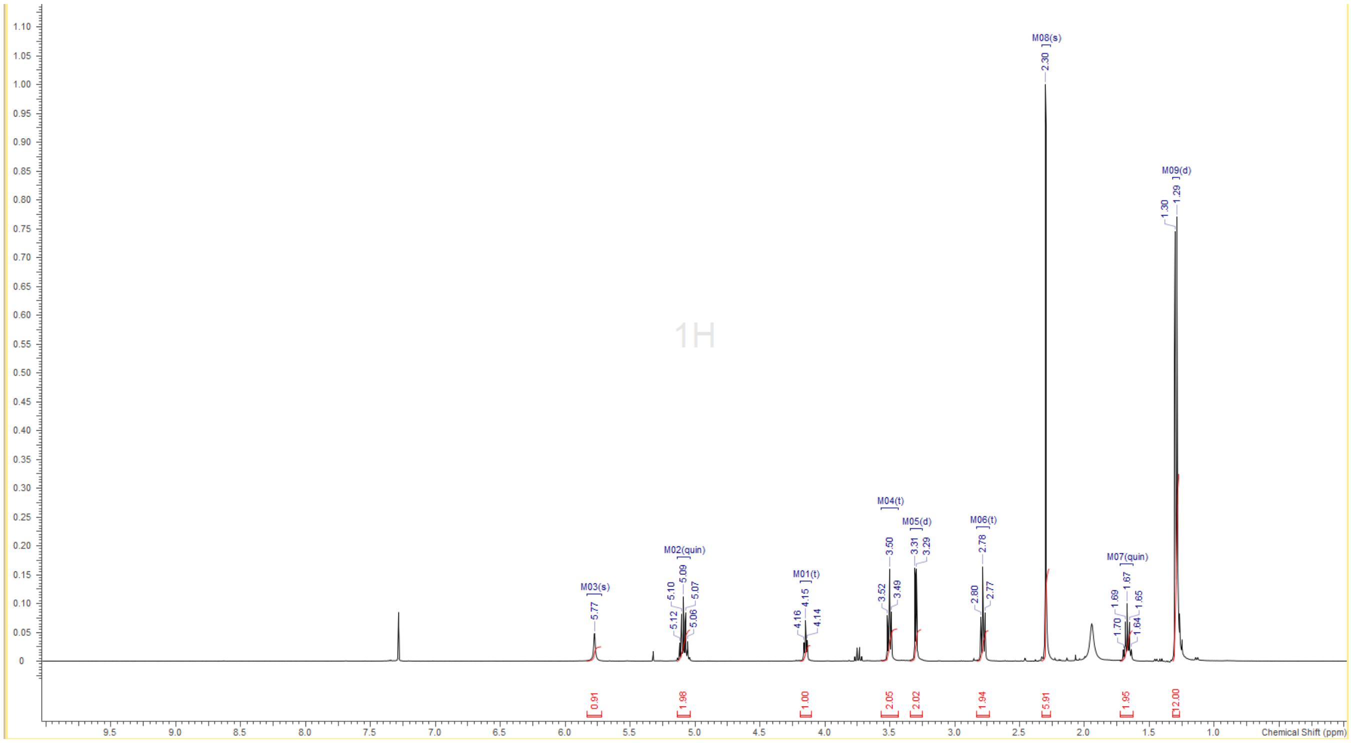

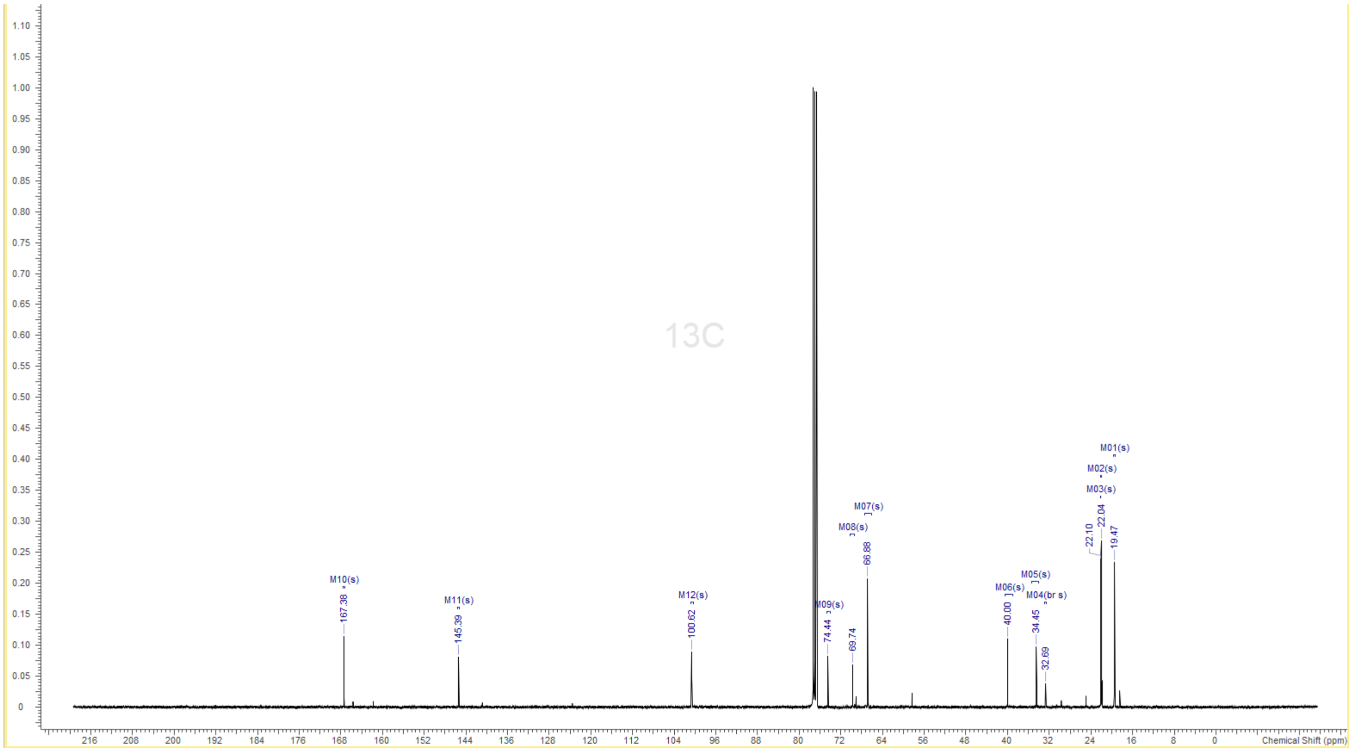
13C
M01(s)
M02(s)
M03(s)
M04(br s)
M05(s)
M06(s)
M07(s)
M08(s)
M09(s)
M10(s)
M11(s)
M12(s)
167.38
145.39
100.62
74.44
69.74
66.88
40.00
34.45
32.69
22.10
22.04
19.47
Chemical Shift (ppm)

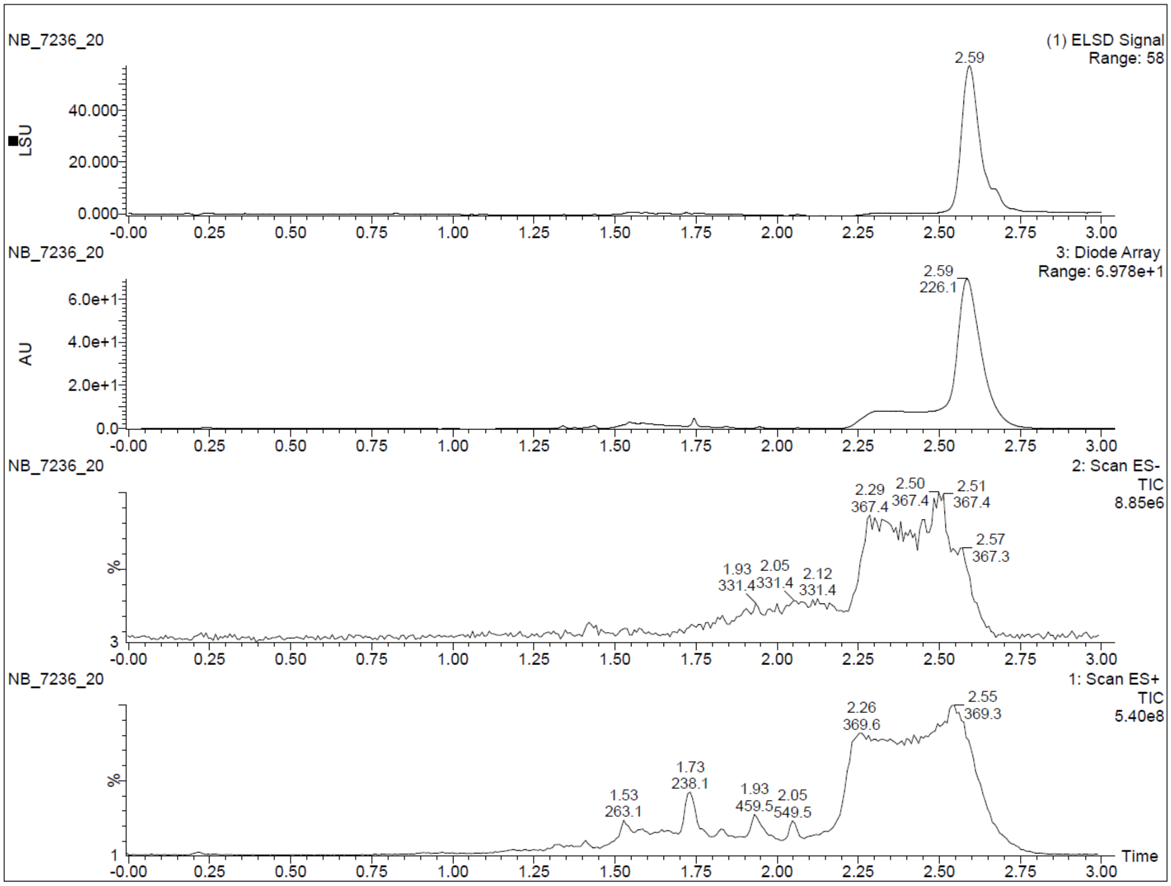
NB_7236_20
(1) ELSD Signal
Range: 58
LSU
2.59
NB_7236_20
3: Diode Array
Range: 6.978e+1
AU
2.59
226.1
NB_7236_20
2: Scan ES-
TIC
8.85e6
2.29 367.4
2.50 367.4
2.51 367.4
2.57 367.3
1.93 331.4
2.05 331.4
2.12 331.4
NB_7236_20
1: Scan ES+
TIC
5.40e8
2.26 369.6
2.55 369.3
1.53 263.1
1.73 238.1
1.93 459.5
2.05 549.5
Time

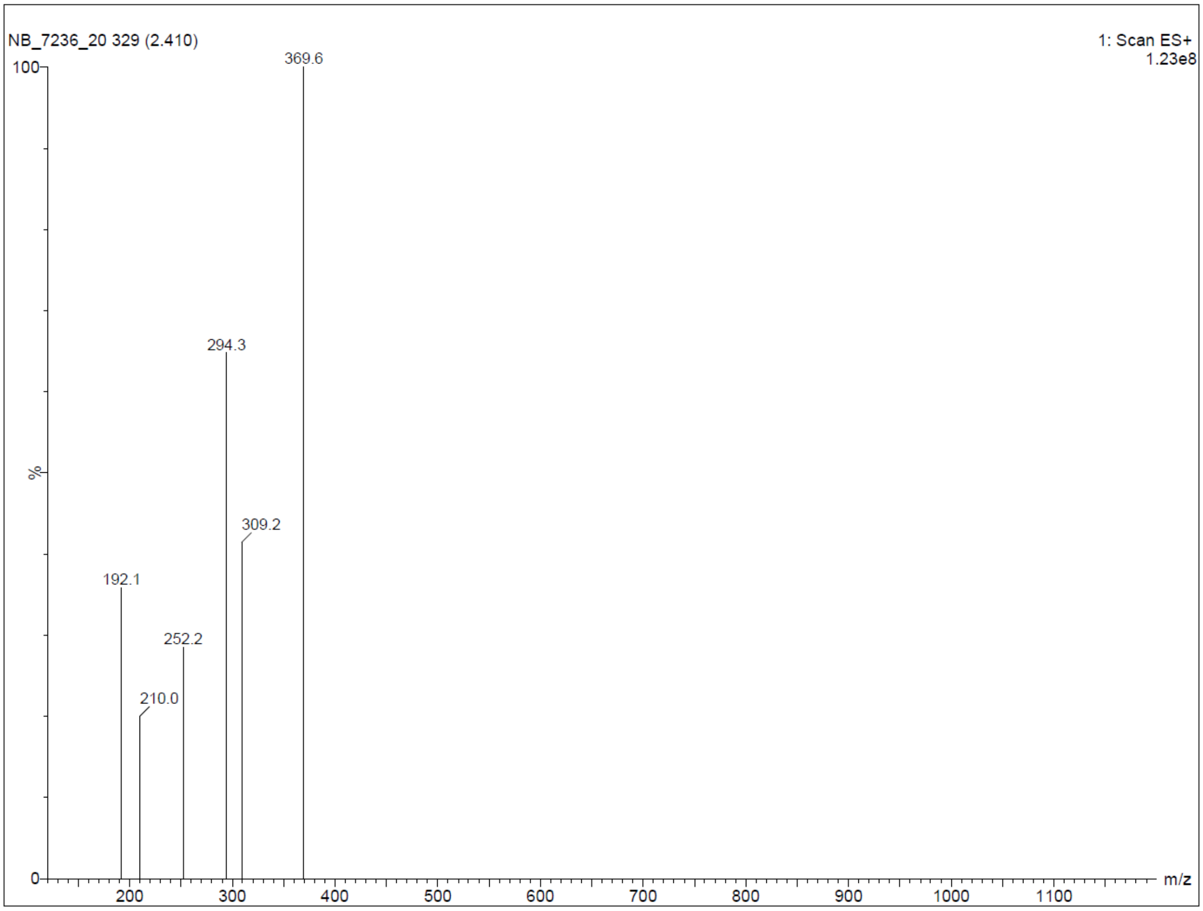

Thiomorpholine-Forming Amino-DHP Reagents

2-(2-Mercaptoethyl)isoindoline-1,3-dione

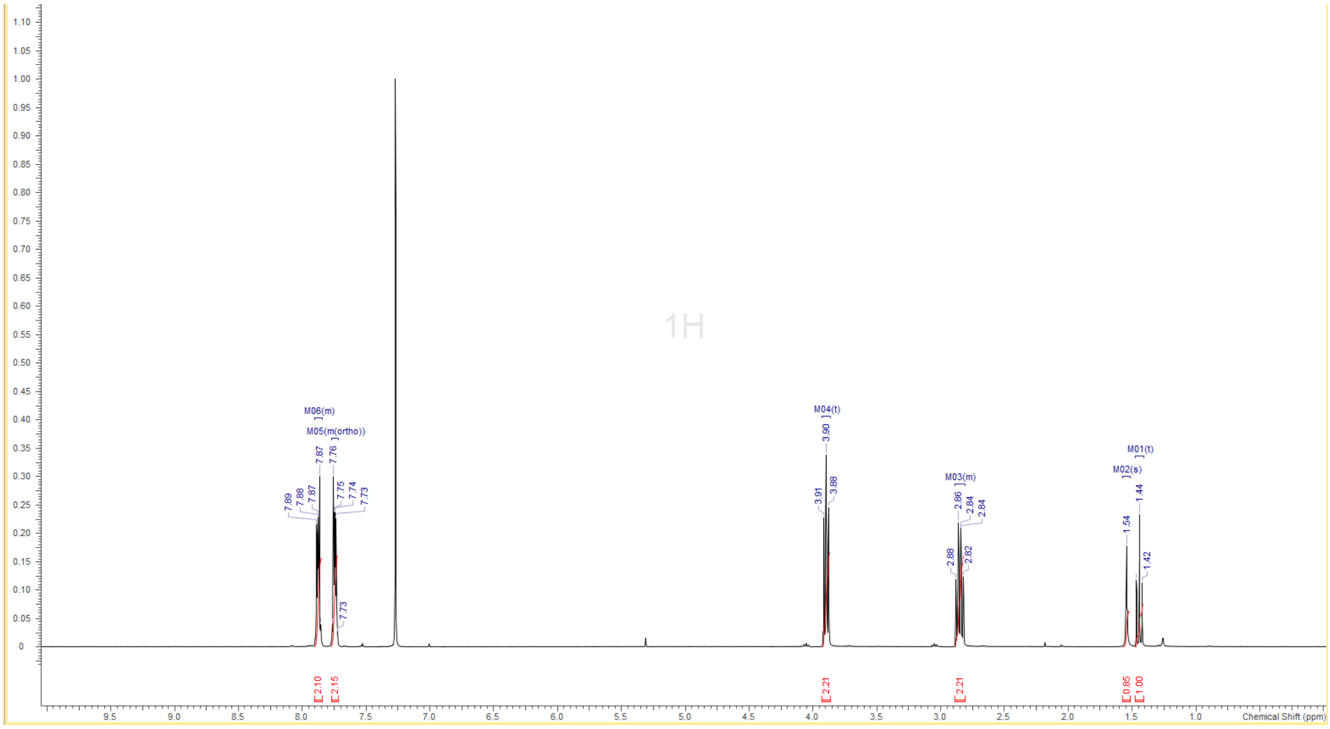

2-(2-((2,2-Dimethoxyethyl)thio)ethyl)isoindoline-1,3-dione

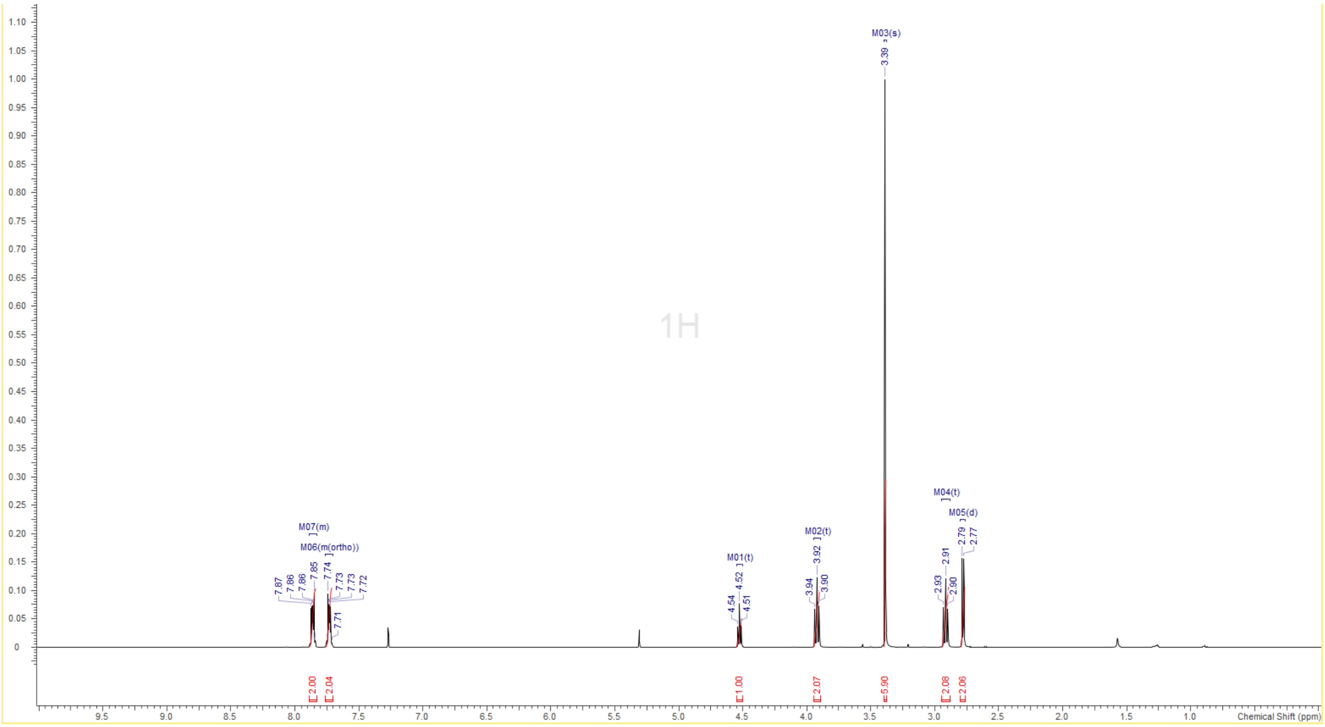

2-((2-(1,3-Dioxoisoindolin-2-yl)ethyl)thio)acetaldehyde

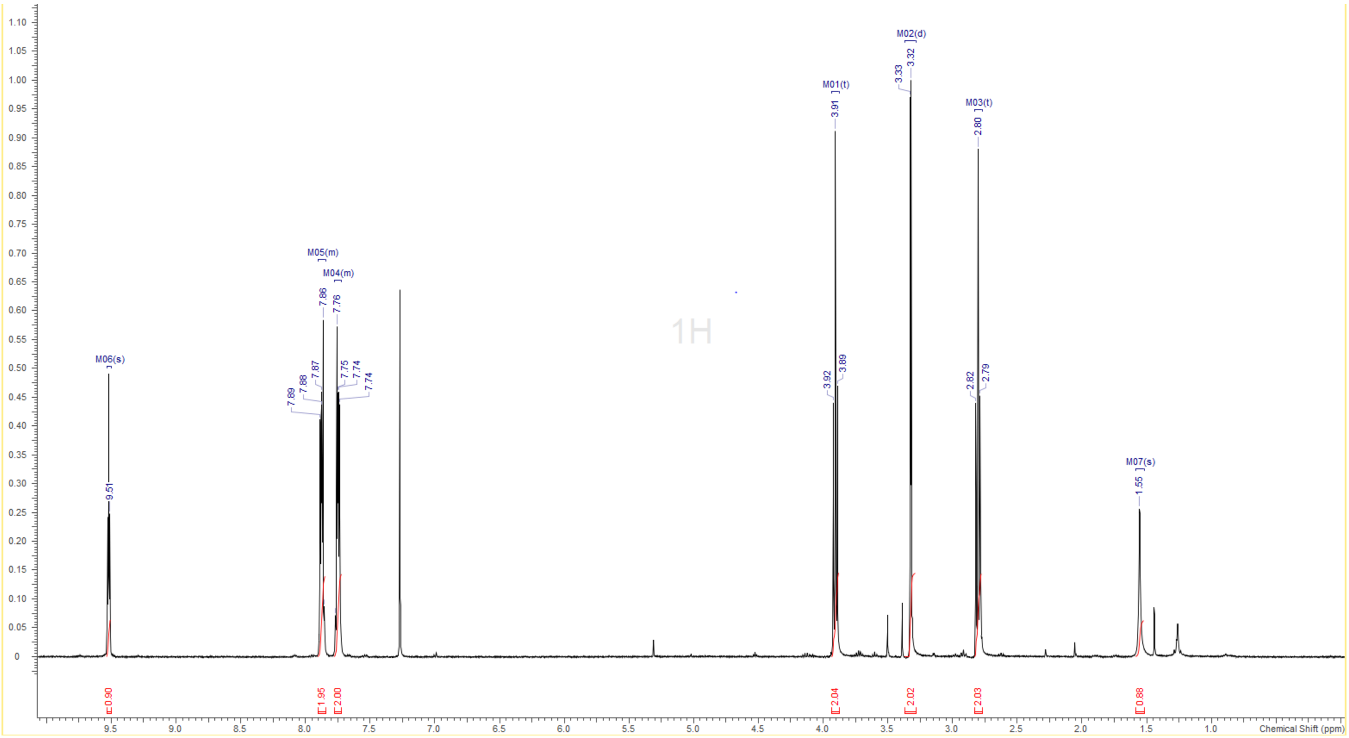

Diisopropyl 4-(((2-(1,3-dioxoisoindolin-2-yl)ethyl)thio)methyl)-2,6-dimethyl-1,4-dihydropyridine-3,5-dicarboxylate

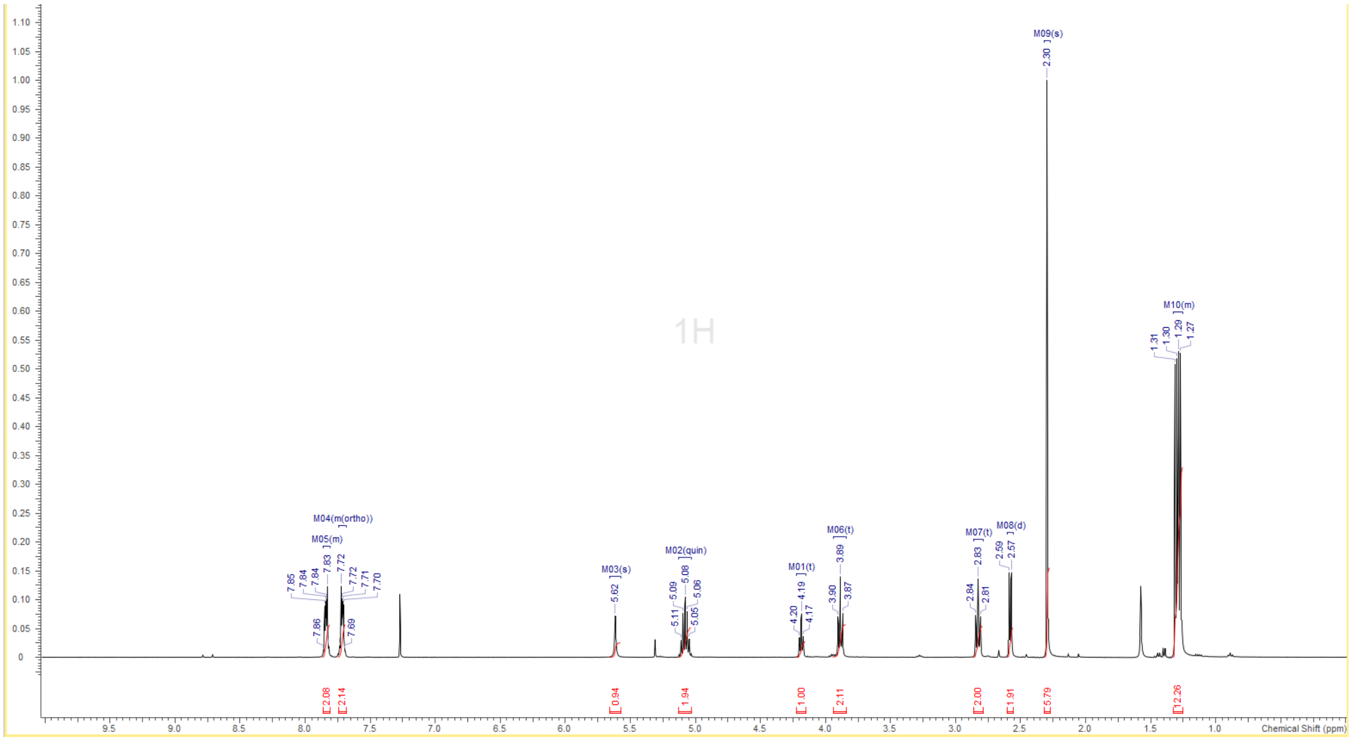

Diisopropyl 4-(((2-aminoethyl)thio)methyl)-2,6-dimethyl-1,4-dihydropyridine-3,5-dicarboxylate (1j)

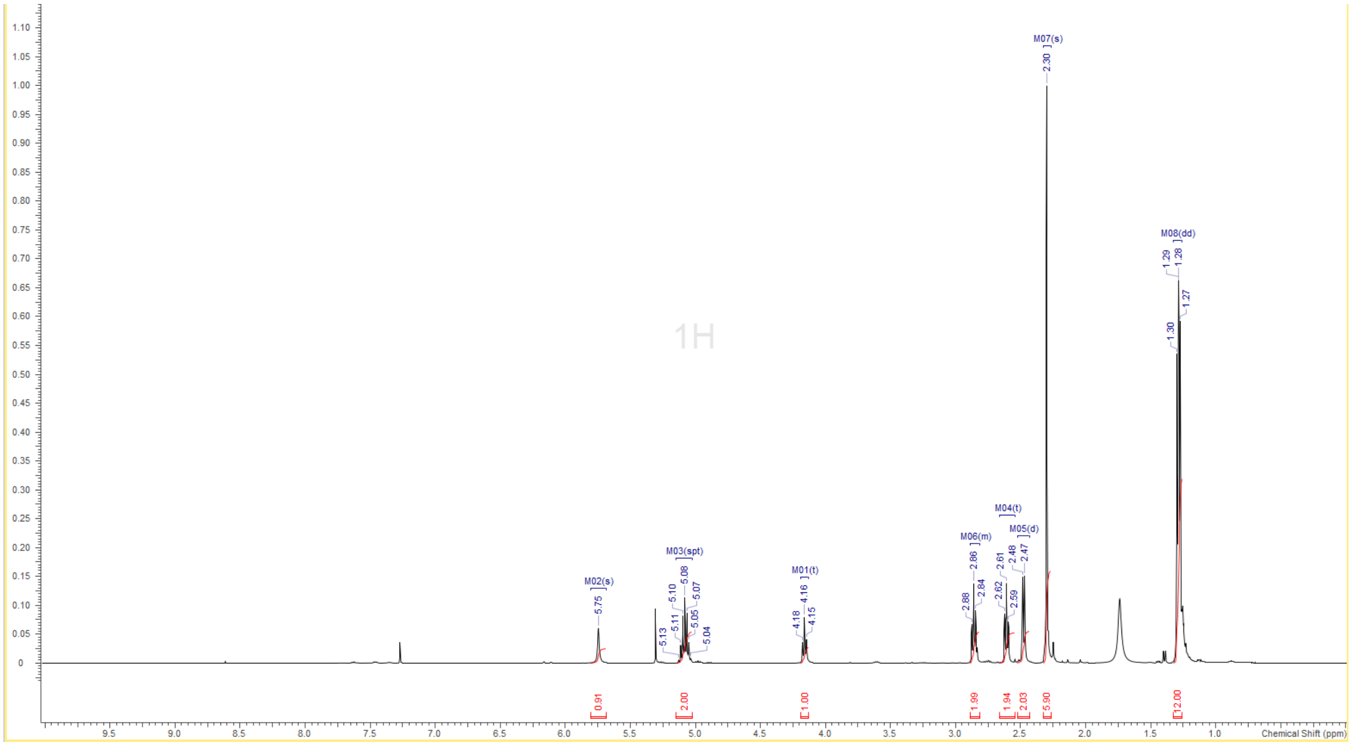

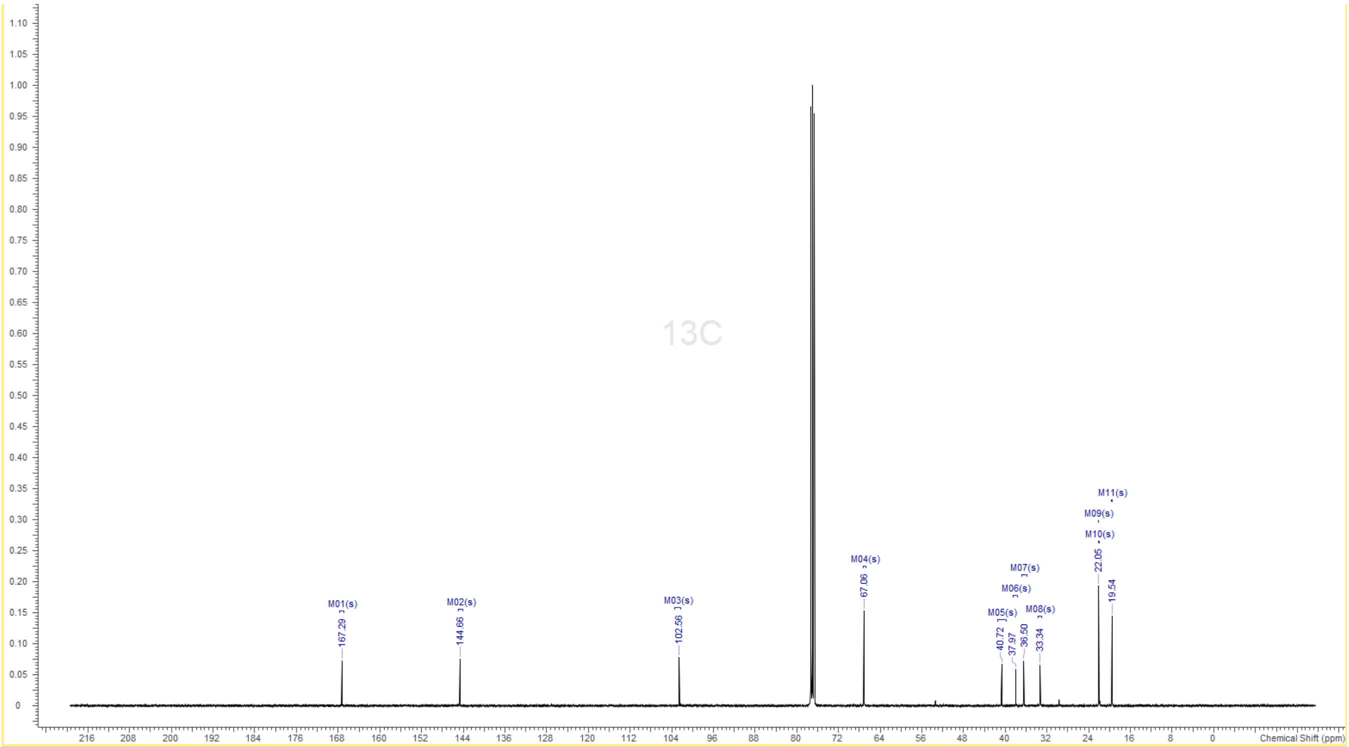
13C
M01(s)
167.29
M02(s)
144.66
M03(s)
102.56
M04(s)
67.06
M05(s)
40.72
M06(s)
37.97
M07(s)
36.50
M08(s)
33.34
M09(s)
M10(s)
22.05
M11(s)
19.54
Chemical Shift (ppm)

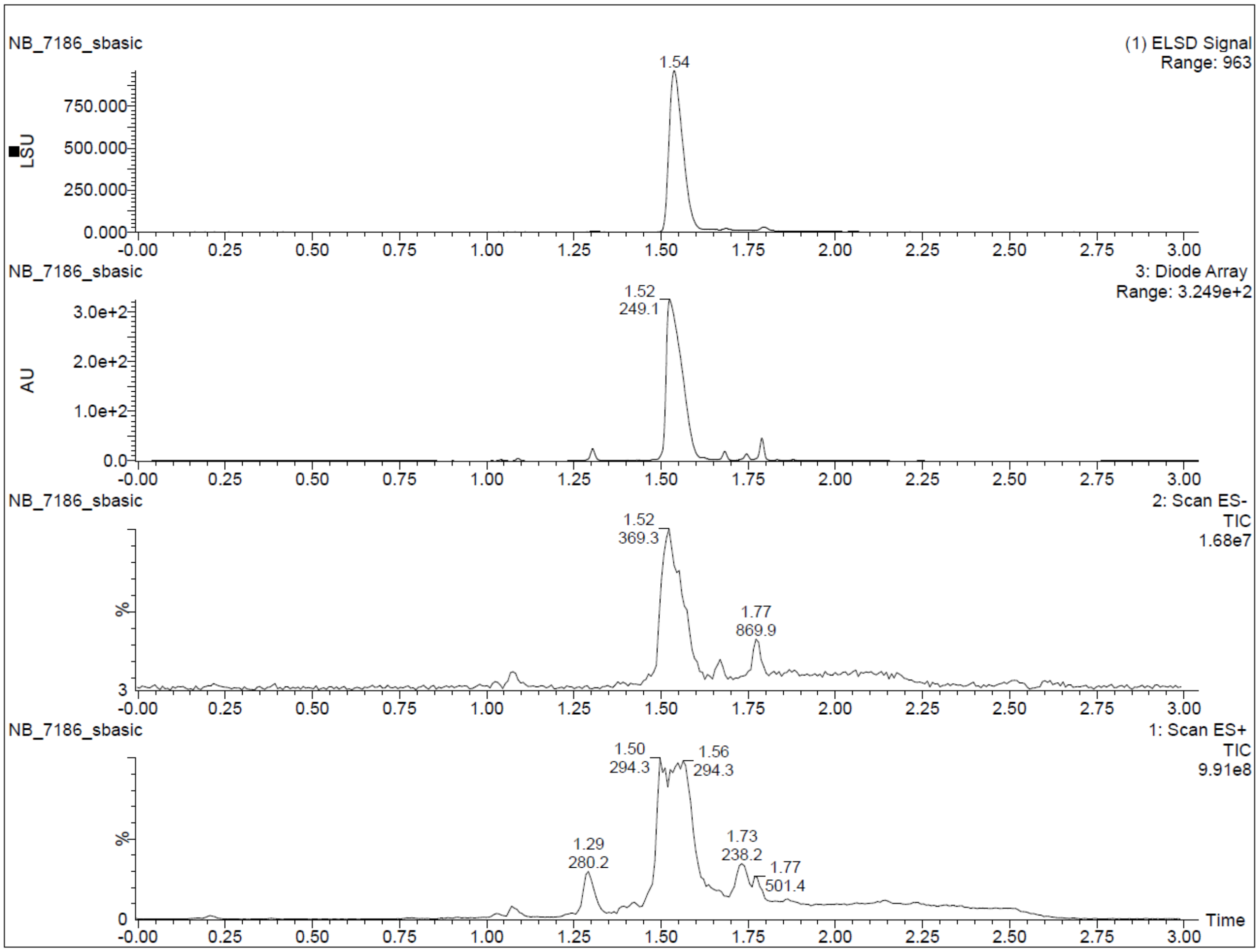
NB_7186_sbasic
(1) ELSD Signal
Range: 963
1.54
LSU
NB_7186_sbasic
3: Diode Array
Range: 3.249e+2
1.52
249.1
AU
NB_7186_sbasic
2: Scan ES-
TIC
1.68e7
1.52
369.3
1.77
869.9
NB_7186_sbasic
1: Scan ES+
TIC
9.91e8
1.50
294.3
1.56
294.3
1.29
280.2
1.73
238.2
1.77
501.4
Time

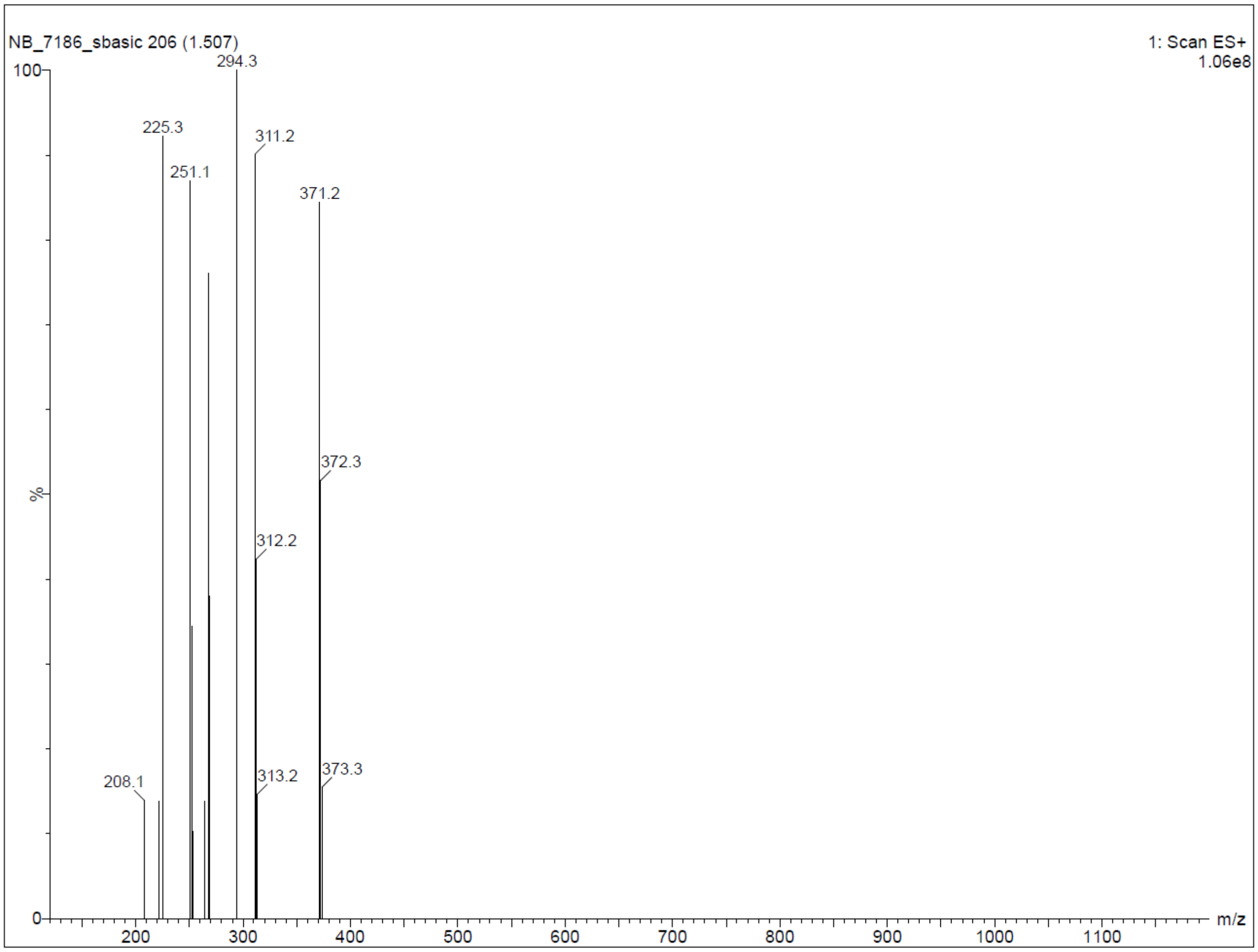

2-(2-((2,2-Dimethoxyethyl)thio)propyl)isoindoline-1,3-dione

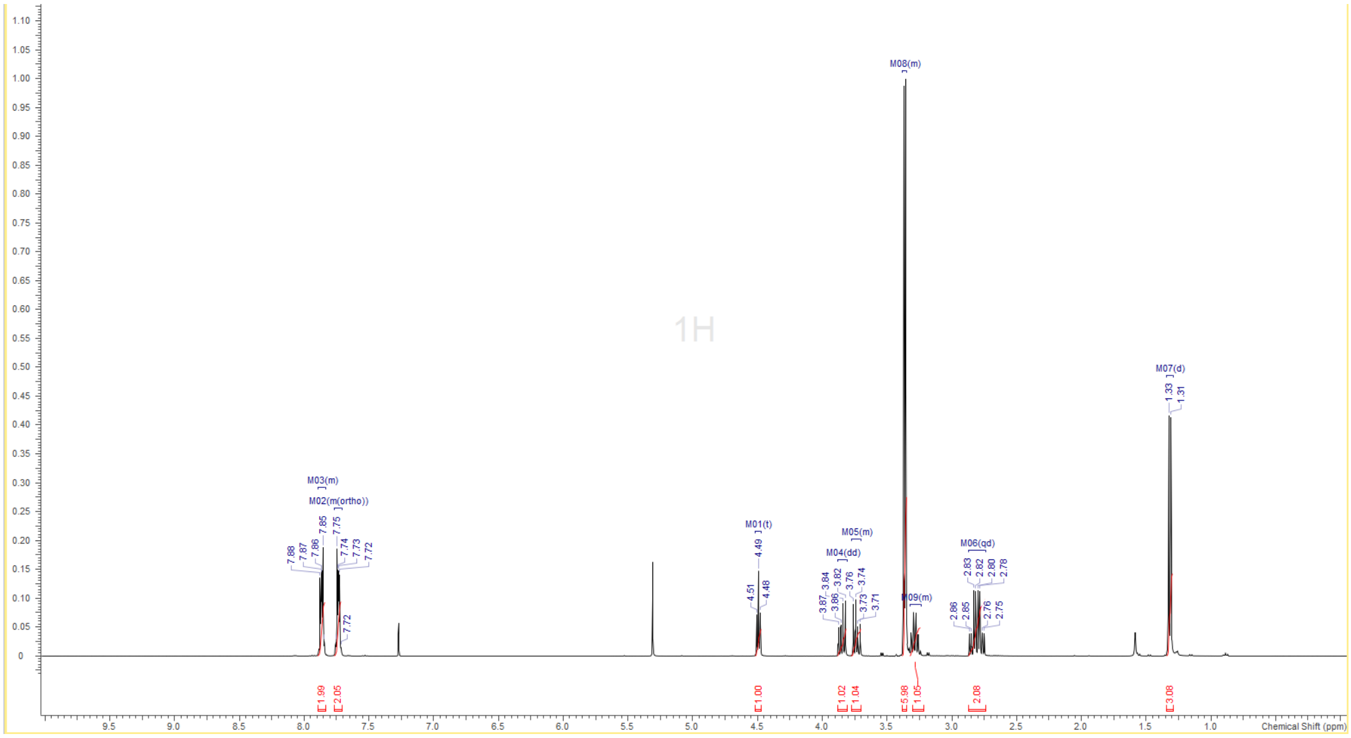

2-((2-(1,3-Dioxoisoindolin-2-yl)propyl)thio)acetaldehyde

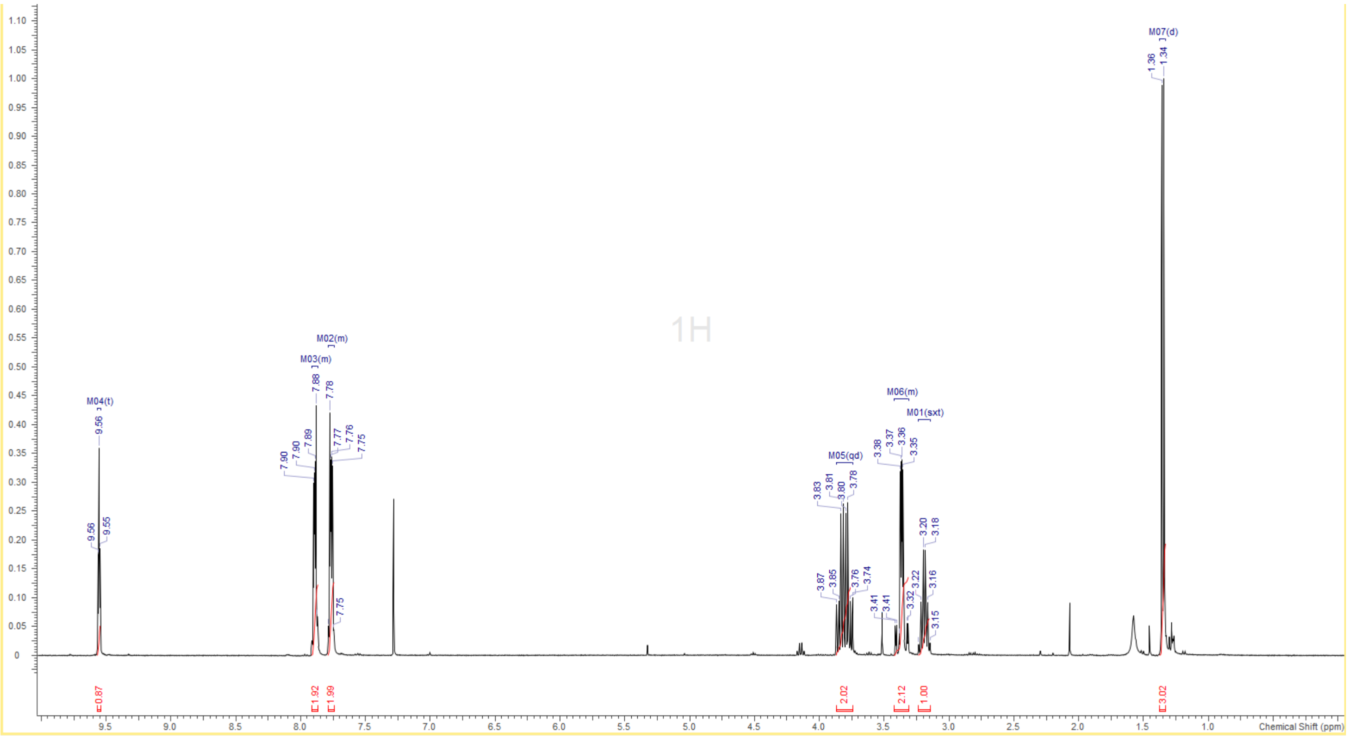

Diisopropyl 4-(((1-(1,3-dioxoisoindolin-2-yl)propan-2-yl)thio)methyl)-2,6-dimethyl-1,4-dihydropyridine-3,5-dicarboxylate

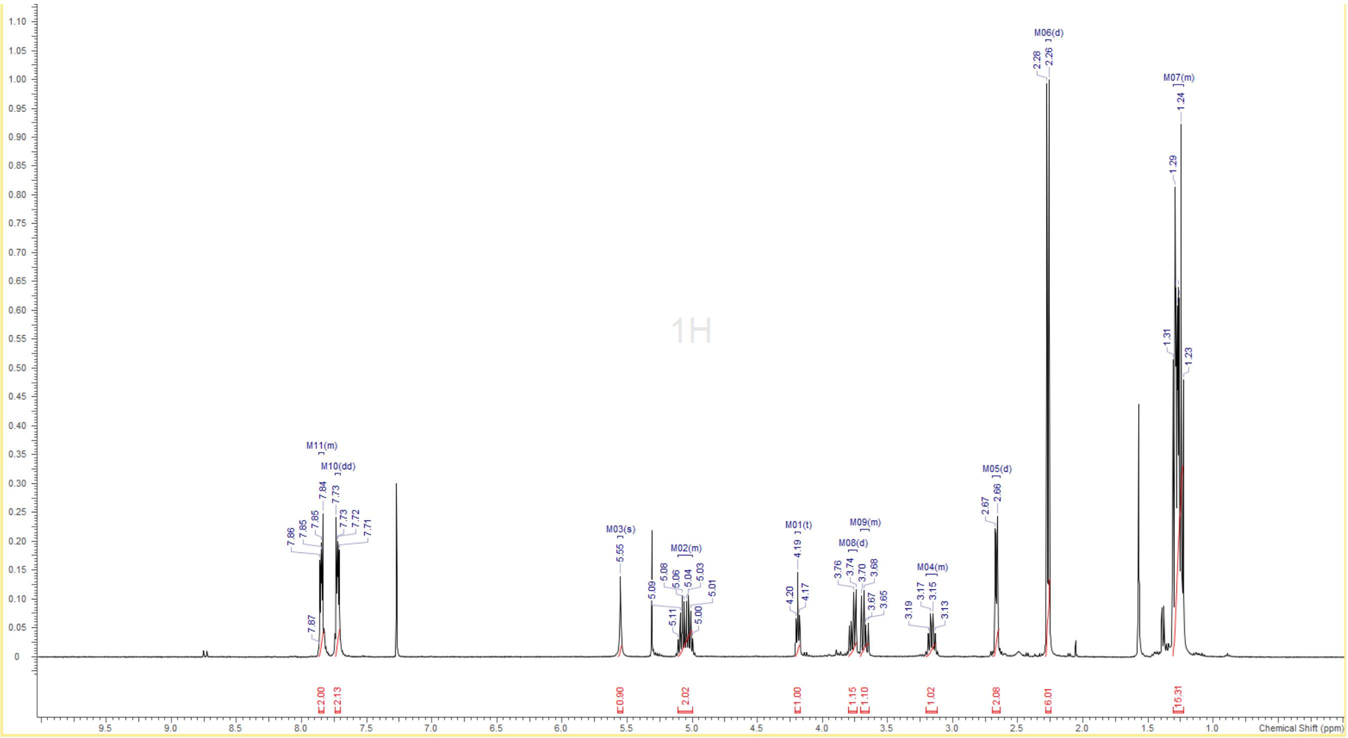

Diisopropyl 4-(((1-aminopropan-2-yl)thio)methyl)-2,6-dimethyl-1,4-dihydropyridine-3,5-dicarboxylate (1k)

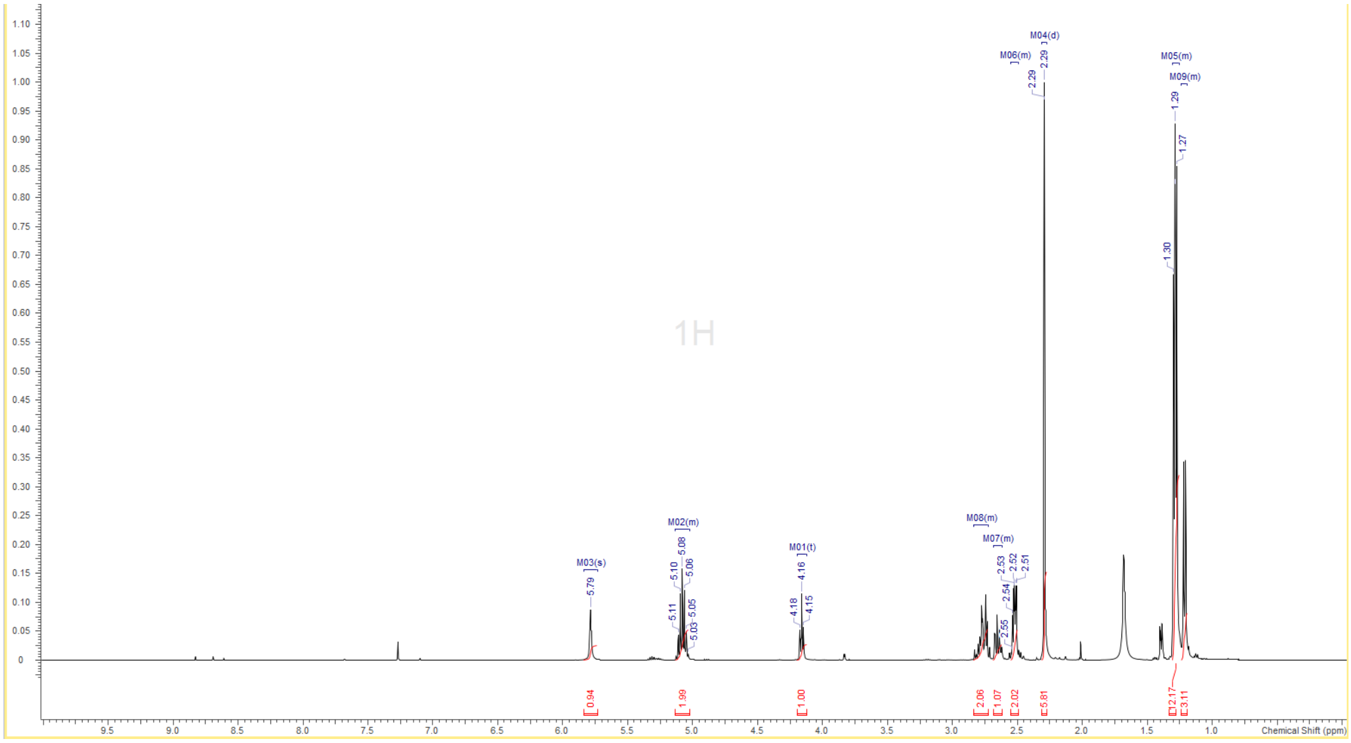
1H
M03(s)
M02(m)
M01(t)
M08(m)
M07(m)
M06(m)
M04(d)
M05(m)
M09(m)
Chemical Shift (ppm)

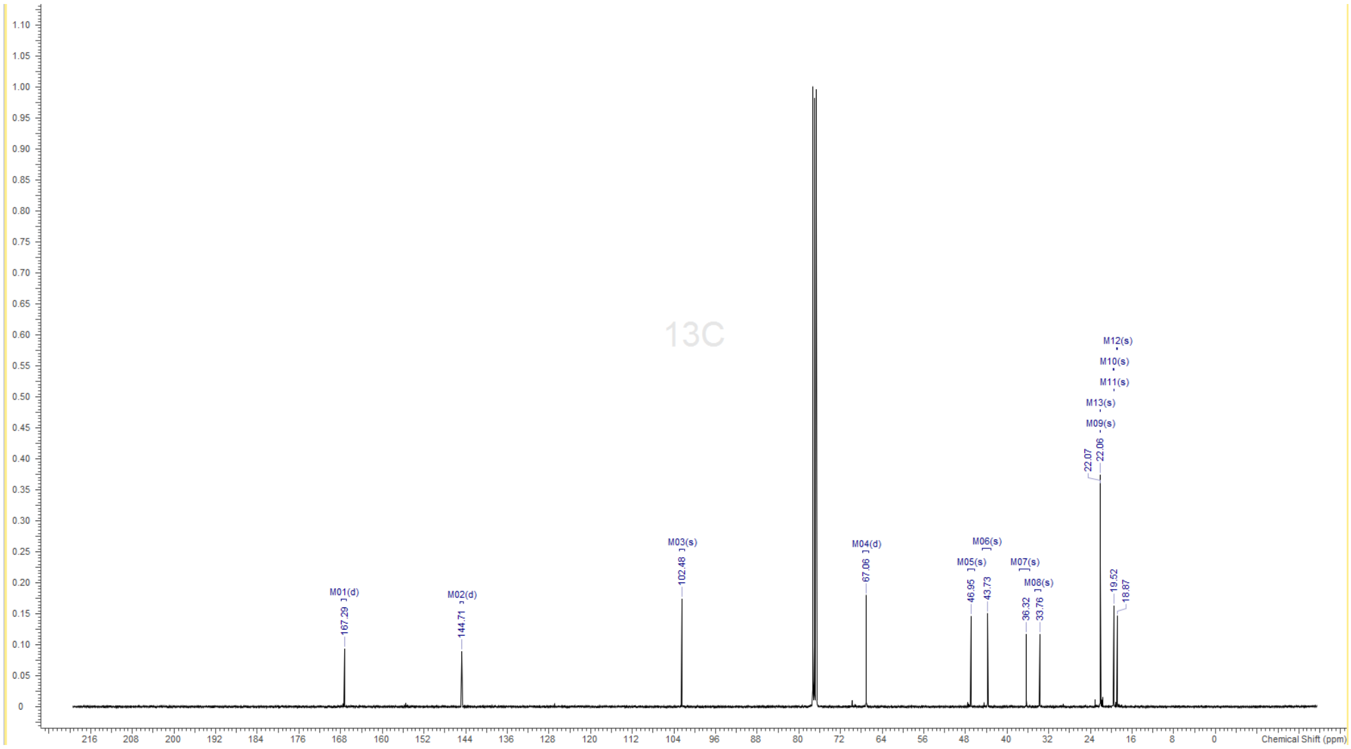
13C
M01(d)
167.29
M02(d)
144.71
M03(s)
102.48
M04(d)
67.06
M05(s)
46.95
M06(s)
43.73
M07(s)
36.32
M08(s)
33.76
22.07
22.06
19.52
18.87
Chemical Shift (ppm)

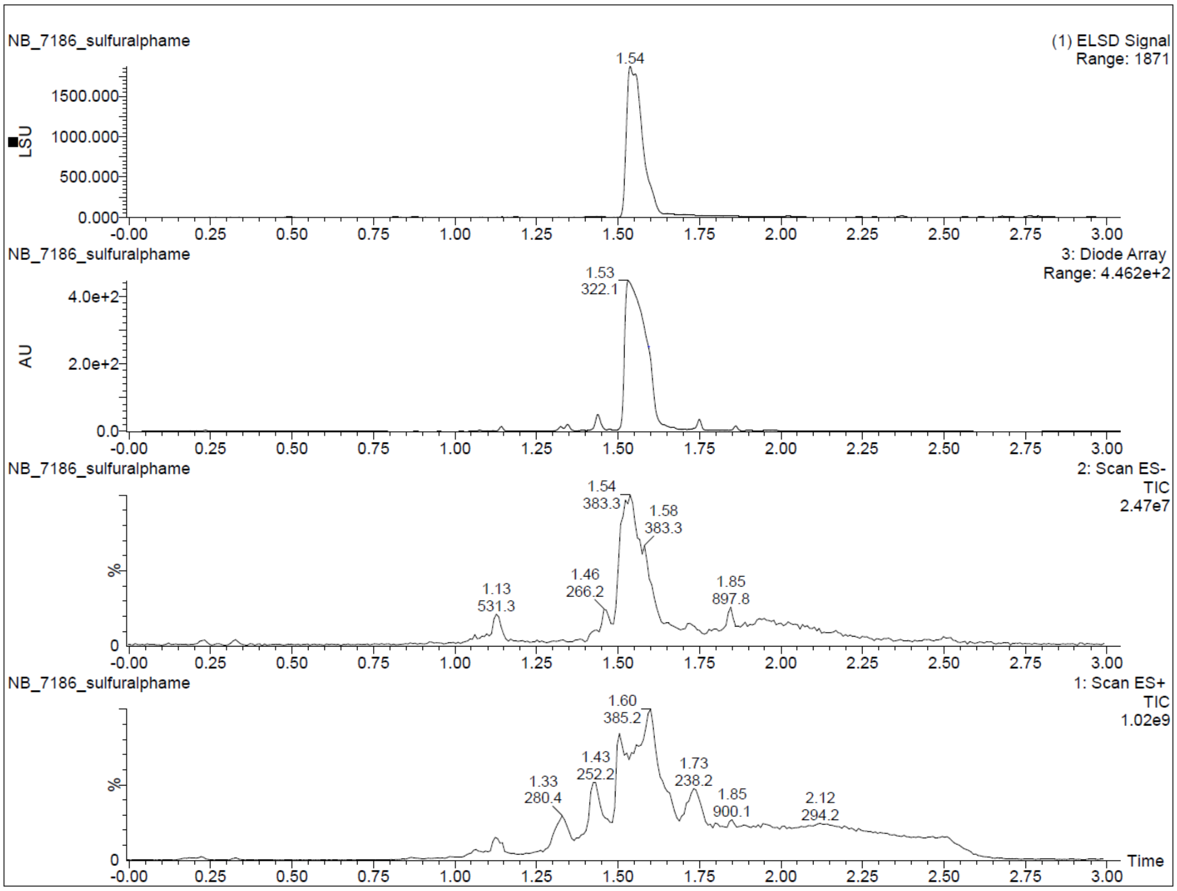
NB_7186_sulfuralphame
(1) ELSD Signal
Range: 1871
1.54
LSU
3: Diode Array
Range: 4.462e+2
1.53
322.1
AU
2: Scan ES-
TIC
2.47e7
1.54
383.3
1.58
383.3
1.46
266.2
1.13
531.3
1.85
897.8
1: Scan ES+
TIC
1.02e9
1.60
385.2
1.43
252.2
1.33
280.4
1.73
238.2
1.85
900.1
2.12
294.2
Time

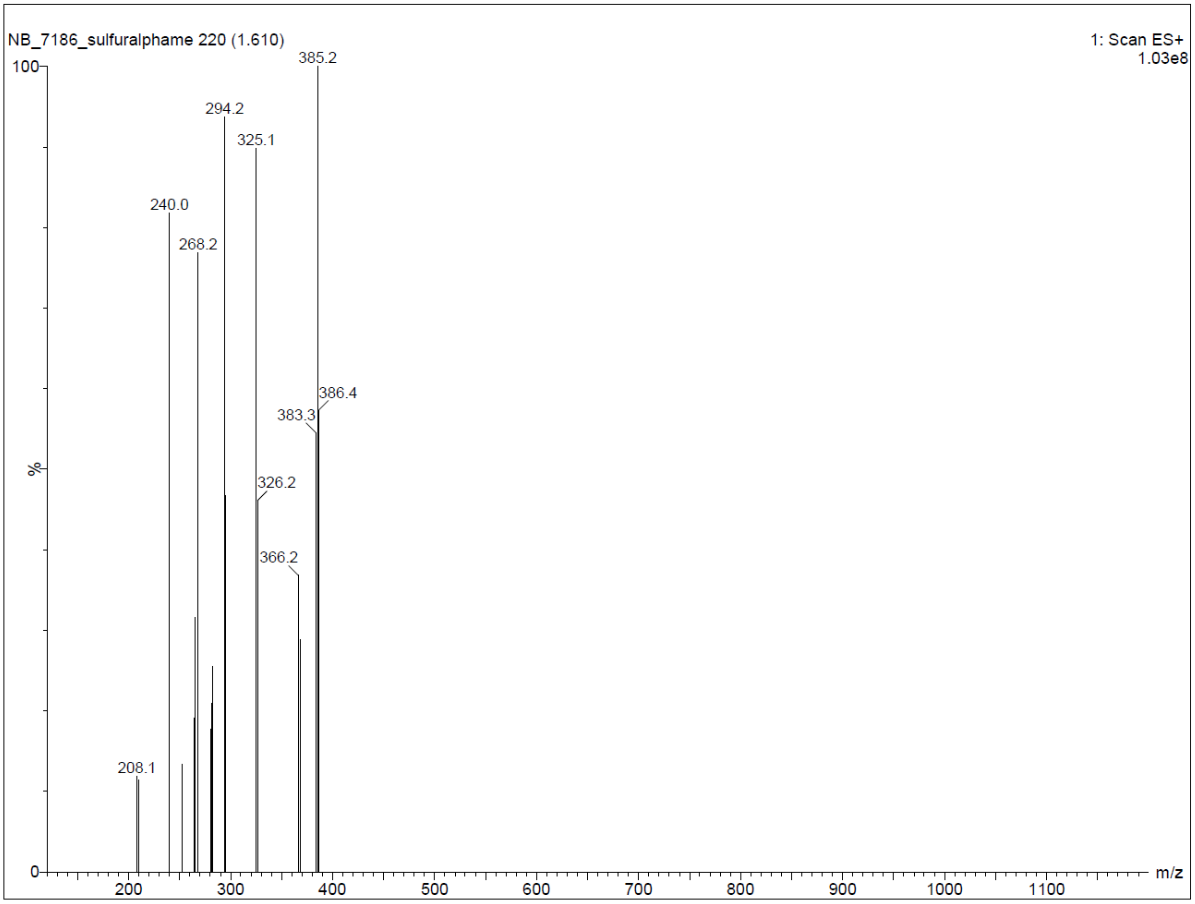
NB_7186_sulfuralphame 220 (1.610)
1: Scan ES+
1.03e8
385.2
294.2
325.1
240.0
268.2
386.4
383.3
326.2
366.2
208.1
%
m/z

2-(1-Mercaptopropan-2-yl)isoindoline-1,3-dione

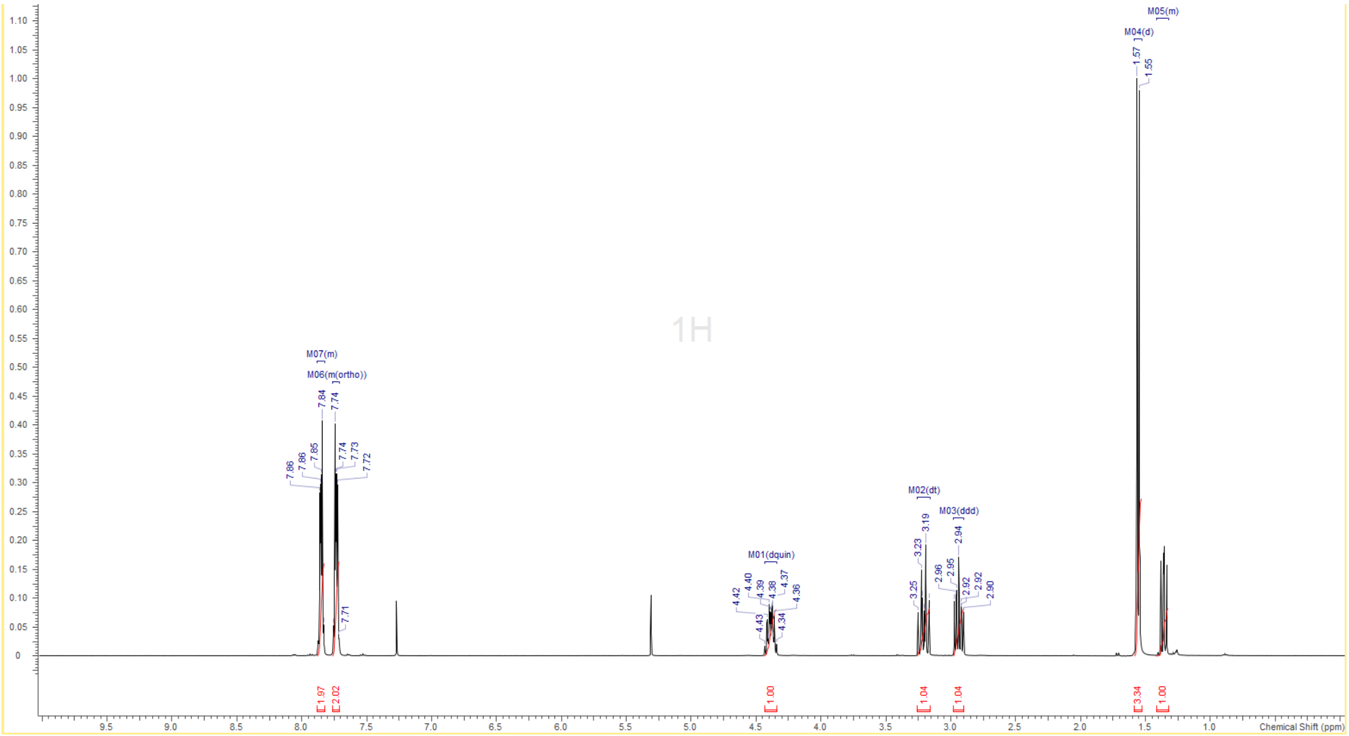

2-(1-((2,2-Dimethoxyethyl)thio)propan-2-yl)isoindoline-1,3-dione

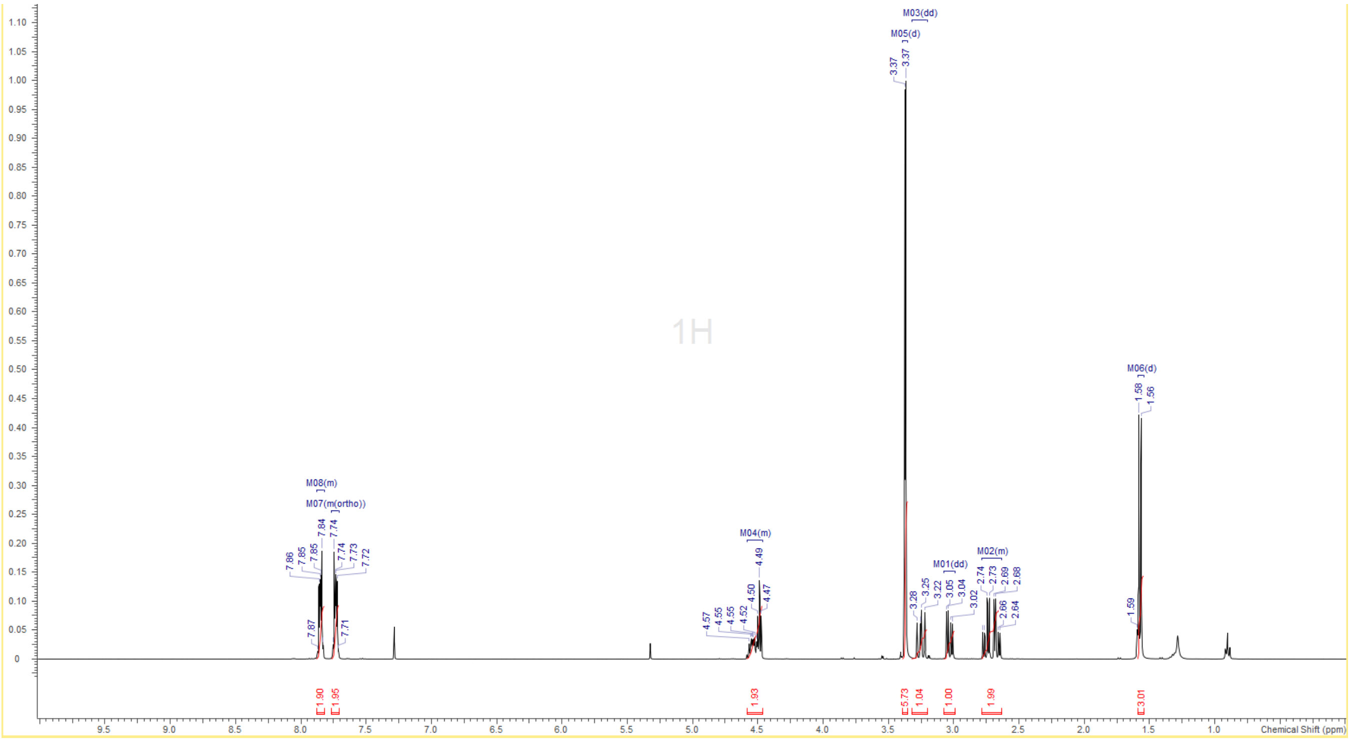

2-((2-(1,3-Dioxoisoindolin-2-yl)propyl)thio)acetaldehyde

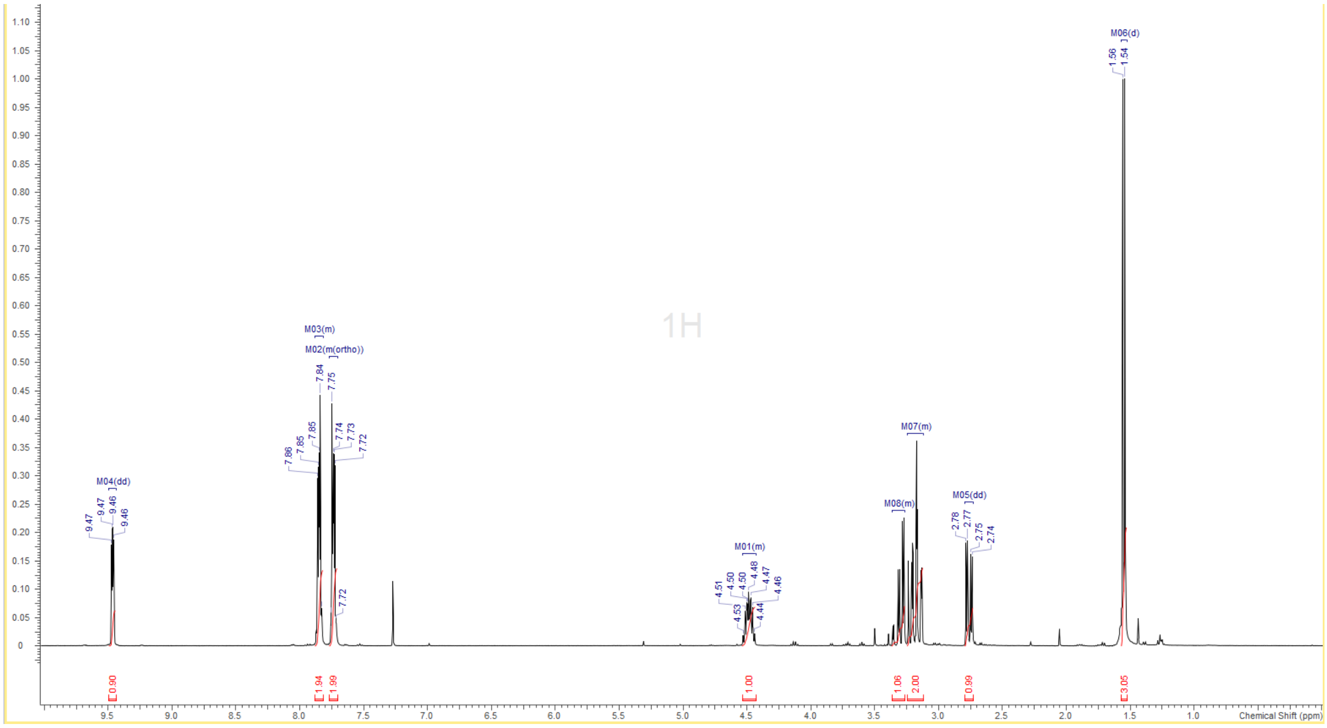

Diisopropyl 4-(((2-(1,3-dioxoisoindolin-2-yl)propyl)thio)methyl)-2,6-dimethyl-1,4-dihydropyridine-3,5-dicarboxylate

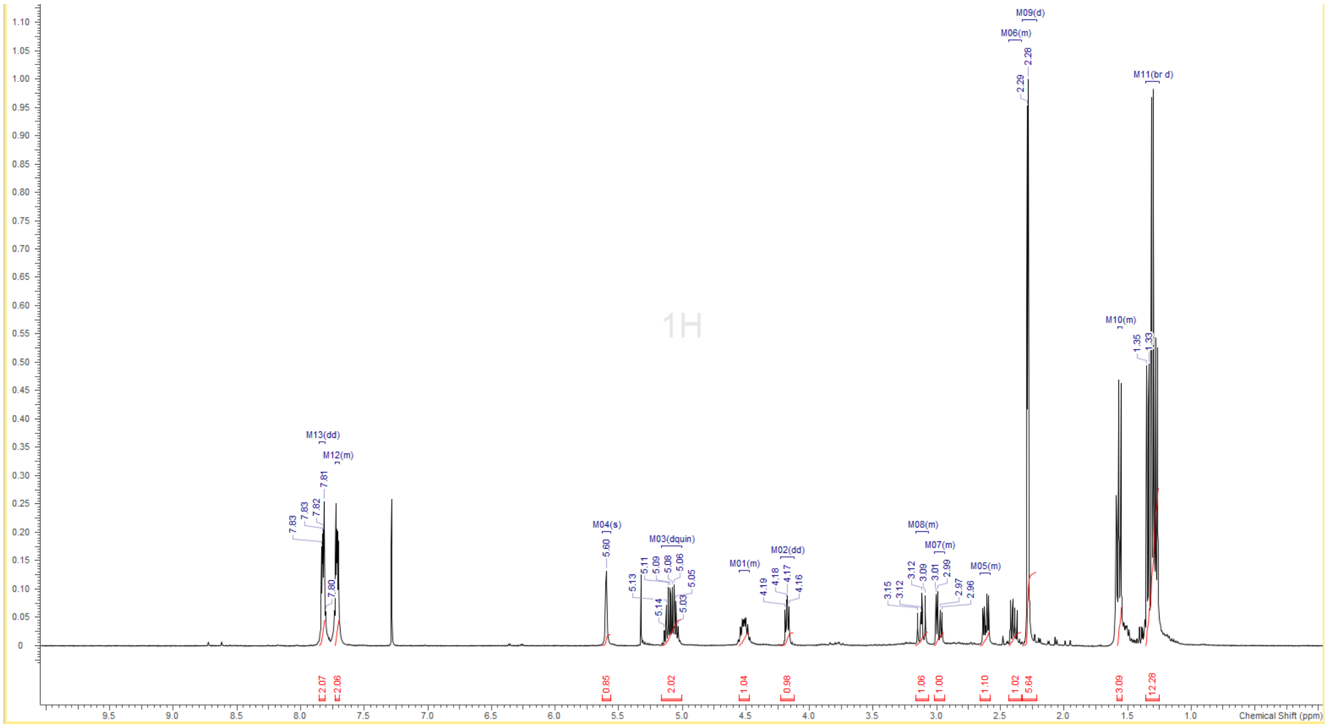

Diisopropyl 4-(((2-aminopropyl)thio)methyl)-2,6-dimethyl-1,4-dihydropyridine-3,5-dicarboxylate (1l)

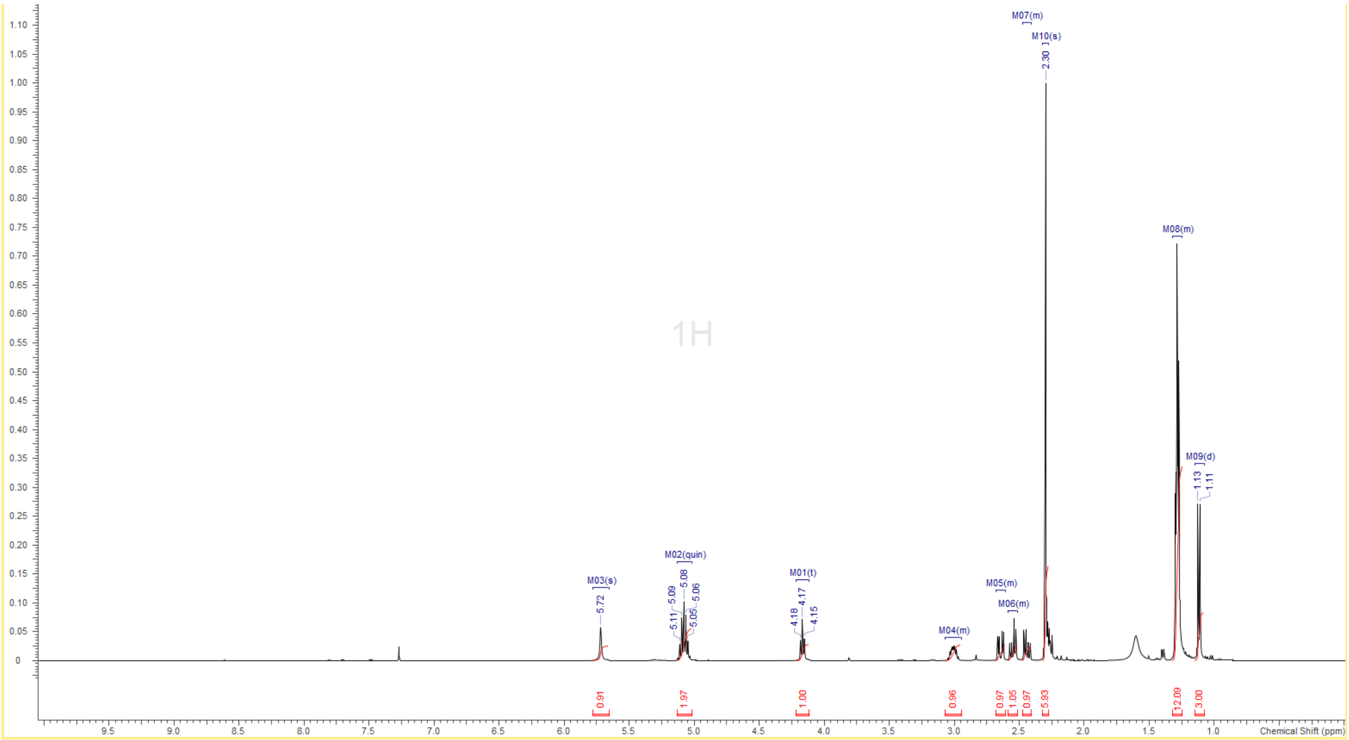
1H
M01(t)
M02(quin)
M03(s)
M04(m)
M05(m)
M06(m)
M07(m)
M08(m)
M09(d)
M10(s)
Chemical Shift (ppm)

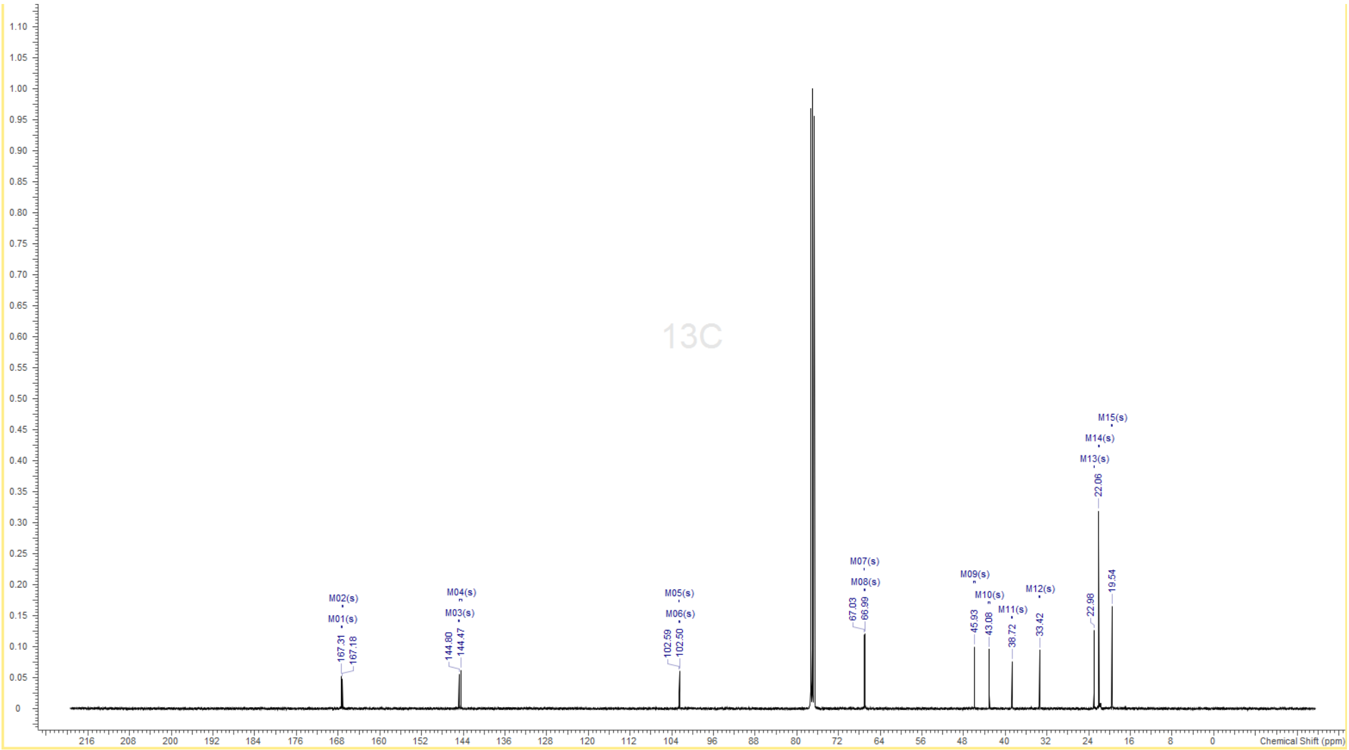
13C
M01(s)
M02(s)
M03(s)
M04(s)
M05(s)
M06(s)
M07(s)
M08(s)
M09(s)
M10(s)
M11(s)
M12(s)
M13(s)
M14(s)
M15(s)
Chemical Shift (ppm)

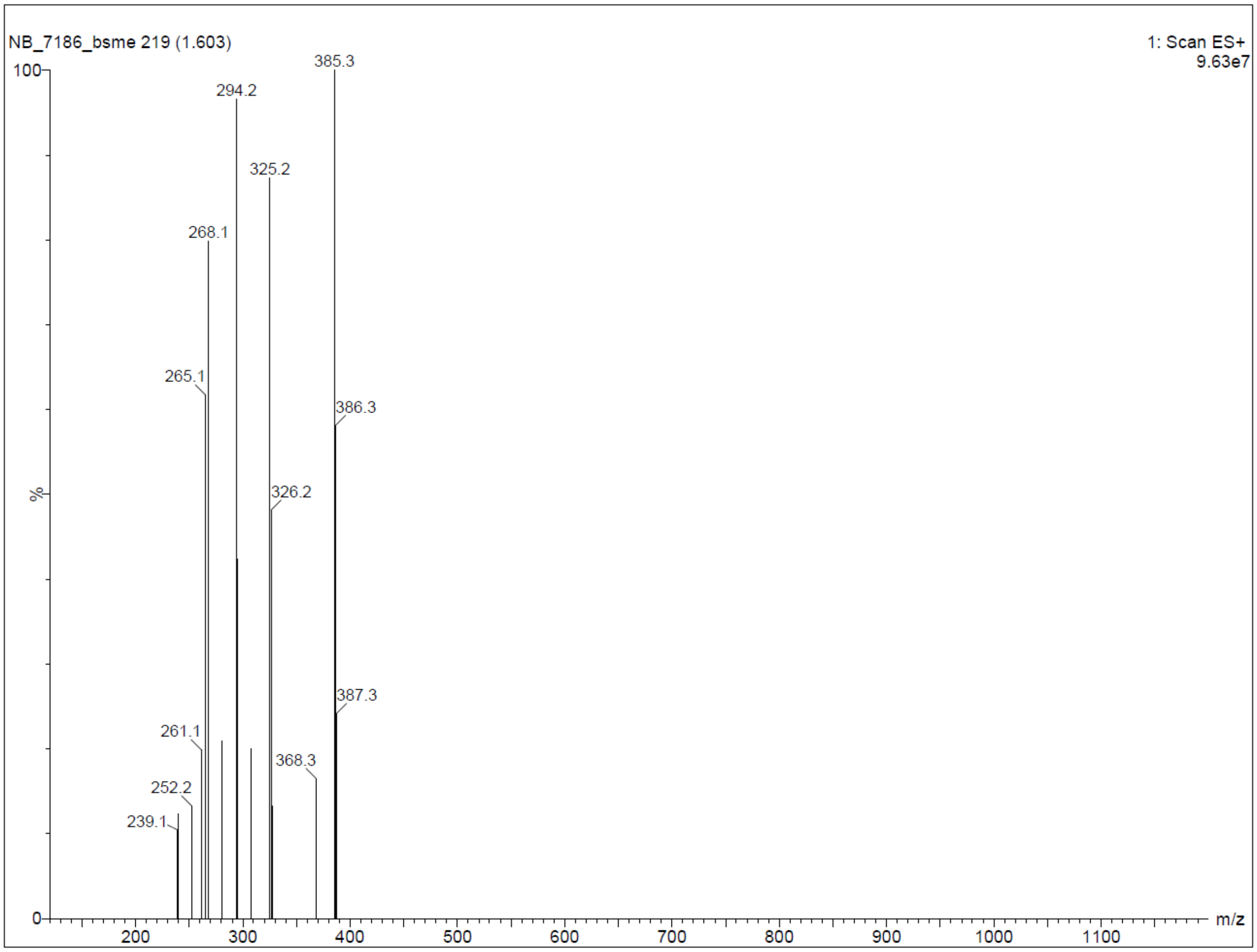
NB_7186_bsme 219 (1.603)
1: Scan ES+
9.63e7
100
%
0
385.3
294.2
325.2
268.1
265.1
386.3
326.2
387.3
261.1
368.3
252.2
239.1
200
300
400
500
600
700
800
900
1000
1100
m/z

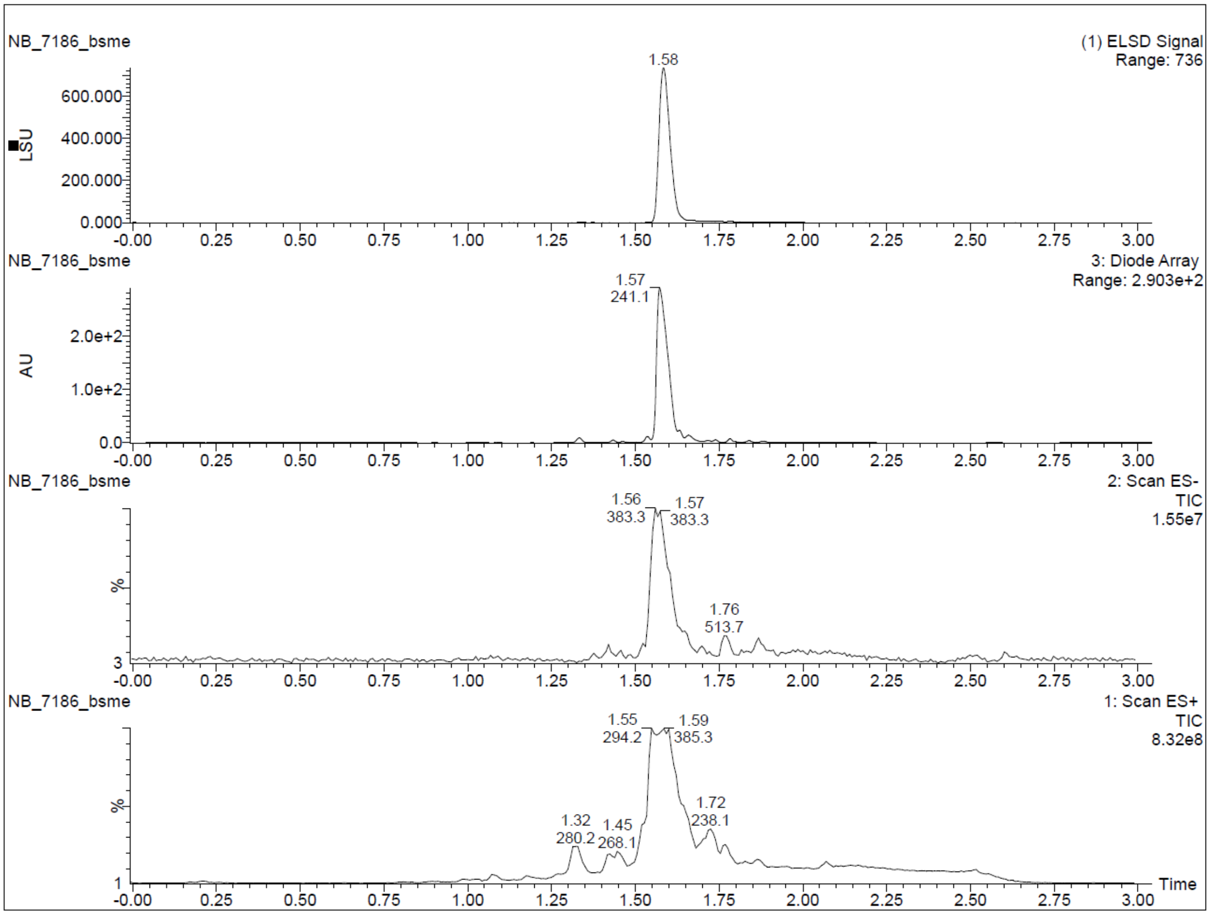

Piperazine-Forming Amino-DHP Reagents

2-(2-((2,2-Dimethoxyethyl)amino)ethyl)isoindoline-1,3-dione

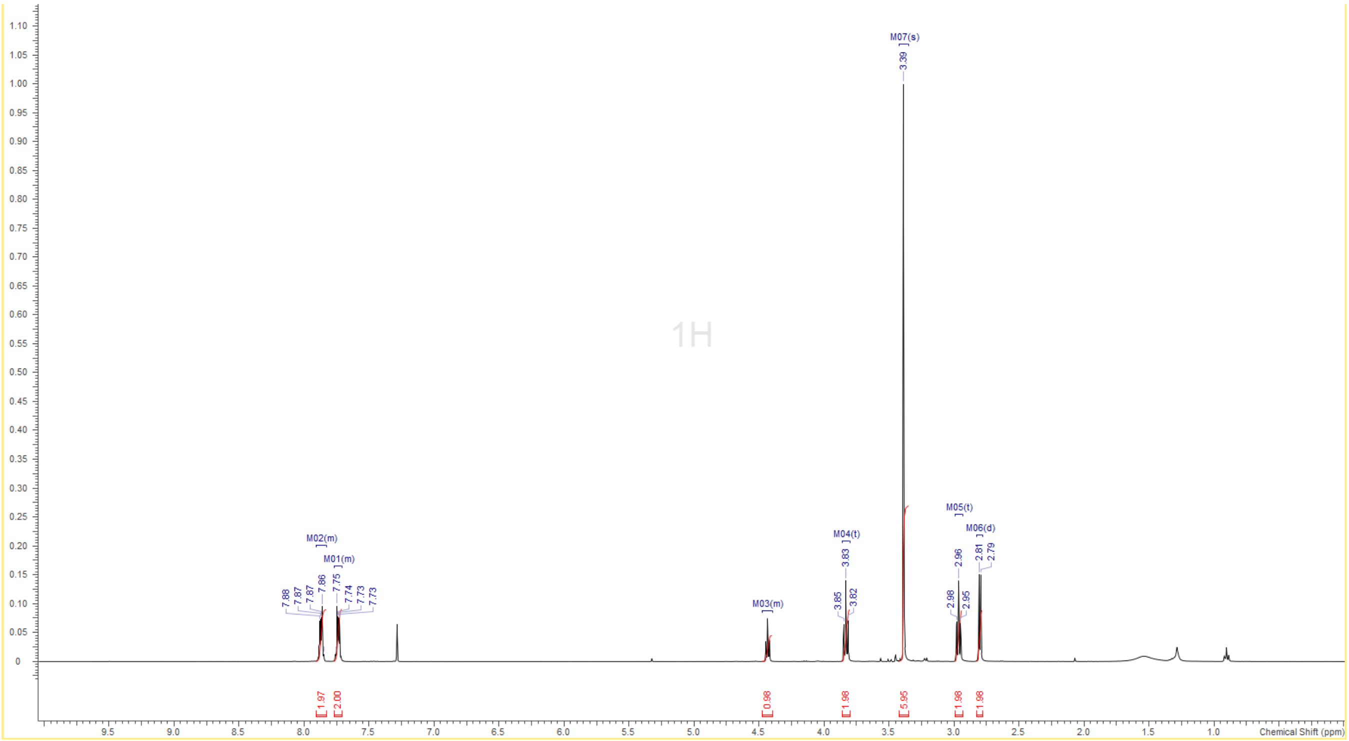

N-(2,2-Dimethoxyethyl)-N-(2-(1,3-dioxoisoindolin-2-yl)ethyl)-4-methylbenzenesulfonamide

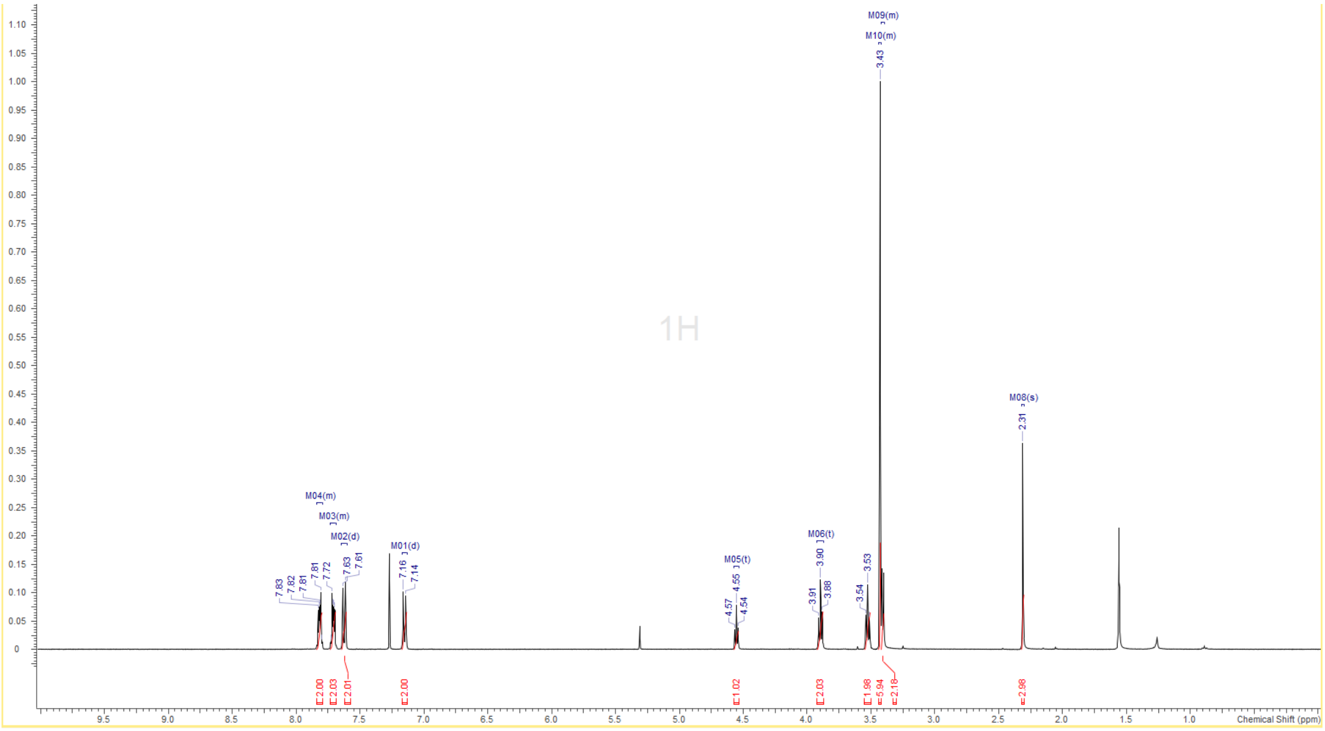

N-(2-(1,3-Dioxoisoindolin-2-yl)ethyl)-4-methyl-N-(2-oxoethyl)benzenesulfonamide

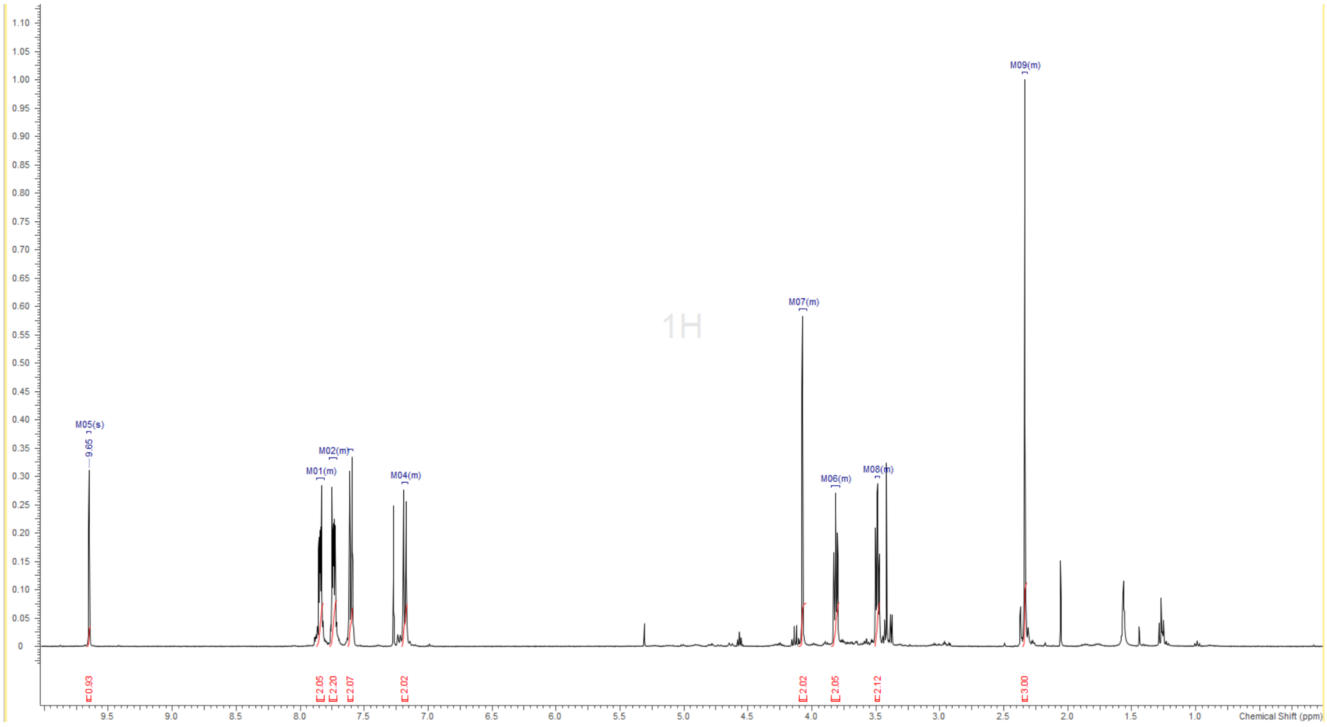

Diisopropyl 4-(((N-(2-(1,3-dioxoisoindolin-2-yl)ethyl)-4-methylphenyl)sulfonamido)methyl)-2,6-dimethyl-1,4-dihydropyridine-3,5-dicarboxylate

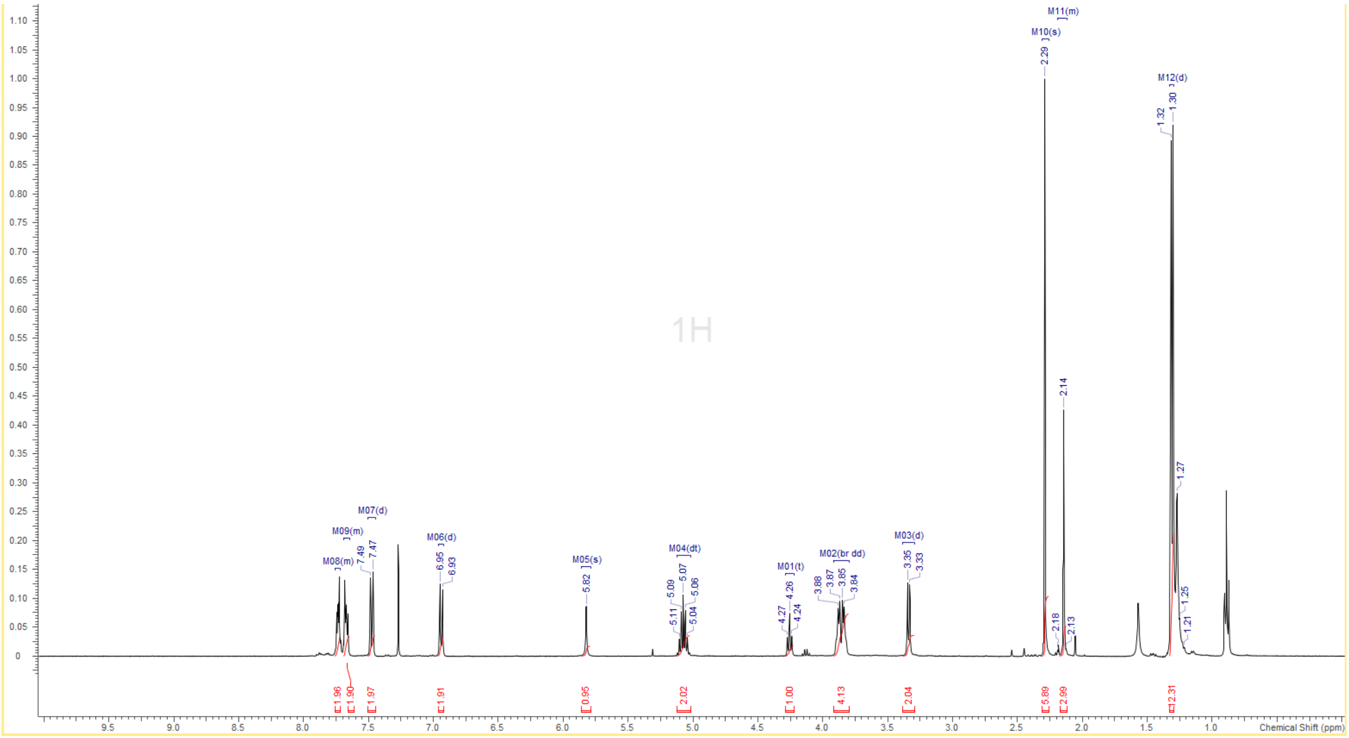

Diisopropyl 4-(((N-(2-aminoethyl)-4-methylphenyl)sulfonamido)methyl)-2,6-dimethyl-1,4-dihydropyridine-3,5-dicarboxylate (1n)

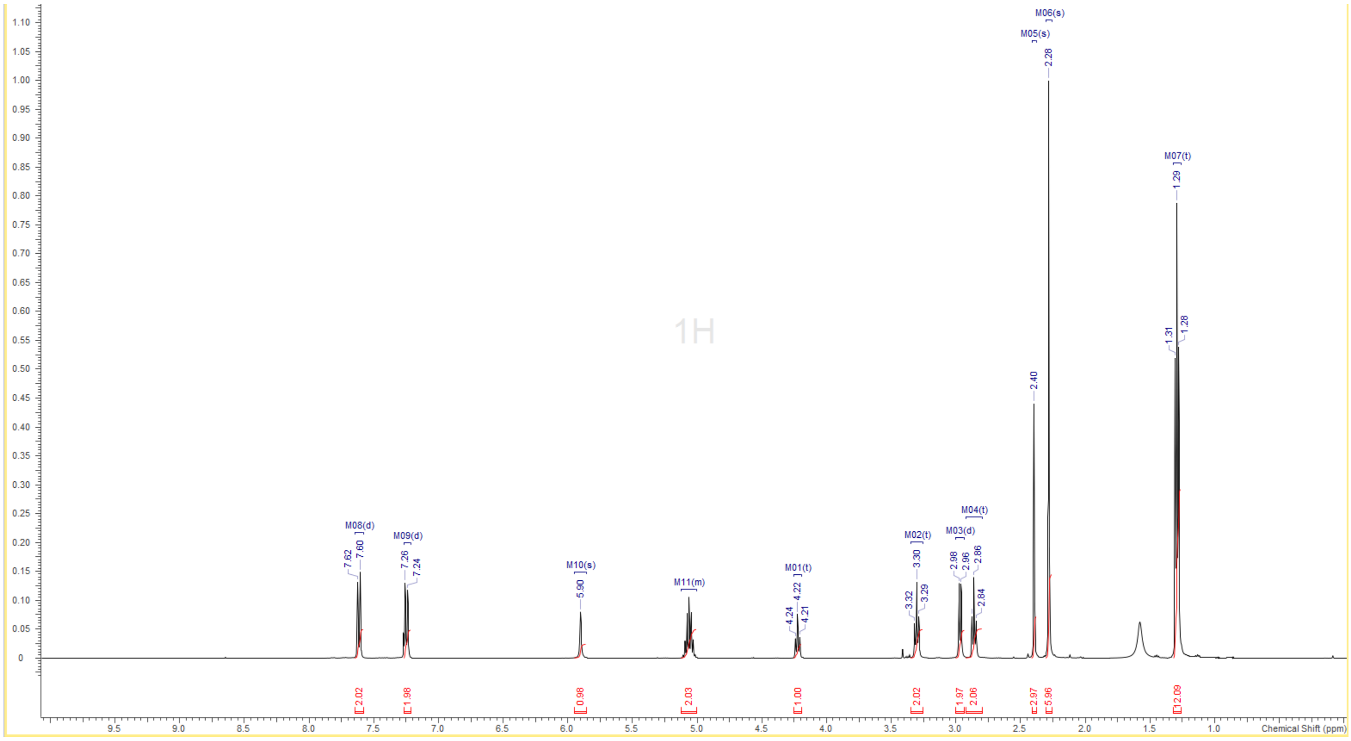

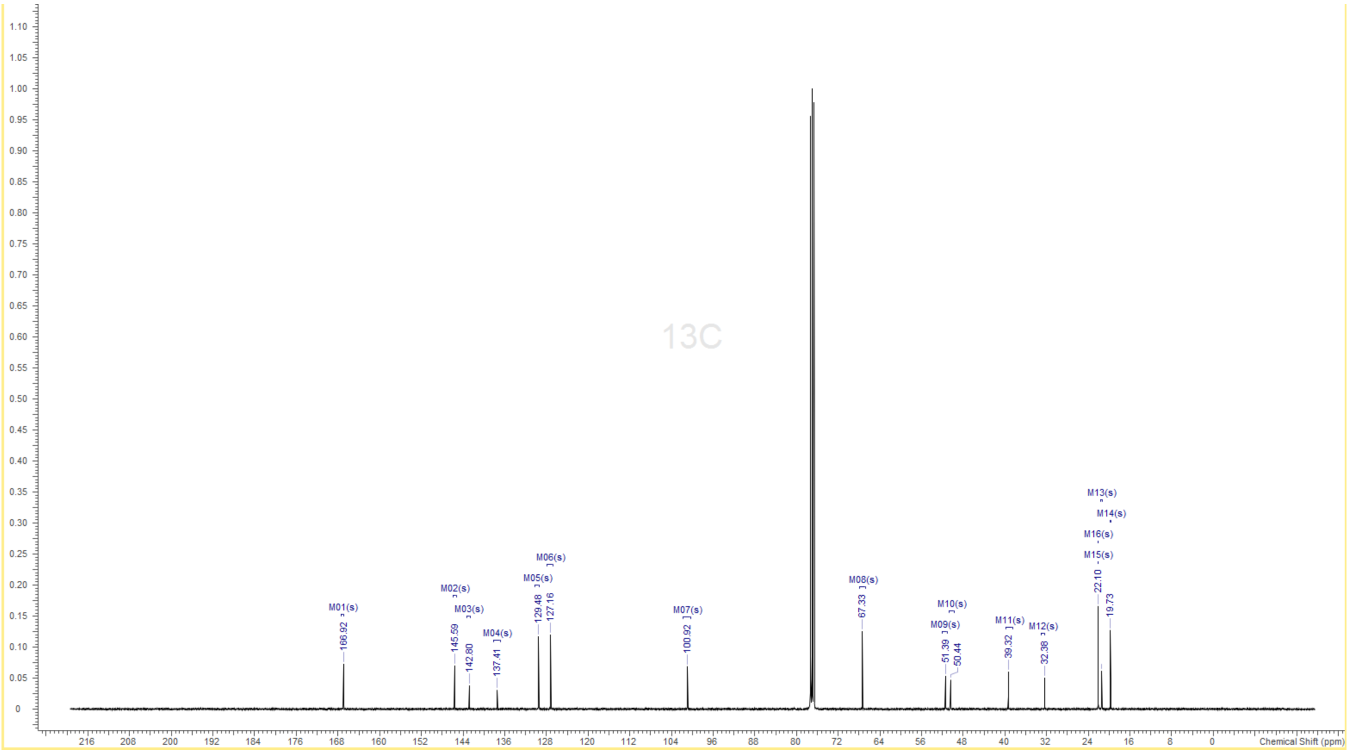

13C
M01(s) 166.92
M02(s) 145.59
M03(s) 142.80
M04(s) 137.41
M05(s) 129.48
M06(s) 127.16
M07(s) 100.92
M08(s) 67.33
M09(s) 51.39
M10(s) 50.44
M11(s) 39.32
M12(s) 32.38
M13(s)
M14(s)
M15(s)
M16(s)
22.10
19.73
Chemical Shift (ppm)

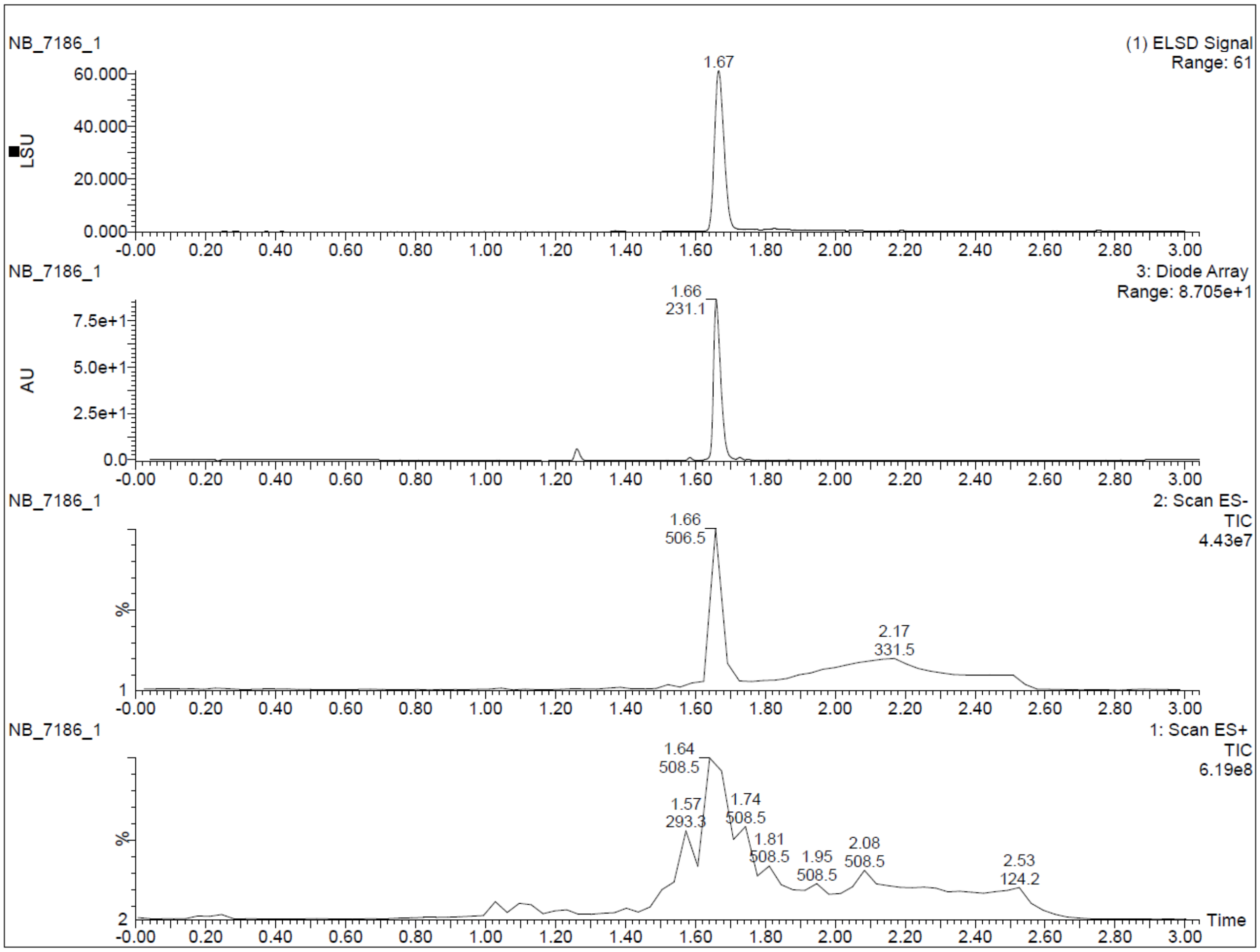

NB_7186_1
(1) ELSD Signal
Range: 61
1.67
LSU
NB_7186_1
3: Diode Array
Range: 8.705e+1
1.66
231.1
AU
NB_7186_1
2: Scan ES-
TIC
4.43e7
1.66
506.5
2.17
331.5
%
NB_7186_1
1: Scan ES+
TIC
6.19e8
1.64
508.5
1.57
293.3
1.74
508.5
1.81
508.5
1.95
508.5
2.08
508.5
2.53
124.2
%
Time

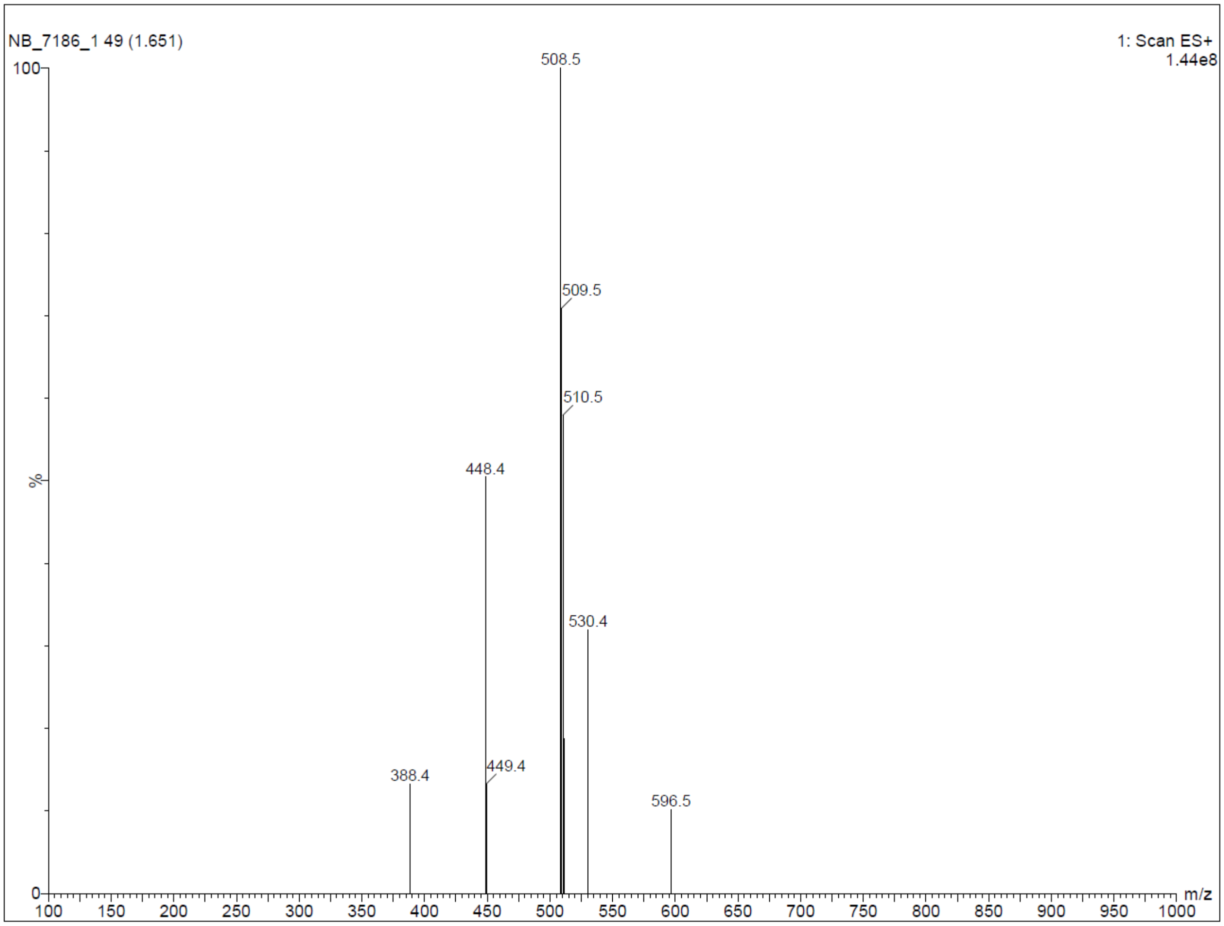

Benzyl (2,2-dimethoxyethyl)(2-(1,3-dioxoisoindolin-2-yl)ethyl)carbamate

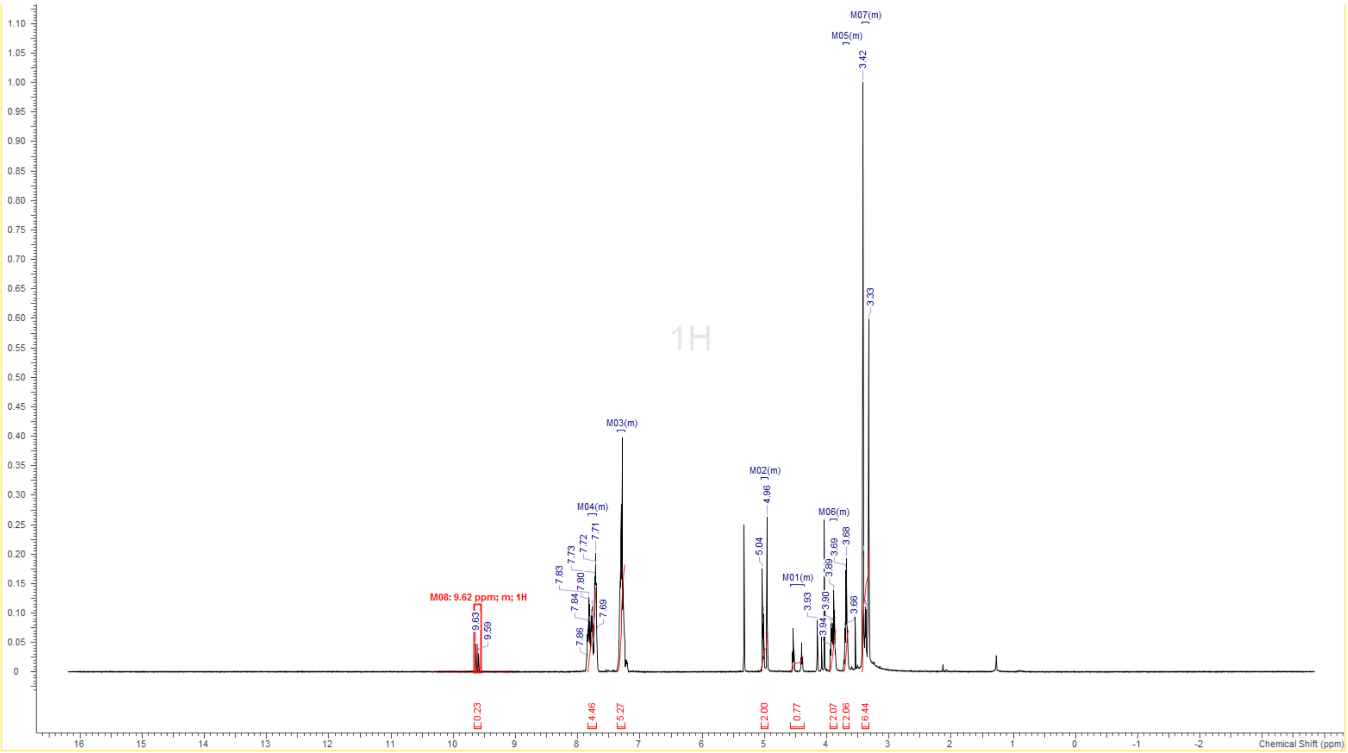

Diisopropyl 4-((((benzyloxy)carbonyl)(2-(1,3-dioxoisoindolin-2-yl)ethyl)amino)methyl)-2,6-dimethyl-1,4-dihydropyridine-3,5-dicarboxylate

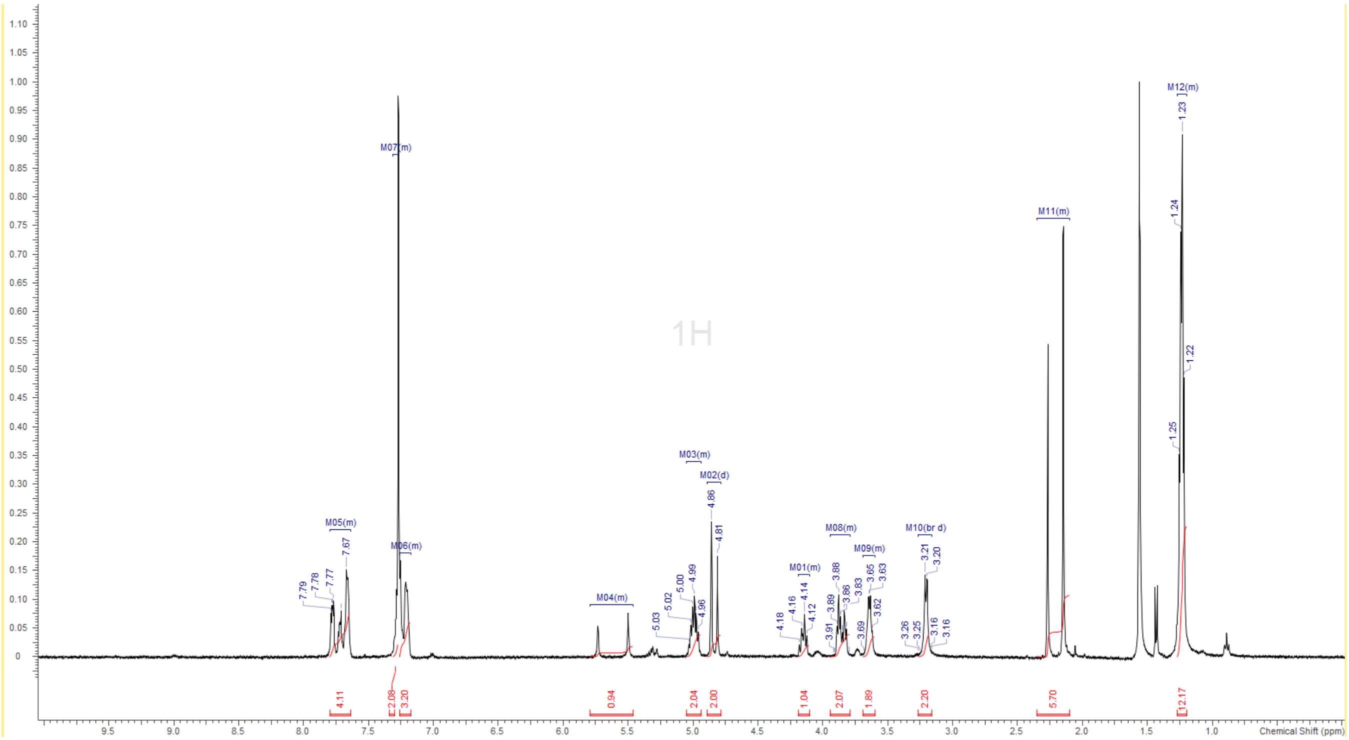

Diisopropyl 4-(((2-aminoethyl)((benzyloxy)carbonyl)amino)methyl)-2,6-dimethyl-1,4-dihydropyridine-3,5-dicarboxylate (1m)

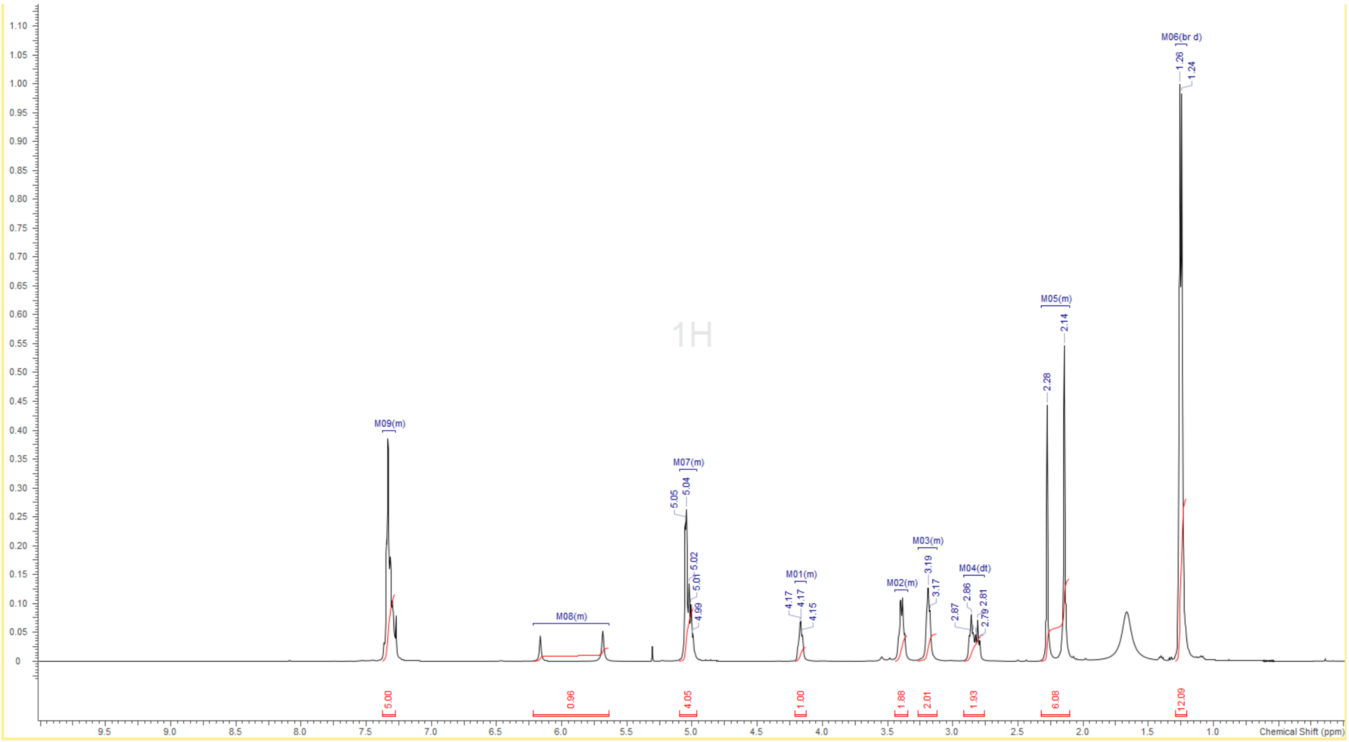

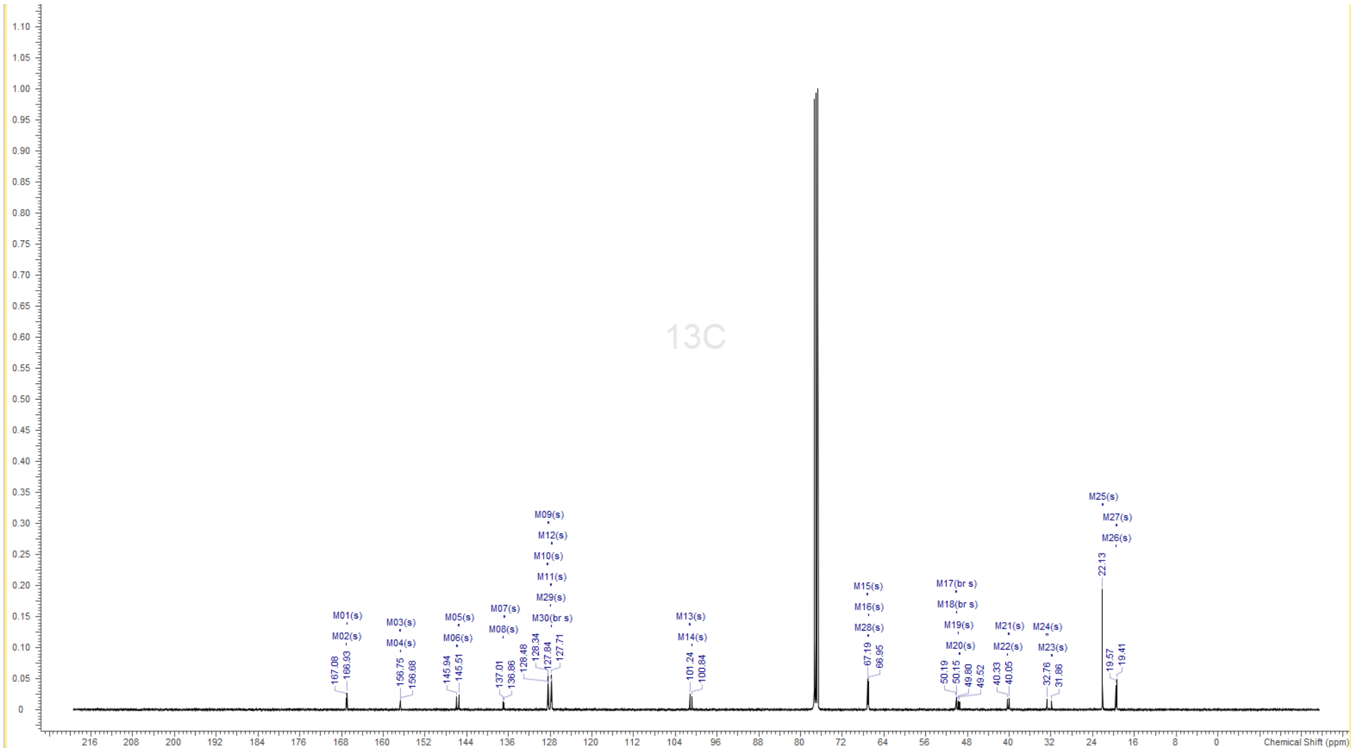
13C
Chemical Shift (ppm)
167.08
166.93
156.75
156.68
145.94
145.51
137.01
136.86
128.48
128.34
127.84
127.71
101.24
100.84
67.19
66.95
50.19
50.15
49.80
49.52
40.33
40.05
32.76
31.86
22.13
19.57
19.41

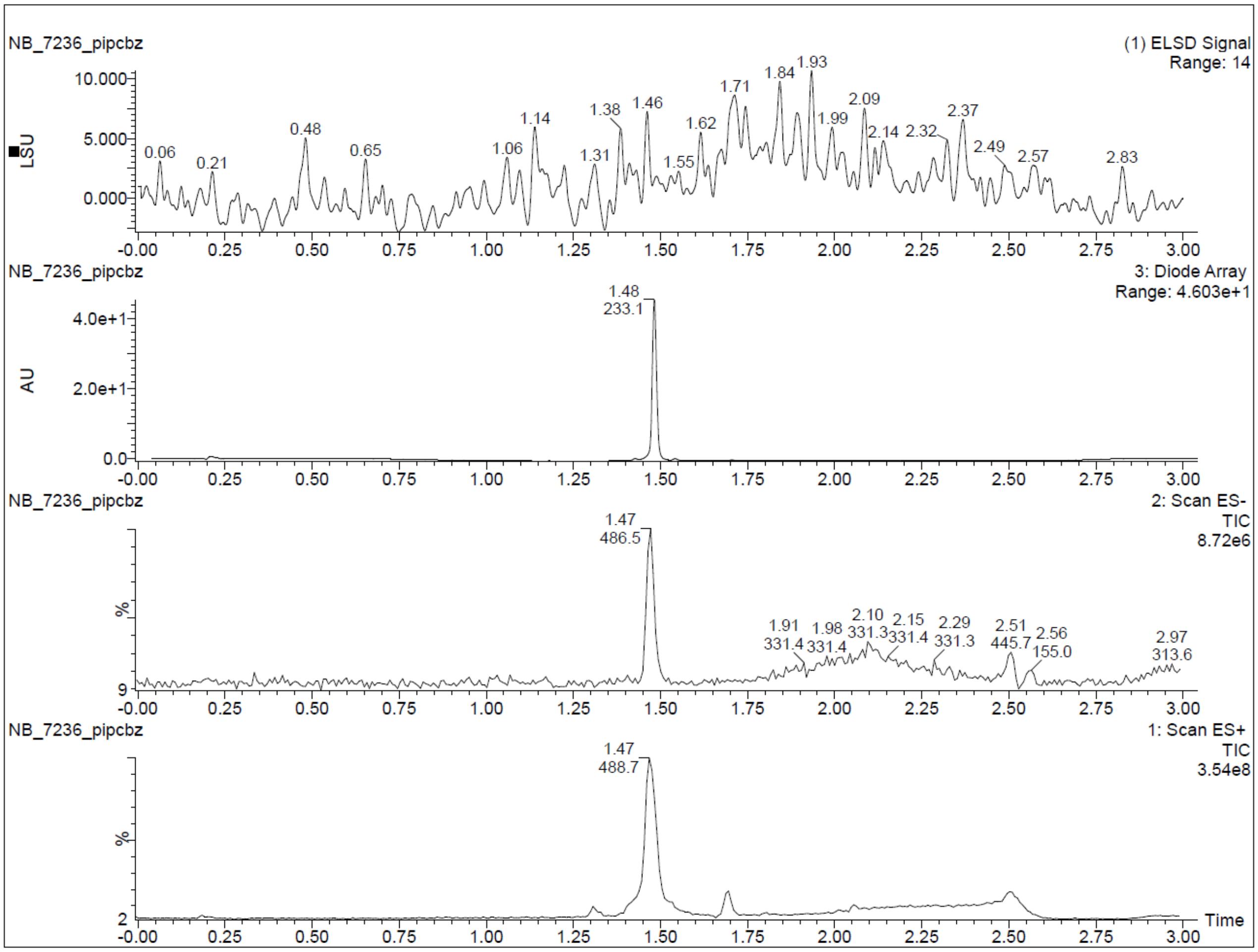
NB_7236_pipcbz
(1) ELSD Signal
Range: 14
LSU
NB_7236_pipcbz
3: Diode Array
Range: 4.603e+1
AU
1.48
233.1
NB_7236_pipcbz
2: Scan ES-
TIC
8.72e6
1.47
486.5
NB_7236_pipcbz
1: Scan ES+
TIC
3.54e8
1.47
488.7
Time

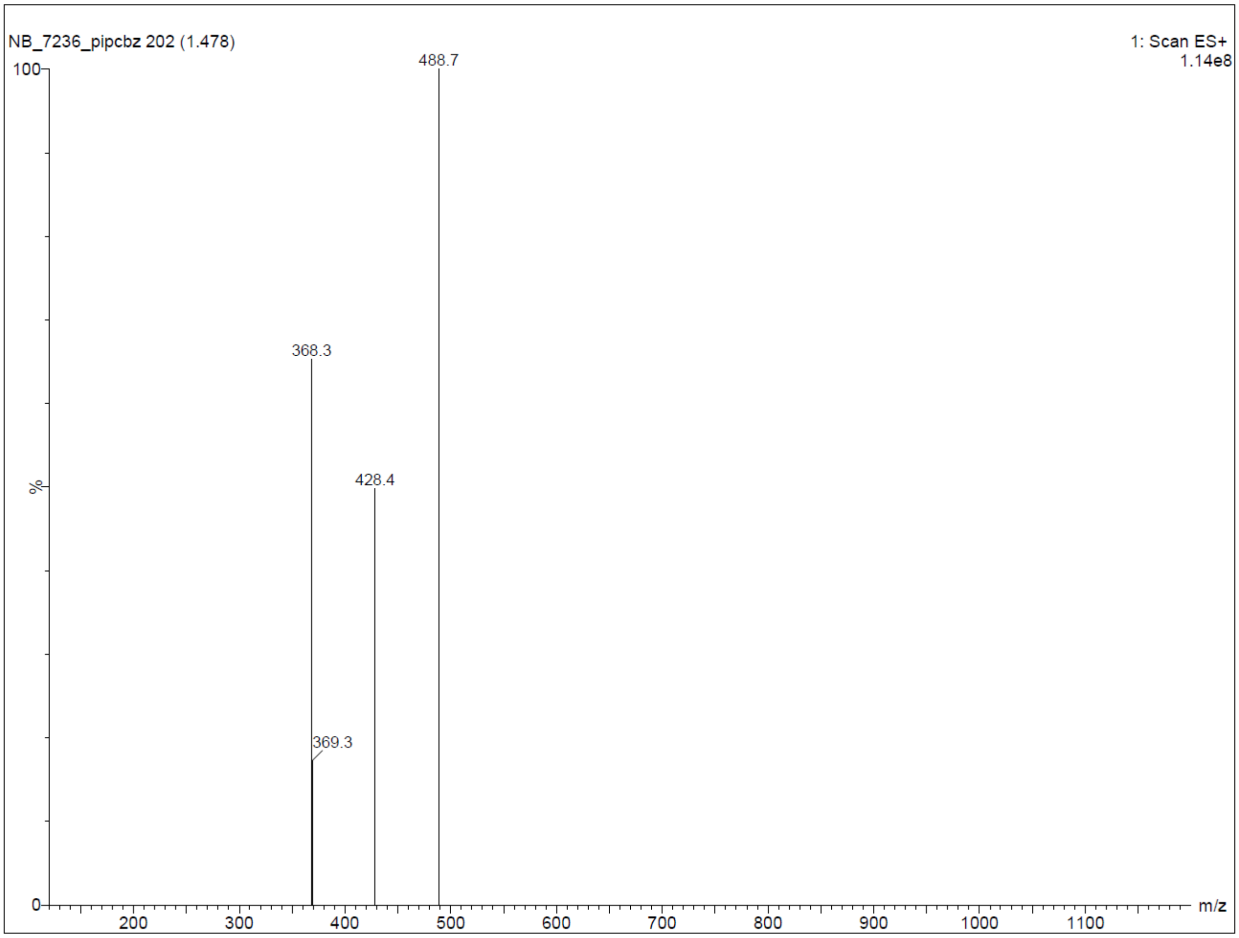

***tert*-Butyl (*S*)-(2-(1,3-dioxoisoindolin-2-yl)propyl)carbamate**

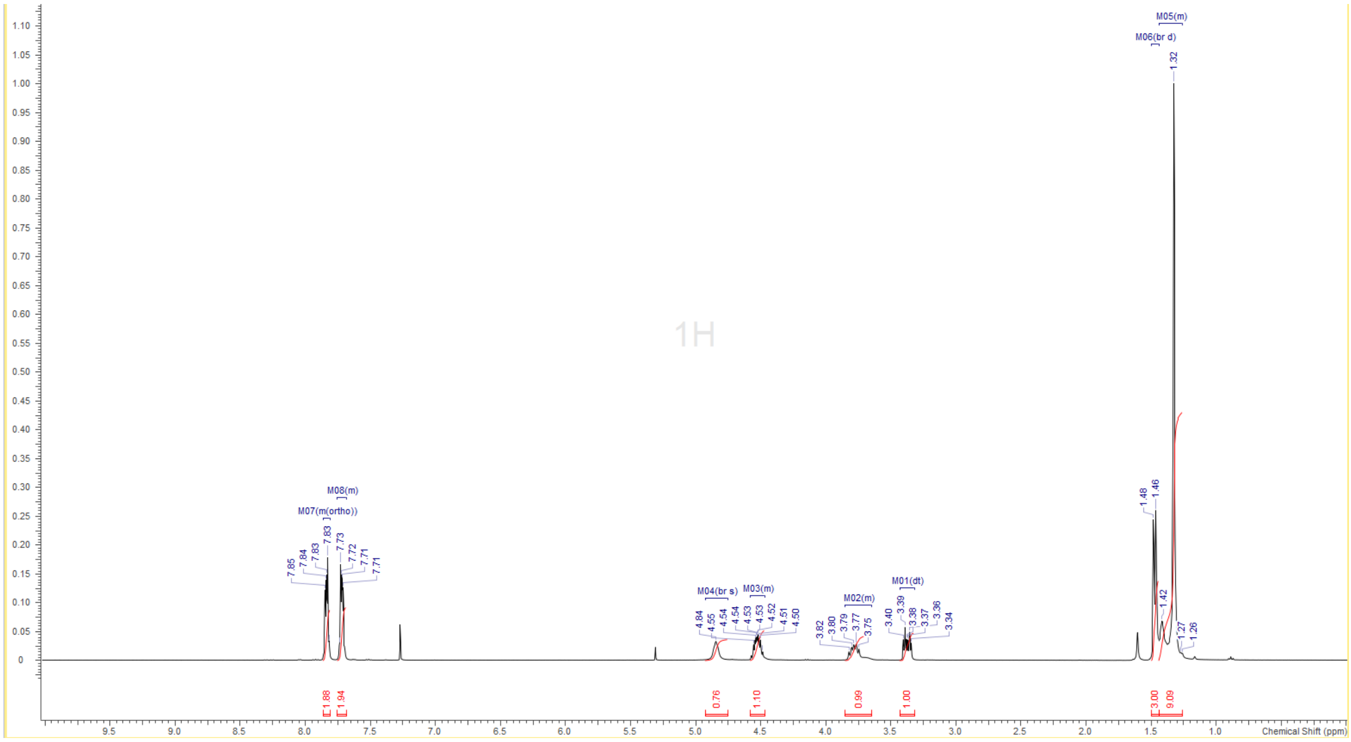

(S)-2-(1-Aminopropan-2-yl)isoindoline-1,3-dione, TFA Salt

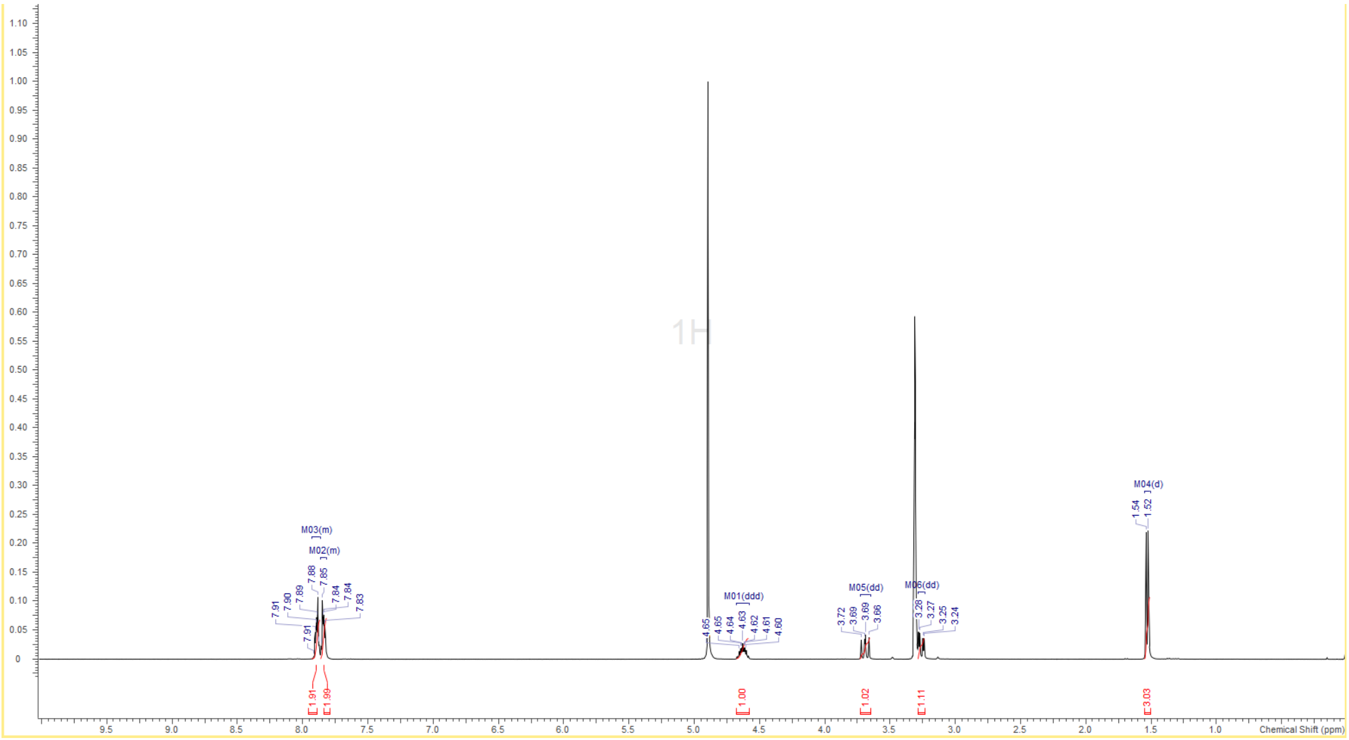

(S)-2-(1-((2,2-Dimethoxyethyl)amino)propan-2-yl)isoindoline-1,3-dione

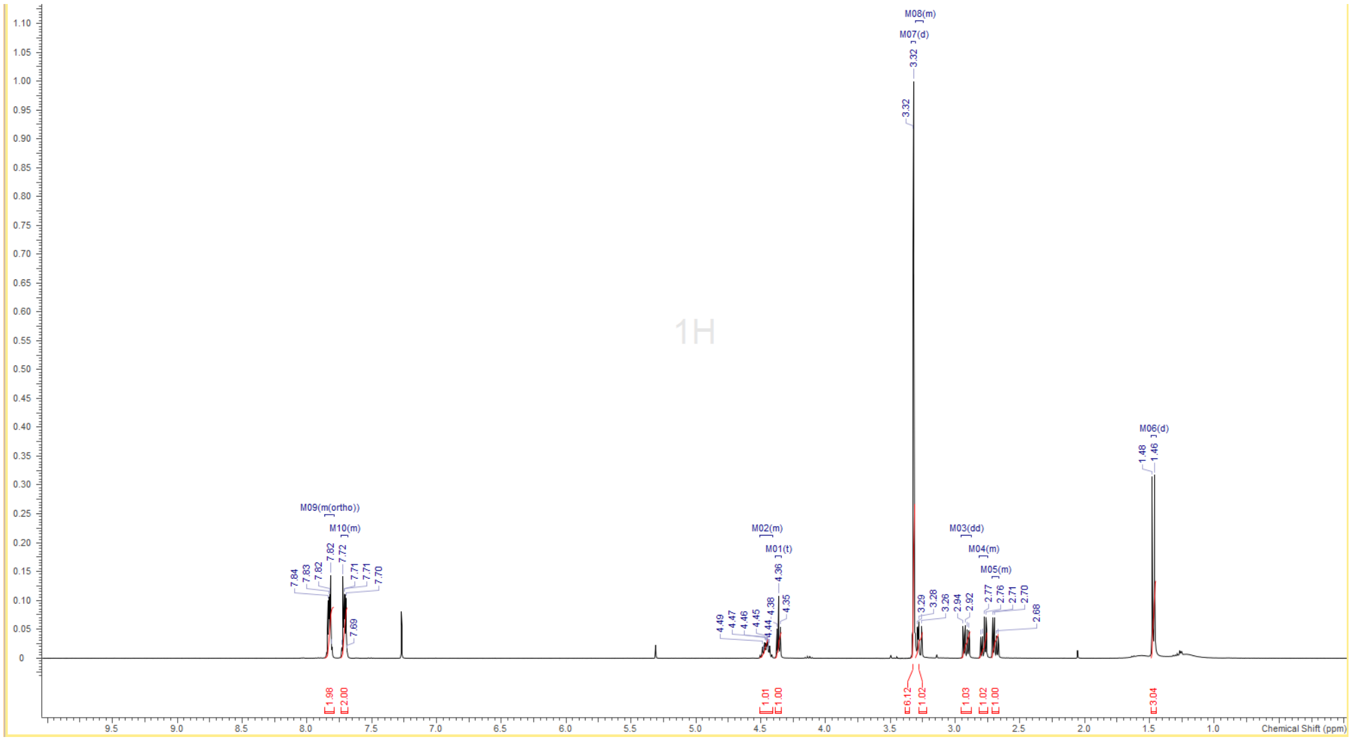

(S)-N-(2,2-Dimethoxyethyl)-N-(2-(1,3-dioxoisoindolin-2-yl)propyl)-4-methylbenzenesulfonamide

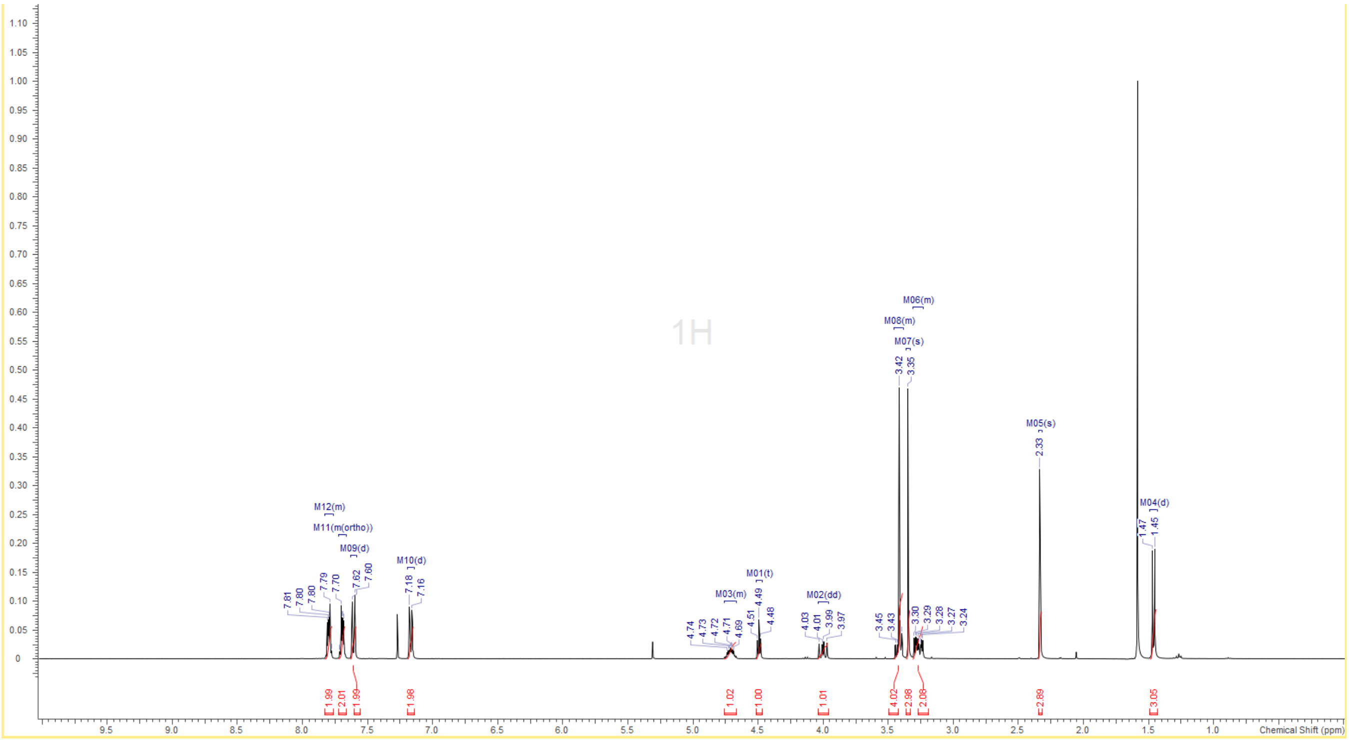

(S)-N-(2-(1,3-Dioxoisoindolin-2-yl)propyl)-4-methyl-N-(2-oxoethyl)benzenesulfonamide

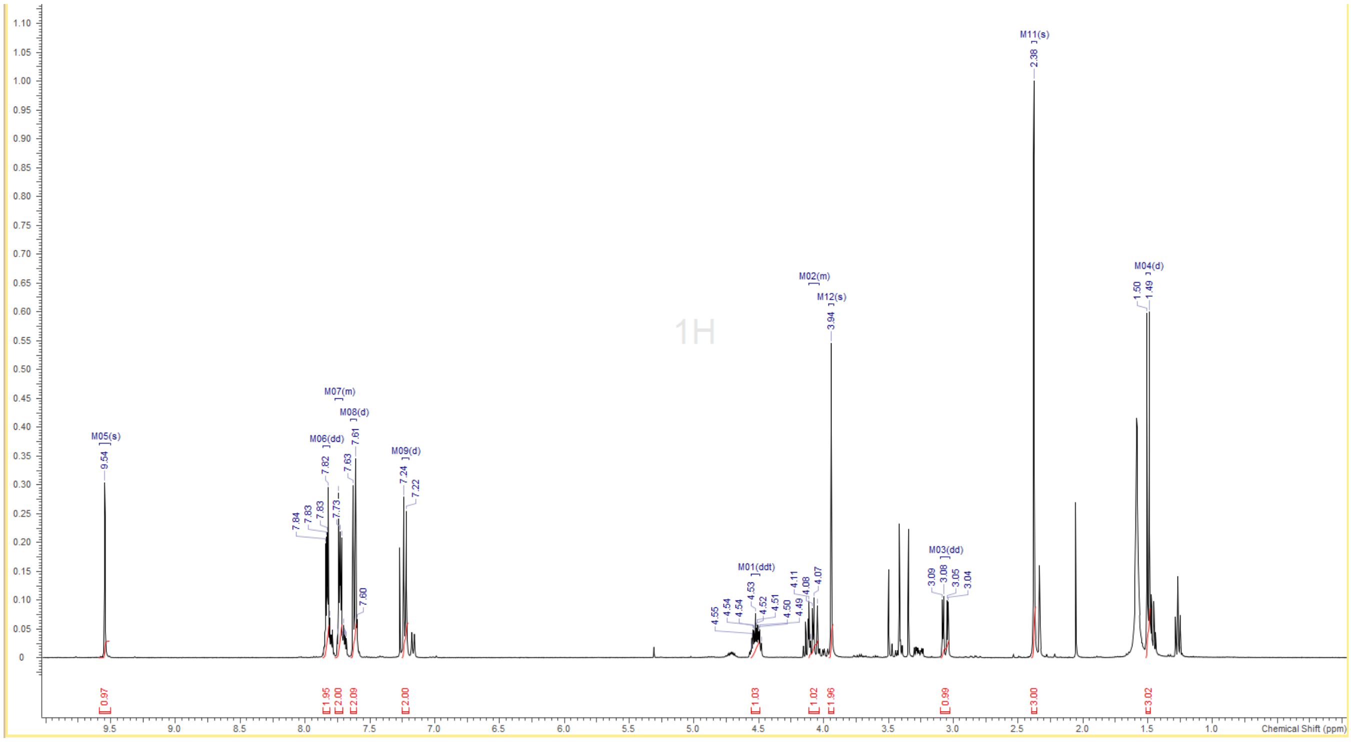

Diisopropyl (S)-4-(((N-(2-(1,3-dioxoisoindolin-2-yl)propyl)-4-methylphenyl)sulfonamido)methyl)-2,6-dimethyl-1,4-dihydropyridine-3,5-dicarboxylate

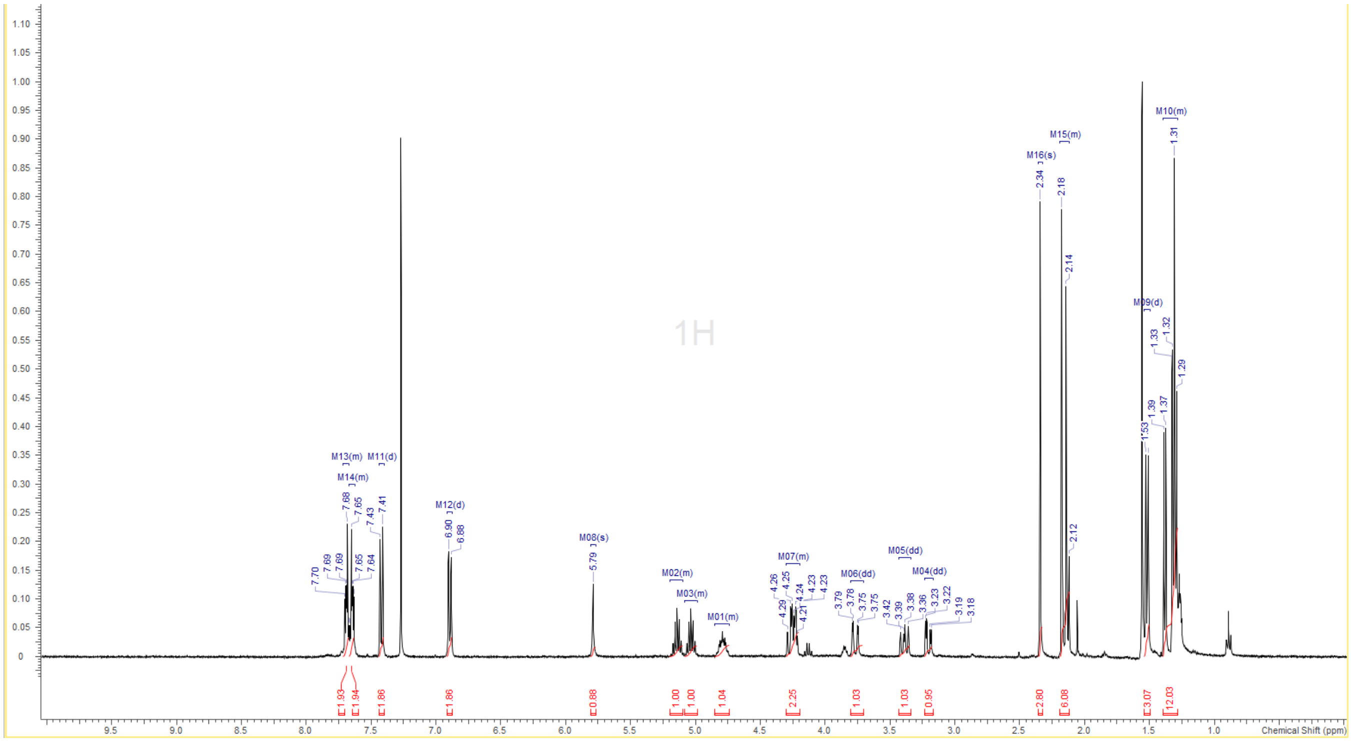

Diisopropyl (S)-4-(((N-(2-aminopropyl)-4-methylphenyl)sulfonamido)methyl)-2,6-dimethyl-1,4-dihydropyridine-3,5-dicarboxylate (1o)

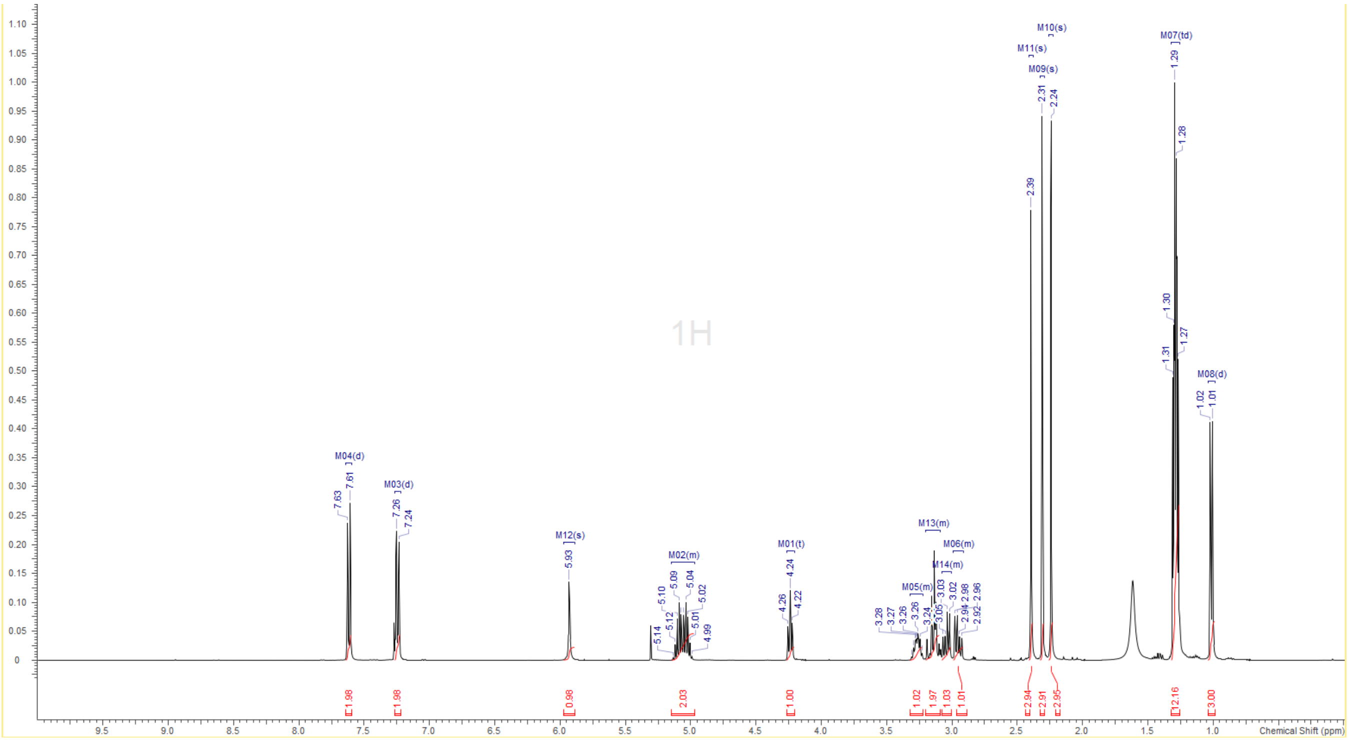

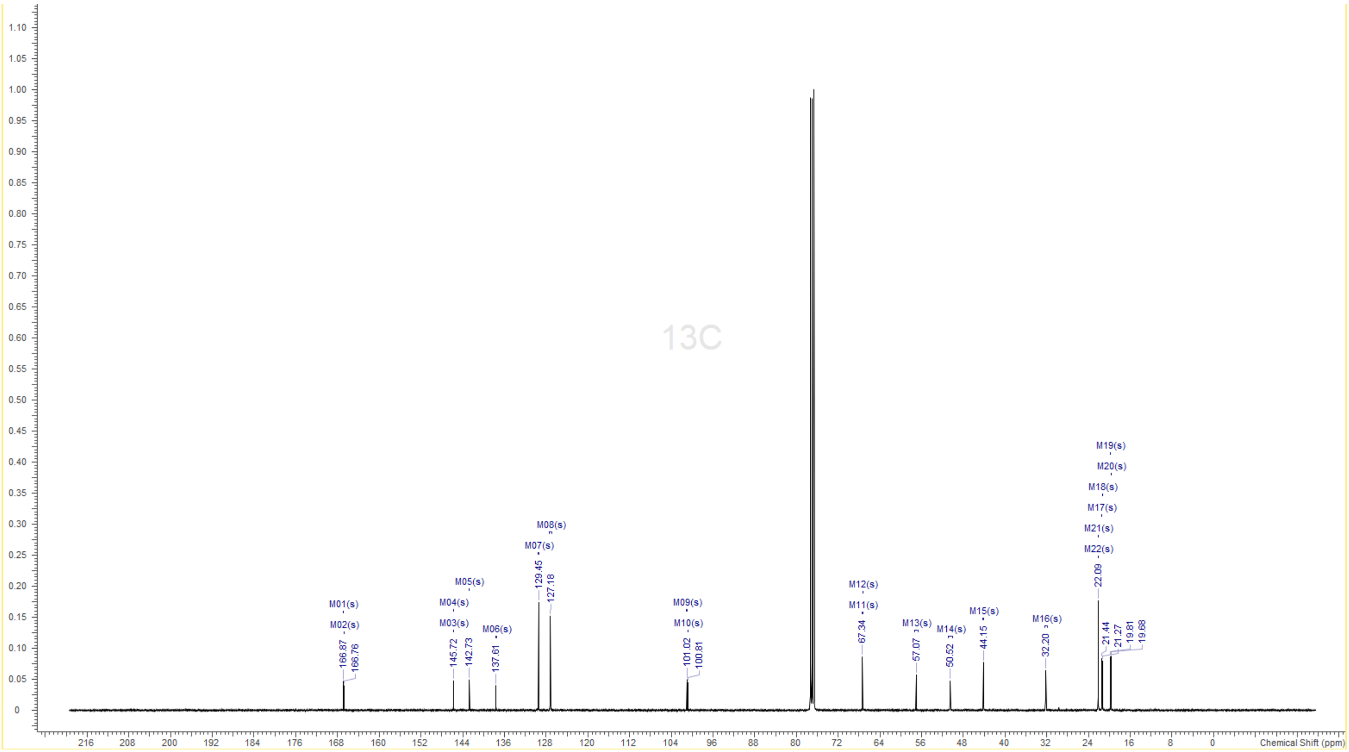
13C
M01(s)
M02(s)
166.87
166.76
M03(s)
M04(s)
M05(s)
145.72
142.73
M06(s)
137.61
M07(s)
M08(s)
129.45
127.18
M09(s)
M10(s)
101.02
100.81
M11(s)
M12(s)
67.34
M13(s)
57.07
M14(s)
50.52
M15(s)
44.15
M16(s)
32.20
M22(s)
M21(s)
M17(s)
M18(s)
M20(s)
M19(s)
22.09
21.44
21.27
19.81
19.68
Chemical Shift (ppm)

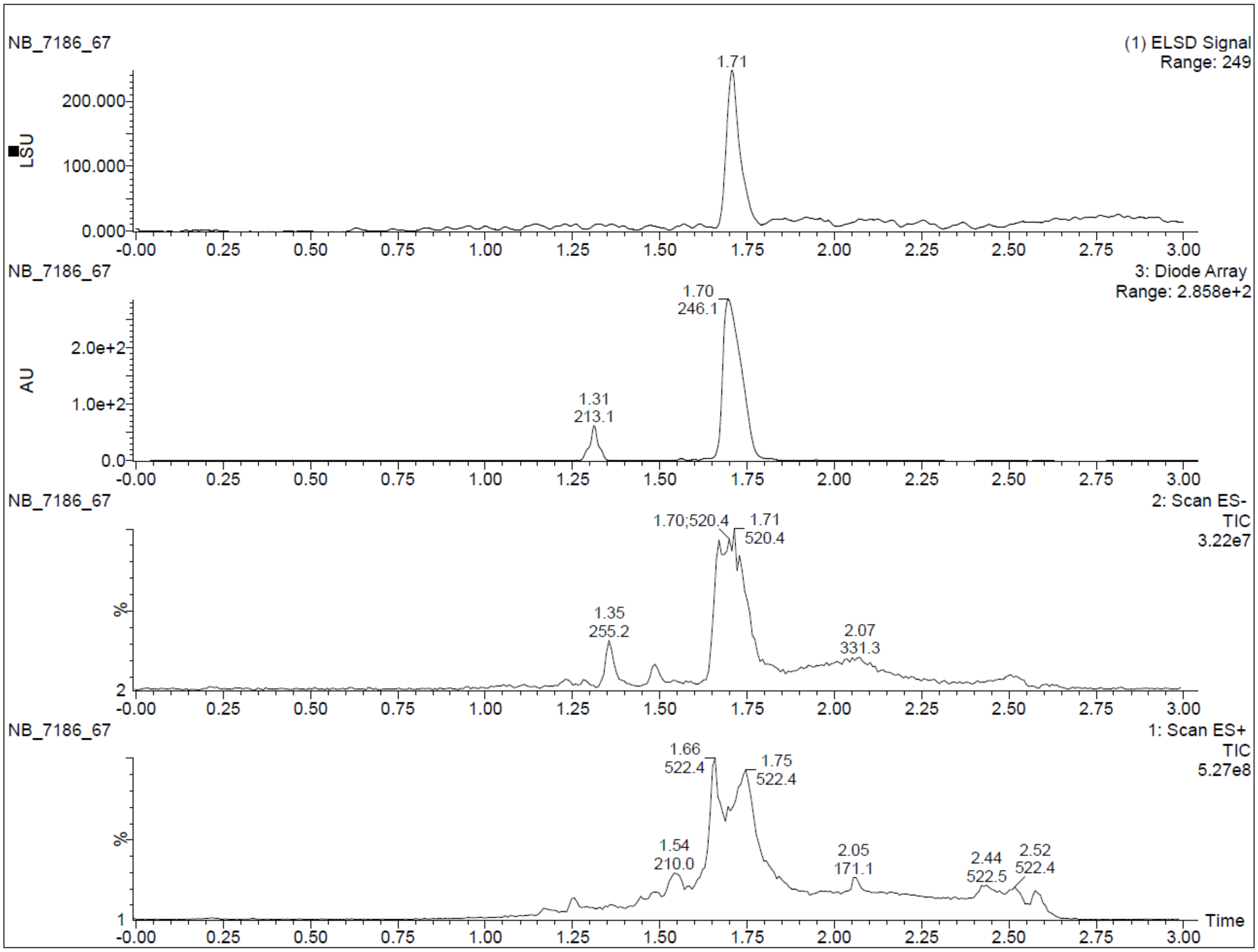
NB_7186_67
(1) ELSD Signal
Range: 249
LSU
1.71
NB_7186_67
3: Diode Array
Range: 2.858e+2
AU
1.70
246.1
1.31
213.1
NB_7186_67
2: Scan ES-
TIC
3.22e7
%
1.70;520.4
1.71
520.4
1.35
255.2
2.07
331.3
NB_7186_67
1: Scan ES+
TIC
5.27e8
%
1.66
522.4
1.75
522.4
1.54
210.0
2.05
171.1
2.44
522.5
2.52
522.4
Time

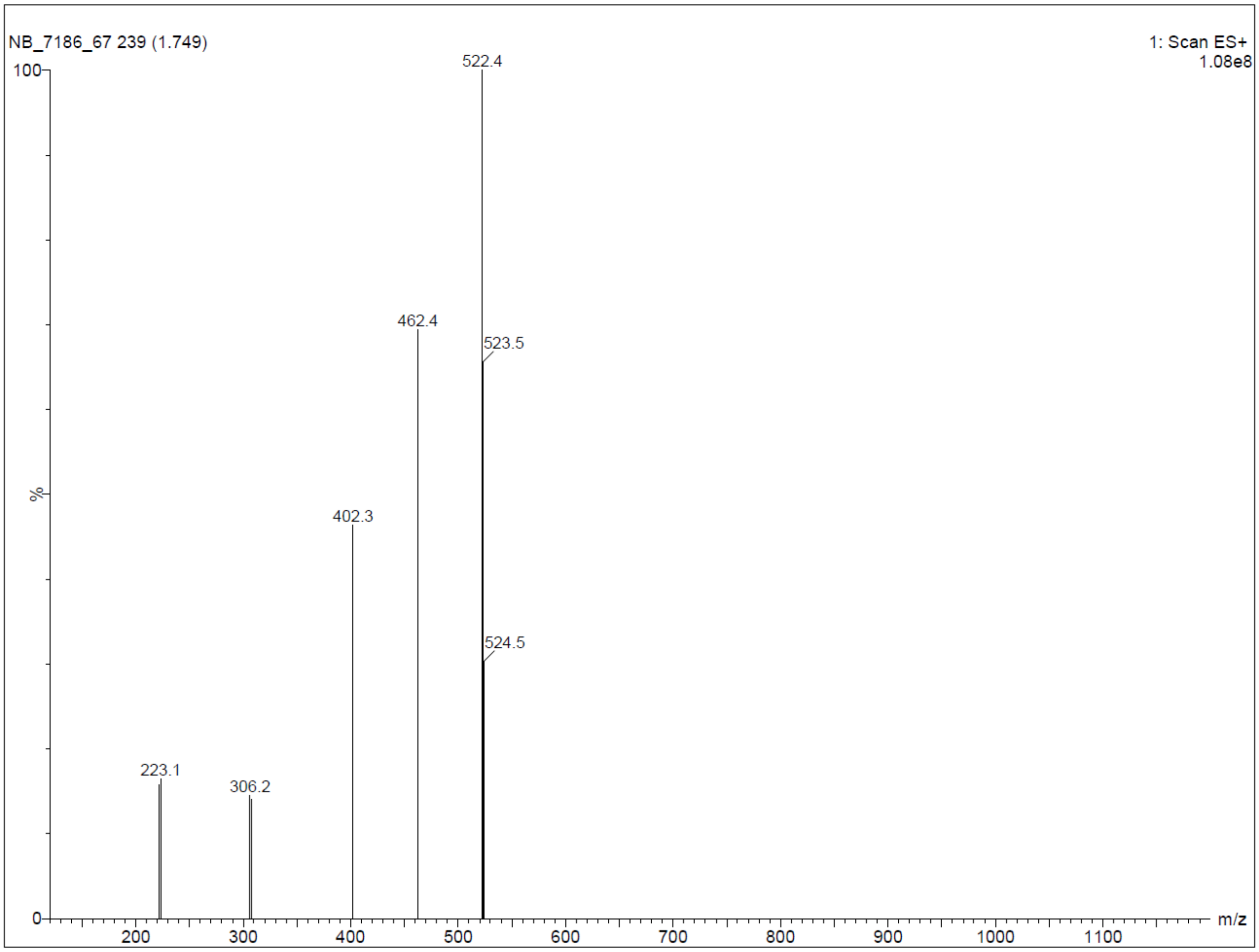

***tert*-Butyl (1-(1,3-dioxoisoindolin-2-yl)propan-2-yl)carbamate**

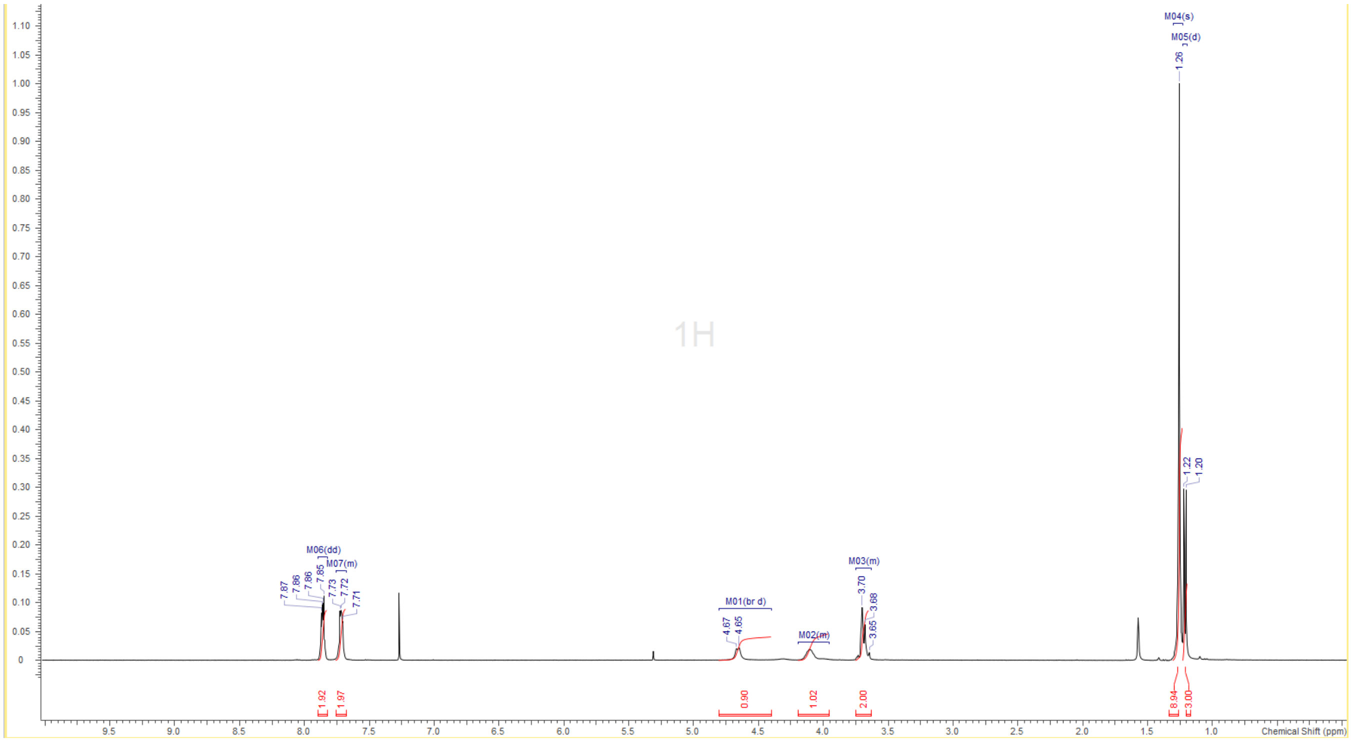

2-(2-Aminopropyl)isoindoline-1,3-dione, TFA Salt

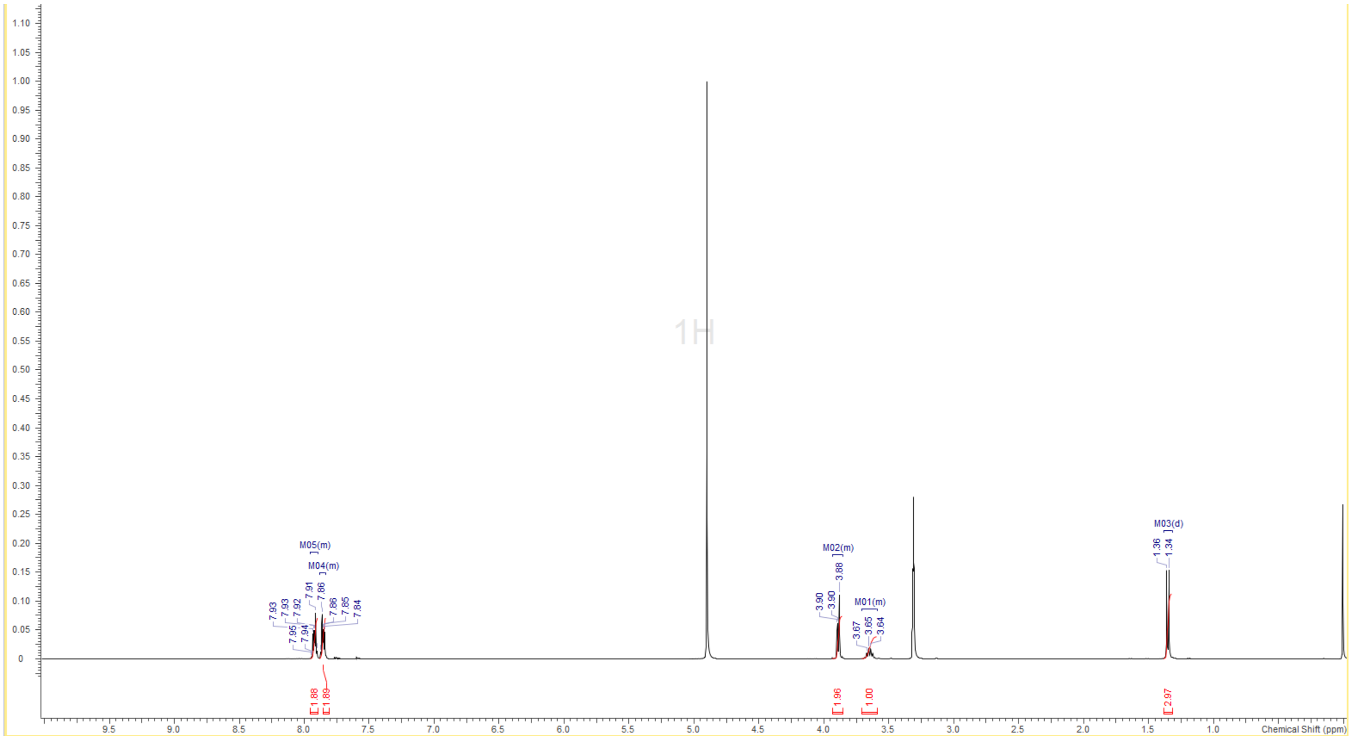

2-(2-((2,2-Dimethoxyethyl)amino)propyl)isoindoline-1,3-dione

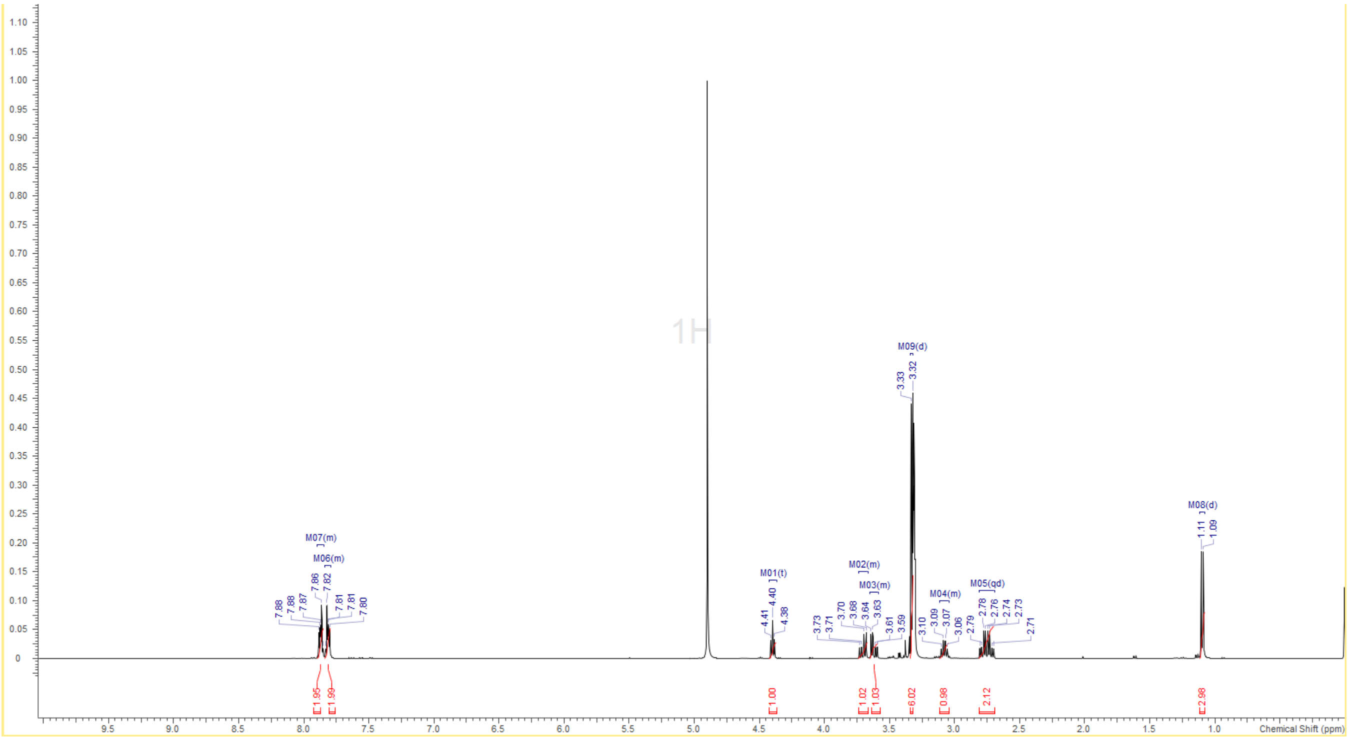

N-(2,2-Dimethoxyethyl)-N-(1-(1,3-dioxoisoindolin-2-yl)propan-2-yl)-4-methylbenzenesulfonamide

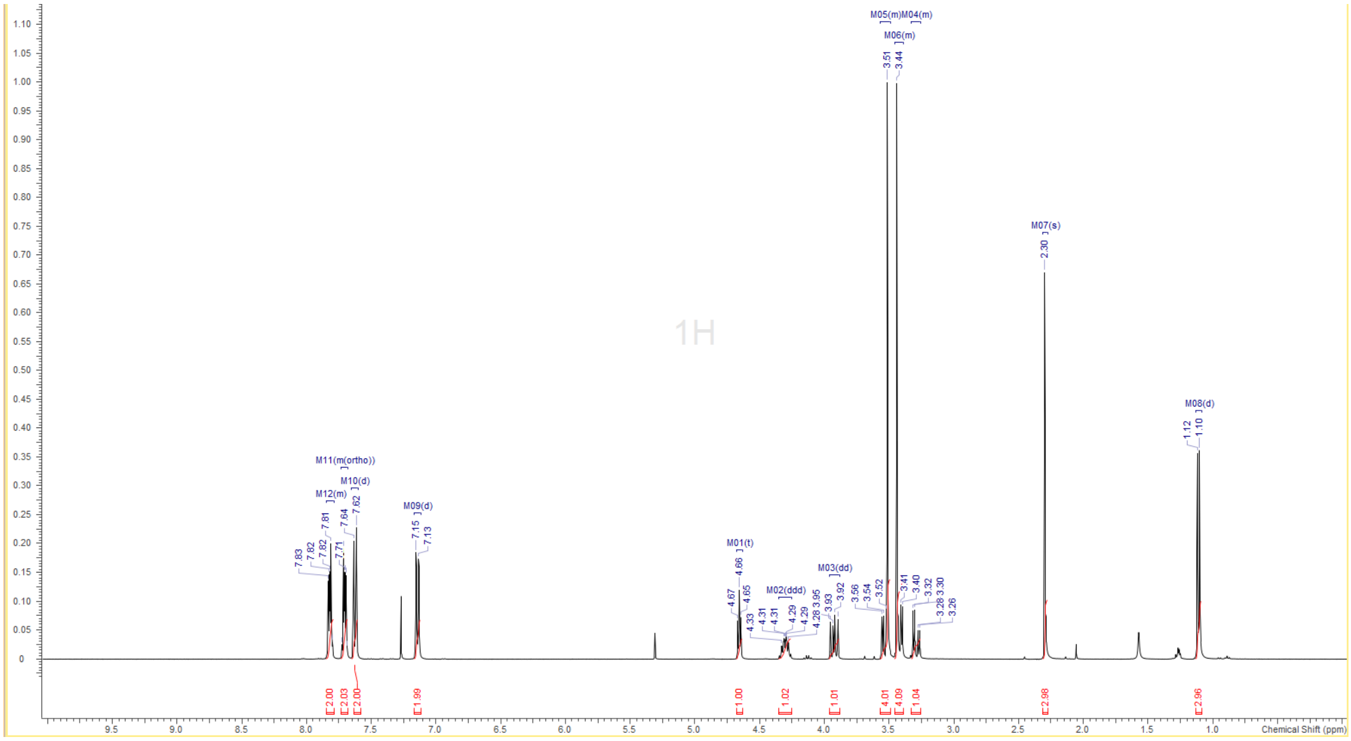

N-(1-(1,3-Dioxoisoindolin-2-yl)propan-2-yl)-4-methyl-N-(2-oxoethyl)benzenesulfonamide

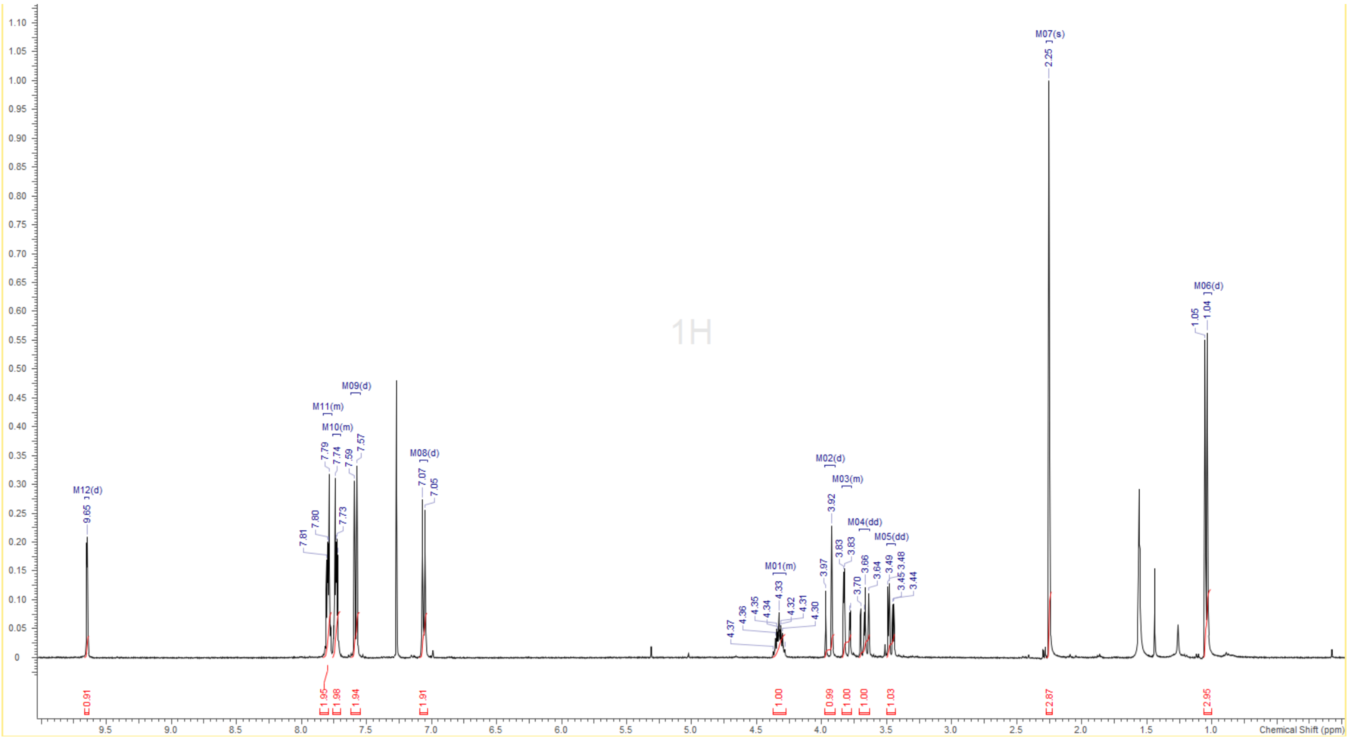

Diisopropyl 4-(((N-(1-(1,3-dioxoisoindolin-2-yl)propan-2-yl)-4-methylphenyl)sulfonamido)methyl)-2,6-dimethyl-1,4-dihydropyridine-3,5-dicarboxylate

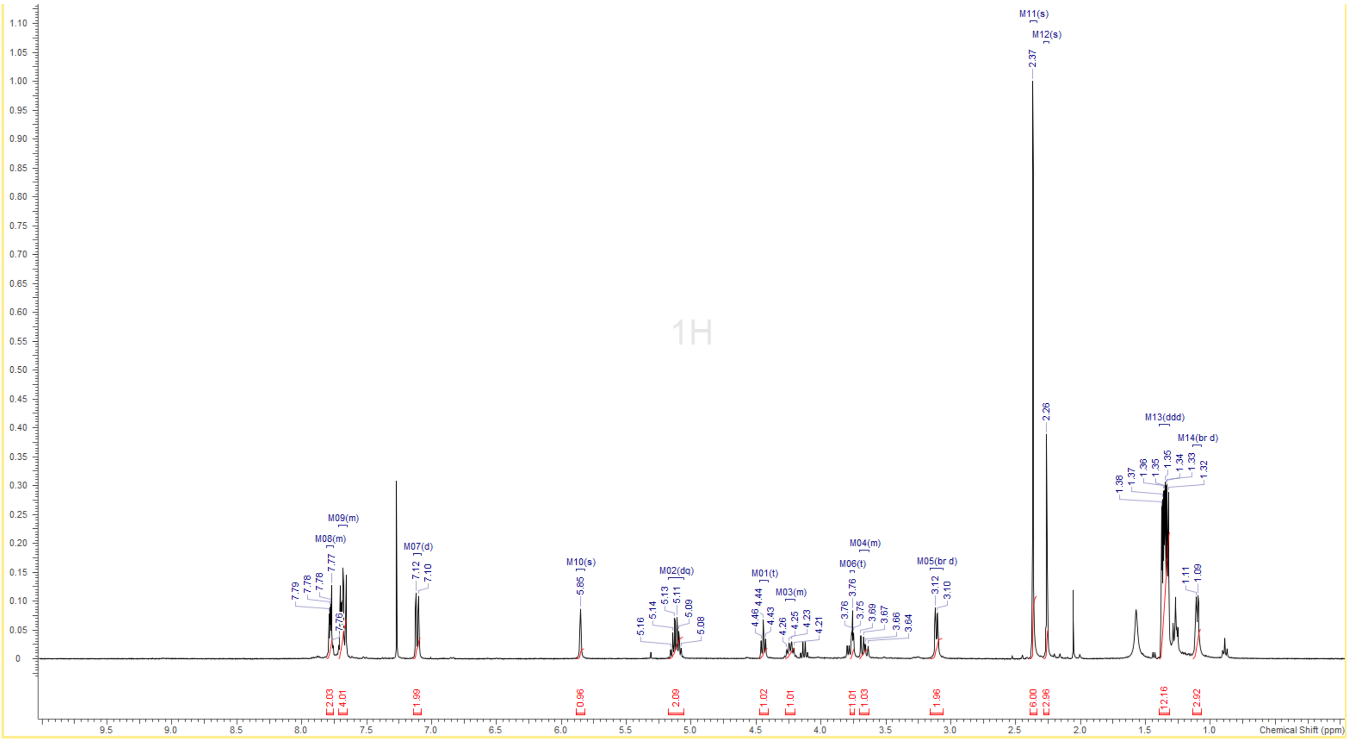

Diisopropyl 4-(((N-(1-aminopropan-2-yl)-4-methylphenyl)sulfonamido)methyl)-2,6-dimethyl-1,4-dihydropyridine-3,5-dicarboxylate (1p)

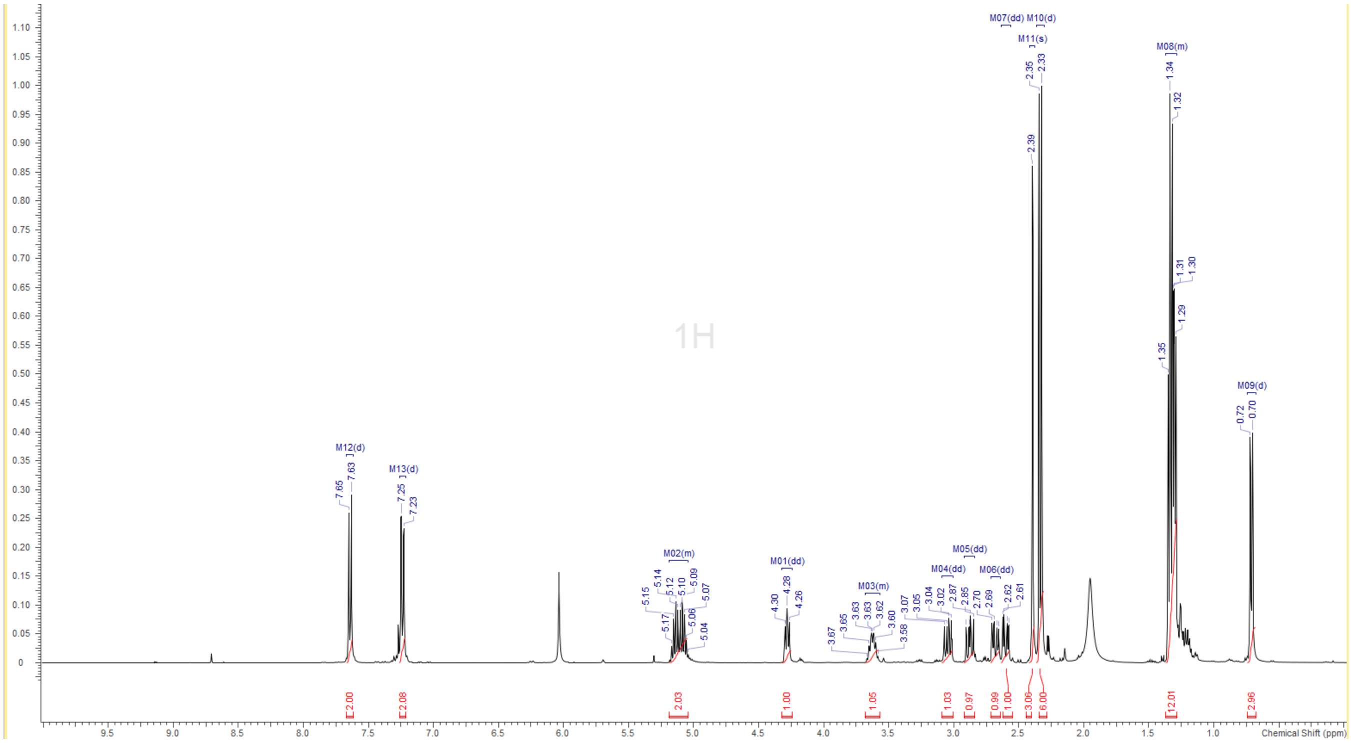

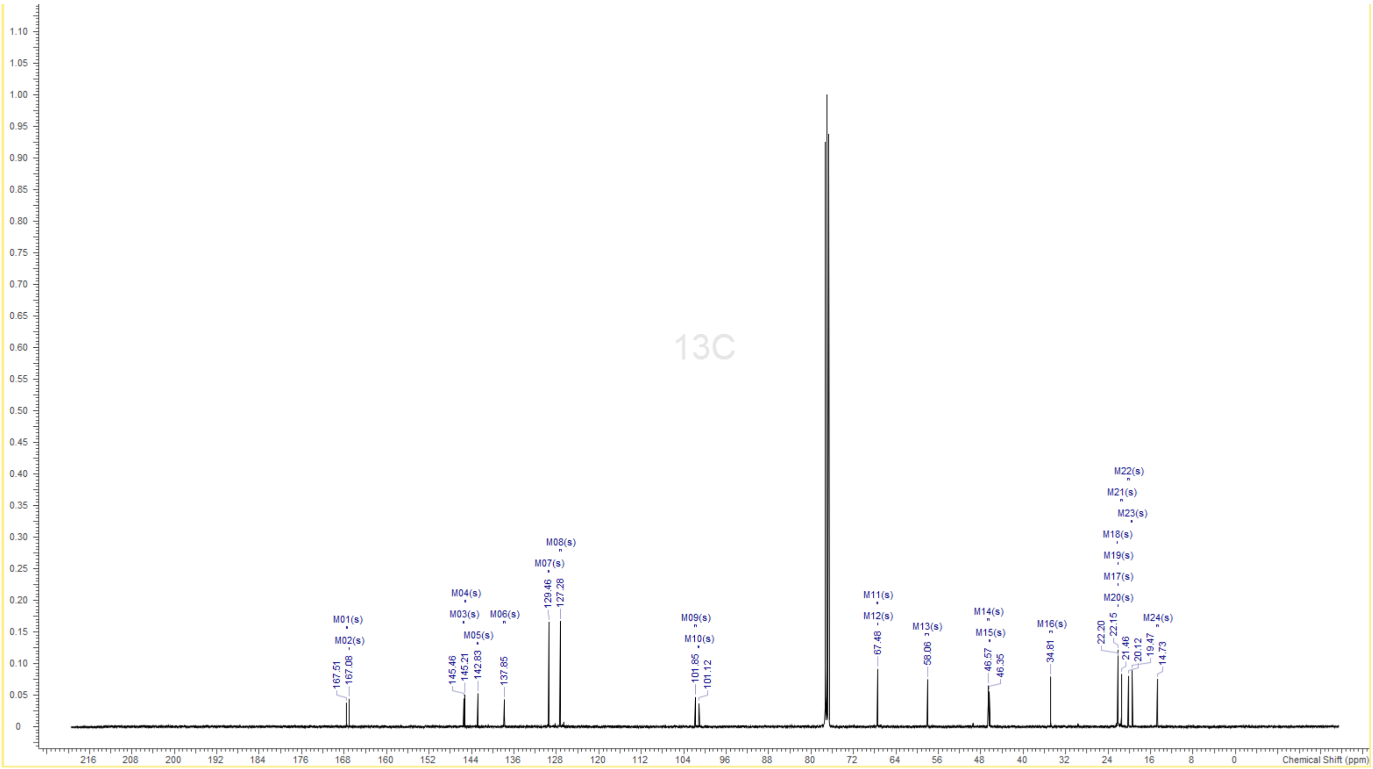
13C
167.51
167.08
145.46
145.21
142.83
137.85
129.46
127.28
101.85
101.12
67.48
58.06
46.57
46.35
34.81
22.20
22.15
21.46
20.12
19.47
14.73
Chemical Shift (ppm)

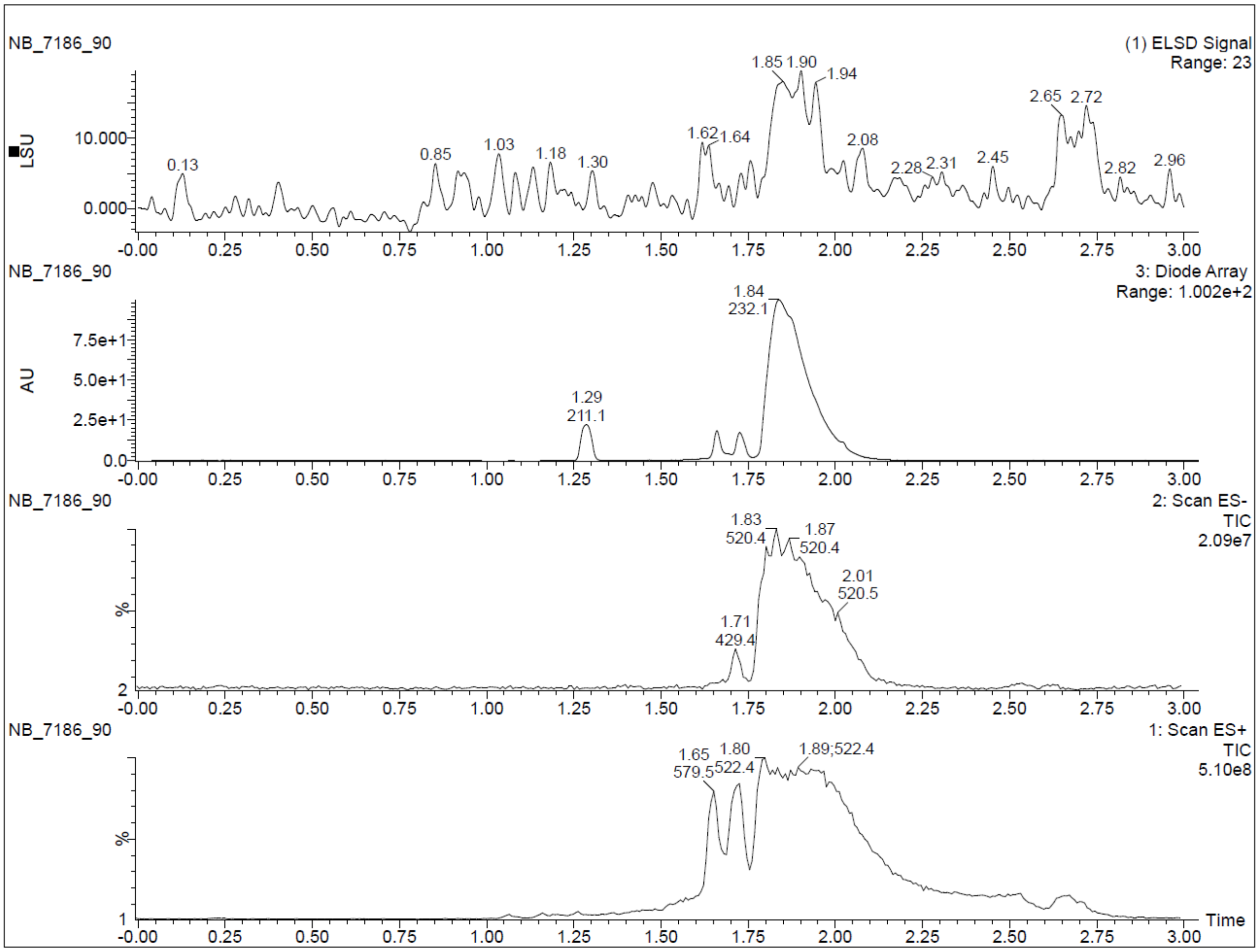
NB_7186_90
(1) ELSD Signal
Range: 23
LSU
NB_7186_90
3: Diode Array
Range: 1.002e+2
AU
1.84
232.1
1.29
211.1
NB_7186_90
2: Scan ES-
TIC
2.09e7
1.83
520.4
1.87
520.4
2.01
520.5
1.71
429.4
NB_7186_90
1: Scan ES+
TIC
5.10e8
1.65
579.5
1.80
522.4
1.89;522.4
Time

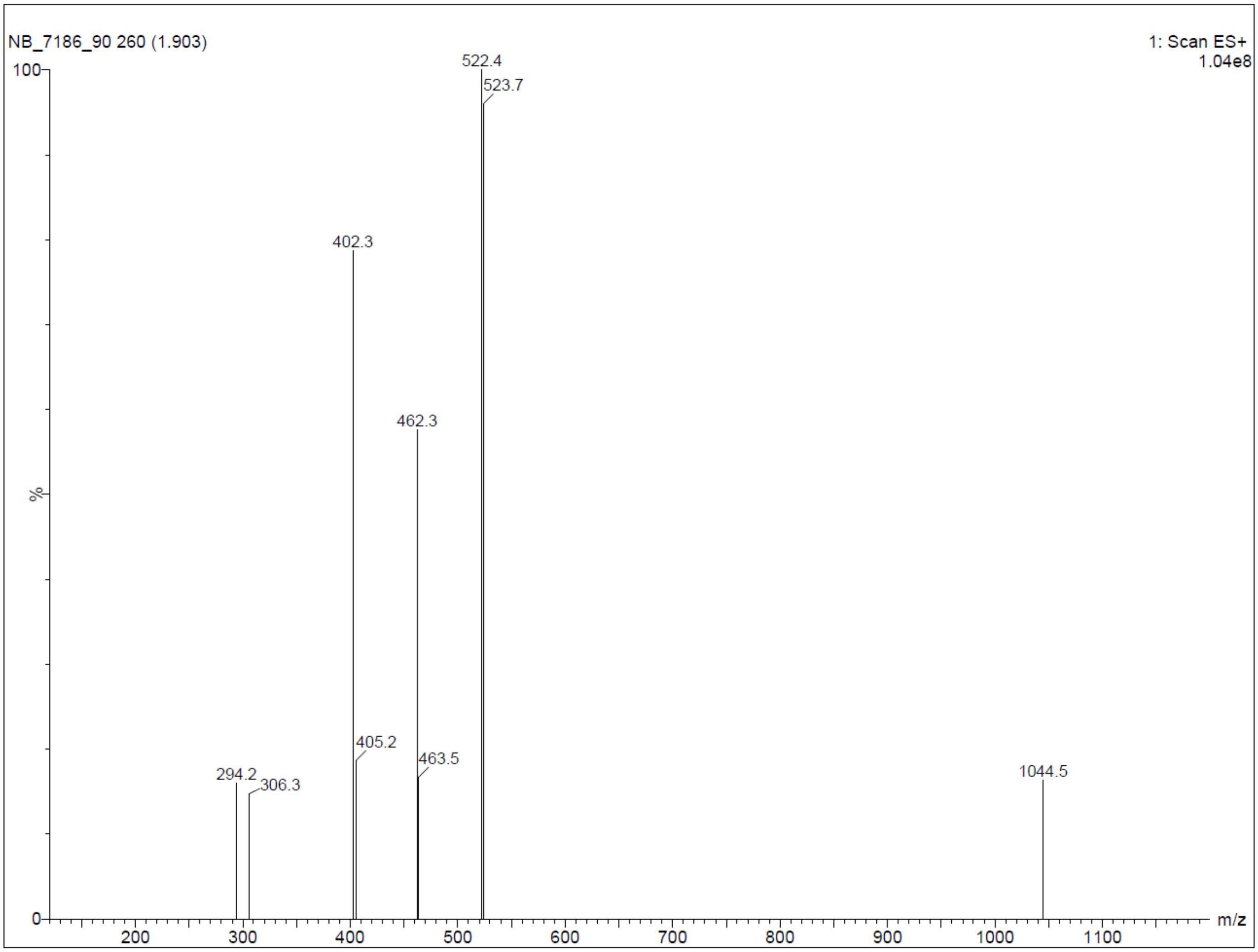
NB_7186_90 260 (1.903)
1: Scan ES+
1.04e8
100
%
0
522.4
523.7
402.3
462.3
405.2
463.5
294.2
306.3
1044.5
200
300
400
500
600
700
800
900
1000
1100
m/z

N-Heterocyclic Products

3-(4-Methoxyphenyl)morpholine, TFA salt (4aa)

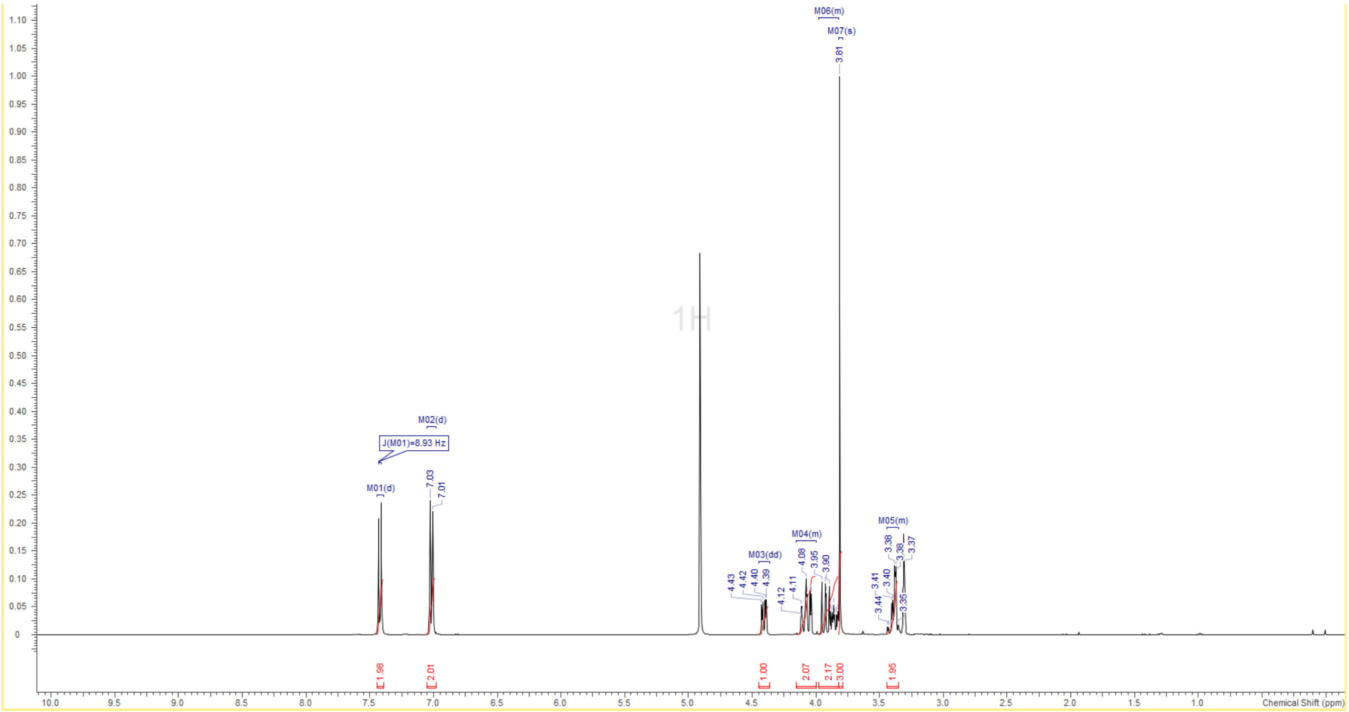

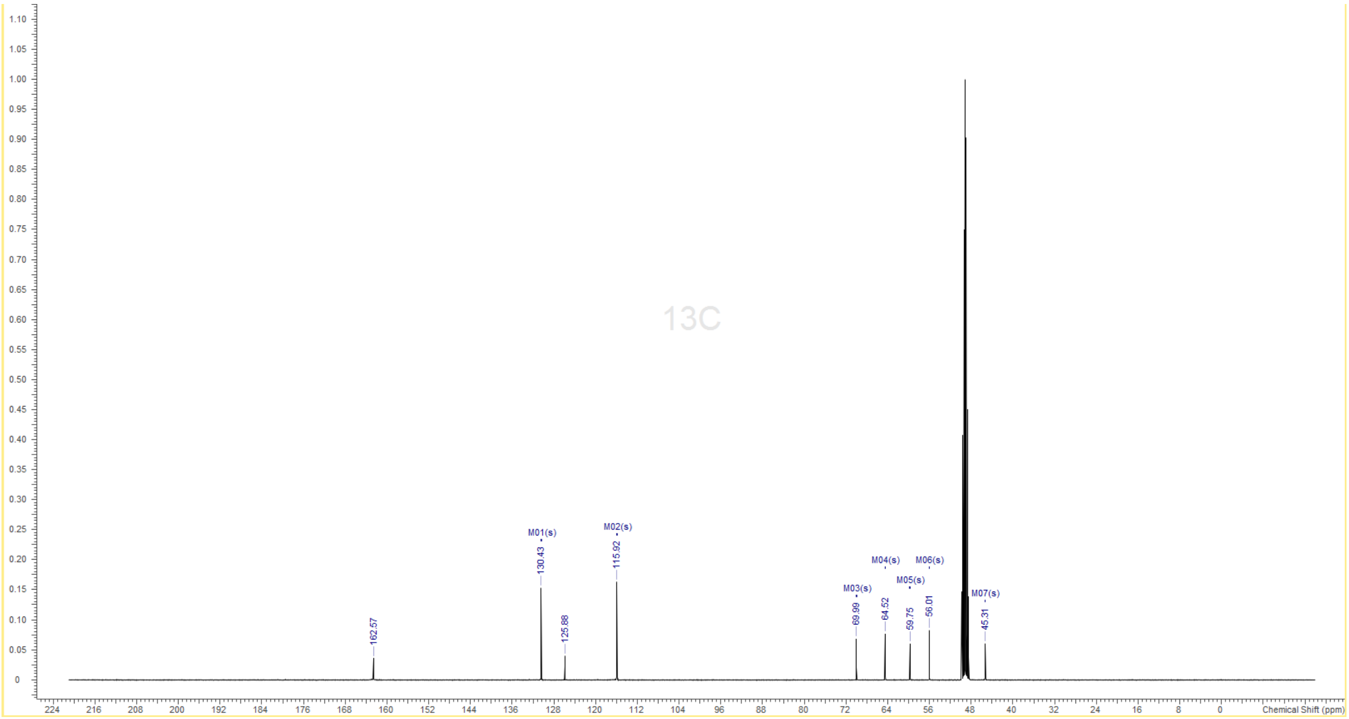

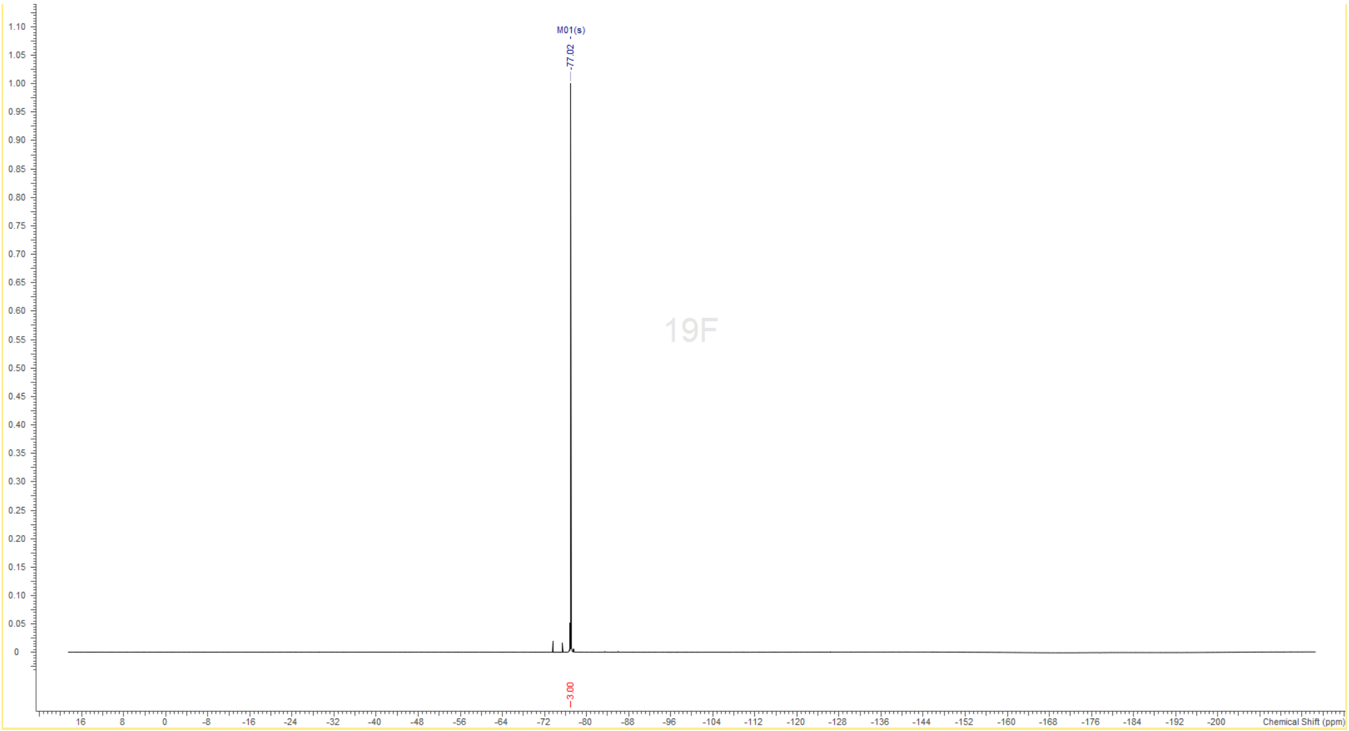
M01(s)
-77.02
19F
3.00
Chemical Shift (ppm)

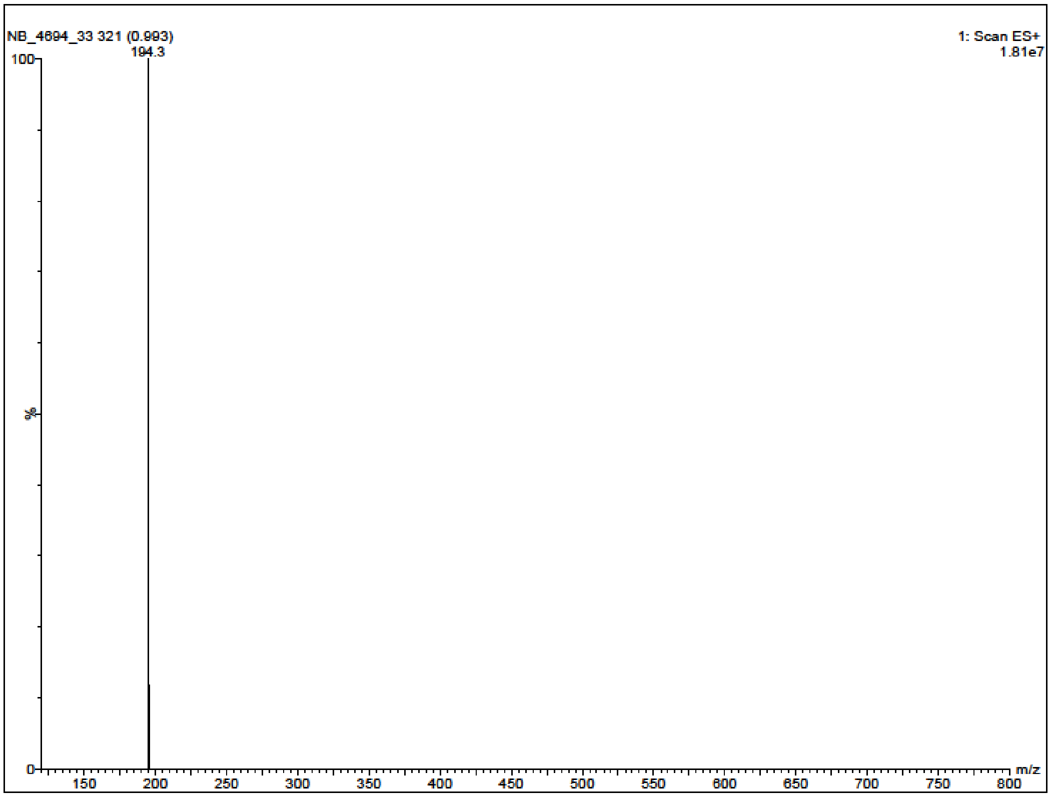
NB_4694_33 321 (0.993)
1: Scan ES+
1.81e7
194.3
100
%
0
150
200
250
300
350
400
450
500
550
600
650
700
750
800
m/z

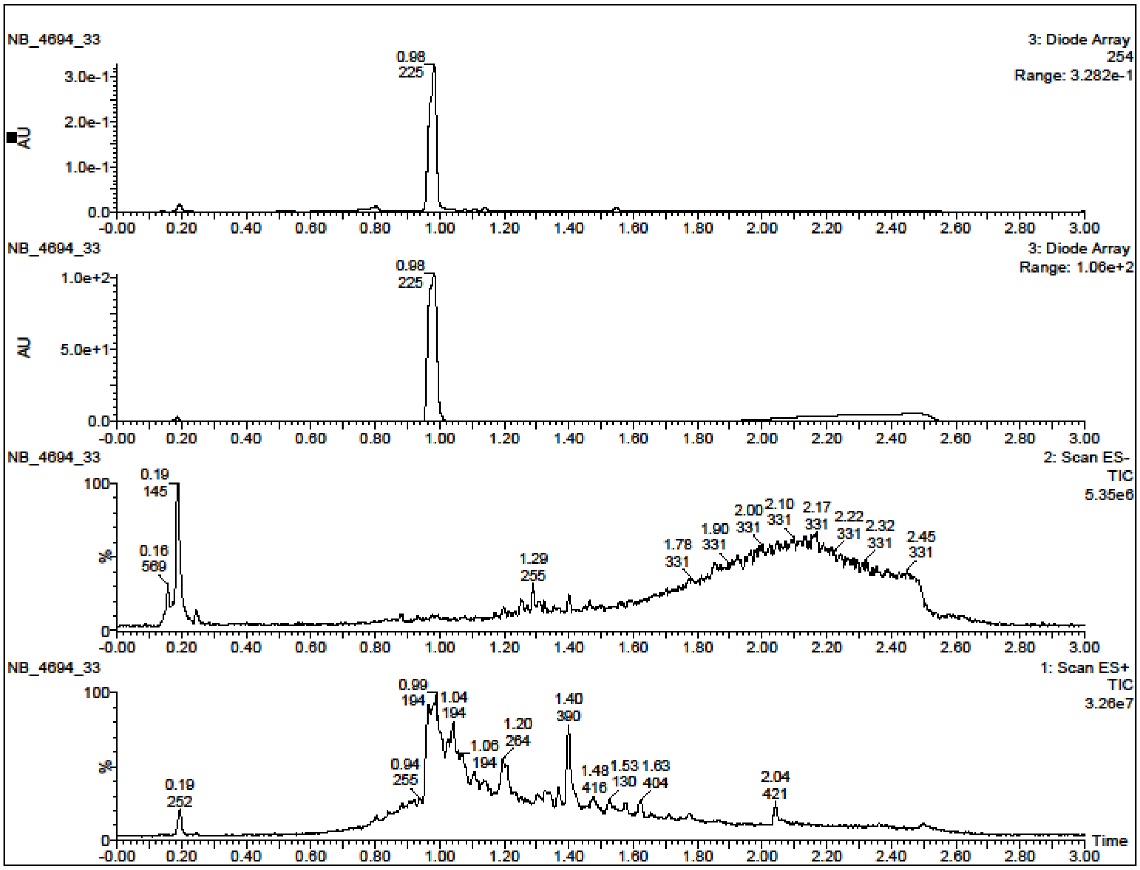
NB_4694_33
3: Diode Array
254
Range: 3.282e-1
0.98
225
3.0e-1
2.0e-1
1.0e-1
0.0
AU
NB_4694_33
3: Diode Array
Range: 1.06e+2
1.0e+2
5.0e+1
NB_4694_33
2: Scan ES-
TIC
5.35e6
0.19
145
0.16
569
1.29
255
1.78
331
1.90
331
2.00
331
2.10
331
2.17
331
2.22
331
2.32
331
2.45
331
NB_4694_33
1: Scan ES+
TIC
3.26e7
0.99
194
1.04
194
1.06
194
1.20
264
1.40
390
0.94
255
1.48
416
1.53
130
1.63
404
2.04
421
0.19
252
-0.00
0.20
0.40
0.60
0.80
1.00
1.20
1.40
1.60
1.80
2.00
2.20
2.40
2.60
2.80
3.00
Time

3-(4-Bromophenyl)morpholine, TFA salt (4ad)

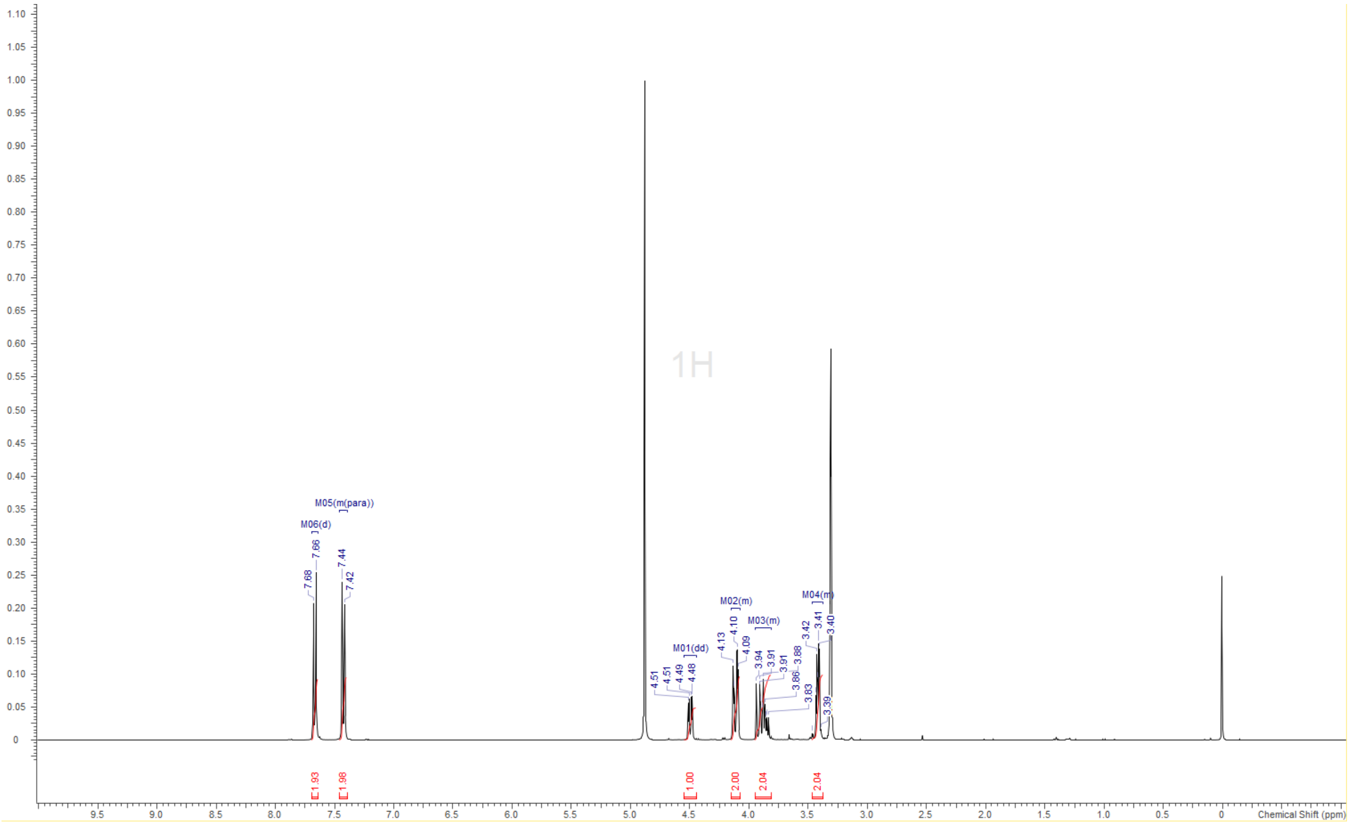

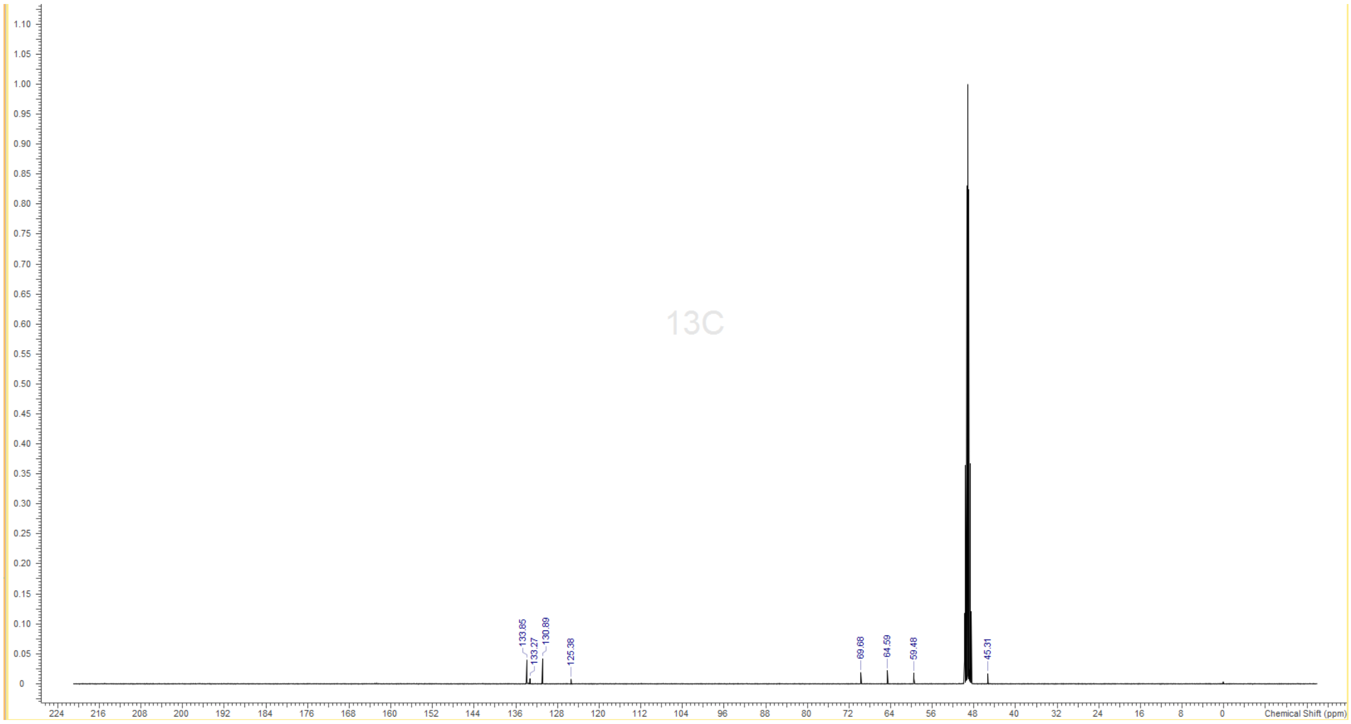

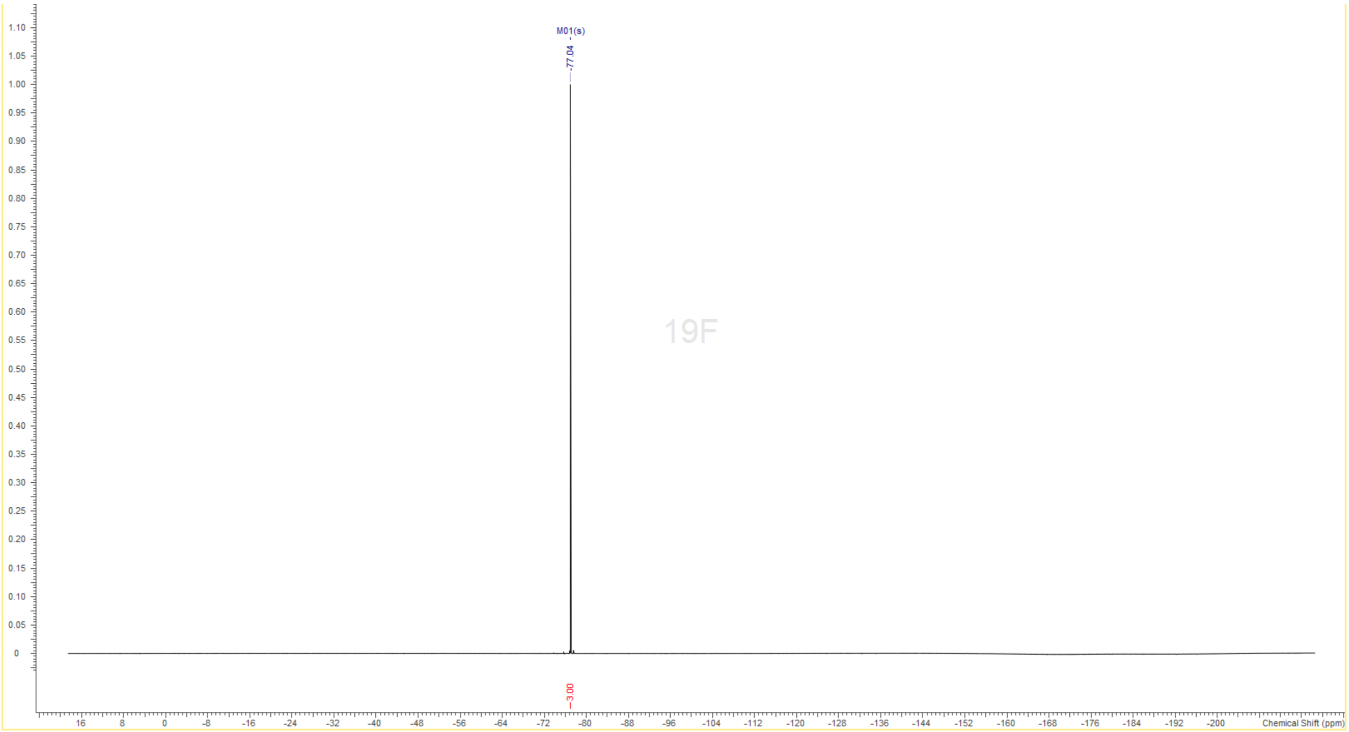
M01(s)
-77.04
19F
3.00
Chemical Shift (ppm)

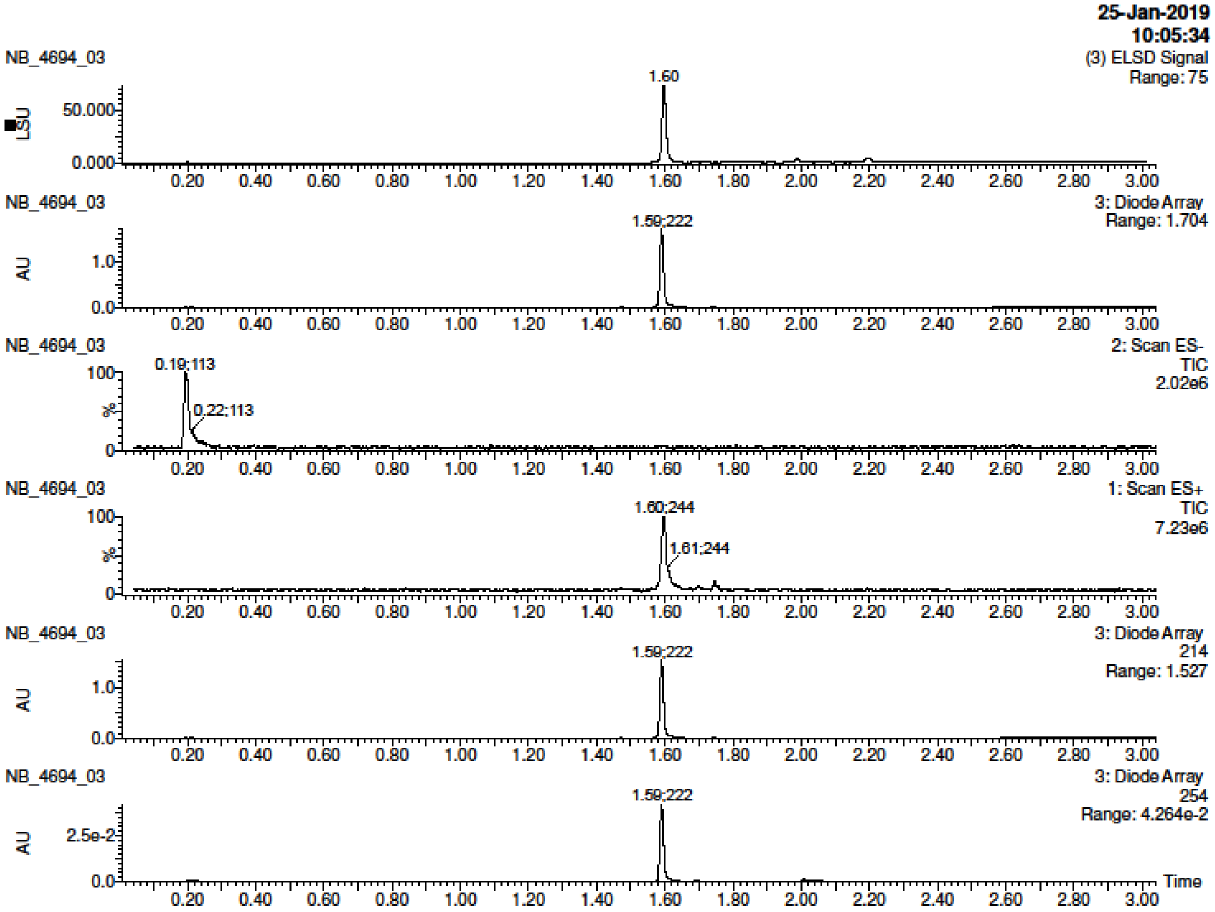
25-Jan-2019
10:05:34
NB_4694_03
(3) ELSD Signal
Range: 75
LSU
1.60
3: Diode Array
Range: 1.704
AU
1.59;222
2: Scan ES-
TIC
2.02e6
0.19;113
0.22;113
1: Scan ES+
TIC
7.23e6
1.60;244
1.61;244
3: Diode Array
214
Range: 1.527
3: Diode Array
254
Range: 4.264e-2
Time

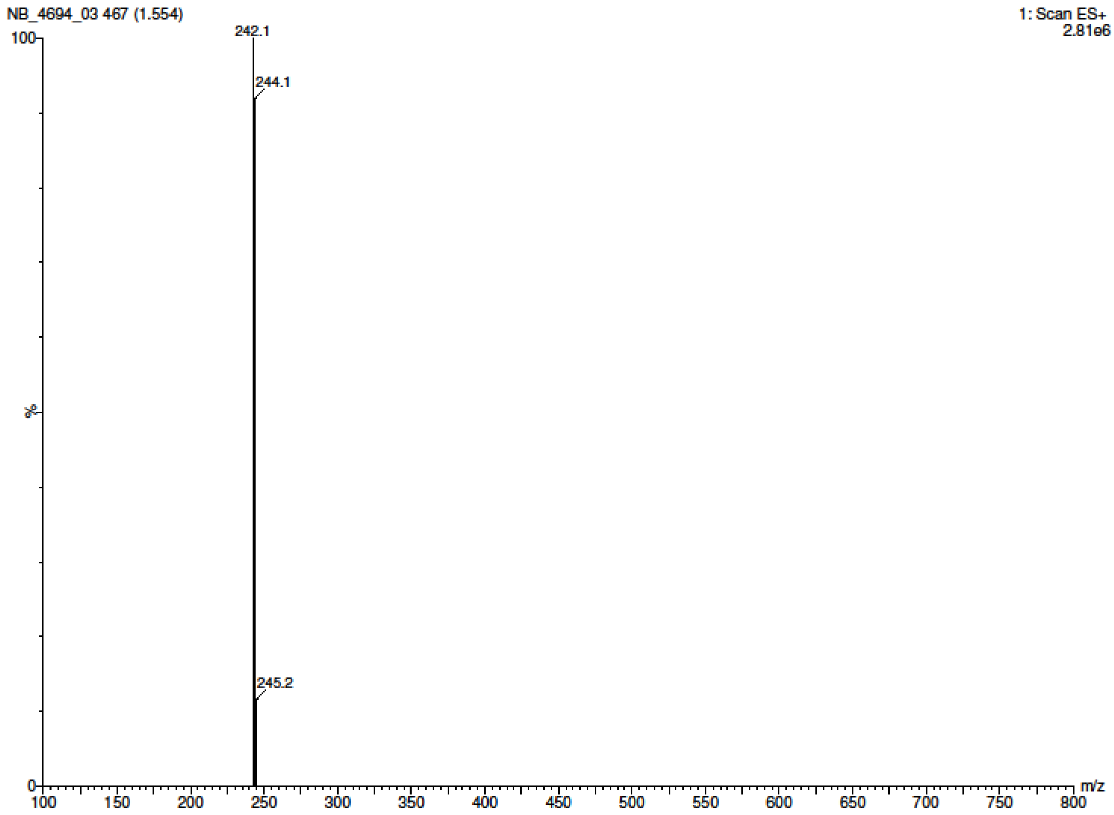

3-(4-Fluorophenyl)morpholine, TFA salt (4ac)

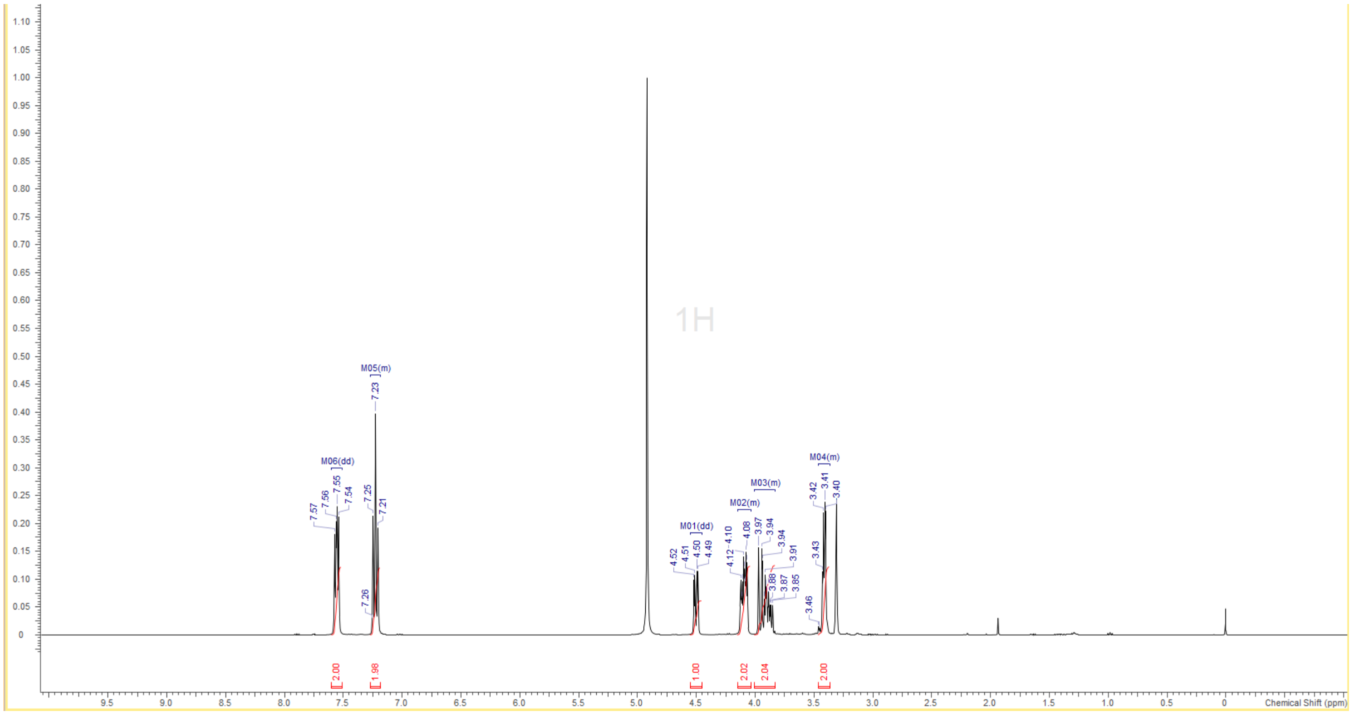

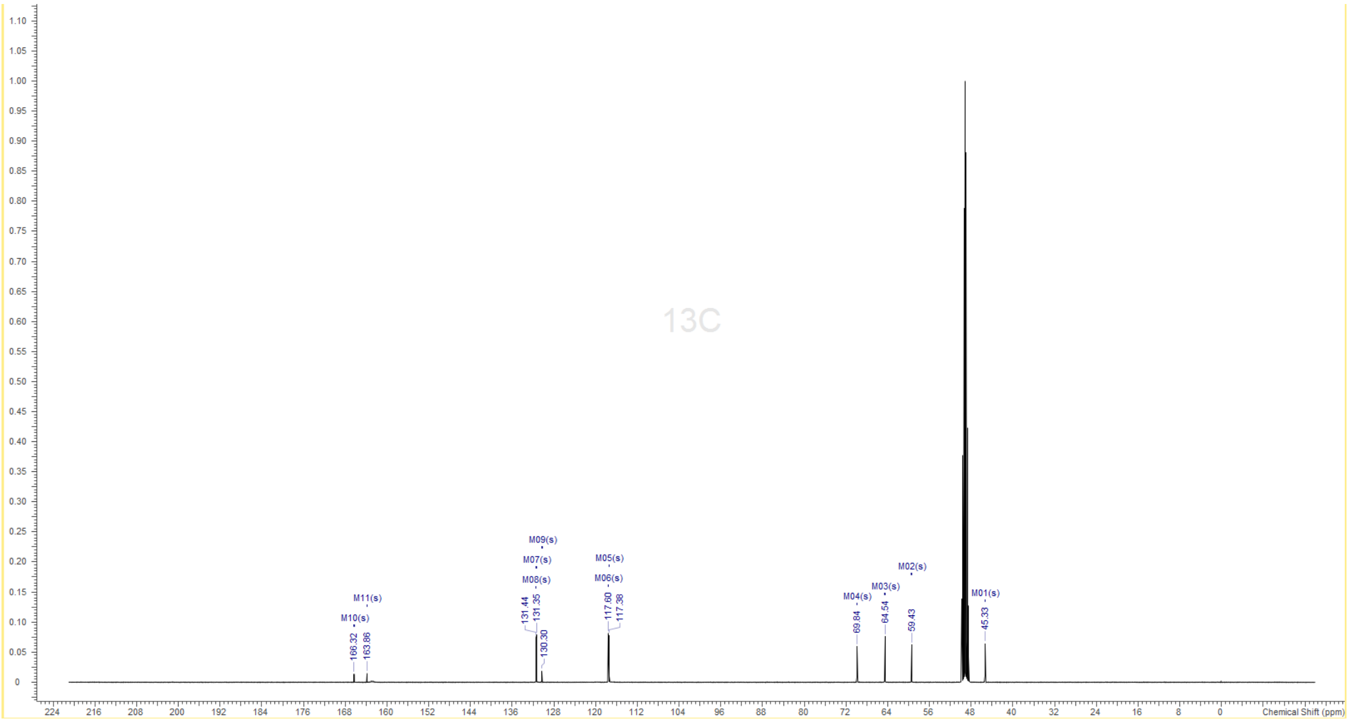
13C
M10(s)
166.32
M11(s)
163.86
M09(s)
M07(s)
M08(s)
131.44
131.35
130.30
M05(s)
M06(s)
117.60
117.38
M04(s)
69.84
M03(s)
64.54
M02(s)
59.43
M01(s)
45.33
Chemical Shift (ppm)

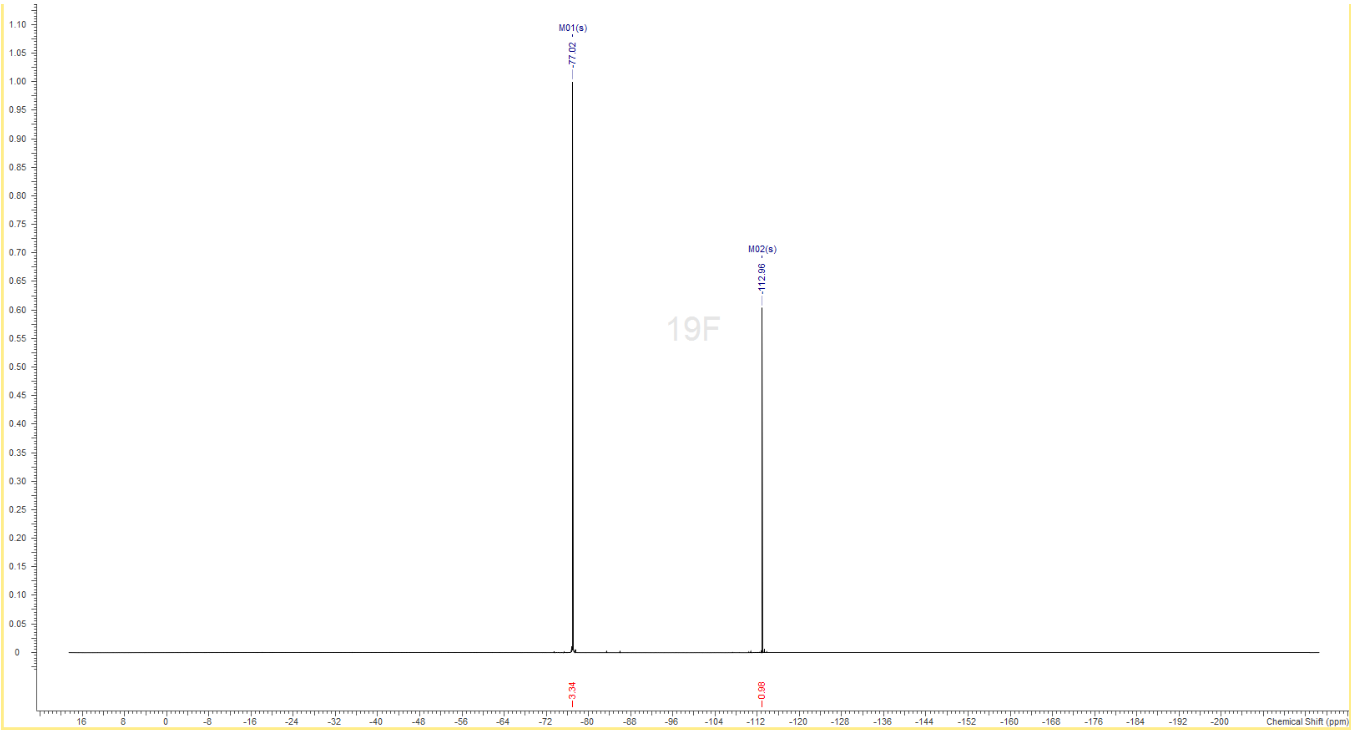
19F
M01(s)
-77.02
M02(s)
-112.96
3.34
0.98
Chemical Shift (ppm)

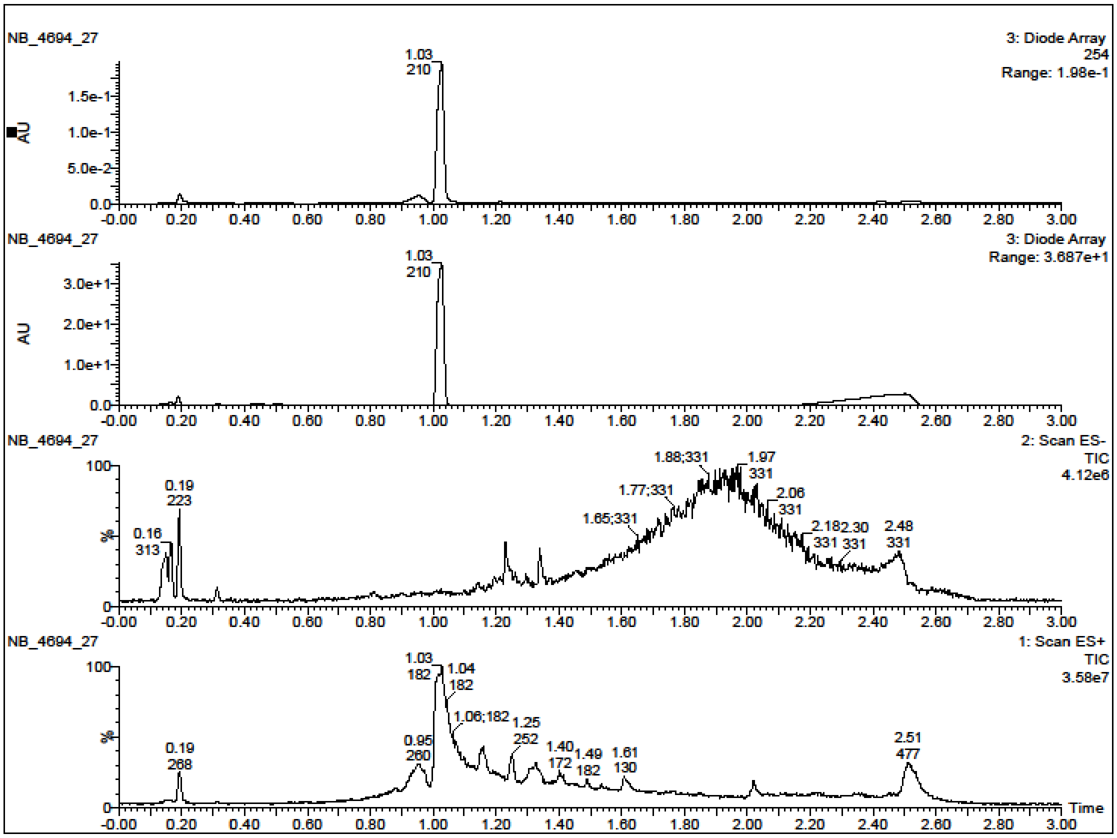
NB_4694_27
3: Diode Array
254
Range: 1.98e-1
1.03
210
AU
NB_4694_27
3: Diode Array
Range: 3.687e+1
1.03
210
AU
NB_4694_27
2: Scan ES-
TIC
4.12e6
0.19
223
0.16
313
1.88;331
1.97
331
1.77;331
1.65;331
2.06
331
2.18
331
2.30
331
2.48
331
%
NB_4694_27
1: Scan ES+
TIC
3.58e7
1.03
182
1.04
182
1.06;182
1.25
252
0.95
260
1.40
172
1.49
182
1.61
130
0.19
268
2.51
477
%
Time

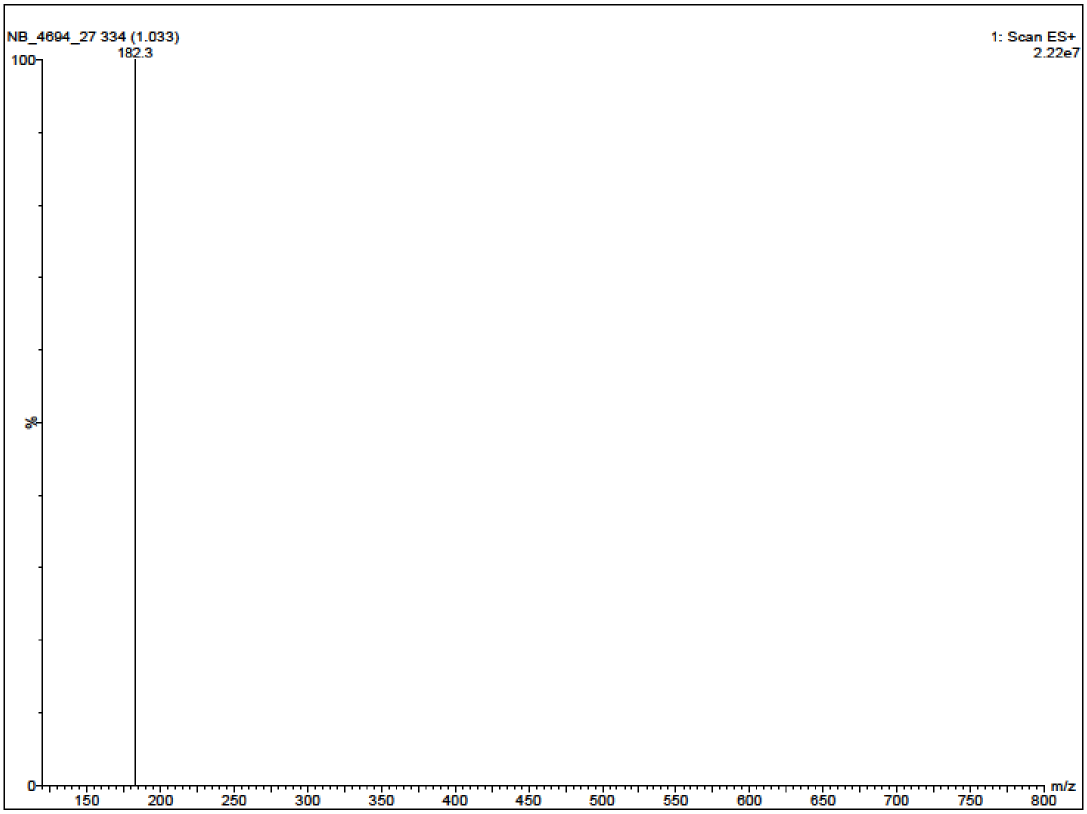
NB_4694_27 334 (1.033)
1: Scan ES+
2.22e7
182.3
%
m/z

3-(p-Tolyl)morpholine, TFA salt (4ab)

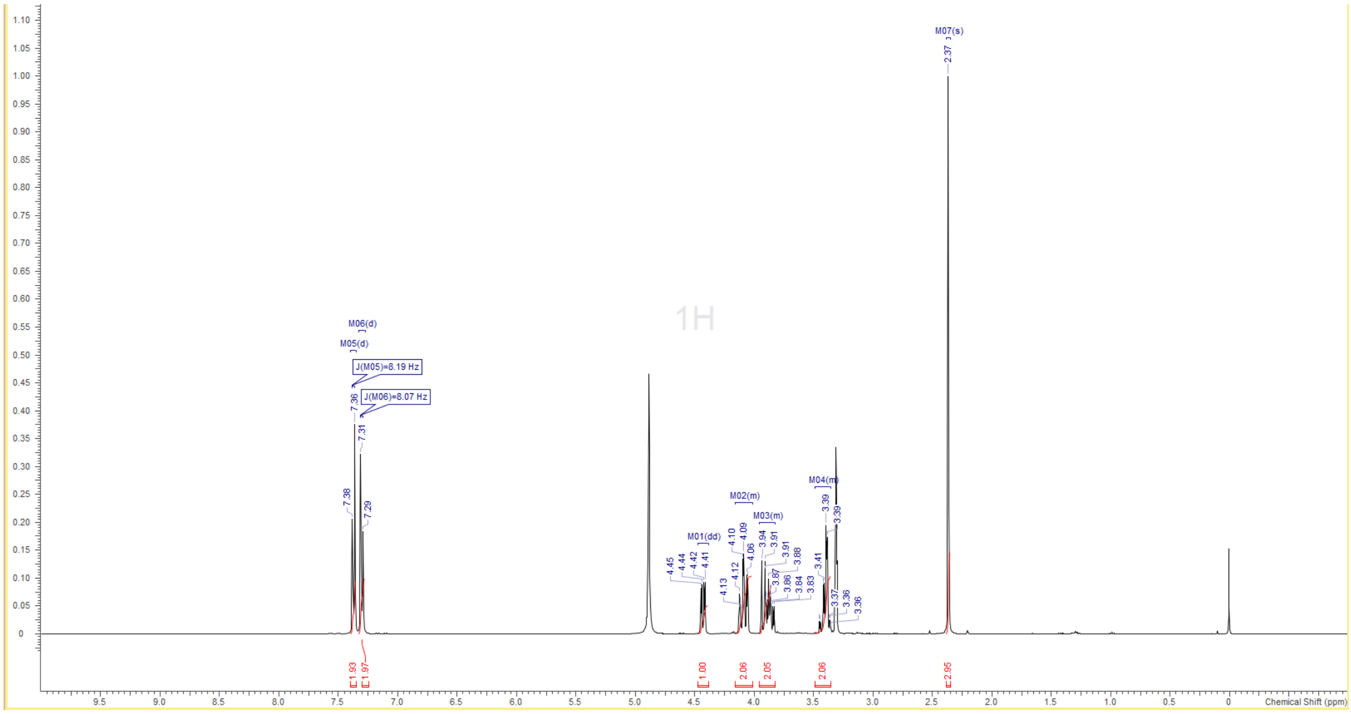

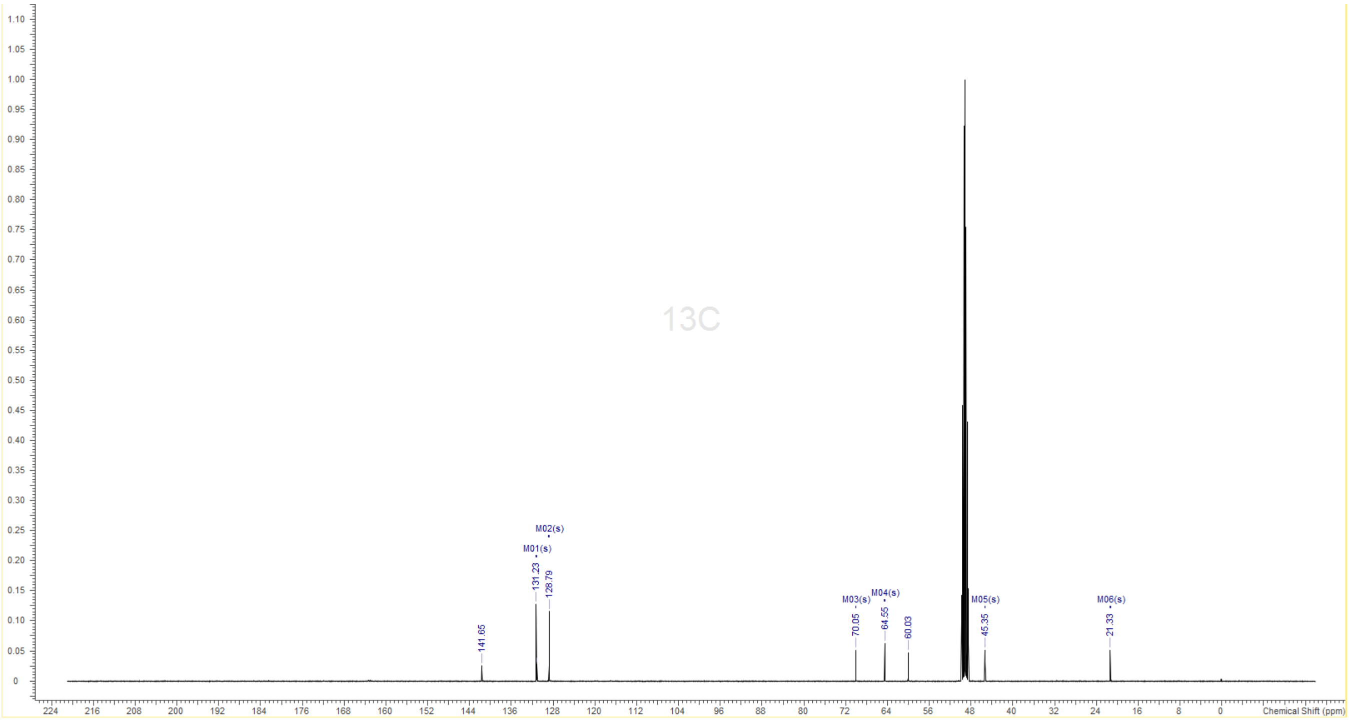

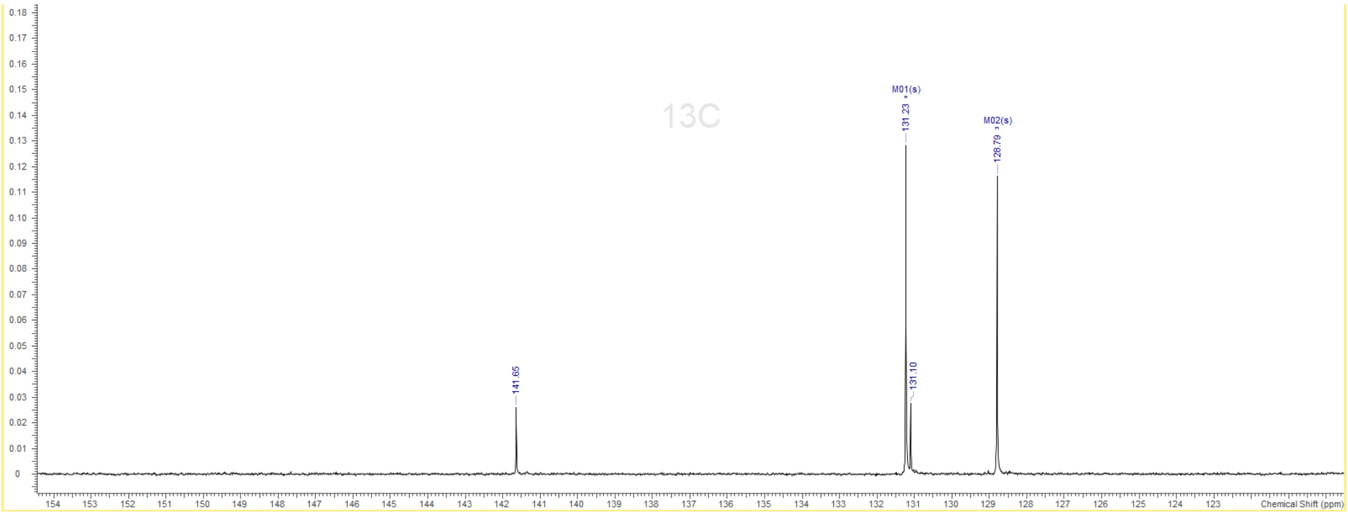

Aromatic Region (Zoomed)

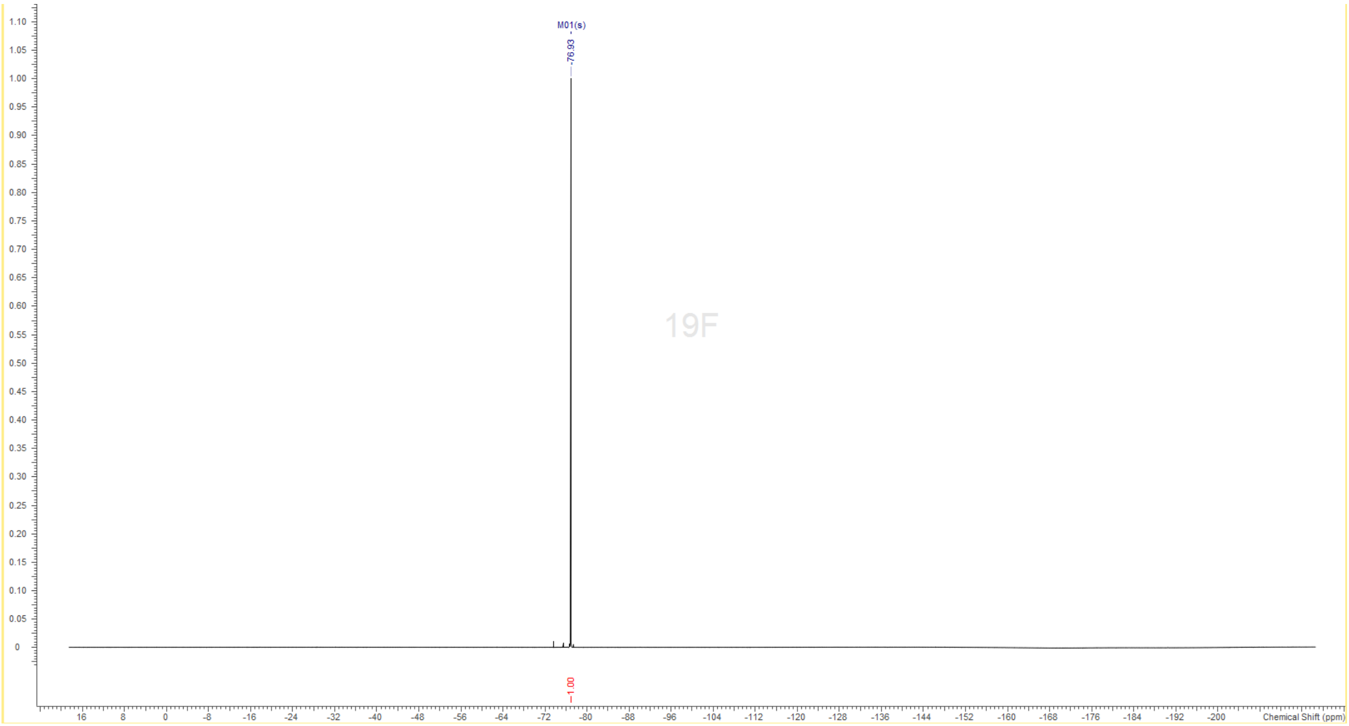

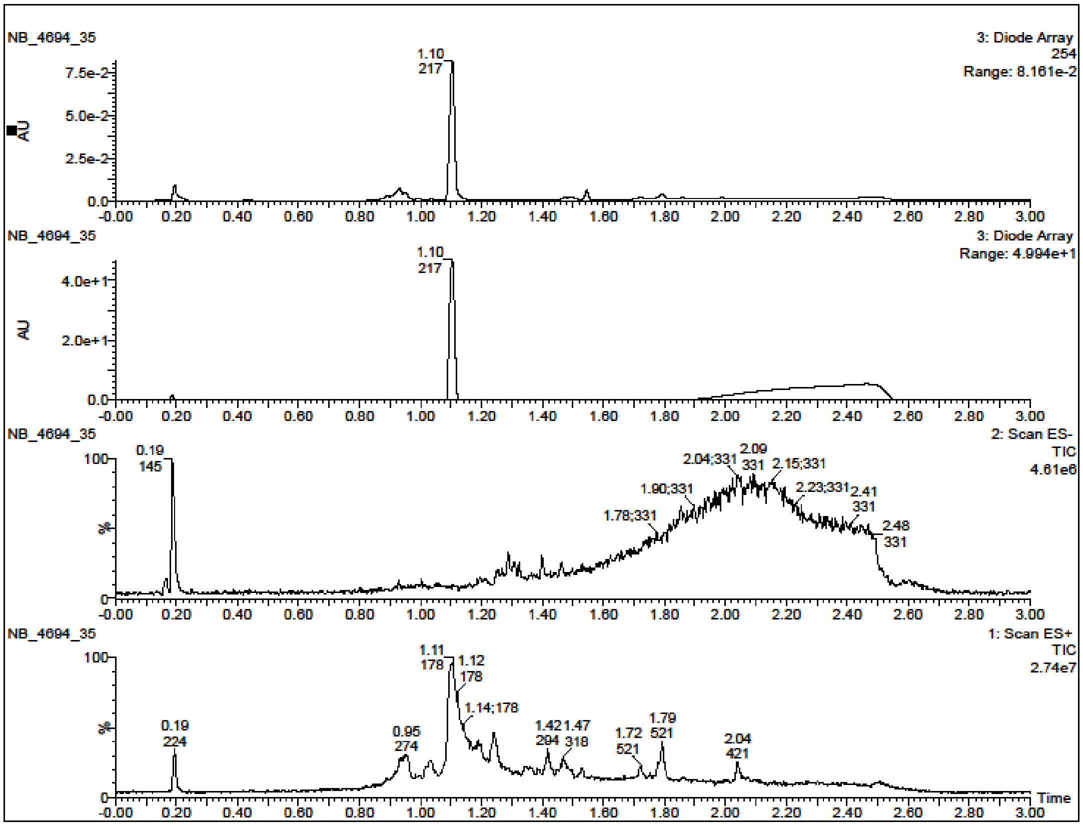
NB_4694_35
3: Diode Array
254
Range: 8.161e-2
1.10
217
NB_4694_35
3: Diode Array
Range: 4.994e+1
NB_4694_35
2: Scan ES-
TIC
4.61e6
NB_4694_35
1: Scan ES+
TIC
2.74e7
Time

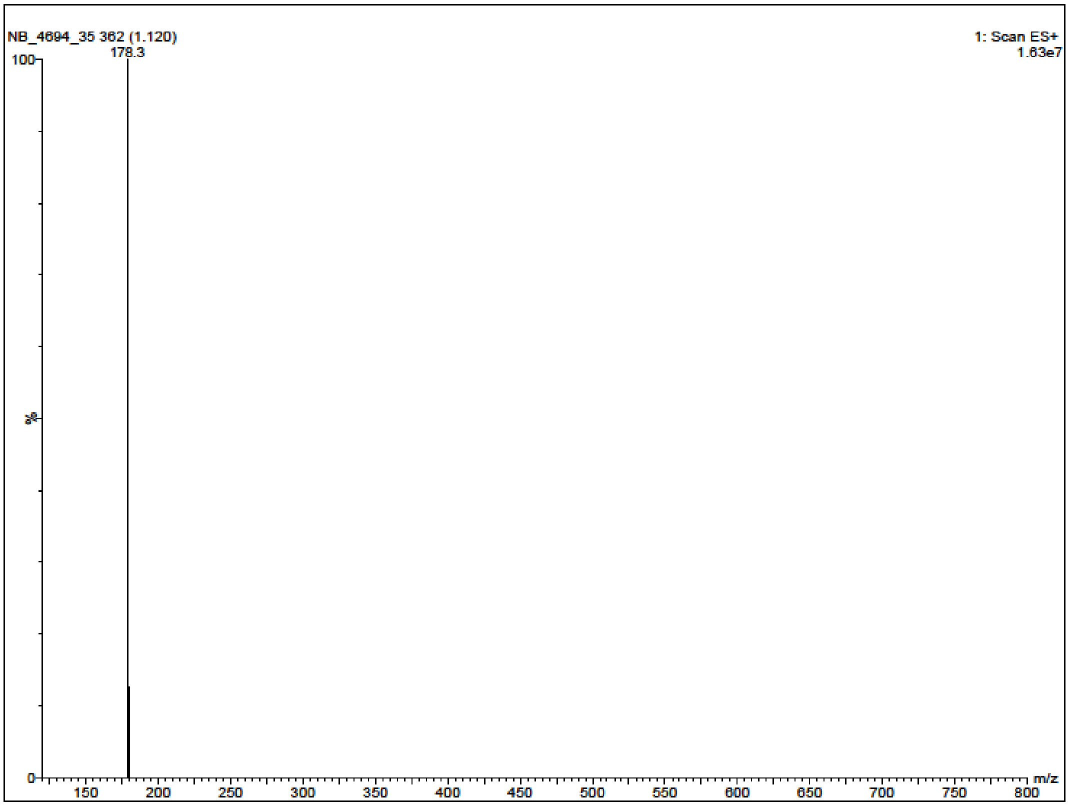
NB_4694_35 362 (1.120)
1: Scan ES+
1.63e7
178.3
m/z

3-(Thieno[2,3-b]pyridin-2-yl)morpholine, TFA salt (4av)

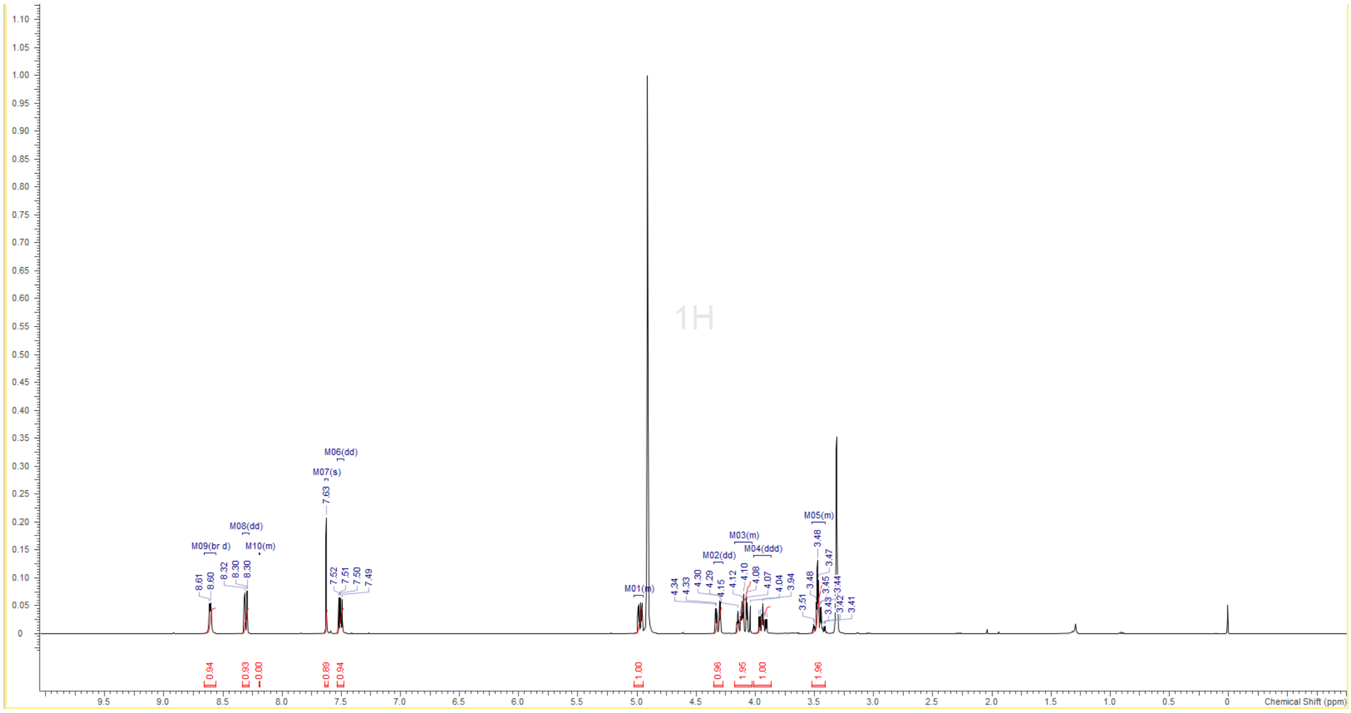

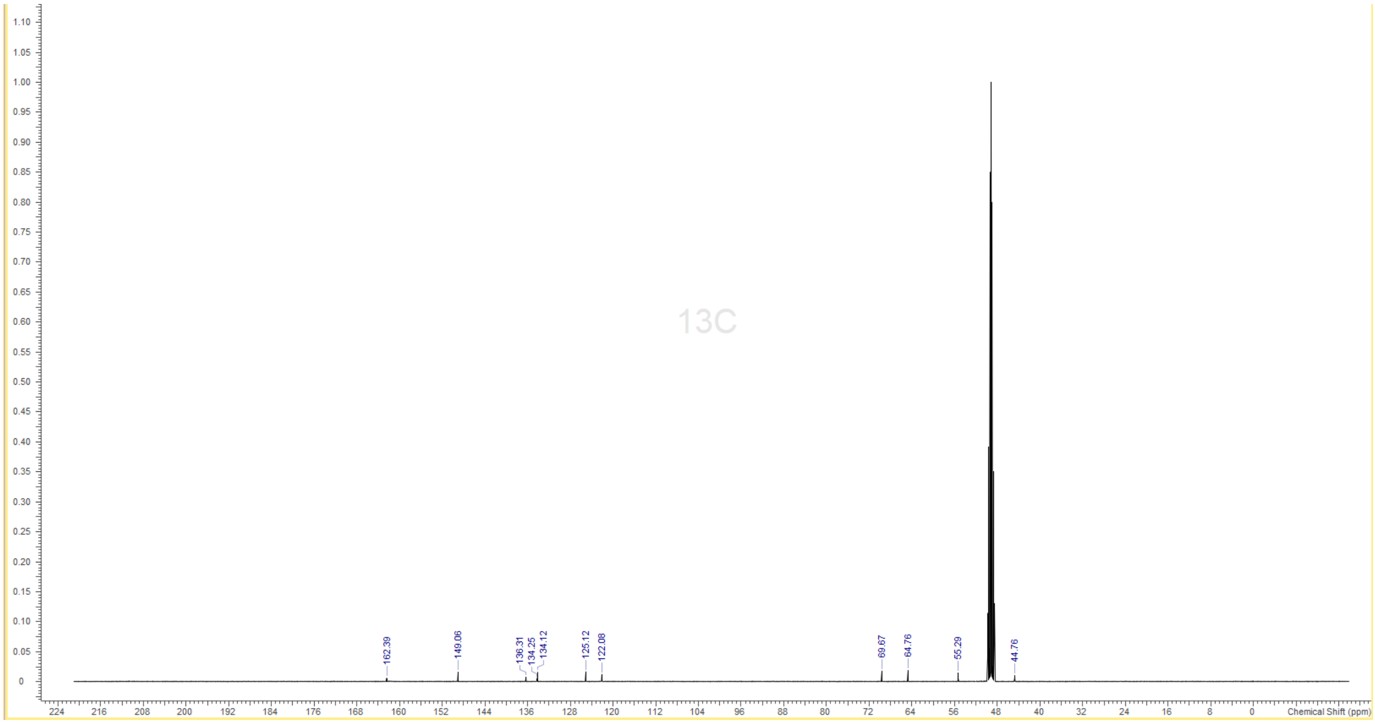

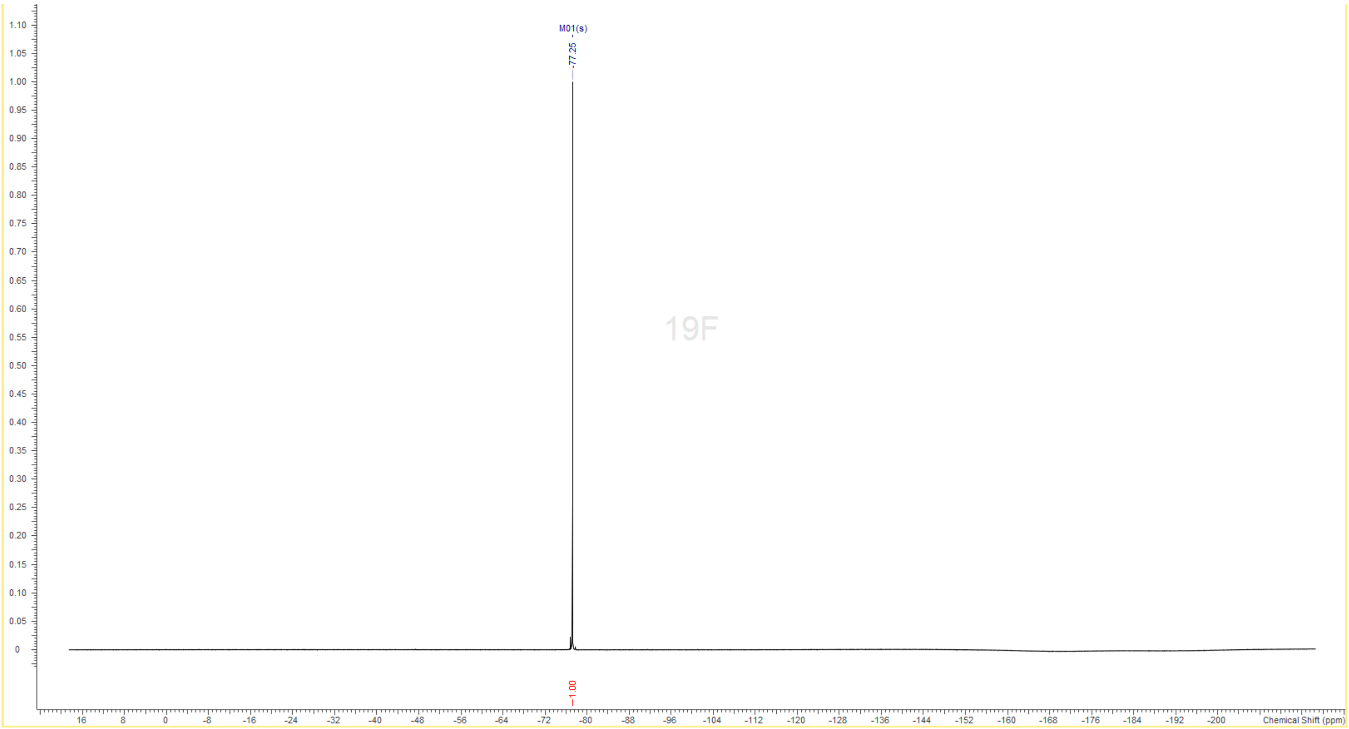
M01(s)
-77.25
19F
1.00
Chemical Shift (ppm)

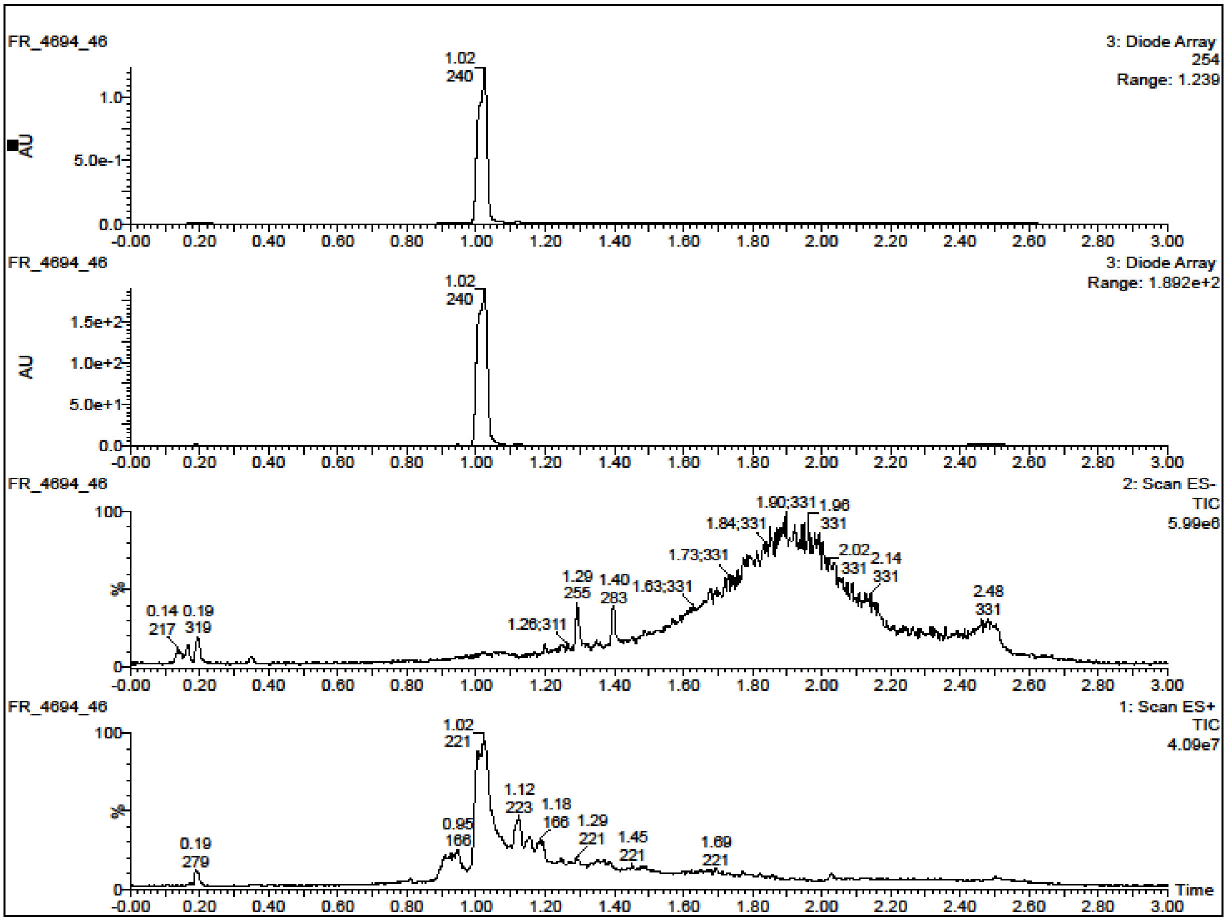
FR_4694_46
3: Diode Array
254
Range: 1.239
1.02
240
AU
FR_4694_46
3: Diode Array
Range: 1.892e+2
1.02
240
AU
FR_4694_46
2: Scan ES-
TIC
5.99e6
1.90;331
1.96
331
1.84;331
1.73;331
2.02
331
2.14
331
1.29
255
1.40
283
1.63;331
2.48
331
0.14 0.19
217 319
1.26;311
%
FR_4694_46
1: Scan ES+
TIC
4.09e7
1.02
221
1.12
223
1.18
166
1.29
221
0.95
166
1.45
221
1.69
221
0.19
279
%
Time

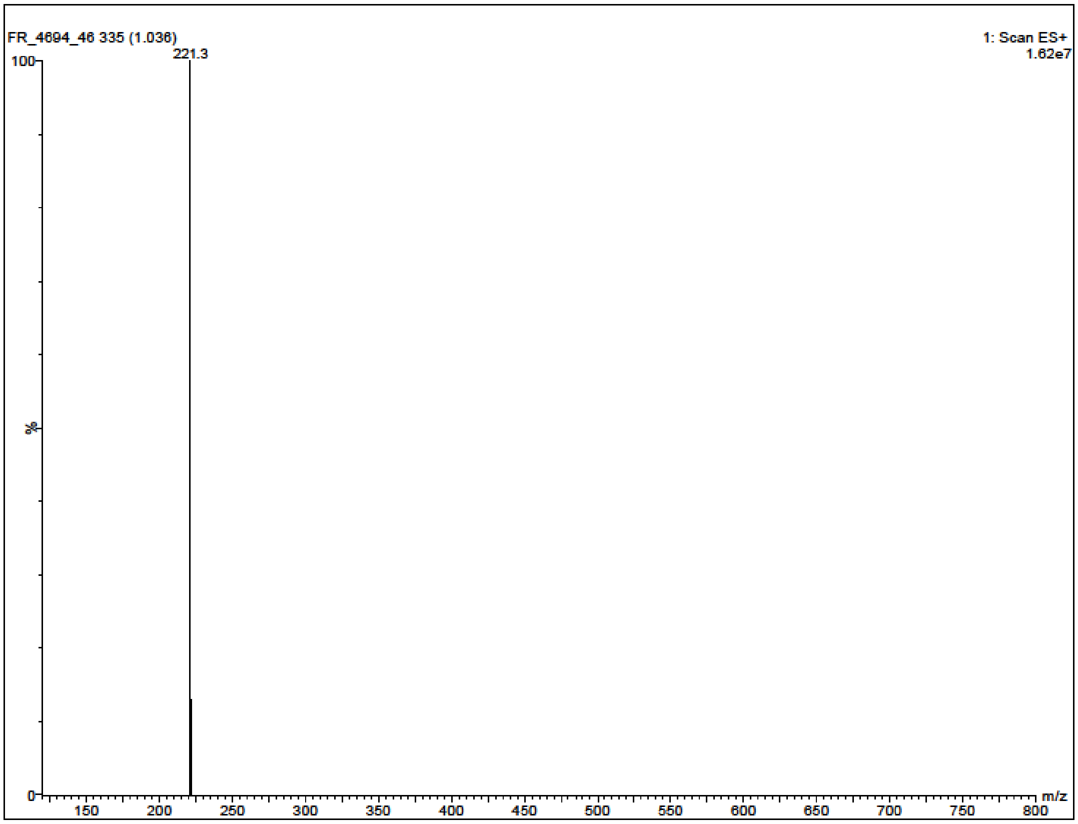

3-(4-(Trifluoromethoxy)phenyl)morpholine, TFA salt (4ag)

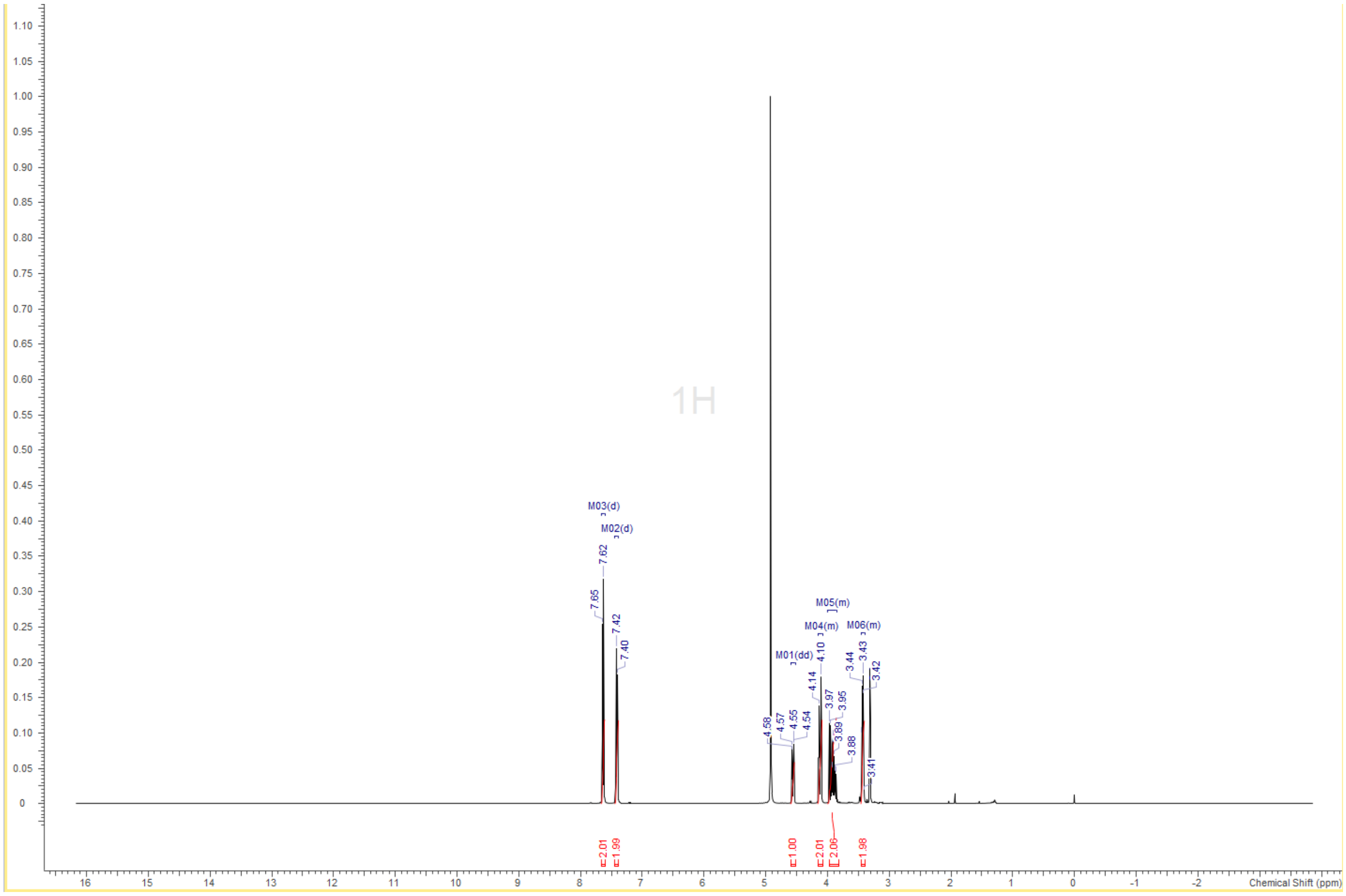

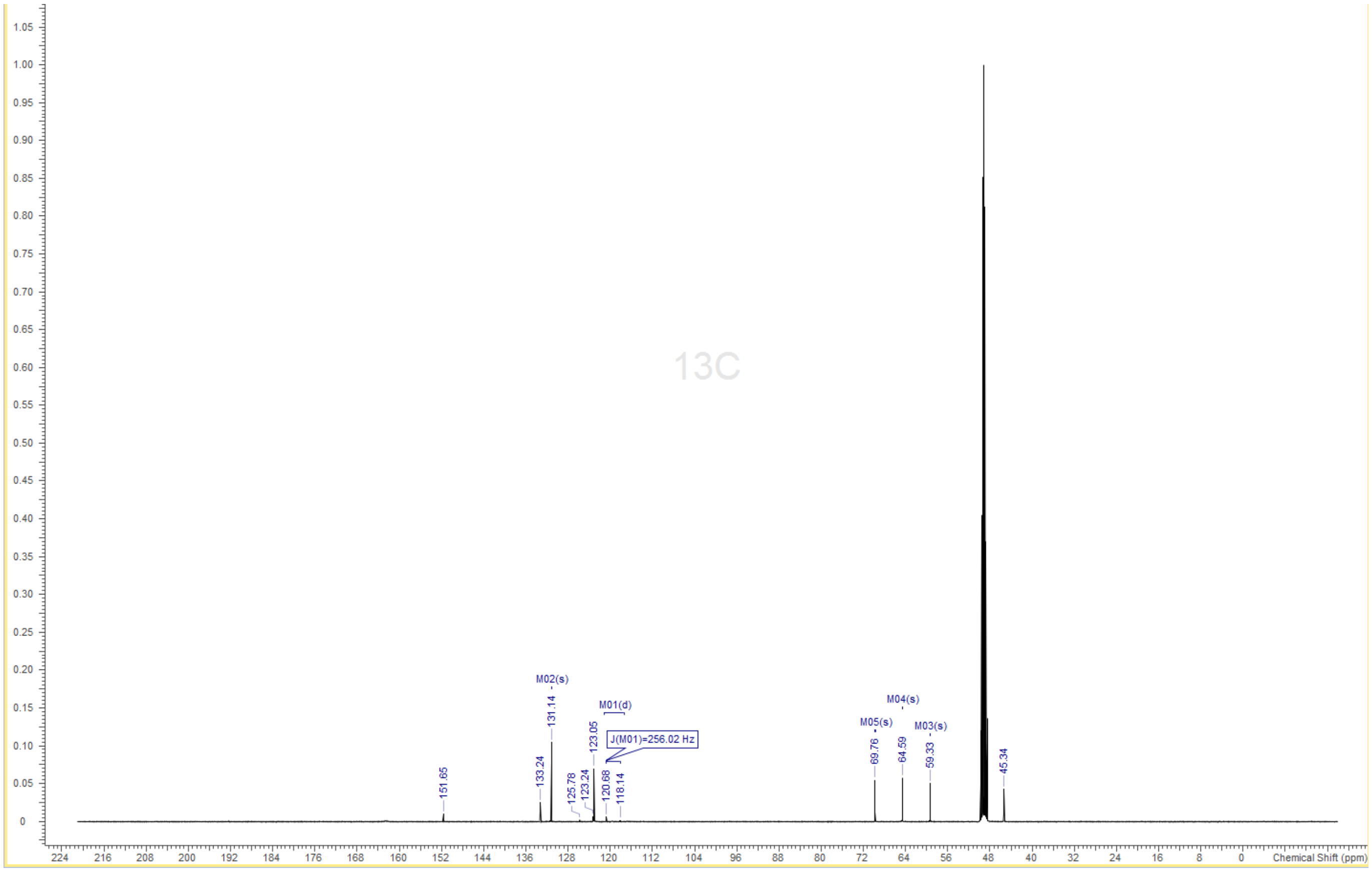

Aromatic Region (zoomed):

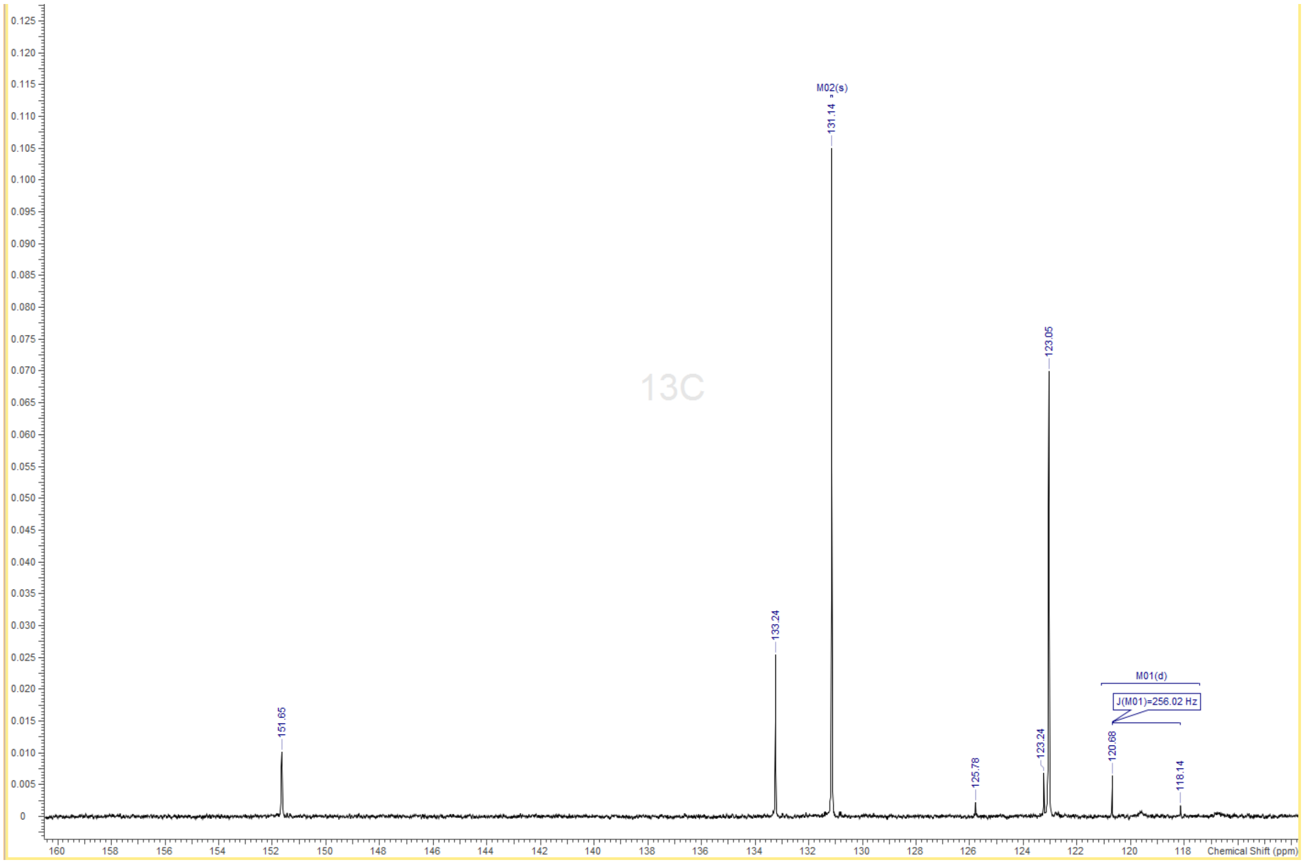

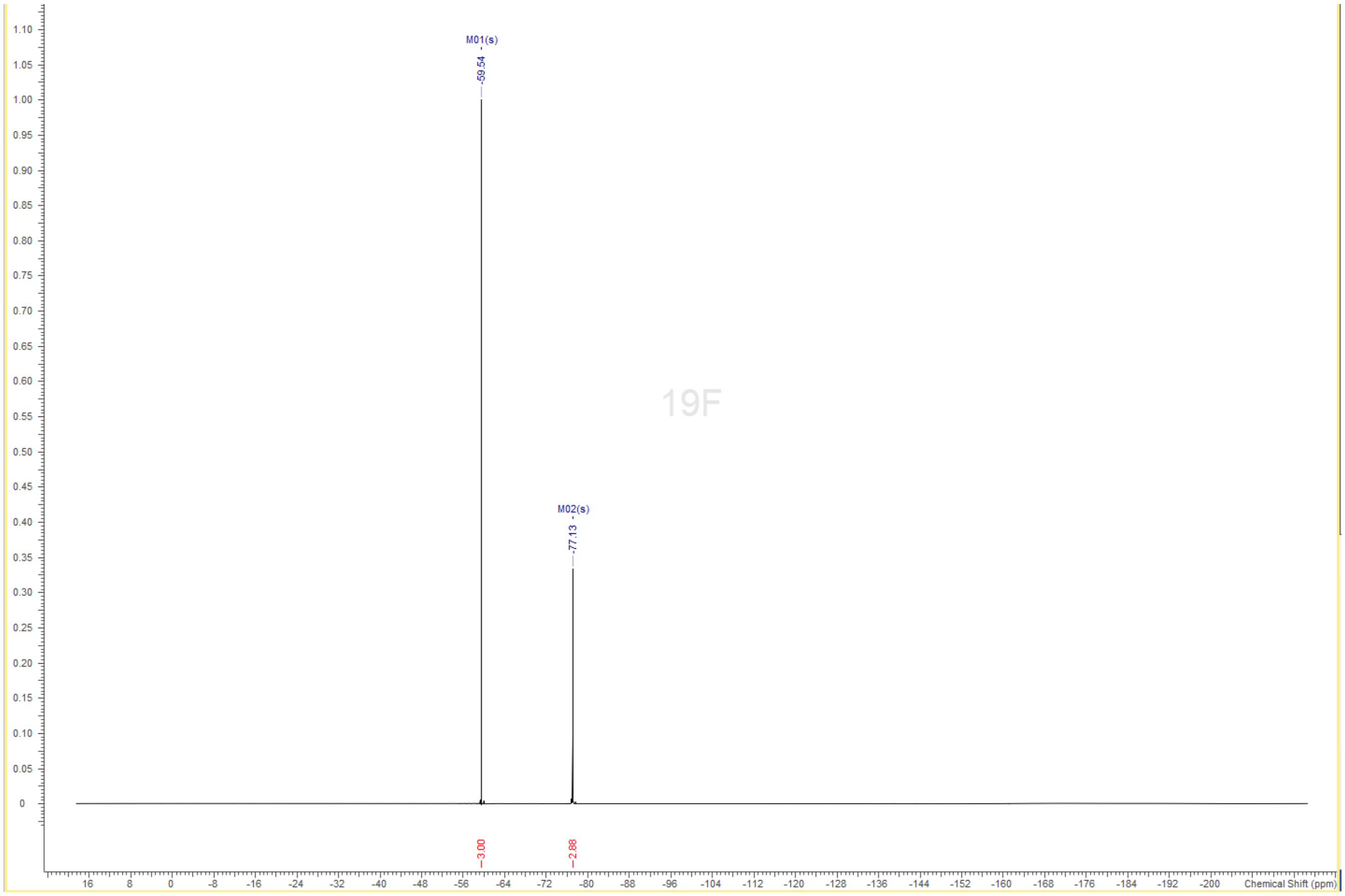

M01(s)
-59.54
M02(s)
-77.13
19F
3.00
2.88
Chemical Shift (ppm)

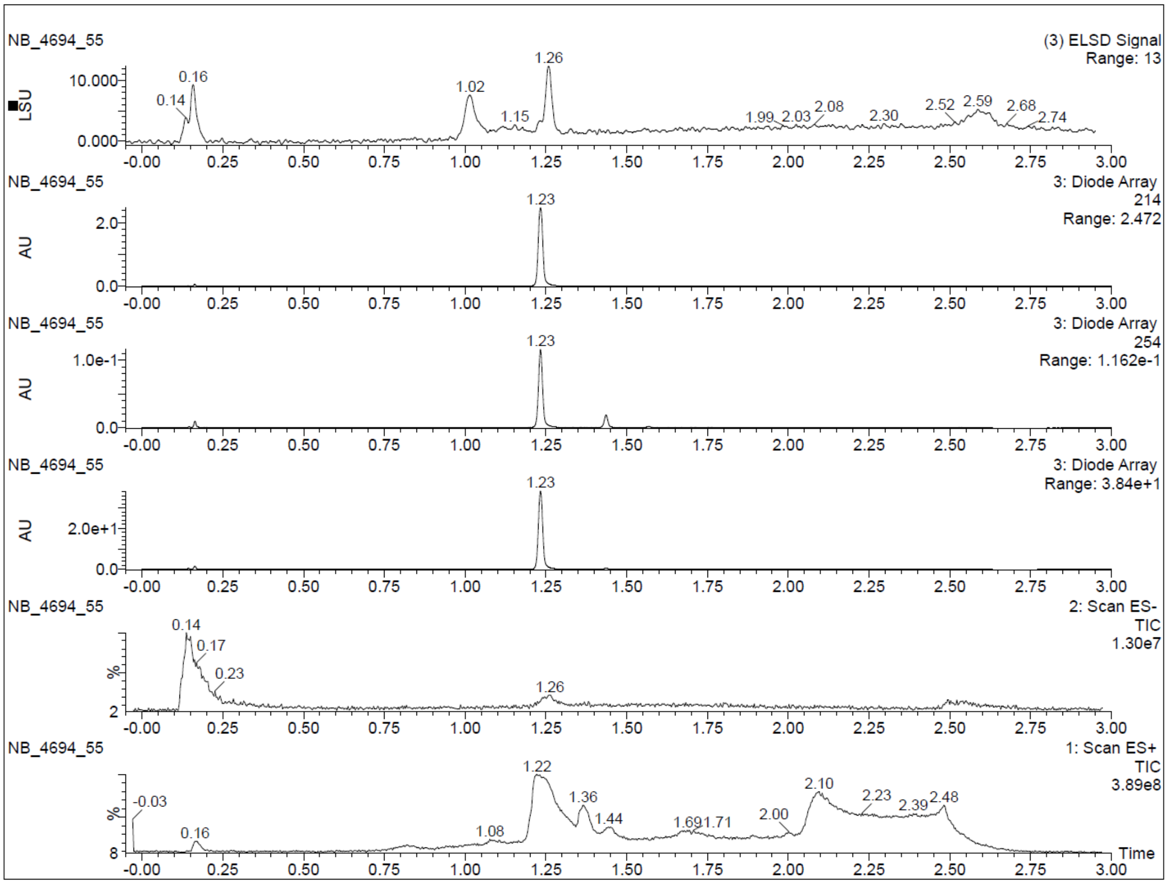

NB_4694_55
(3) ELSD Signal
Range: 13
LSU
3: Diode Array
214
Range: 2.472
AU
3: Diode Array
254
Range: 1.162e-1
3: Diode Array
Range: 3.84e+1
2: Scan ES-
TIC
1.30e7
1: Scan ES+
TIC
3.89e8
%
Time

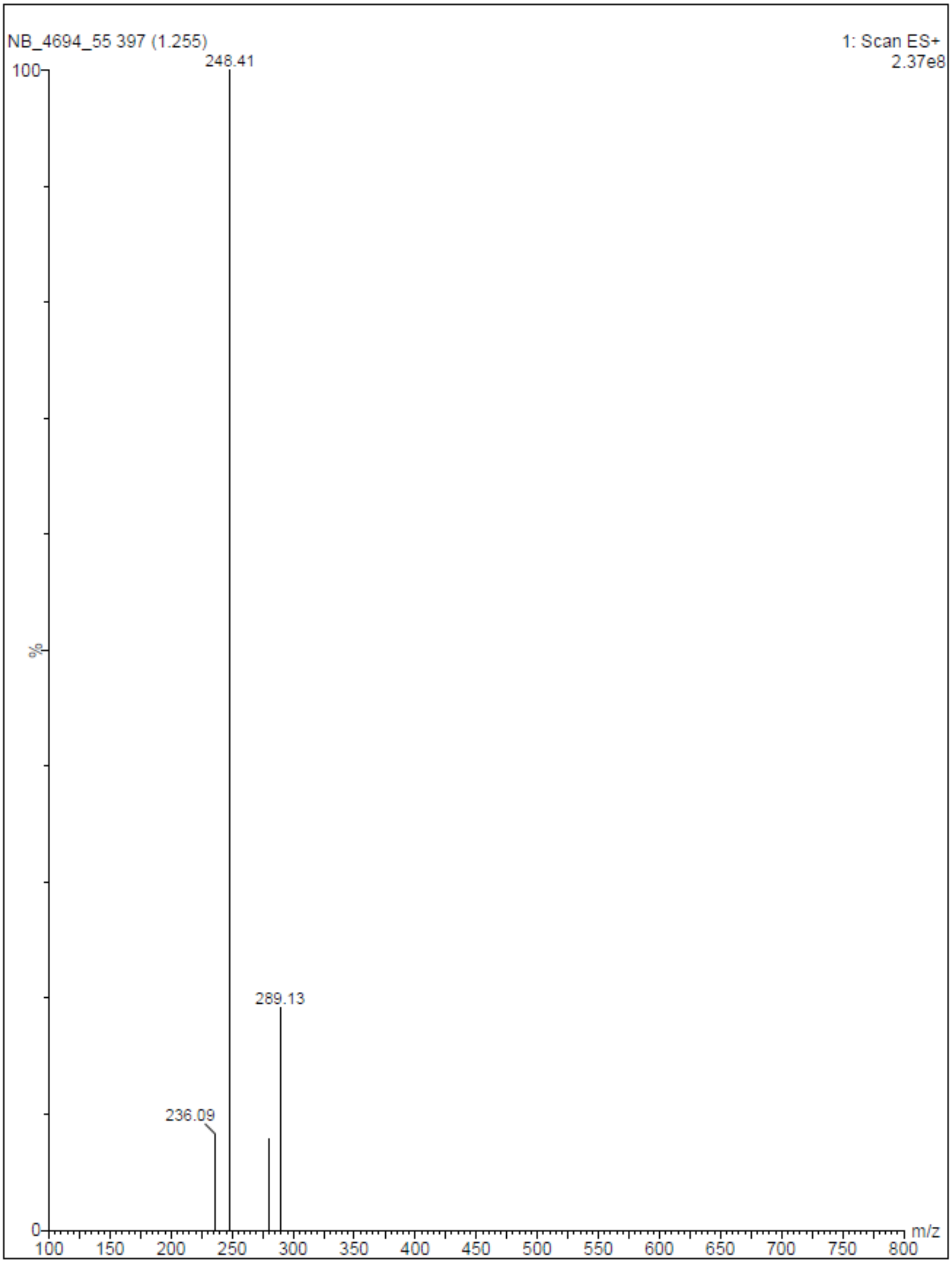
NB_4694_55 397 (1.255)
1: Scan ES+
2.37e8
248.41
289.13
236.09
100
%
0
100 150 200 250 300 350 400 450 500 550 600 650 700 750 800
m/z

3-(4-(Trimethylsilyl)phenyl)morpholine, TFA salt (4ae)

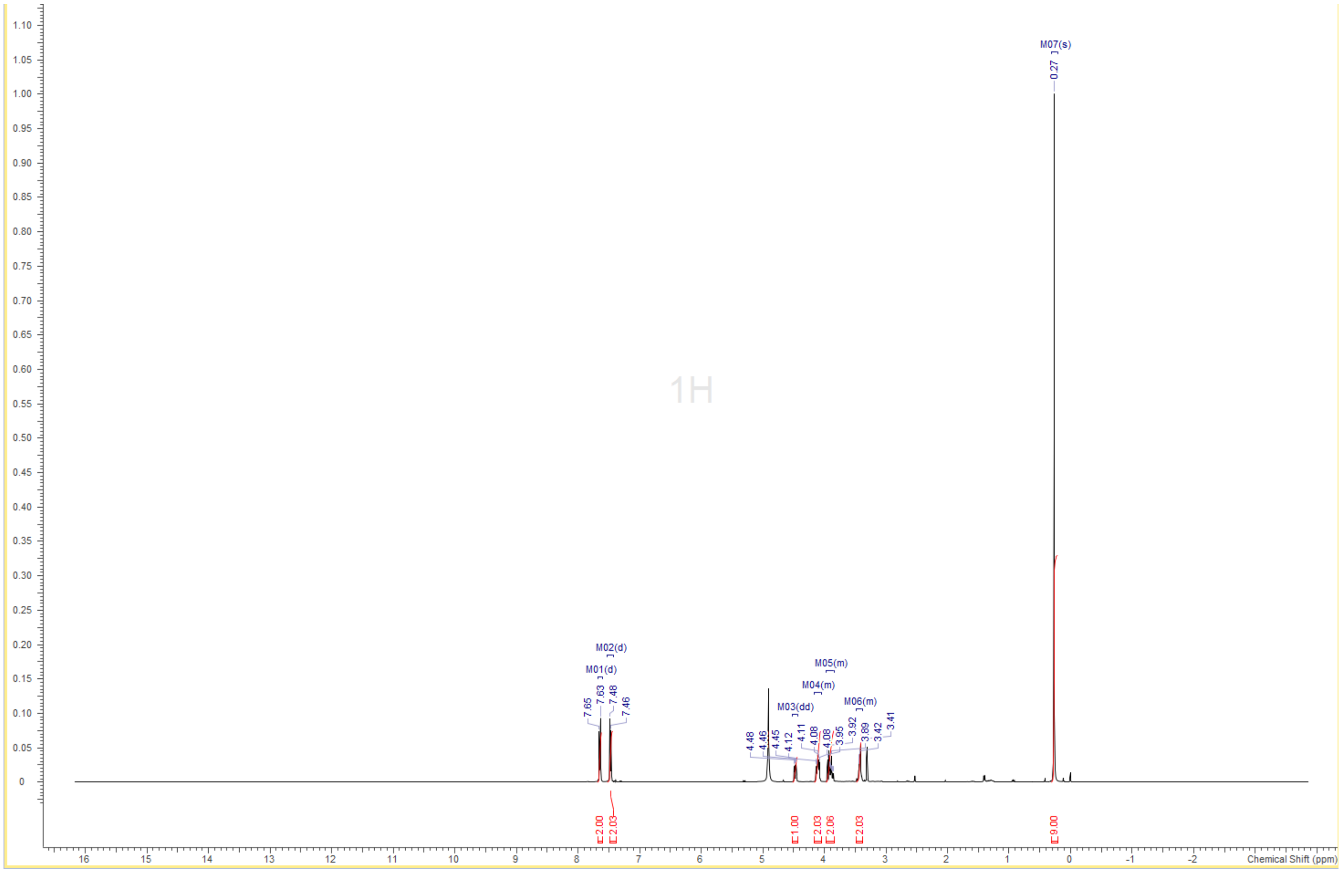

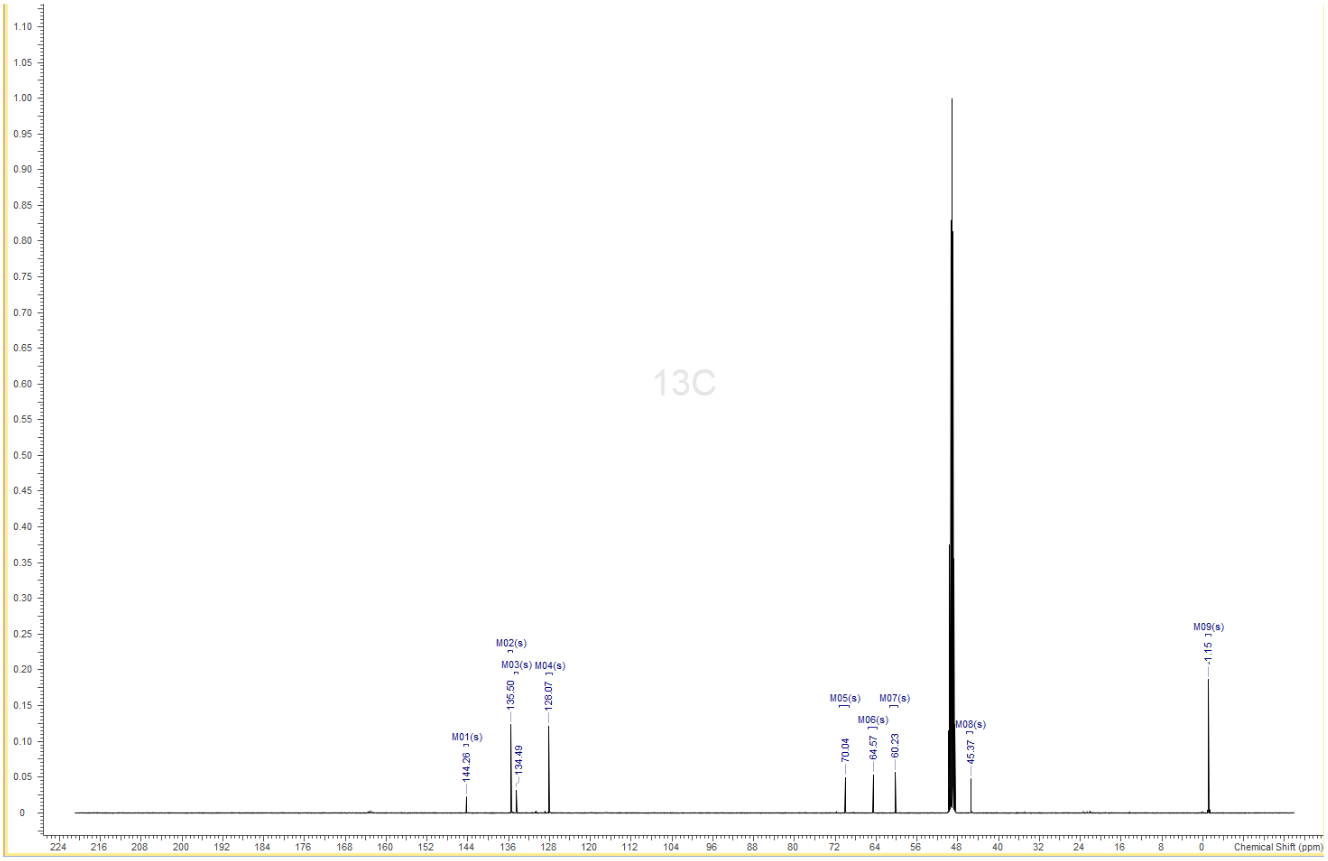

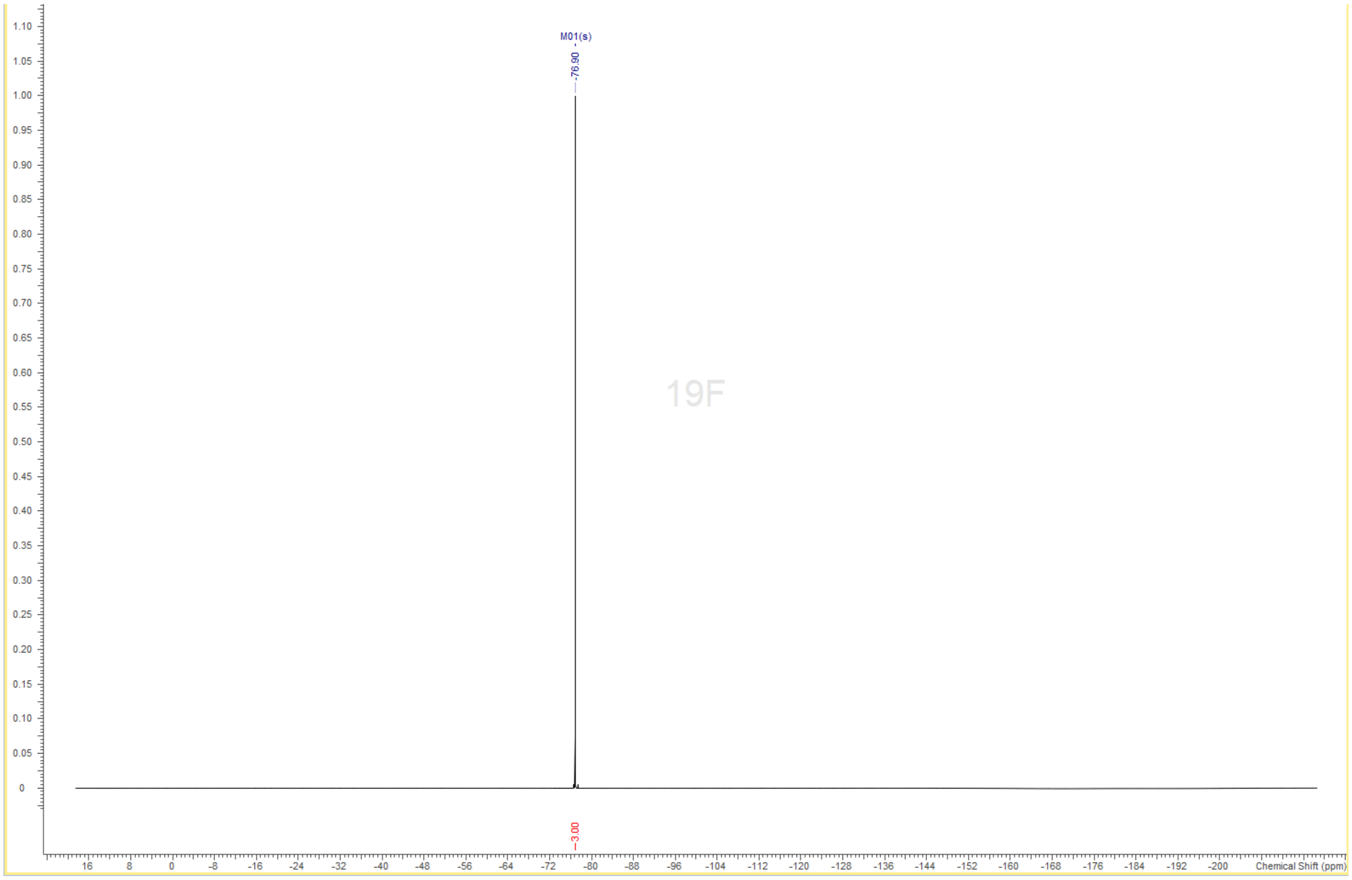
M01(s)
-76.90
19F
-3.00
Chemical Shift (ppm)

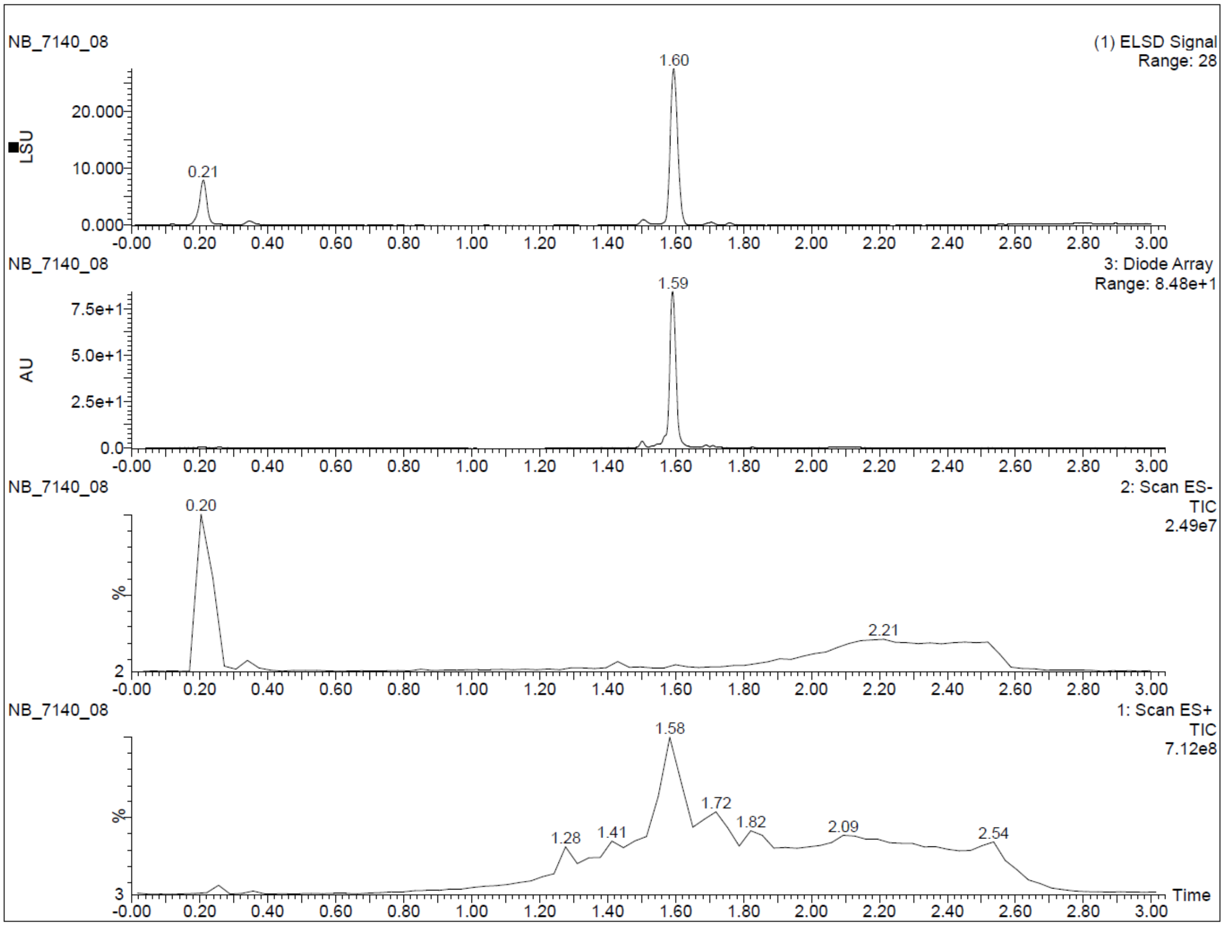
NB_7140_08
(1) ELSD Signal
Range: 28
LSU
1.60
0.21
NB_7140_08
3: Diode Array
Range: 8.48e+1
AU
1.59
NB_7140_08
2: Scan ES-
TIC
2.49e7
0.20
2.21
NB_7140_08
1: Scan ES+
TIC
7.12e8
1.58
1.72
1.82
1.28
1.41
2.09
2.54
Time

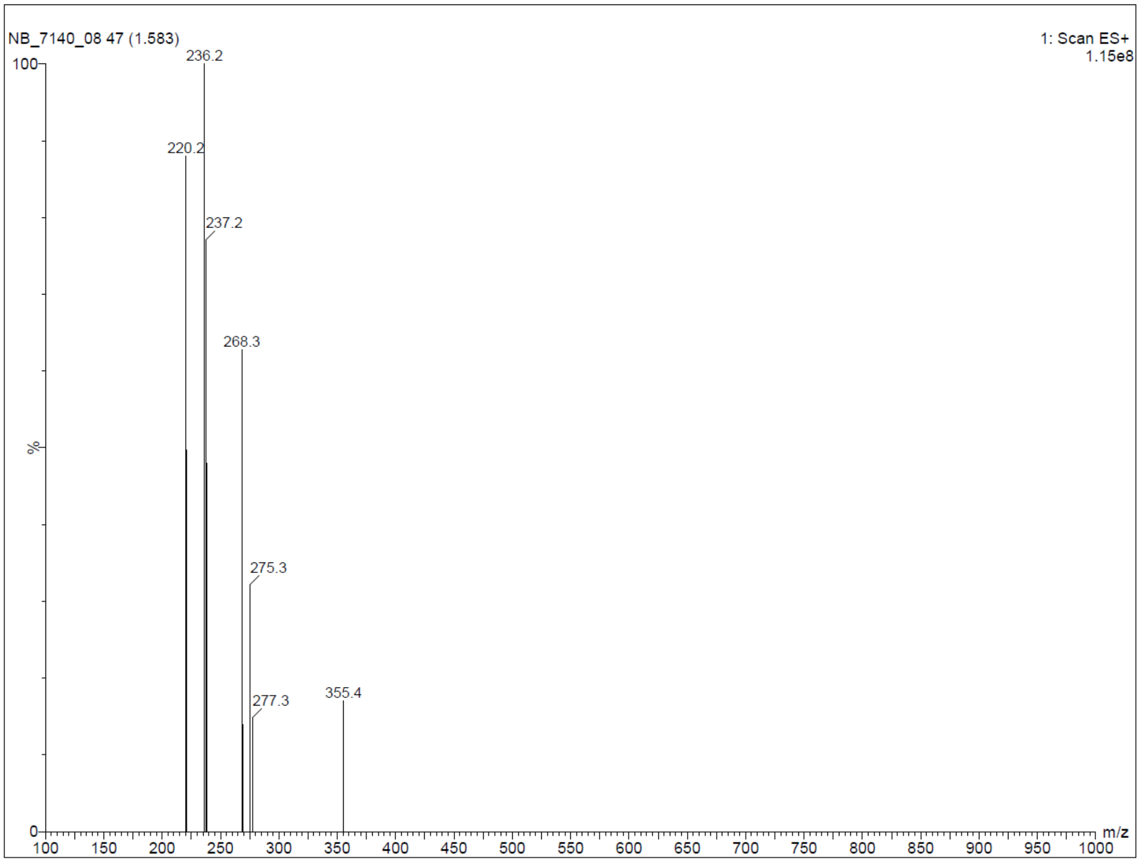

3-(4-(Trifluoromethyl)phenyl)morpholine, TFA salt (4af)

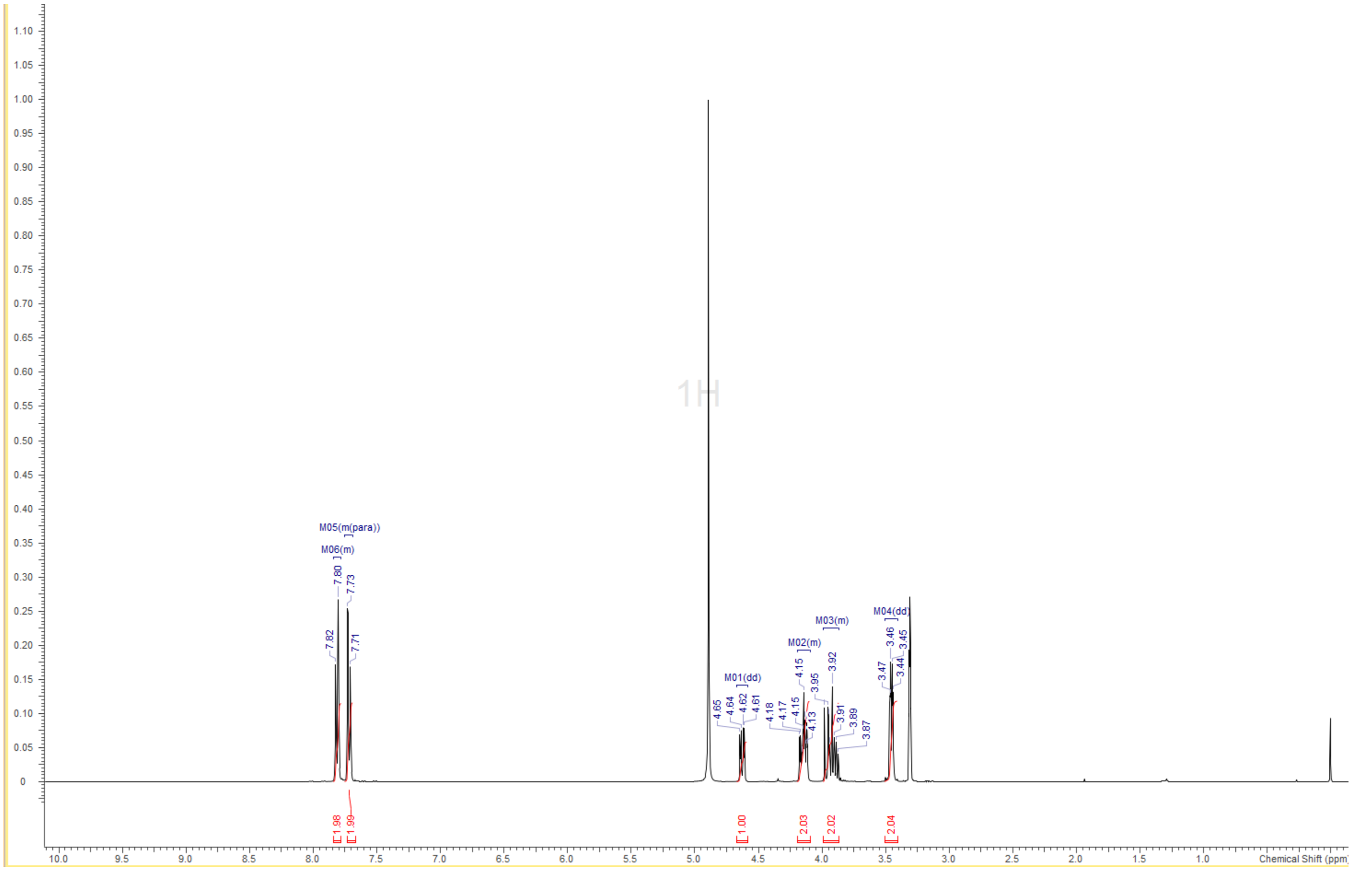

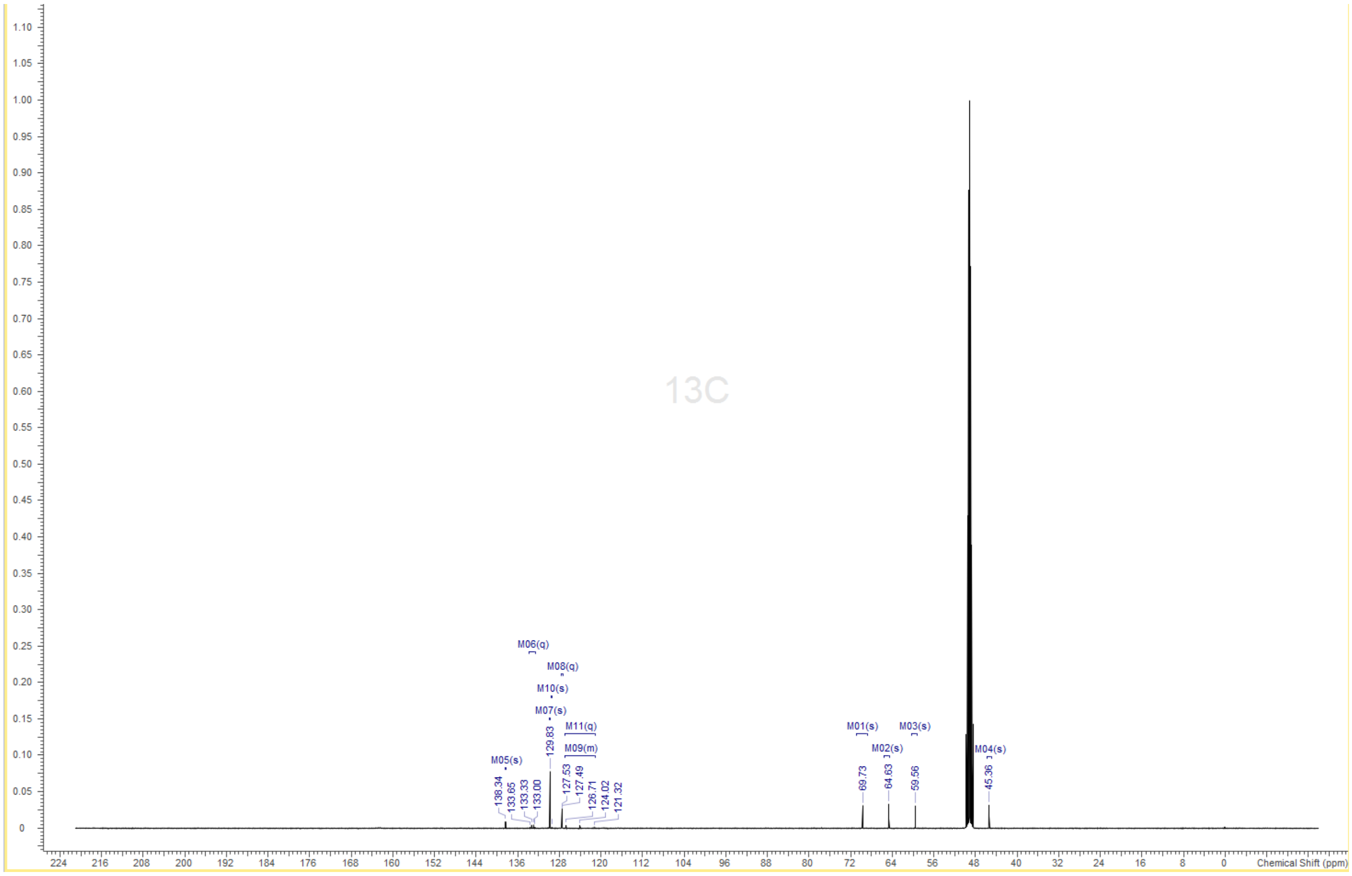

Aromatic Region (Zoomed):

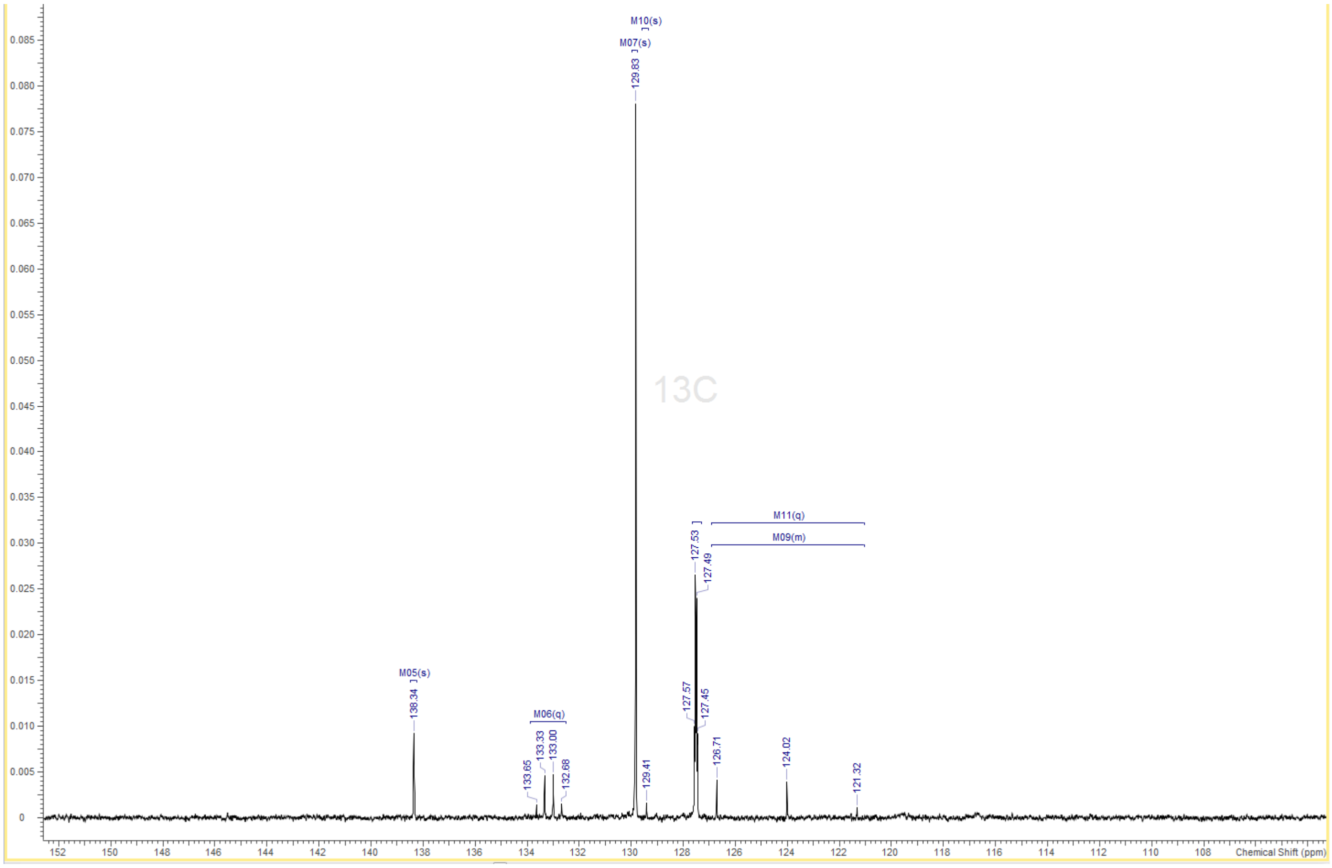

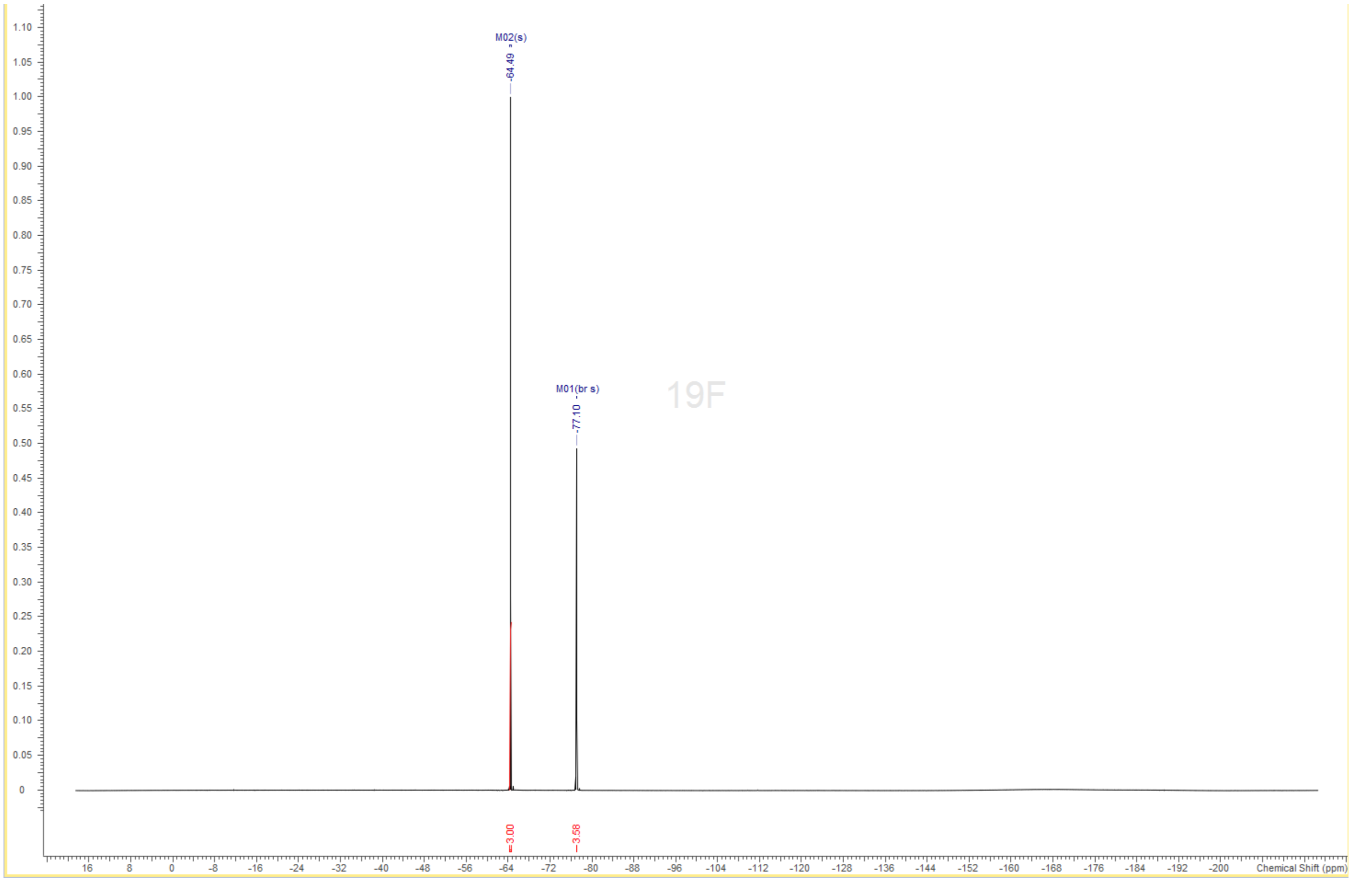
M02(s)
-64.49
M01(br s)
-77.10
19F
3.00
3.58
Chemical Shift (ppm)

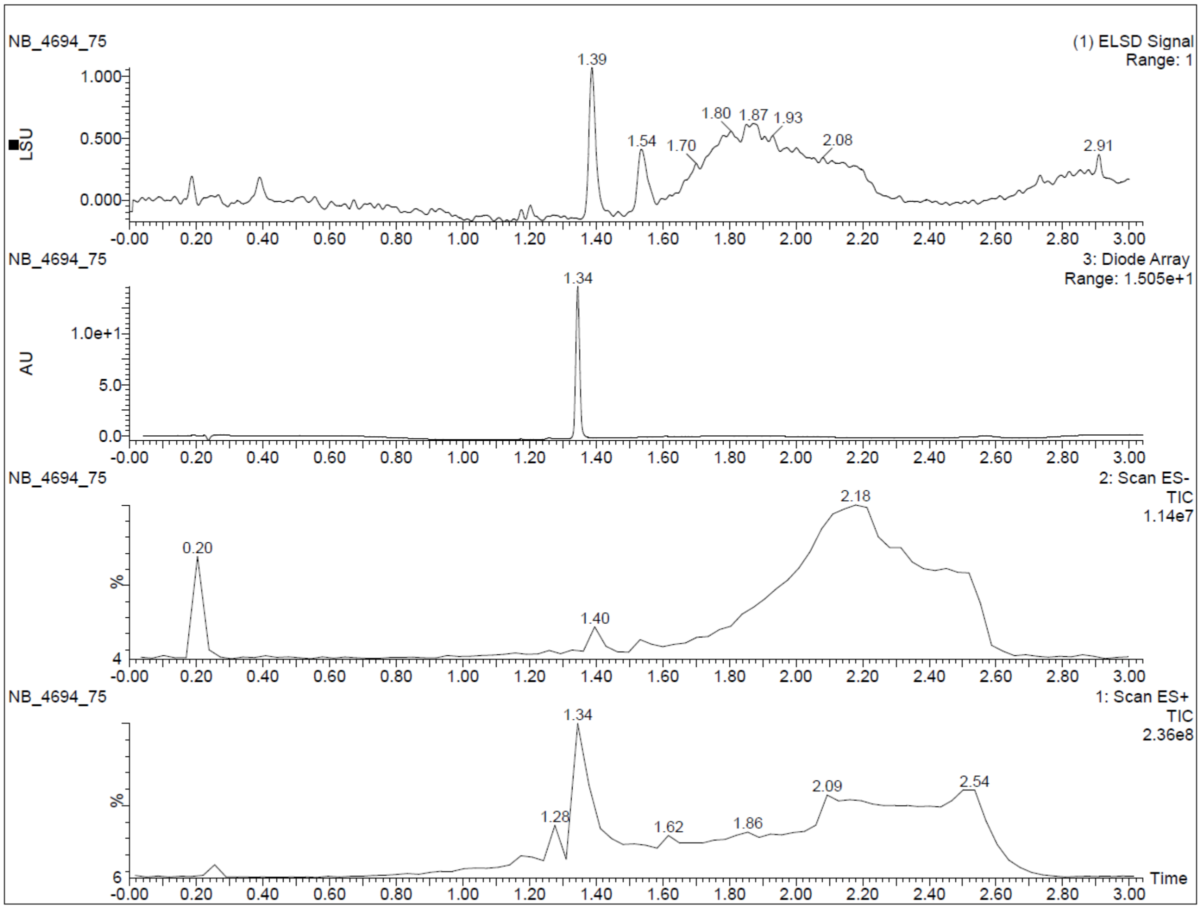
NB_4694_75
(1) ELSD Signal
Range: 1
LSU
1.39
1.54
1.70
1.80
1.87
1.93
2.08
2.91
3: Diode Array
Range: 1.505e+1
AU
1.34
2: Scan ES-
TIC
1.14e7
0.20
1.40
2.18
1: Scan ES+
TIC
2.36e8
1.28
1.34
1.62
1.86
2.09
2.54
Time

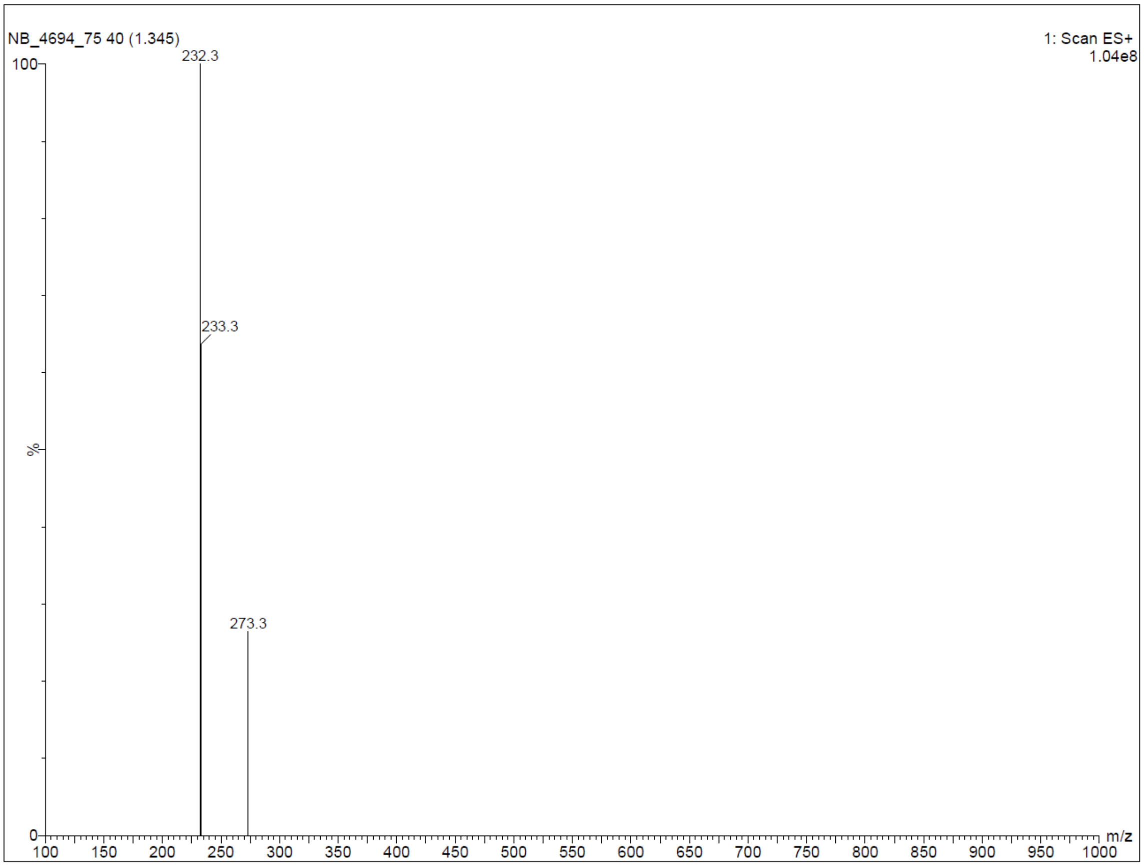

3-(Benzo[b]thiophen-2-yl)morpholine, TFA salt (4au)

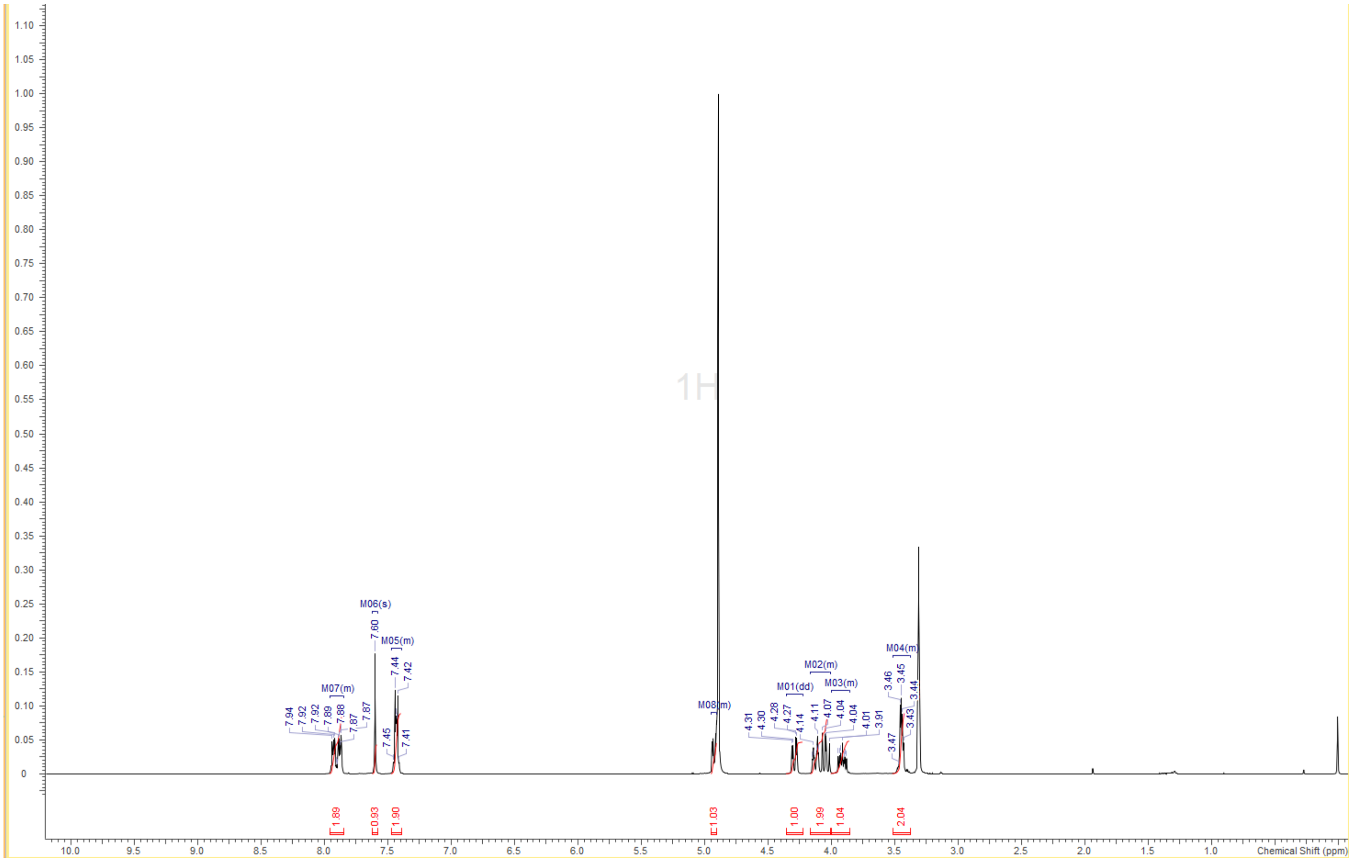

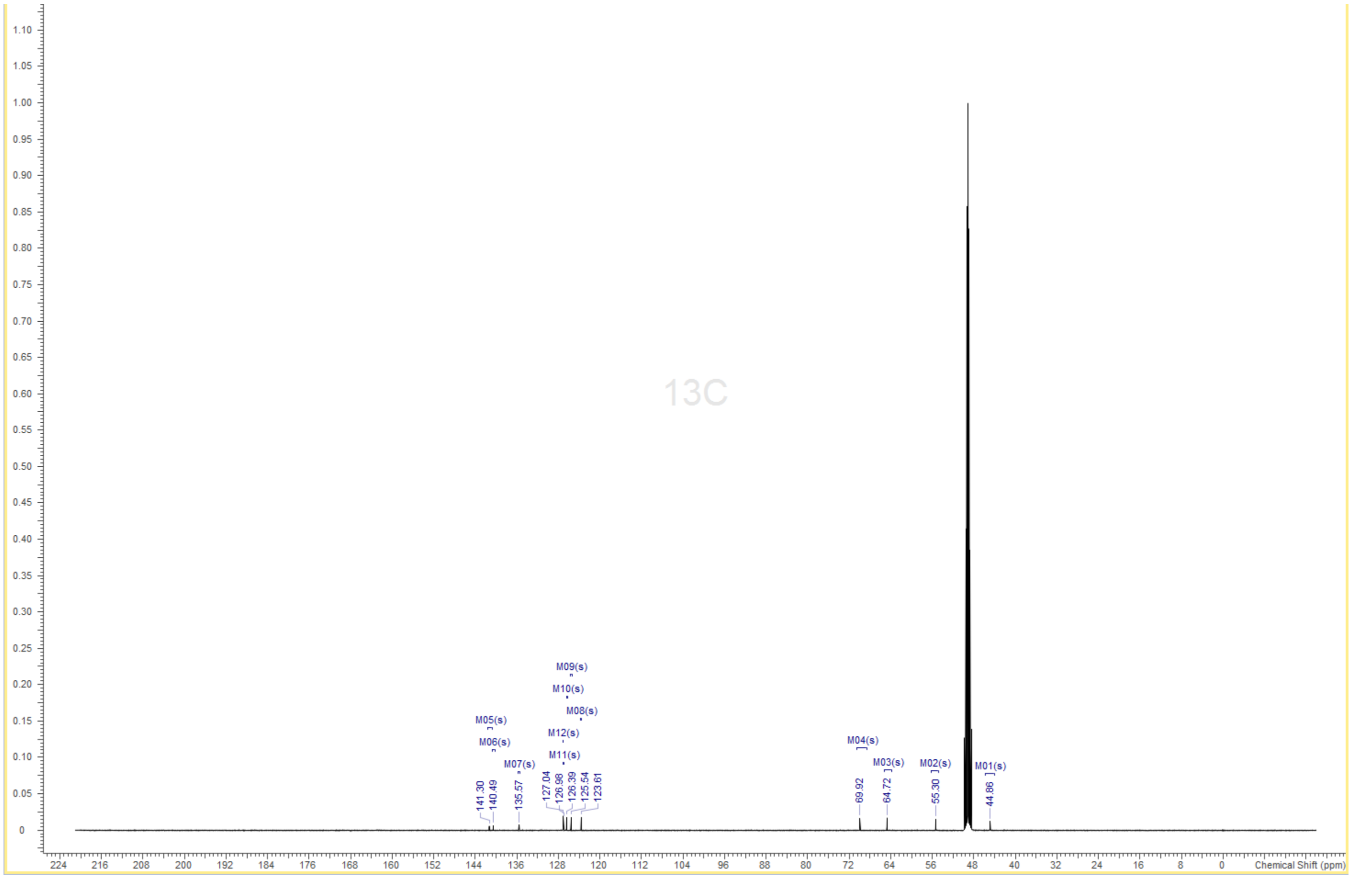
13C
M05(s)
M06(s)
M07(s)
M09(s)
M10(s)
M08(s)
M12(s)
M11(s)
141.30
140.49
135.57
127.04
126.98
126.39
125.54
123.61
M04(s)
M03(s)
M02(s)
M01(s)
69.92
64.72
55.30
44.86
Chemical Shift (ppm)

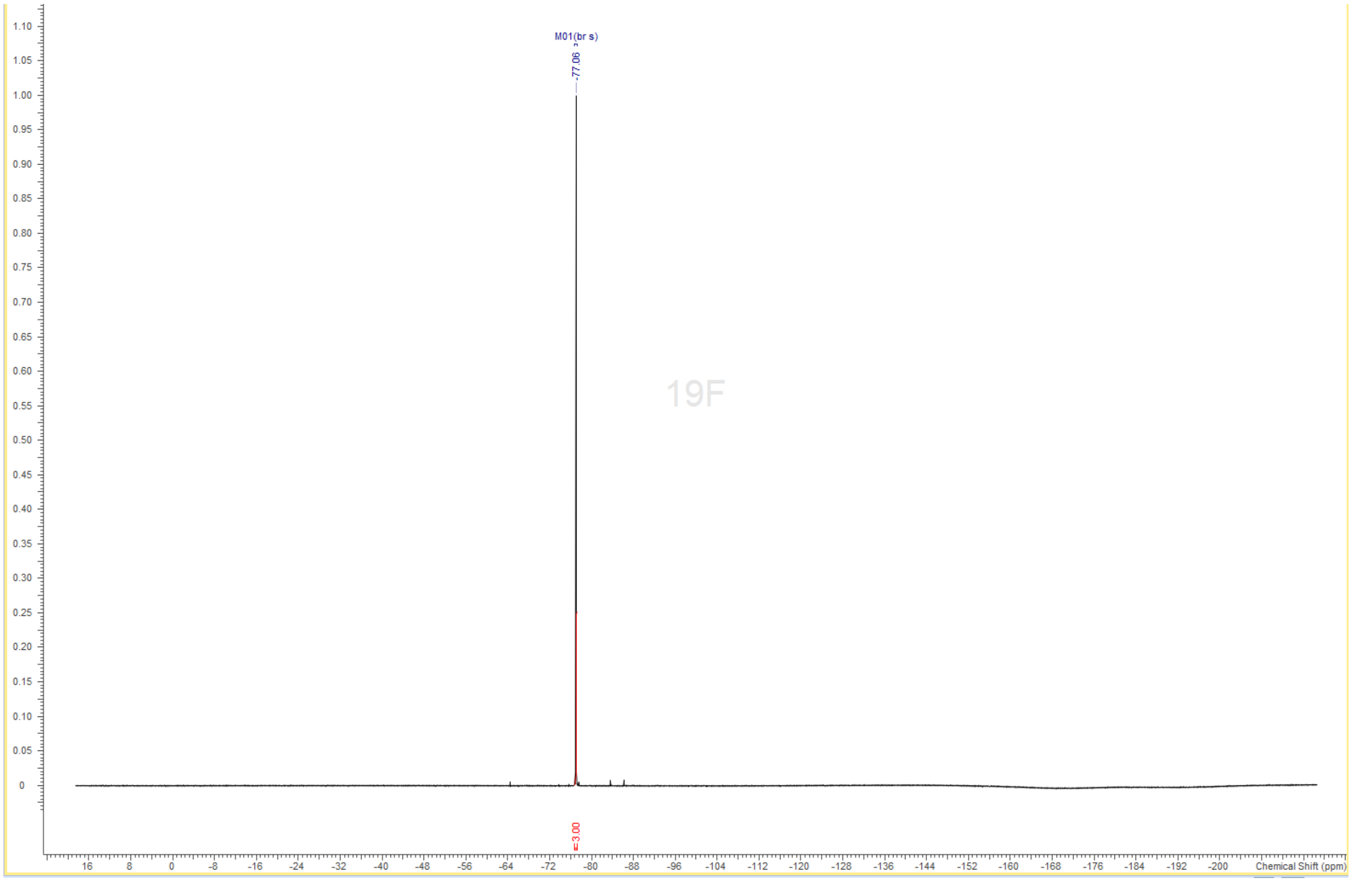
19F
M01(br s)
-77.06
3.00
Chemical Shift (ppm)

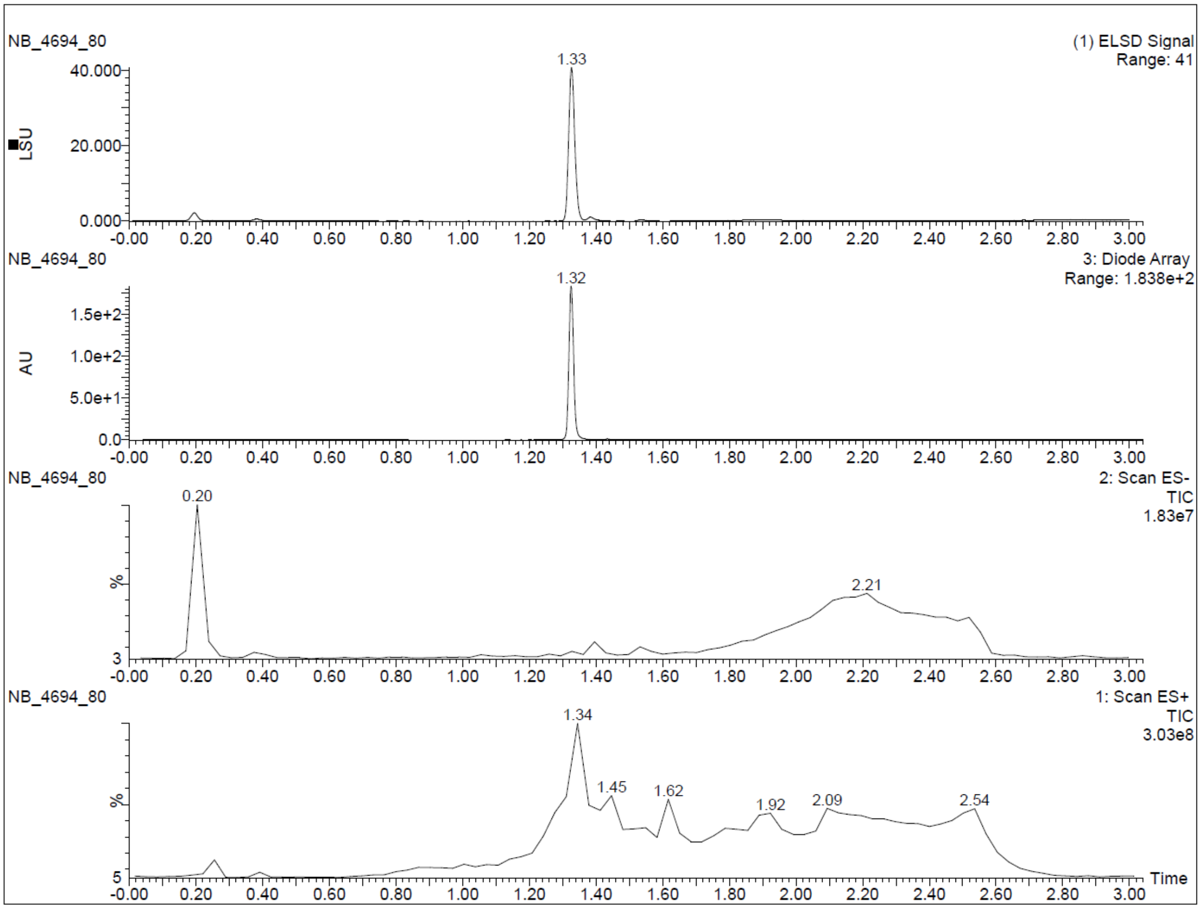
NB_4694_80
(1) ELSD Signal
Range: 41
1.33
LSU
NB_4694_80
3: Diode Array
Range: 1.838e+2
1.32
AU
NB_4694_80
2: Scan ES-
TIC
1.83e7
0.20
2.21
%
NB_4694_80
1: Scan ES+
TIC
3.03e8
1.34
1.45
1.62
1.92
2.09
2.54
%
Time

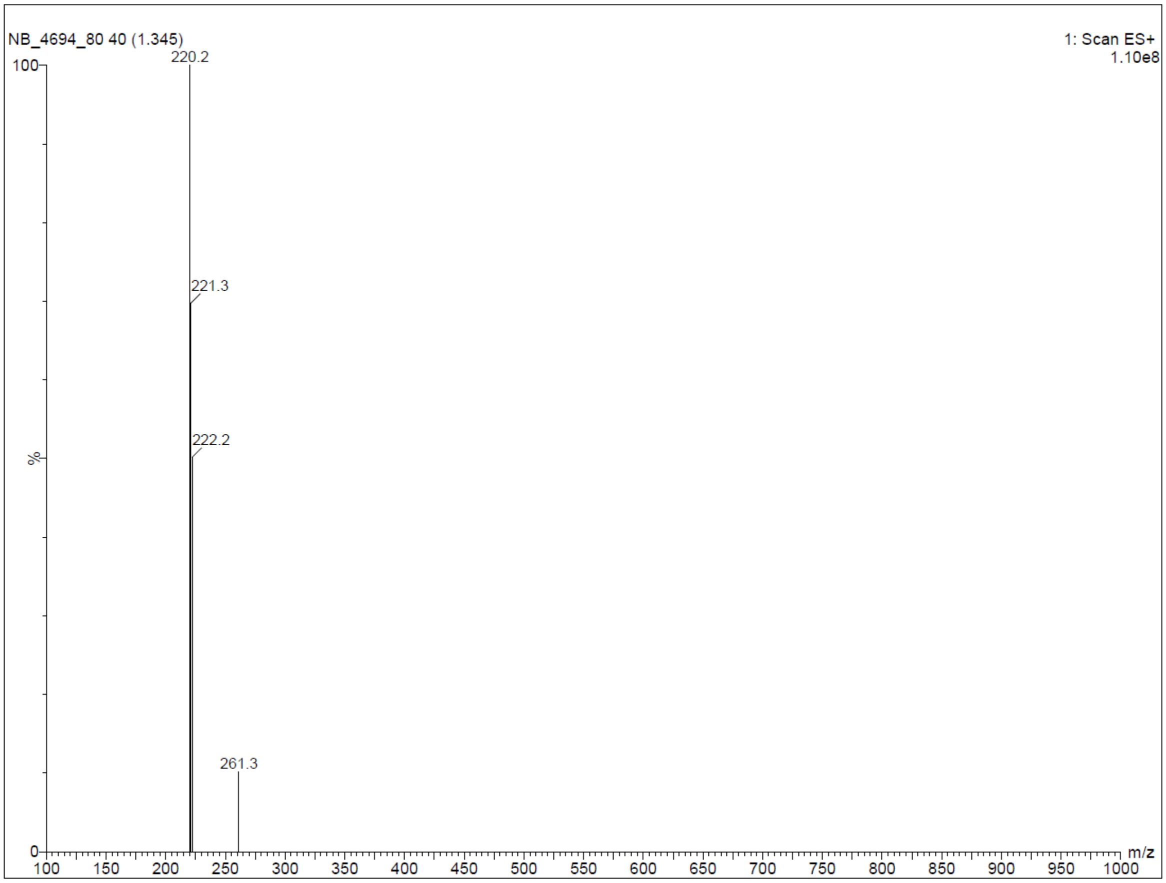
NB_4694_80 40 (1.345)
1: Scan ES+
1.10e8
220.2
221.3
222.2
261.3
%
m/z

3-(2,3-Dihydrobenzofuran-5-yl)morpholine, TFA salt (4ak)

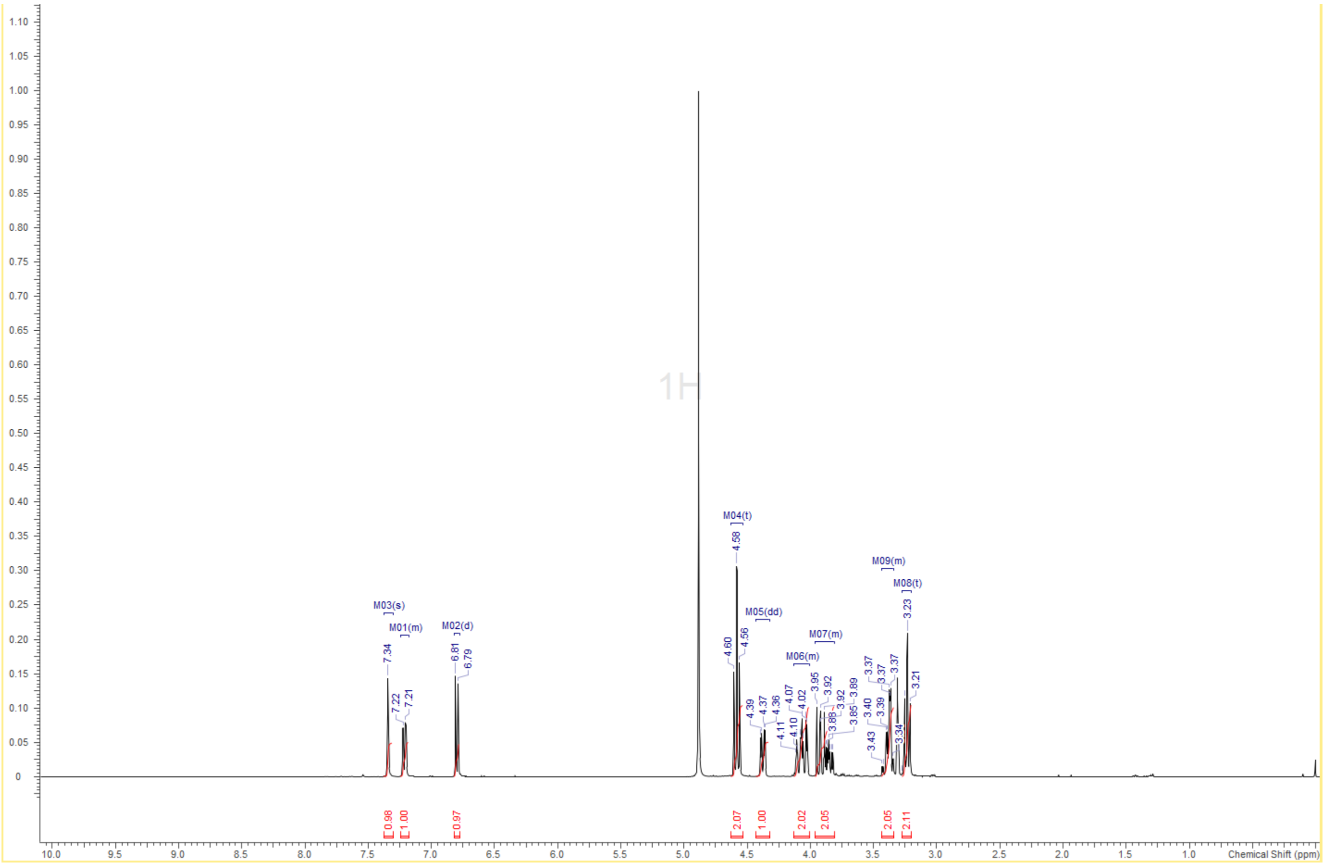

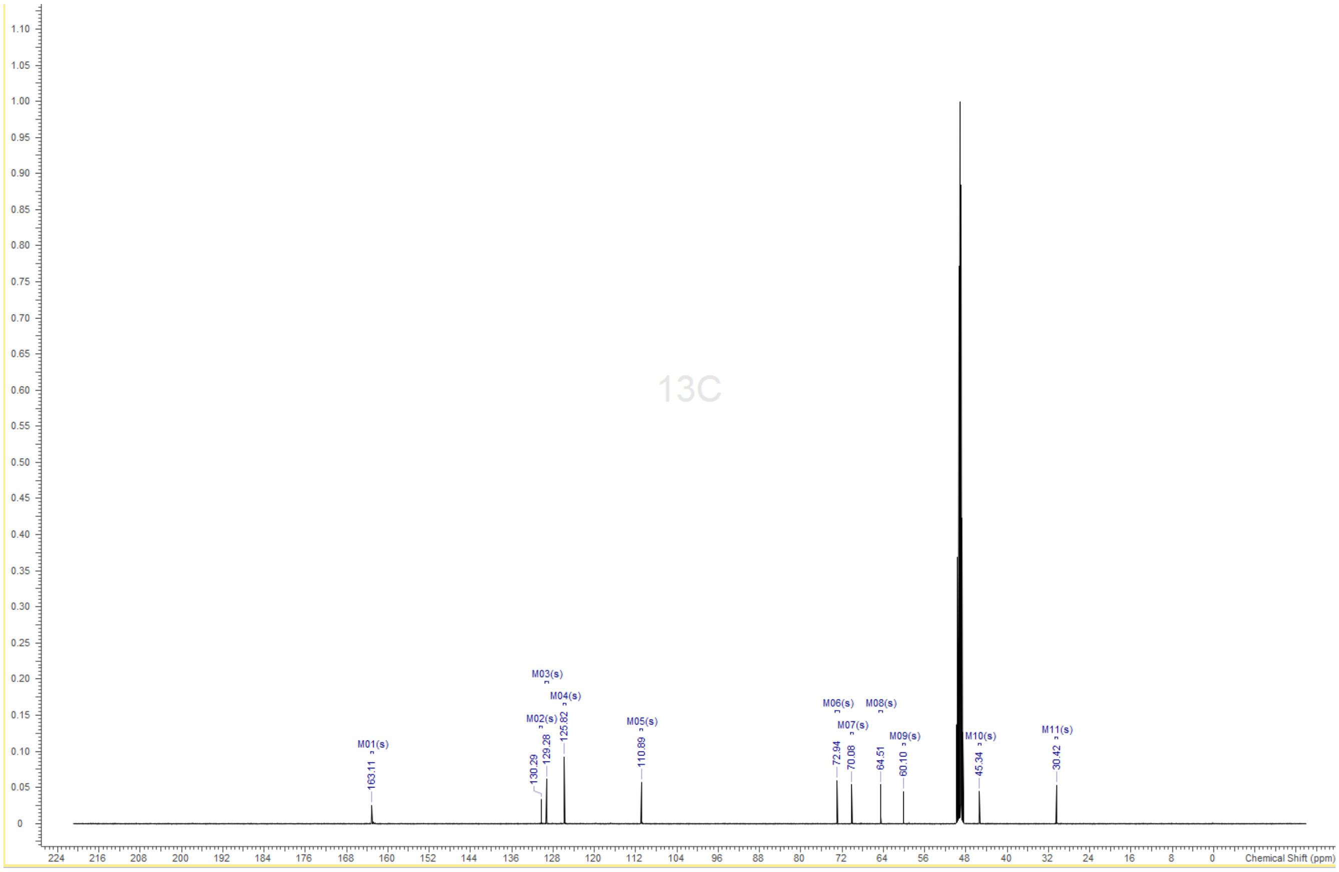

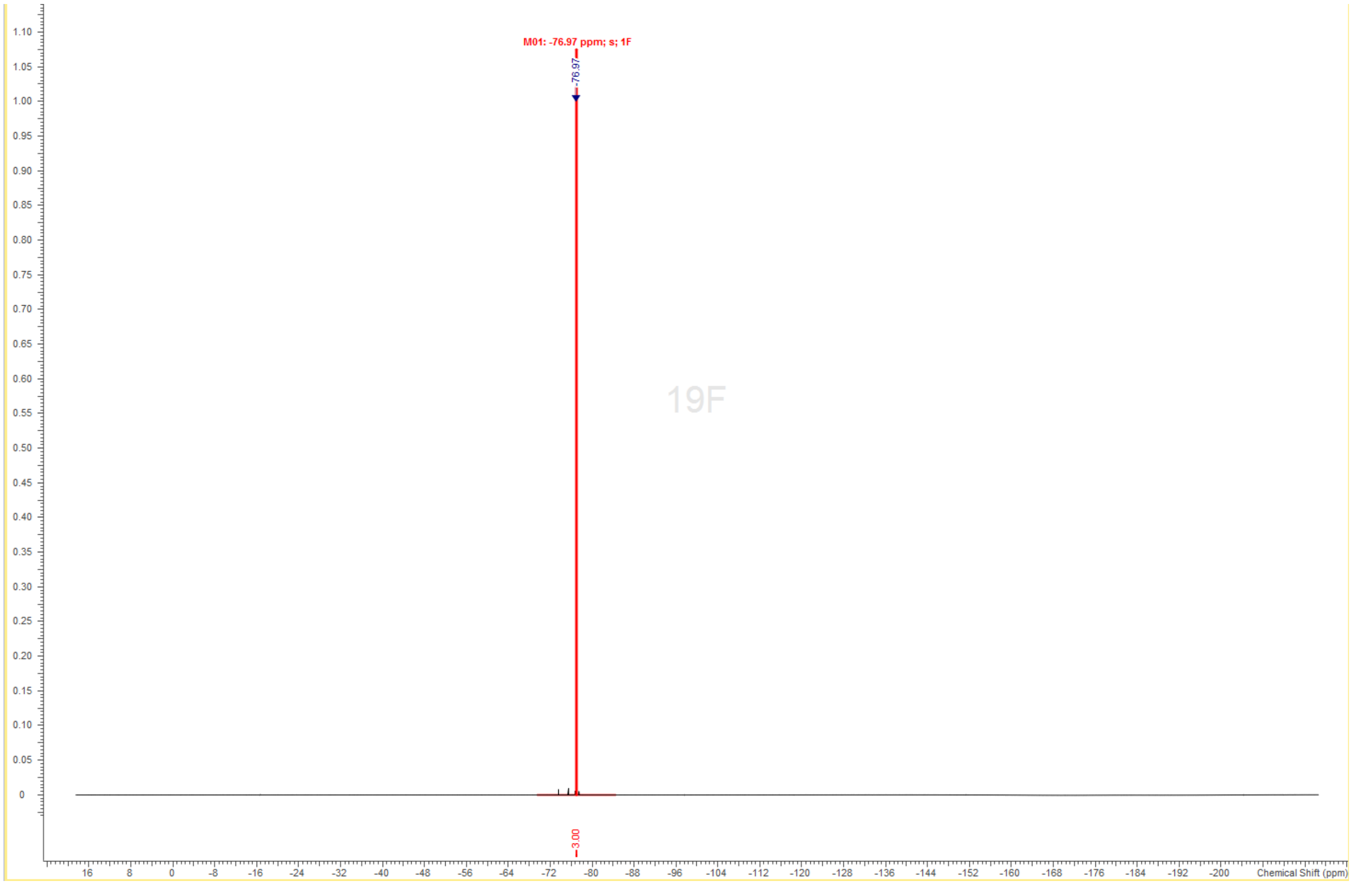
M01: -76.97 ppm; s; 1F
-76.97
19F
3.00
Chemical Shift (ppm)

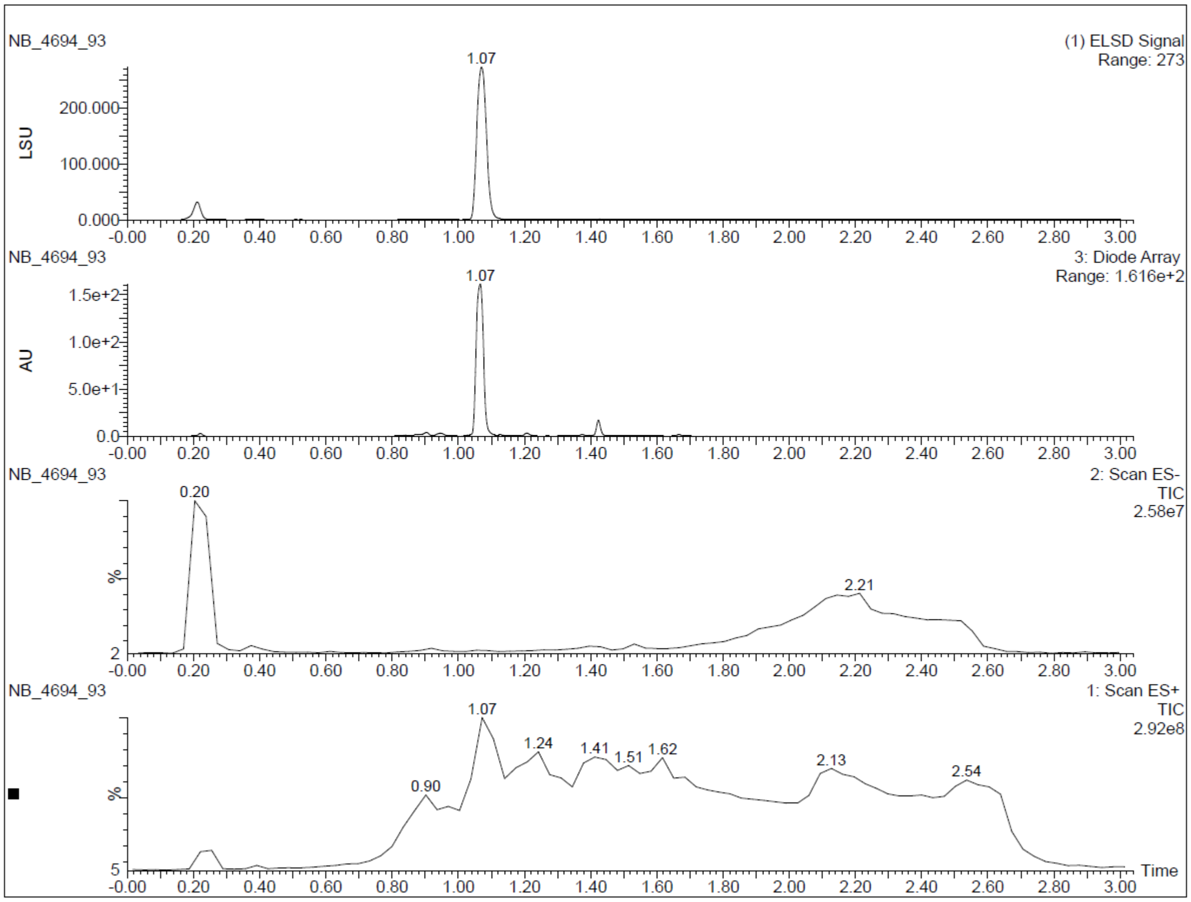
NB_4694_93
(1) ELSD Signal
Range: 273
LSU
1.07
NB_4694_93
3: Diode Array
Range: 1.616e+2
AU
1.07
NB_4694_93
2: Scan ES-
TIC
2.58e7
0.20
2.21
NB_4694_93
1: Scan ES+
TIC
2.92e8
1.07
1.24
1.41
1.51
1.62
0.90
2.13
2.54
Time

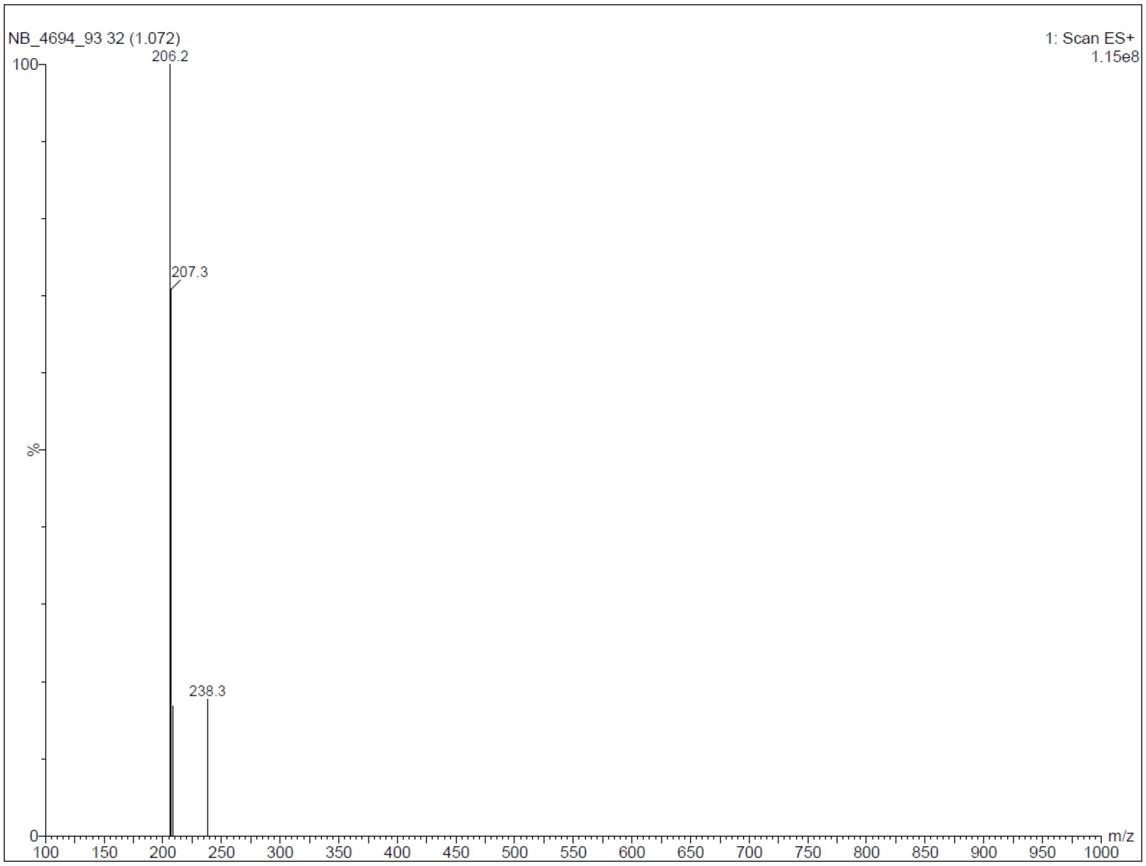

3-(2,5-Dimethylphenyl)morpholine, TFA salt (4ah)

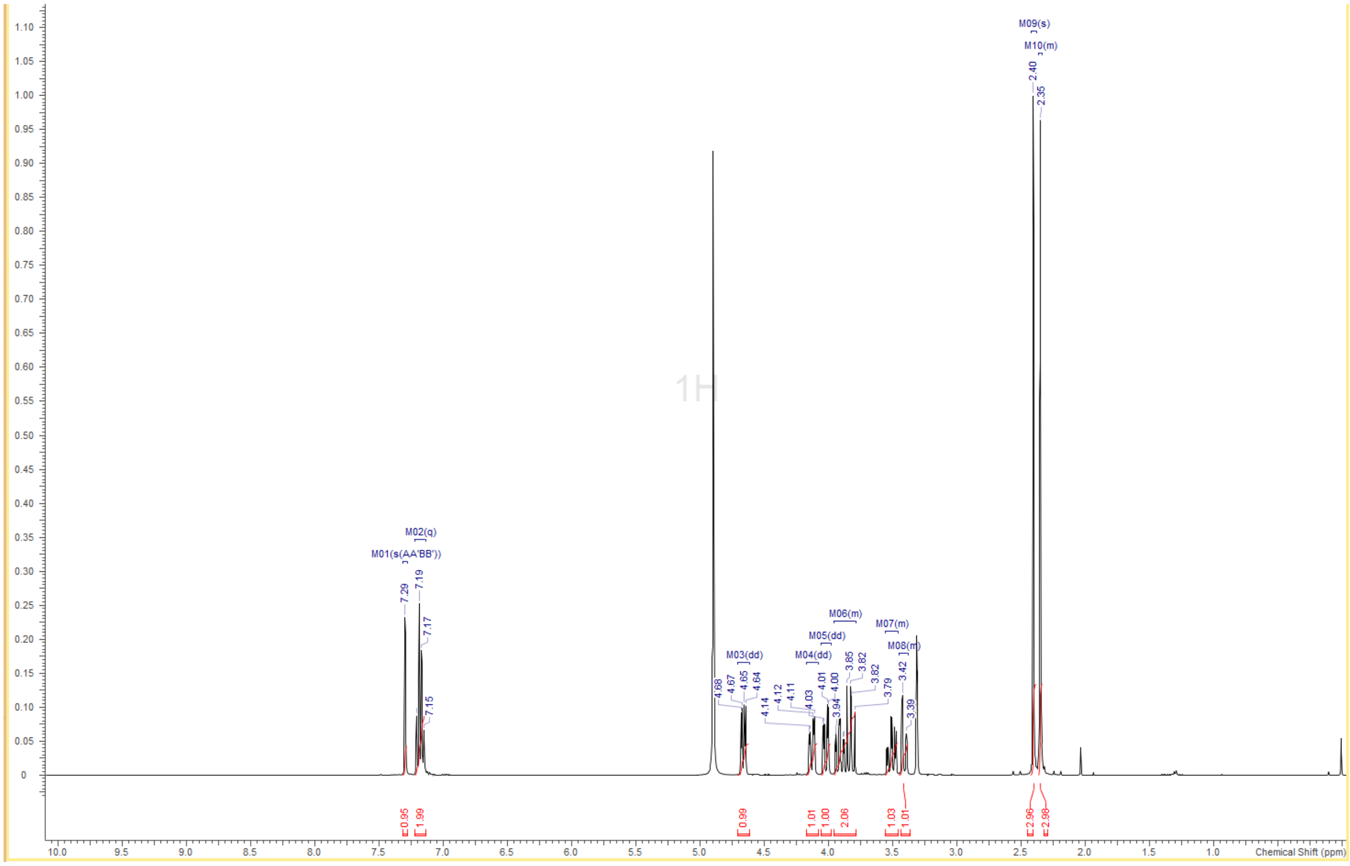

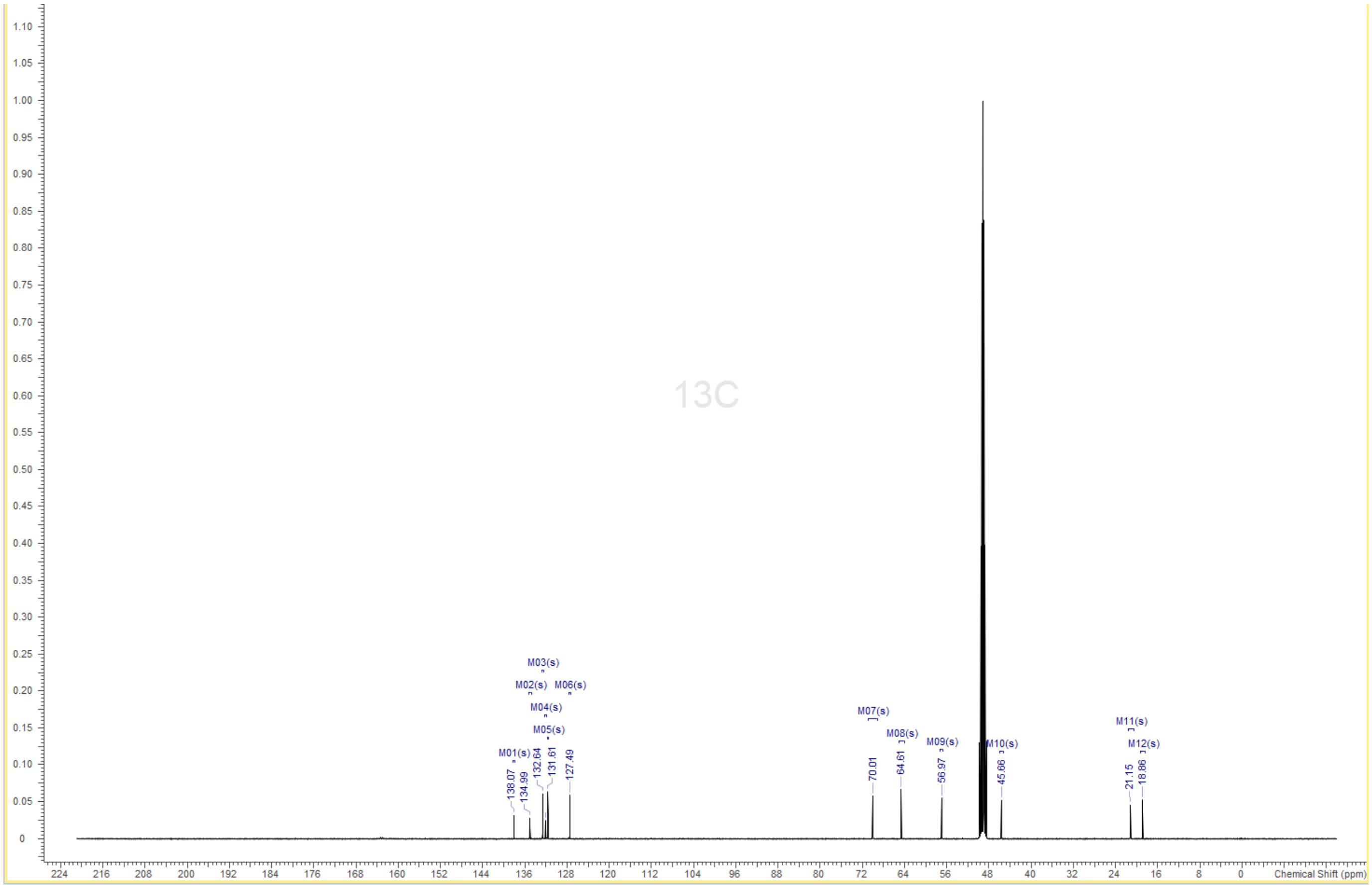
13C
M01(s)
M02(s)
M03(s)
M04(s)
M05(s)
M06(s)
M07(s)
M08(s)
M09(s)
M10(s)
M11(s)
M12(s)
138.07
134.99
132.64
131.61
127.49
70.01
64.61
56.97
45.66
21.15
18.86
Chemical Shift (ppm)

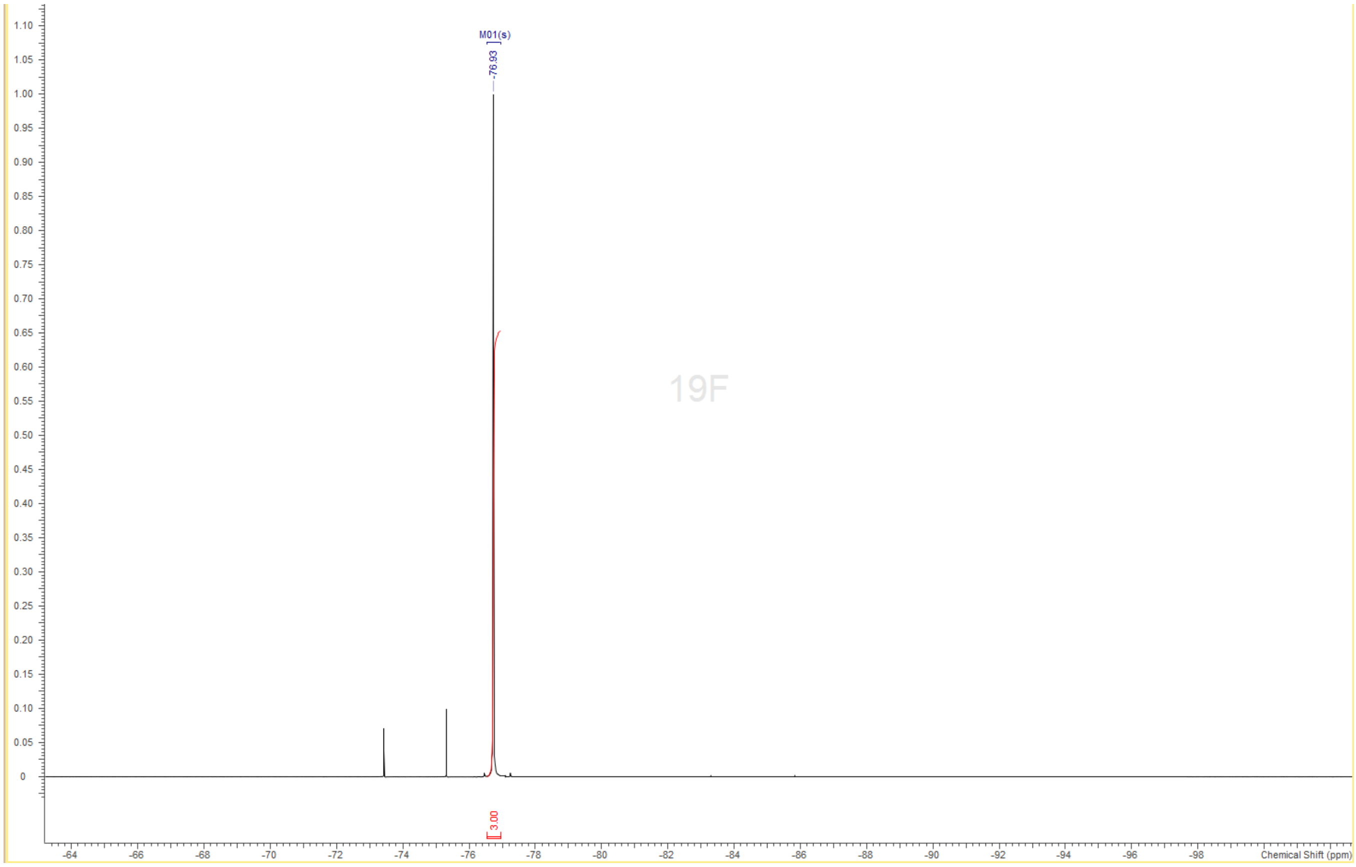
19F
M01(s)
-76.93
3.00
Chemical Shift (ppm)

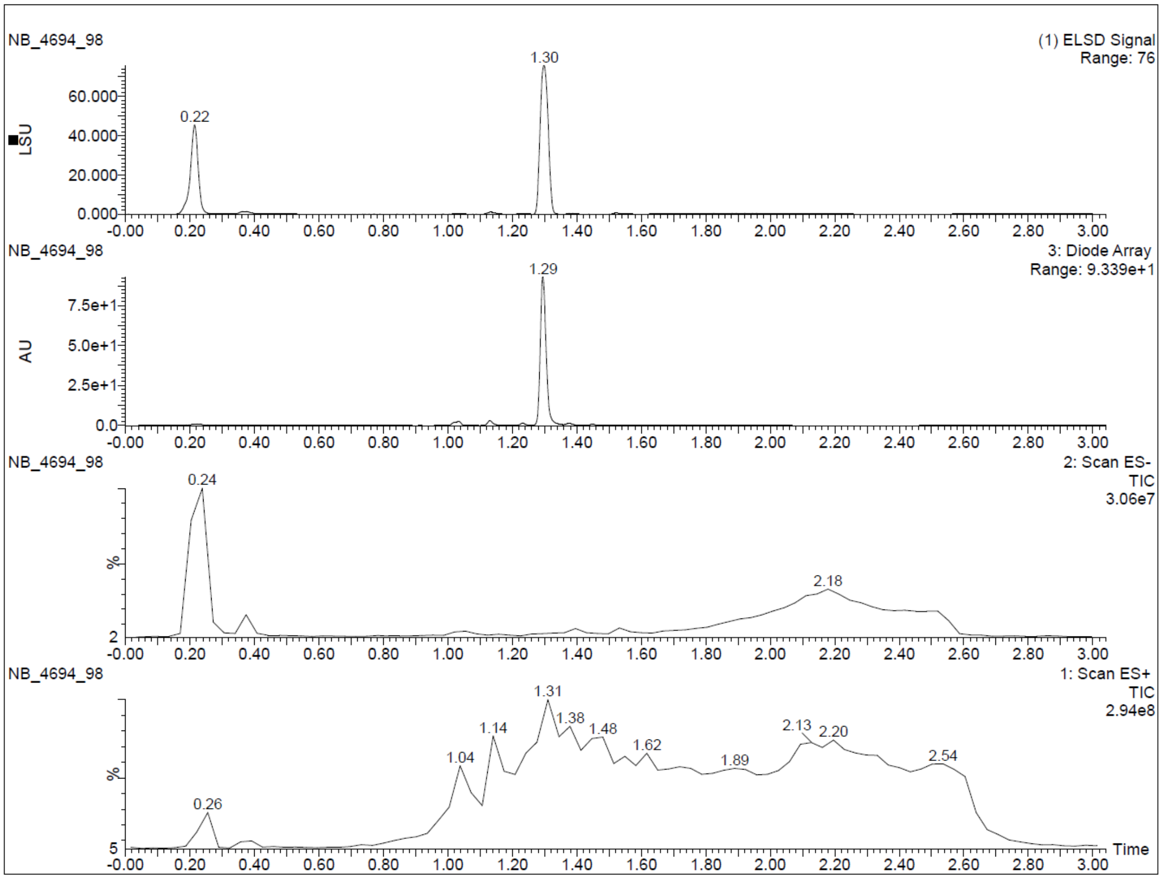
NB_4694_98
(1) ELSD Signal
Range: 76
LSU
0.22
1.30
3: Diode Array
Range: 9.339e+1
AU
1.29
2: Scan ES-
TIC
3.06e7
0.24
2.18
1: Scan ES+
TIC
2.94e8
0.26
1.04
1.14
1.31
1.38
1.48
1.62
1.89
2.13
2.20
2.54
Time

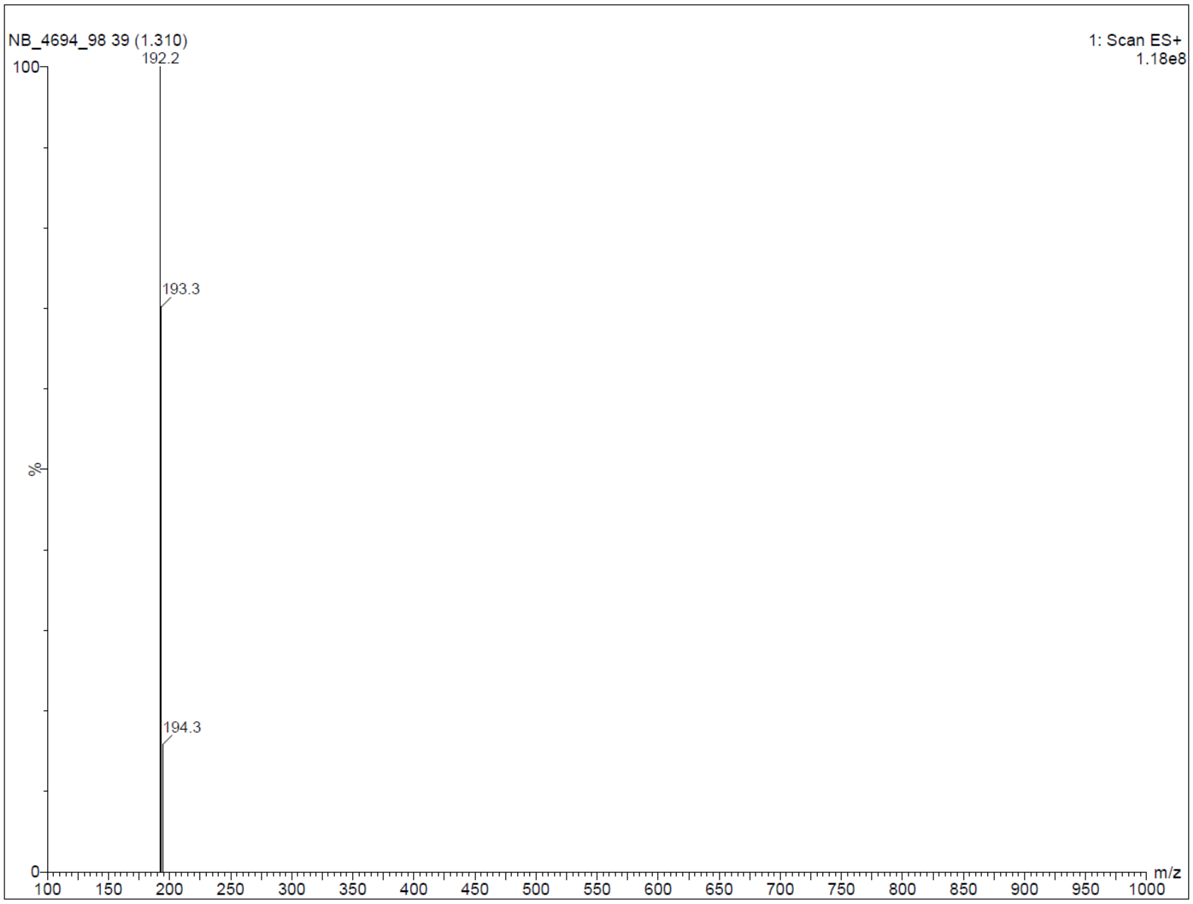
NB_4694_98 39 (1.310)
1: Scan ES+
1.18e8
192.2
193.3
194.3
%
m/z

3-(5-Bromopyridin-3-yl)morpholine, TFA salt (4ar)

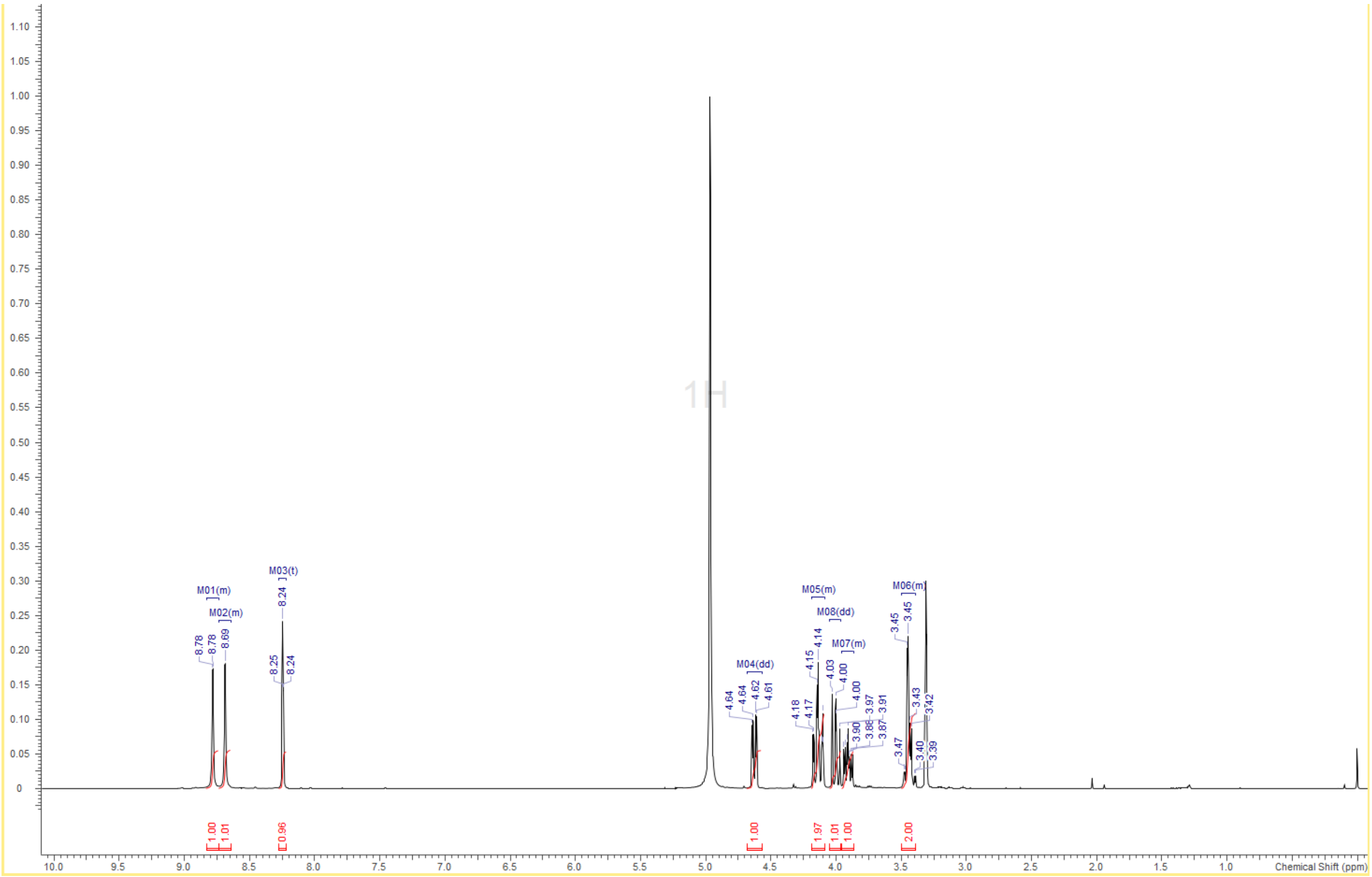

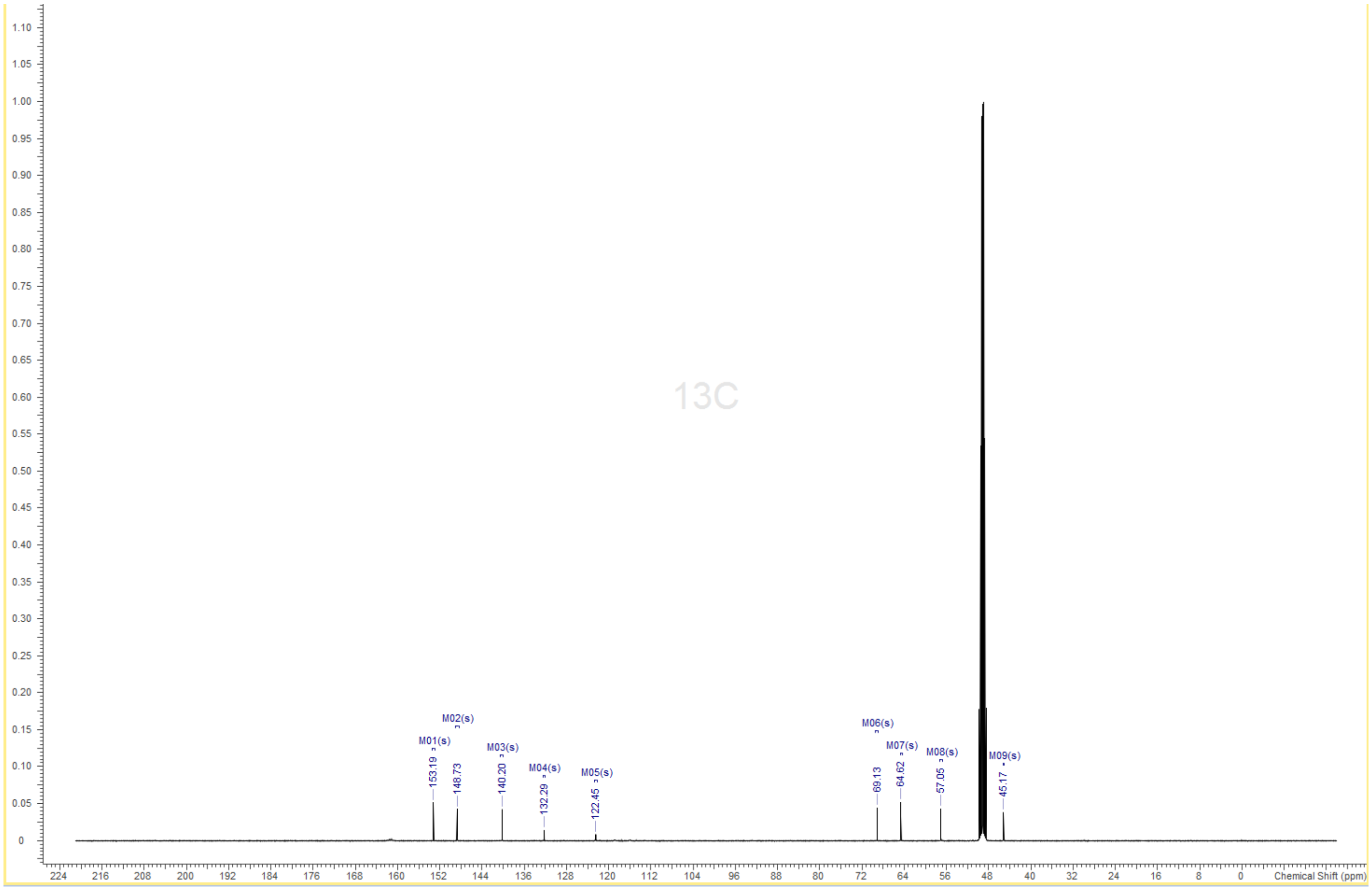
13C
M01(s) 153.19
M02(s) 148.73
M03(s) 140.20
M04(s) 132.29
M05(s) 122.45
M06(s) 69.13
M07(s) 64.62
M08(s) 57.05
M09(s) 45.17
Chemical Shift (ppm)

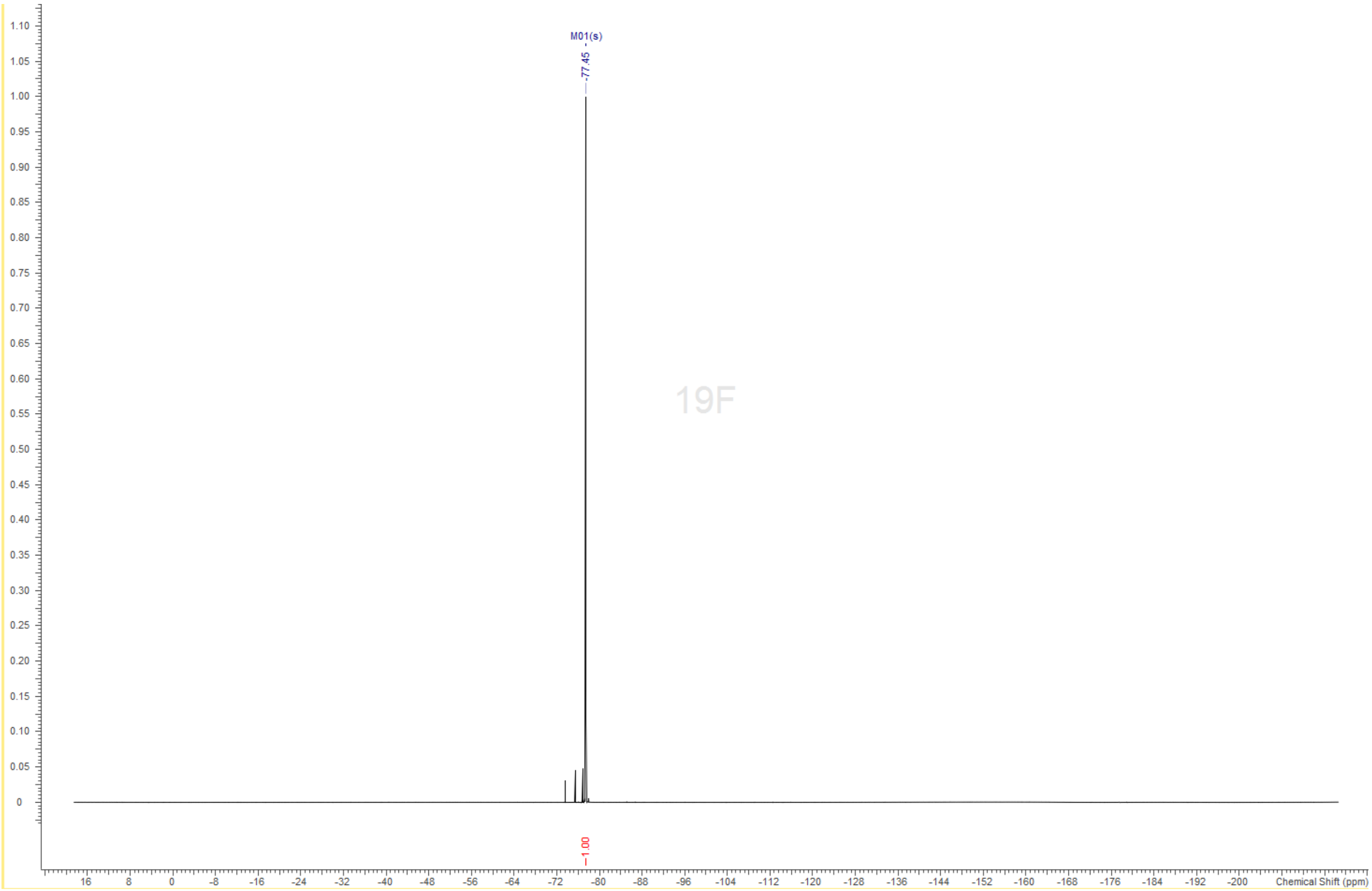
19F
M01(s) -77.45
1.00
Chemical Shift (ppm)

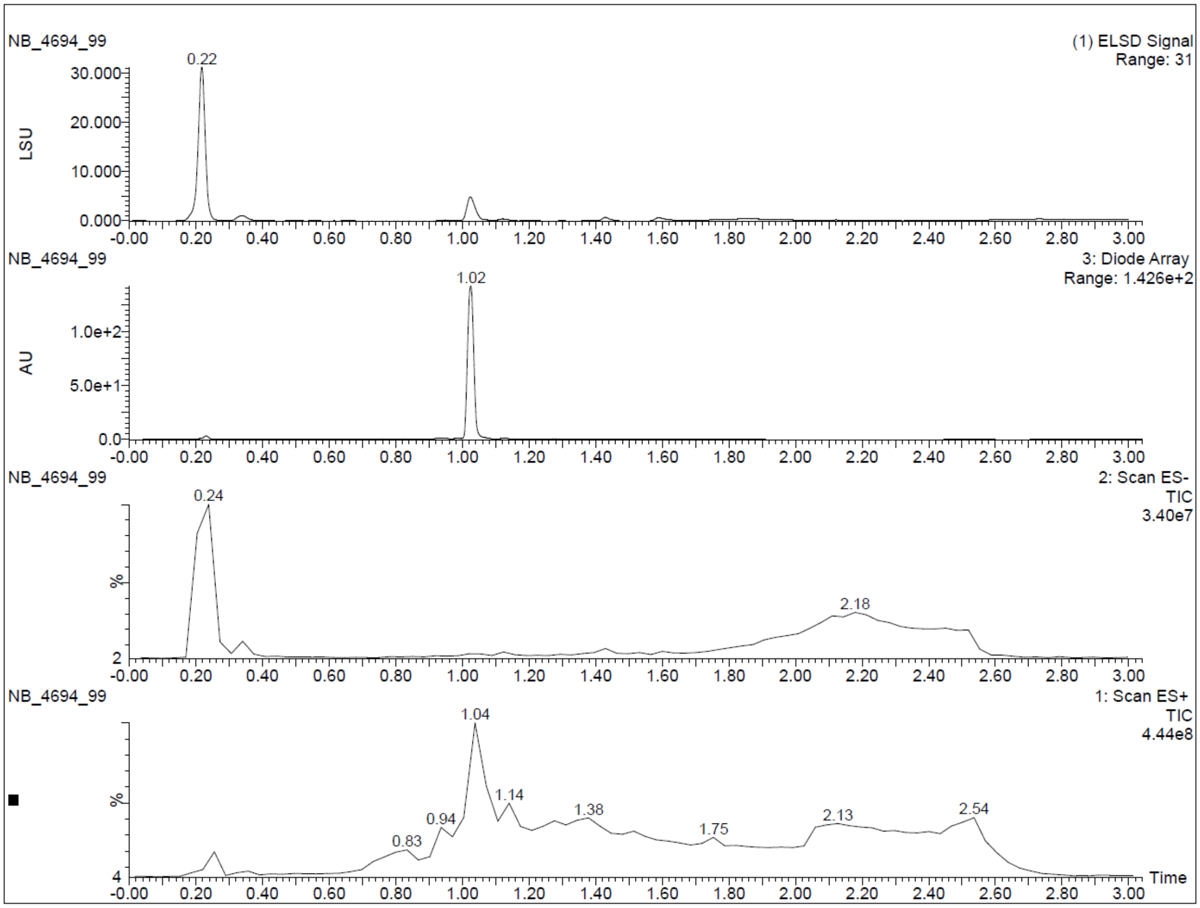
NB_4694_99
(1) ELSD Signal
Range: 31
0.22
LSU
NB_4694_99
3: Diode Array
Range: 1.426e+2
1.02
AU
NB_4694_99
2: Scan ES-
TIC
3.40e7
0.24
2.18
%
NB_4694_99
1: Scan ES+
TIC
4.44e8
1.04
1.14
1.38
0.94
0.83
1.75
2.13
2.54
%
Time

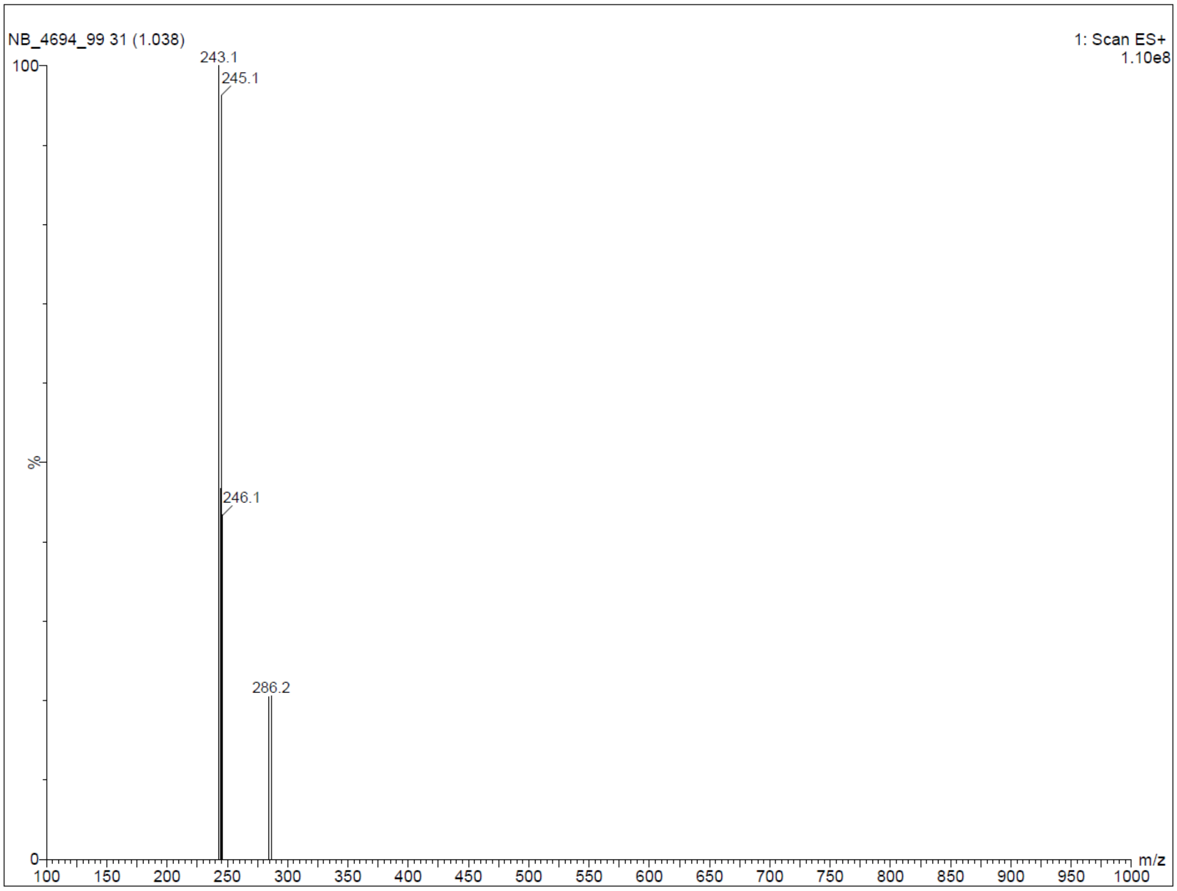
NB_4694_99 31 (1.038)
1: Scan ES+
1.10e8
243.1
245.1
246.1
286.2
%
m/z

3-(1-Methyl-1H-indol-3-yl)morpholine, Free base (4at)

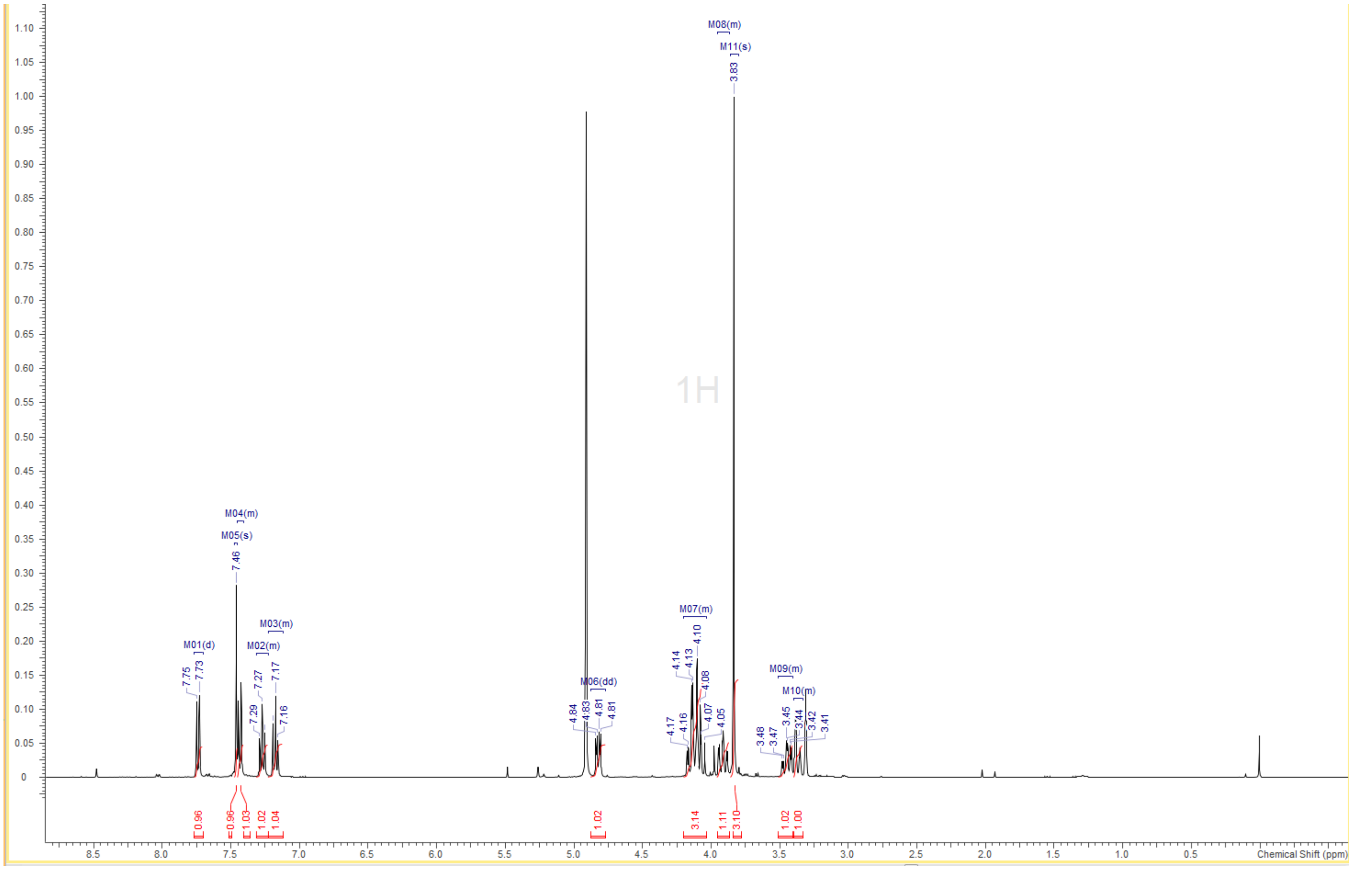

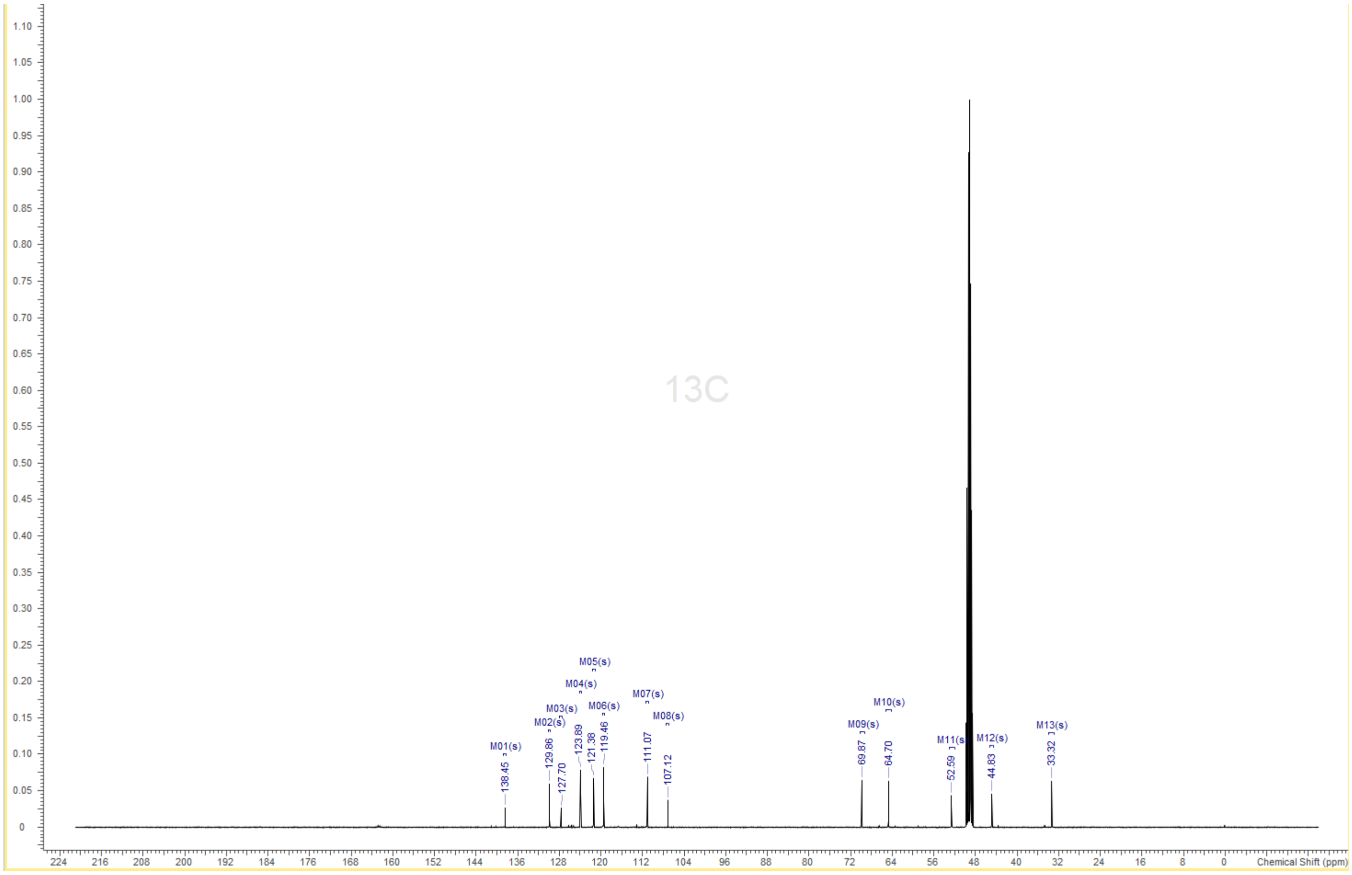

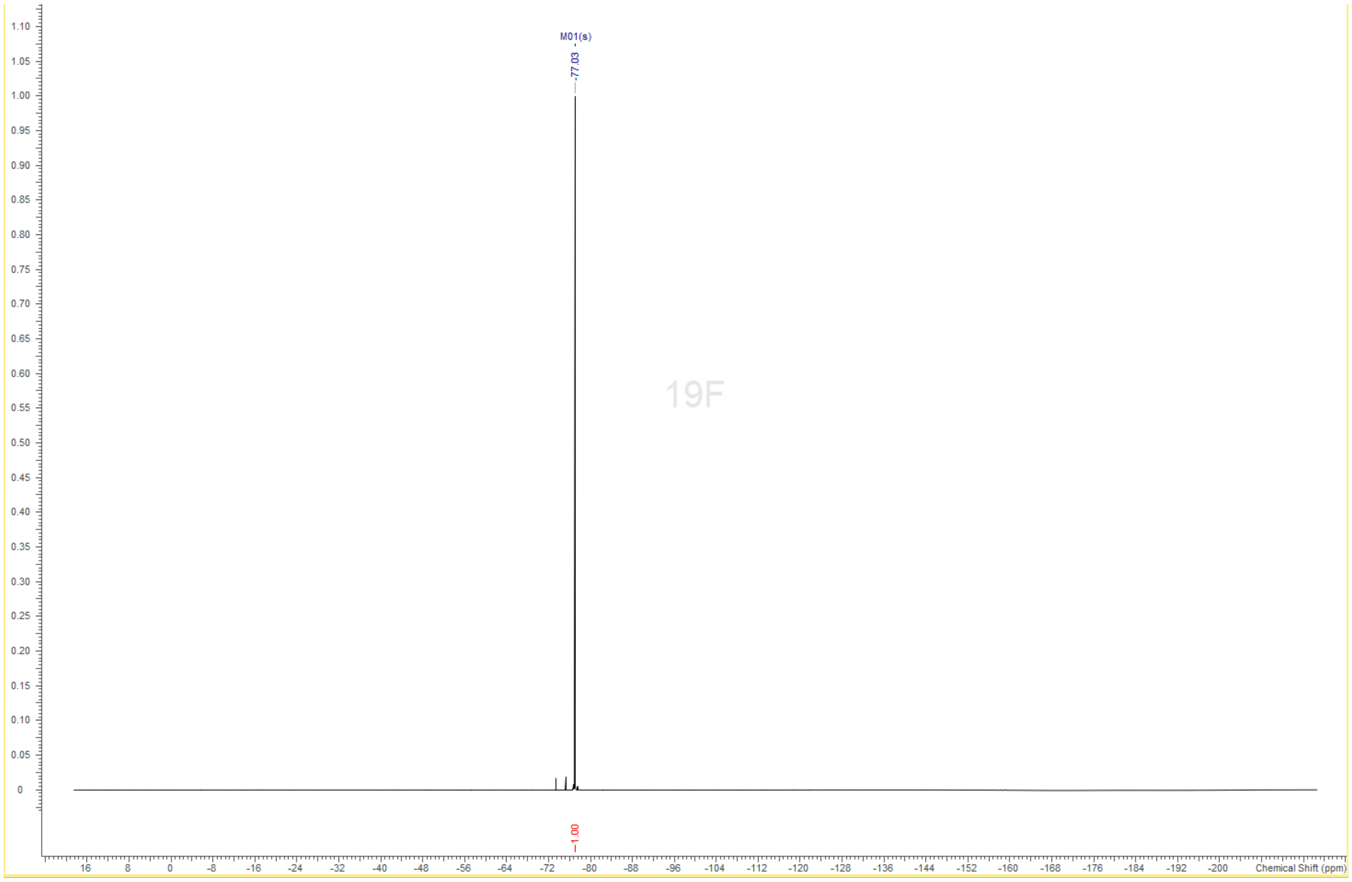
M01(s)
-77.03
19F
-1.00
Chemical Shift (ppm)

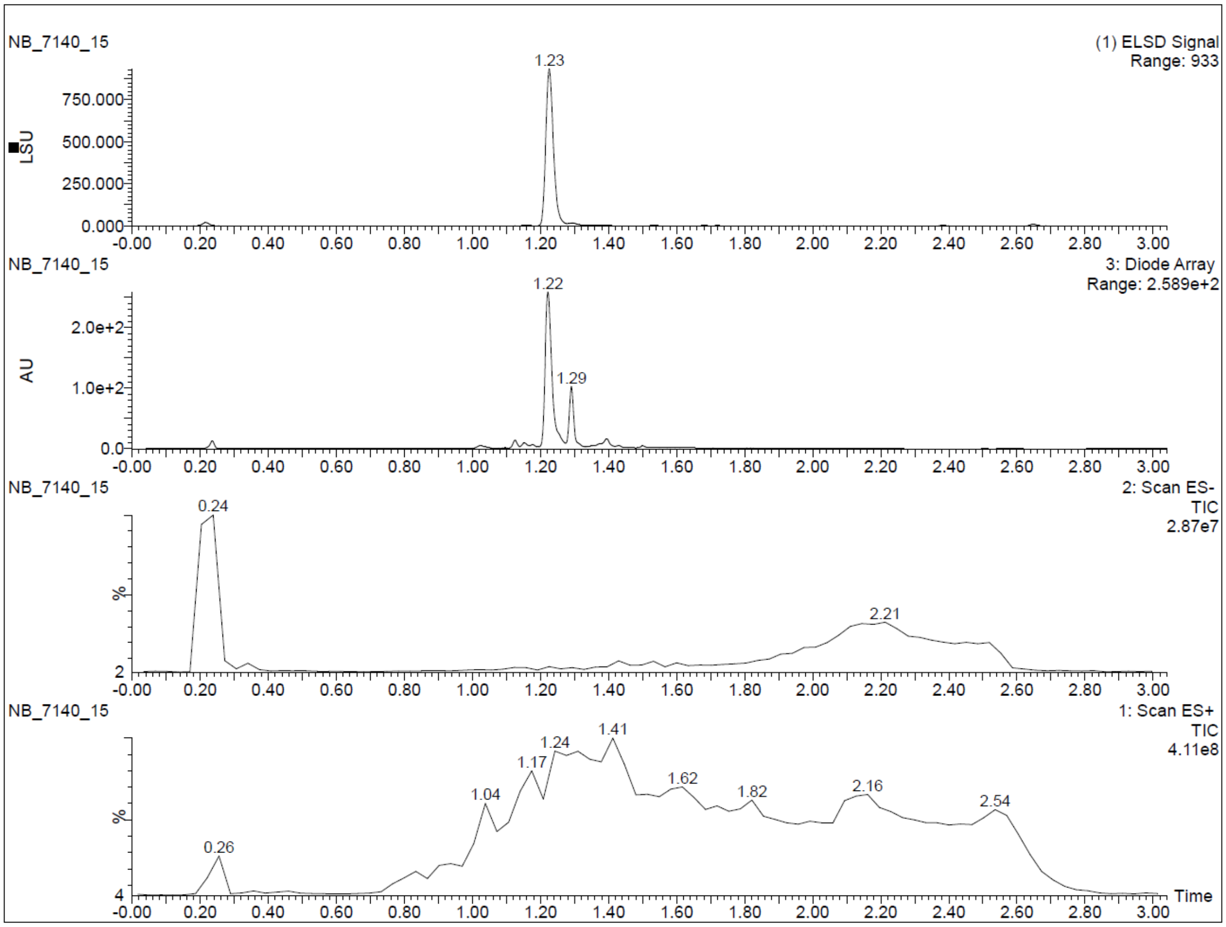
NB_7140_15
(1) ELSD Signal
Range: 933
1.23
LSU
NB_7140_15
3: Diode Array
Range: 2.589e+2
1.22
1.29
AU
NB_7140_15
2: Scan ES-
TIC
2.87e7
0.24
2.21
NB_7140_15
1: Scan ES+
TIC
4.11e8
0.26
1.04
1.17
1.24
1.41
1.62
1.82
2.16
2.54
Time

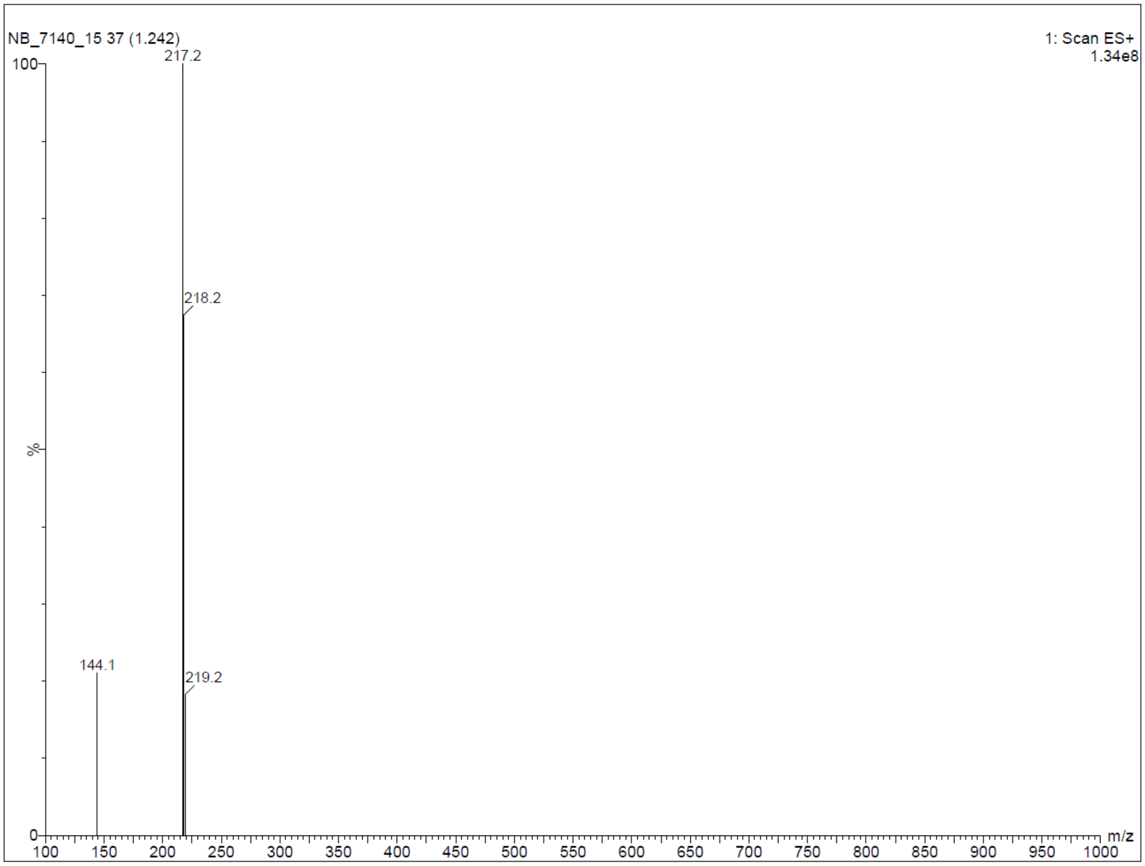

3-(Thiophen-2-yl)morpholine, Free base (4ao)

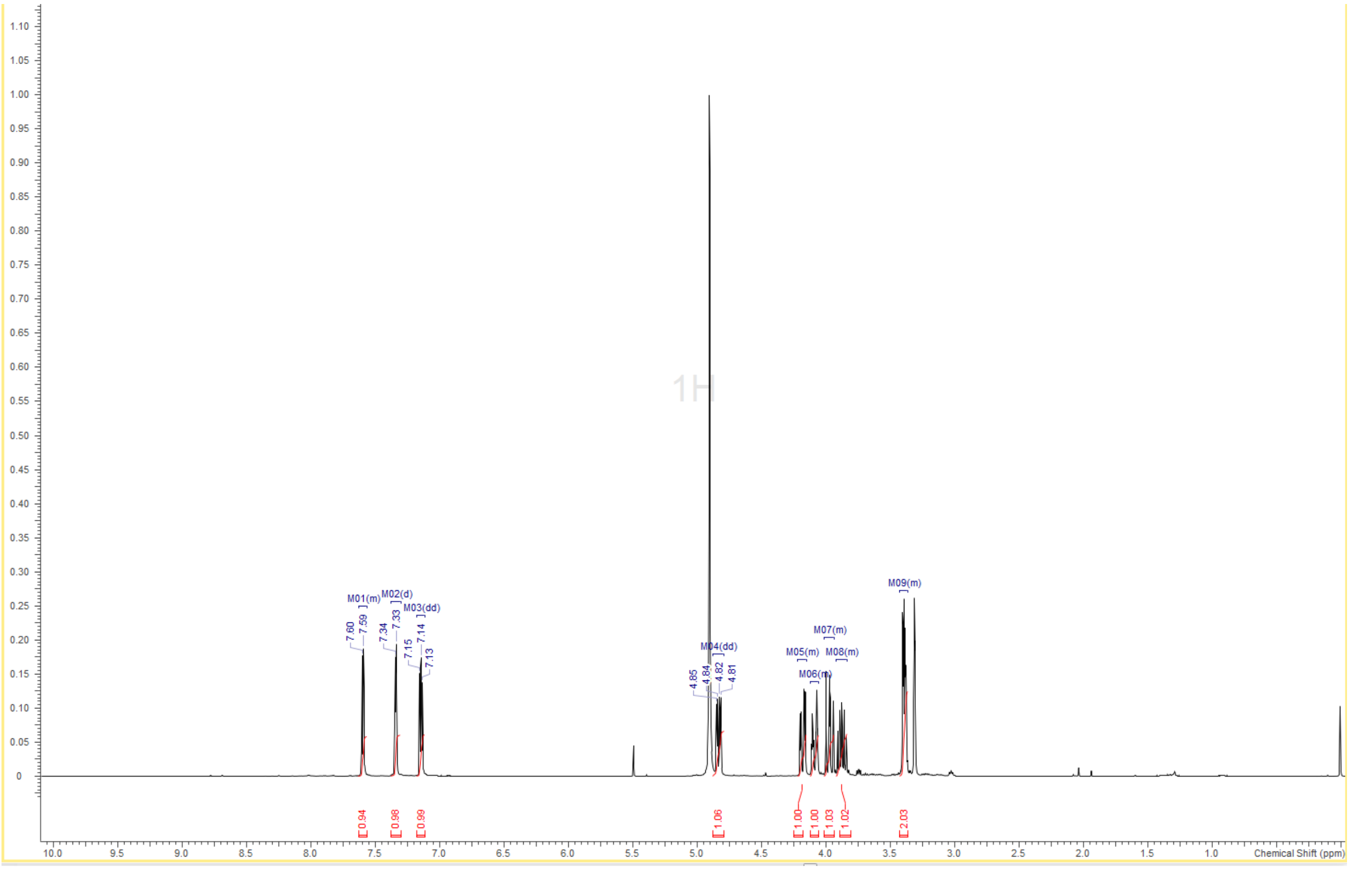

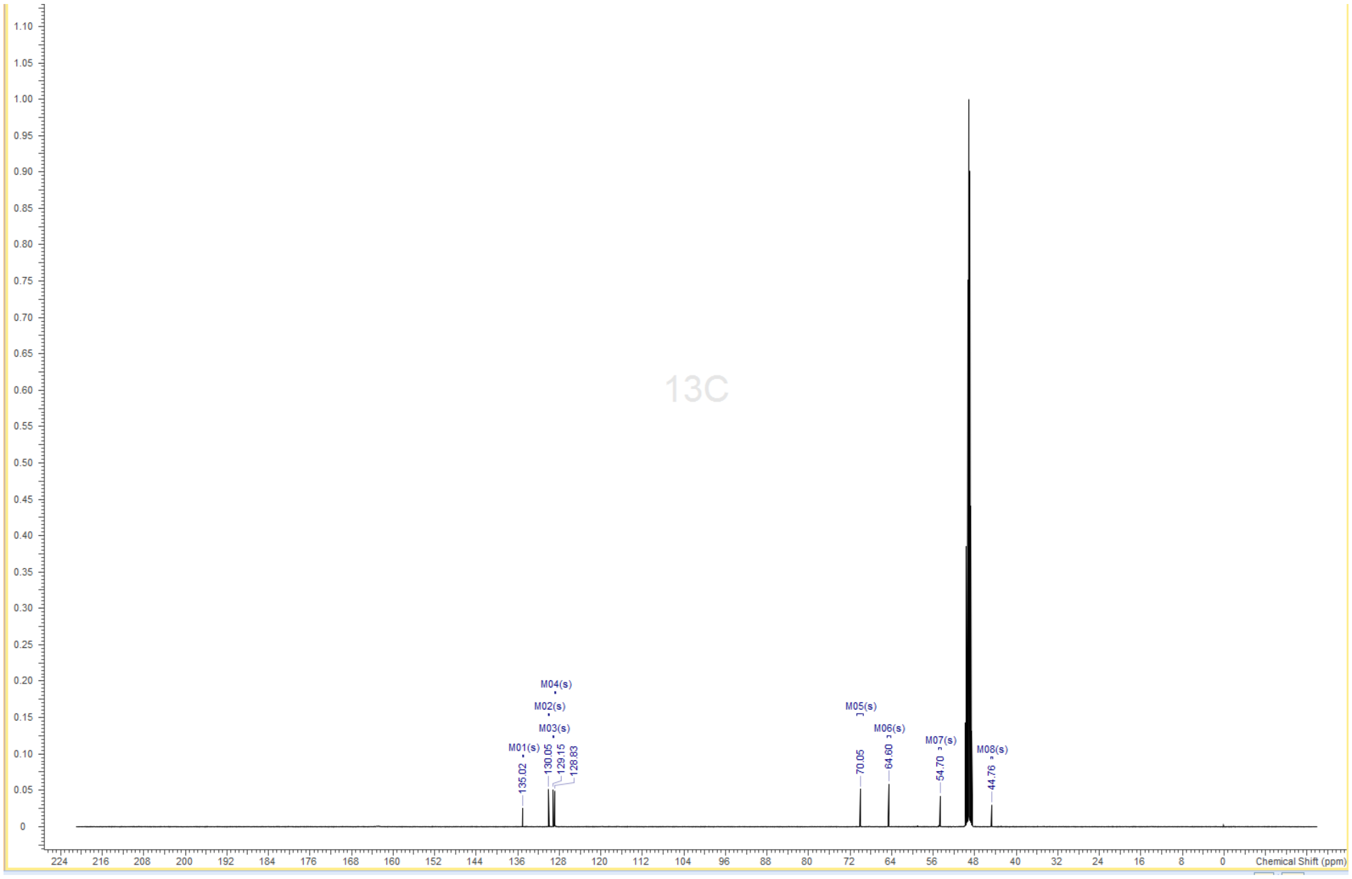
13C
M01(s)
135.02
M02(s)
130.05
M03(s)
129.15
M04(s)
128.83
M05(s)
70.05
M06(s)
64.60
M07(s)
54.70
M08(s)
44.76
Chemical Shift (ppm)

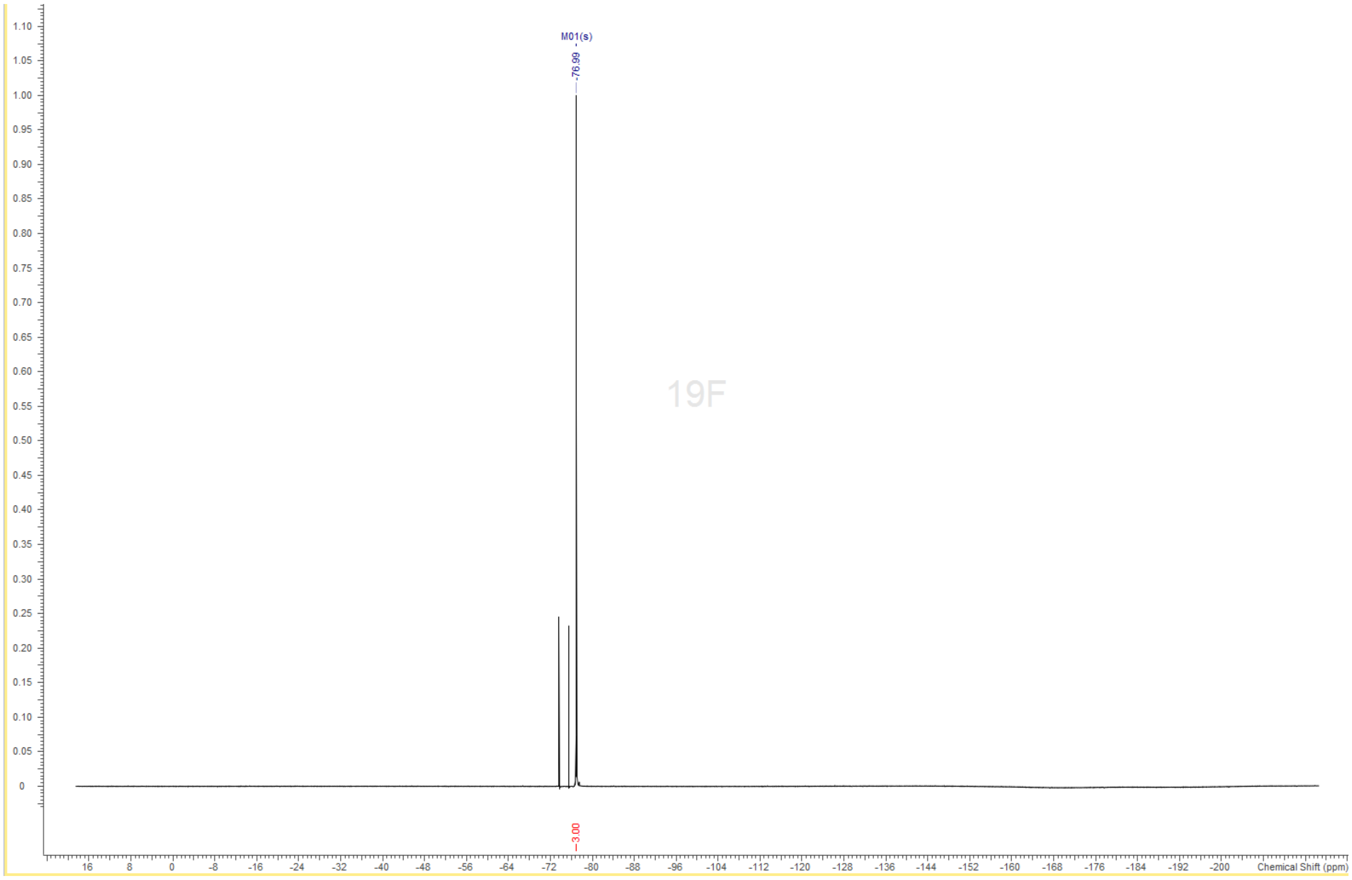
19F
M01(s)
-76.99
3.00
Chemical Shift (ppm)

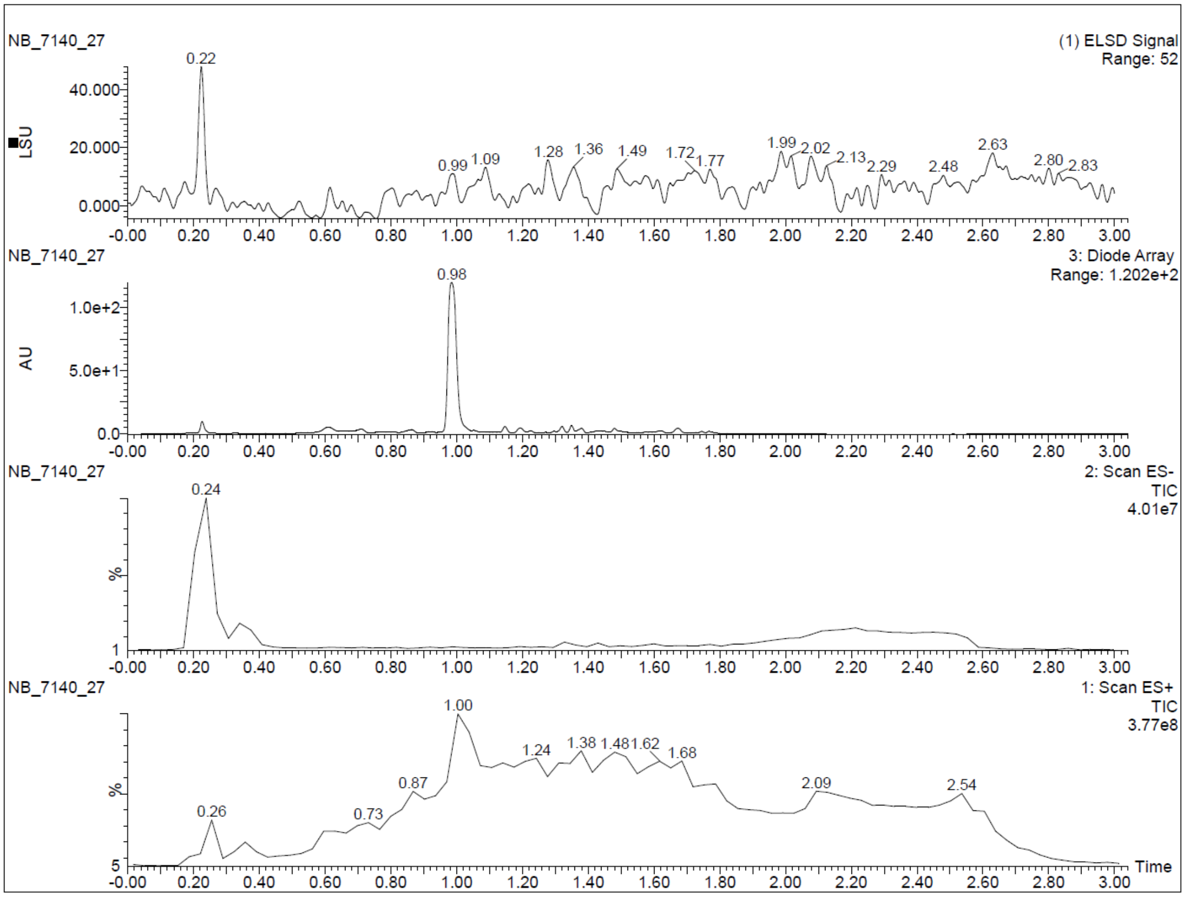

NB_7140_27
(1) ELSD Signal
Range: 52
LSU
0.22
0.99 1.09 1.28 1.36 1.49 1.72 1.77 1.99 2.02 2.13 2.29 2.48 2.63 2.80 2.83
3: Diode Array
Range: 1.202e+2
AU
0.98
2: Scan ES-
TIC
4.01e7
0.24
1: Scan ES+
TIC
3.77e8
0.26 0.73 0.87 1.00 1.24 1.38 1.48 1.62 1.68 2.09 2.54
Time

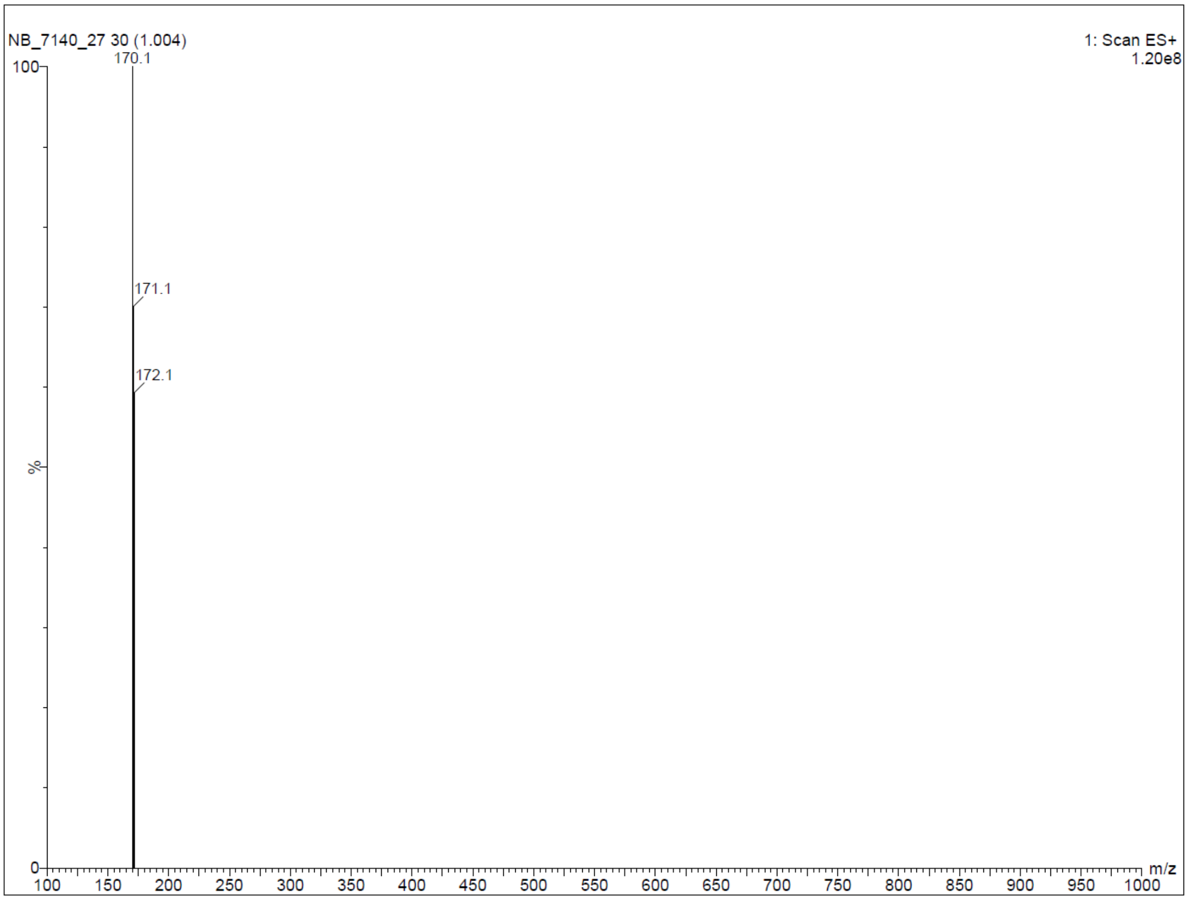

NB_7140_27 30 (1.004)
1: Scan ES+
1.20e8
170.1
171.1
172.1
%
m/z

3-(1-Methyl-1H-pyrazol-5-yl)morpholine, Free base (4ap)

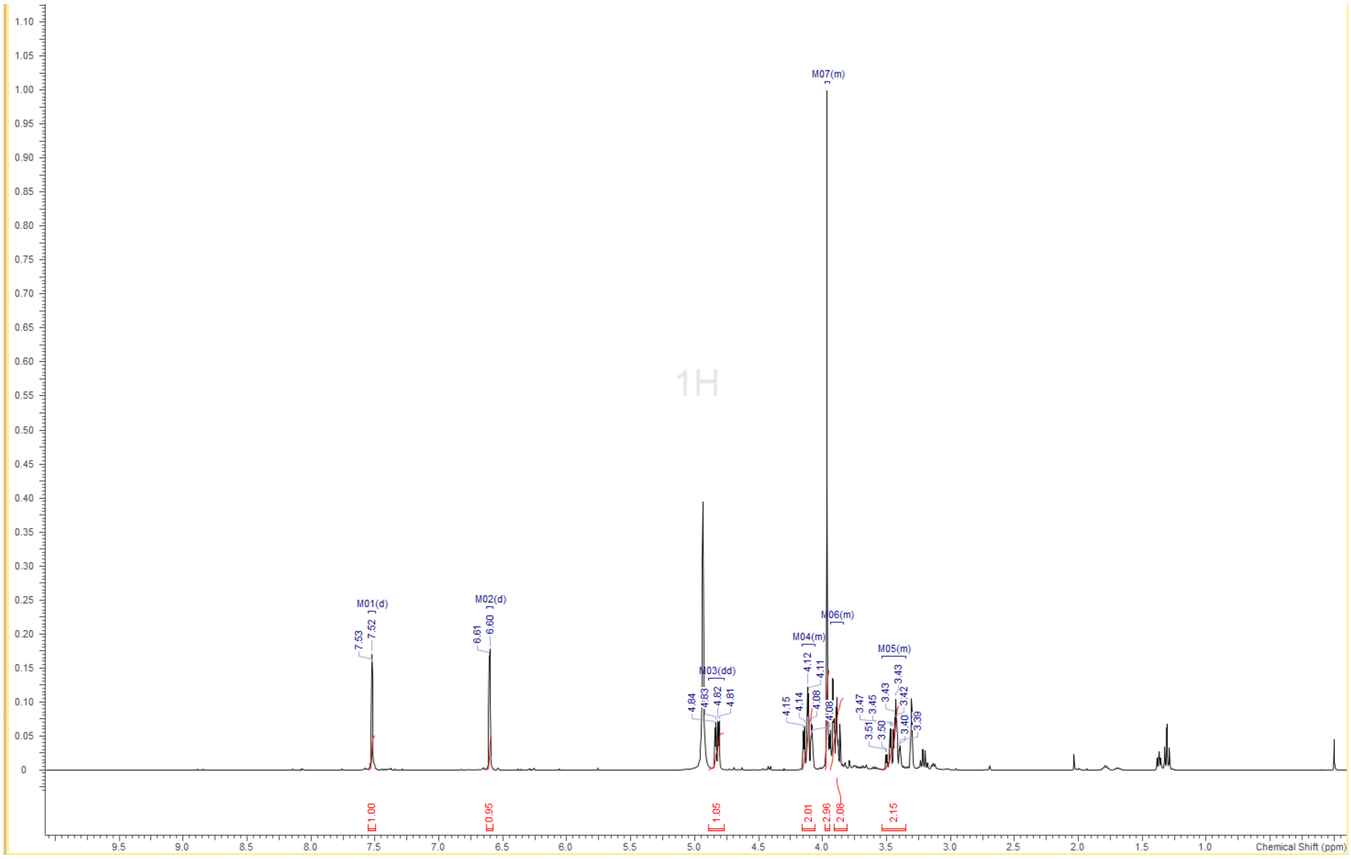

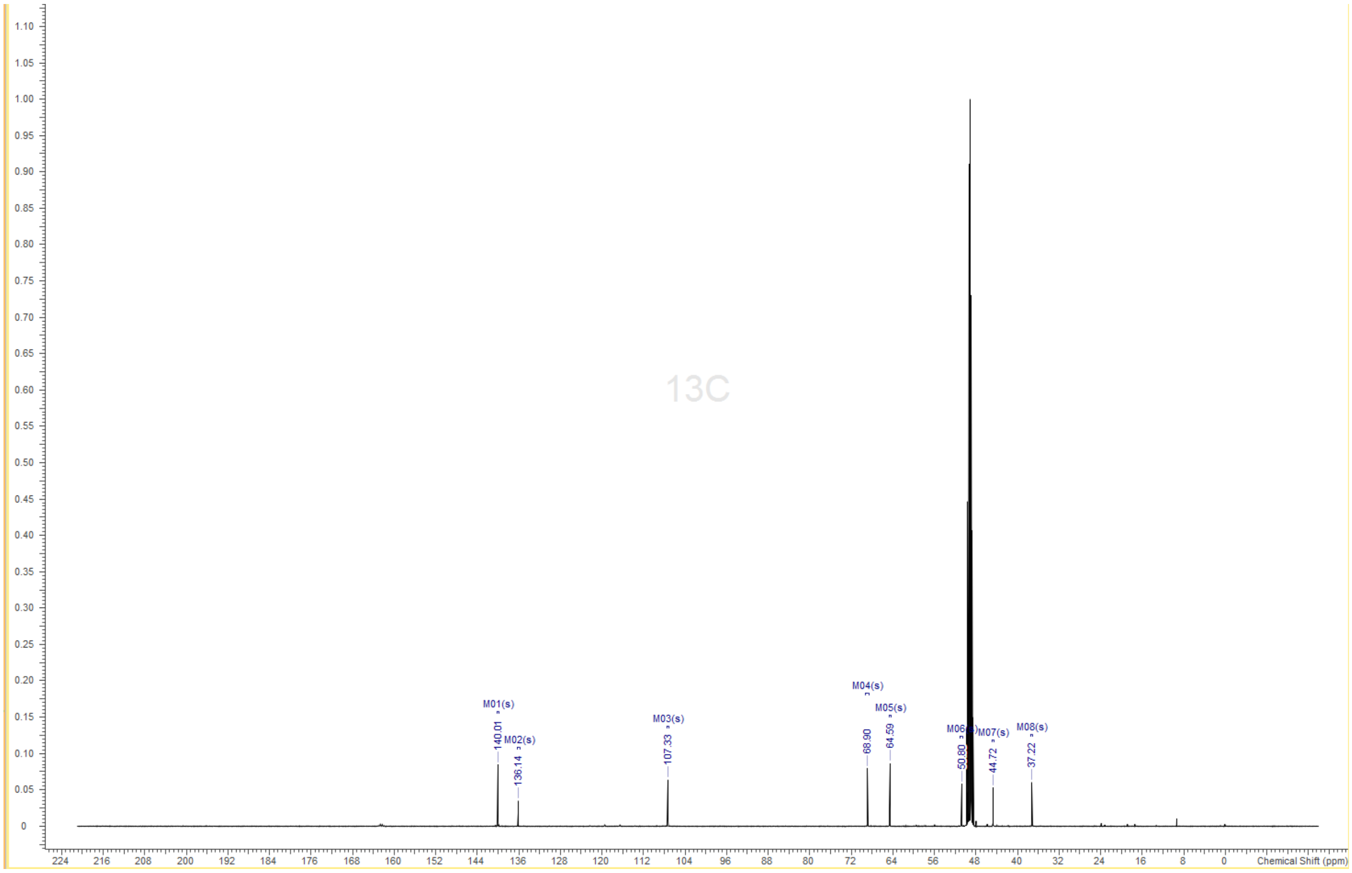

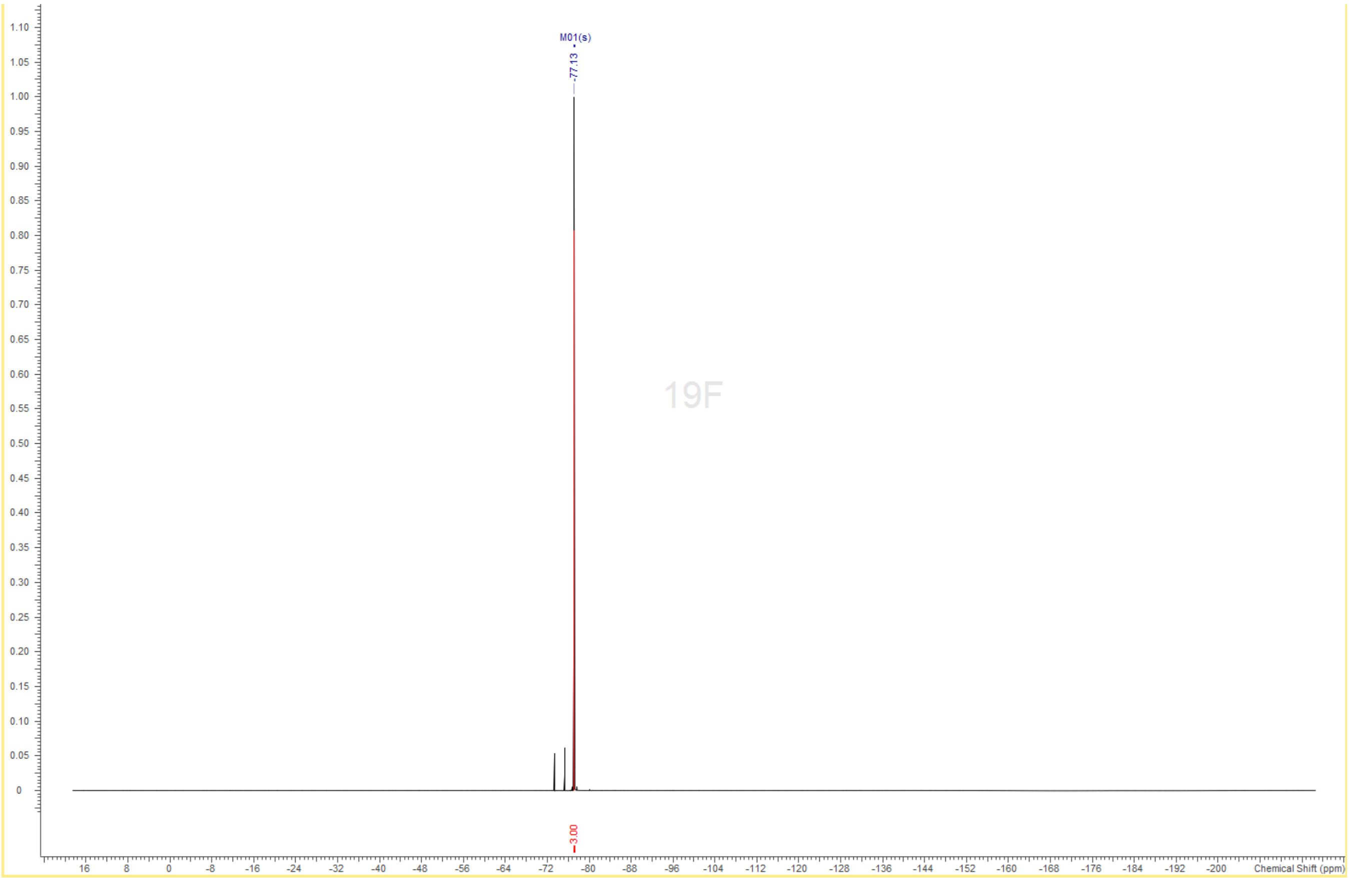
M01(s)
-77.13
19F
-3.00
Chemical Shift (ppm)

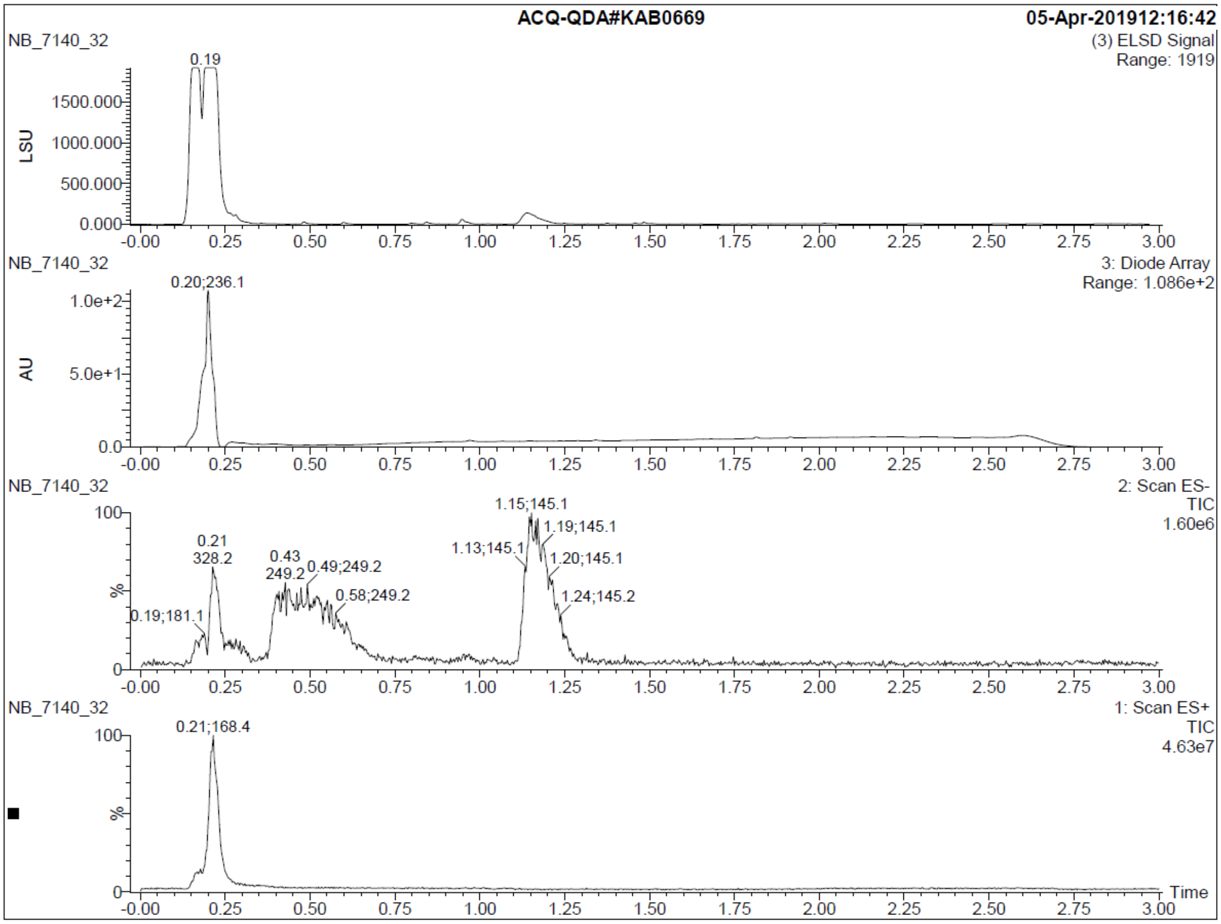
ACQ-QDA#KAB0669
05-Apr-201912:16:42
NB_7140_32
(3) ELSD Signal
Range: 1919
0.19
LSU
3: Diode Array
Range: 1.086e+2
0.20;236.1
AU
2: Scan ES-
TIC
1.60e6
0.21
328.2
0.43
249.2
0.49;249.2
0.58;249.2
0.19;181.1
1.15;145.1
1.13;145.1
1.19;145.1
1.20;145.1
1.24;145.2
1: Scan ES+
TIC
4.63e7
0.21;168.4
Time

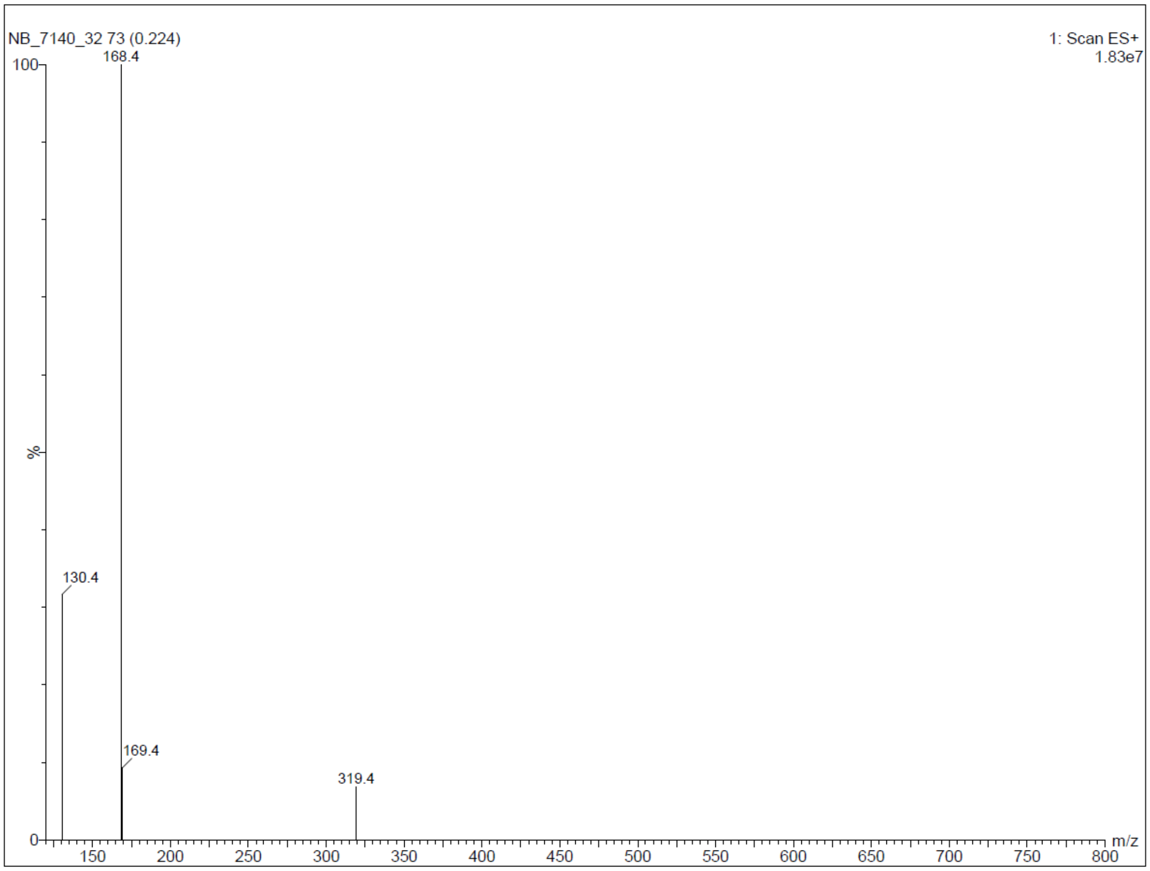

3-(Furan-2-yl)morpholine, Free base (4an)

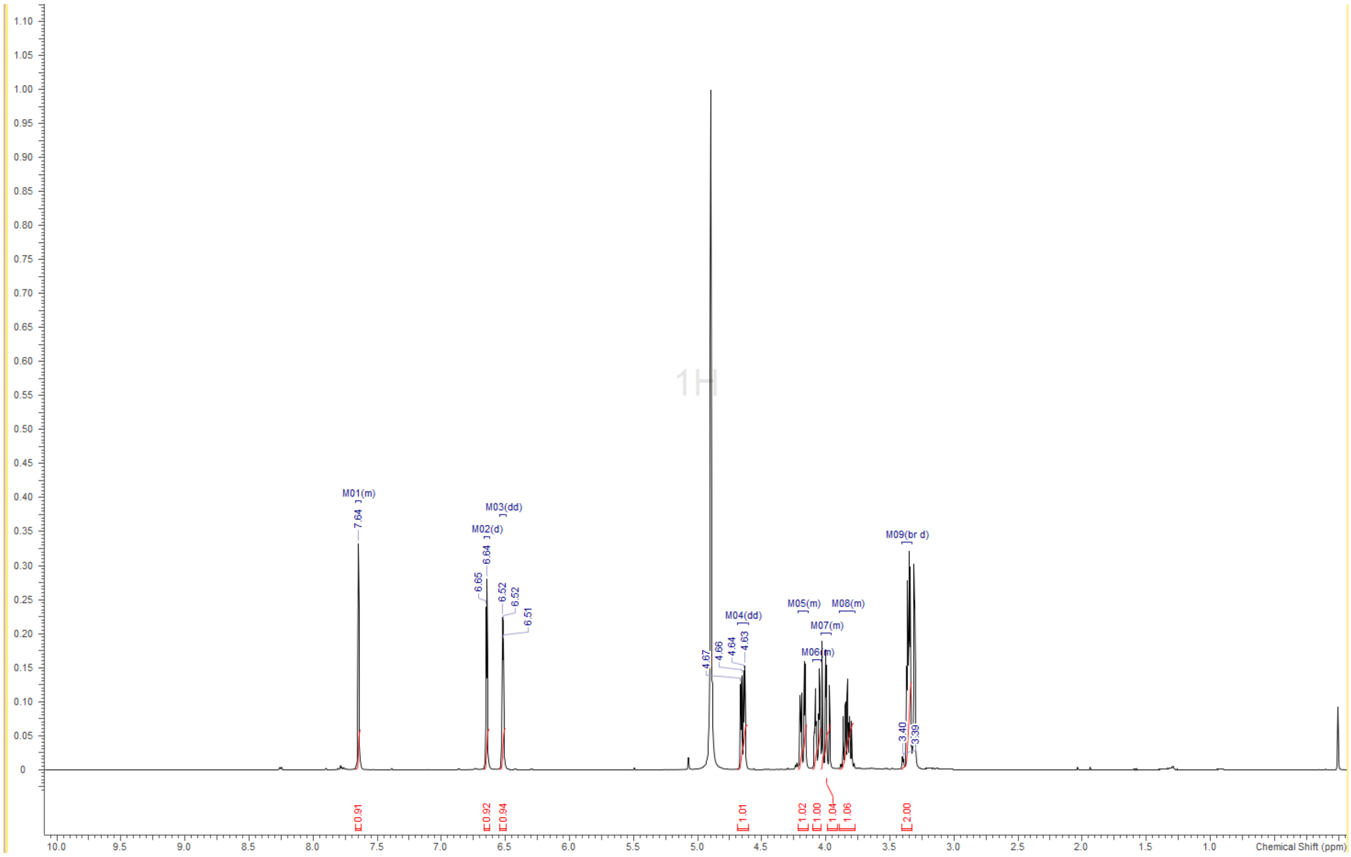

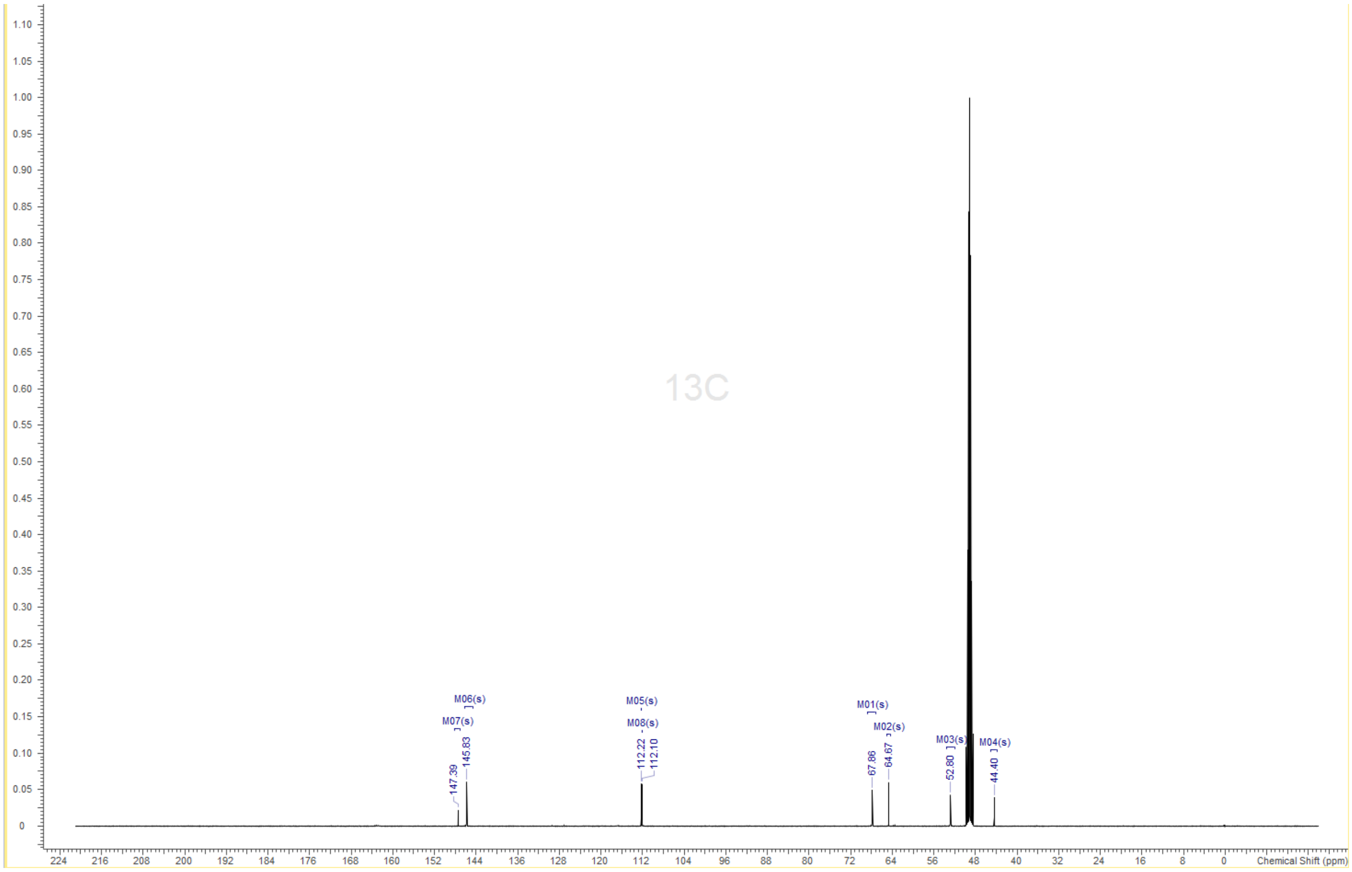
13C
M06(s)
M07(s)
147.39
145.83
M05(s)
M08(s)
112.22
112.10
M01(s)
M02(s)
67.86
64.67
M03(s)
52.80
M04(s)
44.40
Chemical Shift (ppm)

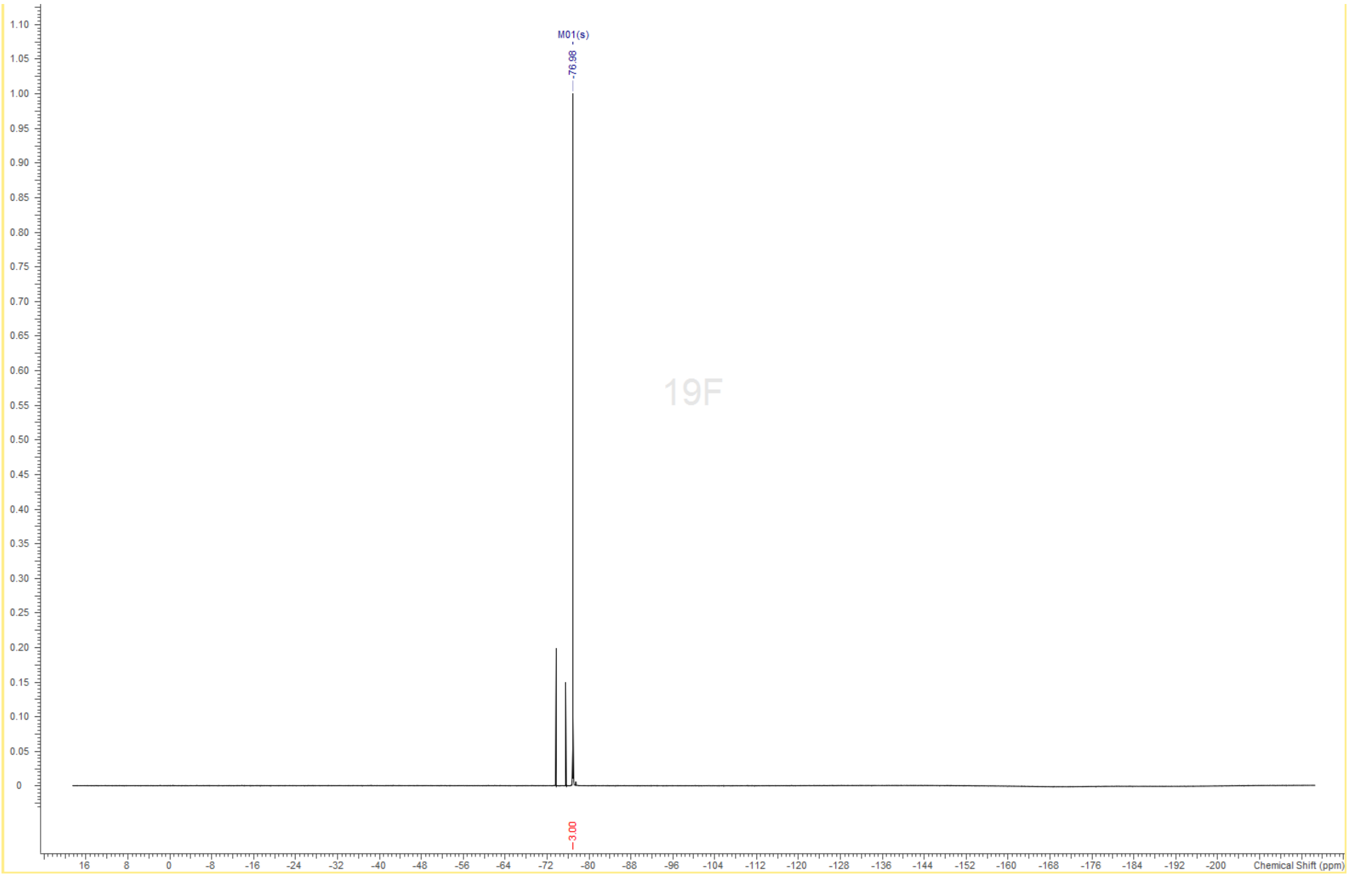
19F
M01(s)
-76.98
3.00
Chemical Shift (ppm)

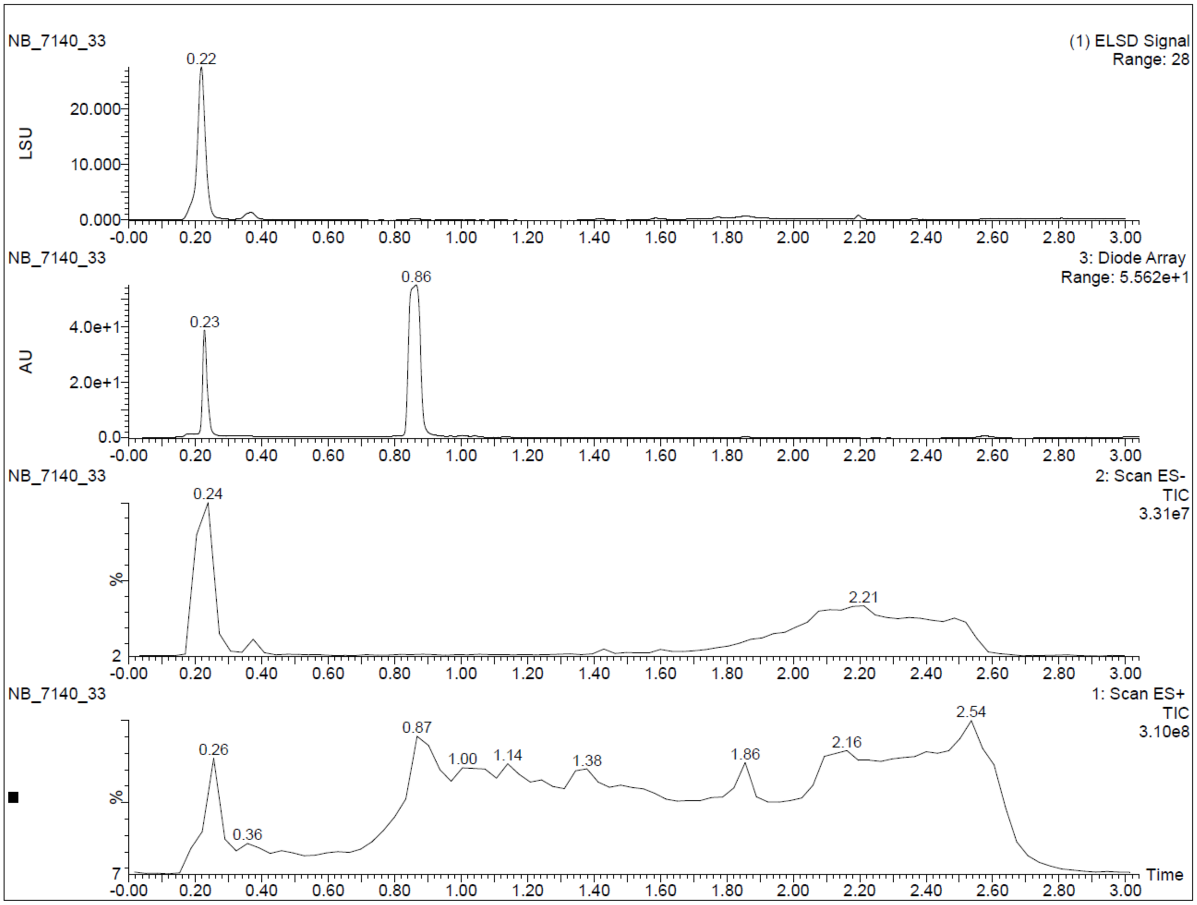
NB_7140_33
(1) ELSD Signal
Range: 28
LSU
0.22
NB_7140_33
3: Diode Array
Range: 5.562e+1
AU
0.23
0.86
NB_7140_33
2: Scan ES-
TIC
3.31e7
0.24
2.21
NB_7140_33
1: Scan ES+
TIC
3.10e8
0.26
0.36
0.87
1.00
1.14
1.38
1.86
2.16
2.54
Time

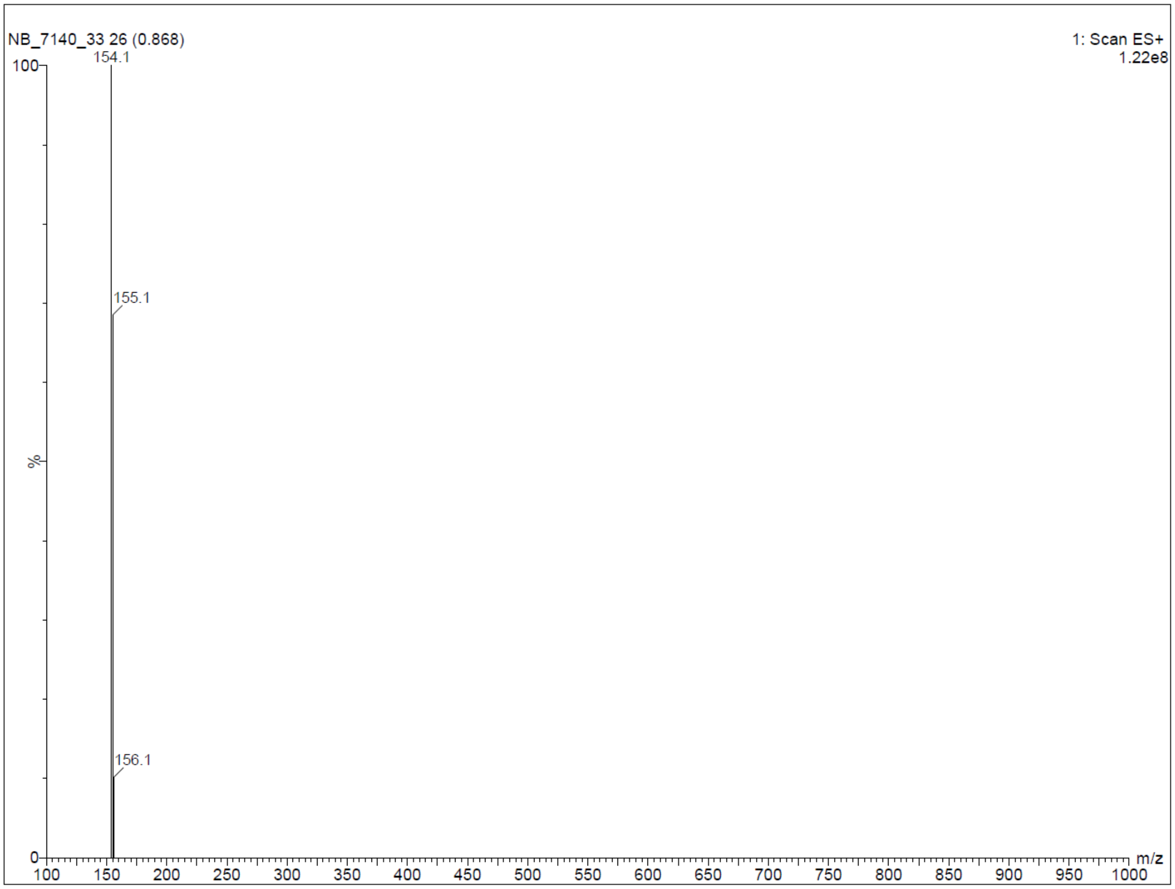
NB_7140_33 26 (0.868)
1: Scan ES+
1.22e8
154.1
155.1
156.1
%
m/z

tert-Butyl 6-(morpholin-3-yl)-indole-1-carboxylate, TFA salt (4as)

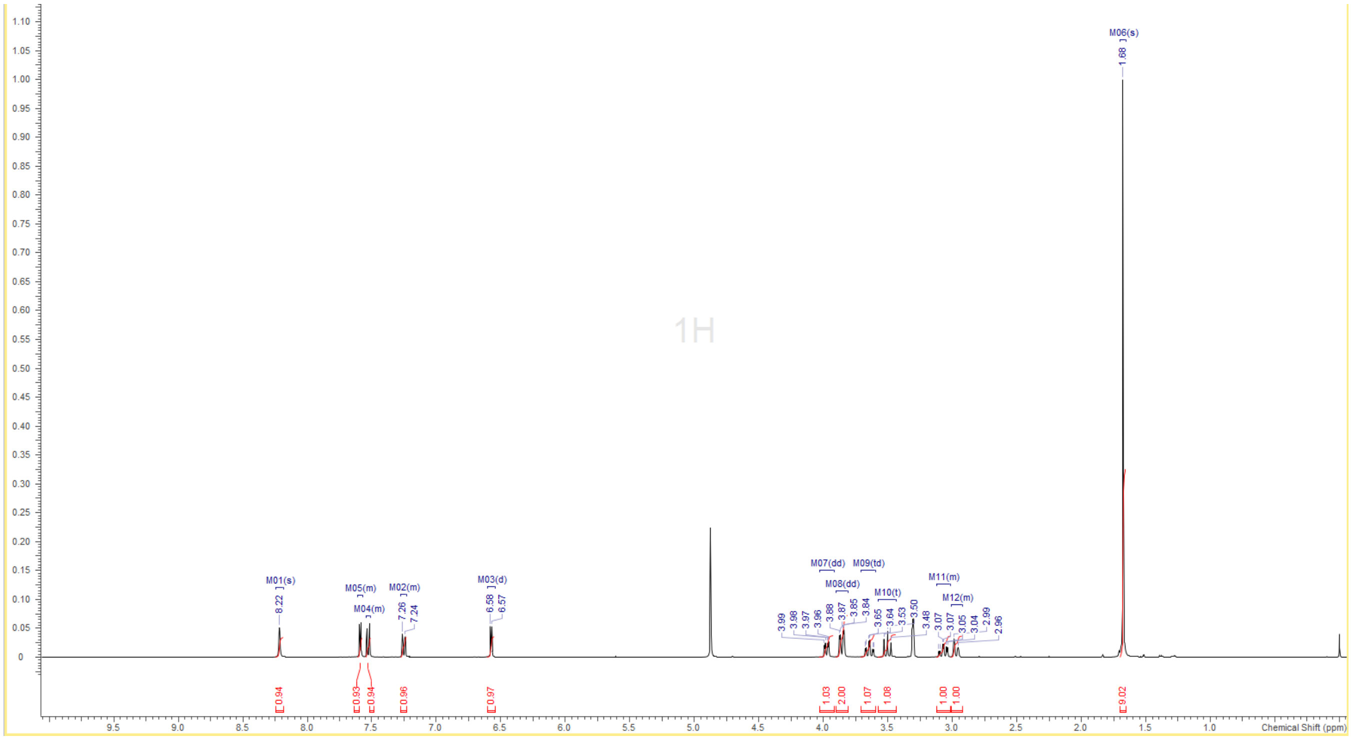

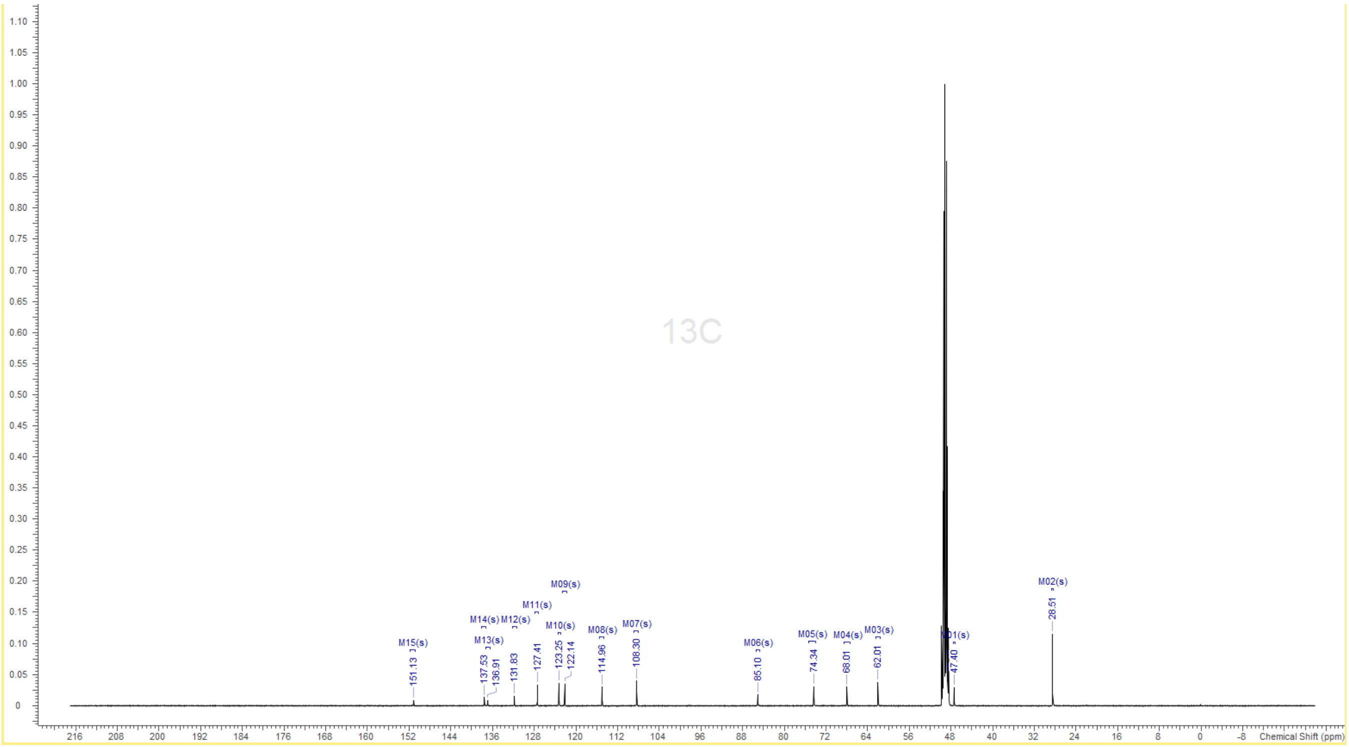

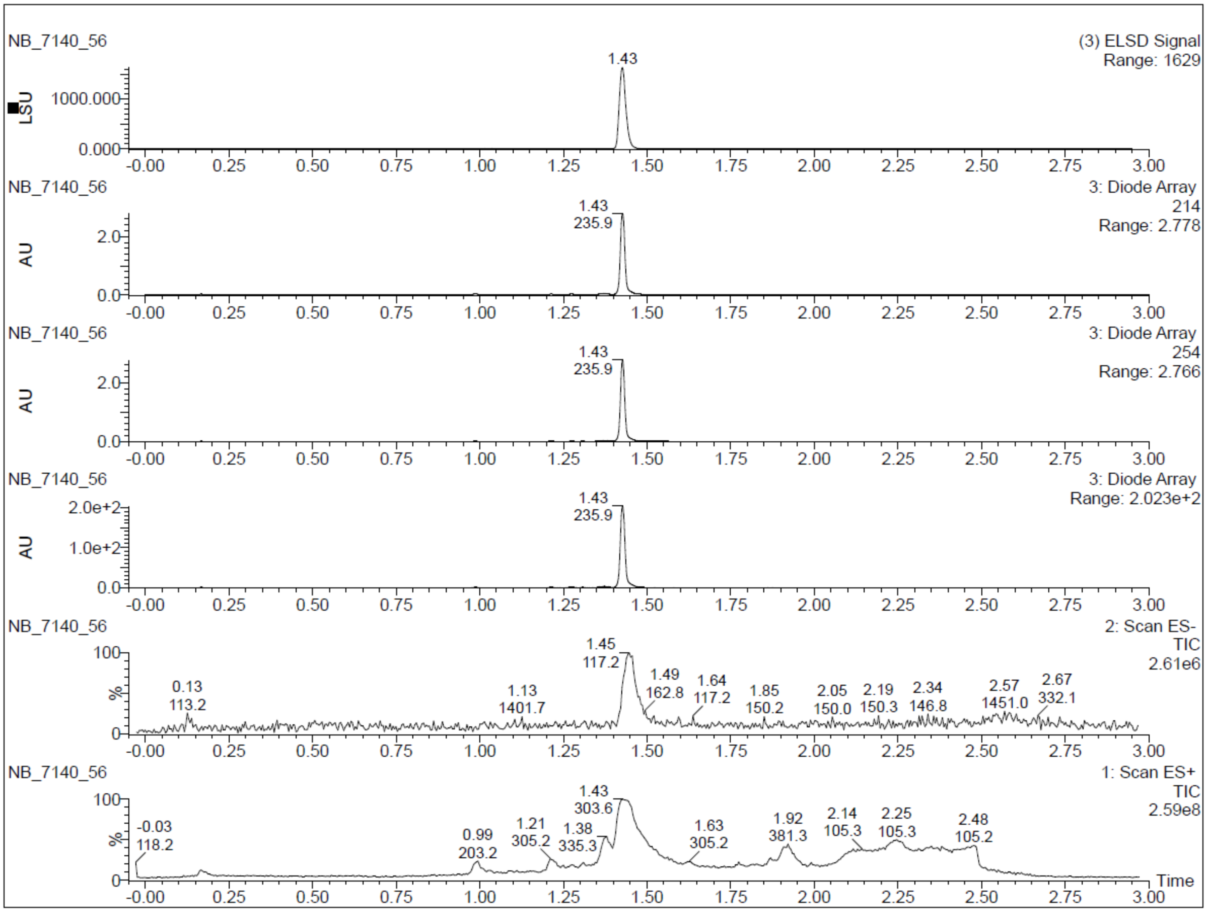

NB_7140_56
(3) ELSD Signal
Range: 1629
LSU
1.43
3: Diode Array
214
Range: 2.778
1.43
235.9
3: Diode Array
254
Range: 2.766
3: Diode Array
Range: 2.023e+2
AU
2: Scan ES-
TIC
2.61e6
1.45
117.2
1: Scan ES+
TIC
2.59e8
1.43
303.6
Time

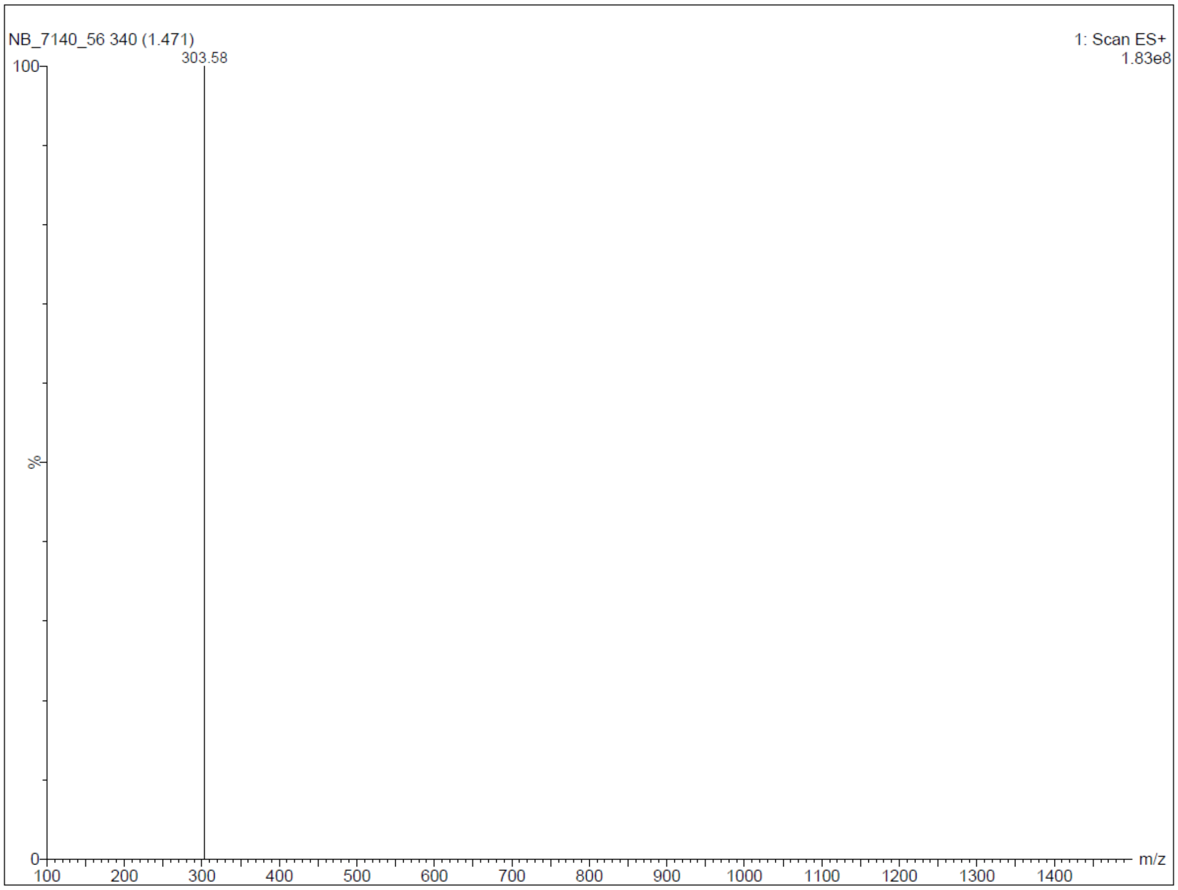

NB_7140_56 340 (1.471)
1: Scan ES+
1.83e8
303.58
%
m/z

3-(Benzo[d][1,3]dioxol-5-yl)morpholine, TFA salt (4am)

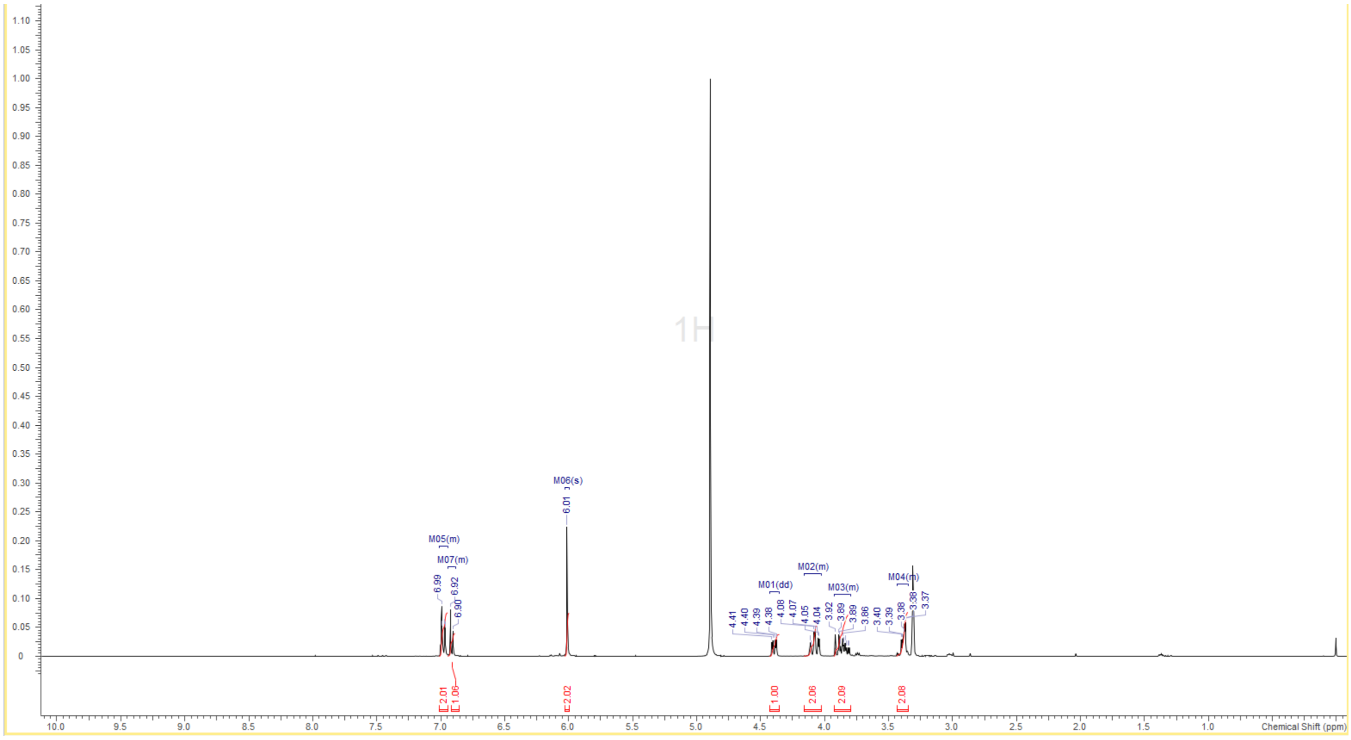

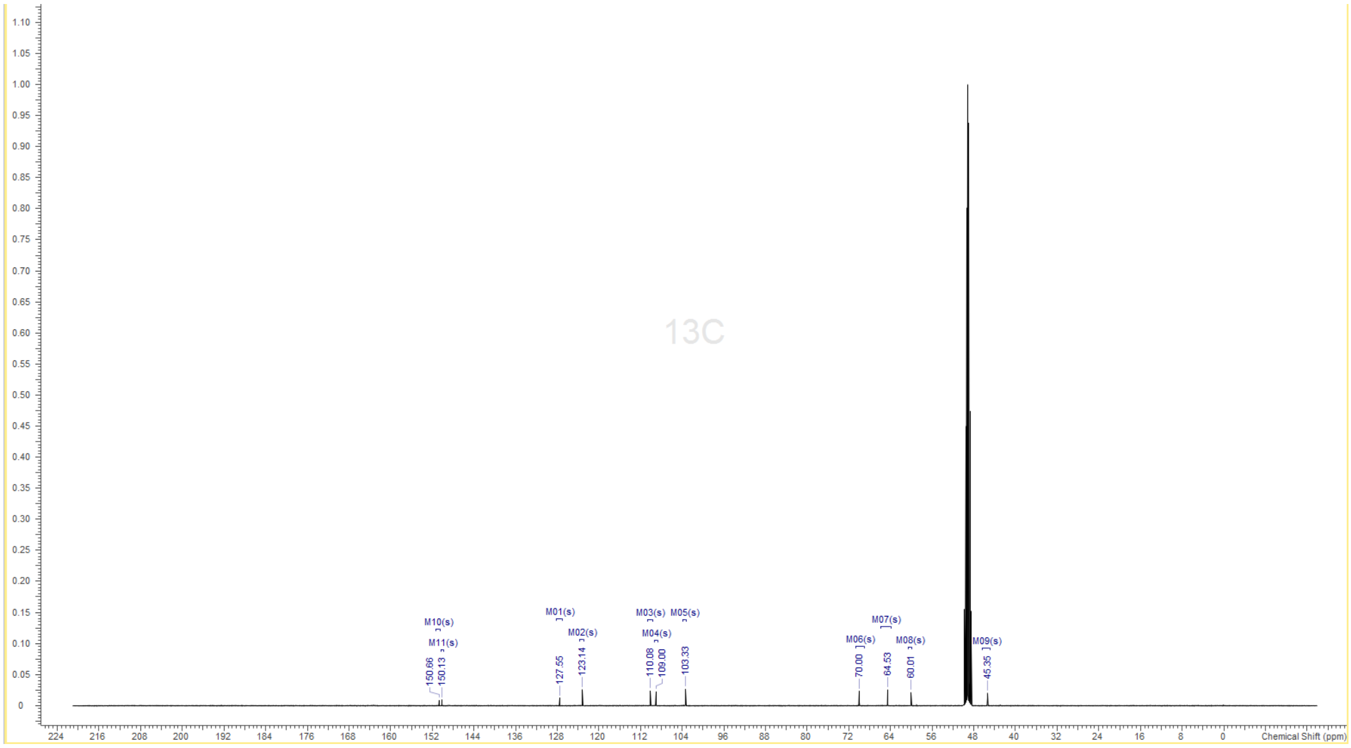

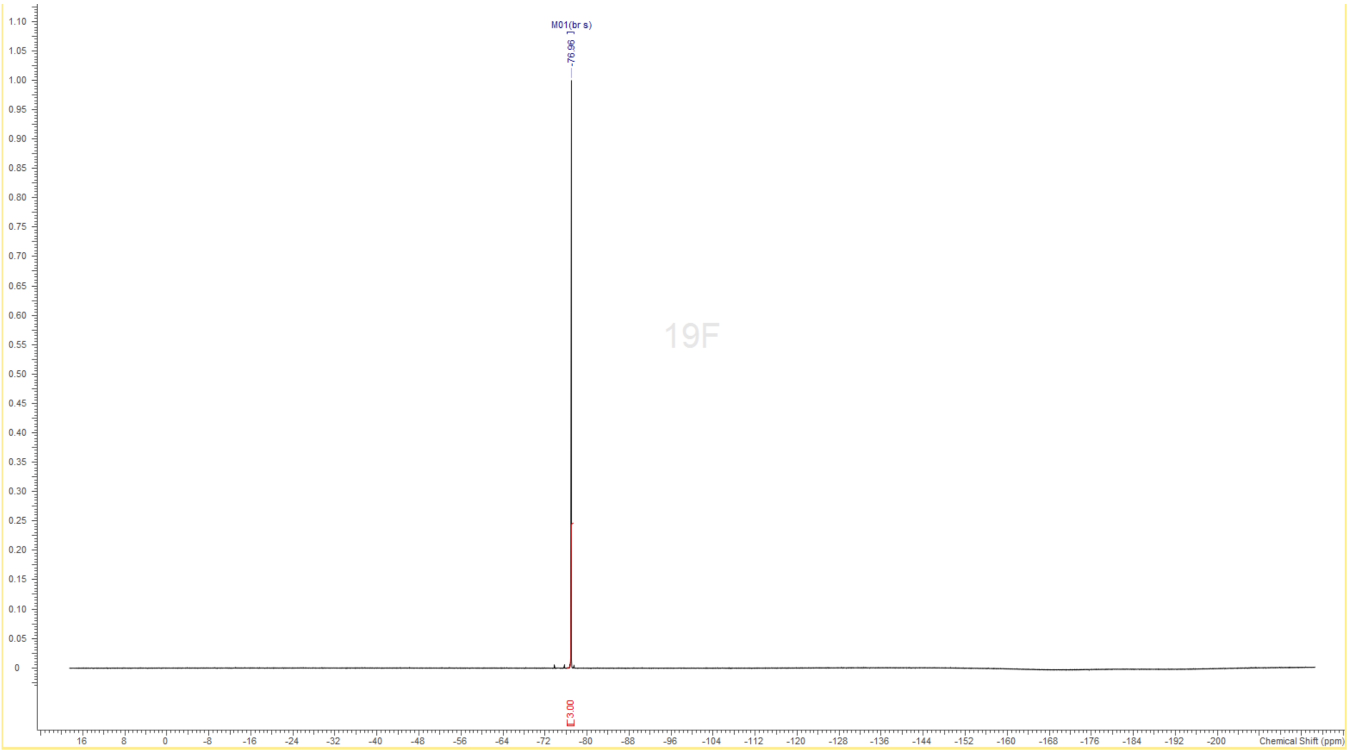
M01(br s)
-76.96
19F
3.00
Chemical Shift (ppm)

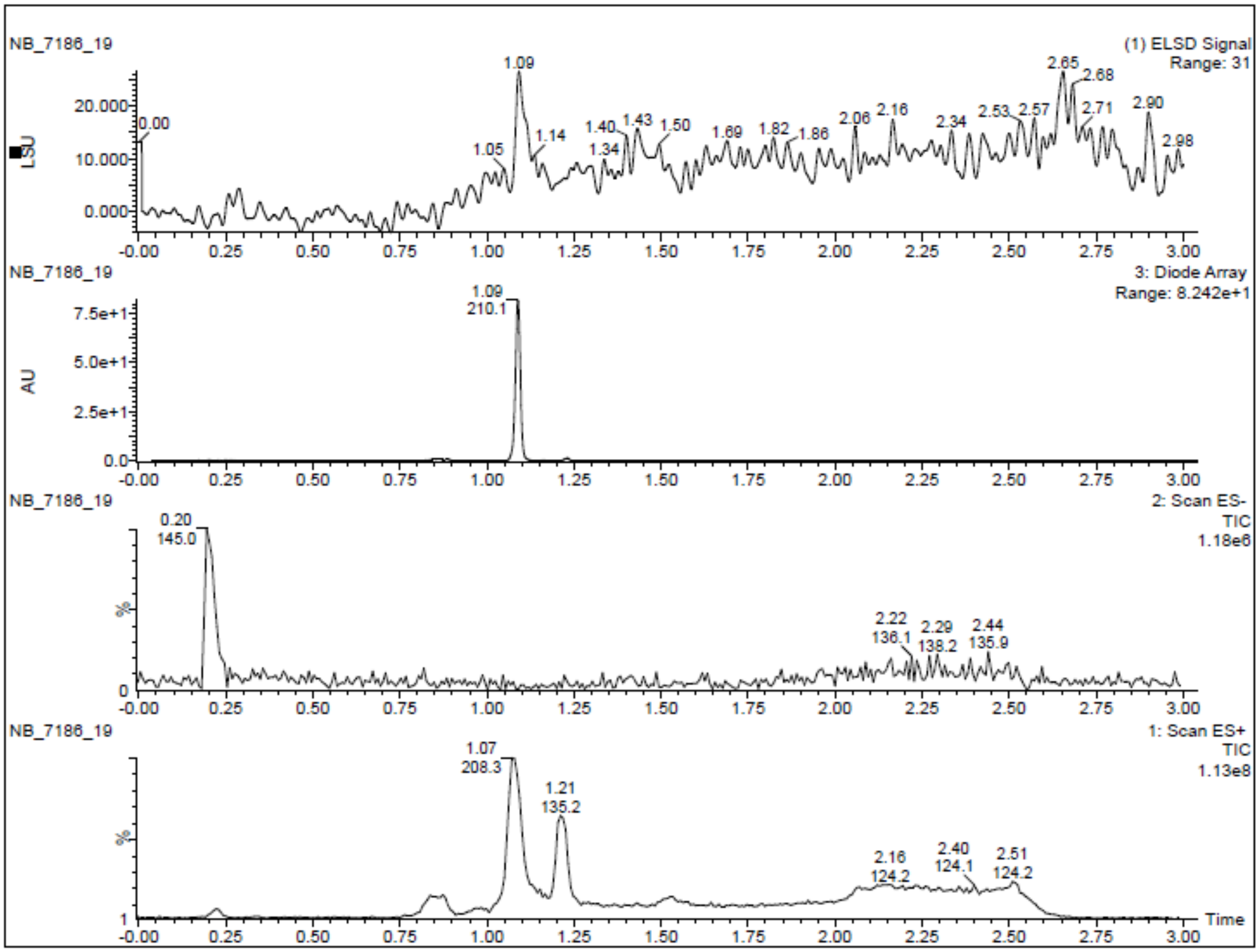
NB_7186_19
(1) ELSD Signal
Range: 31
LSU
NB_7186_19
3: Diode Array
Range: 8.242e+1
AU
1.09
210.1
NB_7186_19
2: Scan ES-
TIC
1.18e6
0.20
145.0
%
NB_7186_19
1: Scan ES+
TIC
1.13e8
1.07
208.3
1.21
135.2
Time

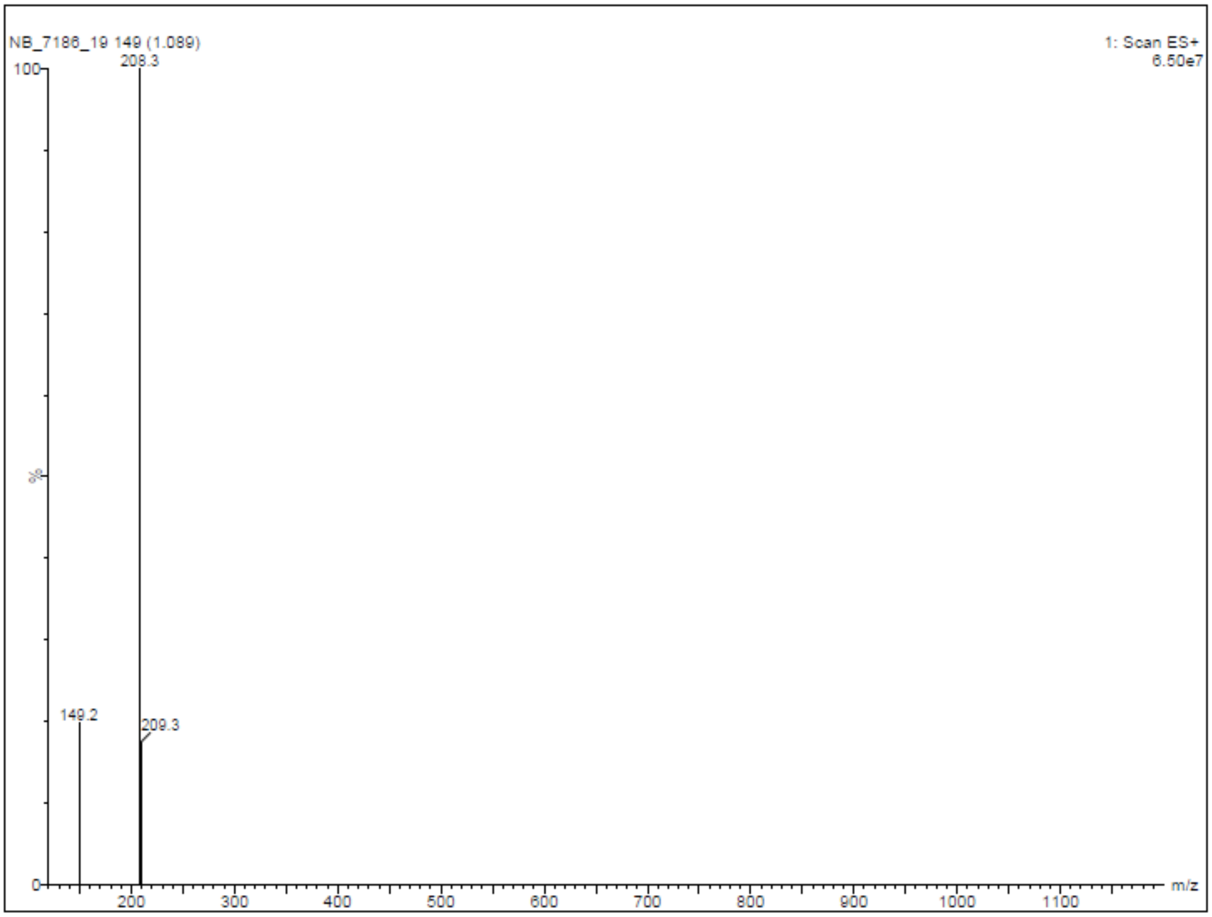

3-(Isoquinolin-5-yl)morpholine, TFA Salt (4aj)

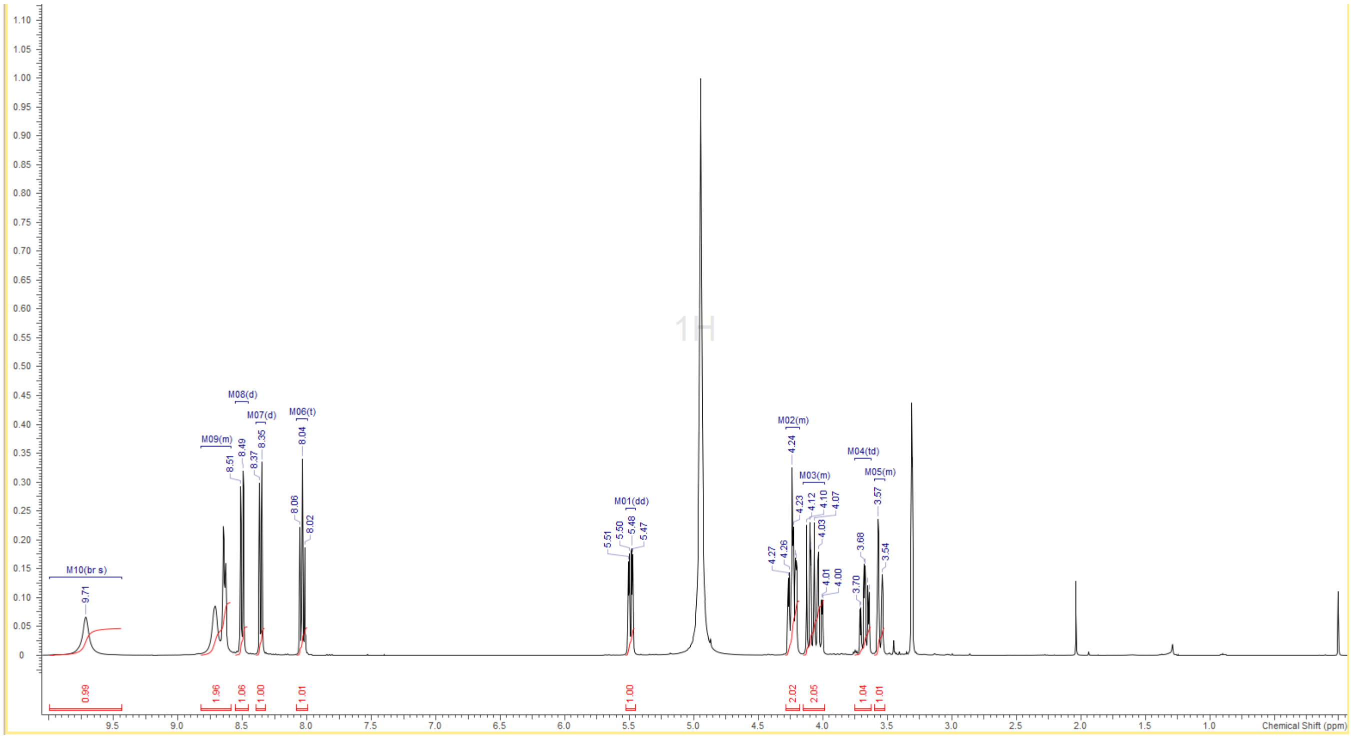

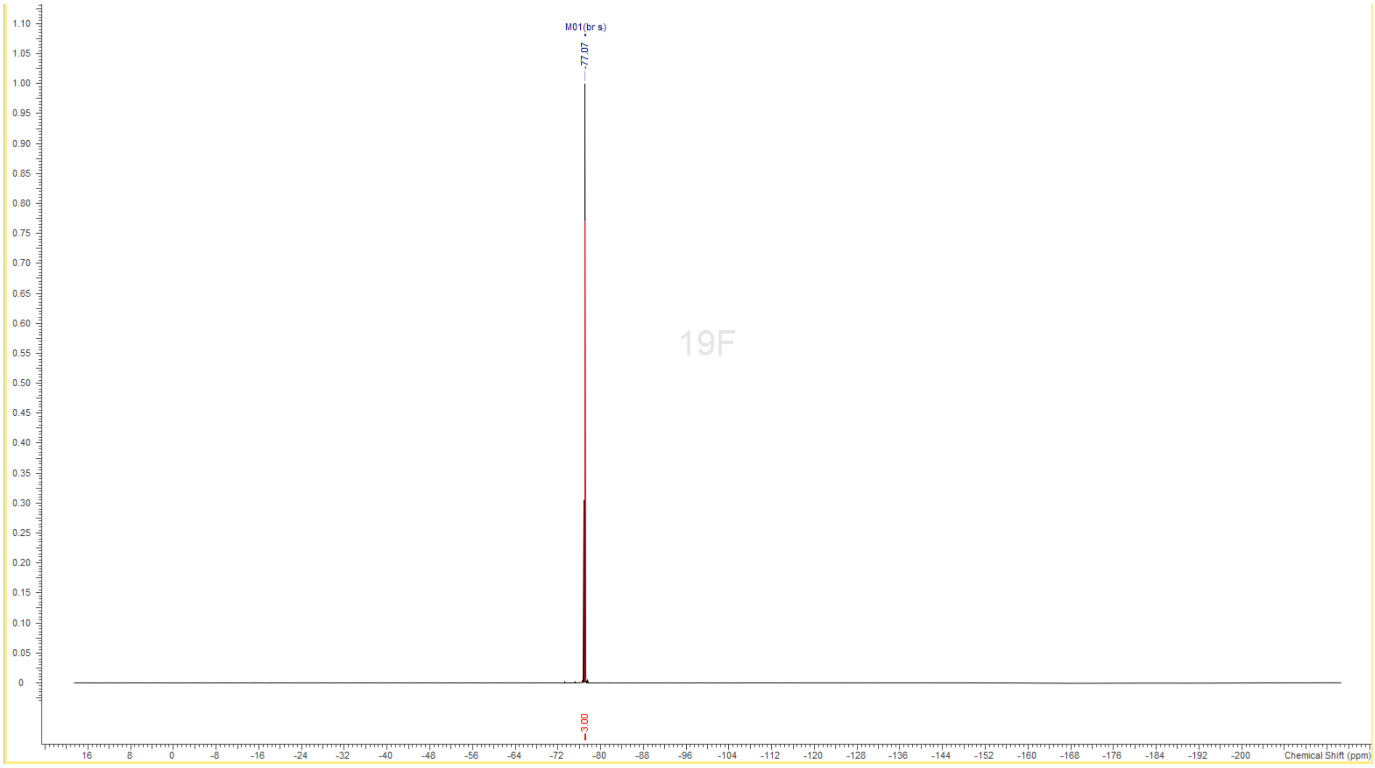
M01(br s)
-77.07
19F
-3.00
Chemical Shift (ppm)

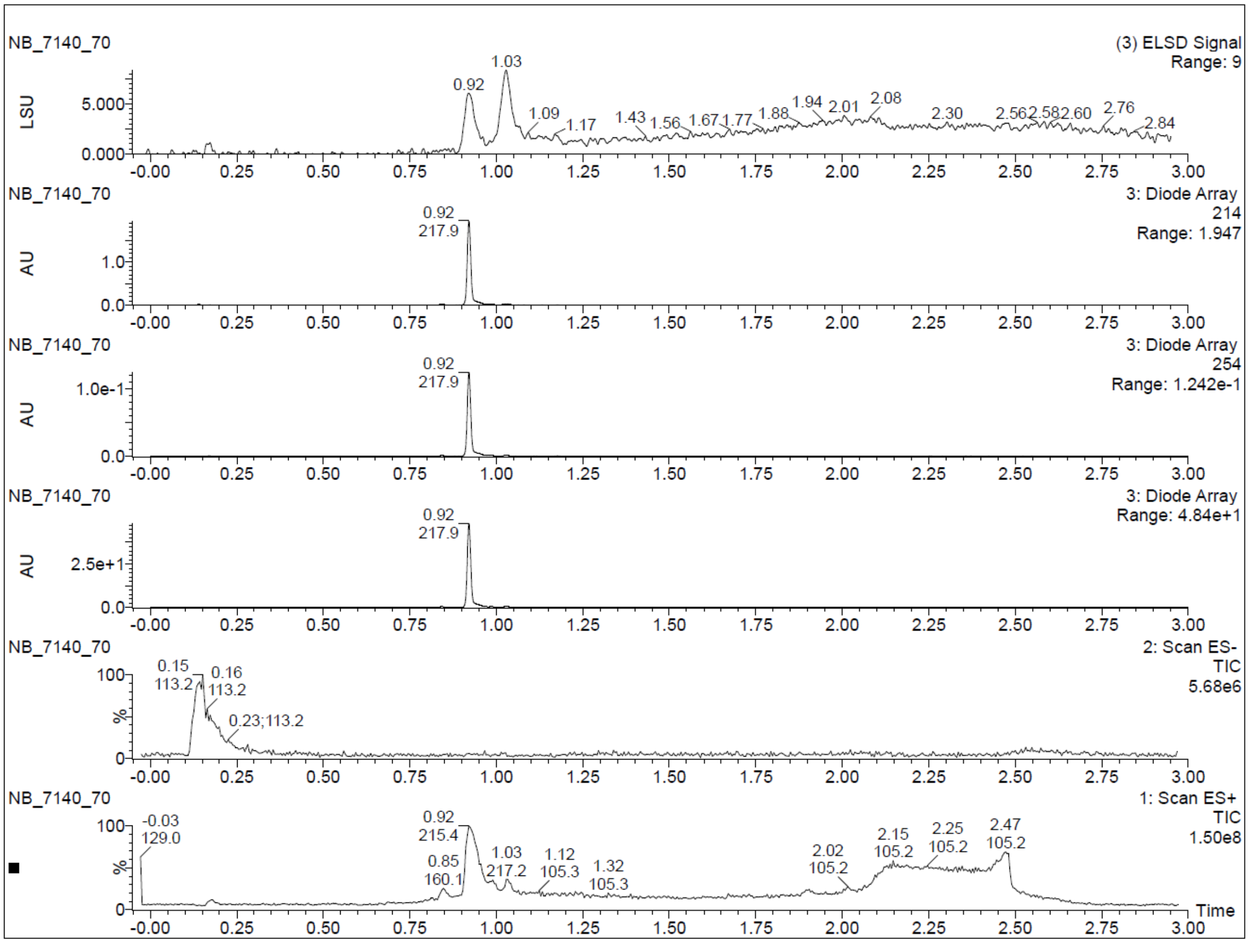
NB_7140_70
(3) ELSD Signal
Range: 9
LSU
3: Diode Array
214
Range: 1.947
AU
3: Diode Array
254
Range: 1.242e-1
3: Diode Array
Range: 4.84e+1
2: Scan ES-
TIC
5.68e6
1: Scan ES+
TIC
1.50e8
Time

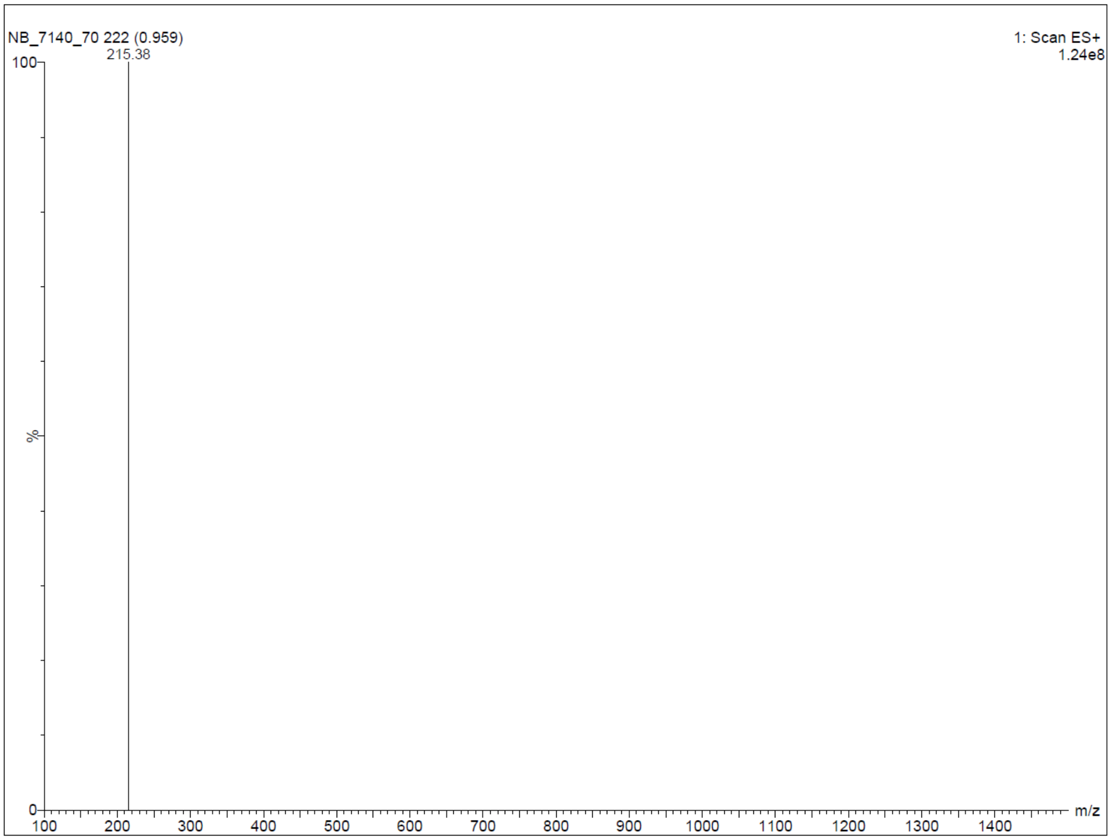

3-(1-Benzyl-imidazol-5-yl)morpholine, TFA Salt (4aq)

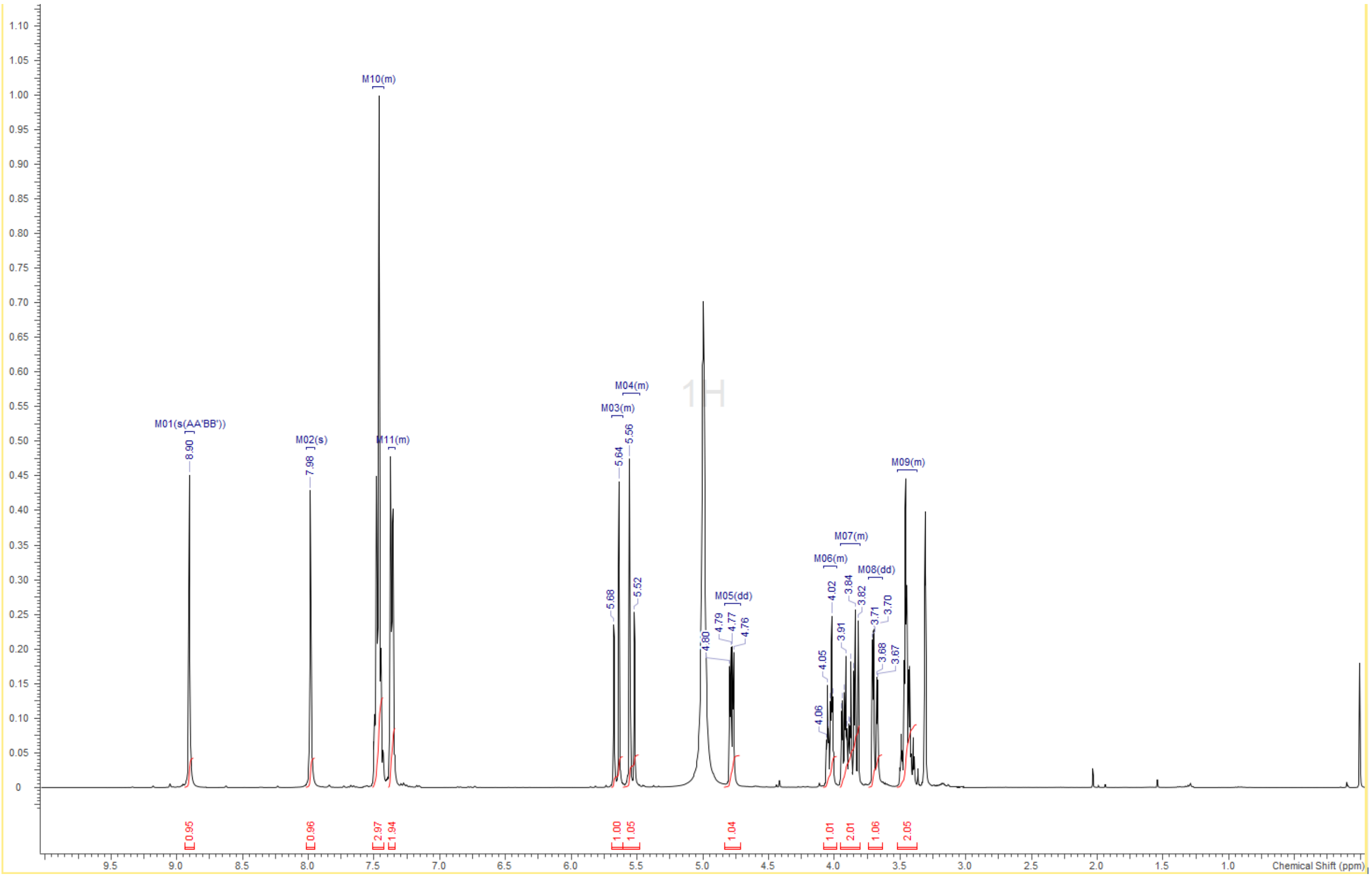

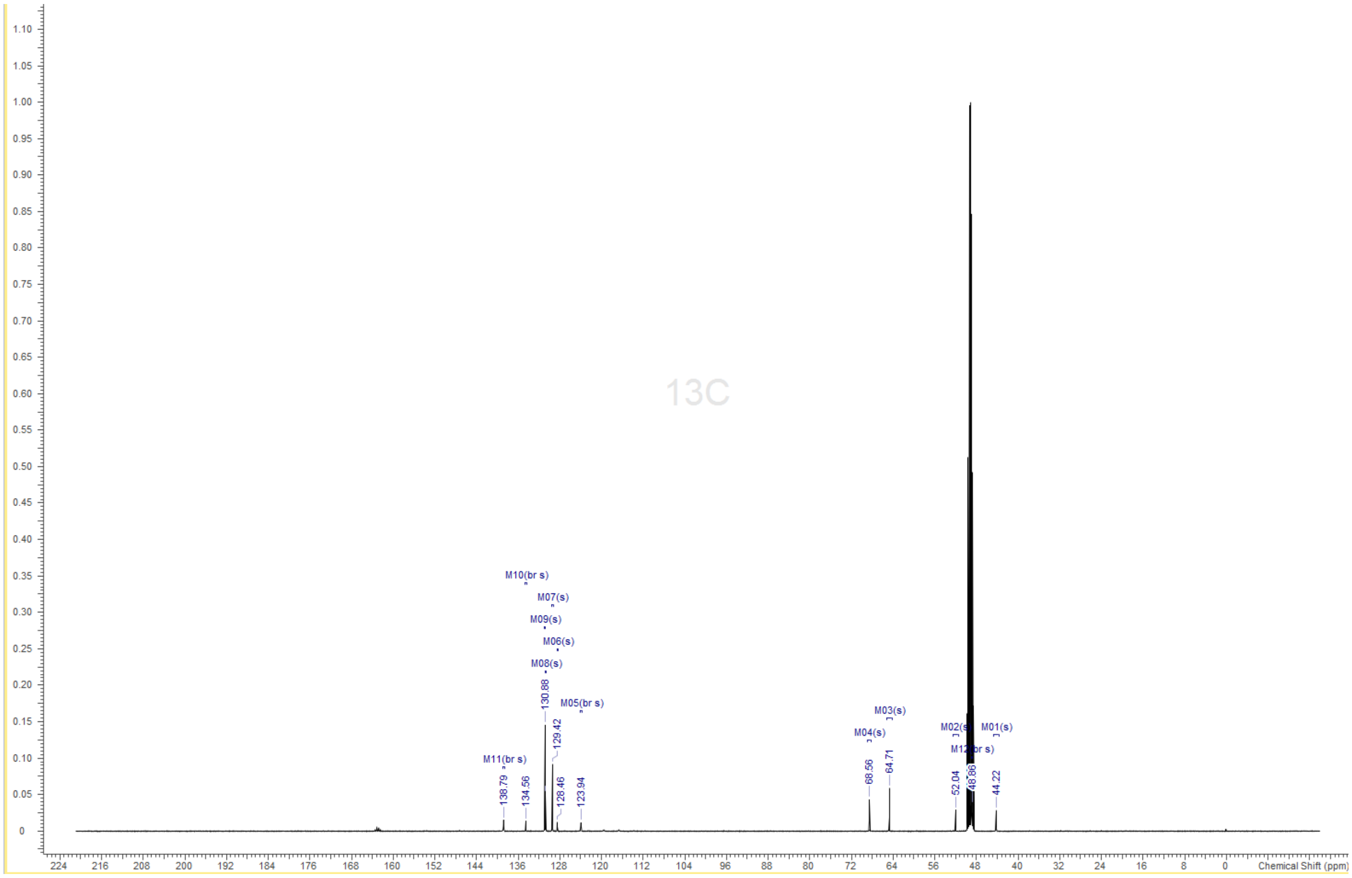
13C
M10(br s)
M07(s)
M09(s)
M06(s)
M08(s)
M05(br s)
M11(br s)
M03(s)
M04(s)
M02(s)
M01(s)
M12(br s)
138.79
134.56
130.88
129.42
128.46
123.94
68.56
64.71
52.04
48.86
44.22
Chemical Shift (ppm)

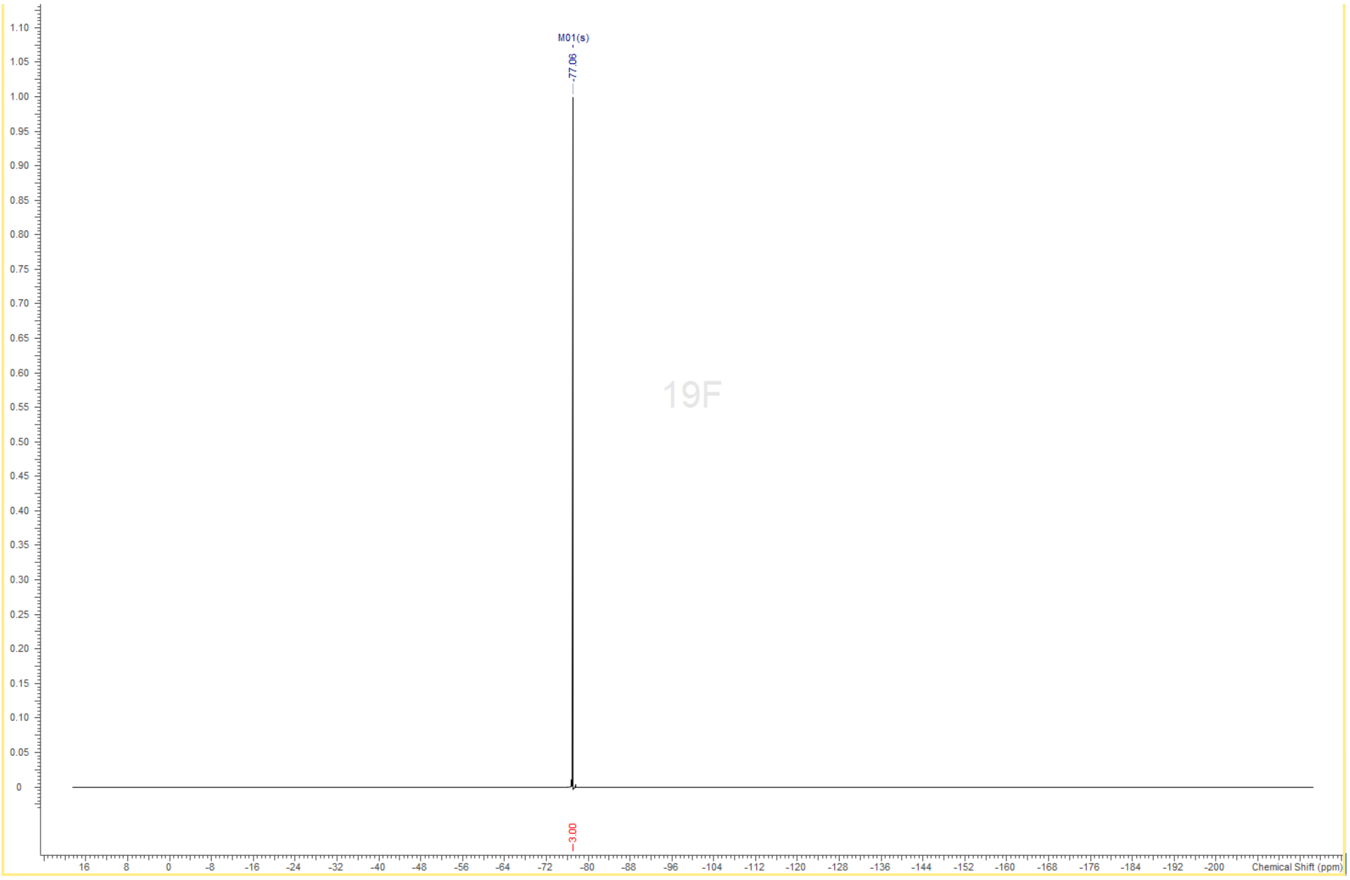
19F
M01(s)
-77.06
-3.00
Chemical Shift (ppm)

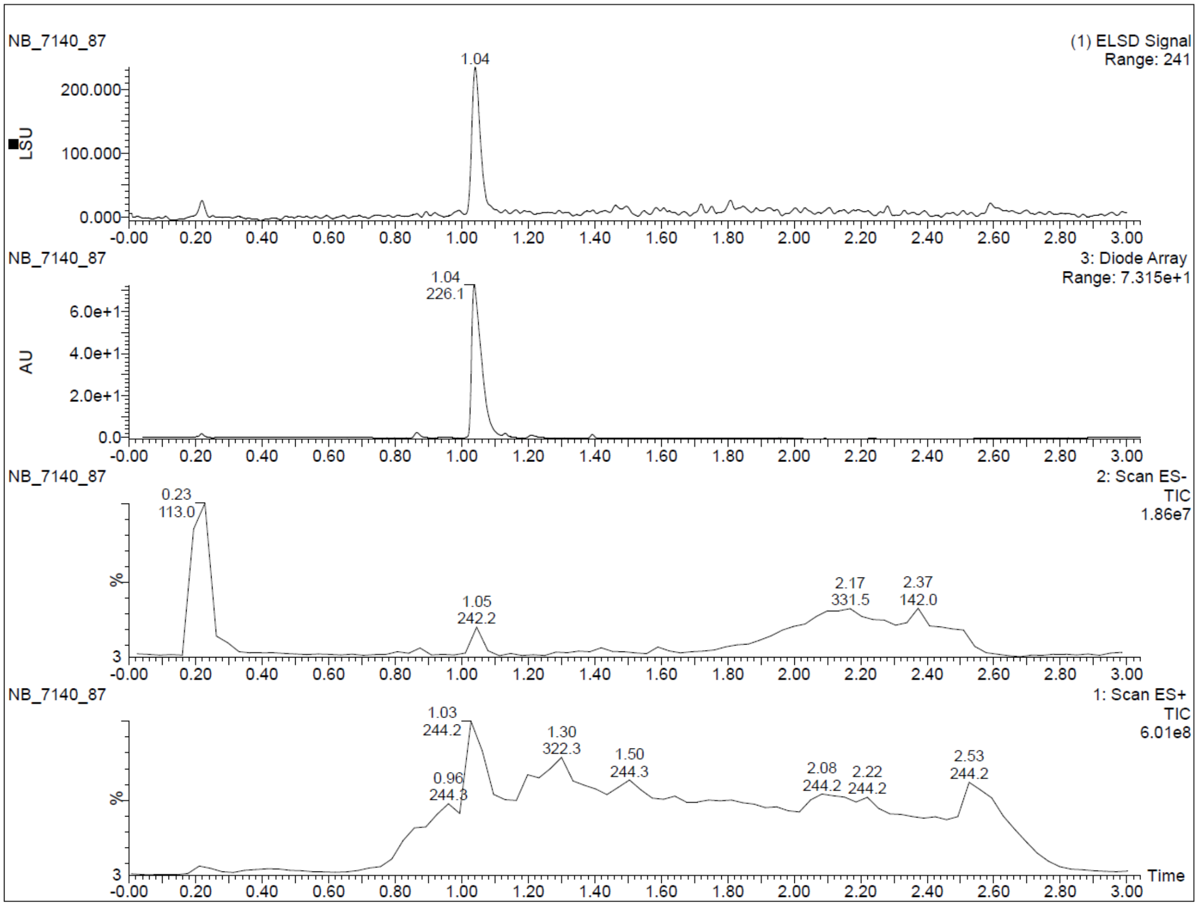
NB_7140_87
(1) ELSD Signal
Range: 241
1.04
ELSU
NB_7140_87
3: Diode Array
Range: 7.315e+1
1.04
226.1
AU
NB_7140_87
2: Scan ES-
TIC
1.86e7
0.23
113.0
1.05
242.2
2.17
331.5
2.37
142.0
NB_7140_87
1: Scan ES+
TIC
6.01e8
1.03
244.2
1.30
322.3
1.50
244.3
0.96
244.3
2.08
244.2
2.22
244.2
2.53
244.2
Time

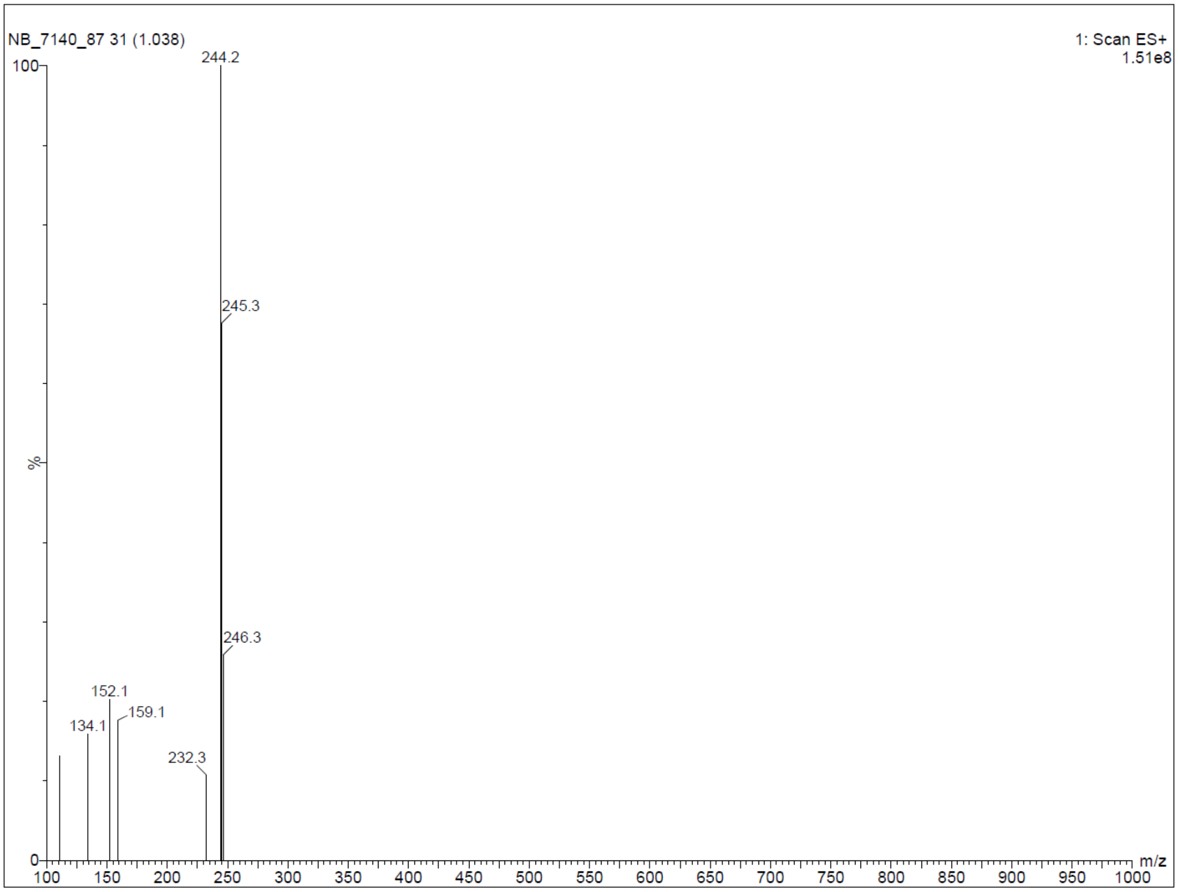
NB_7140_87 31 (1.038)
1: Scan ES+
1.51e8
244.2
245.3
246.3
152.1
159.1
134.1
232.3
%
m/z

3-(Naphthalen-2-yl)morpholine, Free base (4ai)

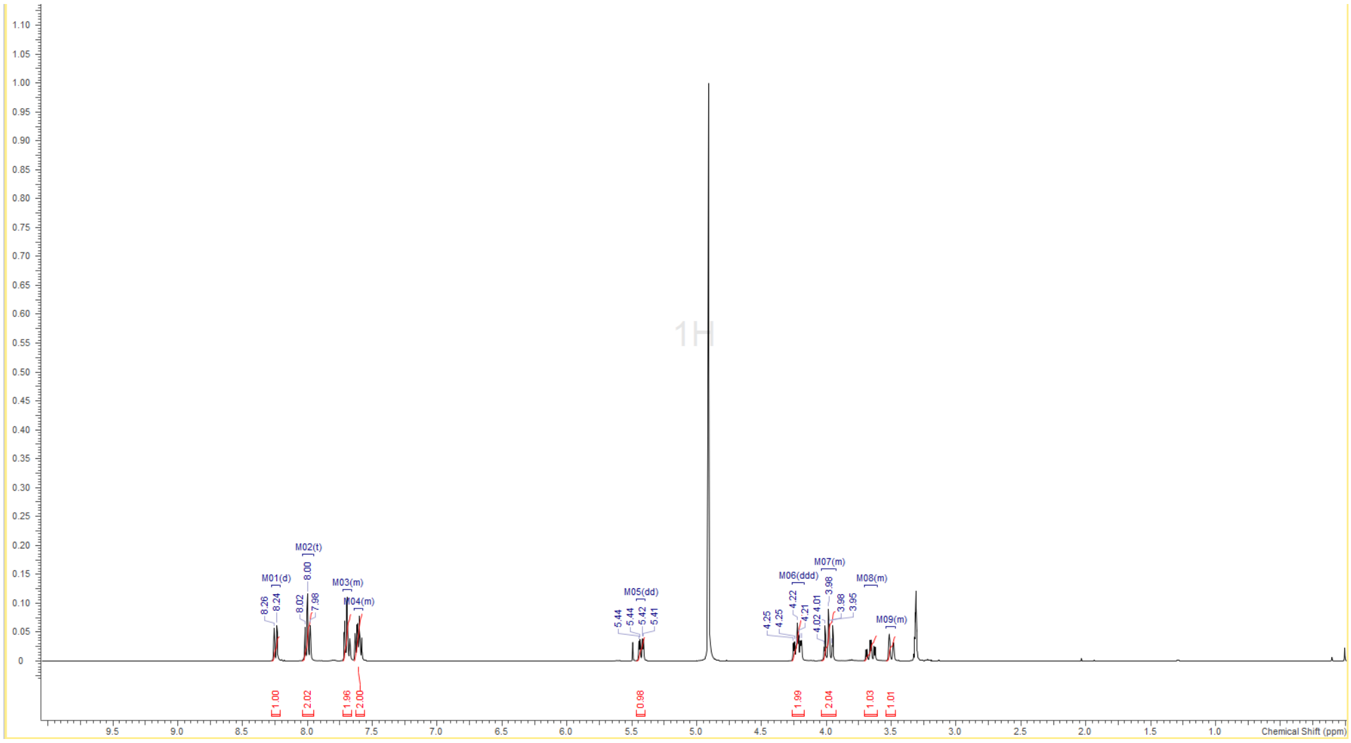

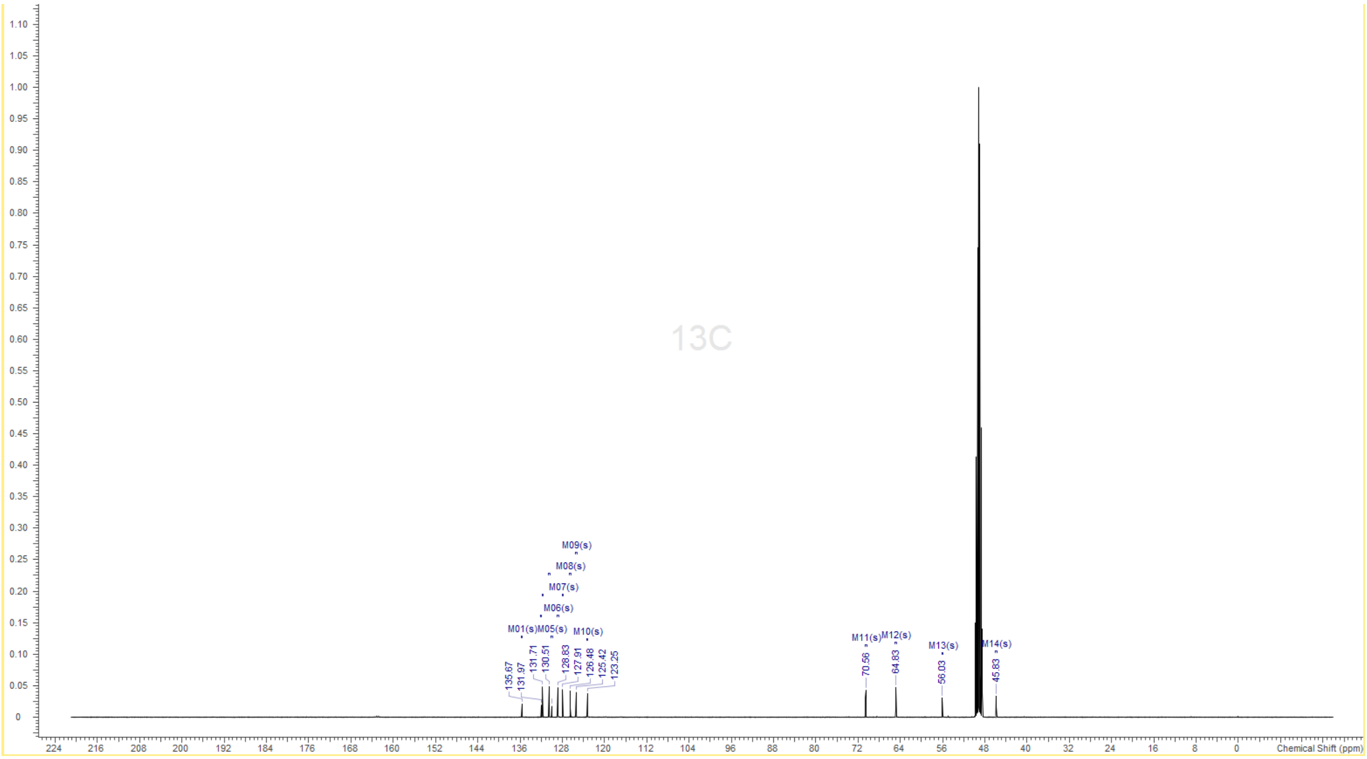

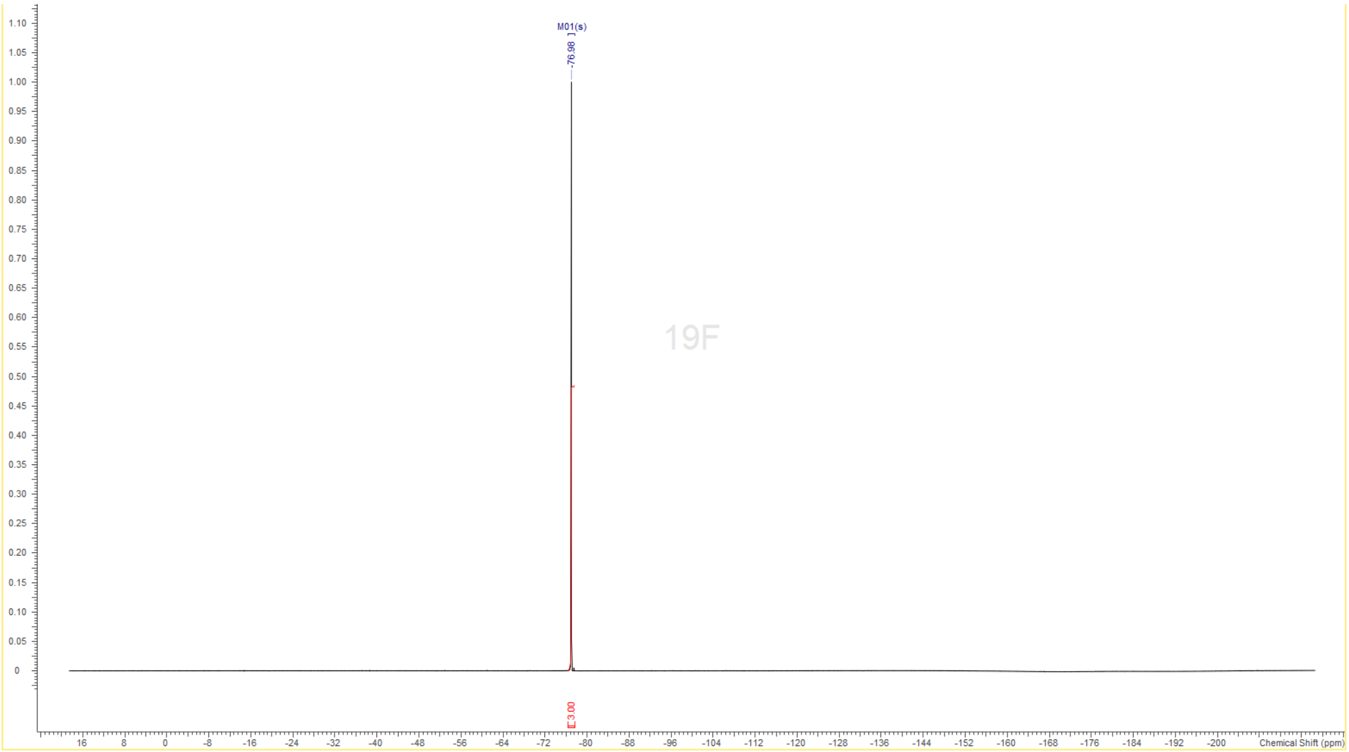

M01(s)
-76.98
19F
3.00
Chemical Shift (ppm)

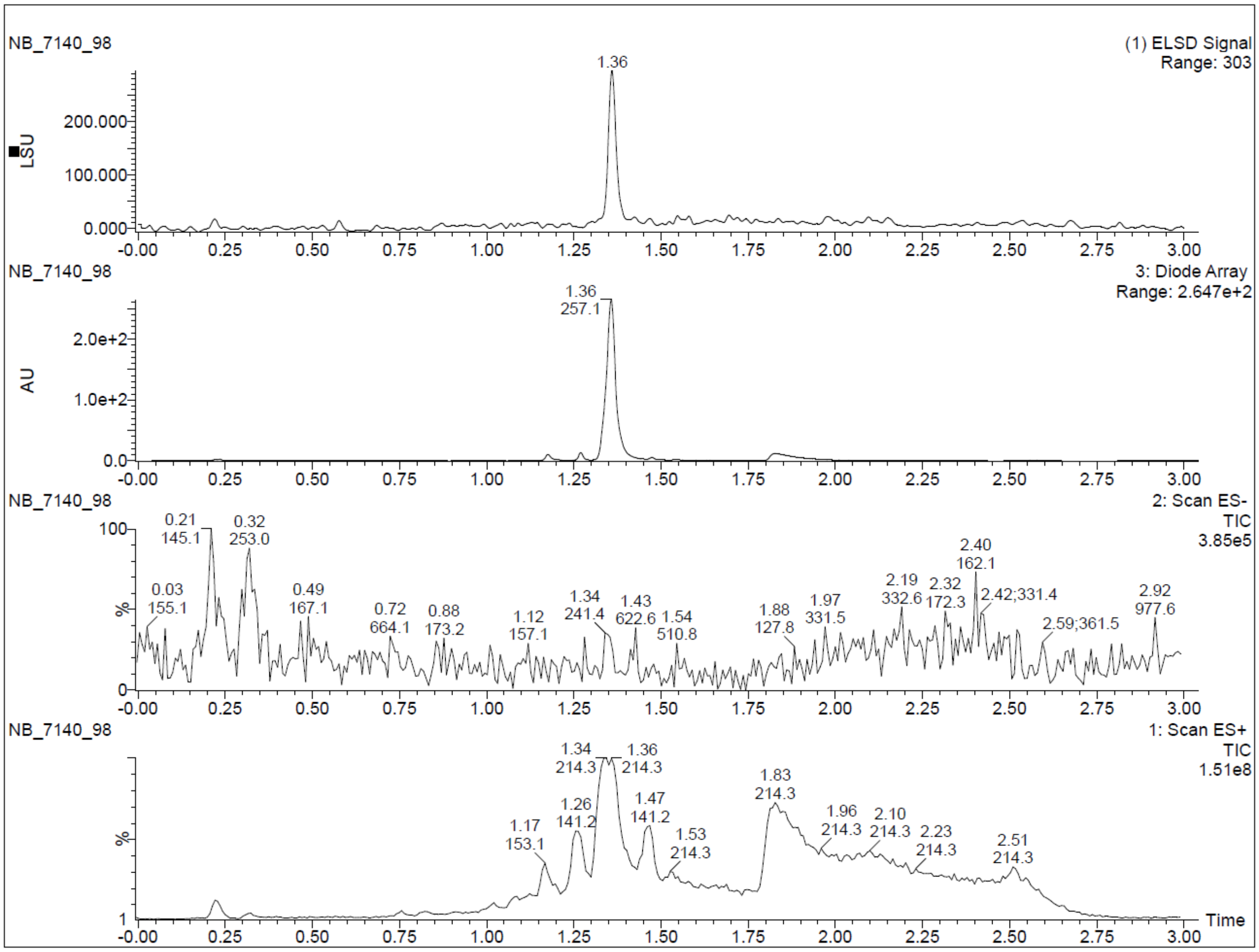

NB_7140_98
(1) ELSD Signal
Range: 303
LSU
NB_7140_98
3: Diode Array
Range: 2.647e+2
AU
NB_7140_98
2: Scan ES-
TIC
3.85e5
NB_7140_98
1: Scan ES+
TIC
1.51e8
Time

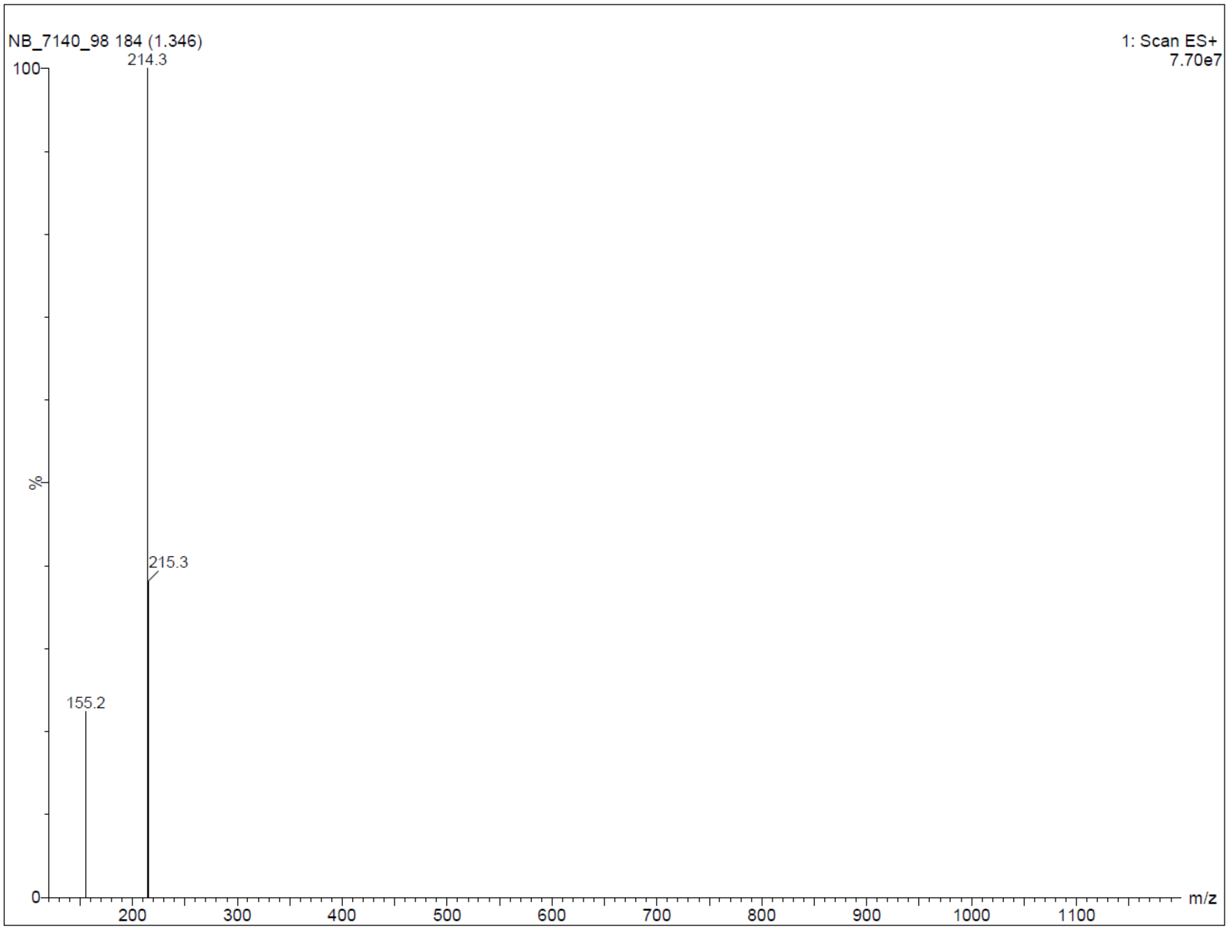

***tert*-Butyl 4-(morpholin-3-yl)indoline-1-carboxylate, Free base (4al)**

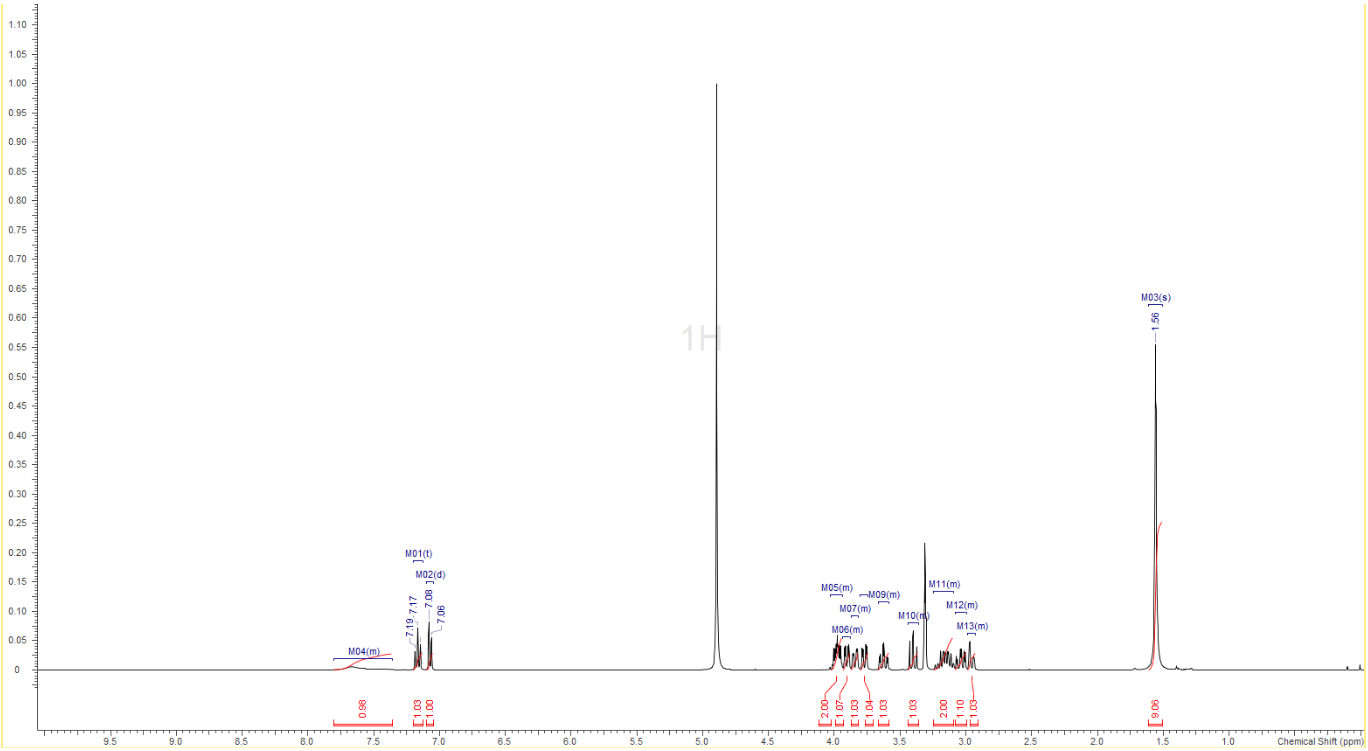

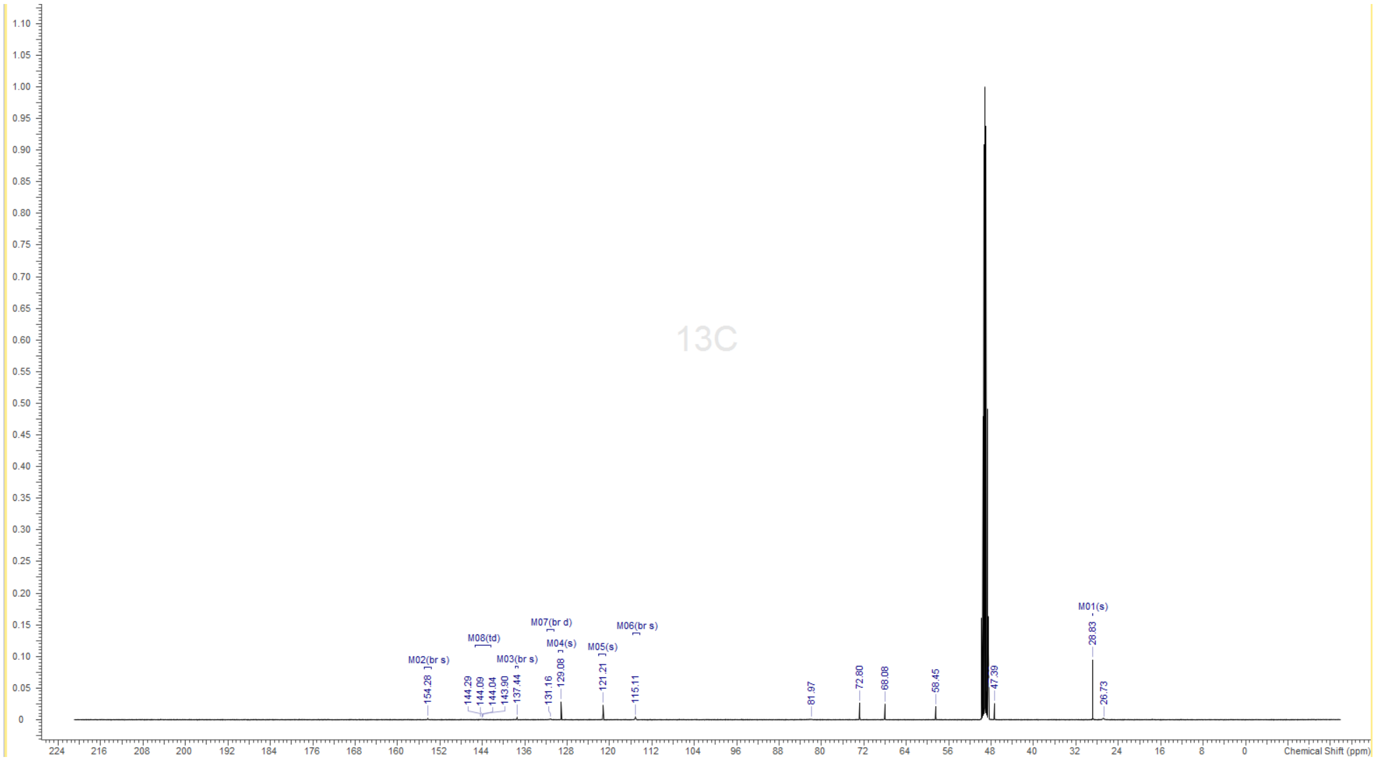

Zoomed Carbon:

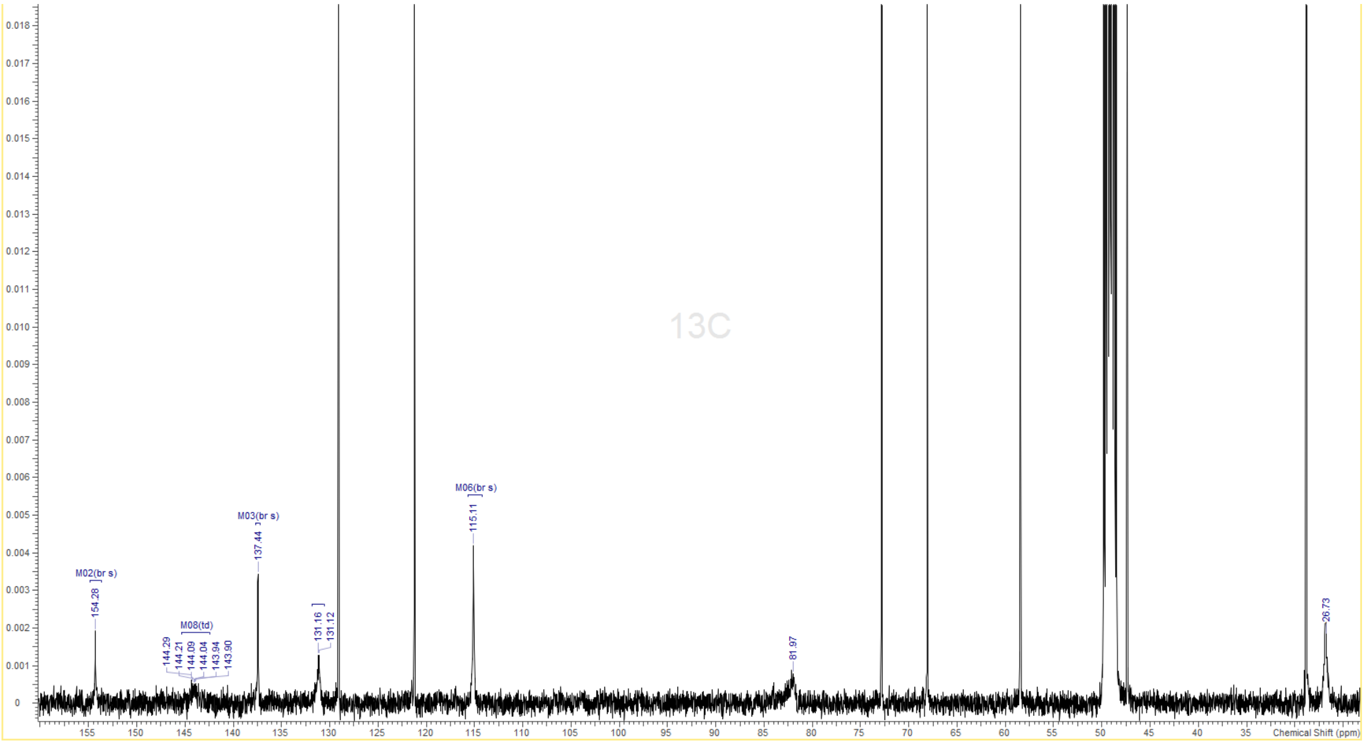

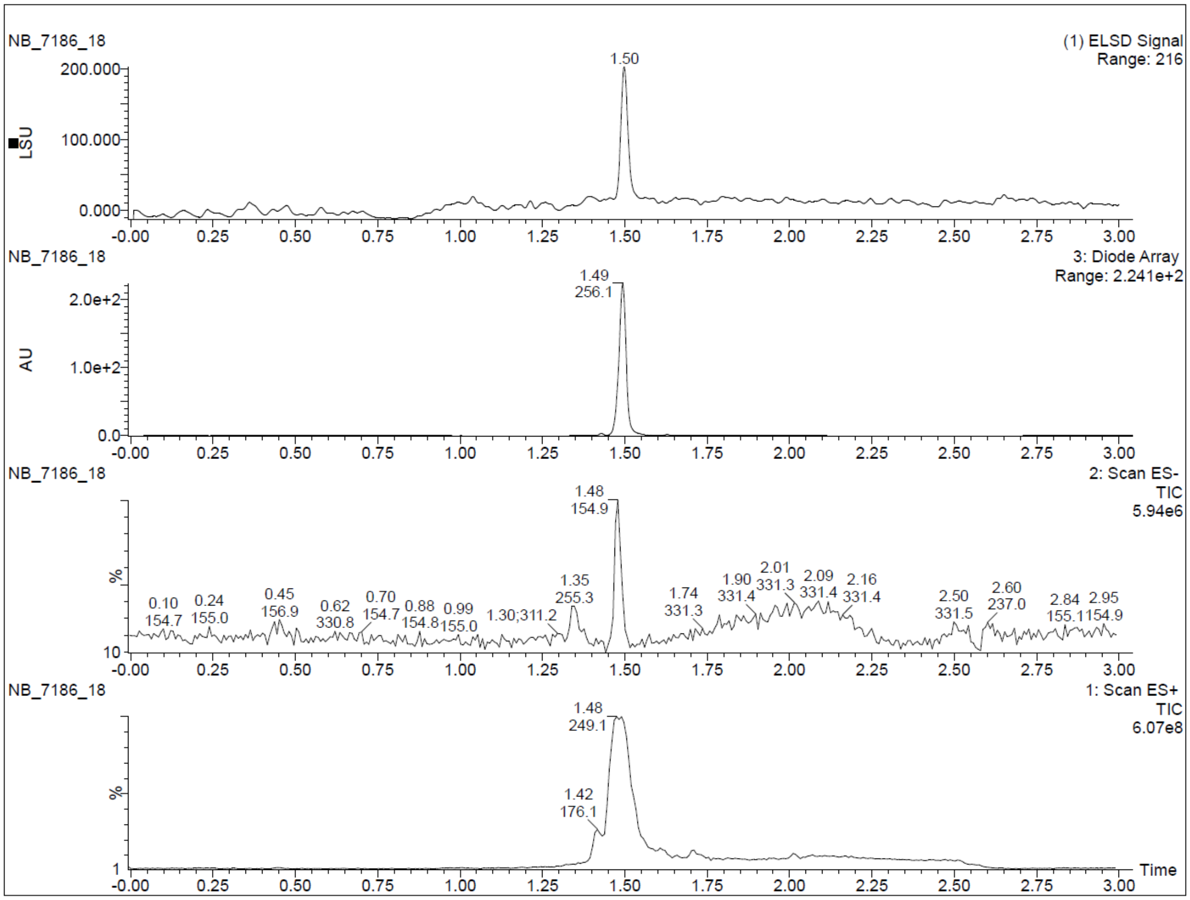
NB_7186_18
(1) ELSD Signal
Range: 216
LSU
3: Diode Array
Range: 2.241e+2
AU
2: Scan ES-
TIC
5.94e6
1: Scan ES+
TIC
6.07e8
Time

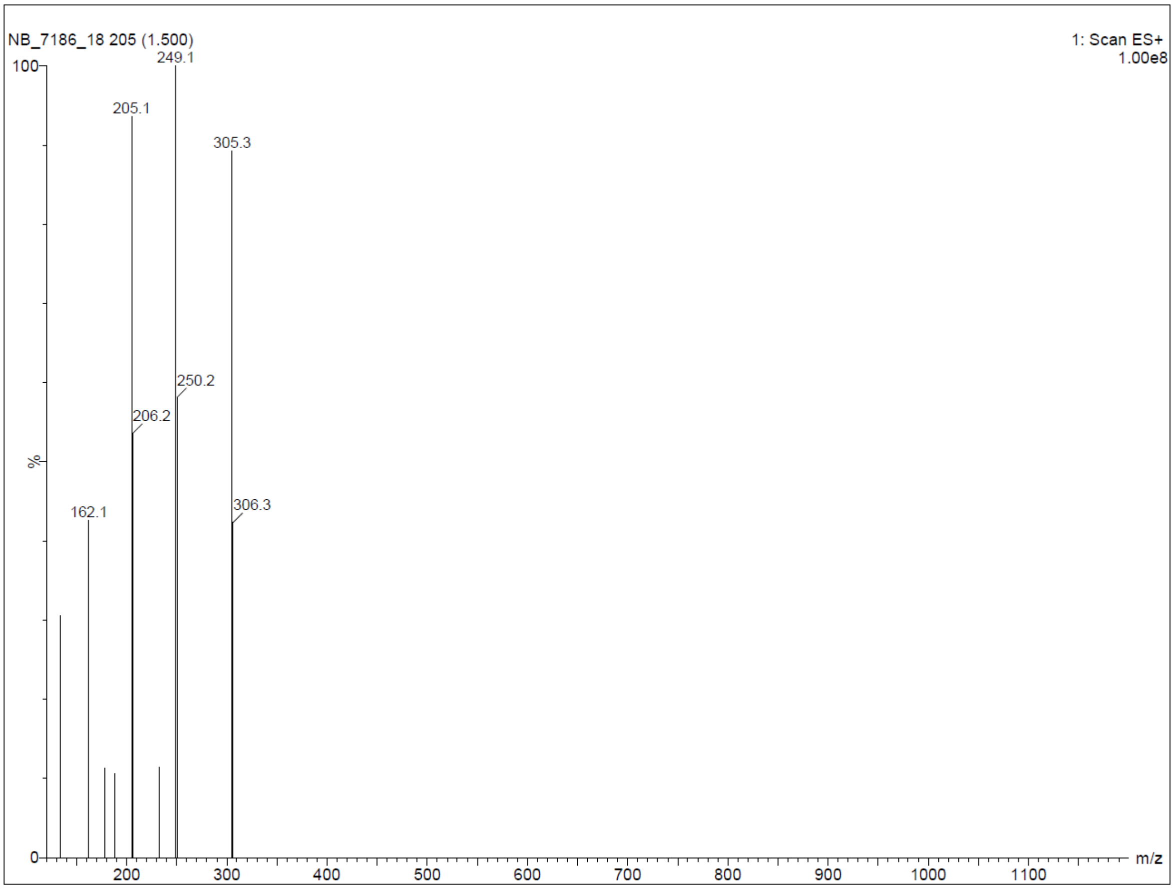
NB_7186_18 205 (1.500)
1: Scan ES+
1.00e8
249.1
205.1
305.3
250.2
206.2
306.3
162.1
m/z

3-(6-Phenyl-2,3-dihydroimidazo[2,1-b]thiazol-5-yl)morpholine, Free base (4aw)

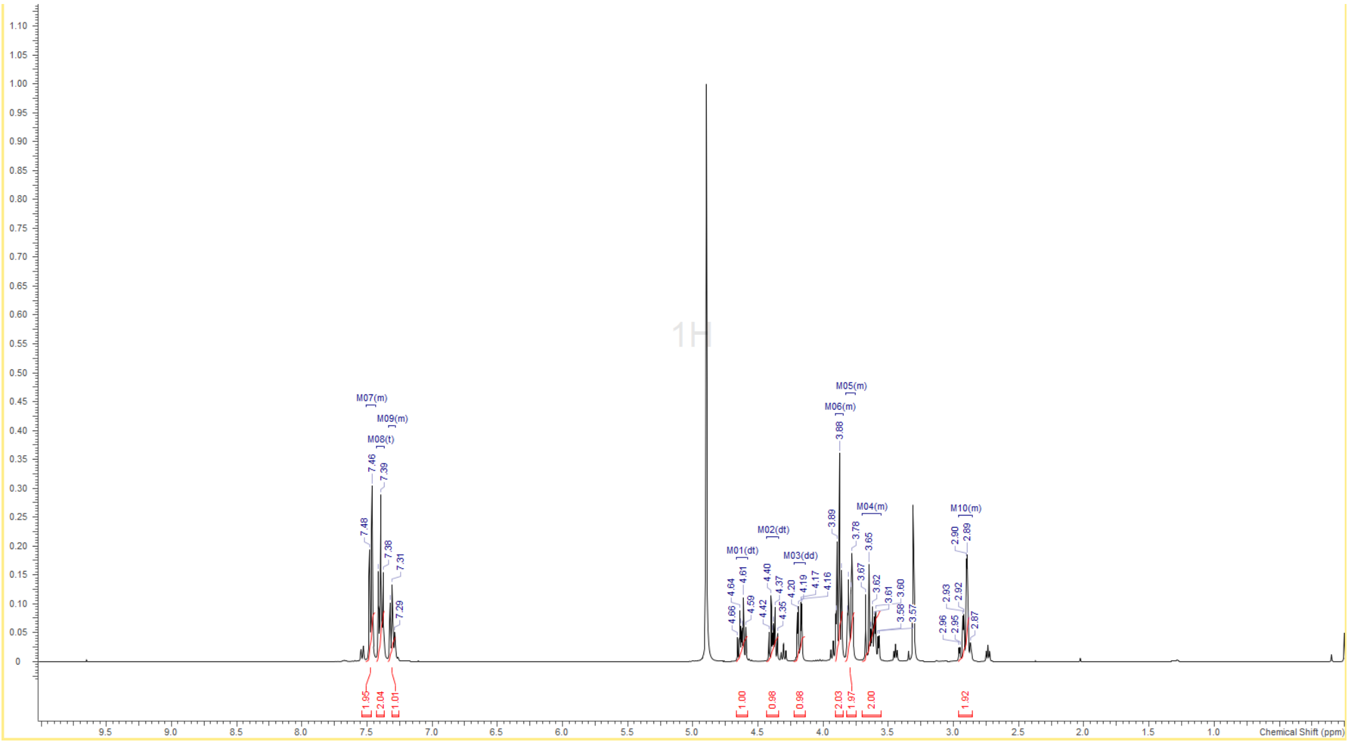

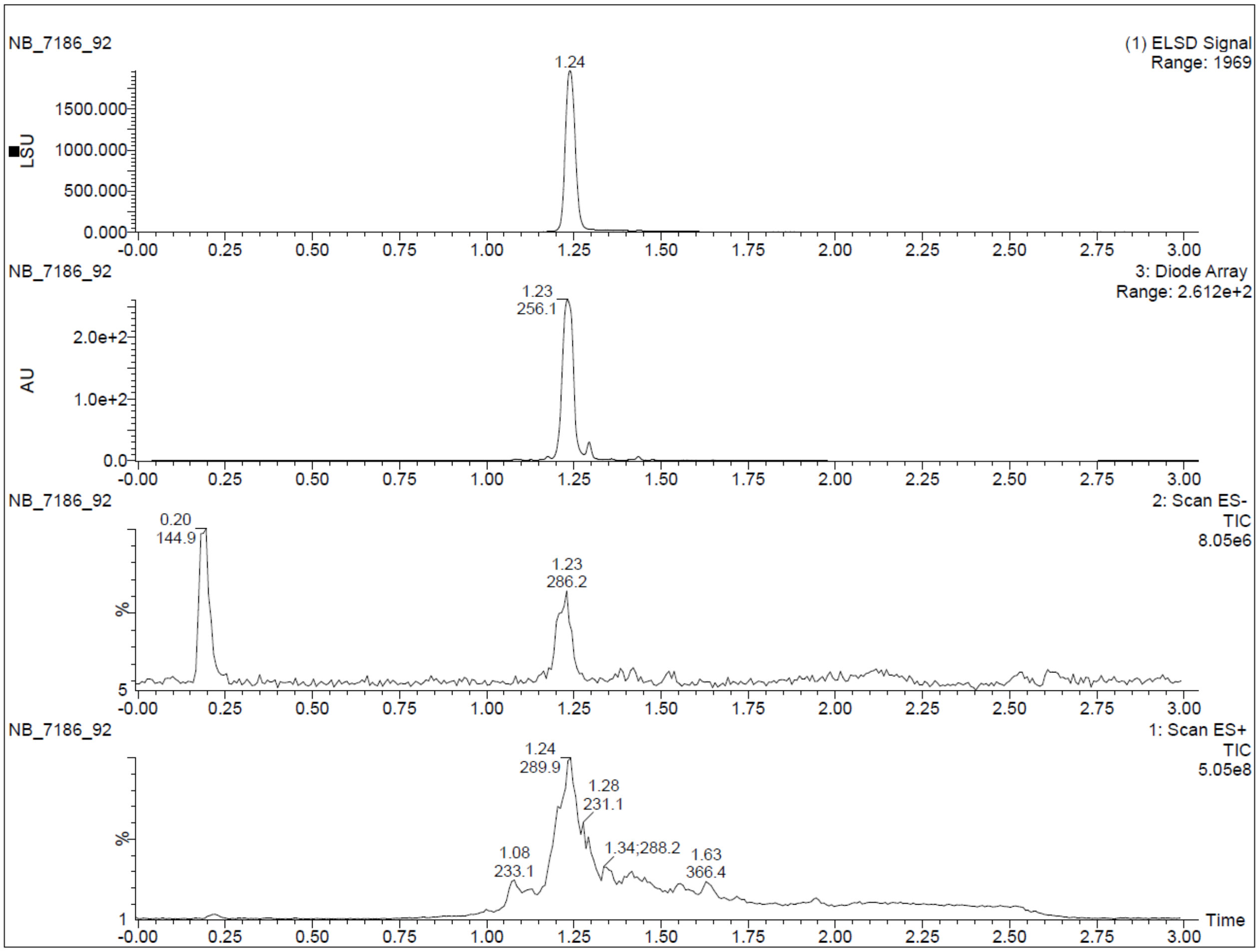

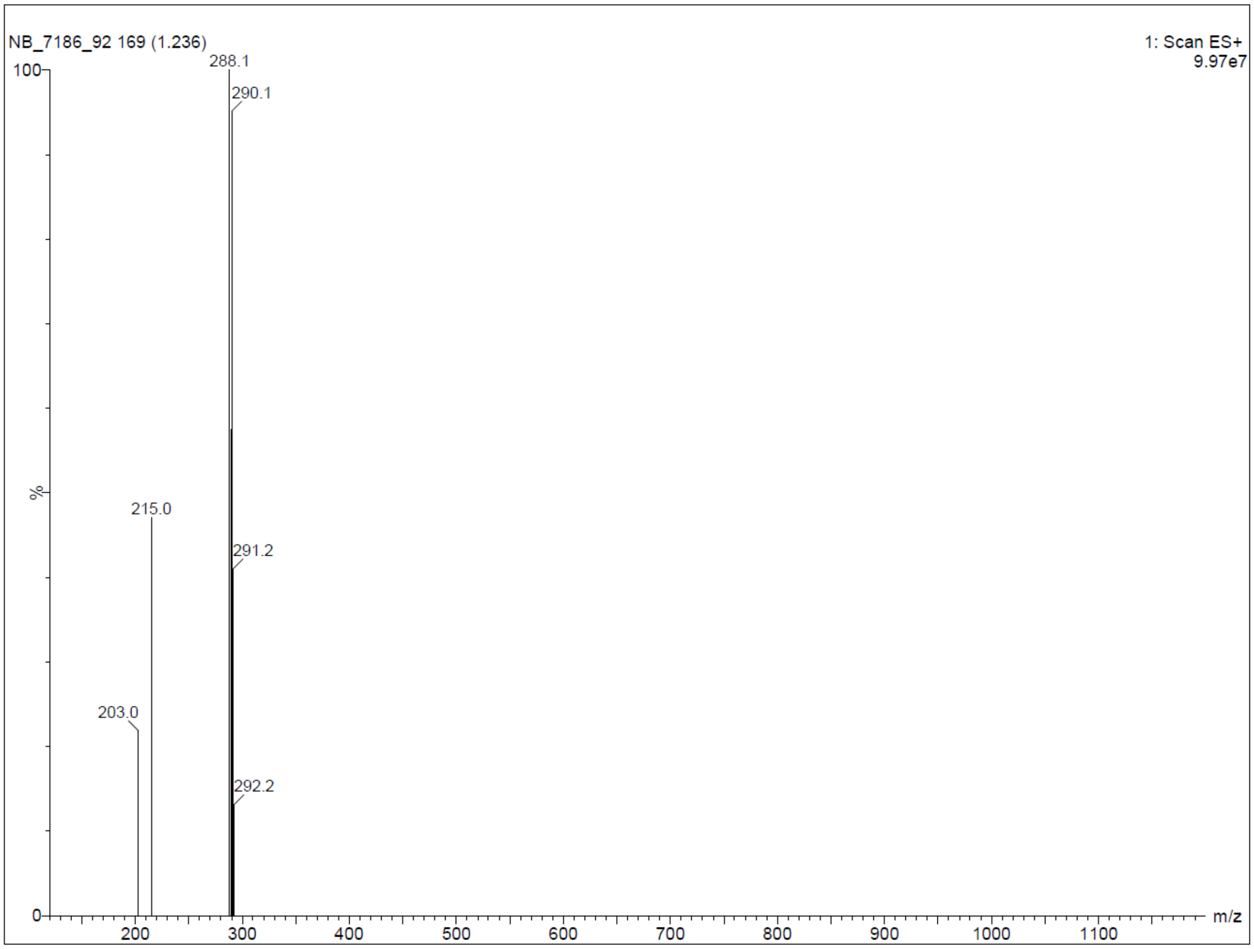

3-(3-Phenylisoxazol-5-yl)morpholine, Free base (4ax)

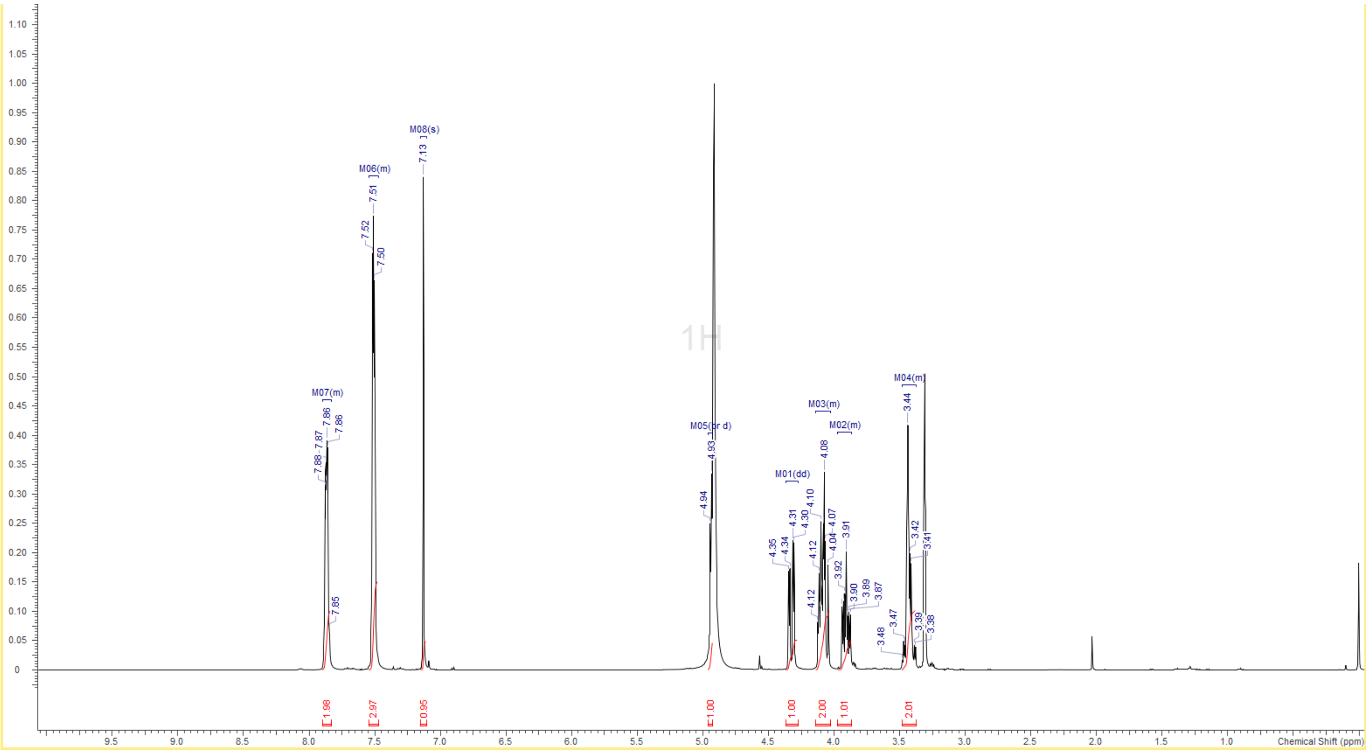

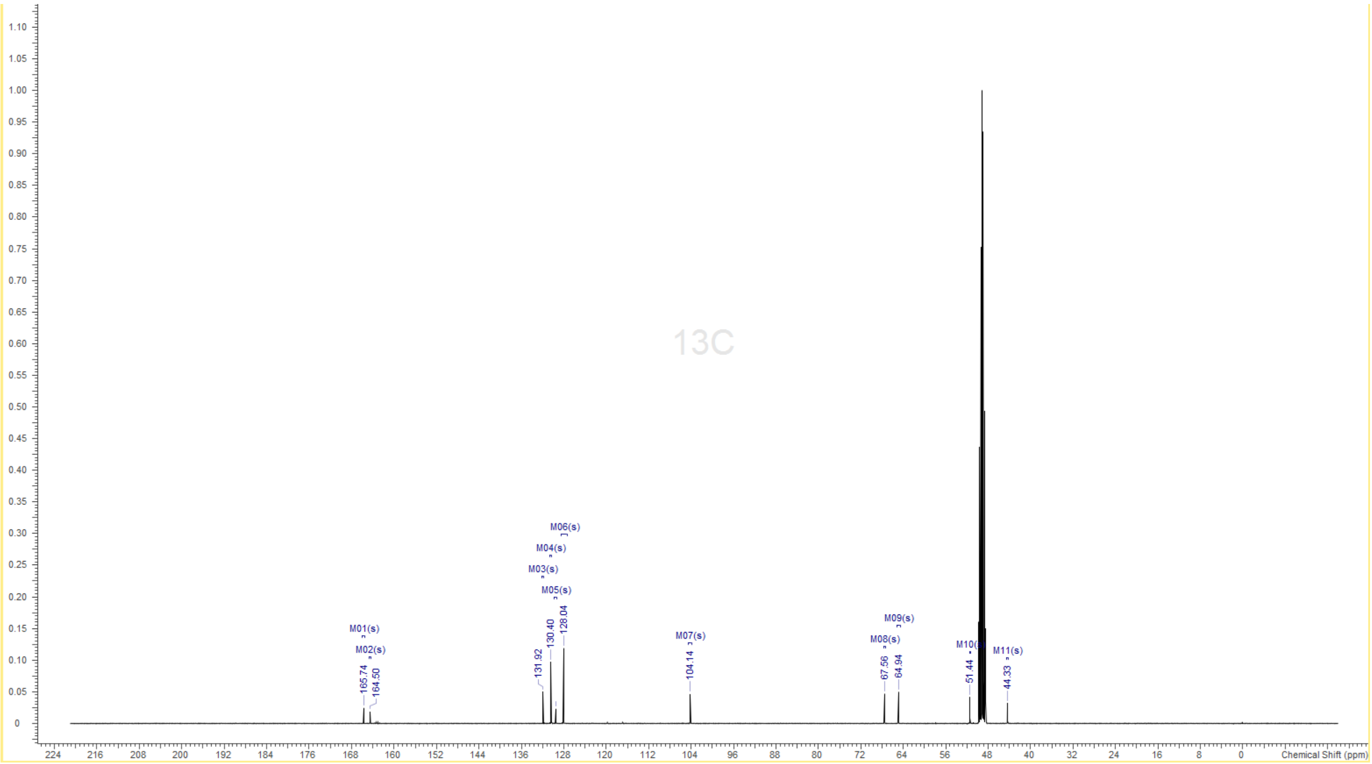
13C
M01(s)
M02(s)
M03(s)
M04(s)
M05(s)
M06(s)
M07(s)
M08(s)
M09(s)
M11(s)
165.74
164.50
131.92
130.40
128.04
104.14
67.56
64.94
51.44
44.33
Chemical Shift (ppm)

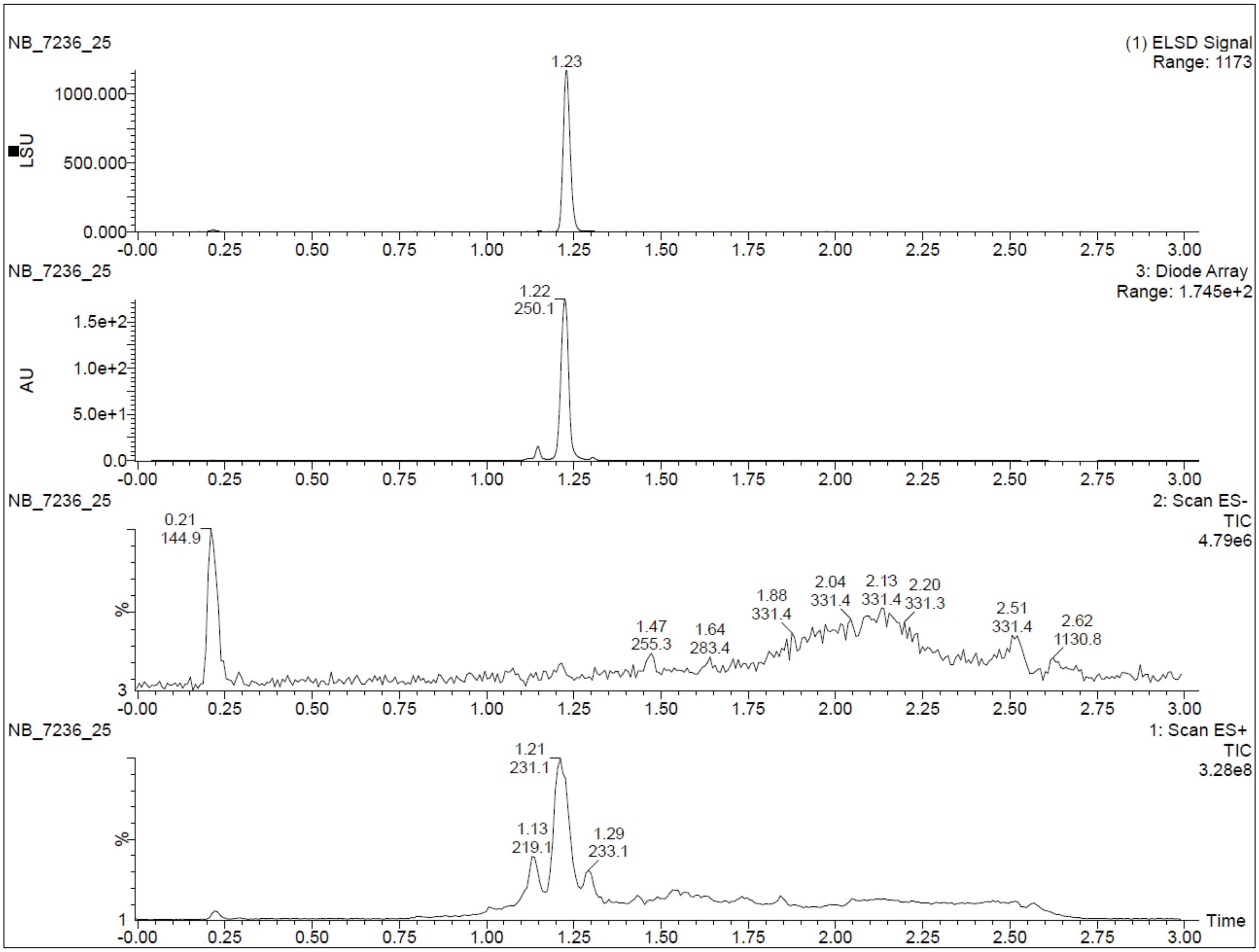
NB_7236_25
(1) ELSD Signal
Range: 1173
LSU
1.23
NB_7236_25
3: Diode Array
Range: 1.745e+2
AU
1.22
250.1
NB_7236_25
2: Scan ES-
TIC
4.79e6
0.21
144.9
1.47
255.3
1.64
283.4
1.88
331.4
2.04
331.4
2.13
331.4
2.20
331.3
2.51
331.4
2.62
1130.8
NB_7236_25
1: Scan ES+
TIC
3.28e8
1.21
231.1
1.13
219.1
1.29
233.1
Time

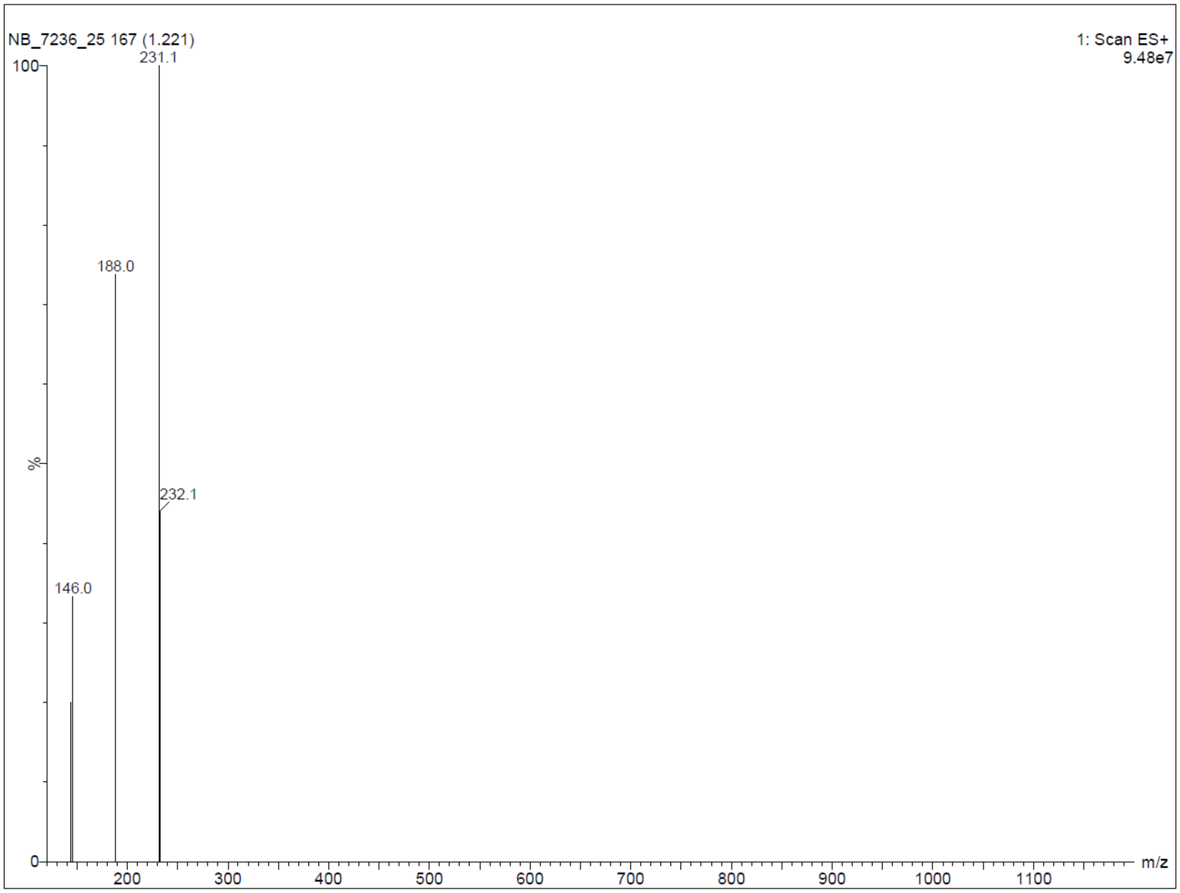

3-(Pyrazolo[1,5-a]pyridin-7-yl)morpholine, Free base (4ay)

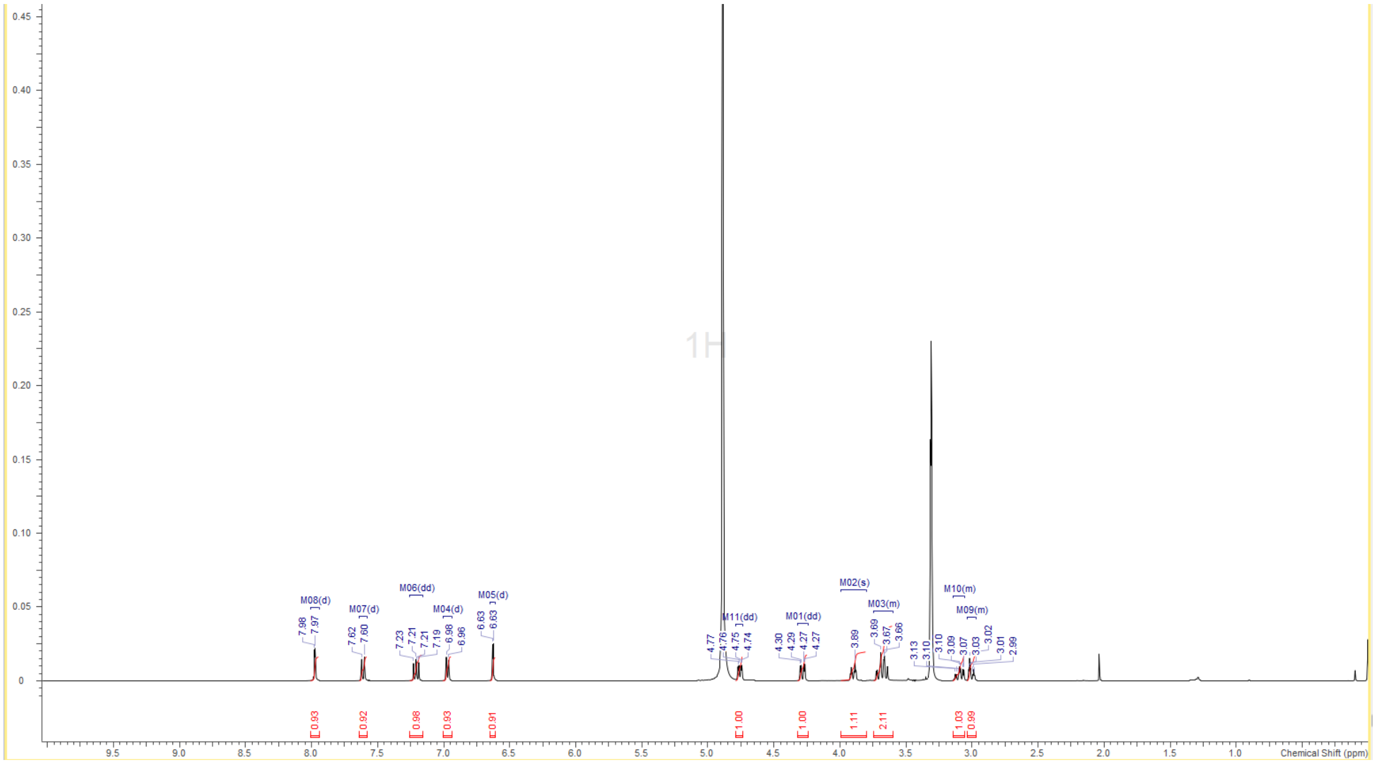

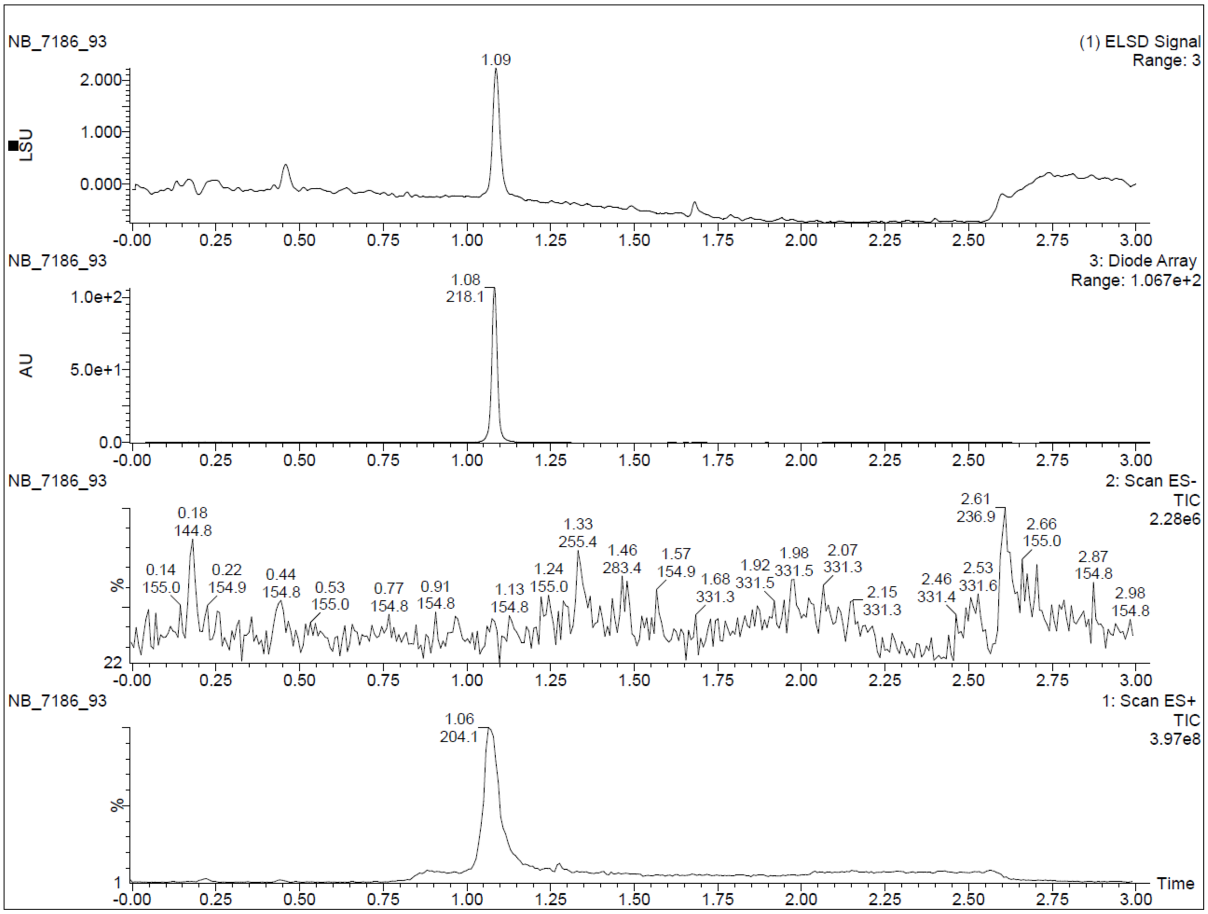
NB_7186_93
(1) ELSD Signal
Range: 3
1.09
LSU
NB_7186_93
3: Diode Array
Range: 1.067e+2
1.08
218.1
AU
NB_7186_93
2: Scan ES-
TIC
2.28e6
NB_7186_93
1: Scan ES+
TIC
3.97e8
1.06
204.1
Time

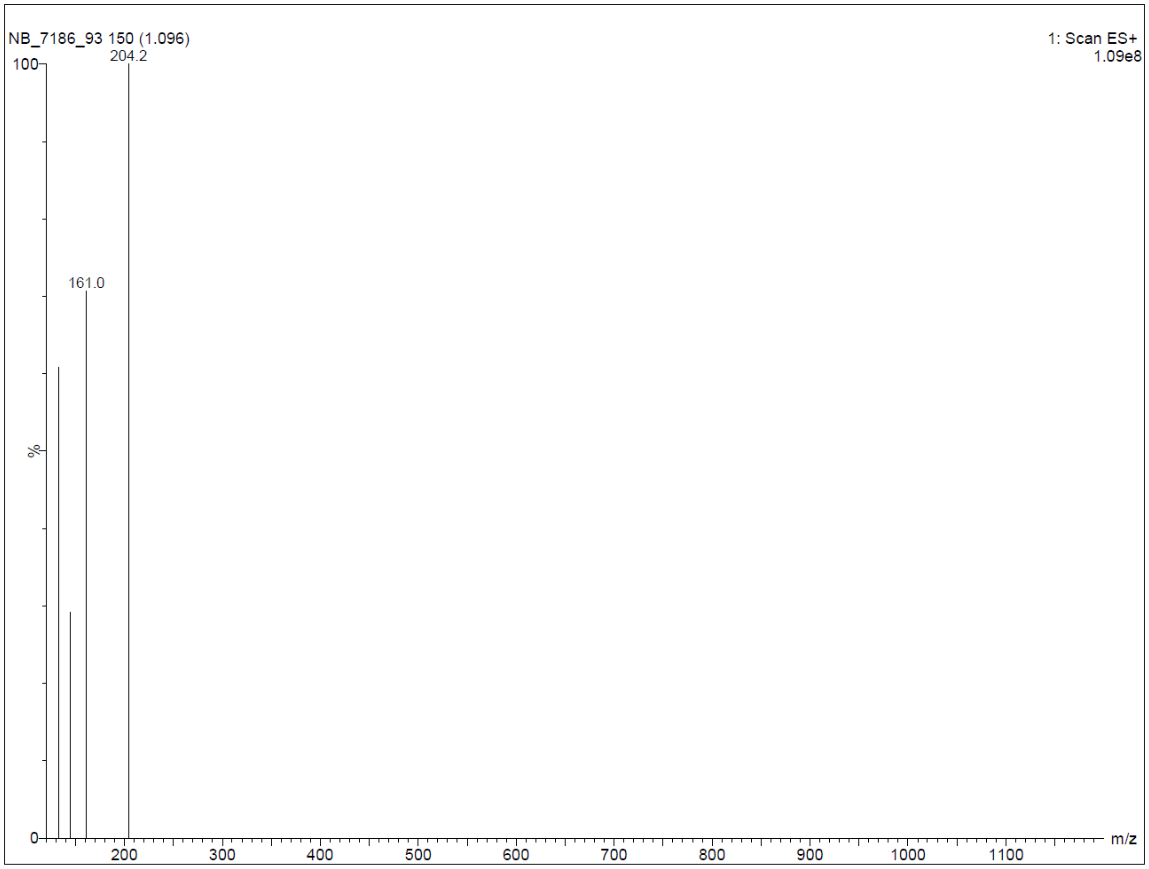
NB_7186_93 150 (1.096)
1: Scan ES+
1.09e8
204.2
161.0
m/z

7-(4-Methoxyphenyl)-5-oxa-8-azaspiro[3.5]nonane, TFA Salt (4ba)

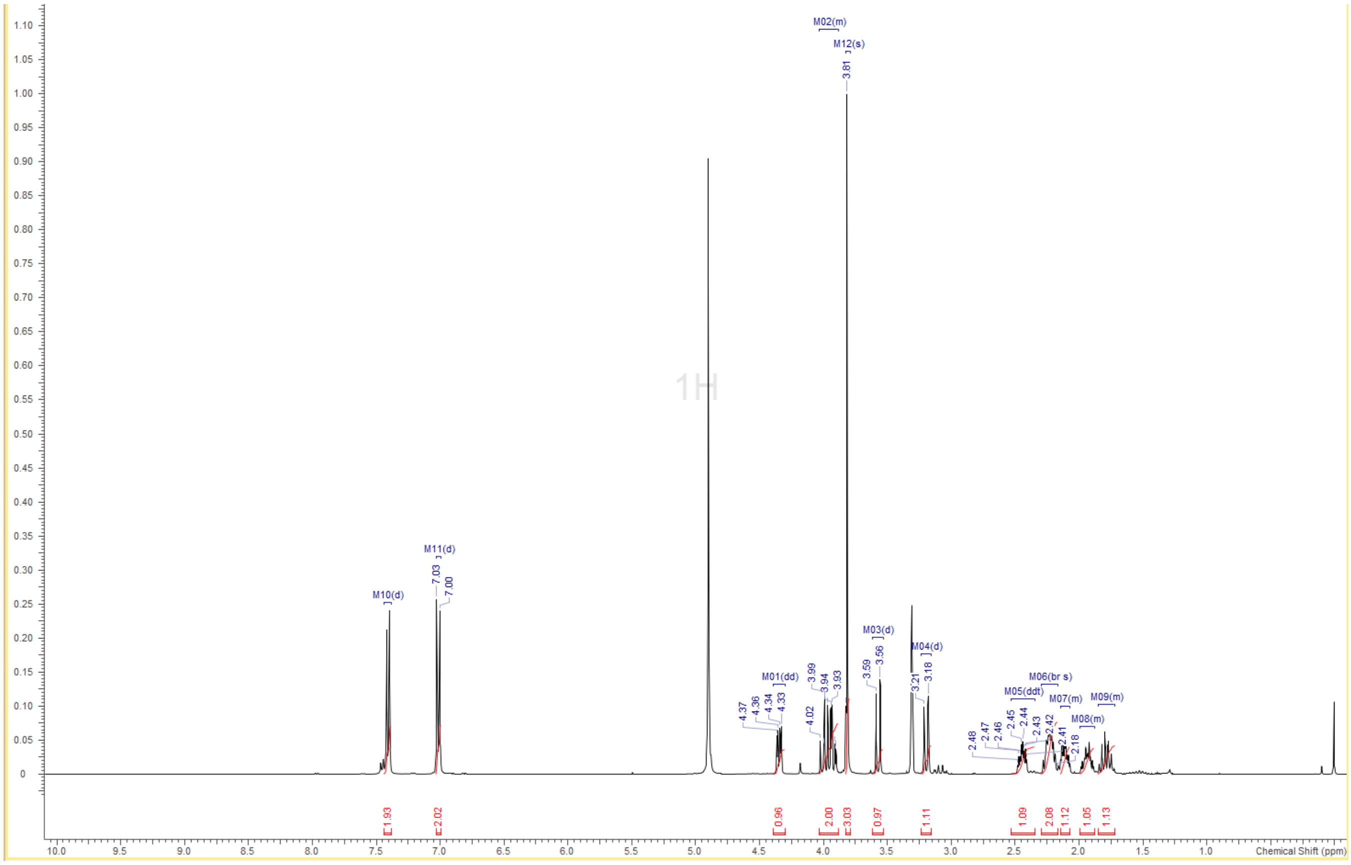

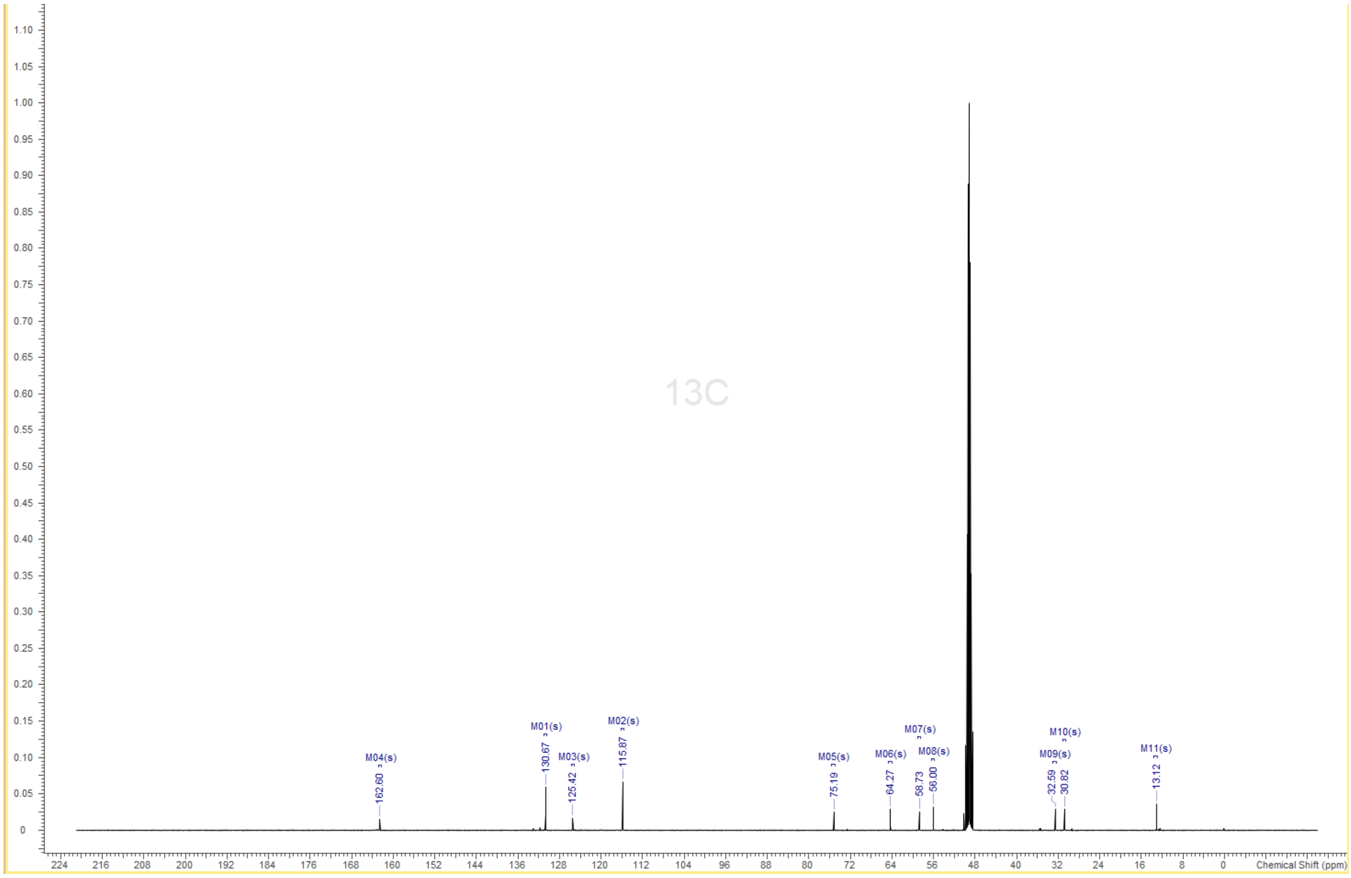

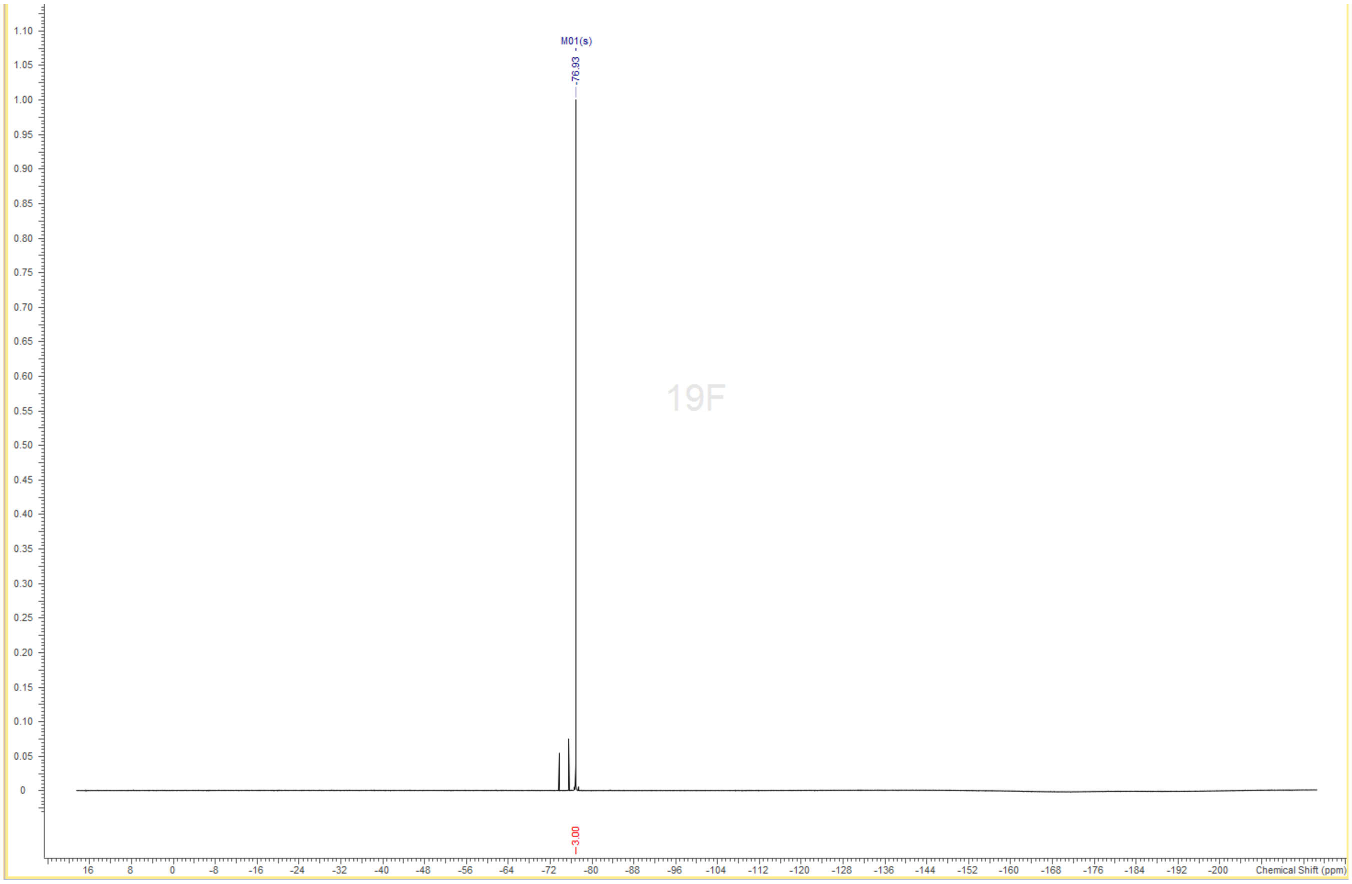
M01(s)
-76.93
19F
3.00
Chemical Shift (ppm)

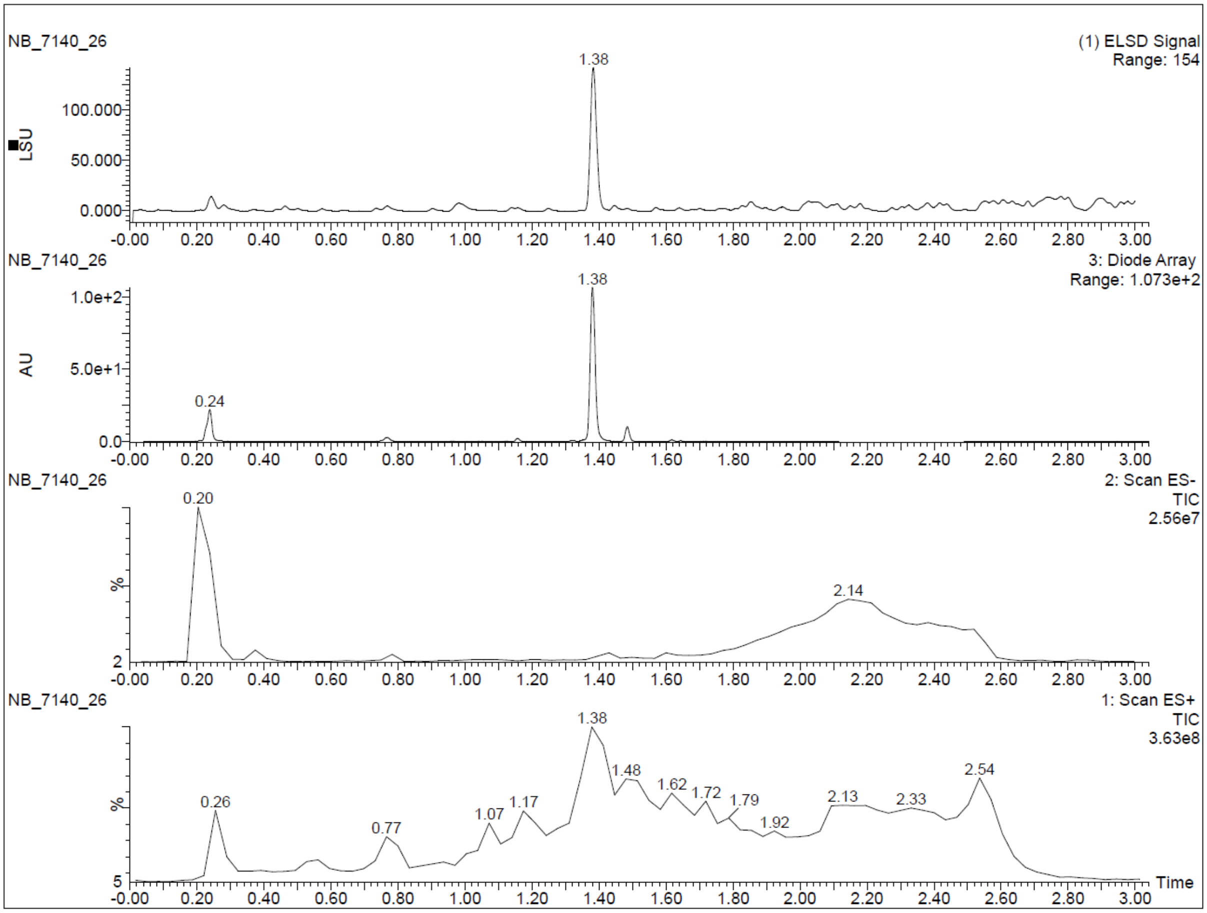
NB_7140_26
(1) ELSD Signal
Range: 154
LSU
1.38
NB_7140_26
3: Diode Array
Range: 1.073e+2
AU
0.24
1.38
NB_7140_26
2: Scan ES-
TIC
2.56e7
0.20
2.14
NB_7140_26
1: Scan ES+
TIC
3.63e8
0.26
0.77
1.07
1.17
1.38
1.48
1.62
1.72
1.79
1.92
2.13
2.33
2.54
Time

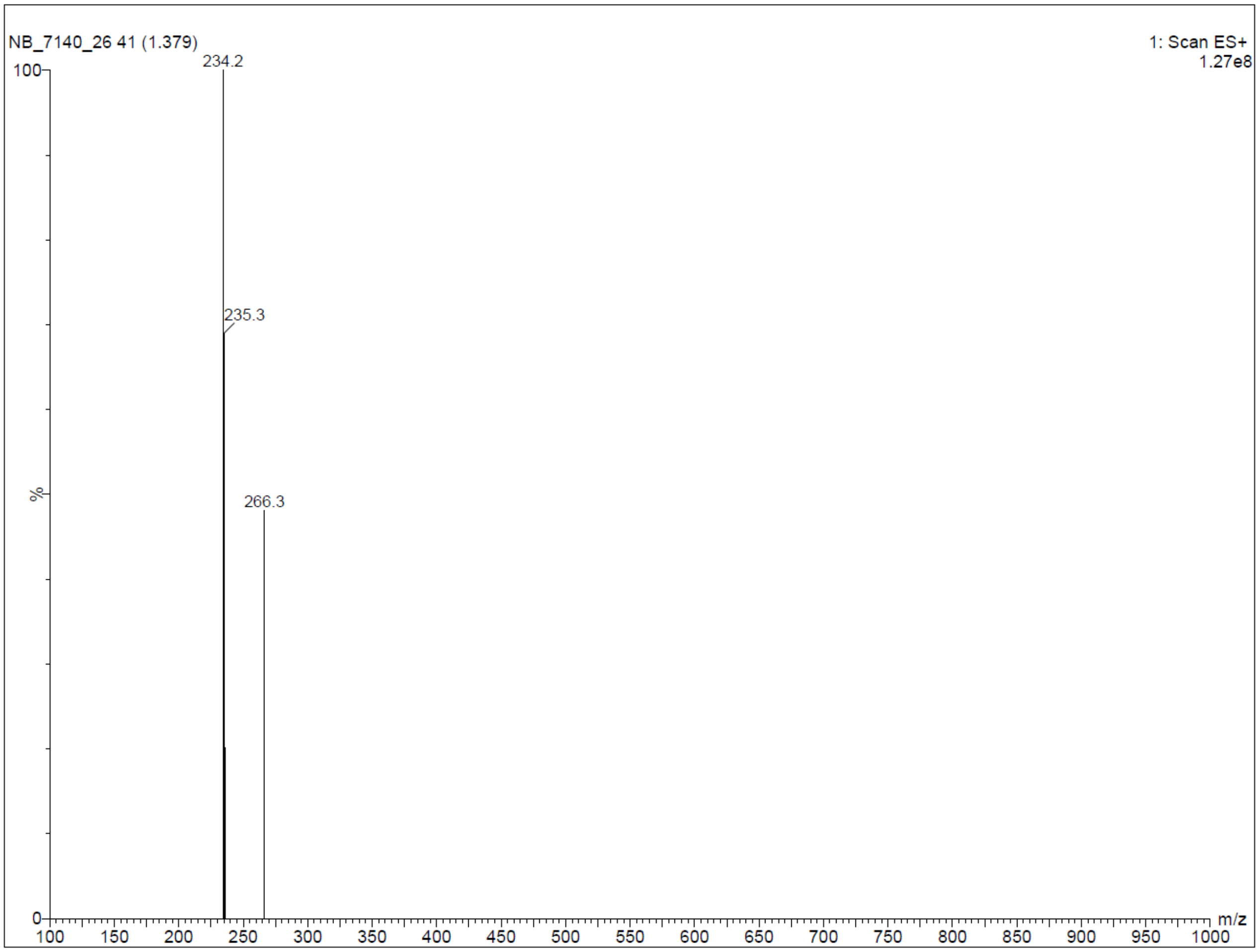

(S)-3-(4-Methoxyphenyl)-5-methylmorpholine, TFA Salt (4ca)

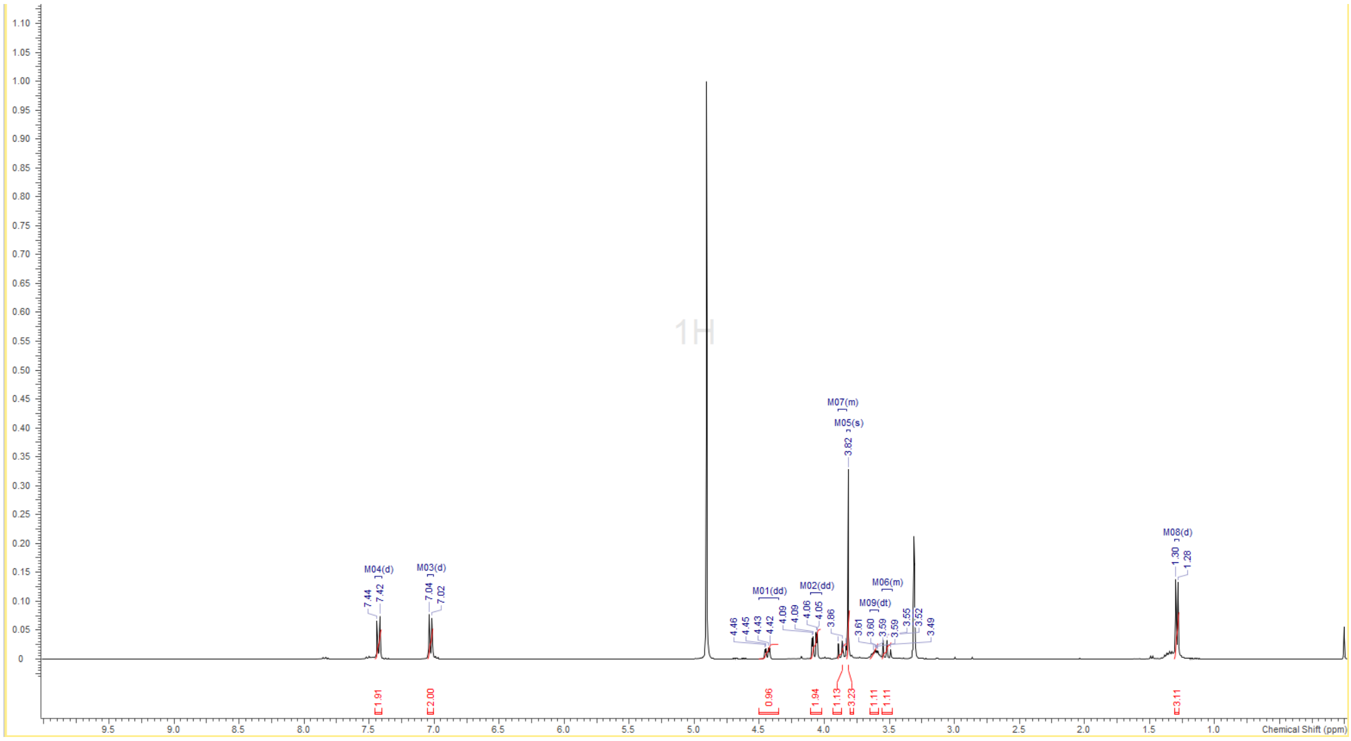

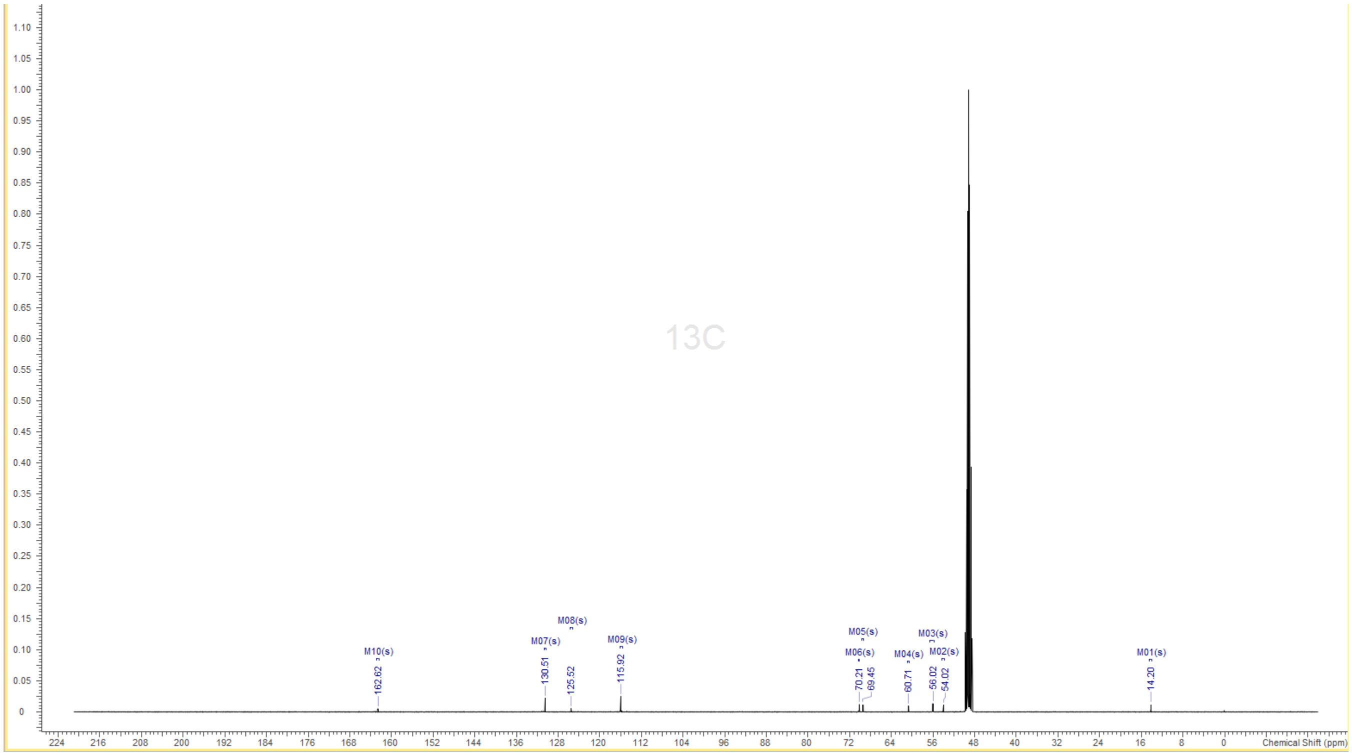
13C
M10(s)
162.62
M07(s)
130.51
M08(s)
125.52
M09(s)
115.92
M05(s)
M06(s)
70.21
69.45
M04(s)
60.71
M03(s)
56.02
M02(s)
54.02
M01(s)
14.20
Chemical Shift (ppm)

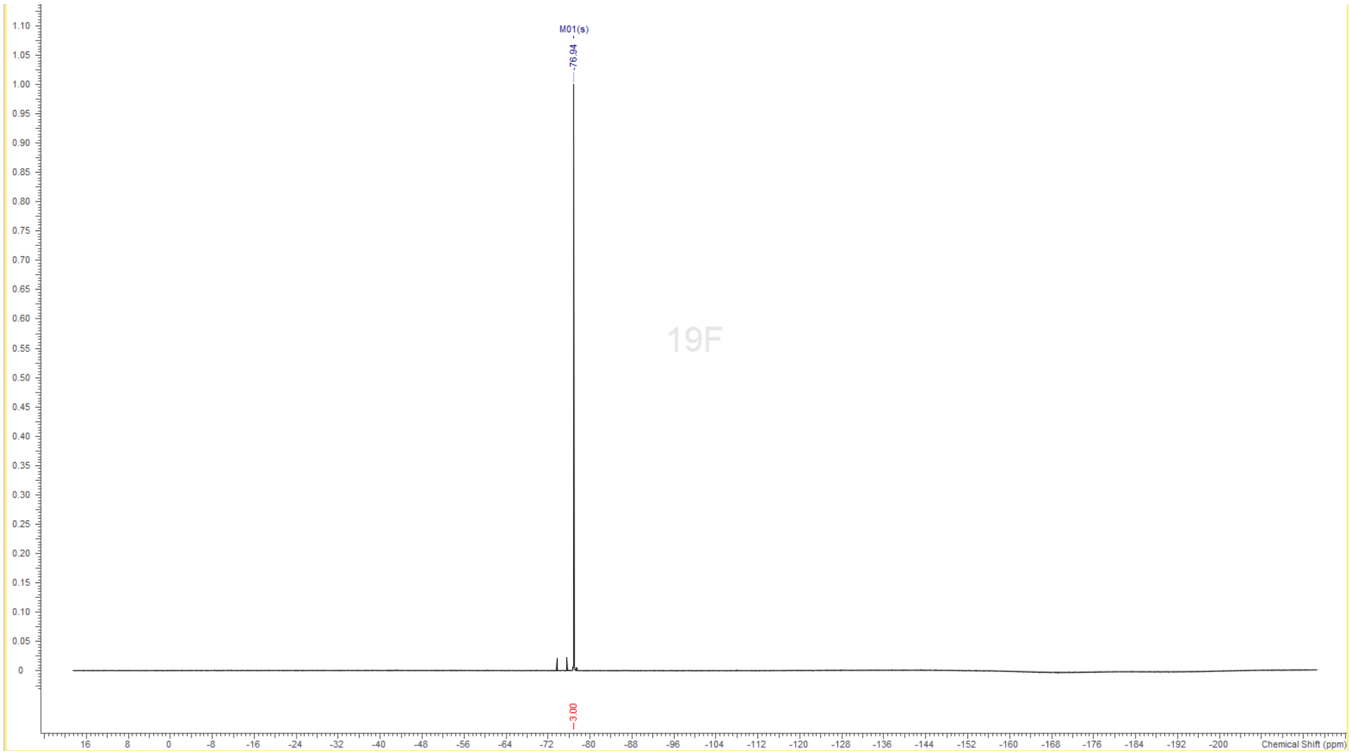
19F
M01(s)
-76.94
3.00
Chemical Shift (ppm)

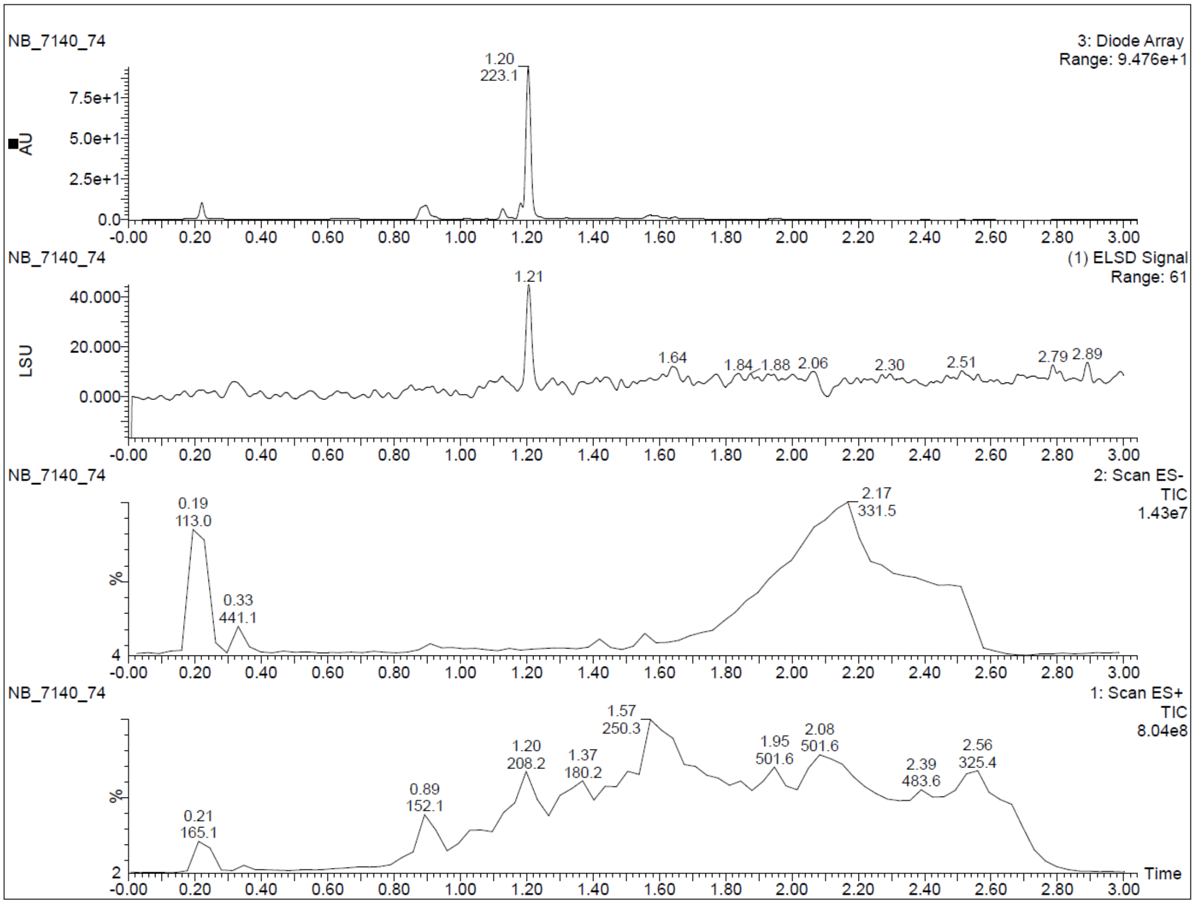

NB_7140_74
3: Diode Array
Range: 9.476e+1
AU
1.20
223.1
NB_7140_74
(1) ELSD Signal
Range: 61
LSU
1.21
1.64
1.84
1.88
2.06
2.30
2.51
2.79
2.89
NB_7140_74
2: Scan ES-
TIC
1.43e7
0.19
113.0
0.33
441.1
2.17
331.5
NB_7140_74
1: Scan ES+
TIC
8.04e8
0.21
165.1
0.89
152.1
1.20
208.2
1.37
180.2
1.57
250.3
1.95
501.6
2.08
501.6
2.39
483.6
2.56
325.4
Time

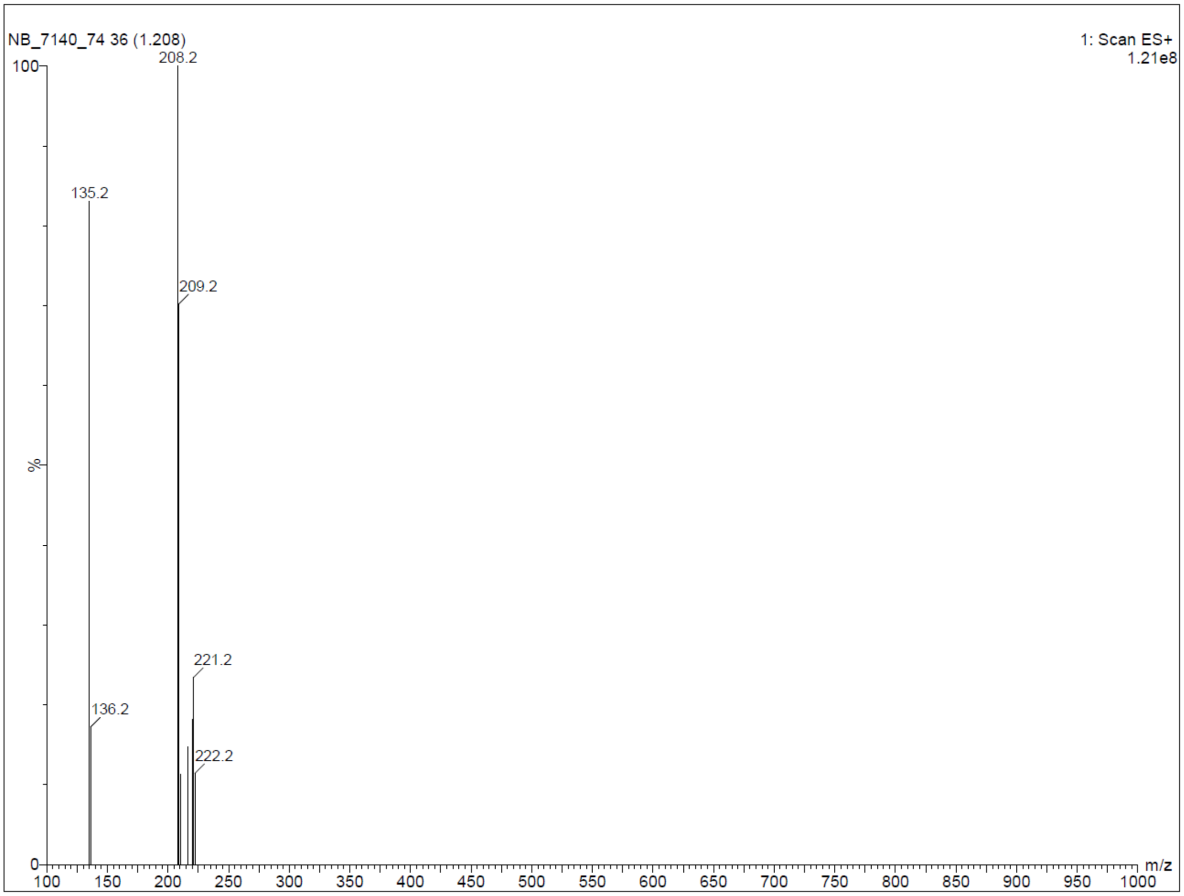

NB_7140_74 36 (1.208)
1: Scan ES+
1.21e8
208.2
135.2
209.2
221.2
136.2
222.2
m/z

(2S)-2-Methyl-5-(6-phenyl-2,3-dihydroimidazo[2,1-b]thiazol-5-yl)morpholine, Free base (4dw)

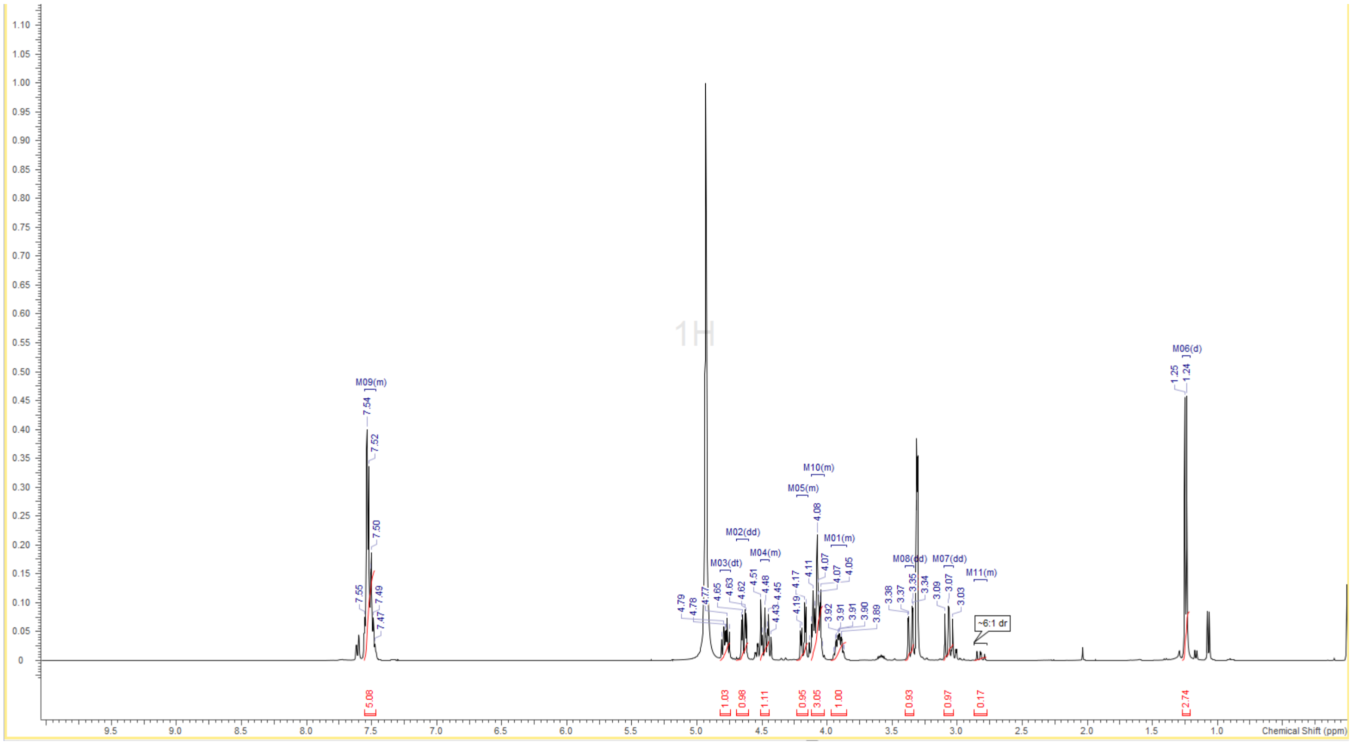

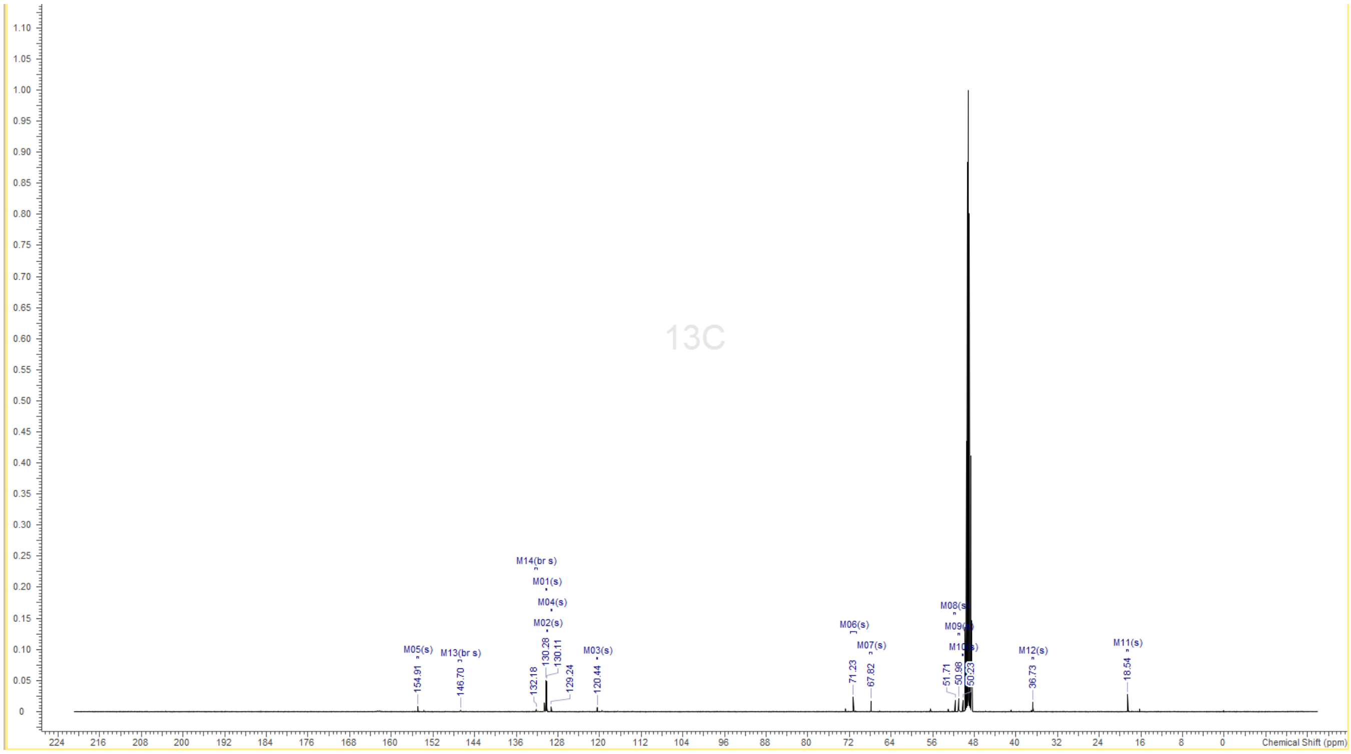

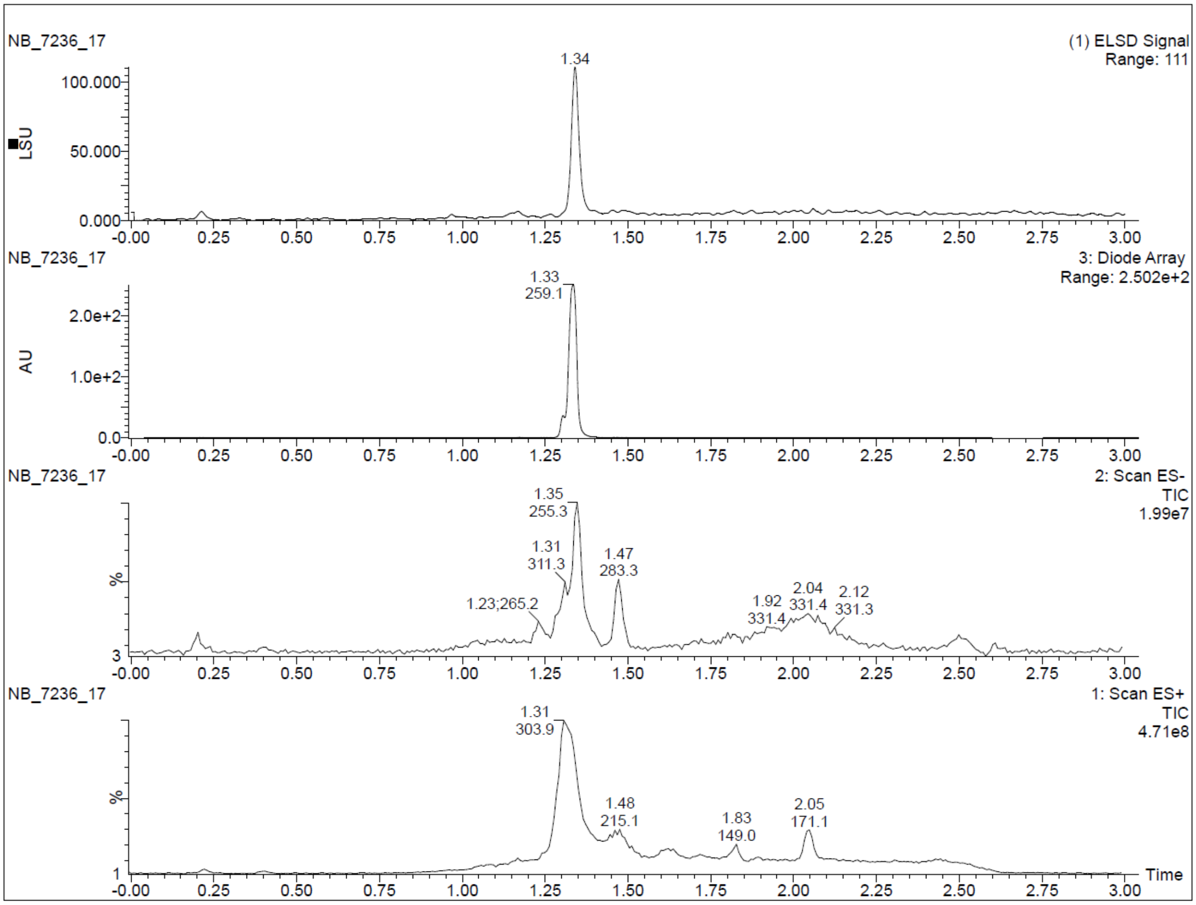
NB_7236_17
(1) ELSD Signal
Range: 111
1.34
LSU
3: Diode Array
Range: 2.502e+2
1.33
259.1
AU
2: Scan ES-
TIC
1.99e7
1.35
255.3
1.31
311.3
1.47
283.3
1.23;265.2
1.92
331.4
2.04
331.4
2.12
331.3
1: Scan ES+
TIC
4.71e8
1.31
303.9
1.48
215.1
1.83
149.0
2.05
171.1
Time

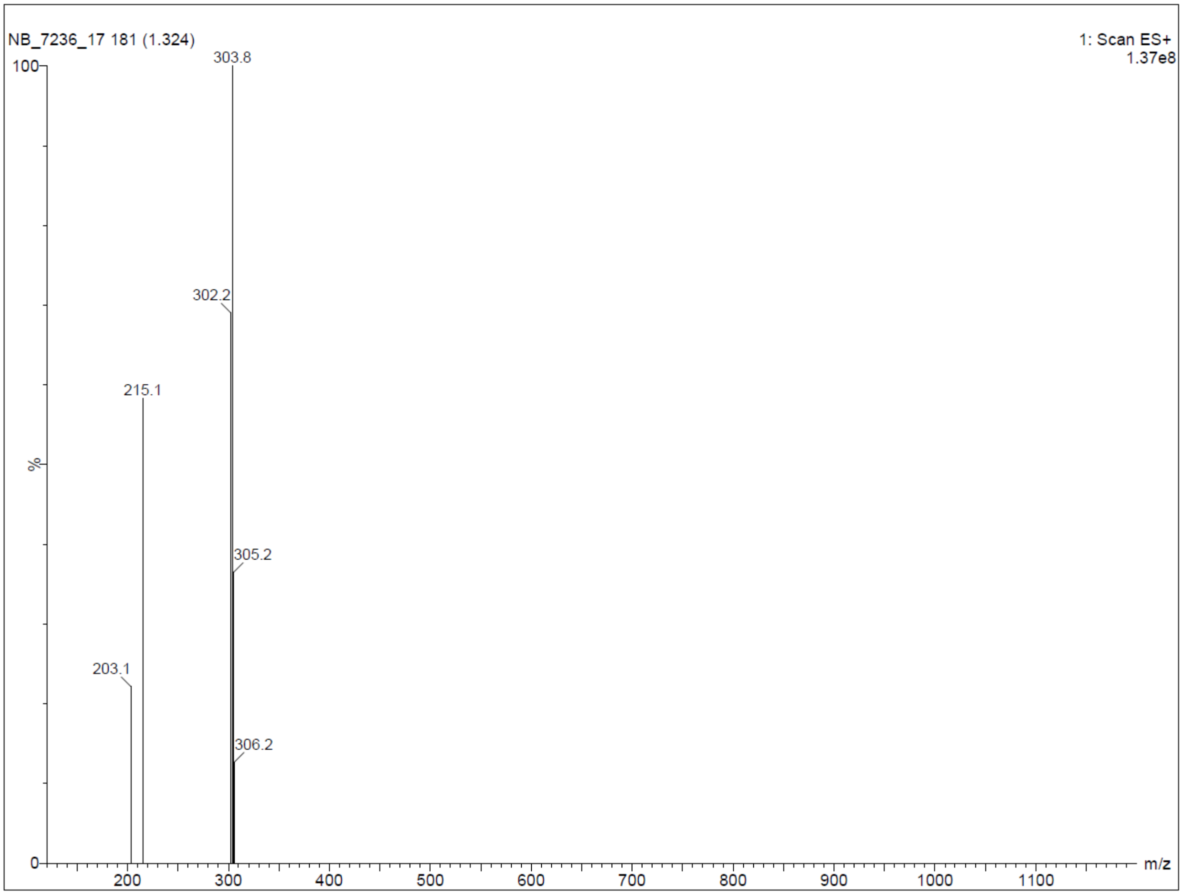
NB_7236_17 181 (1.324)
1: Scan ES+
1.37e8
303.8
302.2
215.1
305.2
203.1
306.2
%
m/z

(3R,4S,7S)-3-(4-Methoxyphenyl)hexahydro-2H-furo[3,4-b][1,4]oxazine, Free base (4fa)

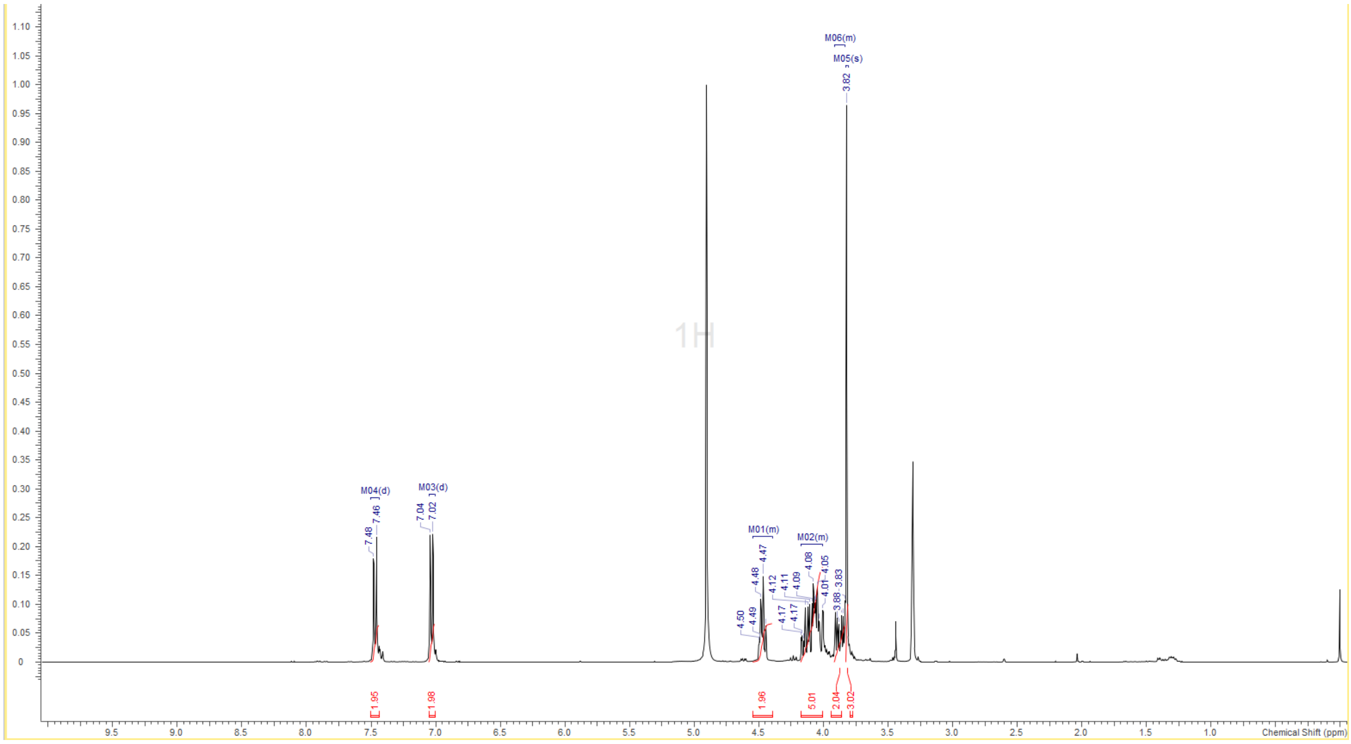

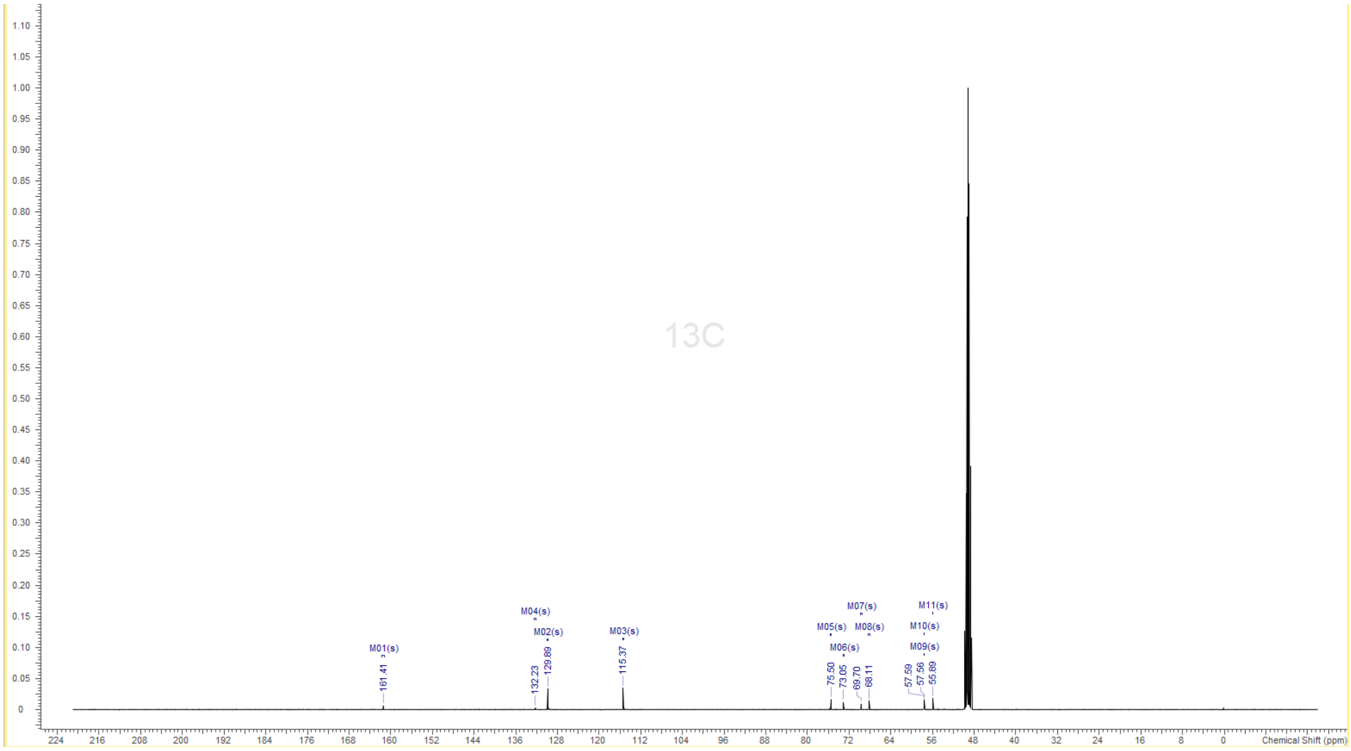

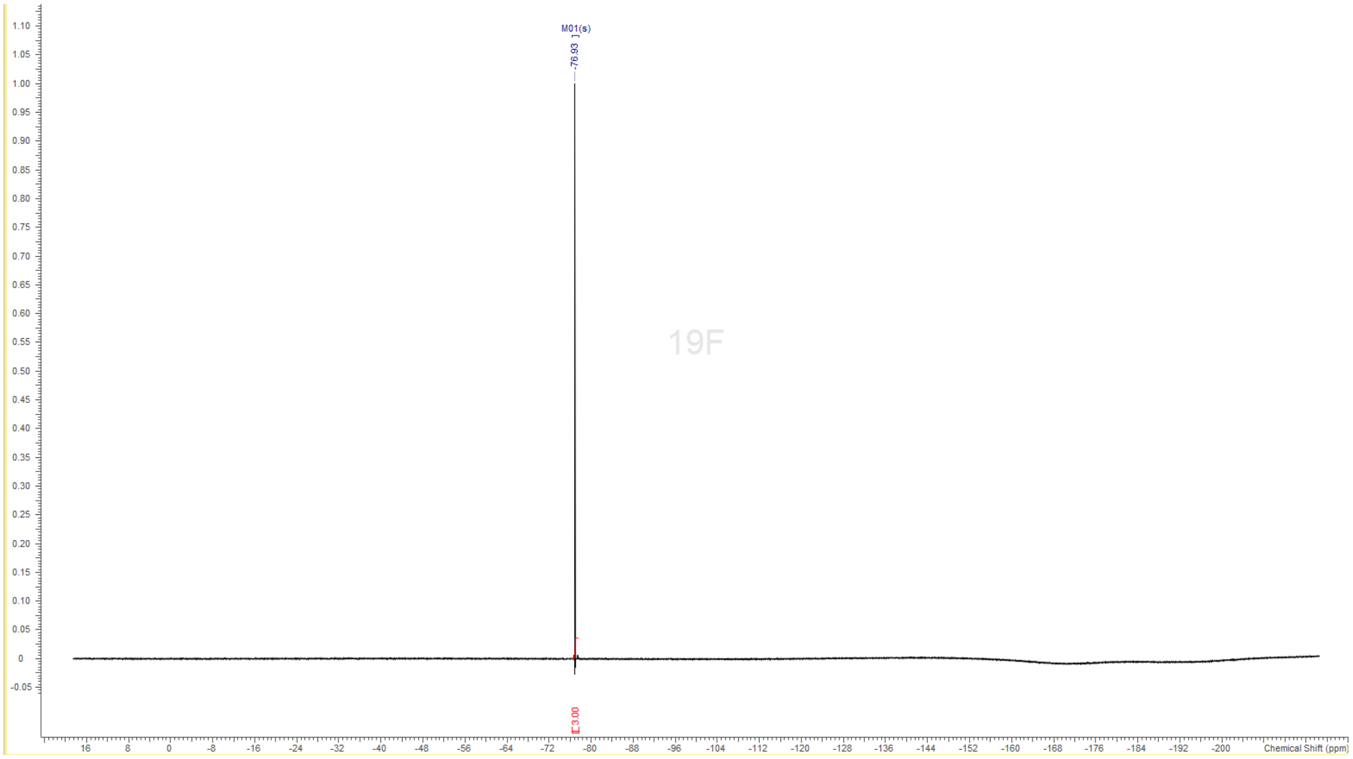

M01(s)
-76.93
19F
3.00
Chemical Shift (ppm)

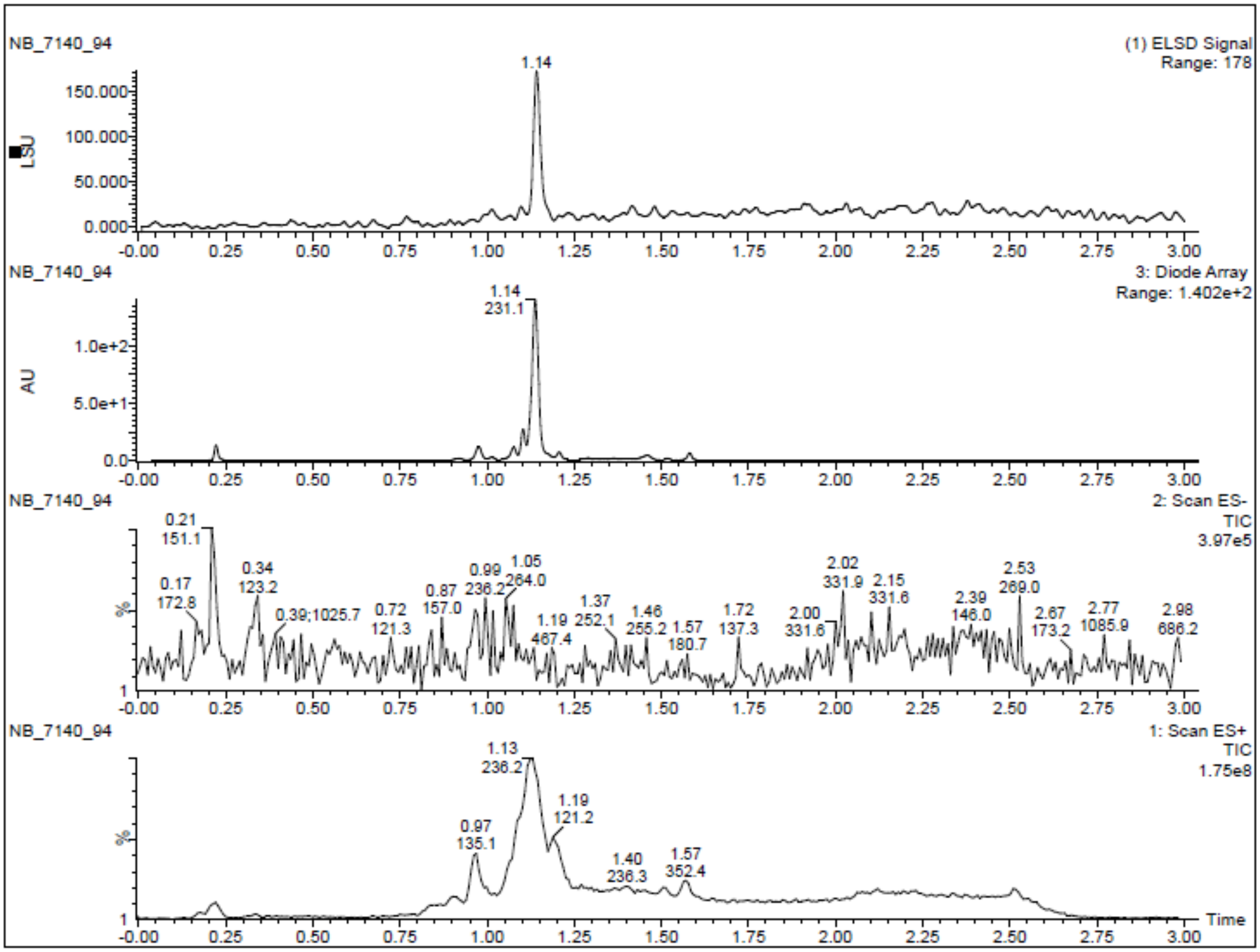

NB_7140_94
(1) ELSD Signal
Range: 178
LSU
1.14
NB_7140_94
3: Diode Array
Range: 1.402e+2
AU
1.14
231.1
NB_7140_94
2: Scan ES-
TIC
3.97e5
0.21
151.1
0.17
172.8
0.34
123.2
0.39;1025.7
0.72
121.3
0.87
157.0
0.99
236.2
1.05
264.0
1.19
467.4
1.37
252.1
1.46
255.2
1.57
180.7
1.72
137.3
2.00
331.6
2.02
331.9
2.15
331.6
2.39
146.0
2.53
269.0
2.67
173.2
2.77
1085.9
2.98
686.2
NB_7140_94
1: Scan ES+
TIC
1.75e8
0.97
135.1
1.13
236.2
1.19
121.2
1.40
236.3
1.57
352.4
Time

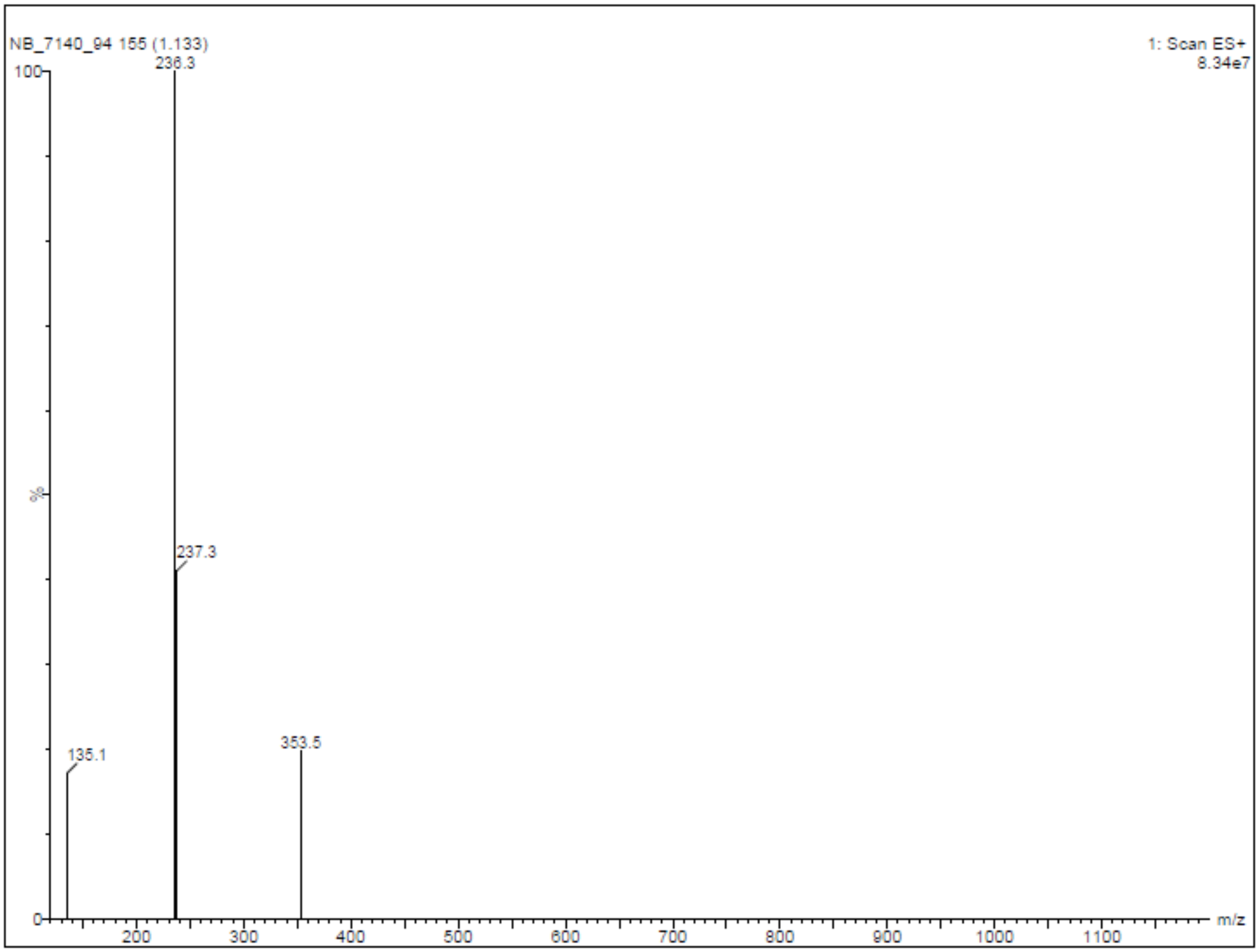

(3R,4S,7R)-3-(4-Methoxyphenyl)hexahydro-2H-furo[3,4-b][1,4]oxazine, Free base (4ga)

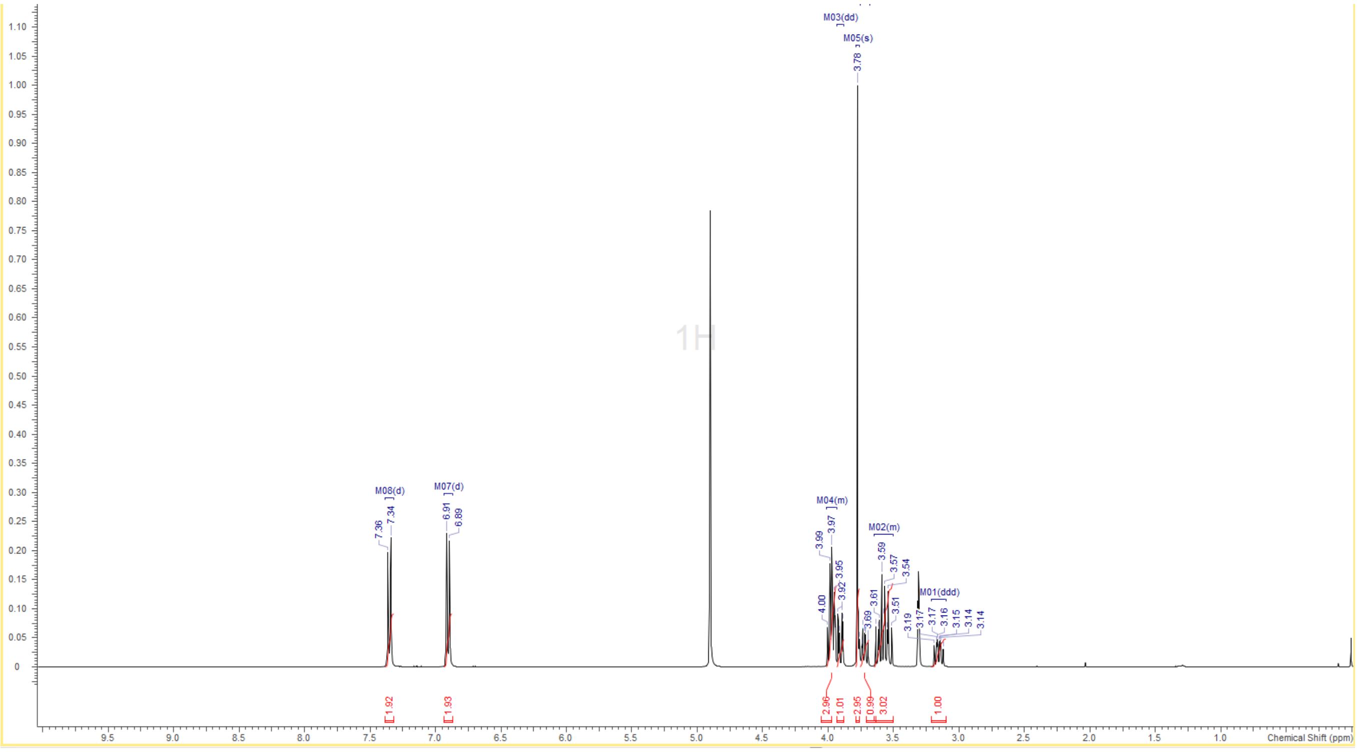

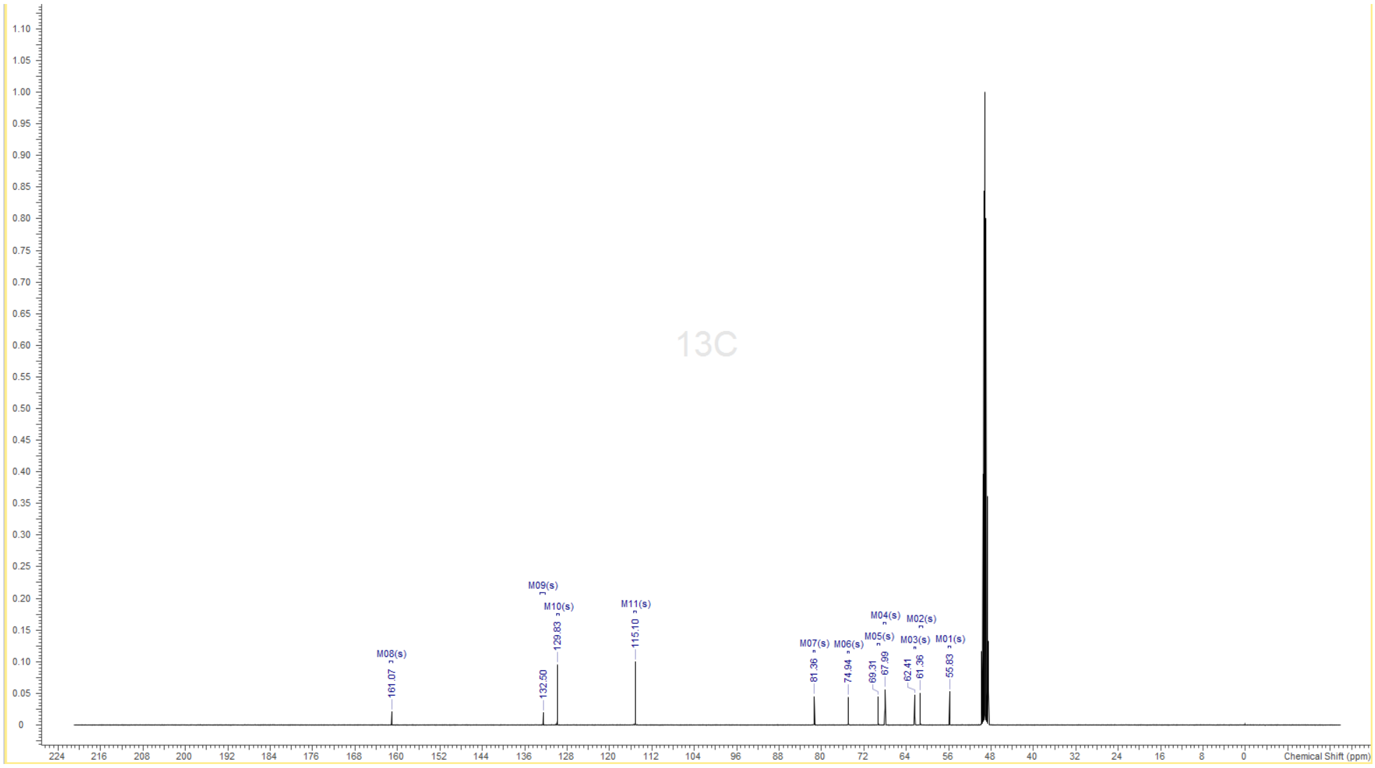
13C
M08(s)
161.07
M09(s)
132.50
M10(s)
129.83
M11(s)
115.10
M07(s)
81.36
M06(s)
74.94
M05(s)
69.31
M04(s)
67.99
M03(s)
62.41
M02(s)
61.36
M01(s)
55.83
Chemical Shift (ppm)

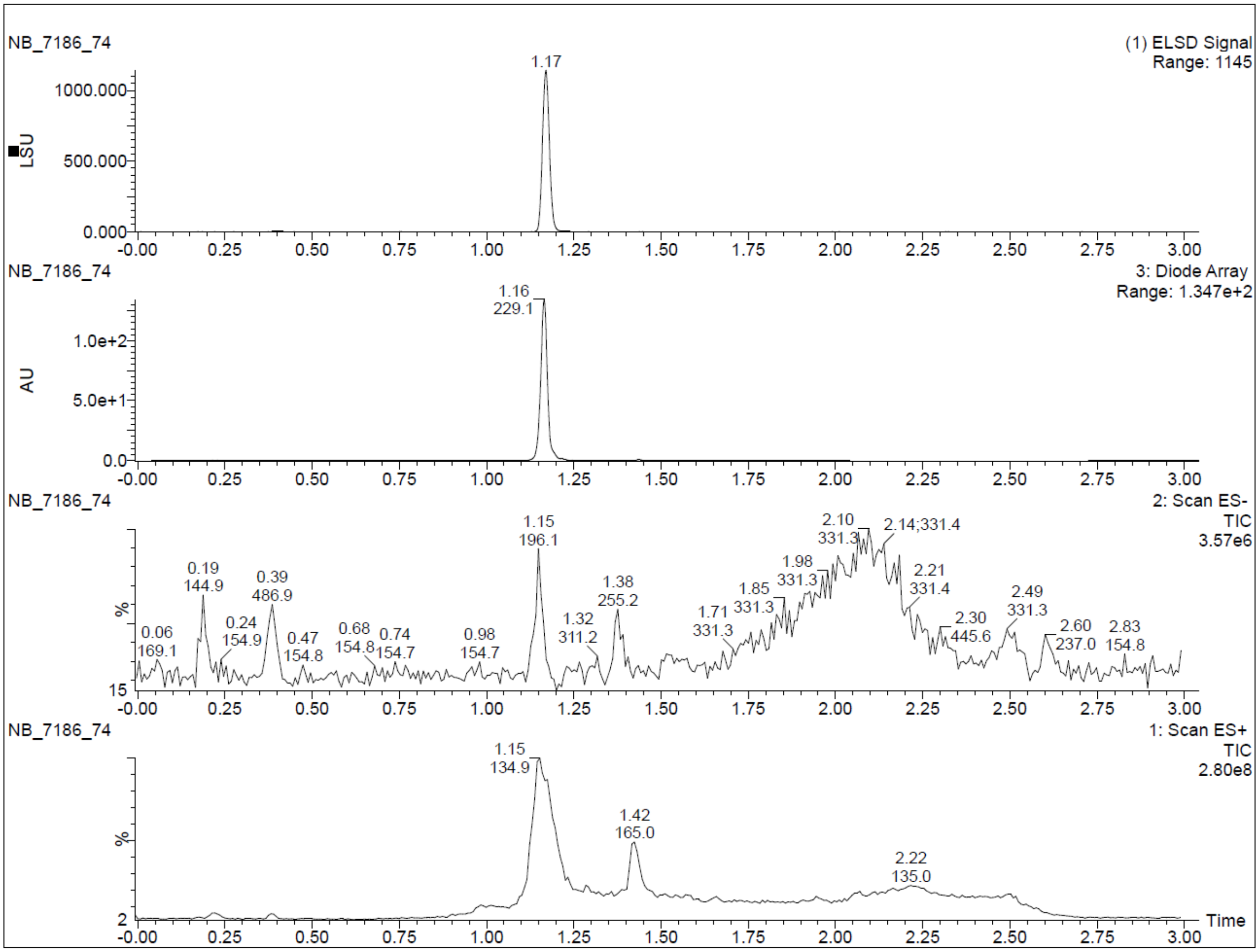
NB_7186_74
(1) ELSD Signal
Range: 1145
1.17
LSU
NB_7186_74
3: Diode Array
Range: 1.347e+2
1.16
229.1
AU
NB_7186_74
2: Scan ES-
TIC
3.57e6
1.15
196.1
1.38
255.2
2.10
331.3
2.14;331.4
NB_7186_74
1: Scan ES+
TIC
2.80e8
1.15
134.9
1.42
165.0
2.22
135.0
Time

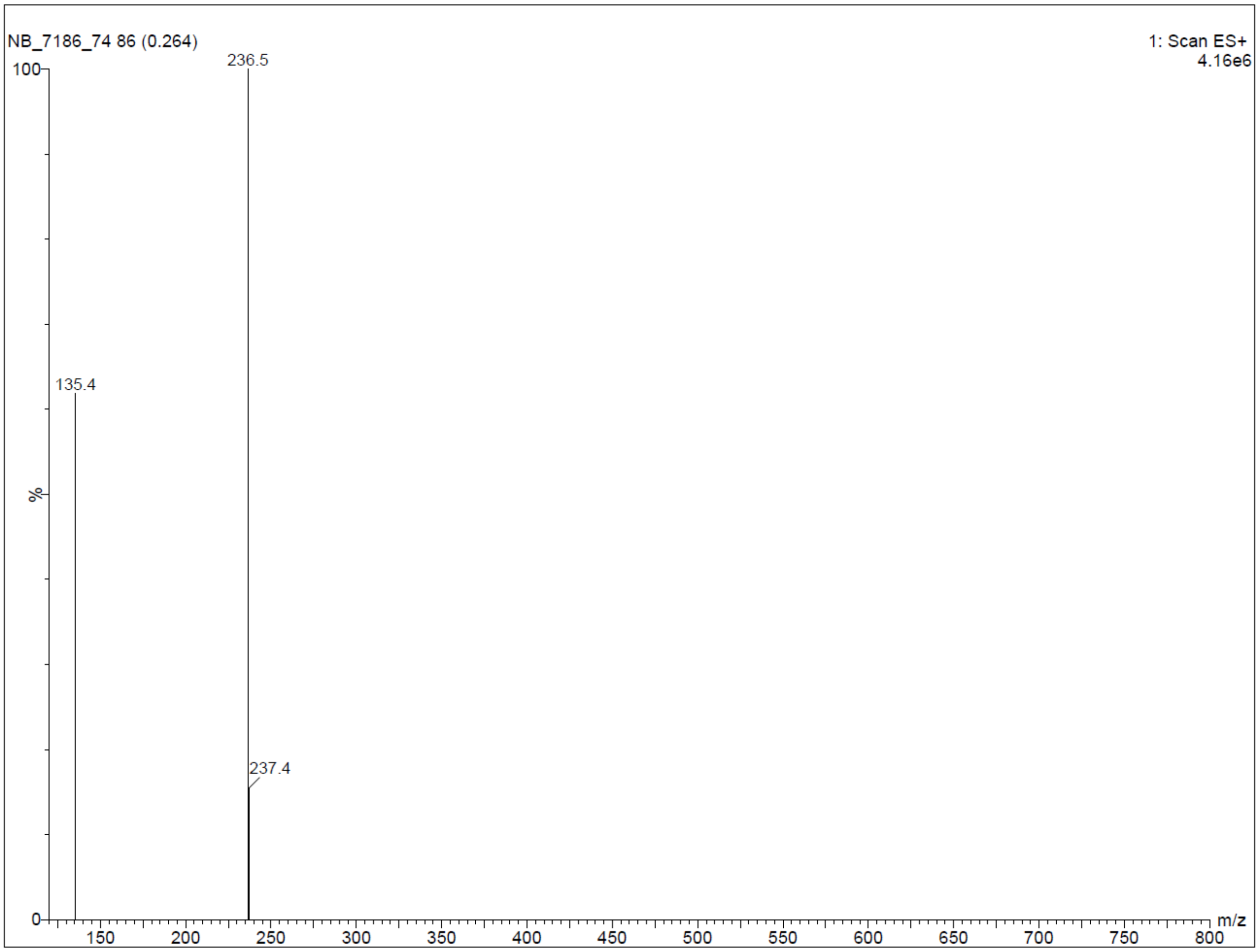

(3R,4S,7R)-3-(4-Methoxyphenyl)-6-tosyloctahydropyrrolo[3,4-b][1,4]oxazine, Free base (4ha)

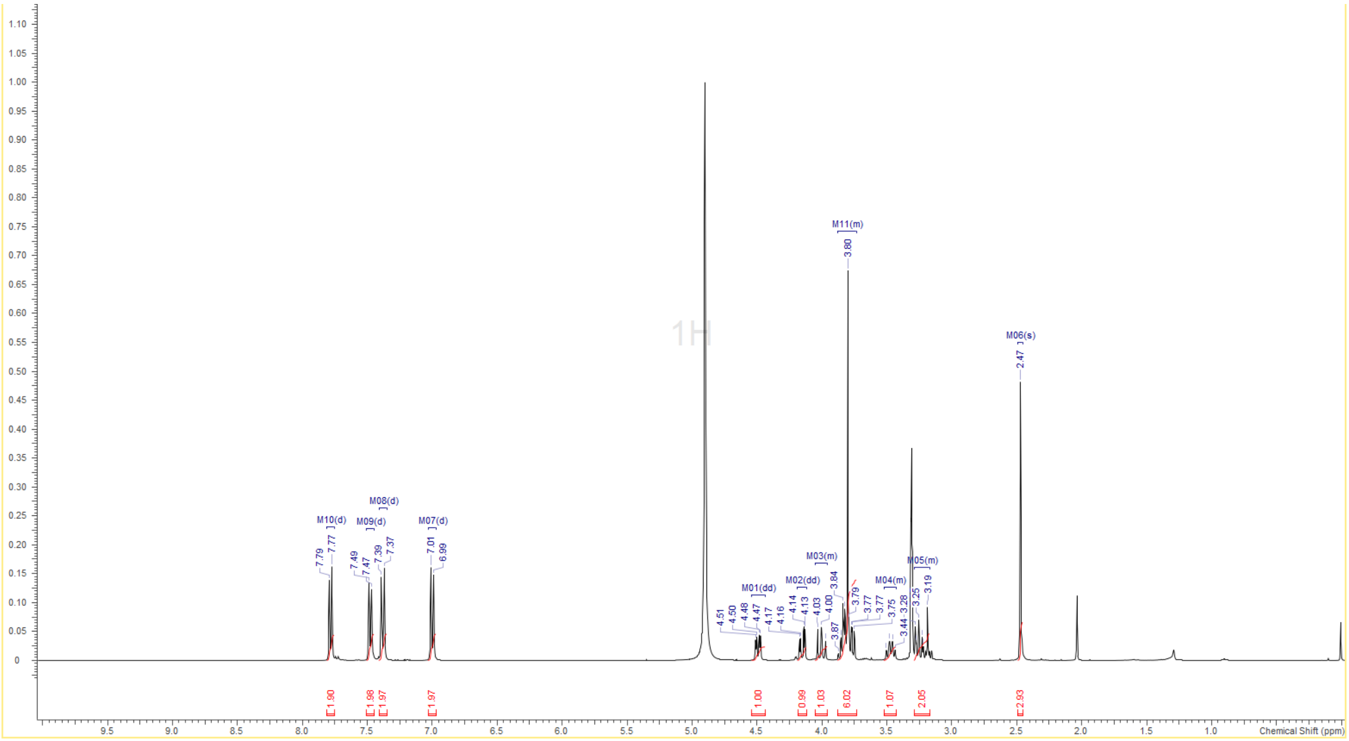

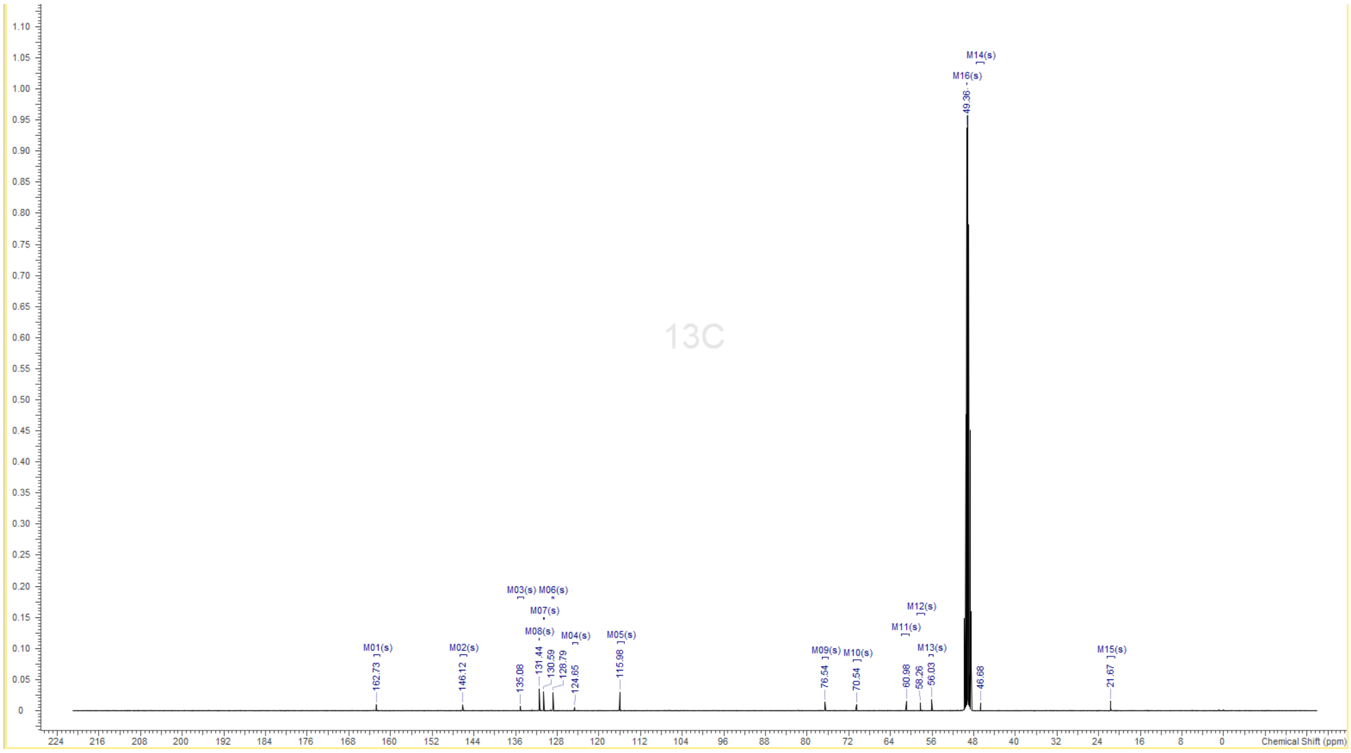
13C
M01(s)
162.73
M02(s)
146.12
M03(s) M06(s)
M07(s)
M08(s)
135.08
131.44
130.59
128.79
M04(s)
124.65
M05(s)
115.98
M09(s)
76.54
M10(s)
70.54
M11(s)
M12(s)
M13(s)
60.98
58.26
56.03
M16(s)
M14(s)
49.36
46.68
M15(s)
21.67
Chemical Shift (ppm)

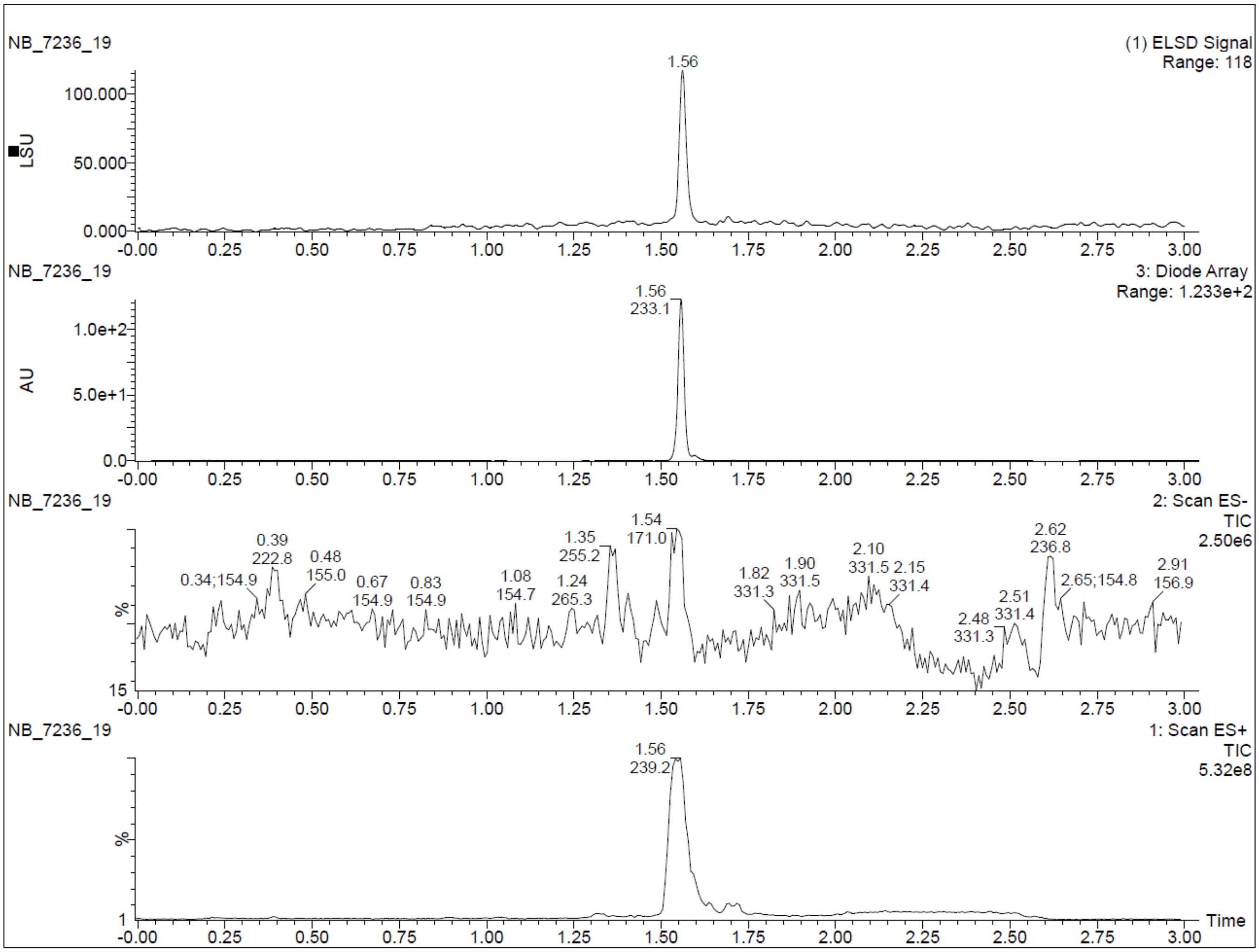
NB_7236_19
(1) ELSD Signal
Range: 118
1.56
LSU
NB_7236_19
3: Diode Array
Range: 1.233e+2
1.56
233.1
AU
NB_7236_19
2: Scan ES-
TIC
2.50e6
0.39
222.8
0.34;154.9
0.48
155.0
0.67
154.9
0.83
154.9
1.08
154.7
1.24
265.3
1.35
255.2
1.54
171.0
1.82
331.3
1.90
331.5
2.10
331.5
2.15
331.4
2.48
331.3
2.51
331.4
2.62
236.8
2.65;154.8
2.91
156.9
%
NB_7236_19
1: Scan ES+
TIC
5.32e8
1.56
239.2
%
Time

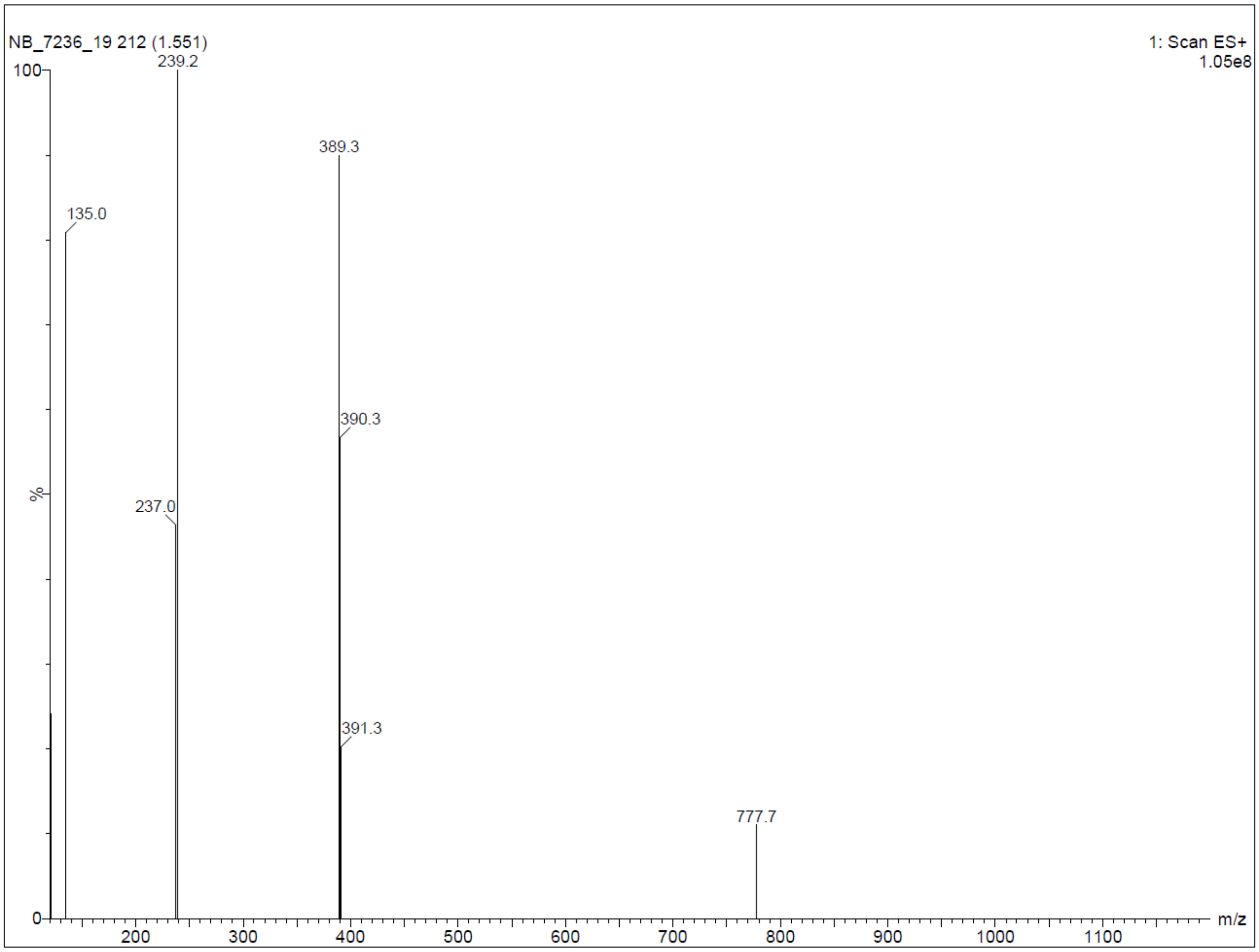

(2S)-5-(4-Methoxyphenyl)-2-phenylmorpholine, Free base (4ea)

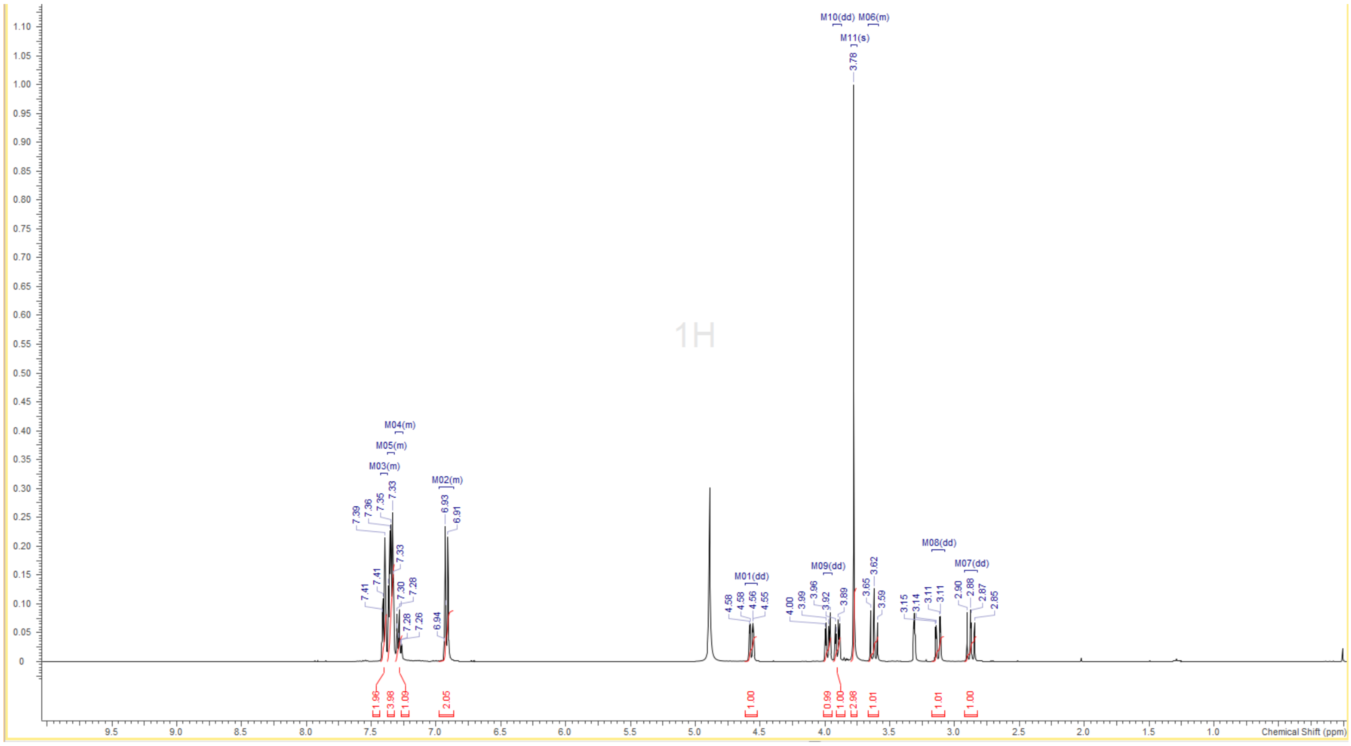

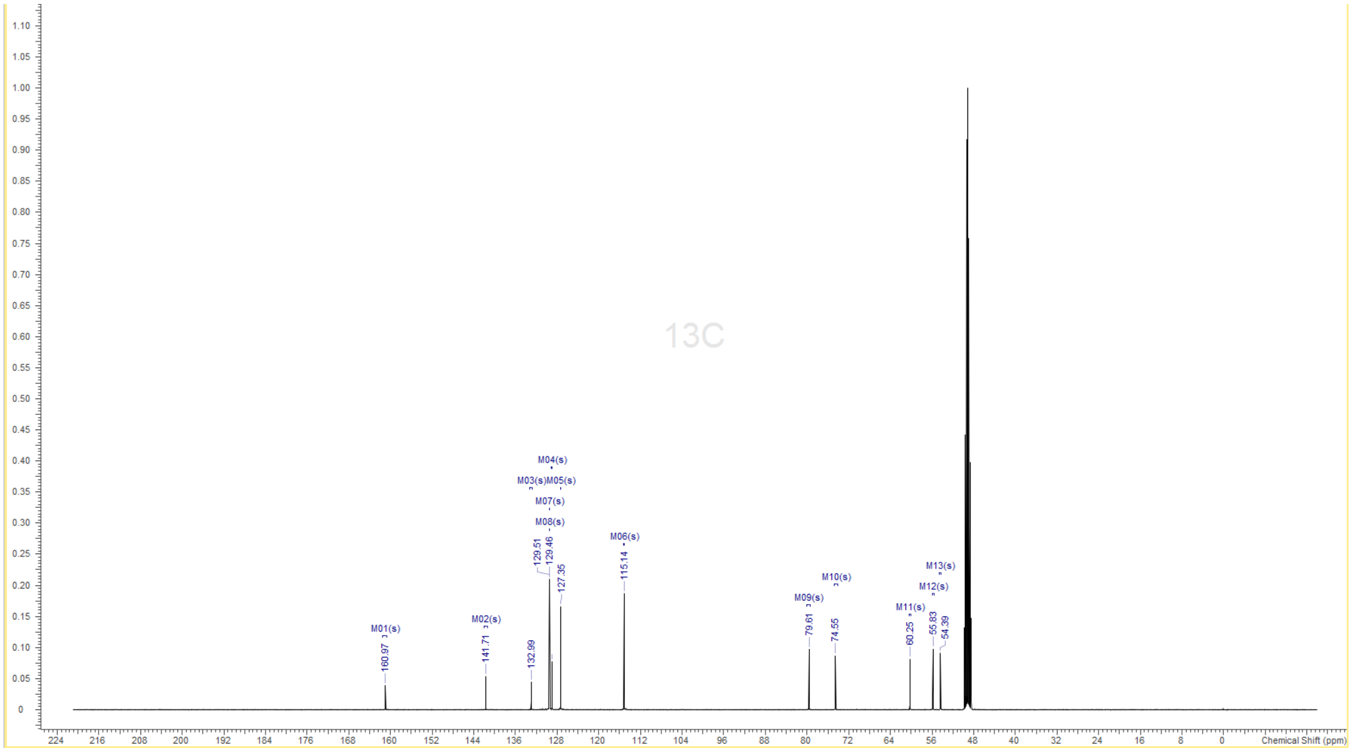
13C
M01(s)
160.97
M02(s)
141.71
132.99
M04(s)
M03(s)M05(s)
M07(s)
M08(s)
129.51
129.46
127.35
M06(s)
115.14
M09(s)
79.61
M10(s)
74.55
M11(s)
60.25
M12(s)
M13(s)
55.83
54.39
Chemical Shift (ppm)

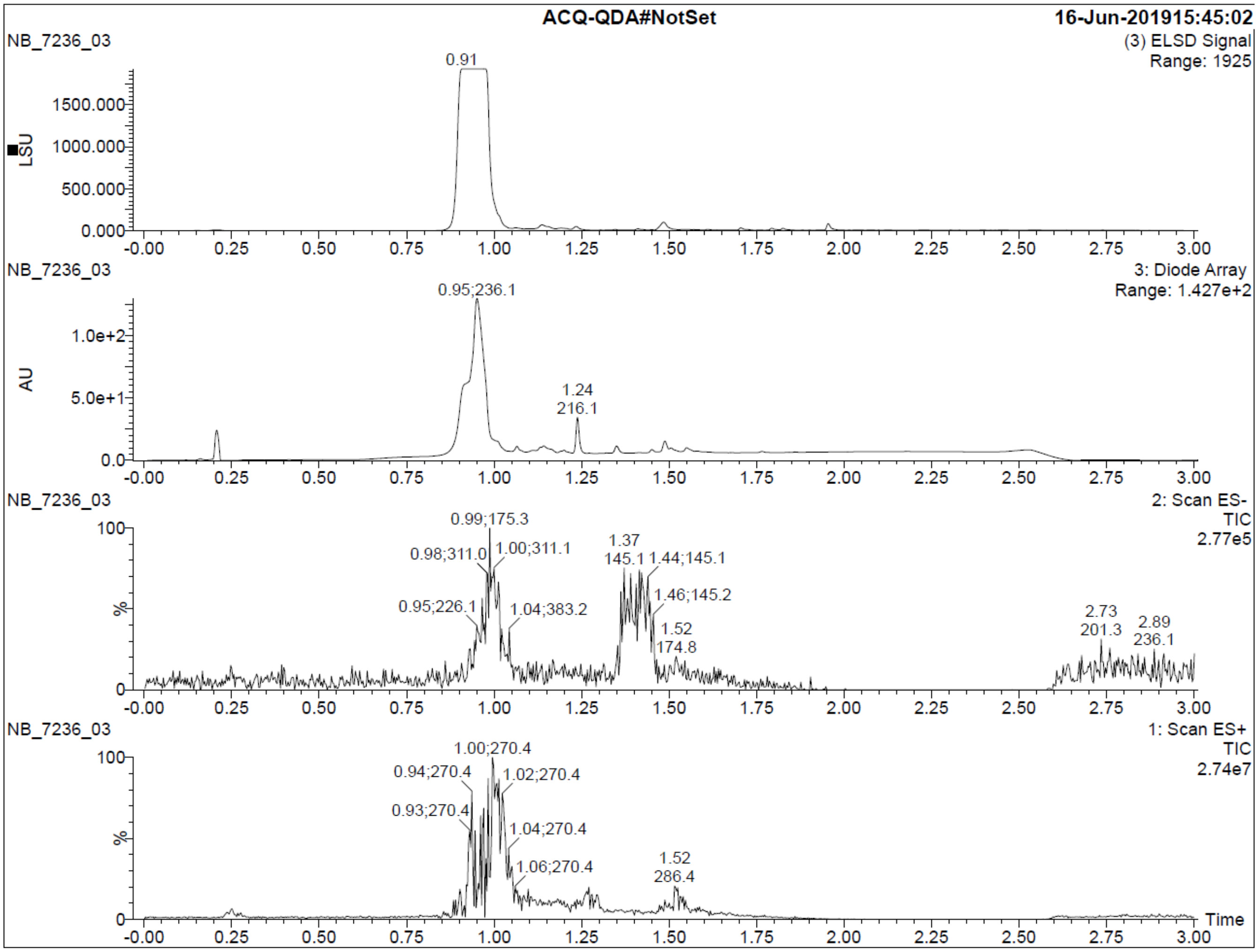
ACQ-QDA#NotSet
16-Jun-201915:45:02
NB_7236_03
(3) ELSD Signal
Range: 1925
0.91
LSU
NB_7236_03
3: Diode Array
Range: 1.427e+2
0.95;236.1
1.24
216.1
AU
NB_7236_03
2: Scan ES-
TIC
2.77e5
0.99;175.3
0.98;311.0
1.00;311.1
1.37
145.1
1.44;145.1
1.46;145.2
0.95;226.1
1.04;383.2
1.52
174.8
2.73
201.3
2.89
236.1
%
NB_7236_03
1: Scan ES+
TIC
2.74e7
1.00;270.4
0.94;270.4
1.02;270.4
0.93;270.4
1.04;270.4
1.06;270.4
1.52
286.4
%
Time

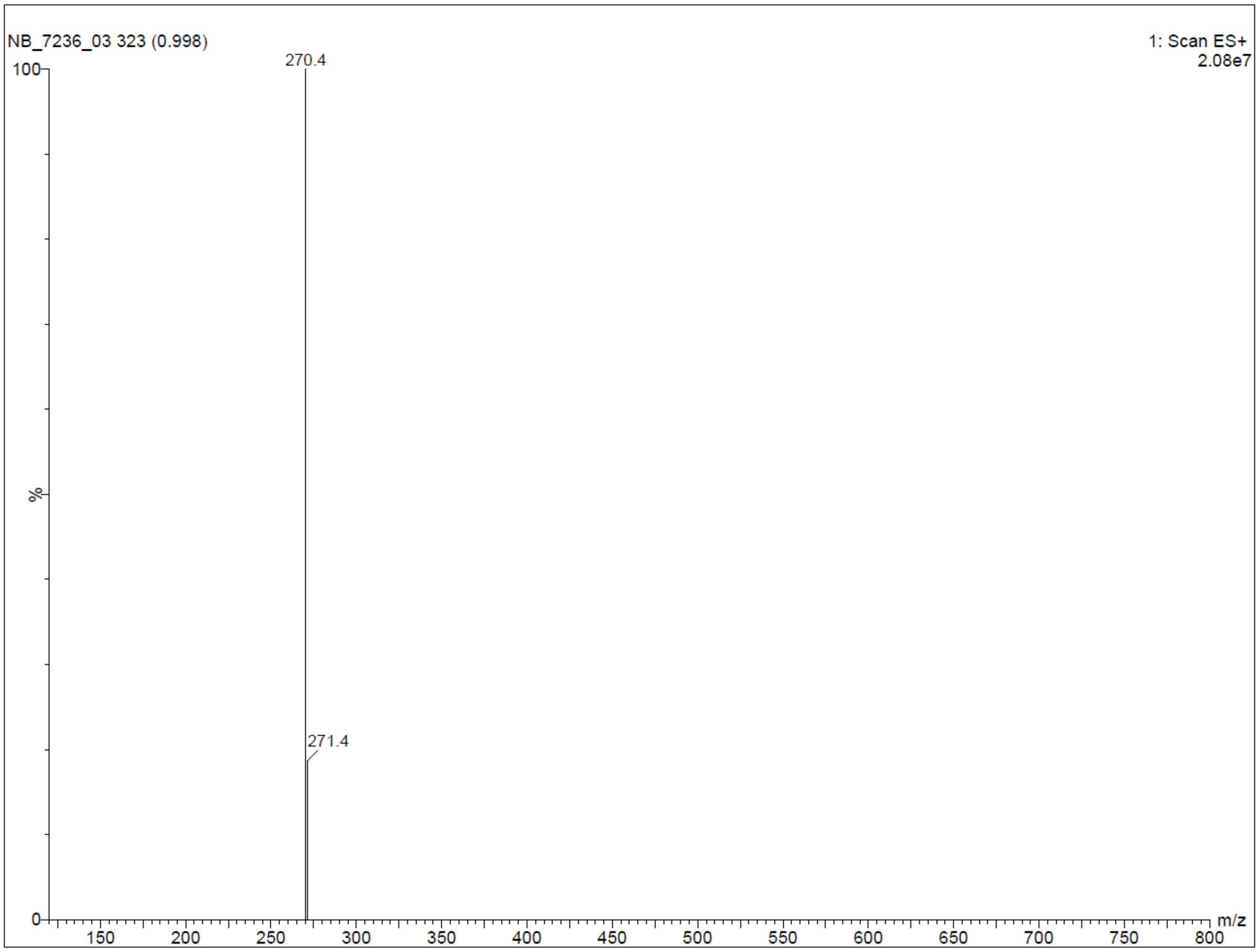

3-(4-Methoxyphenyl)thiomorpholine, Free base (4ja)

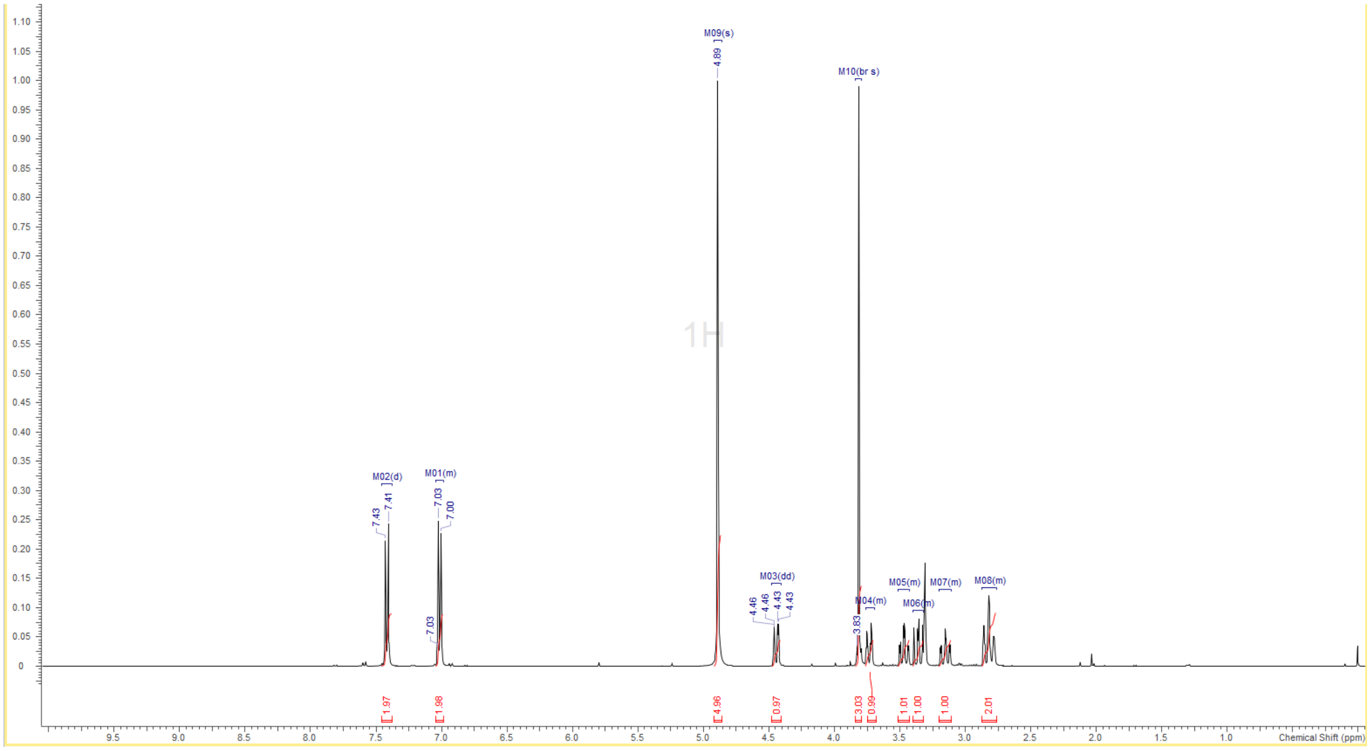

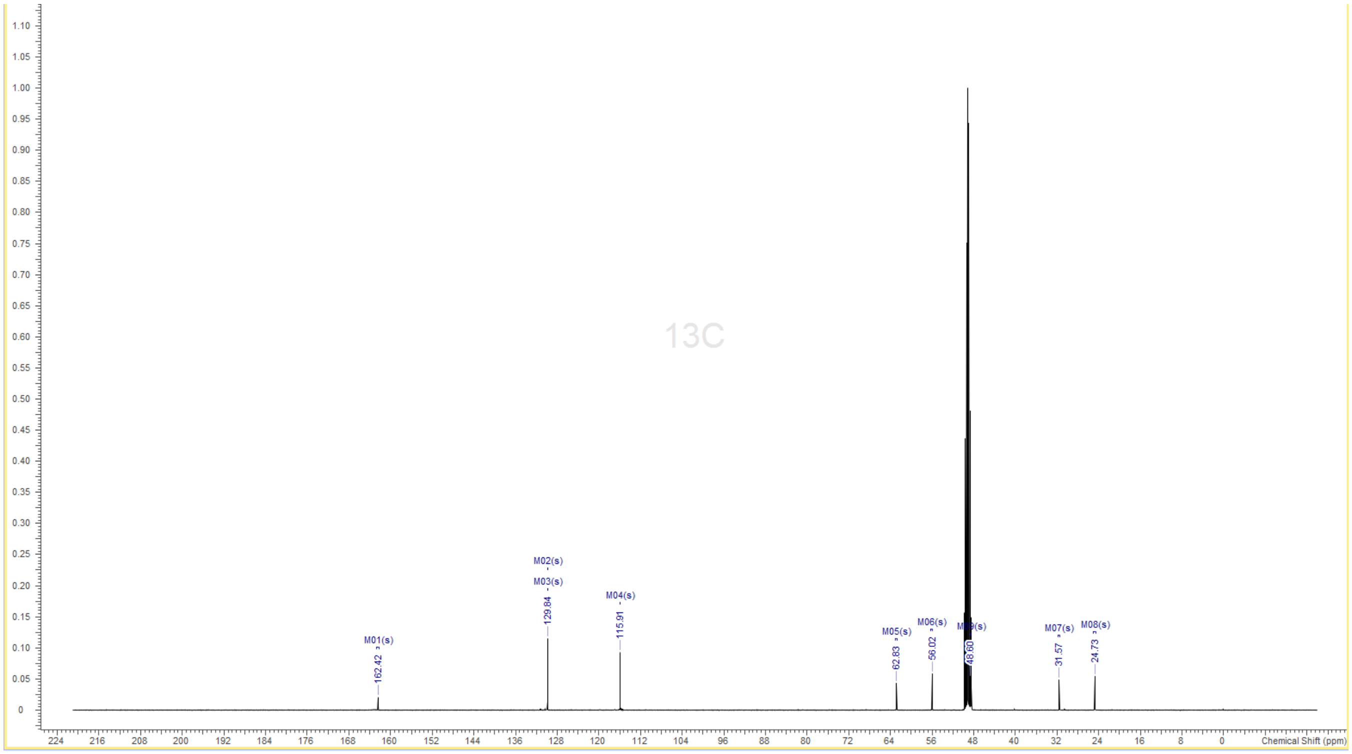
13C
M01(s)
162.42
M02(s)
M03(s)
129.84
M04(s)
115.91
M05(s)
62.83
M06(s)
56.02
48.60
M07(s)
31.57
M08(s)
24.73
Chemical Shift (ppm)

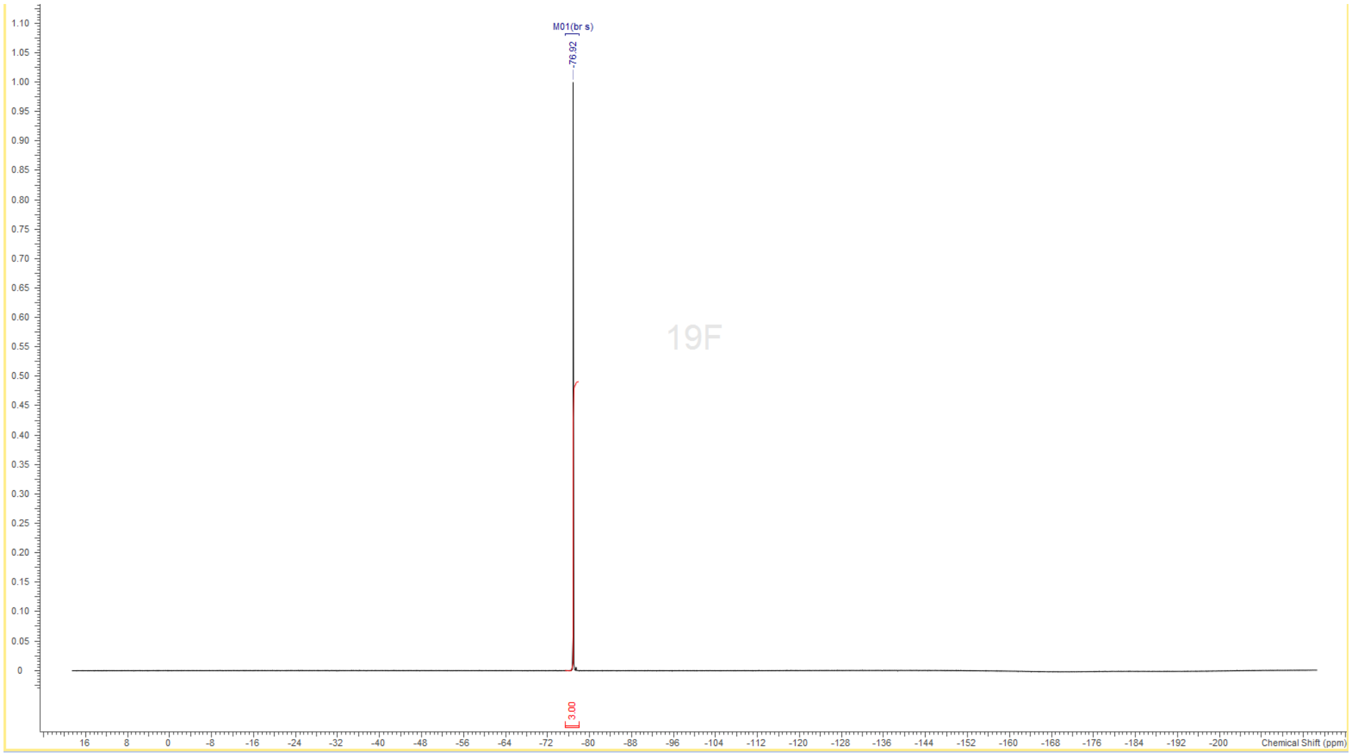
19F
M01(br s)
-76.92
3.00
Chemical Shift (ppm)

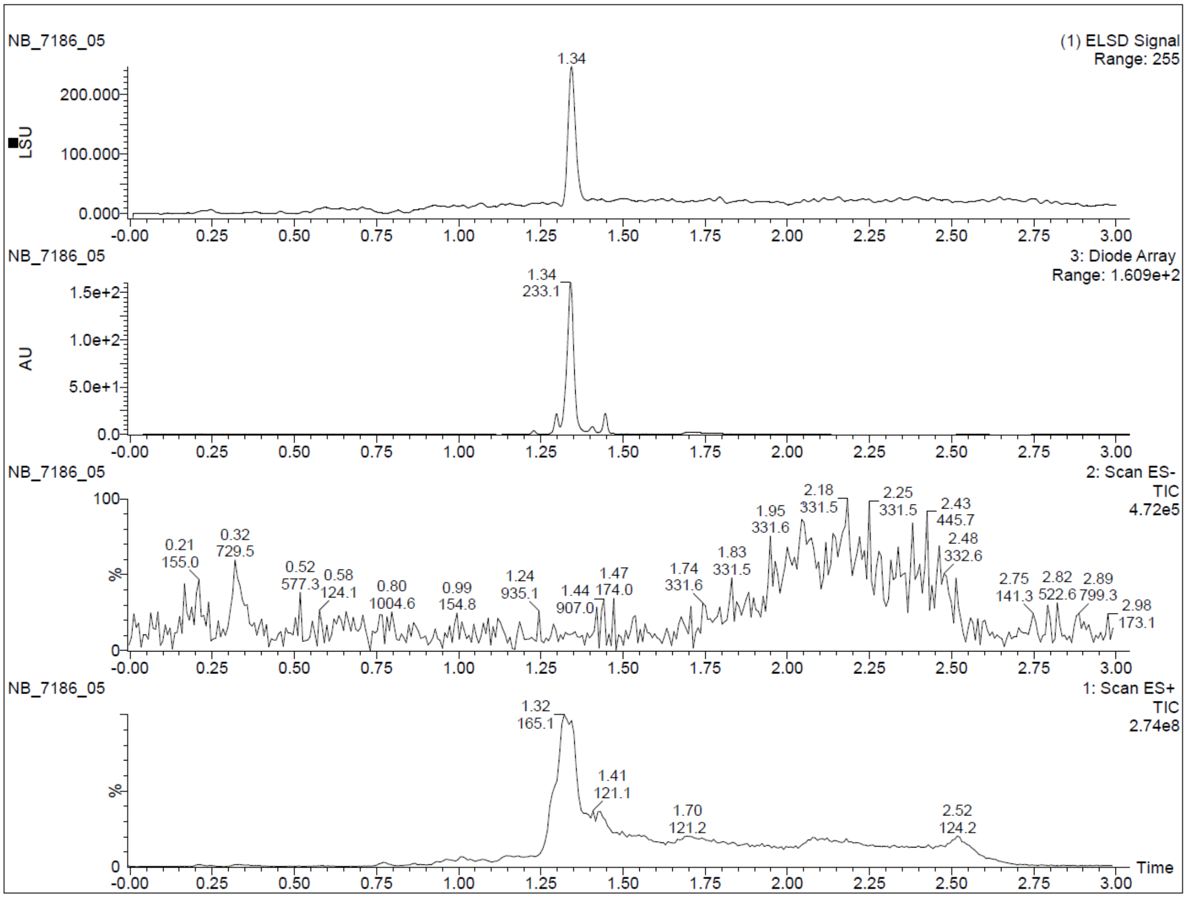

NB_7186_05
(1) ELSD Signal
Range: 255
1.34
LSU
200.000
100.000
0.000
NB_7186_05
3: Diode Array
Range: 1.609e+2
1.34
233.1
AU
1.5e+2
1.0e+2
5.0e+1
0.0
NB_7186_05
2: Scan ES-
TIC
4.72e5
NB_7186_05
1: Scan ES+
TIC
2.74e8
1.32
165.1
1.41
121.1
1.70
121.2
2.52
124.2
Time

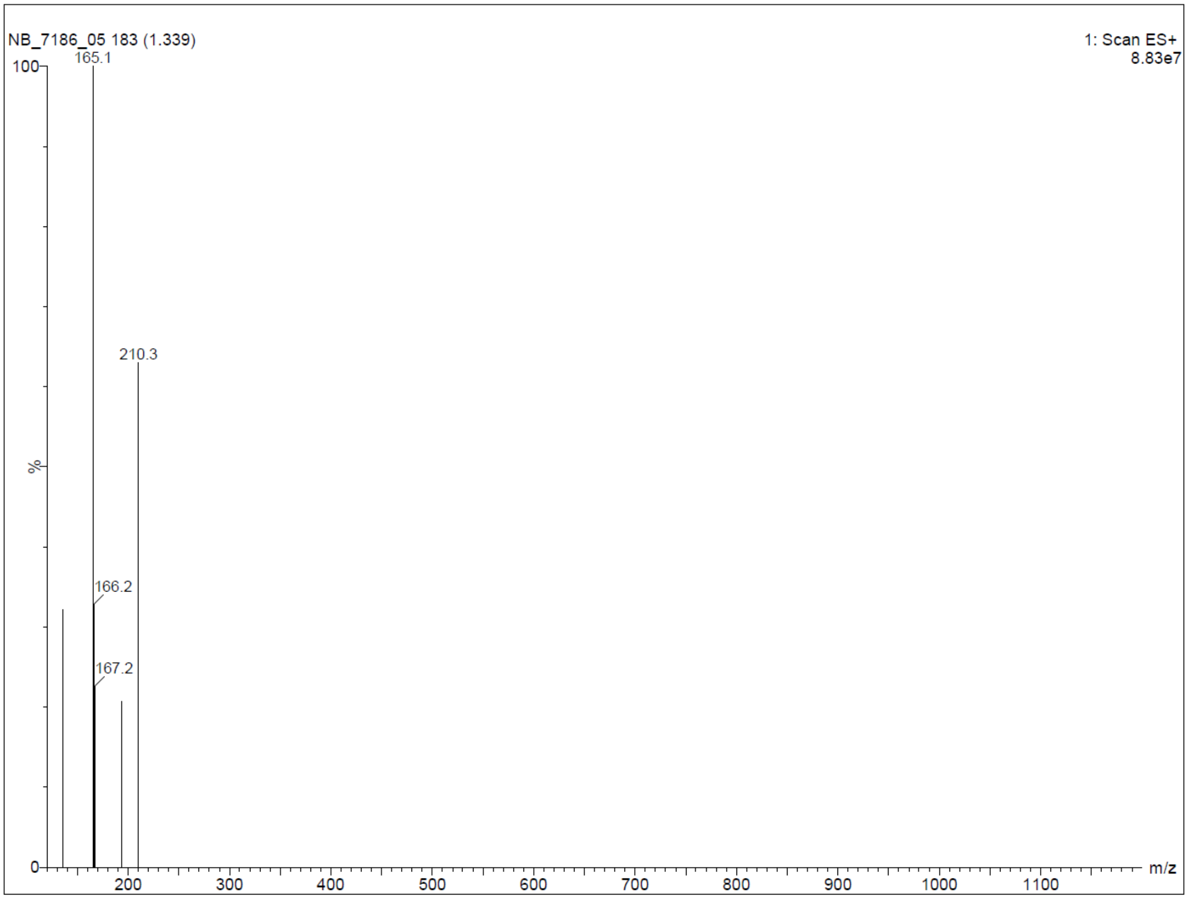

NB_7186_05 183 (1.339)
1: Scan ES+
8.83e7
165.1
210.3
166.2
167.2
m/z

3-(1-Benzyl-1H-imidazol-5-yl)thiomorpholine, Free base (4jq)

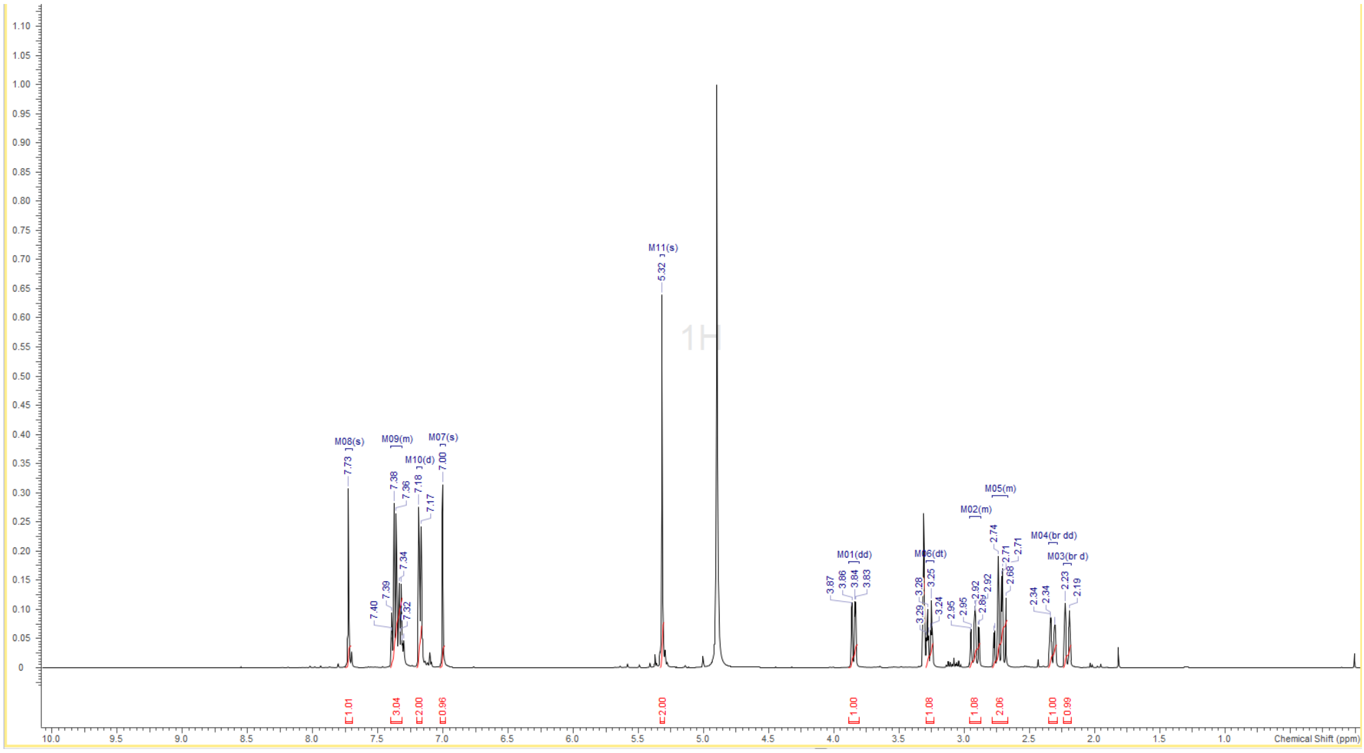

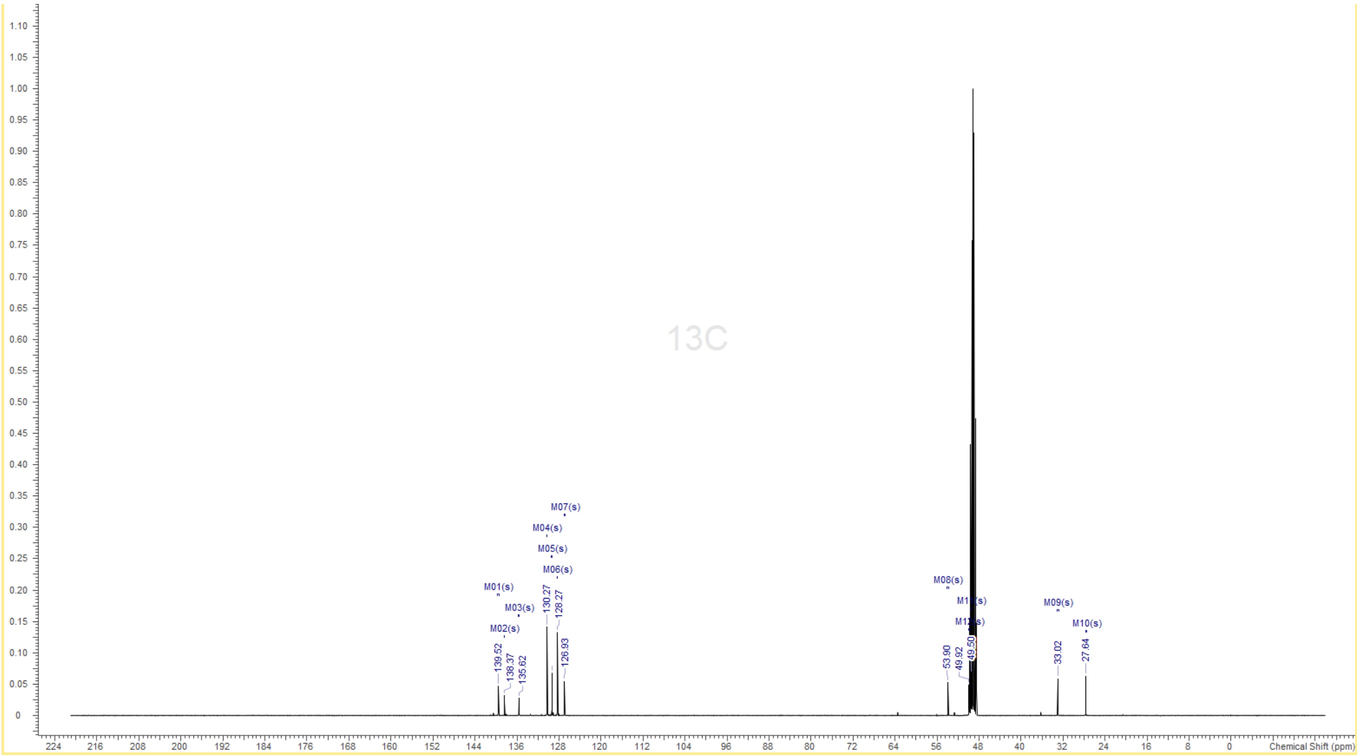

Zoomed "methanol" region:

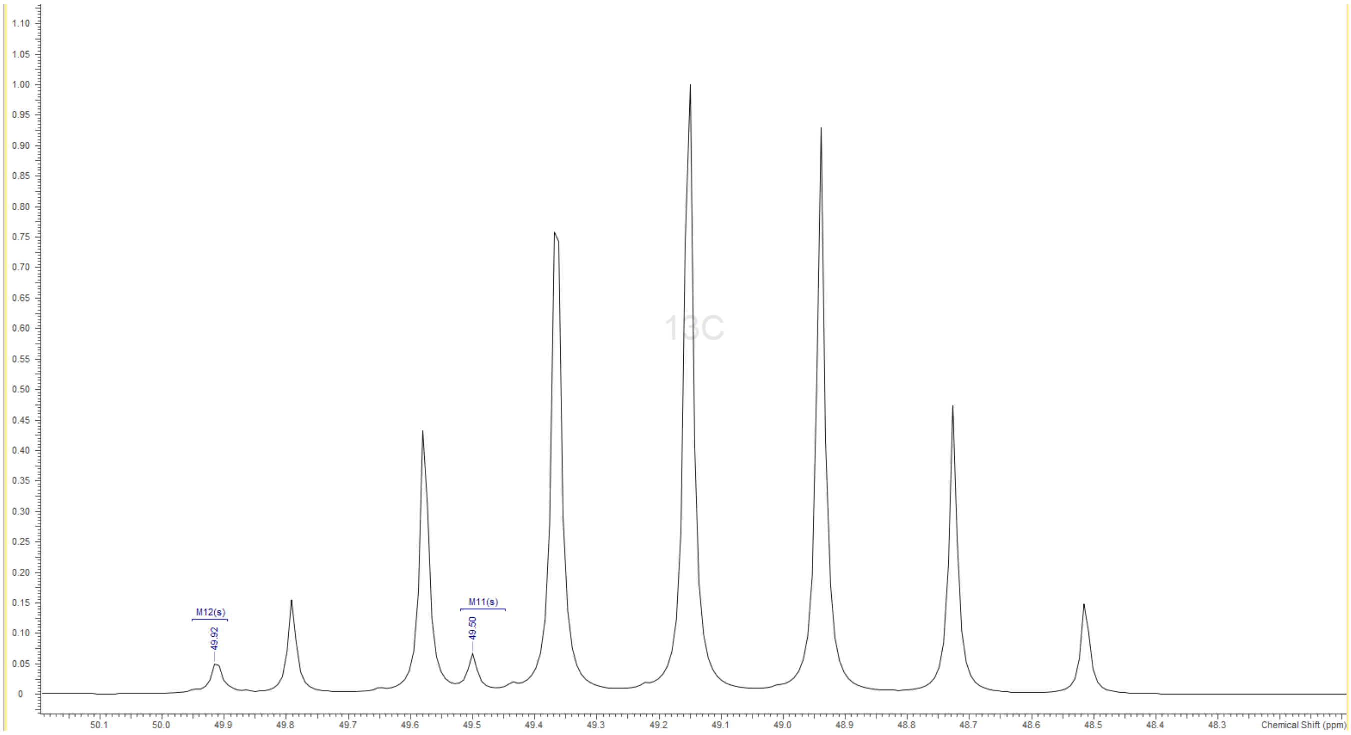

M12(s)
49.92
M11(s)
49.50
13C
Chemical Shift (ppm)

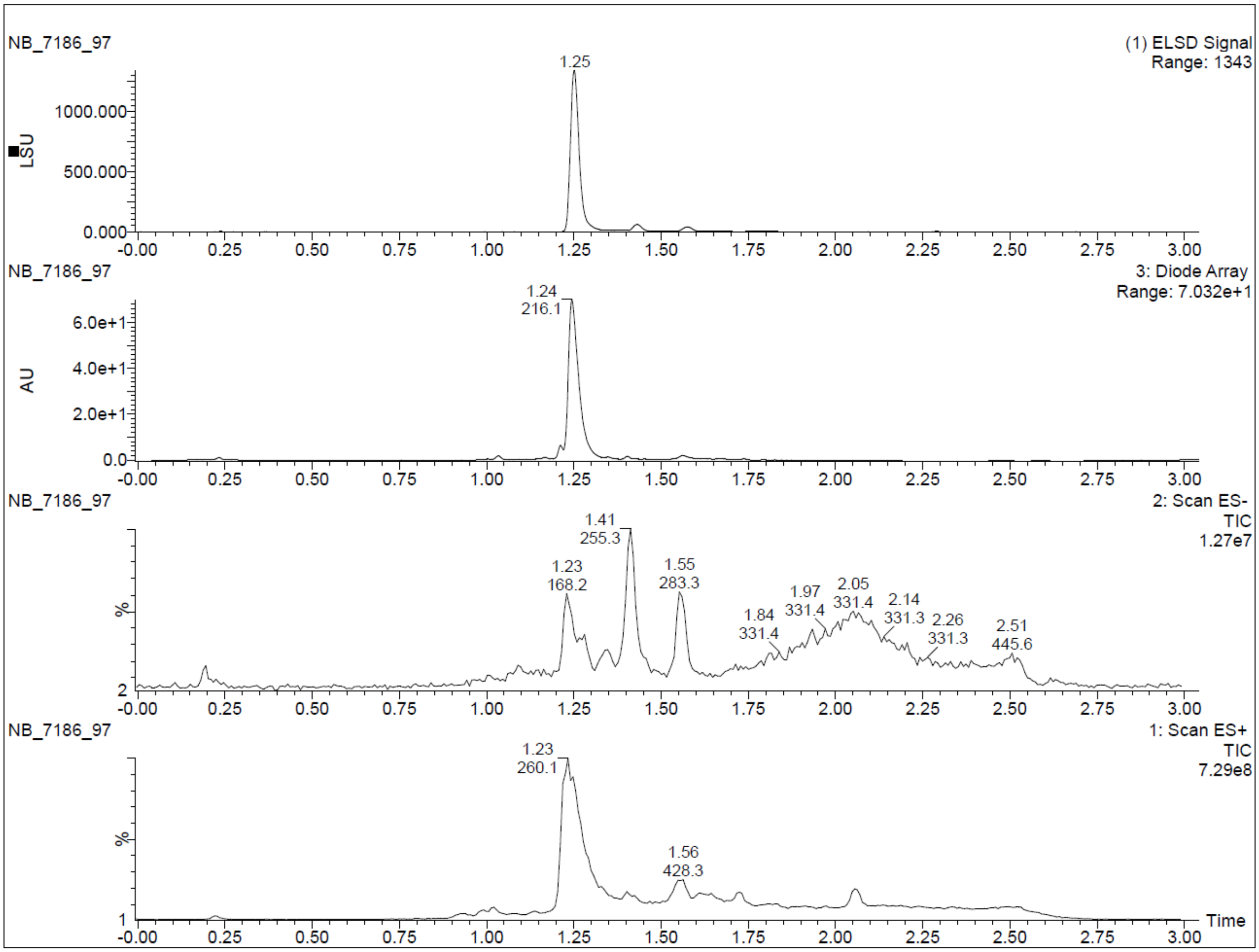

NB_7186_97
(1) ELSD Signal
Range: 1343
1.25
LSU
NB_7186_97
3: Diode Array
Range: 7.032e+1
1.24
216.1
AU
NB_7186_97
2: Scan ES-
TIC
1.27e7
1.41
255.3
1.23
168.2
1.55
283.3
1.84
331.4
1.97
331.4
2.05
331.4
2.14
331.3
2.26
331.3
2.51
445.6
%
NB_7186_97
1: Scan ES+
TIC
7.29e8
1.23
260.1
1.56
428.3
%
Time

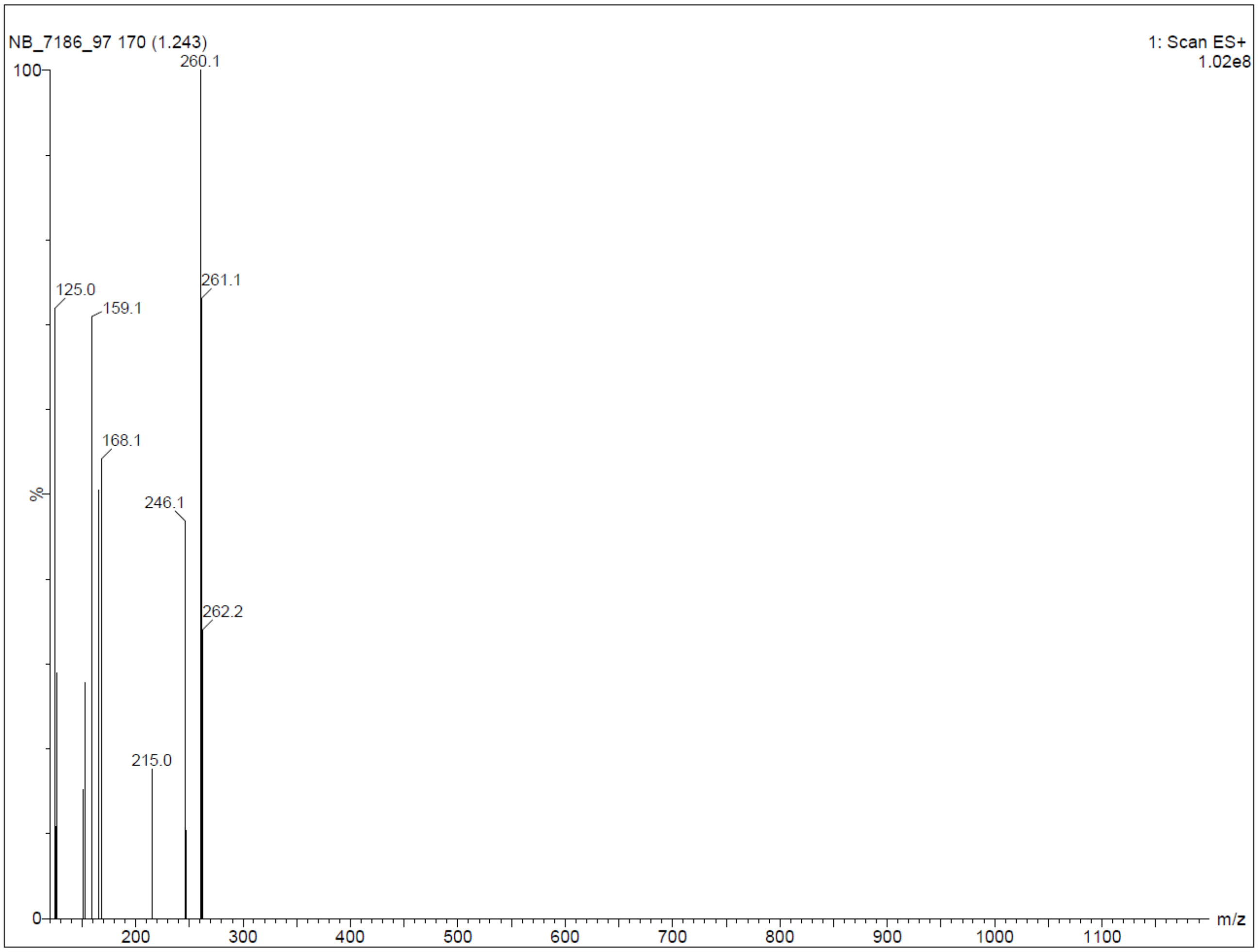

3-(Pyrazolo[1,5-a]pyridin-7-yl)thiomorpholine, Free base (4jy)

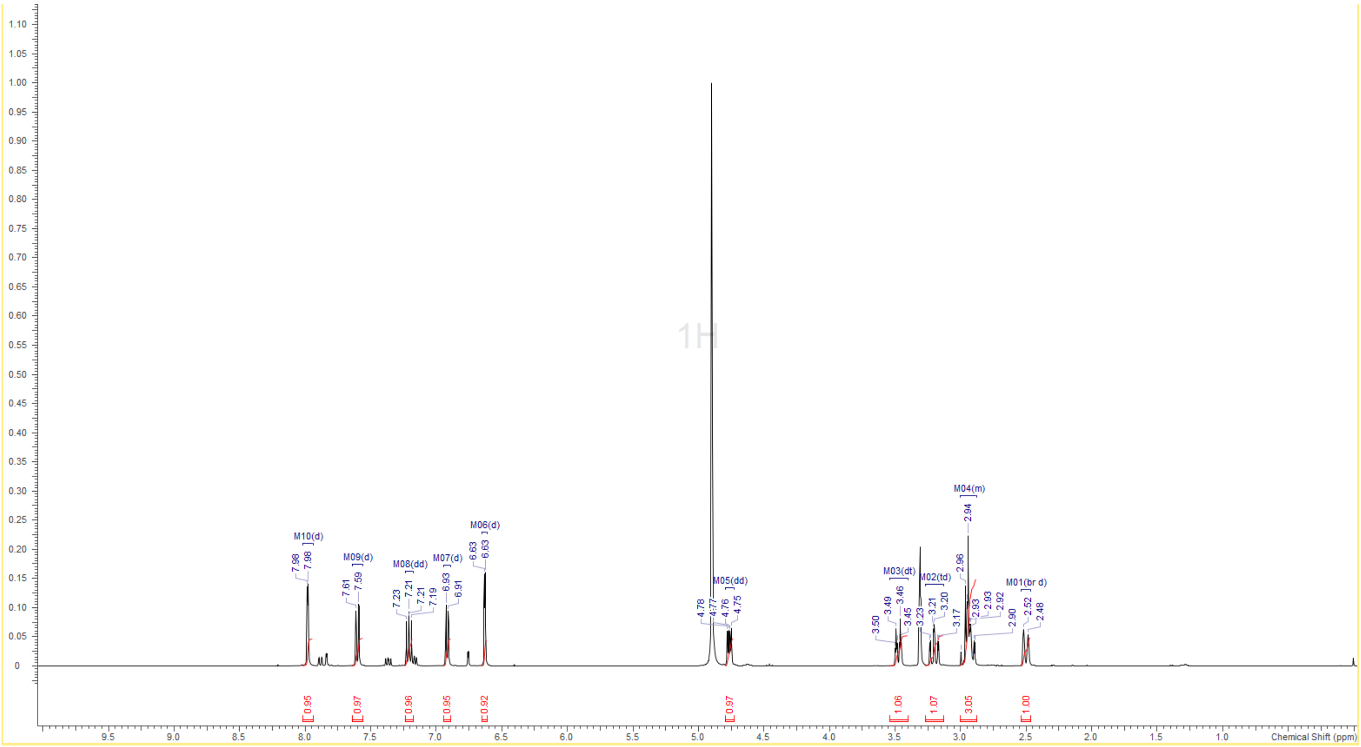

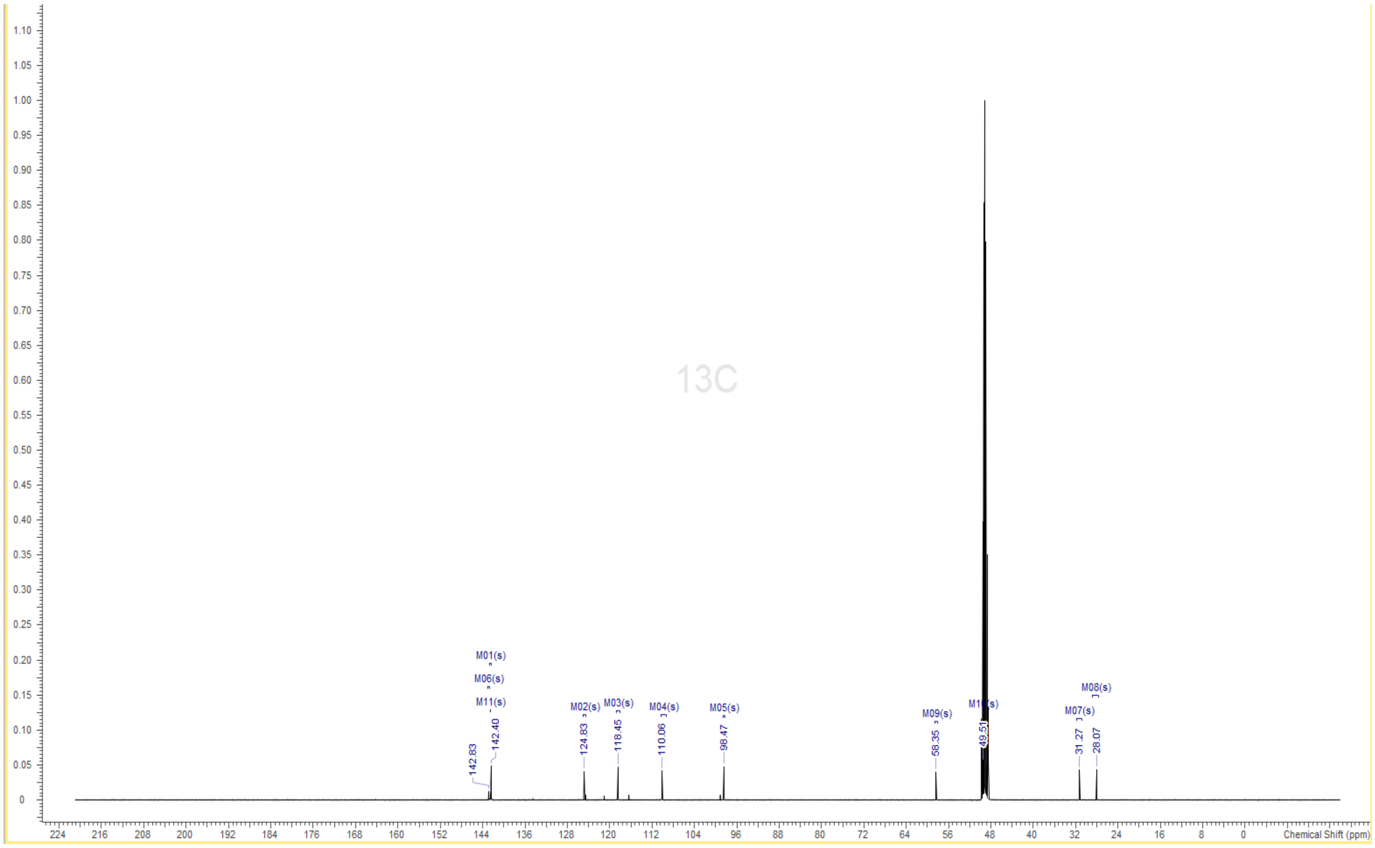

3-(4-Methoxyphenyl)-5-methylthiomorpholine, Free base (4ka)

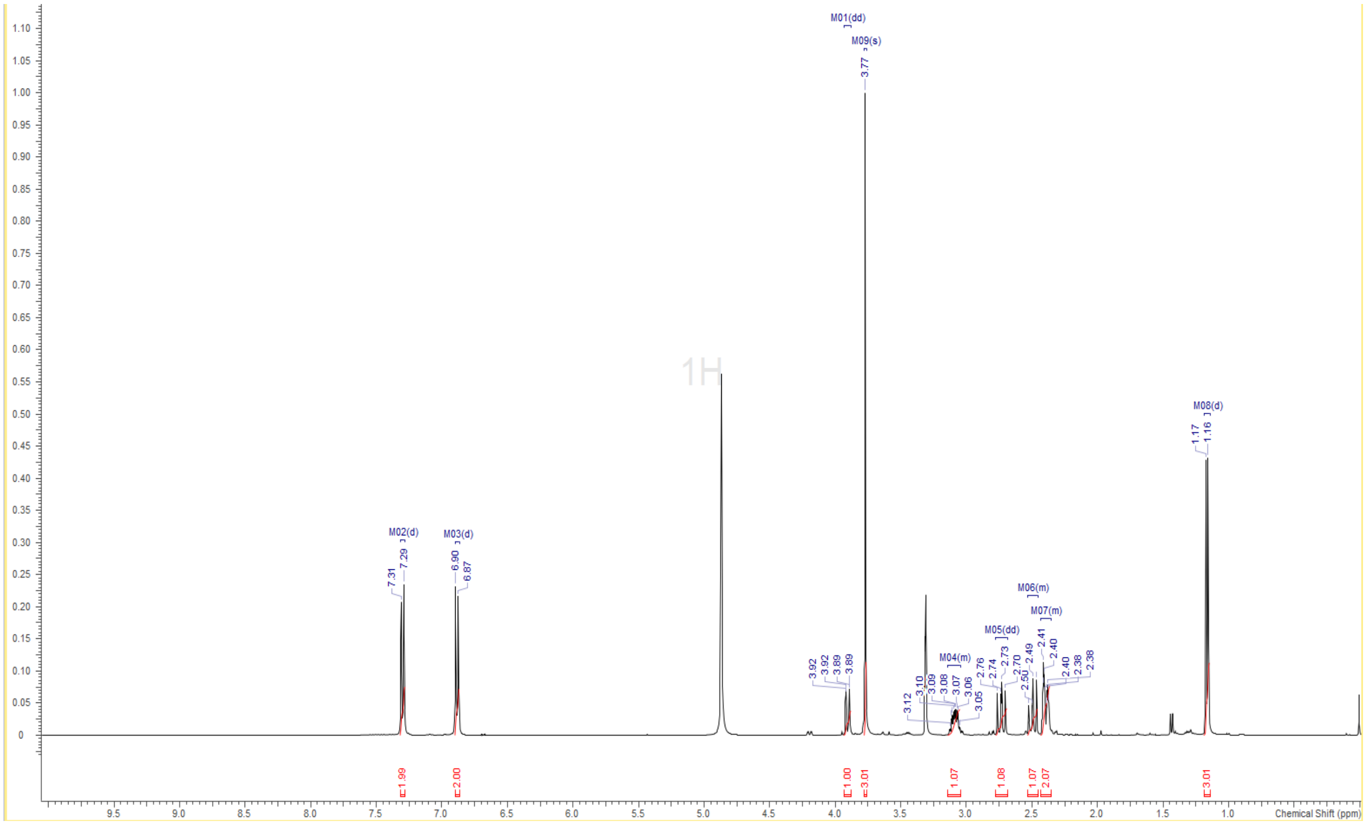

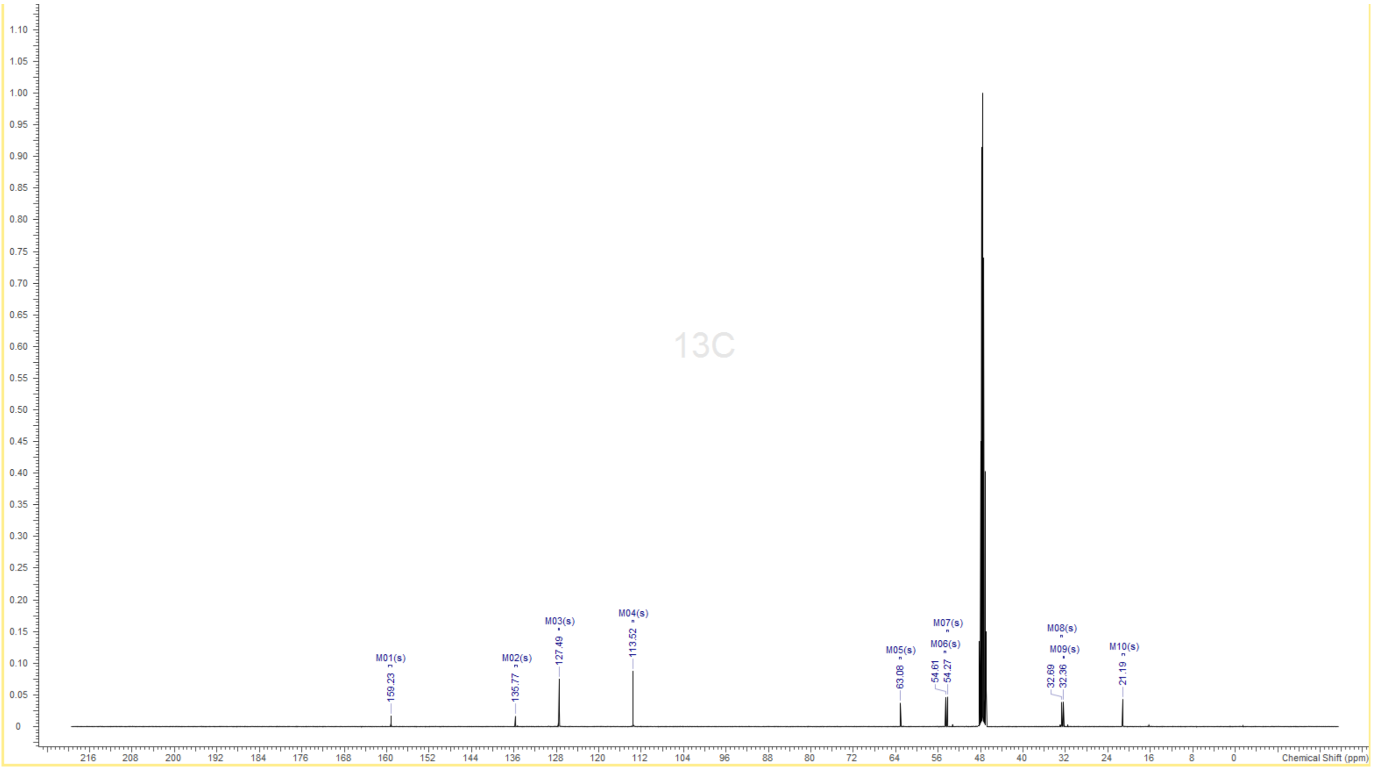
13C
M01(s)
159.23
M02(s)
135.77
M03(s)
127.49
M04(s)
113.52
M05(s)
63.08
M06(s)
54.61
54.27
M07(s)
M08(s)
M09(s)
32.69
32.36
M10(s)
21.19
Chemical Shift (ppm)

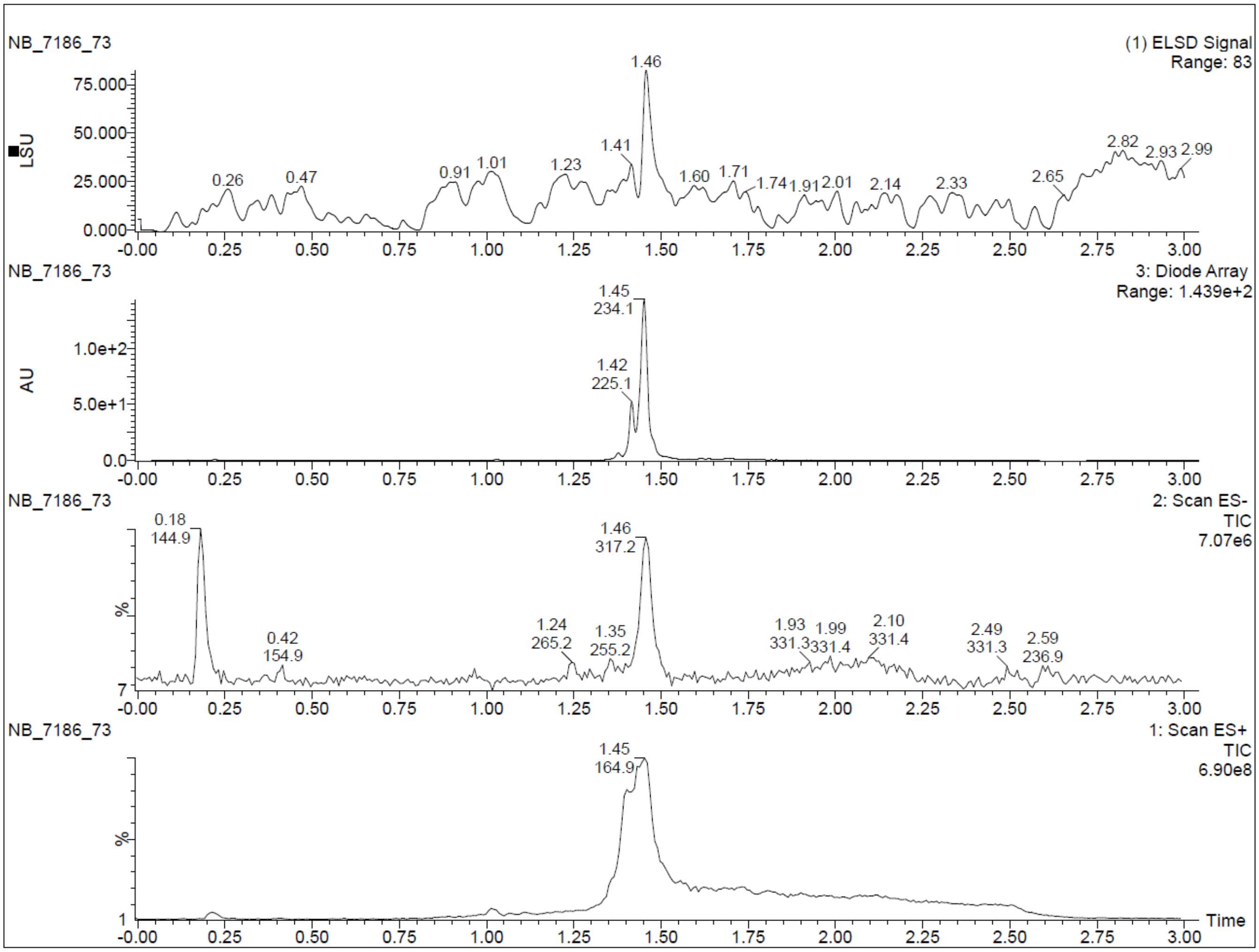
NB_7186_73
(1) ELSD Signal
Range: 83
LSU
NB_7186_73
3: Diode Array
Range: 1.439e+2
AU
NB_7186_73
2: Scan ES-
TIC
7.07e6
NB_7186_73
1: Scan ES+
TIC
6.90e8
Time

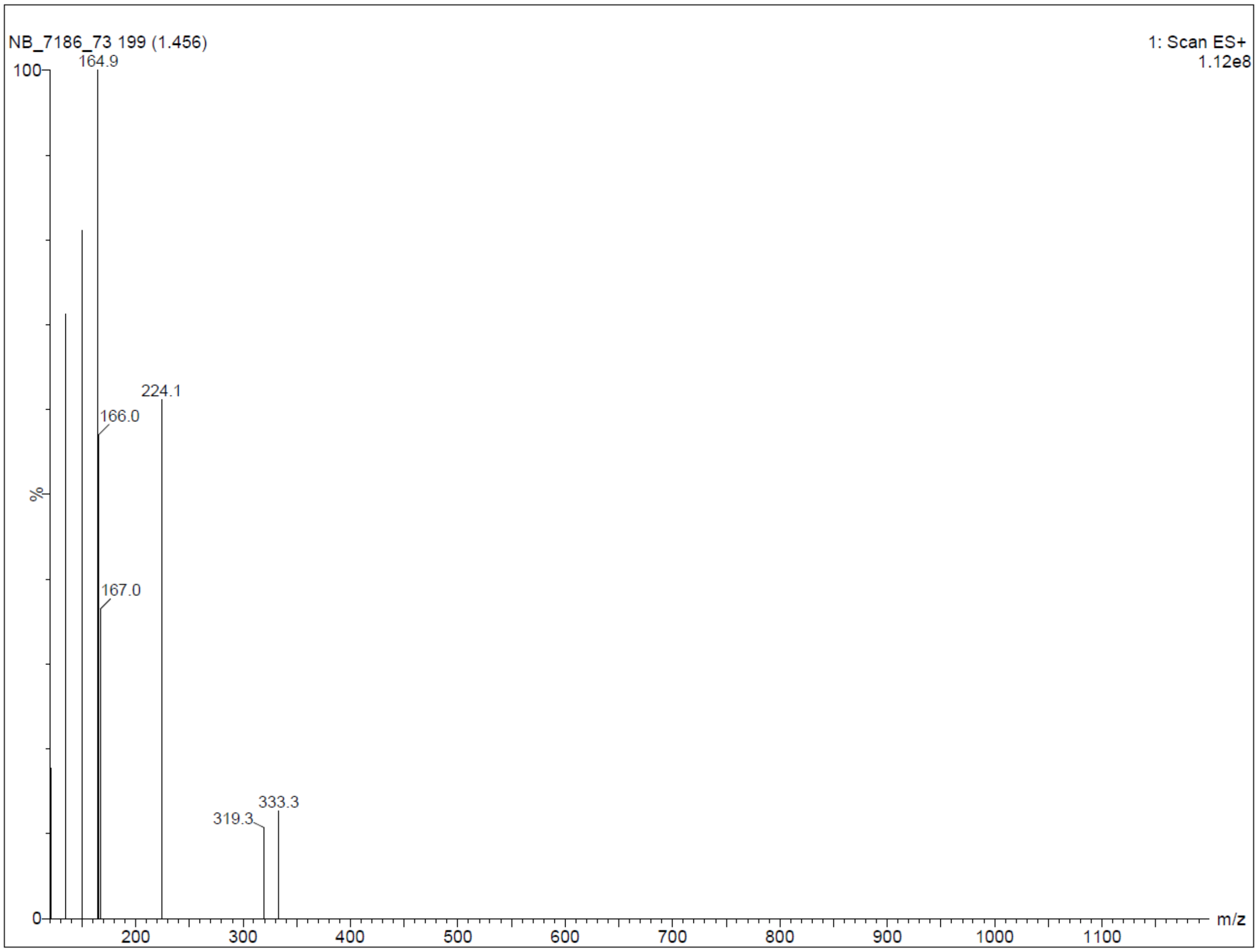

(3S,5R)-3-Methyl-5-(1-methyl-1H-pyrazol-5-yl)thiomorpholine, Free base (4kp)

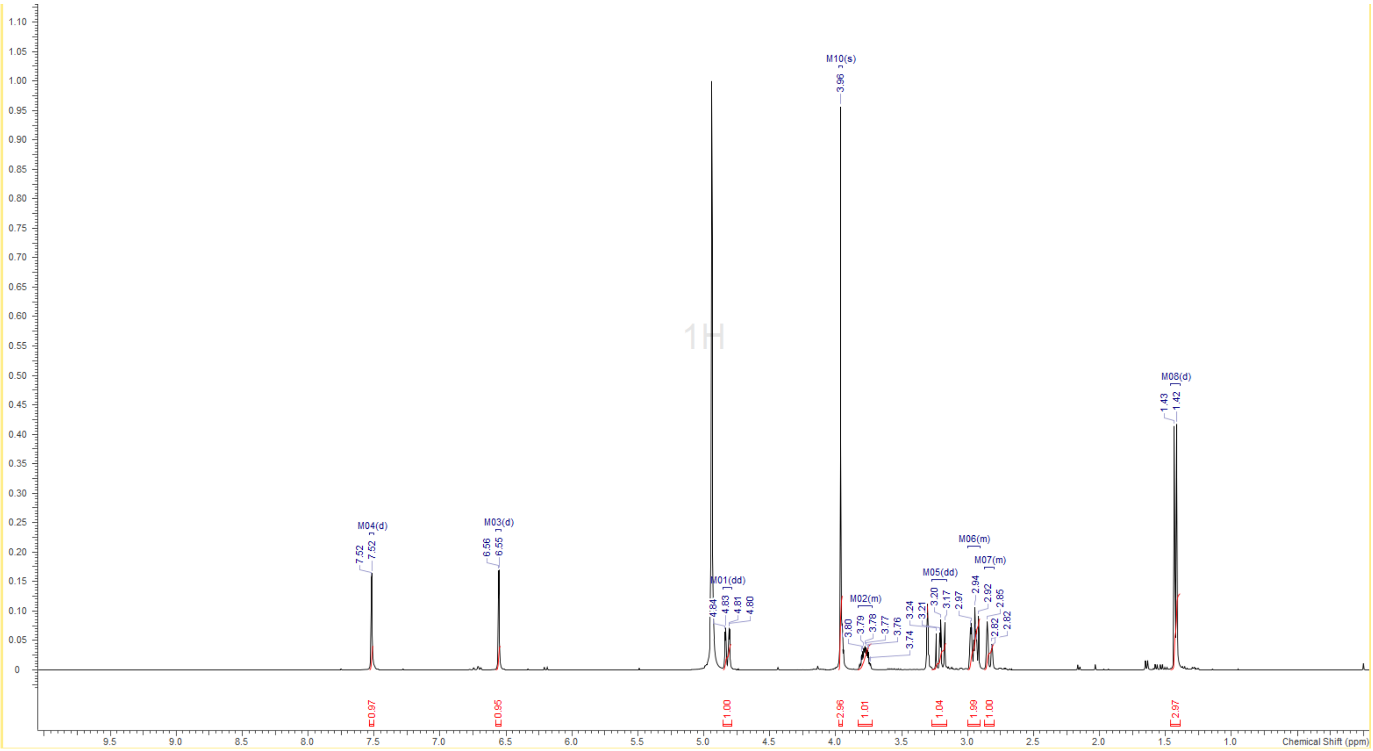

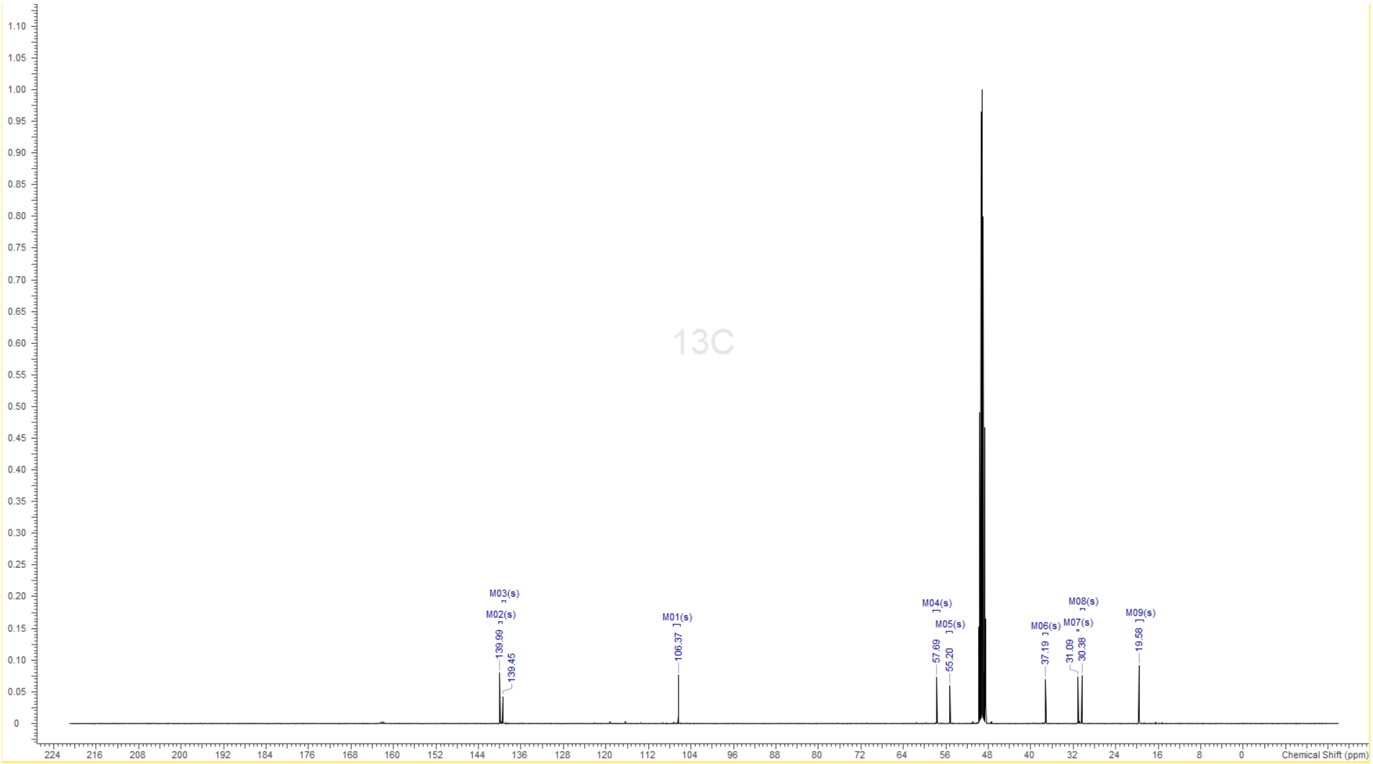

13C
M01(s)
M02(s)
M03(s)
M04(s)
M05(s)
M06(s)
M07(s)
M08(s)
M09(s)
139.99
139.45
106.37
57.69
55.20
37.19
31.09
30.38
19.58
Chemical Shift (ppm)

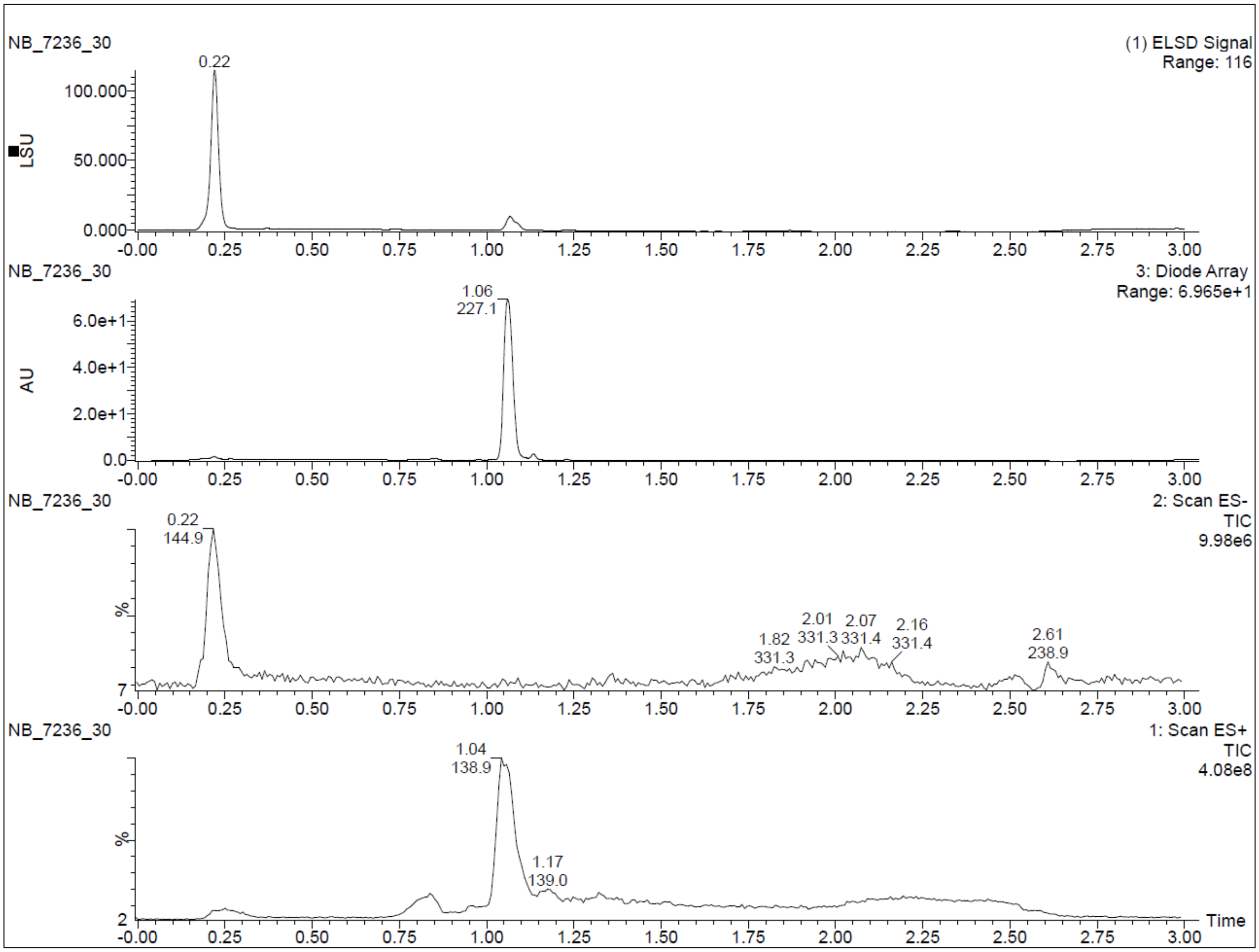

NB_7236_30
(1) ELSD Signal
Range: 116
LSU
0.22
NB_7236_30
3: Diode Array
Range: 6.965e+1
AU
1.06
227.1
NB_7236_30
2: Scan ES-
TIC
9.98e6
0.22
144.9
1.82
331.3
2.01
331.3
2.07
331.4
2.16
331.4
2.61
238.9
NB_7236_30
1: Scan ES+
TIC
4.08e8
1.04
138.9
1.17
139.0
Time

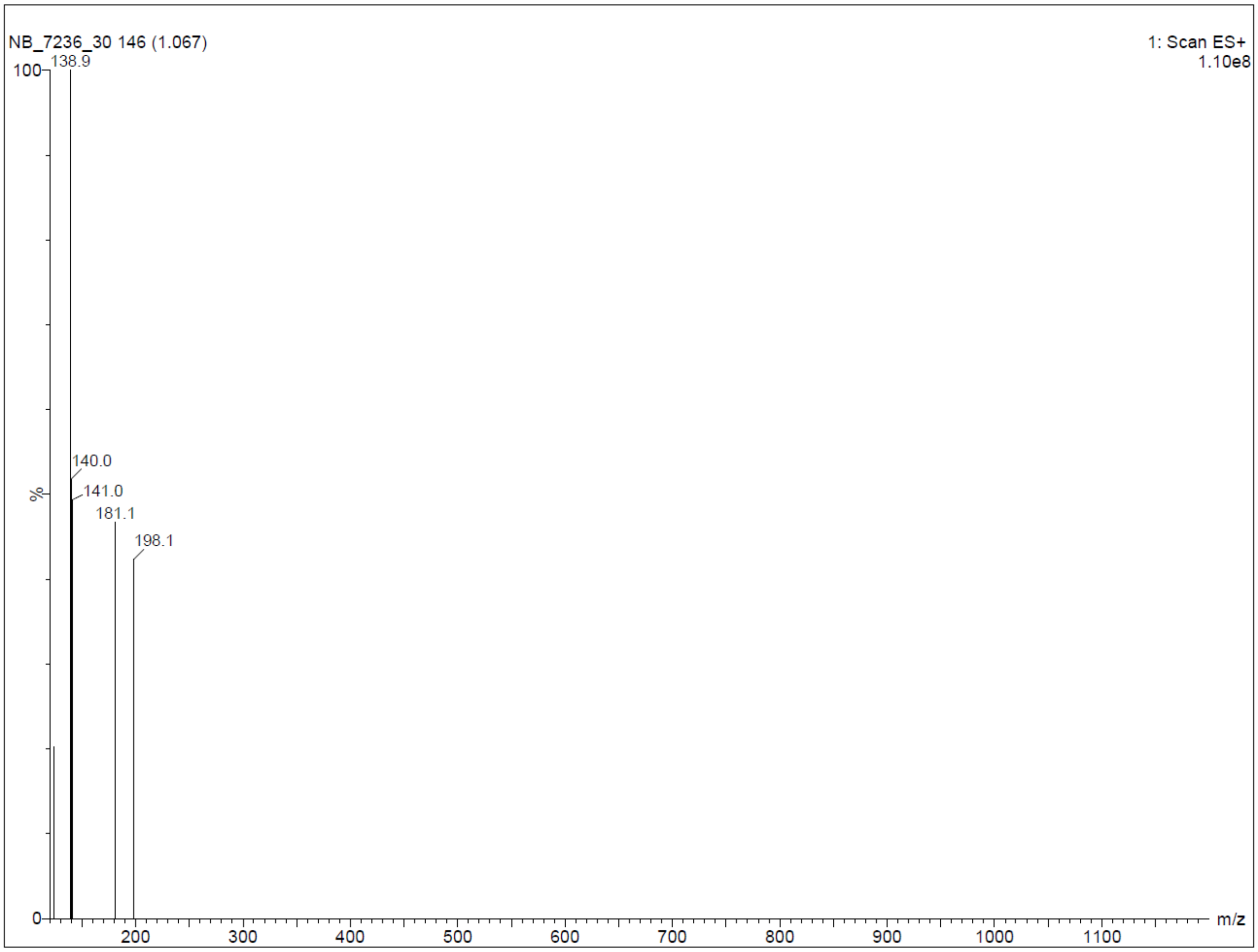

5-(4-Methoxyphenyl)-2-methylthiomorpholine, Free base (4la)

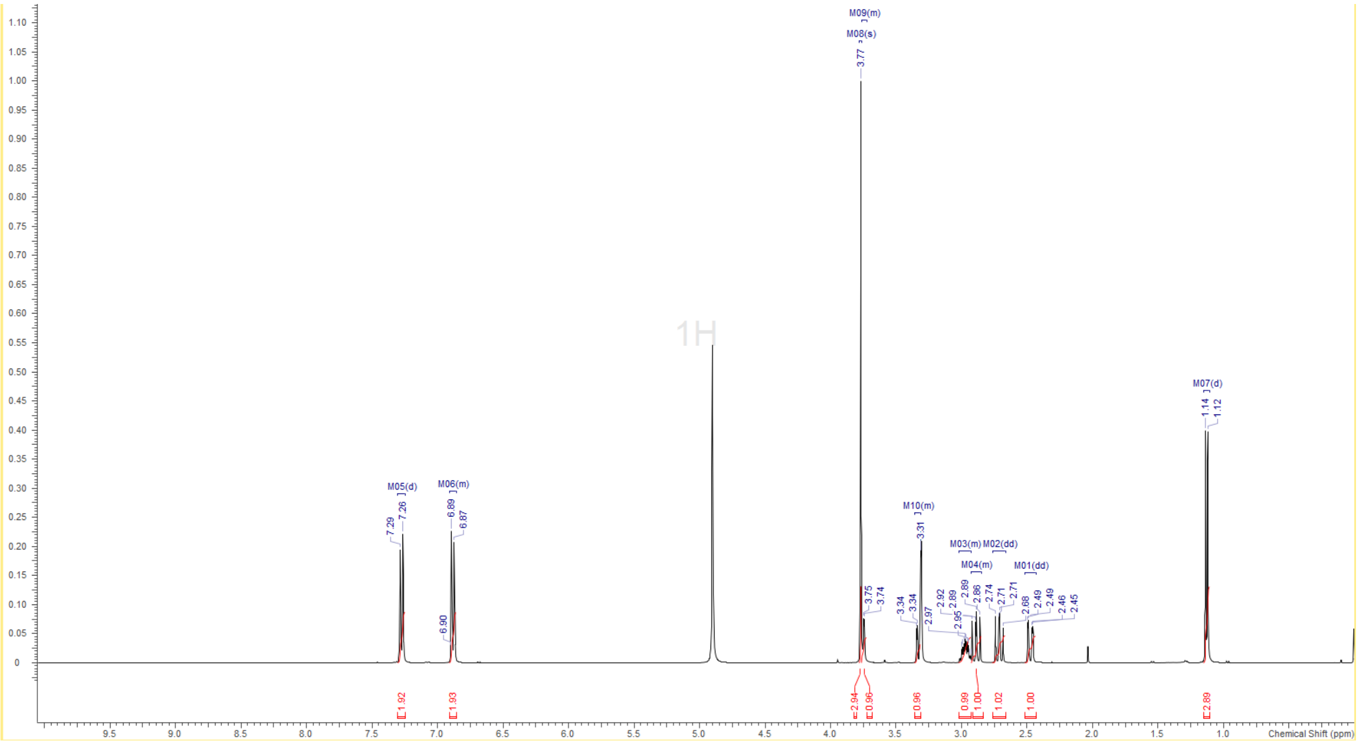

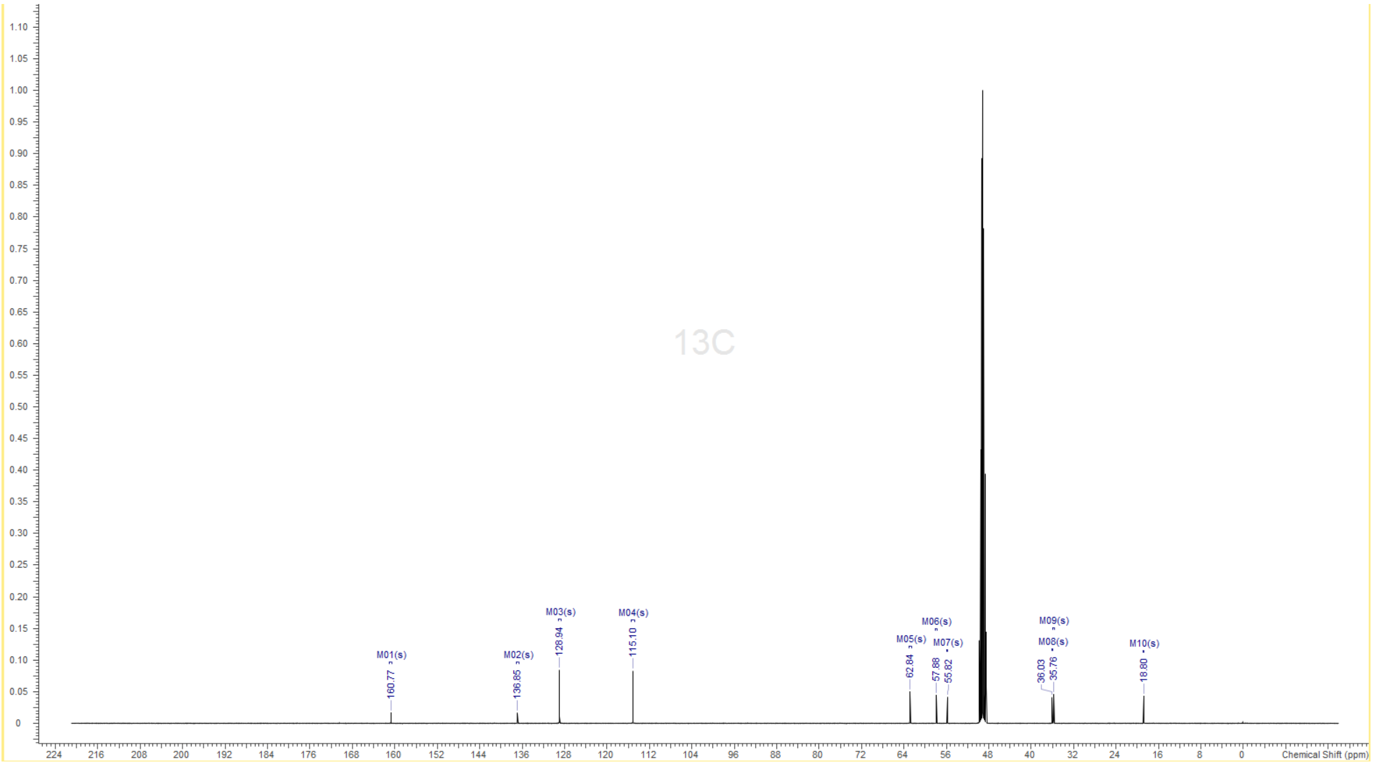
13C
M01(s) 160.77
M02(s) 136.85
M03(s) 128.94
M04(s) 115.10
M05(s) 62.84
M06(s) 57.88
M07(s) 55.82
M08(s) 35.76
M09(s) 36.03
M10(s) 18.80
Chemical Shift (ppm)

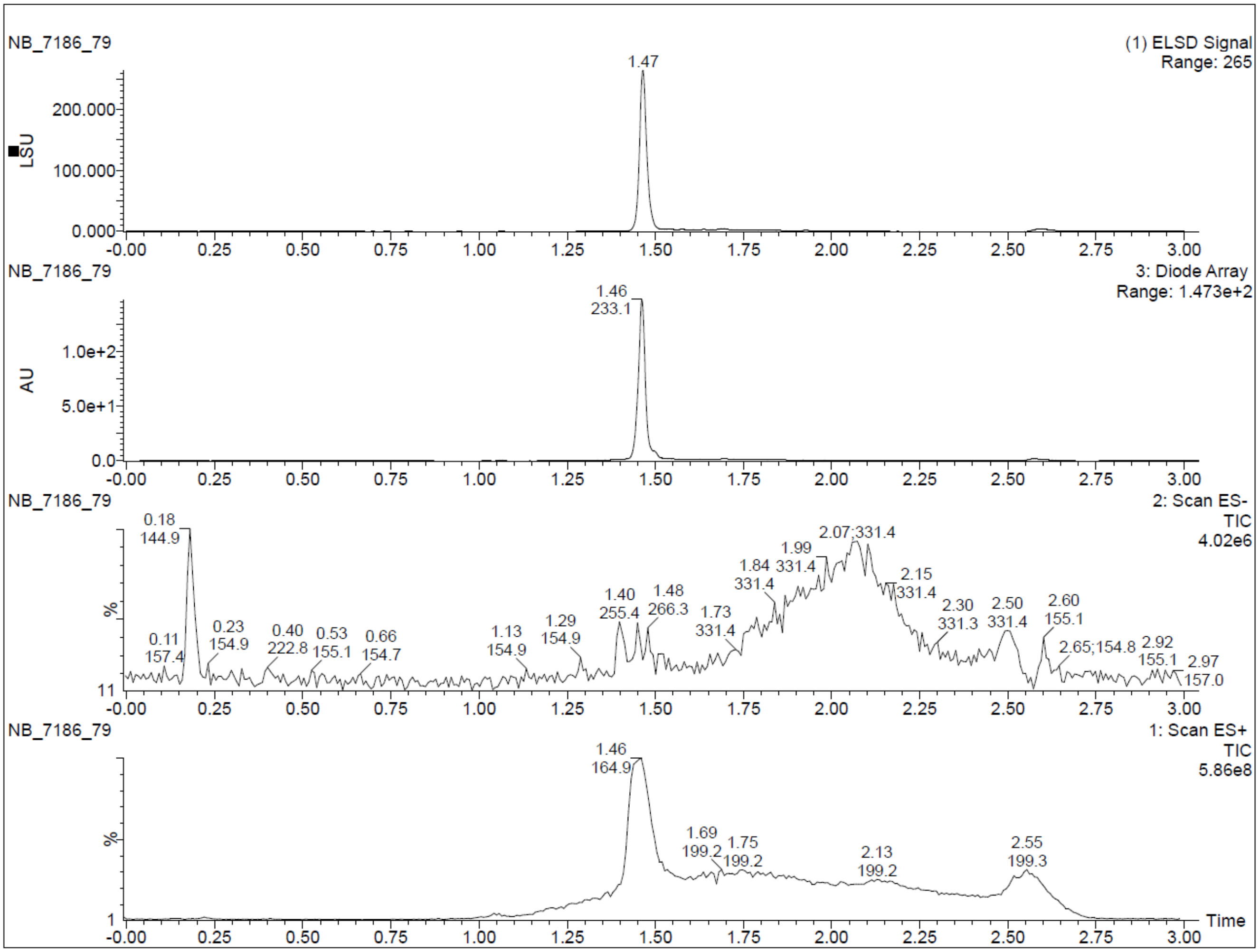
NB_7186_79
(1) ELSD Signal
Range: 265
LSU
1.47
NB_7186_79
3: Diode Array
Range: 1.473e+2
AU
1.46
233.1
NB_7186_79
2: Scan ES-
TIC
4.02e6
NB_7186_79
1: Scan ES+
TIC
5.86e8
1.46
164.9
Time

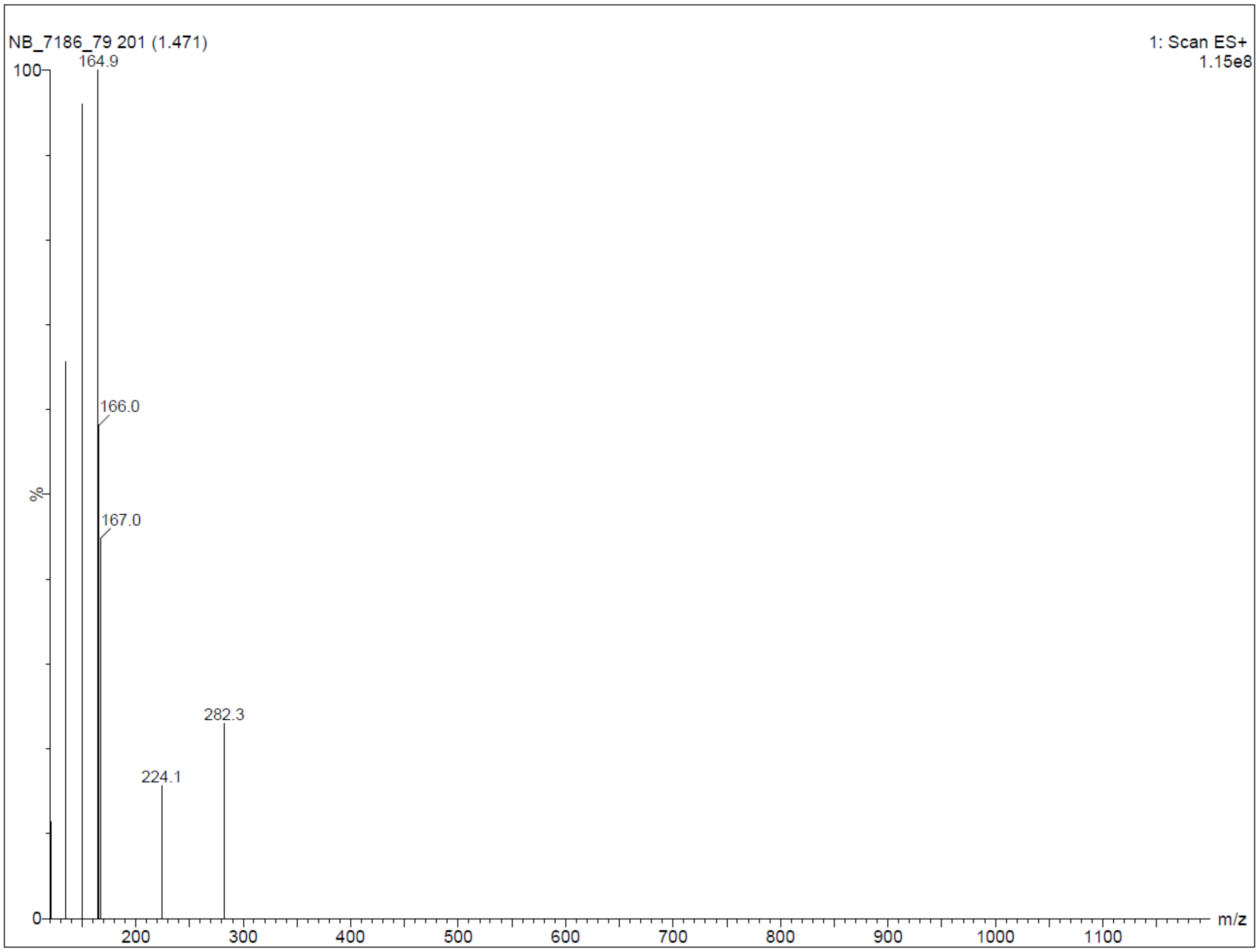

Benzyl 3-(4-methoxyphenyl)piperazine-1-carboxylate, Free base (4ma)

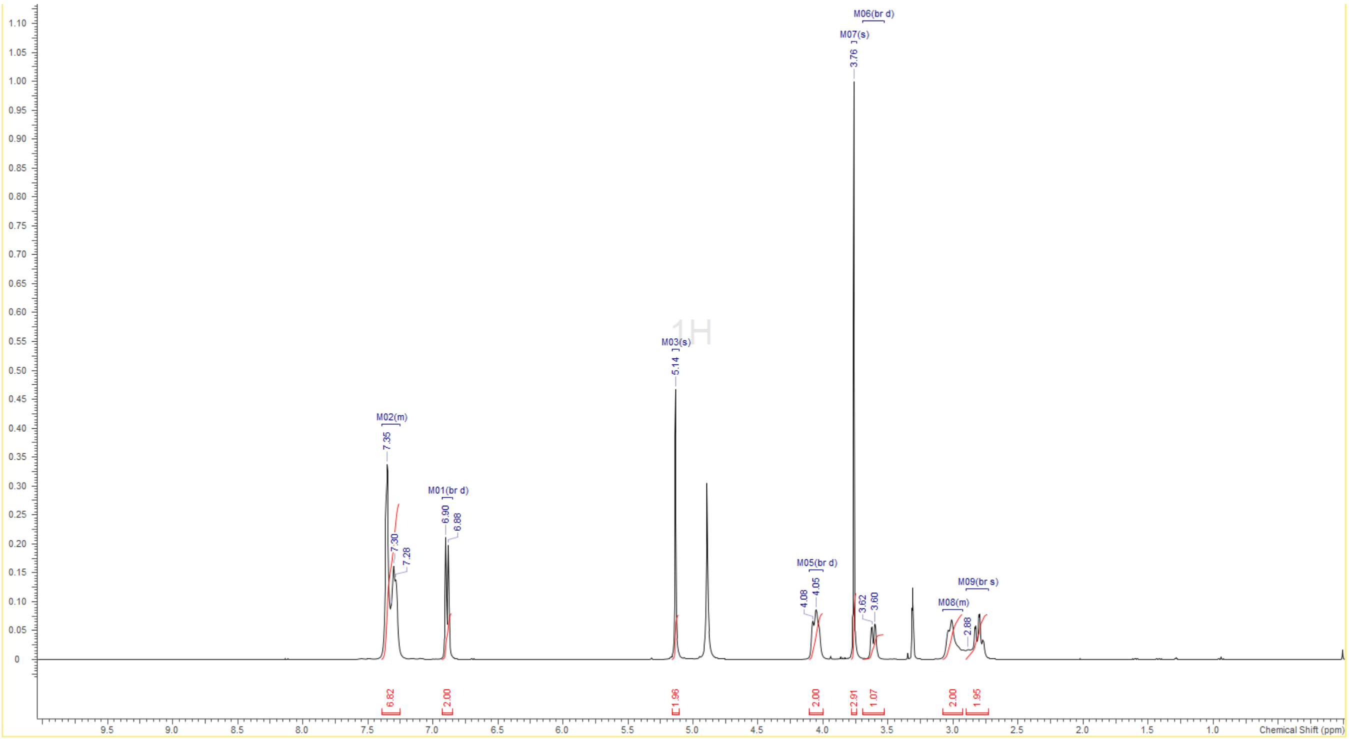

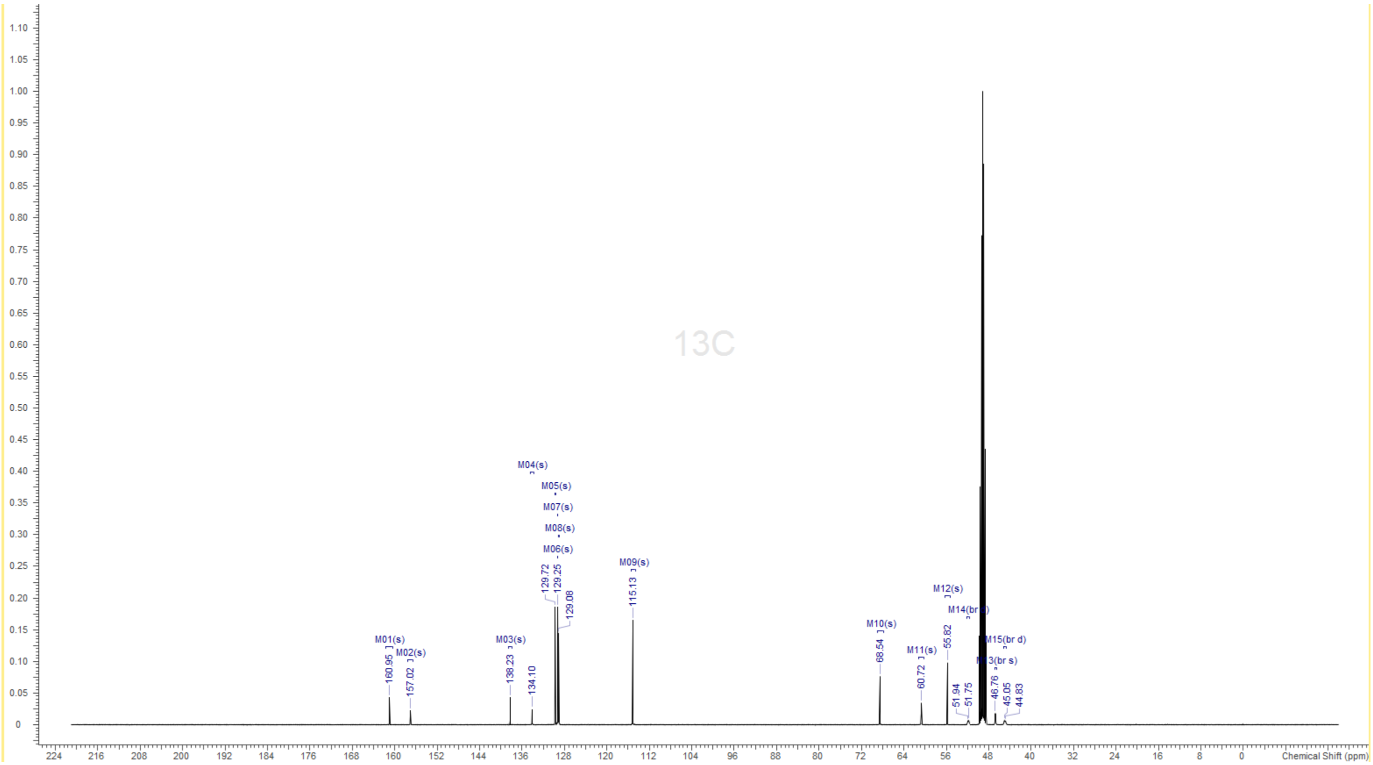
13C
M01(s)
160.95
M02(s)
157.02
M03(s)
138.23
134.10
M04(s)
M05(s)
M07(s)
M08(s)
M06(s)
129.72
129.25
129.08
M09(s)
115.13
M10(s)
68.54
M11(s)
60.72
M12(s)
55.82
51.94
51.75
M15(br d)
46.76
45.05
44.83
Chemical Shift (ppm)

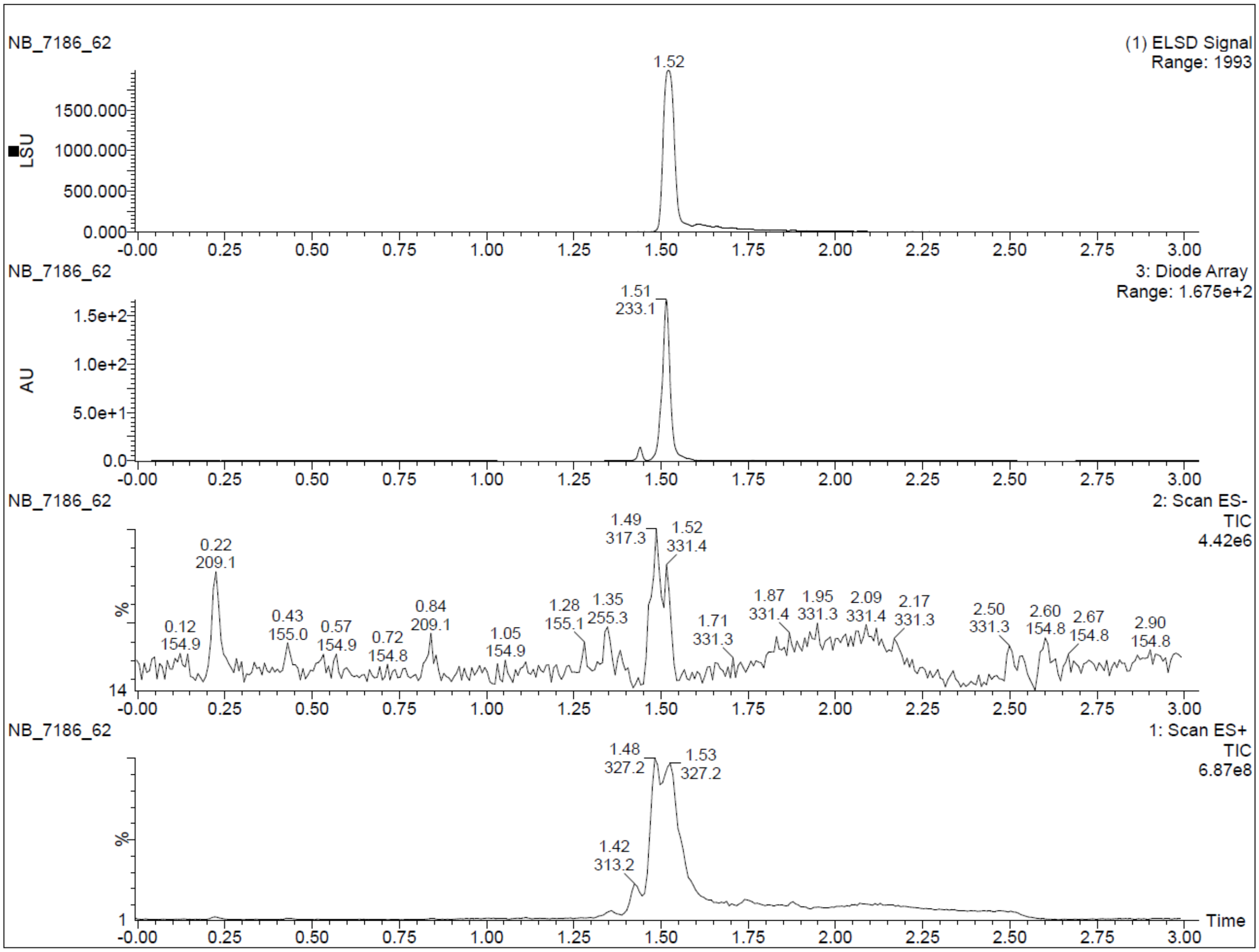
NB_7186_62
(1) ELSD Signal
Range: 1993
1.52
LSU
NB_7186_62
3: Diode Array
Range: 1.675e+2
1.51
233.1
AU
NB_7186_62
2: Scan ES-
TIC
4.42e6
0.22 209.1
0.12 154.9
0.43 155.0
0.57 154.9
0.72 154.8
0.84 209.1
1.05 154.9
1.28 155.1
1.35 255.3
1.49 317.3
1.52 331.4
1.71 331.3
1.87 331.4
1.95 331.3
2.09 331.4
2.17 331.3
2.50 331.3
2.60 154.8
2.67 154.8
2.90 154.8
NB_7186_62
1: Scan ES+
TIC
6.87e8
1.48 327.2
1.53 327.2
1.42 313.2
Time

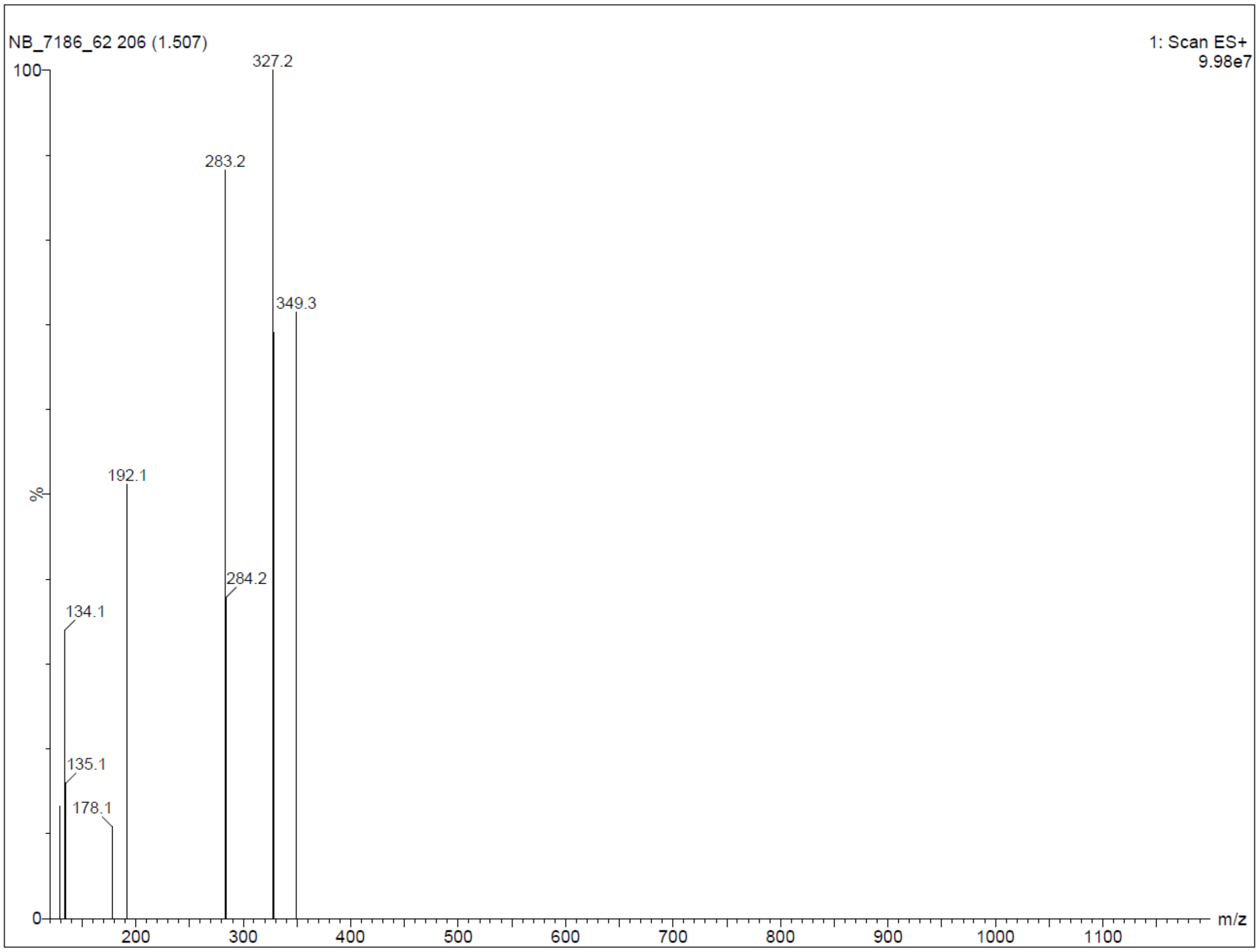

3-(4-Methoxyphenyl)-1-tosylpiperazine, Free base (4na)

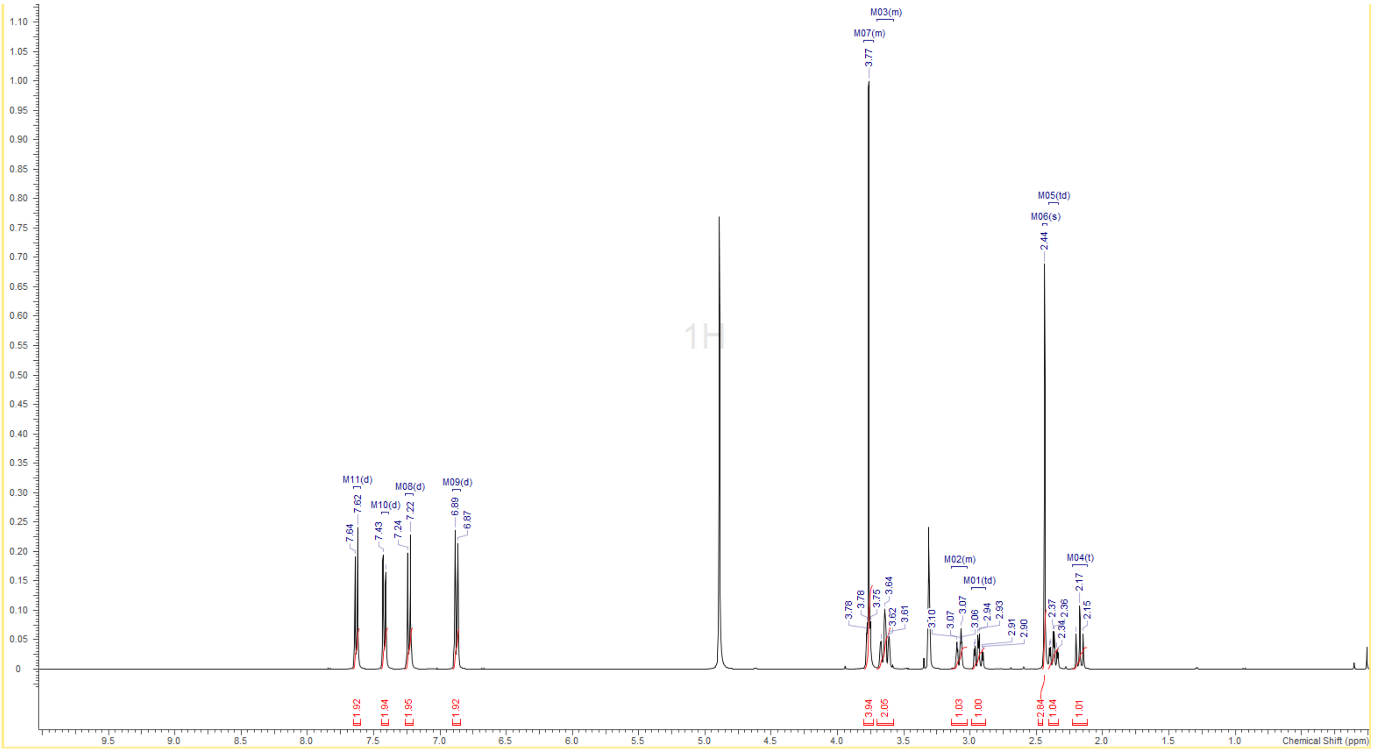

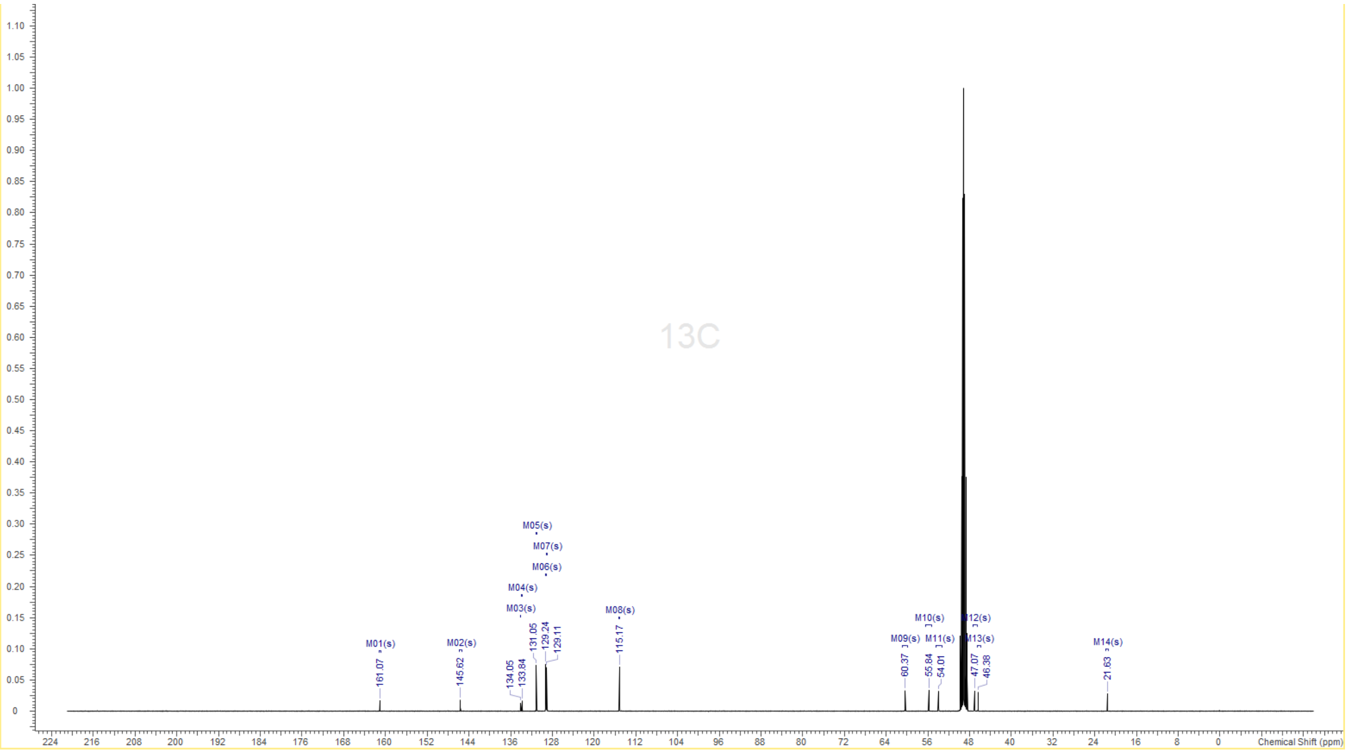
13C
M01(s)
161.07
M02(s)
145.62
M03(s)
M04(s)
134.05
133.84
M05(s)
131.05
M07(s)
129.24
M06(s)
129.11
M08(s)
115.17
M09(s)
60.37
M10(s)
55.84
M11(s)
54.01
M12(s)
M13(s)
47.07
46.38
M14(s)
21.63
Chemical Shift (ppm)

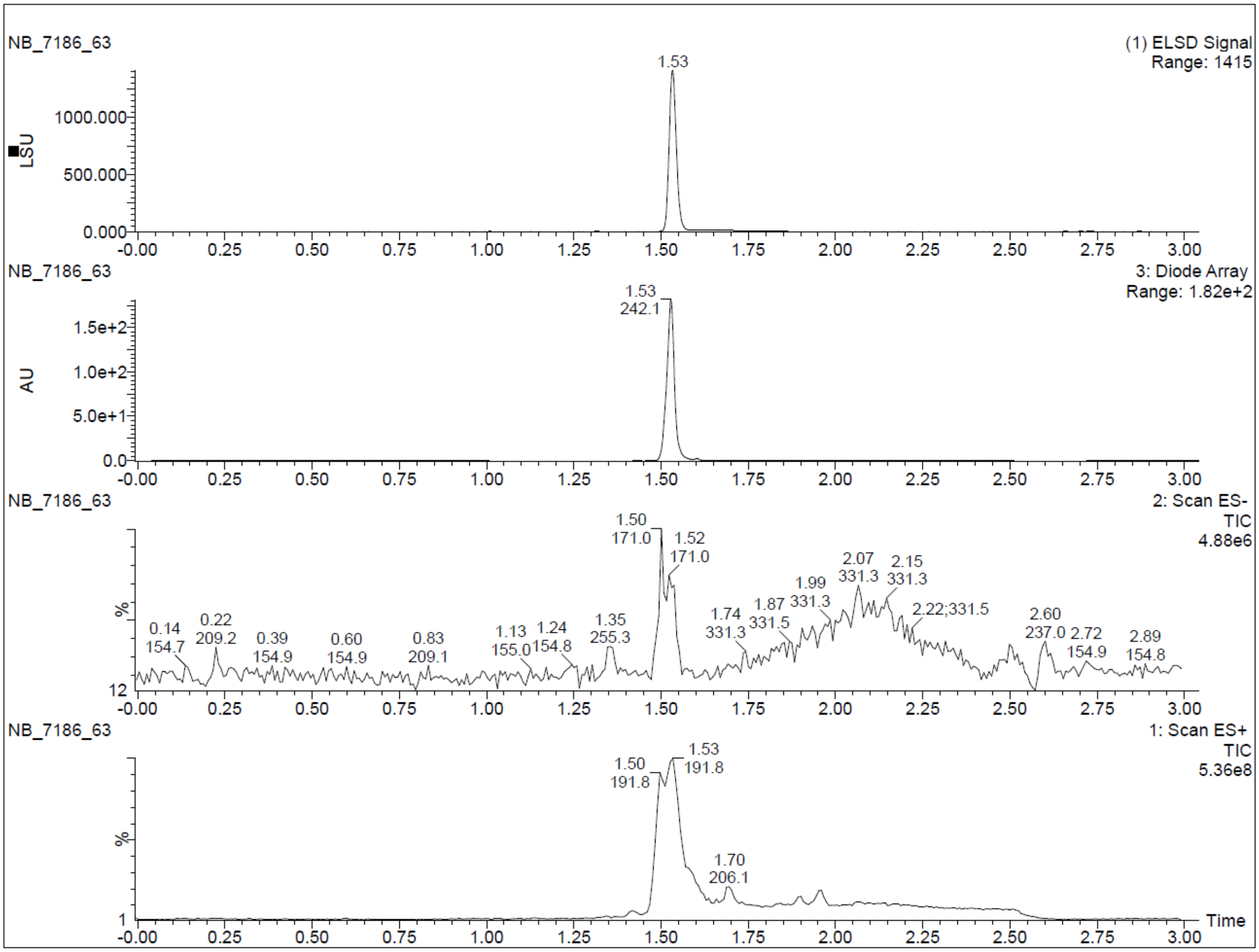
NB_7186_63
(1) ELSD Signal
Range: 1415
1.53
LSU
1000.000
500.000
0.000
NB_7186_63
3: Diode Array
Range: 1.82e+2
1.53
242.1
AU
1.5e+2
1.0e+2
5.0e+1
0.0
NB_7186_63
2: Scan ES-
TIC
4.88e6
1.50
171.0
1.52
171.0
0.14
154.7
0.22
209.2
0.39
154.9
0.60
154.9
0.83
209.1
1.13
155.0
1.24
154.8
1.35
255.3
1.74
331.3
1.87
331.5
1.99
331.3
2.07
331.3
2.15
331.3
2.22;331.5
2.60
237.0
2.72
154.9
2.89
154.8
%
12
NB_7186_63
1: Scan ES+
TIC
5.36e8
1.50
191.8
1.53
191.8
1.70
206.1
%
1
Time
-0.00
0.25
0.50
0.75
1.00
1.25
1.50
1.75
2.00
2.25
2.50
2.75
3.00

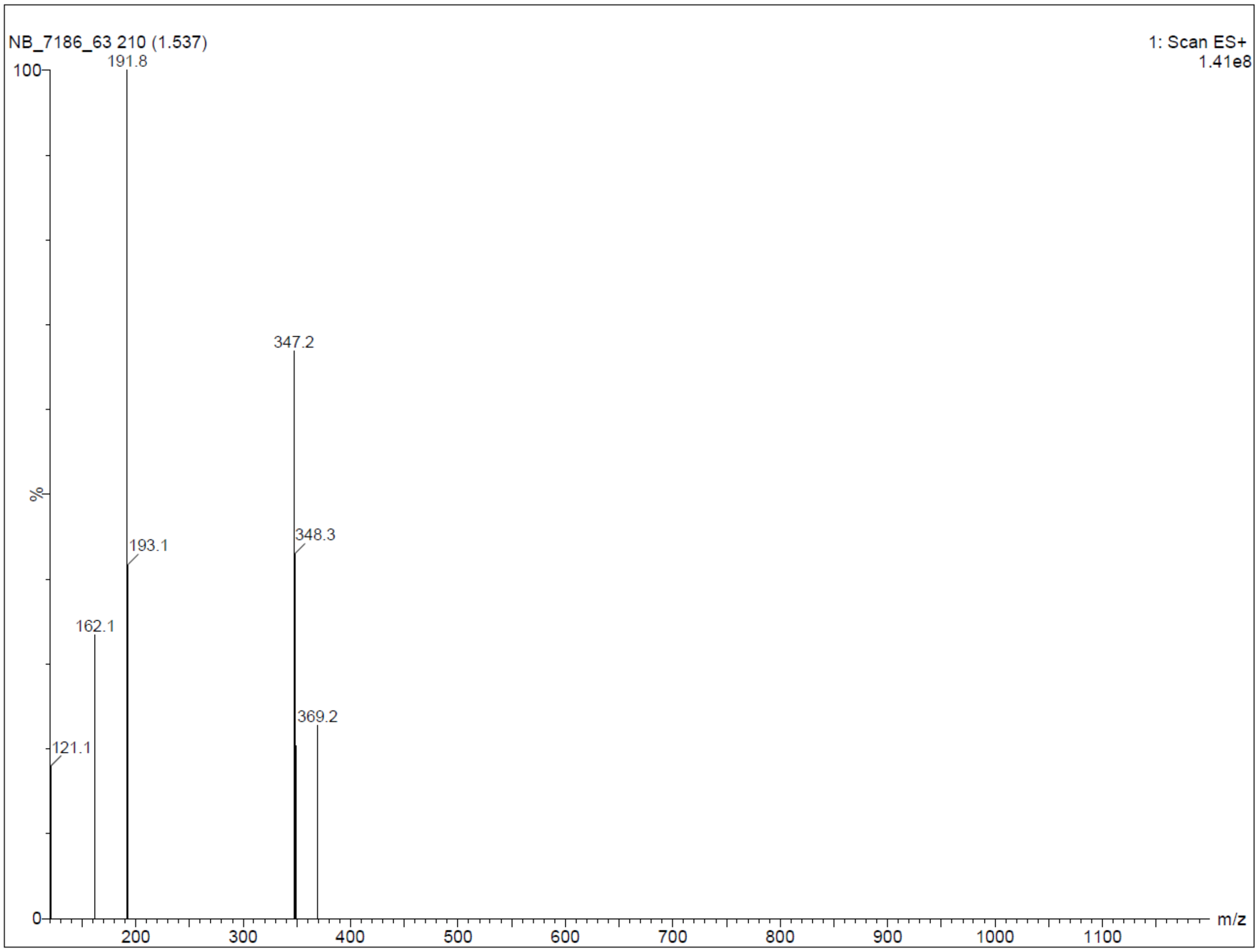

(5S)-3-(4-Methoxyphenyl)-5-methyl-1-tosylpiperazine, Free base (4oa)

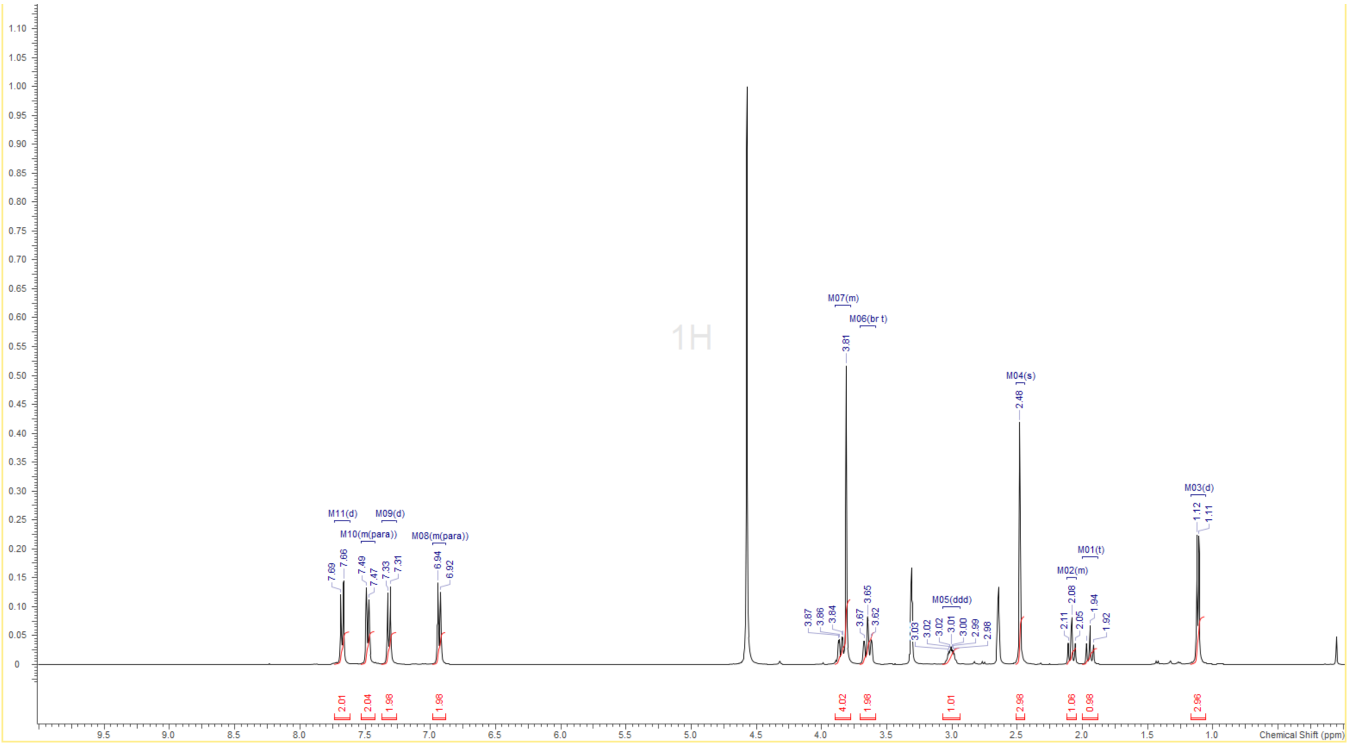

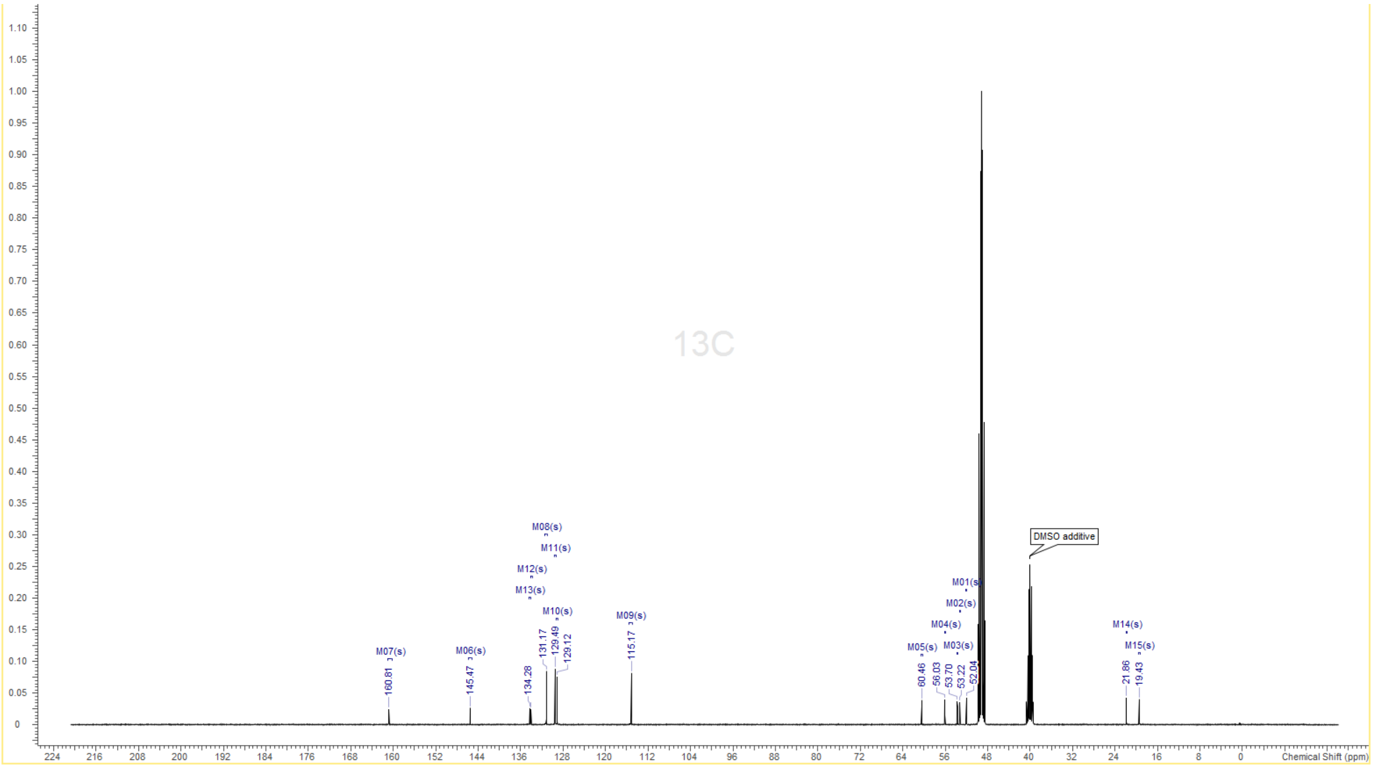
13C
DMSO additive
M07(s)
M06(s)
M08(s)
M11(s)
M12(s)
M13(s)
M10(s)
M09(s)
M01(s)
M02(s)
M04(s)
M05(s)
M03(s)
M14(s)
M15(s)
160.81
145.47
134.28
131.17
129.49
129.12
115.17
60.46
56.03
53.70
53.22
52.04
21.86
19.43
Chemical Shift (ppm)

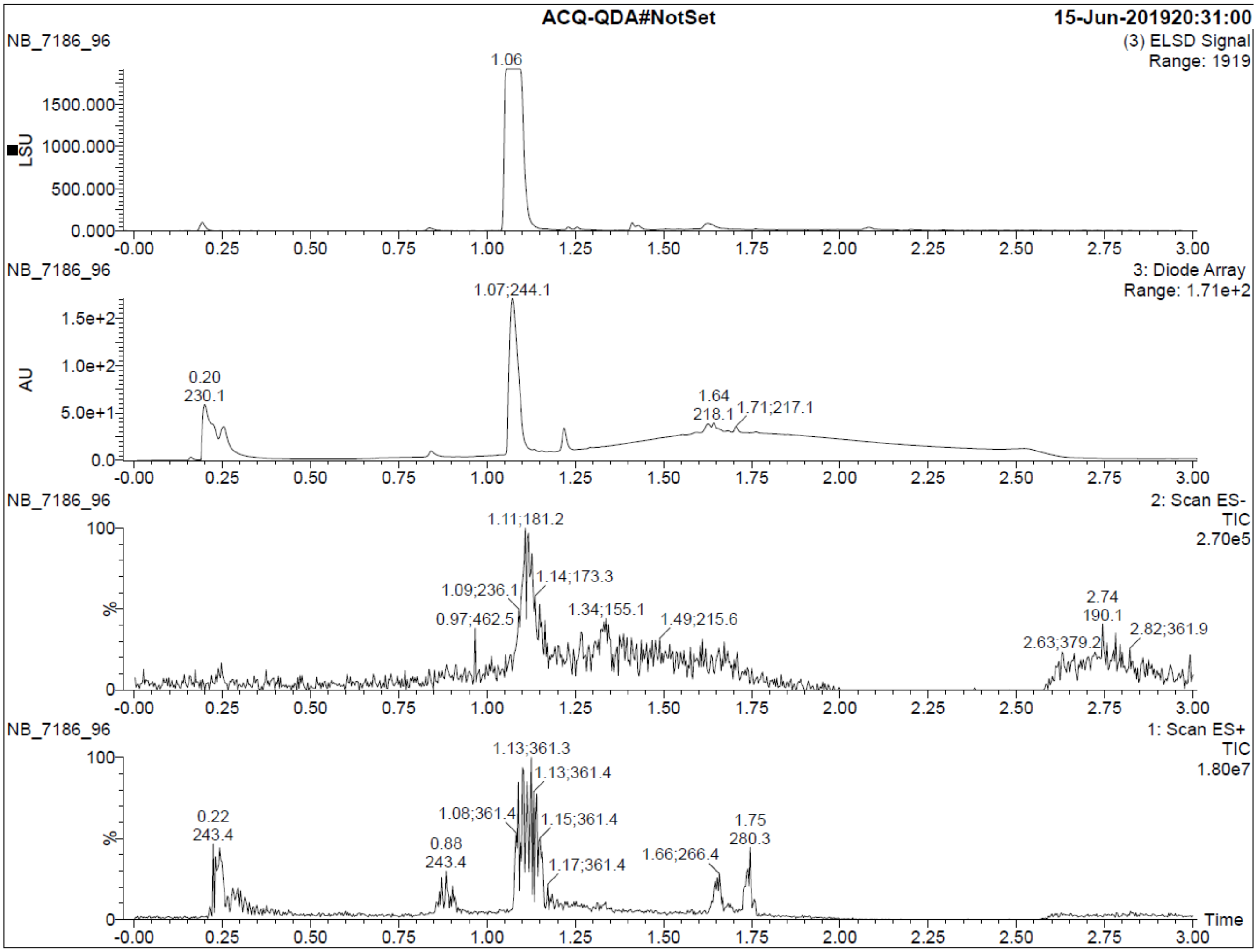
ACQ-QDA#NotSet
15-Jun-201920:31:00
NB_7186_96
(3) ELSD Signal
Range: 1919
LSU
1.06
3: Diode Array
Range: 1.71e+2
AU
0.20
230.1
1.07;244.1
1.64
218.1
1.71;217.1
2: Scan ES-
TIC
2.70e5
1.11;181.2
1.09;236.1
1.14;173.3
0.97;462.5
1.34;155.1
1.49;215.6
2.74
190.1
2.63;379.2
2.82;361.9
1: Scan ES+
TIC
1.80e7
1.13;361.3
1.13;361.4
1.08;361.4
1.15;361.4
0.22
243.4
0.88
243.4
1.17;361.4
1.66;266.4
1.75
280.3
Time

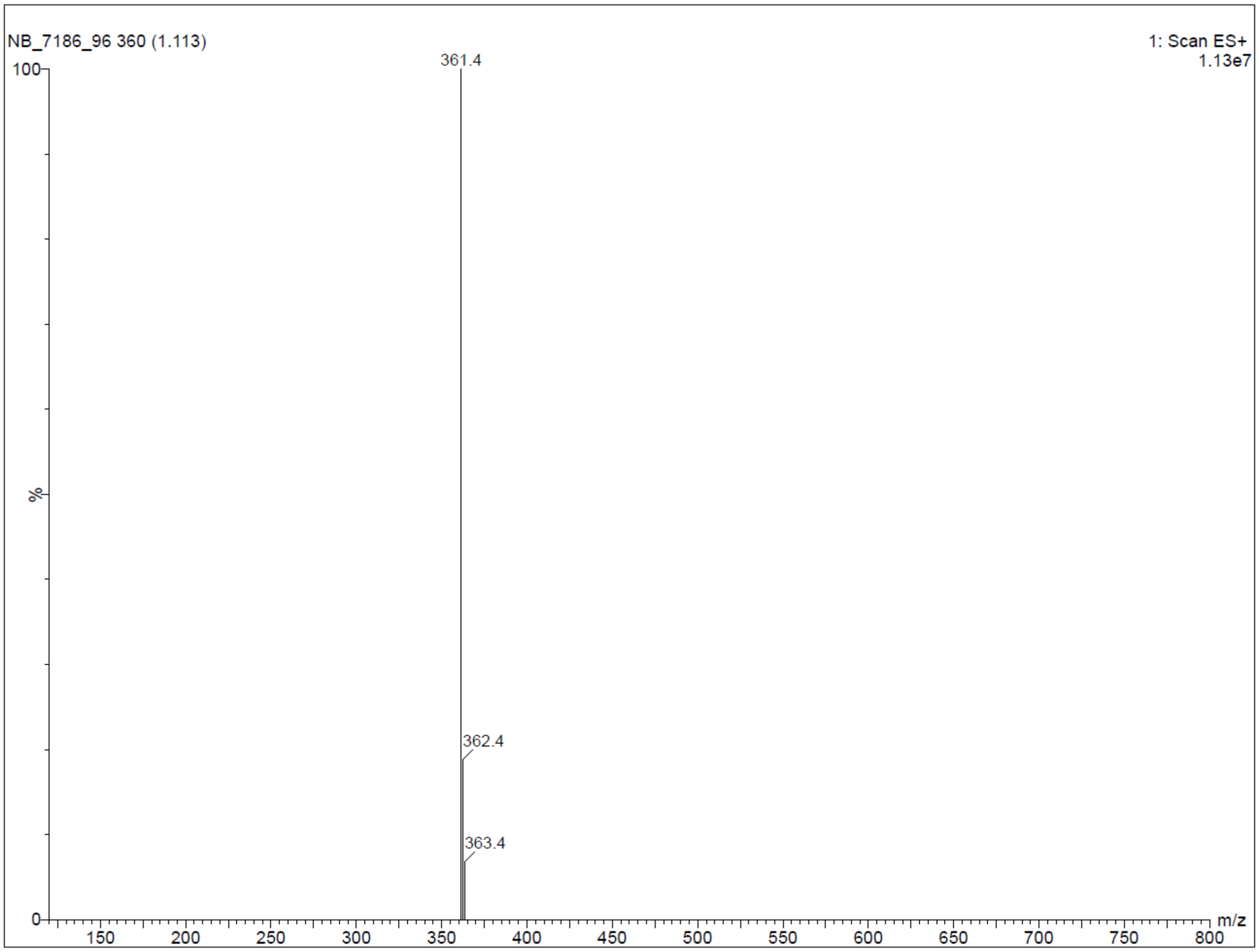

1-Methyl-6-((6S)-6-methyl-4-tosylpiperazin-2-yl)-1H-indole, TFA salt (4os)

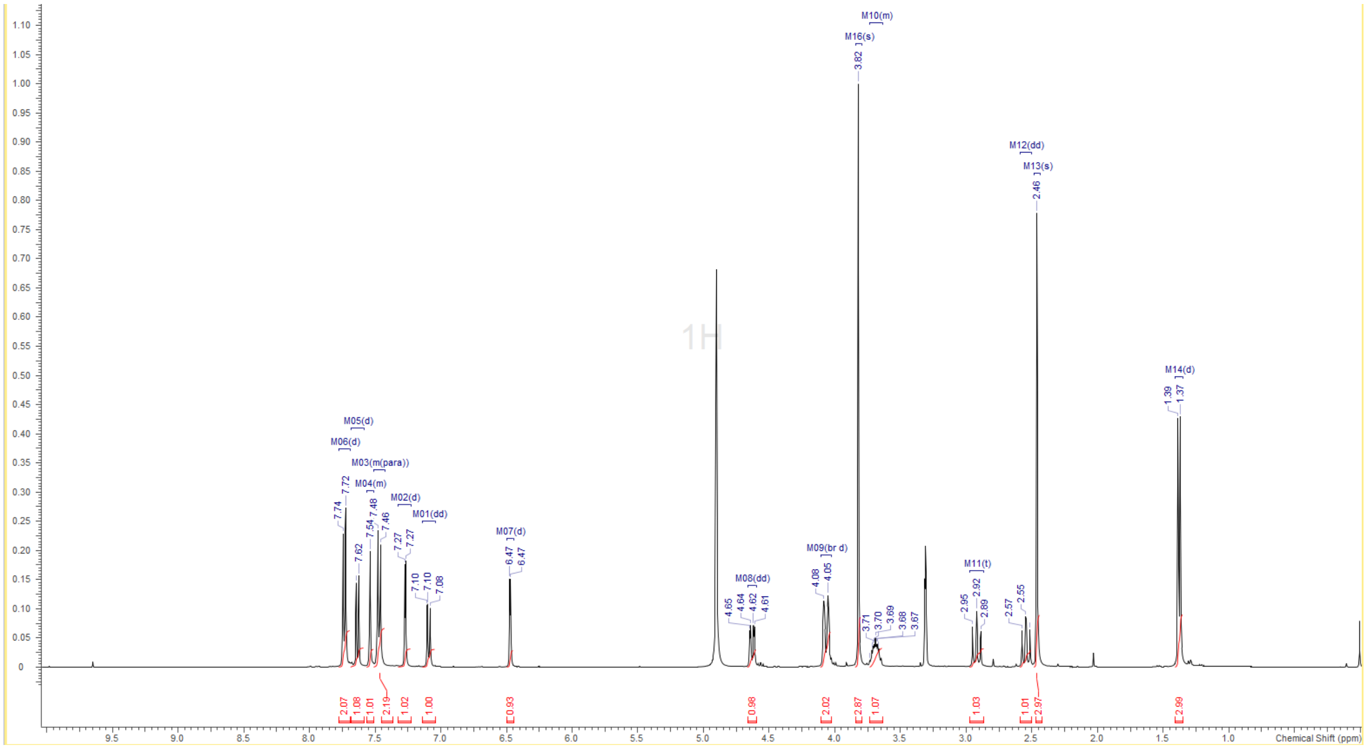

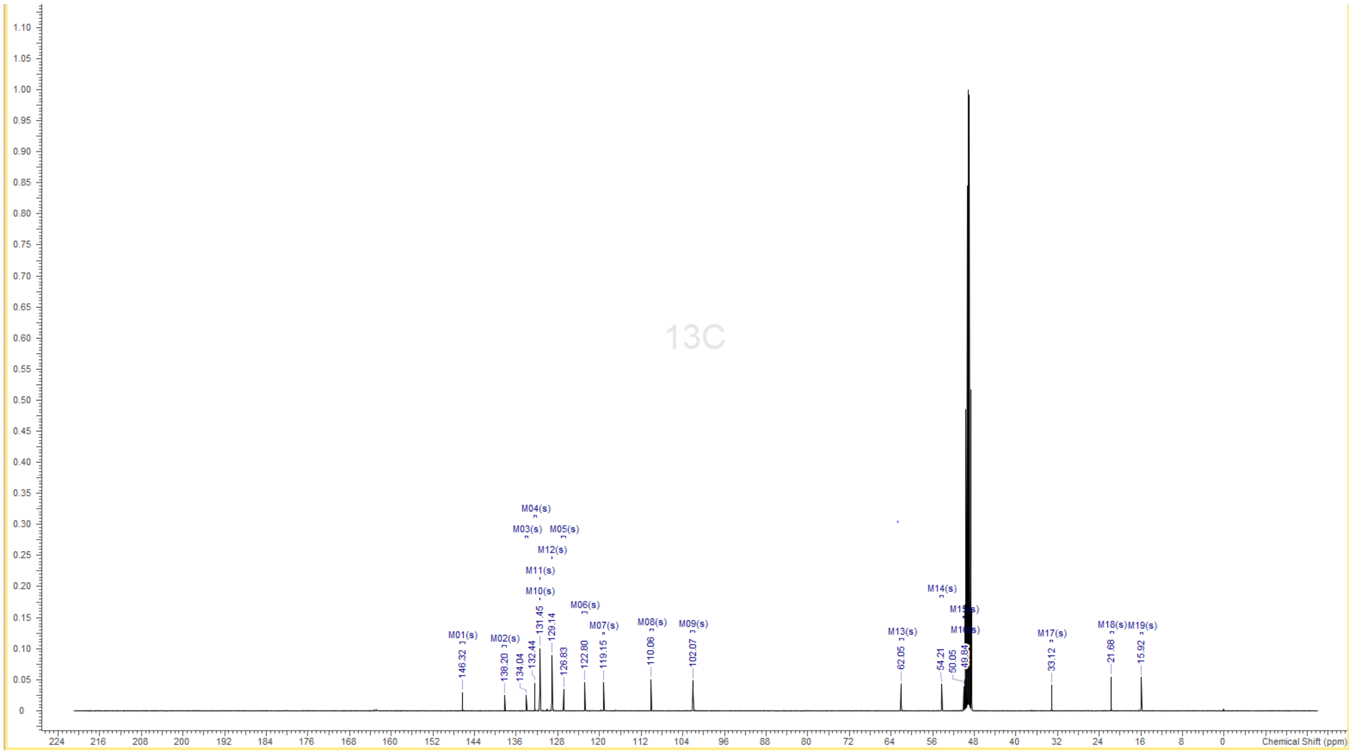
13C
M01(s)
146.32
M02(s)
138.20
134.04
132.44
M03(s)
M04(s)
M05(s)
M10(s)
M11(s)
M12(s)
131.45
129.14
126.83
M06(s)
122.80
M07(s)
119.15
M08(s)
110.06
M09(s)
102.07
M13(s)
62.05
M14(s)
54.21
50.05
49.84
M17(s)
33.12
M18(s)
21.68
M19(s)
15.92
Chemical Shift (ppm)

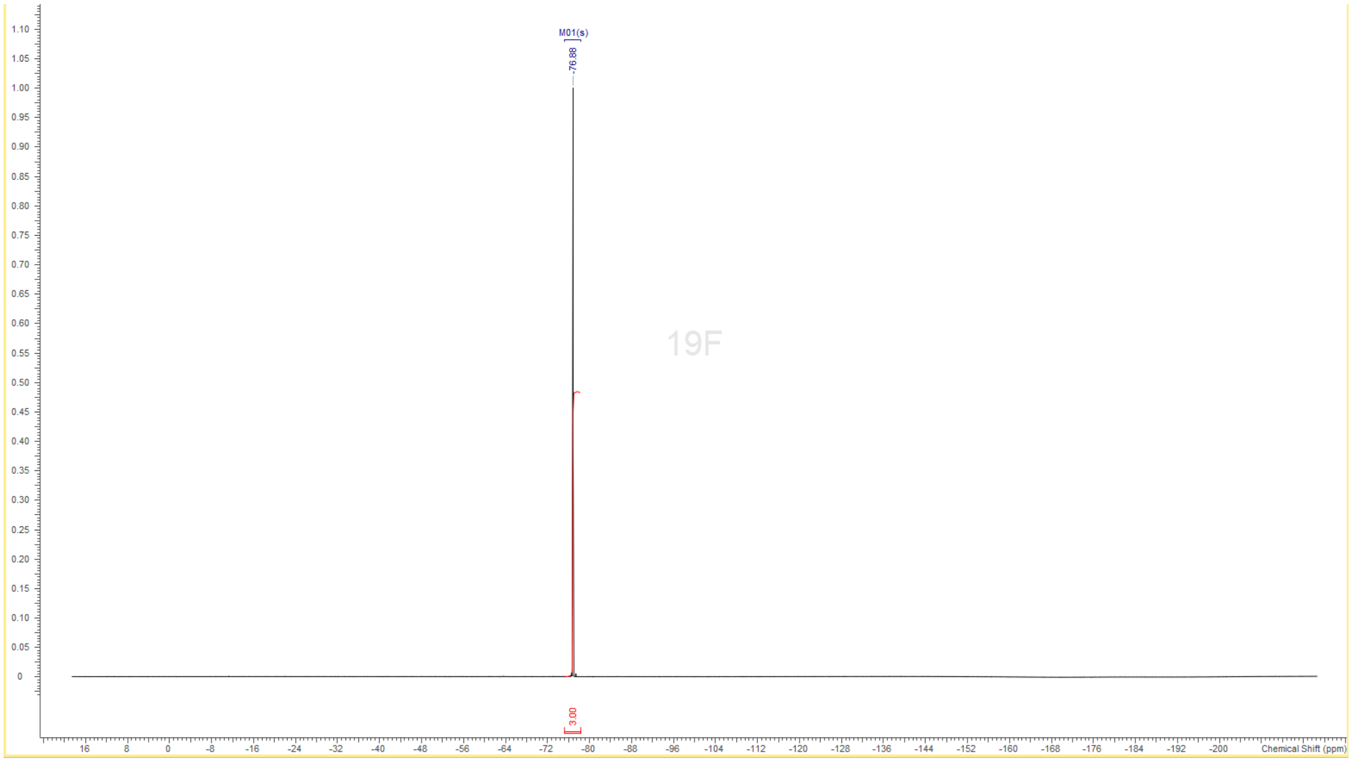
19F
M01(s)
-76.88
3.00
Chemical Shift (ppm)

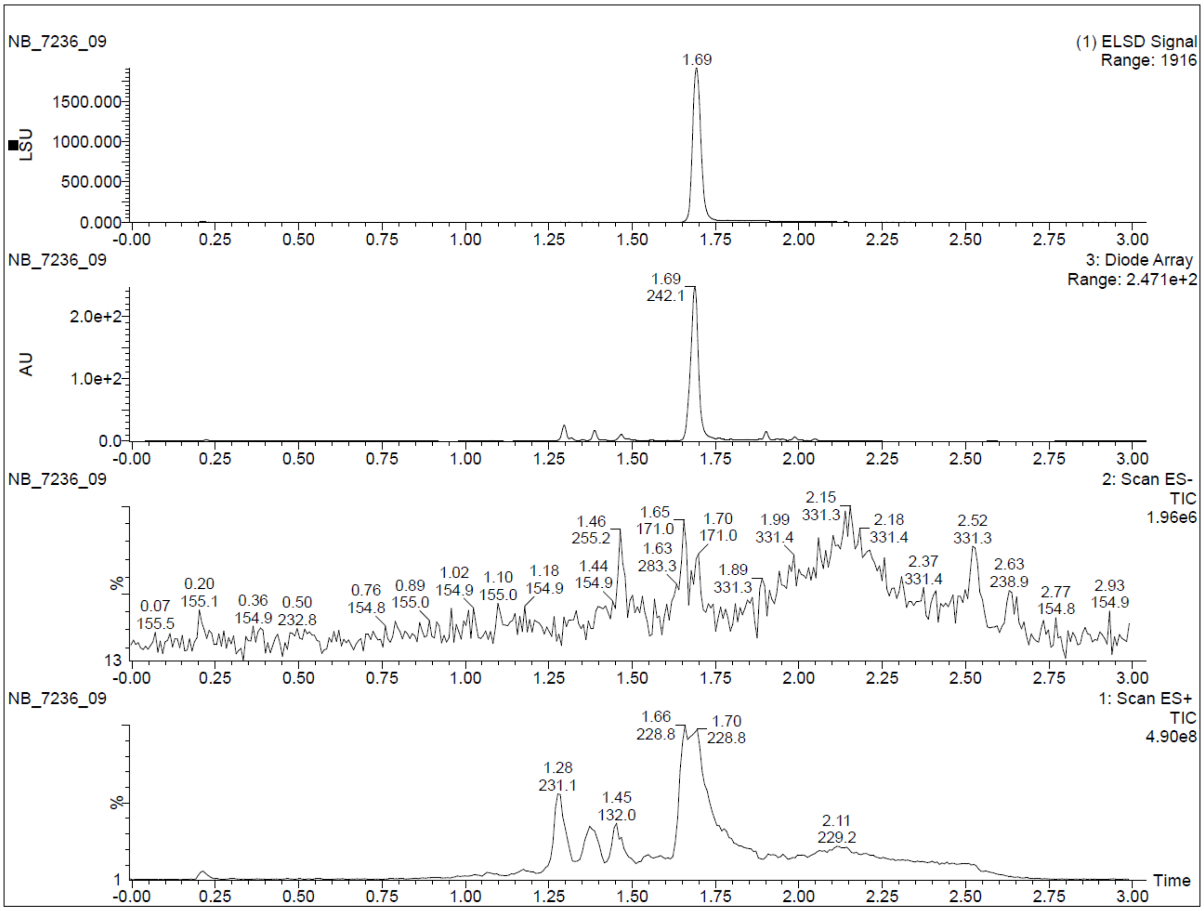
NB_7236_09
(1) ELSD Signal
Range: 1916
1.69
LSU
NB_7236_09
3: Diode Array
Range: 2.471e+2
1.69
242.1
AU
NB_7236_09
2: Scan ES-
TIC
1.96e6
NB_7236_09
1: Scan ES+
TIC
4.90e8
1.28
231.1
1.45
132.0
1.66
228.8
1.70
228.8
2.11
229.2
Time

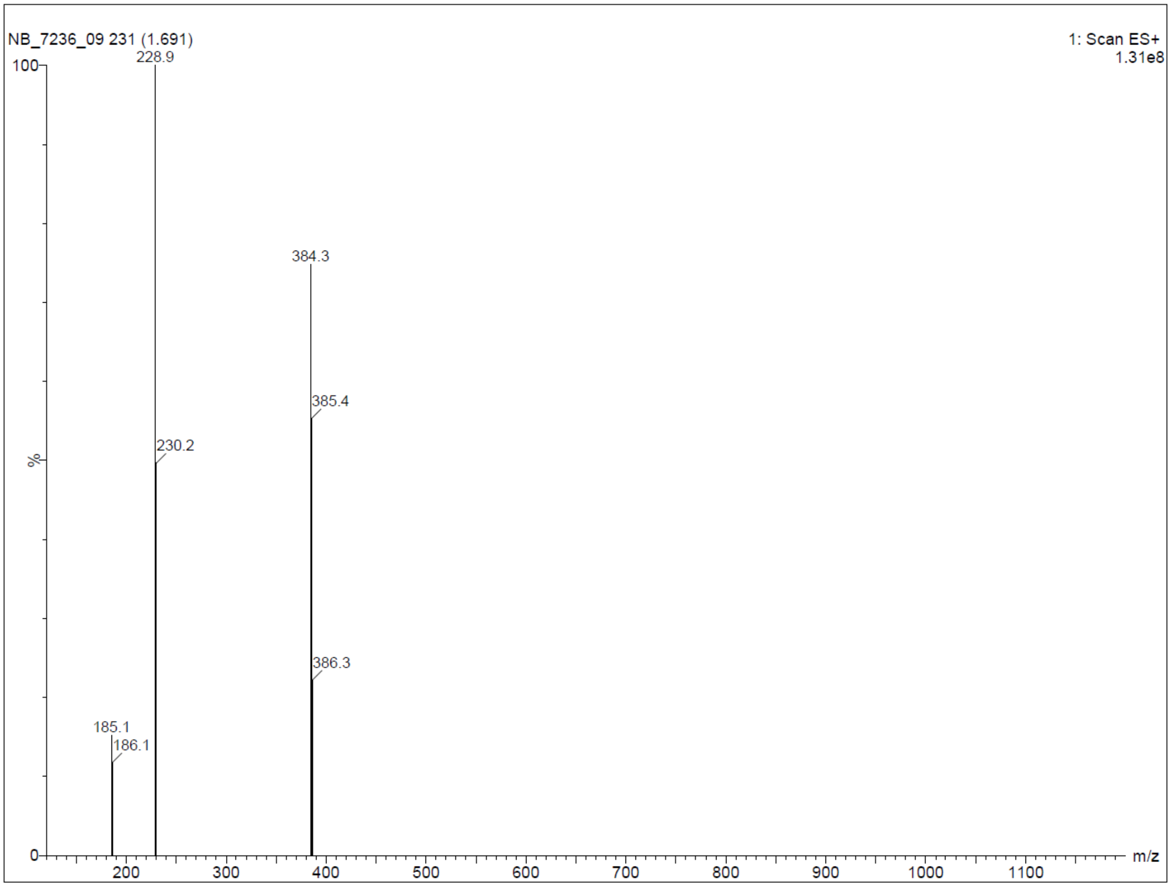
NB_7236_09 231 (1.691)
1: Scan ES+
1.31e8
228.9
230.2
384.3
385.4
386.3
185.1
186.1
%
m/z

(5S)-3-Cyclopropyl-5-methyl-1-tosylpiperazine, Free base (4oα)

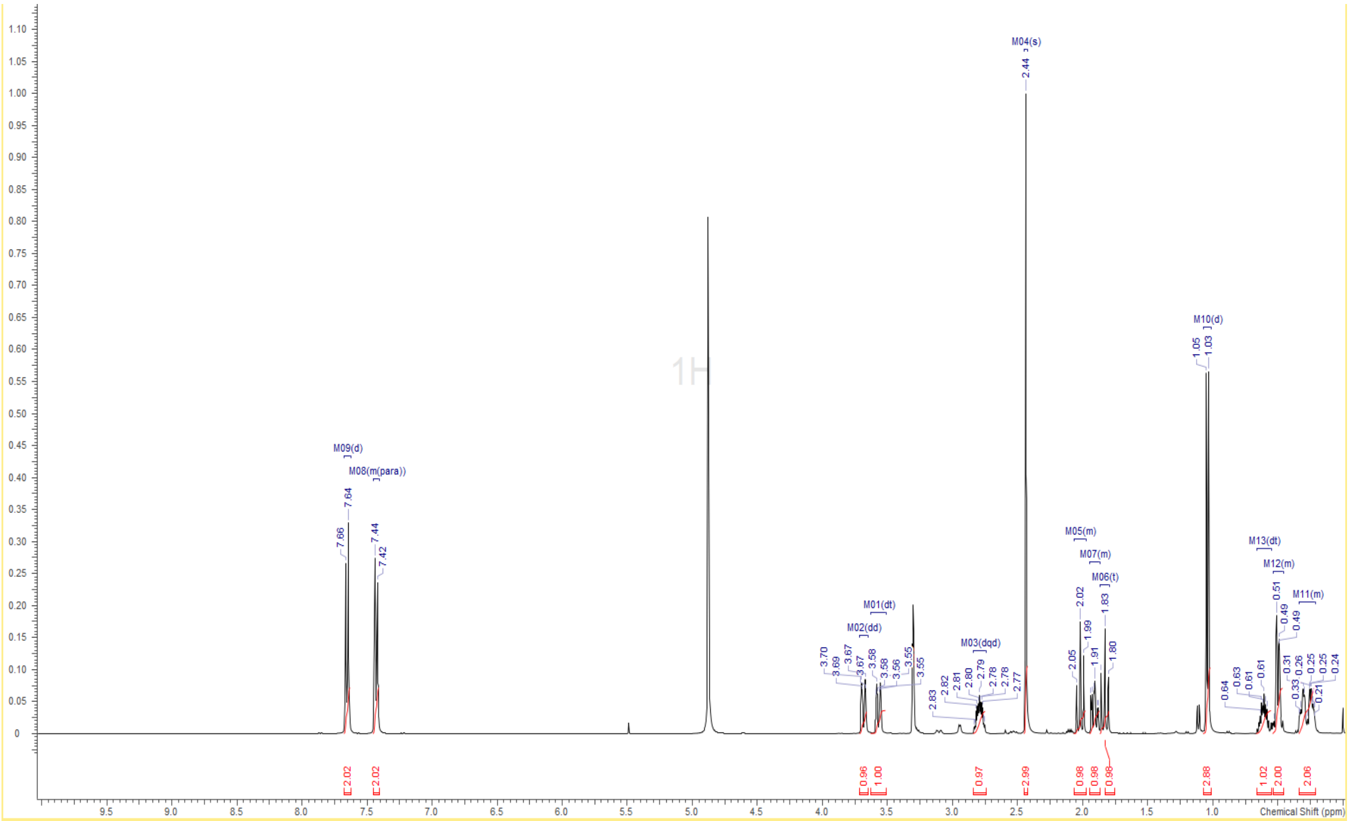

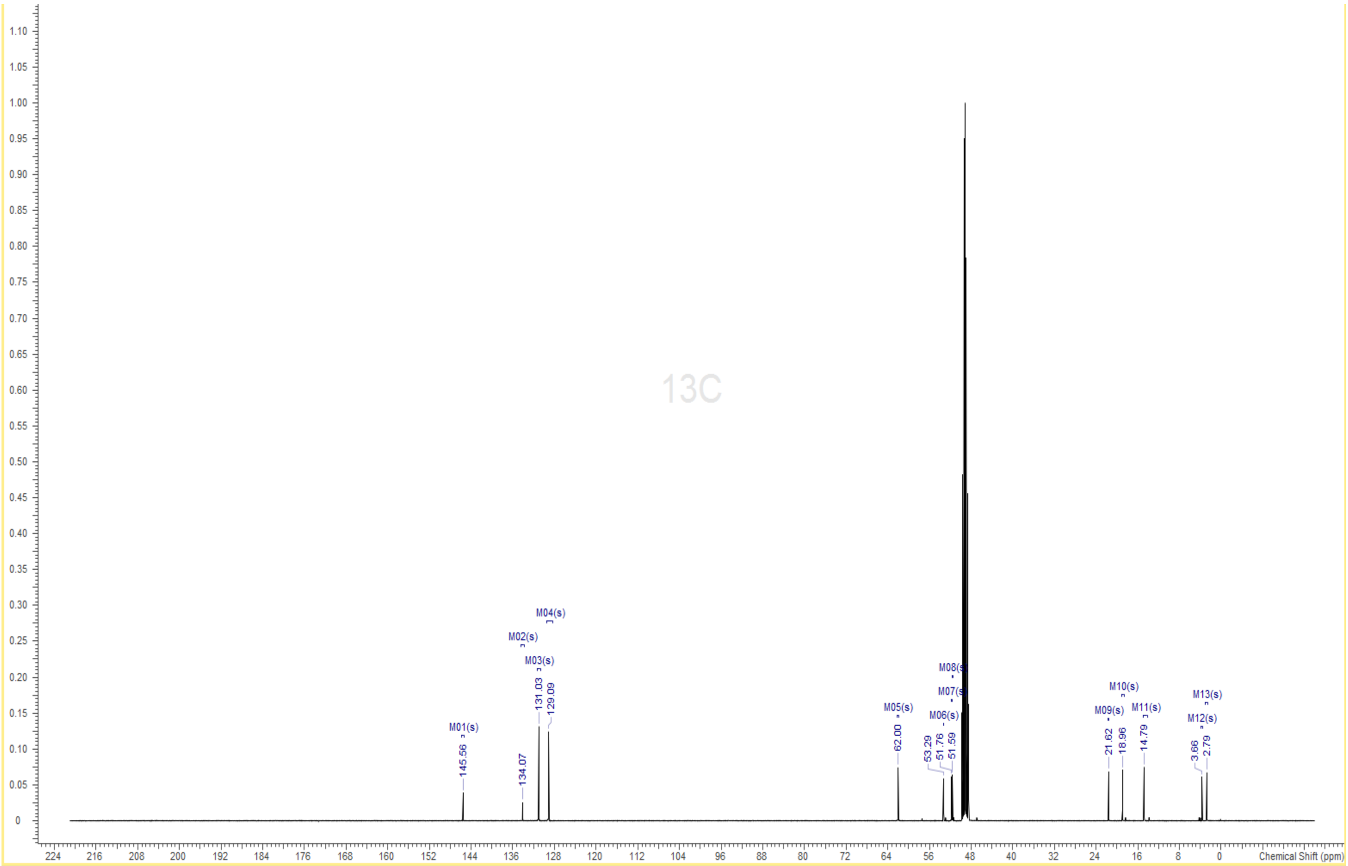

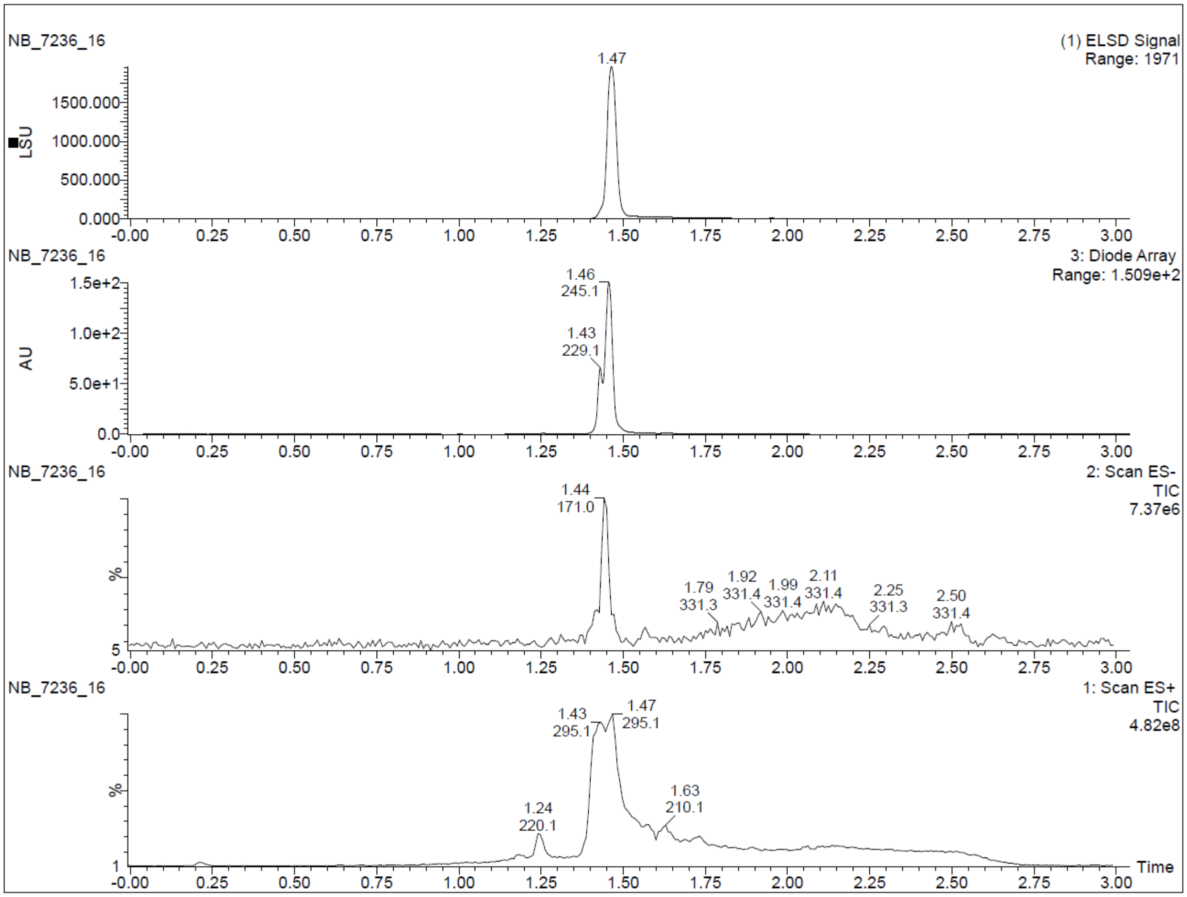

NB_7236_16
(1) ELSD Signal
Range: 1971
1.47
LSU
3: Diode Array
Range: 1.509e+2
1.46
245.1
1.43
229.1
AU
2: Scan ES-
TIC
7.37e6
1.44
171.0
1: Scan ES+
TIC
4.82e8
1.43
295.1
1.47
295.1
1.24
220.1
1.63
210.1
Time

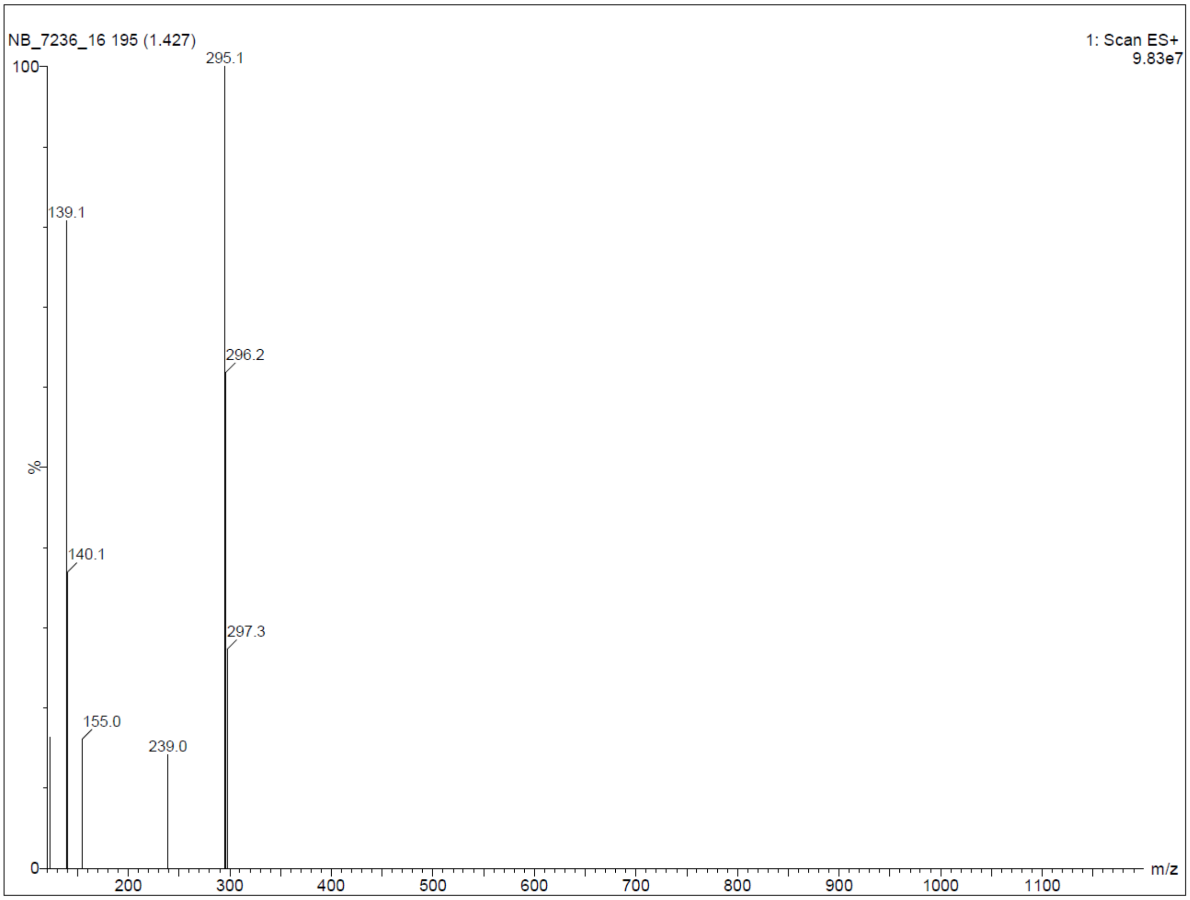

NB_7236_16 195 (1.427)
1: Scan ES+
9.83e7
295.1
139.1
296.2
140.1
297.3
155.0
239.0
m/z

5-(4-Methoxyphenyl)-2-methyl-1-tosylpiperazine, Free base (4pa)

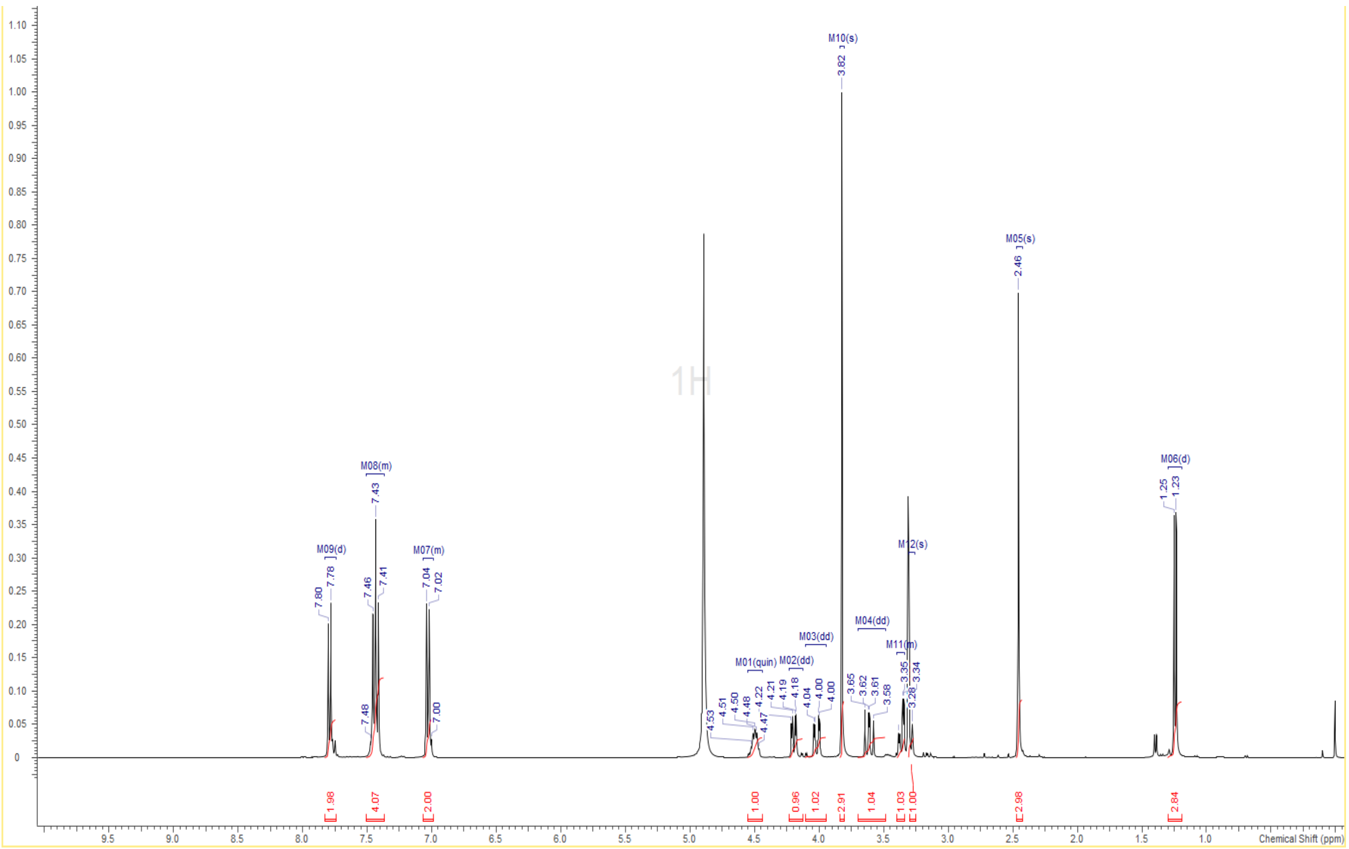

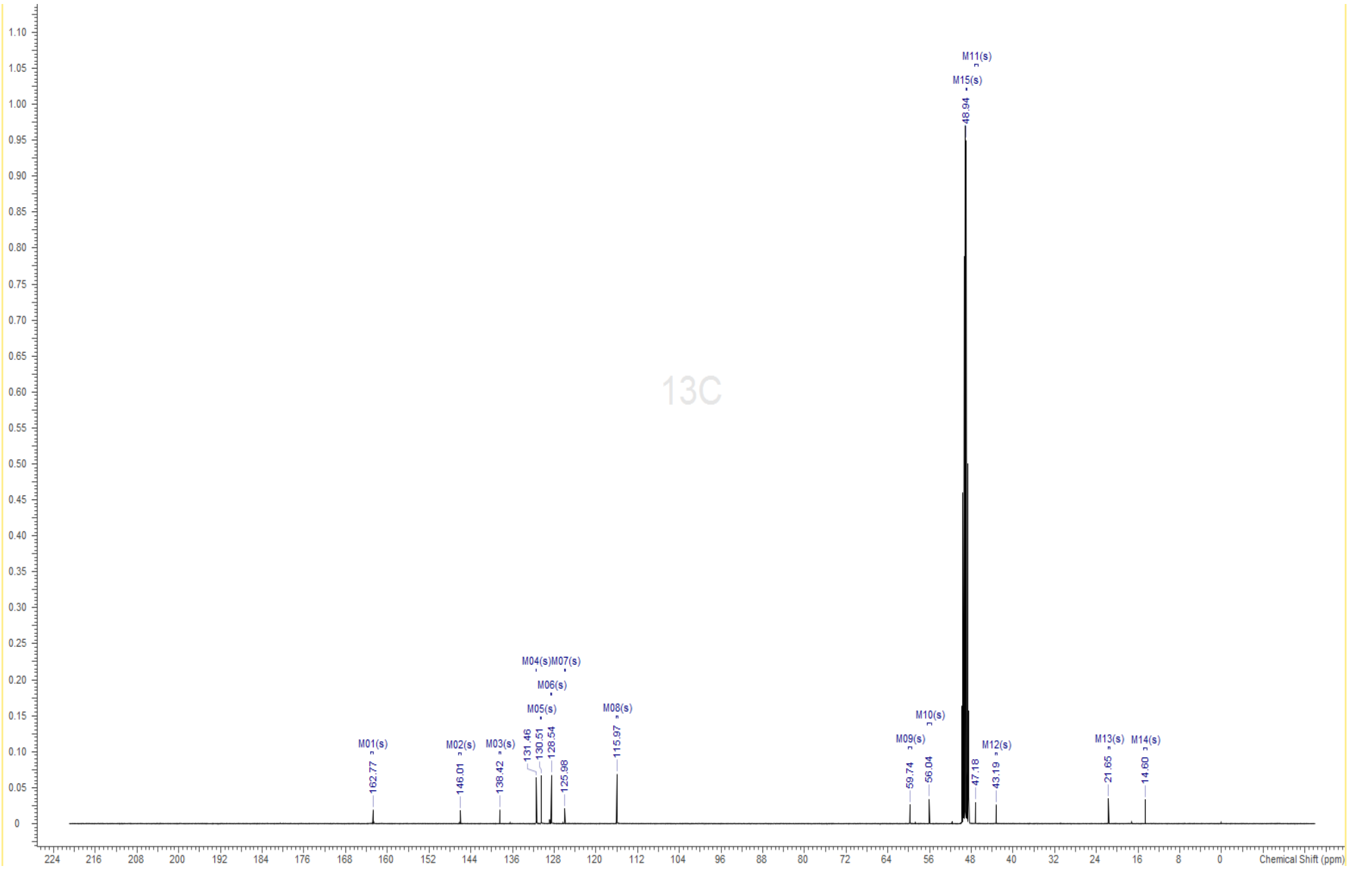

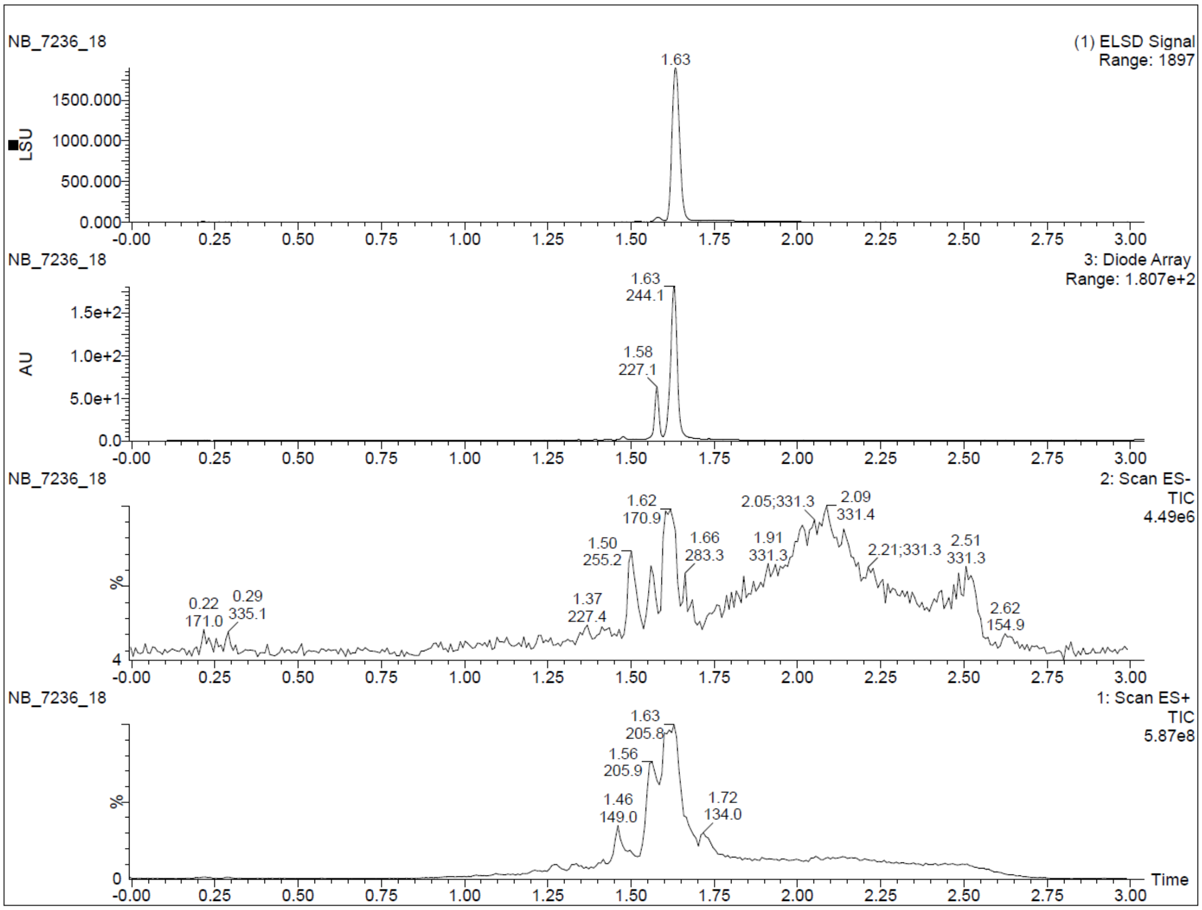

NB_7236_18
(1) ELSD Signal
Range: 1897
1.63
LSU
NB_7236_18
3: Diode Array
Range: 1.807e+2
1.63
244.1
1.58
227.1
AU
NB_7236_18
2: Scan ES-
TIC
4.49e6
1.62
170.9
2.05;331.3
2.09
331.4
1.50
255.2
1.66
283.3
1.91
331.3
2.21;331.3
2.51
331.3
0.22
171.0
0.29
335.1
1.37
227.4
2.62
154.9
NB_7236_18
1: Scan ES+
TIC
5.87e8
1.63
205.8
1.56
205.9
1.46
149.0
1.72
134.0
Time

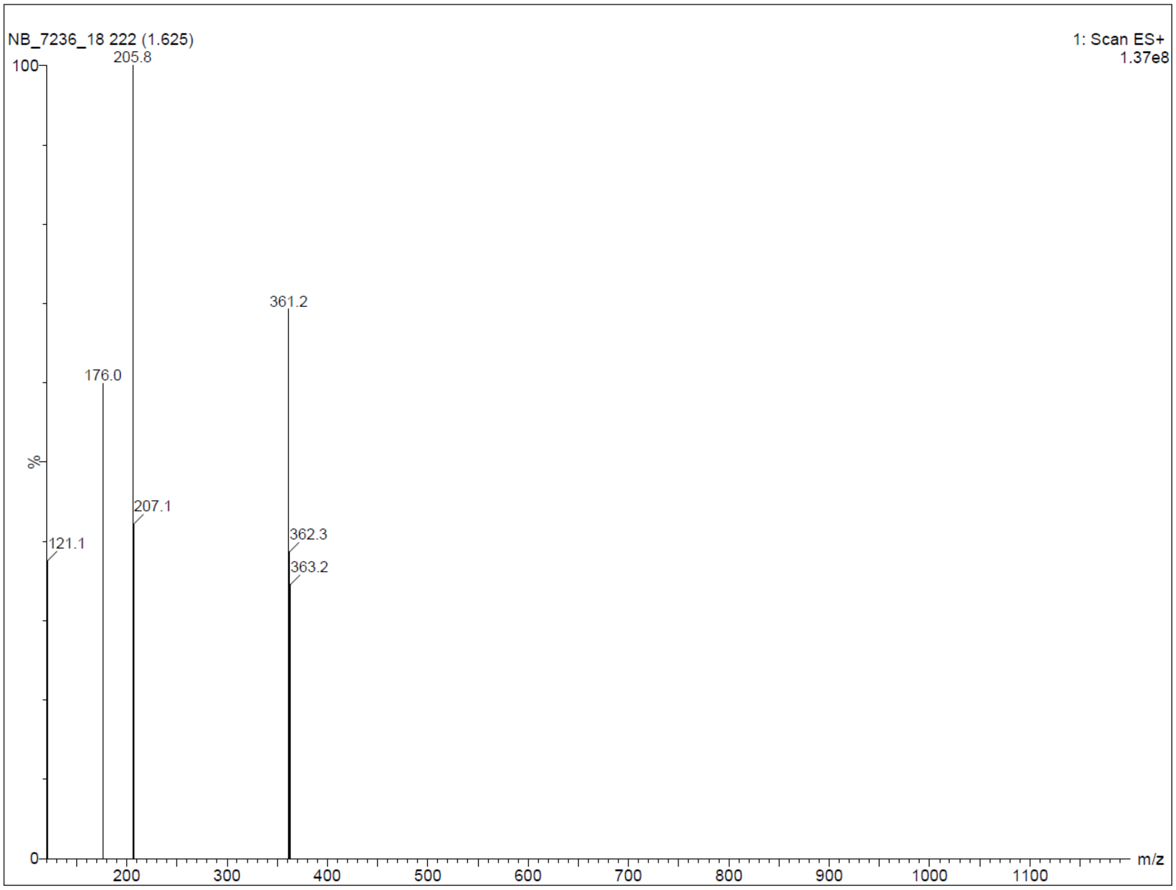

NB_7236_18 222 (1.625)
1: Scan ES+
1.37e8
205.8
361.2
176.0
207.1
121.1
362.3
363.2
%
m/z

5-(5-Methyl-4-tosylpiperazin-2-yl)isoquinoline, Free base (4pj)

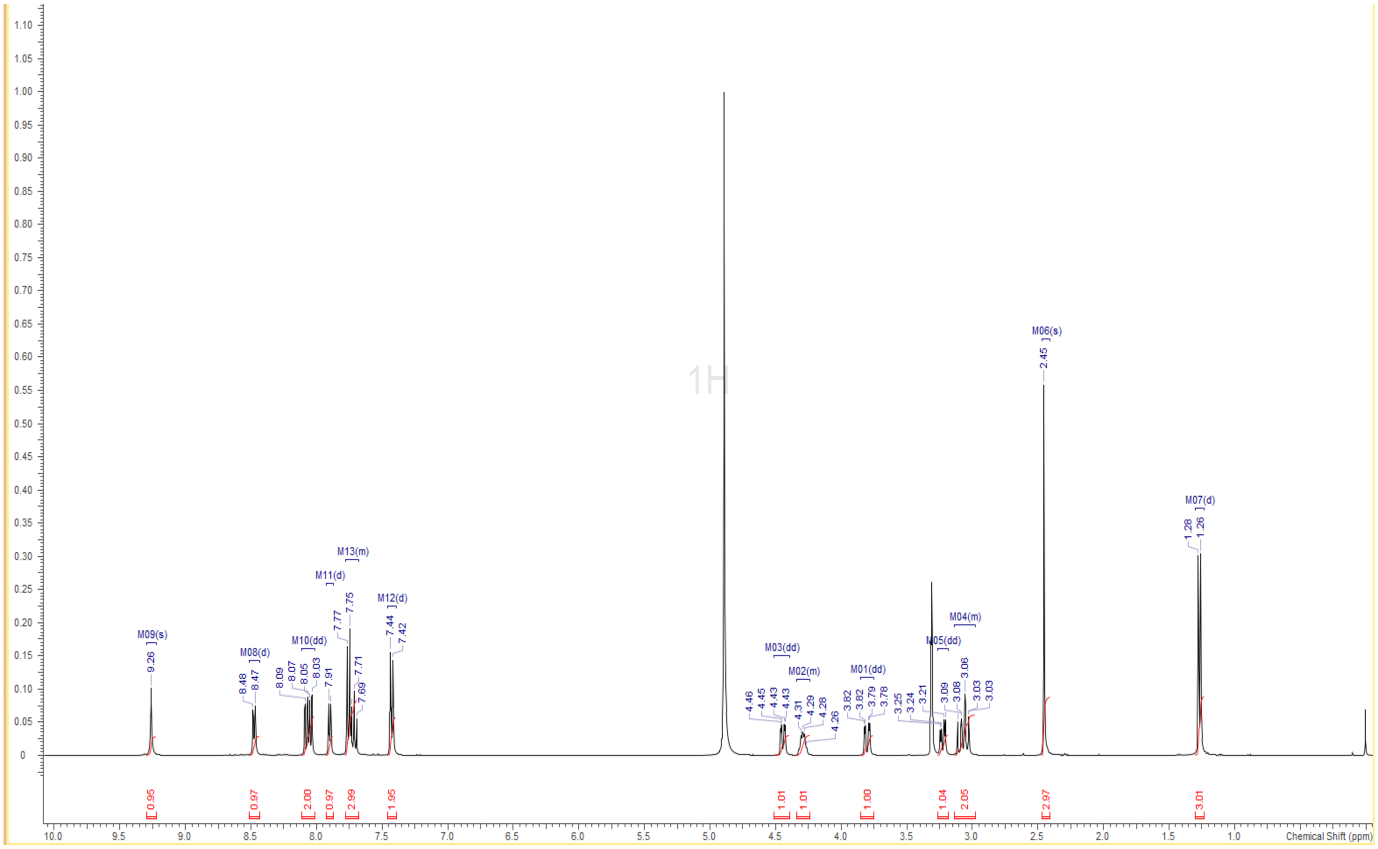

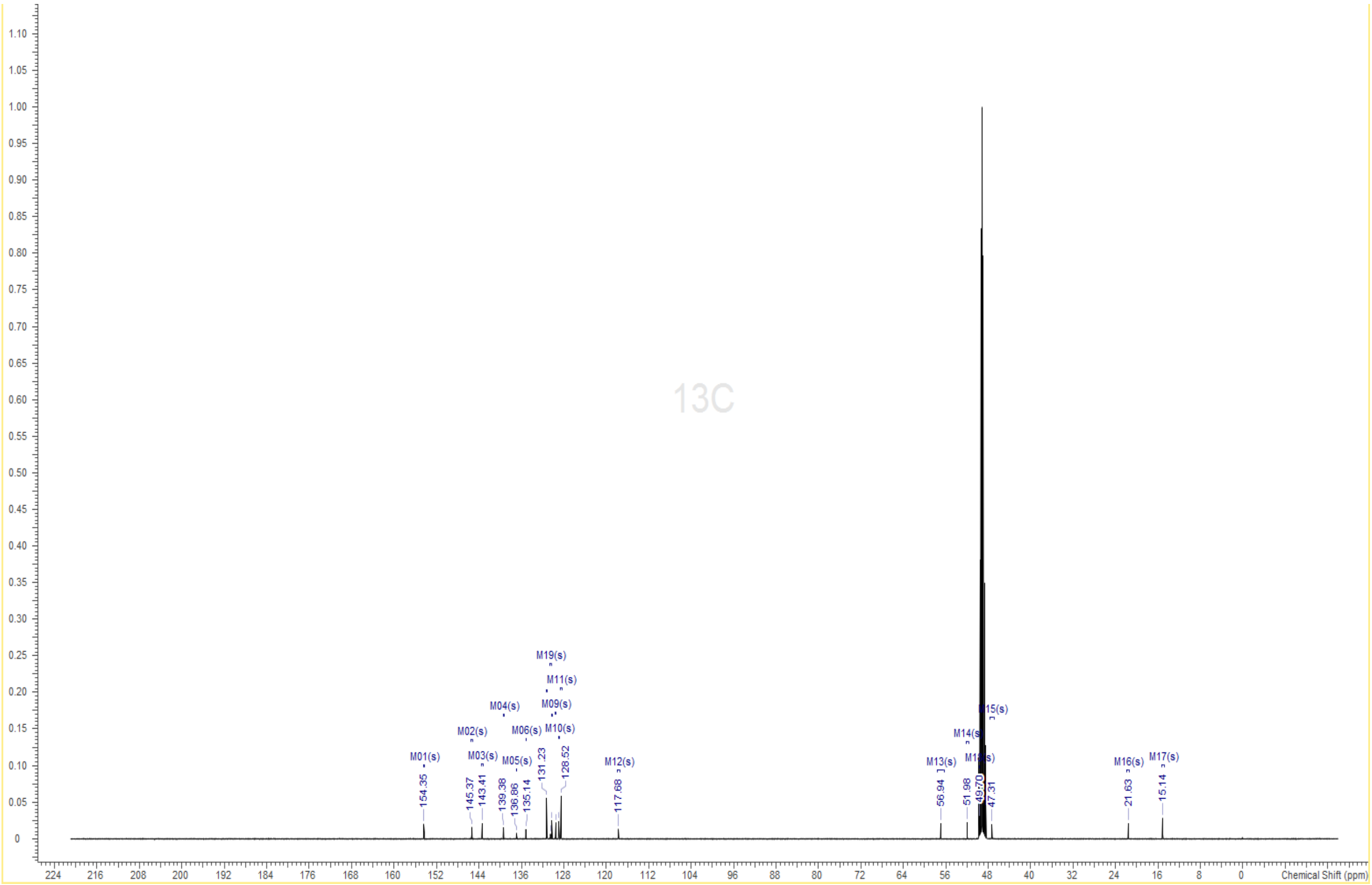

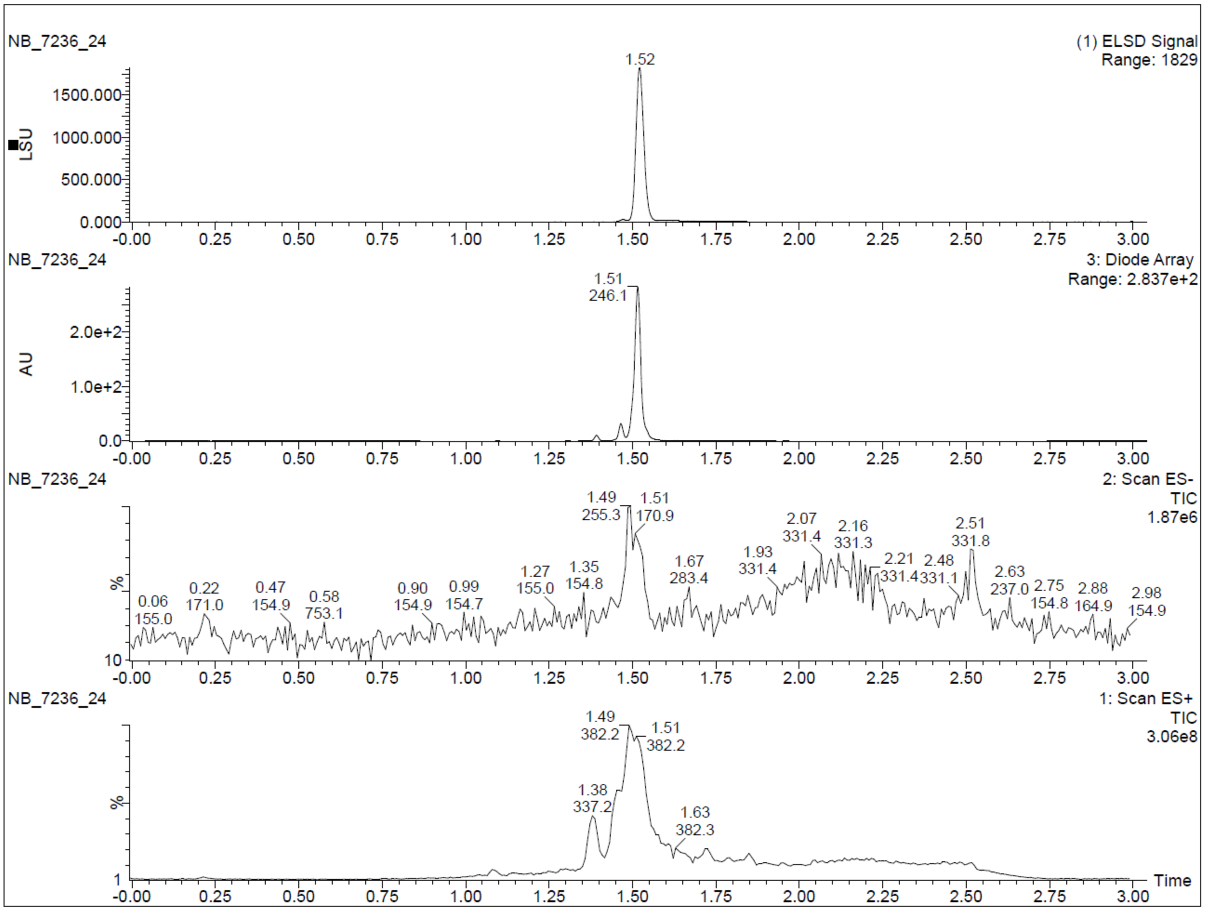
NB_7236_24
(1) ELSD Signal
Range: 1829
1.52
LSU
3: Diode Array
Range: 2.837e+2
1.51
246.1
AU
2: Scan ES-
TIC
1.87e6
1.49
255.3
1.51
170.9
1: Scan ES+
TIC
3.06e8
1.49
382.2
1.51
382.2
1.38
337.2
1.63
382.3
Time

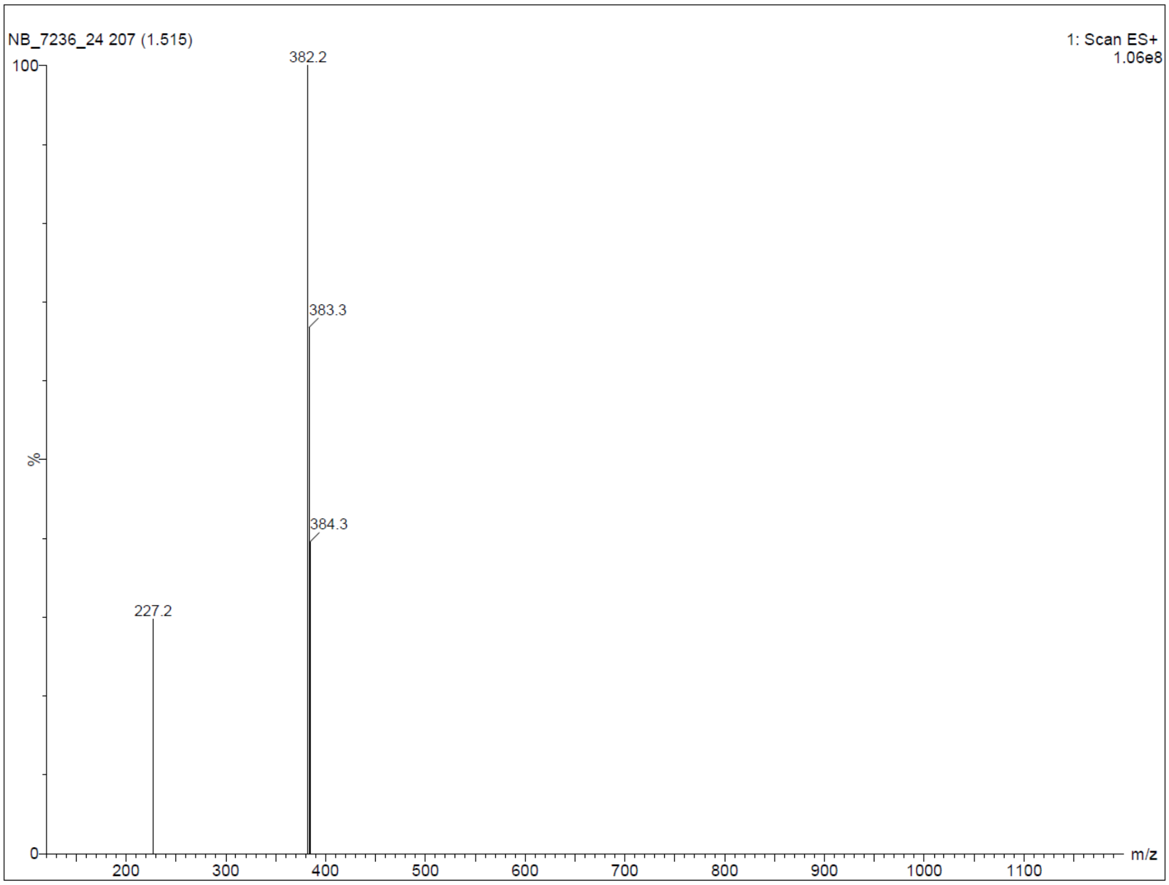
NB_7236_24 207 (1.515)
1: Scan ES+
1.06e8
382.2
383.3
384.3
227.2
%
m/z

7-(6-Phenyl-2,3-dihydroimidazo[2,1-b]thiazol-5-yl)-5-oxa-8-azaspiro[3.5]nonane, Free base (4bw)

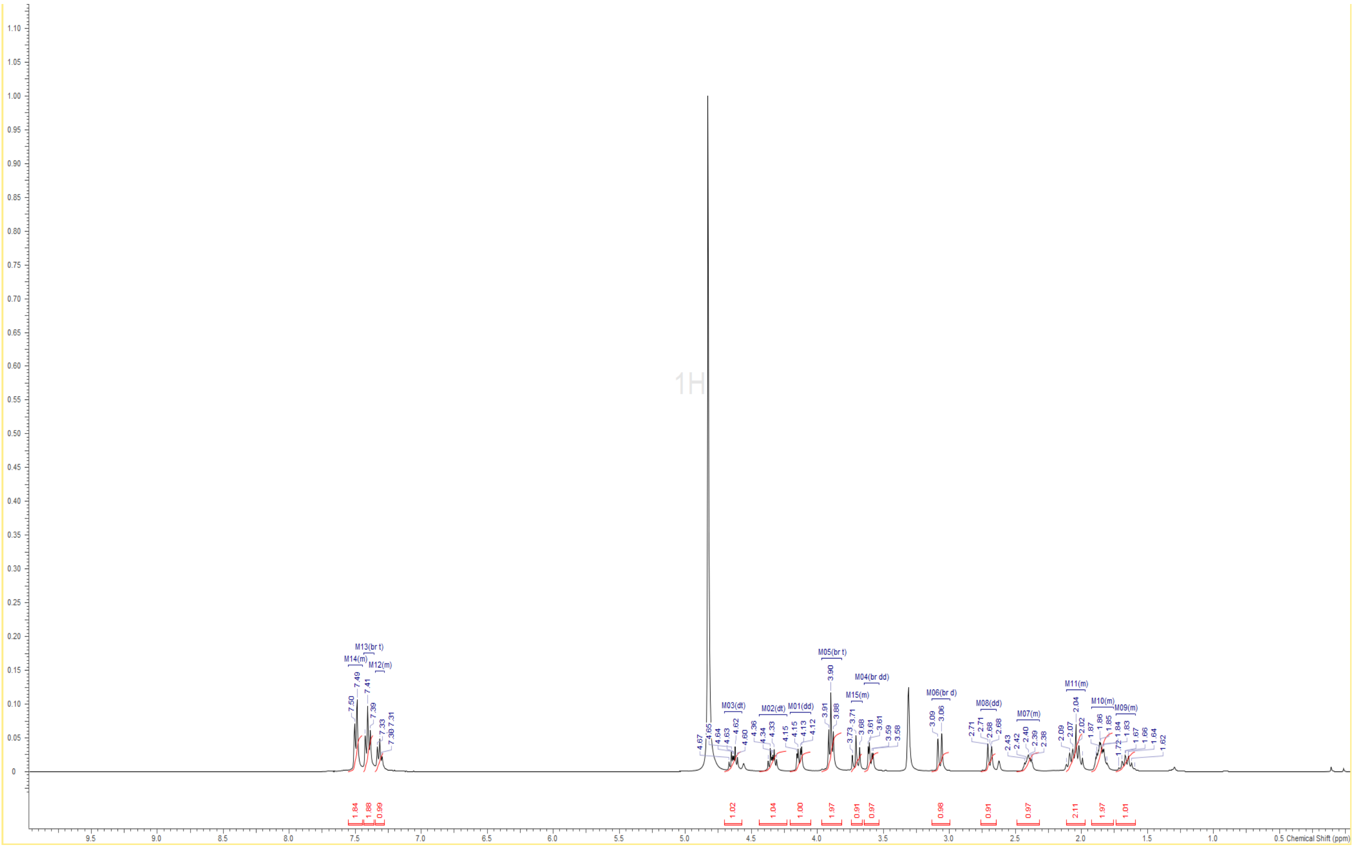

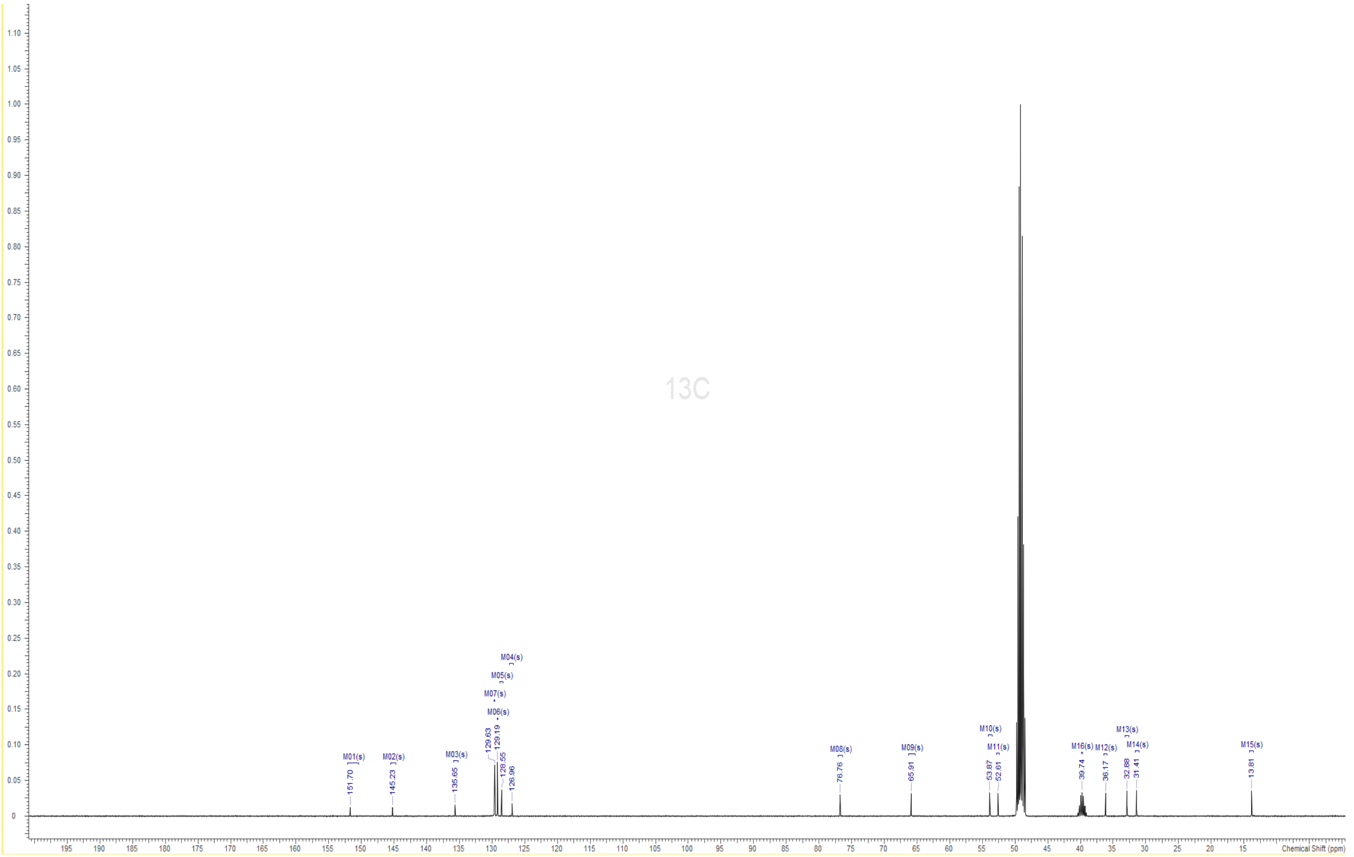

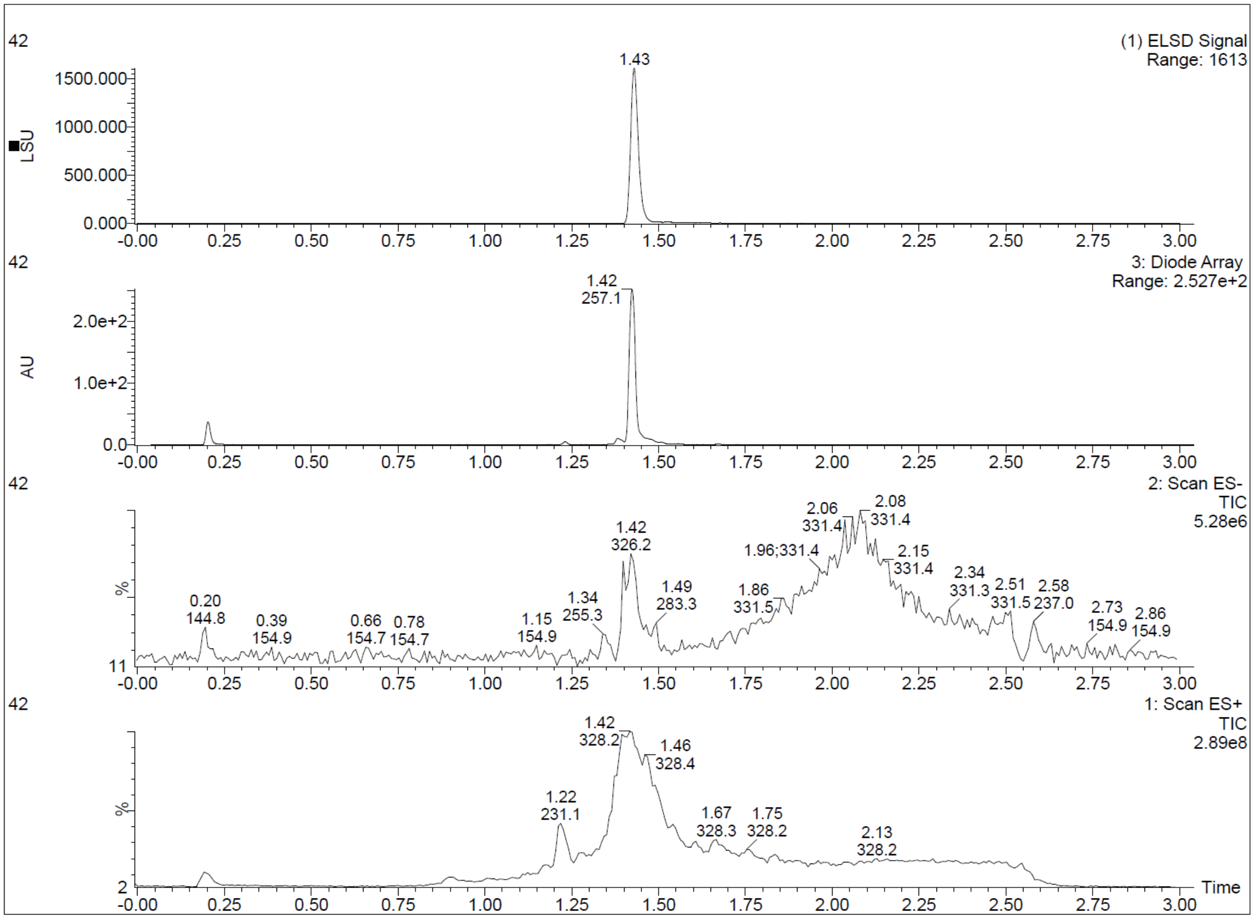

42
(1) ELSD Signal
Range: 1613
1.43
LSU
1500.000
1000.000
500.000
0.000
3: Diode Array
Range: 2.527e+2
1.42
257.1
AU
2.0e+2
1.0e+2
0.0
2: Scan ES-
TIC
5.28e6
1.42
326.2
2.06
331.4
2.08
331.4
1.96;331.4
2.15
331.4
1: Scan ES+
TIC
2.89e8
1.42
328.2
1.46
328.4
1.22
231.1
Time

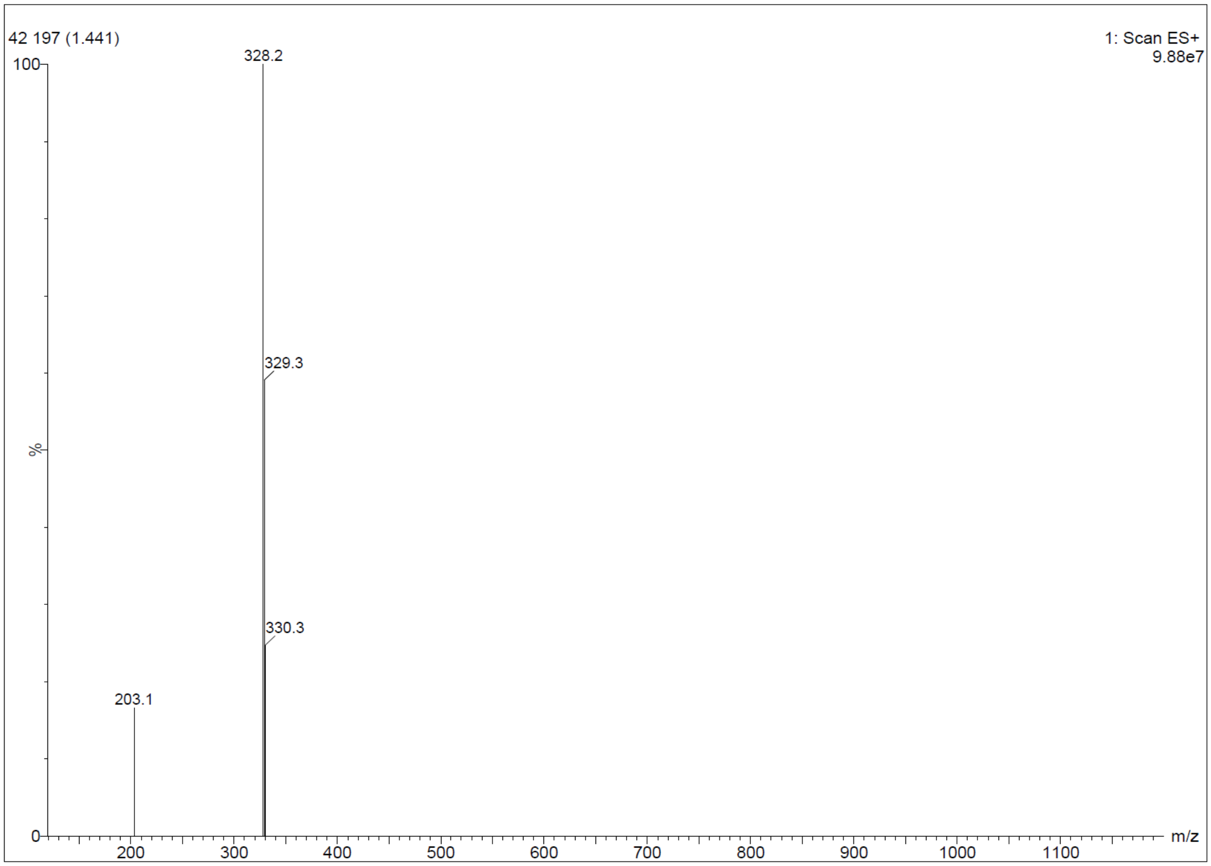

42 197 (1.441)
1: Scan ES+
9.88e7
328.2
329.3
330.3
203.1
%
m/z

tert-Butyl (S)-2,2-dimethyl-4-((3R,6S)-6-phenylmorpholin-3-yl)oxazolidine-3-carboxylate, Free base (4eβ)

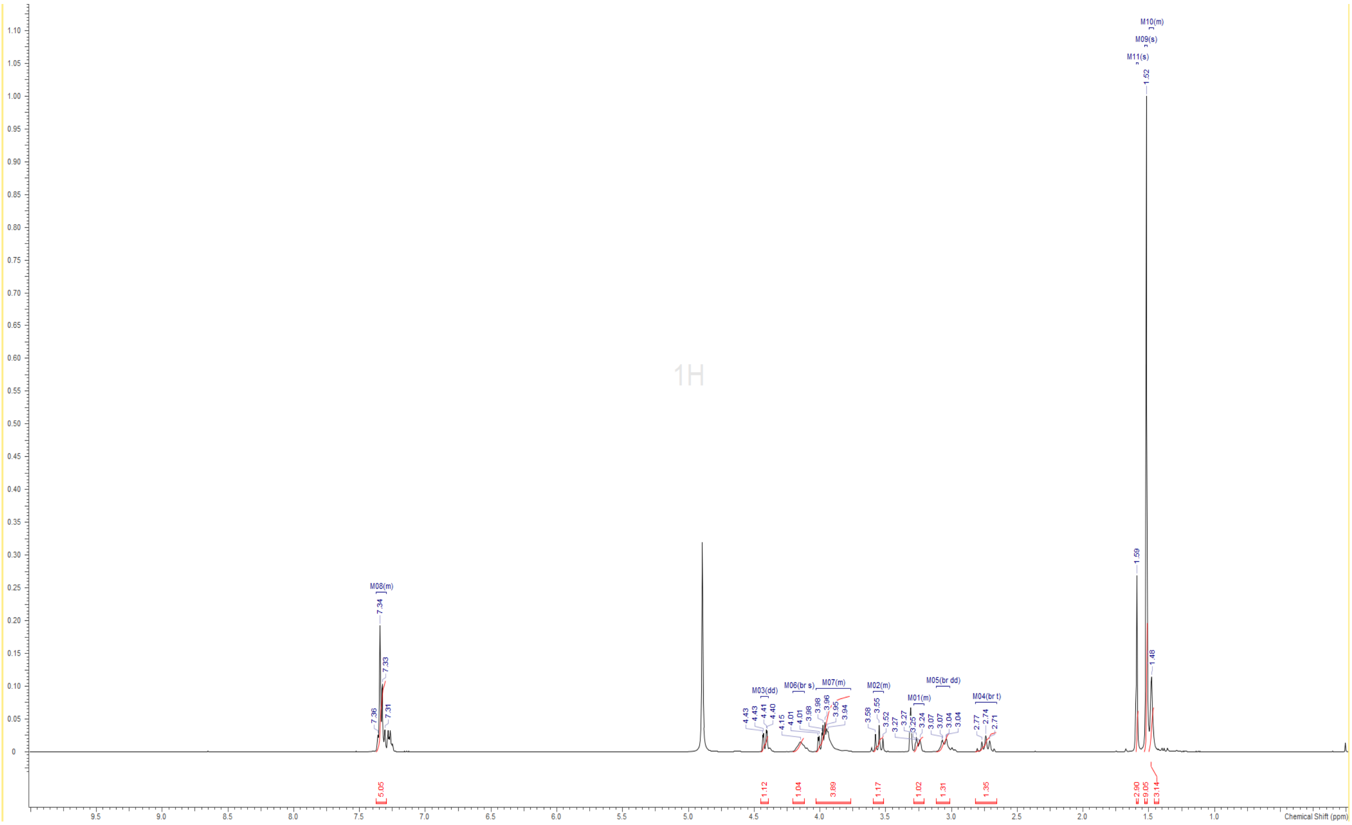

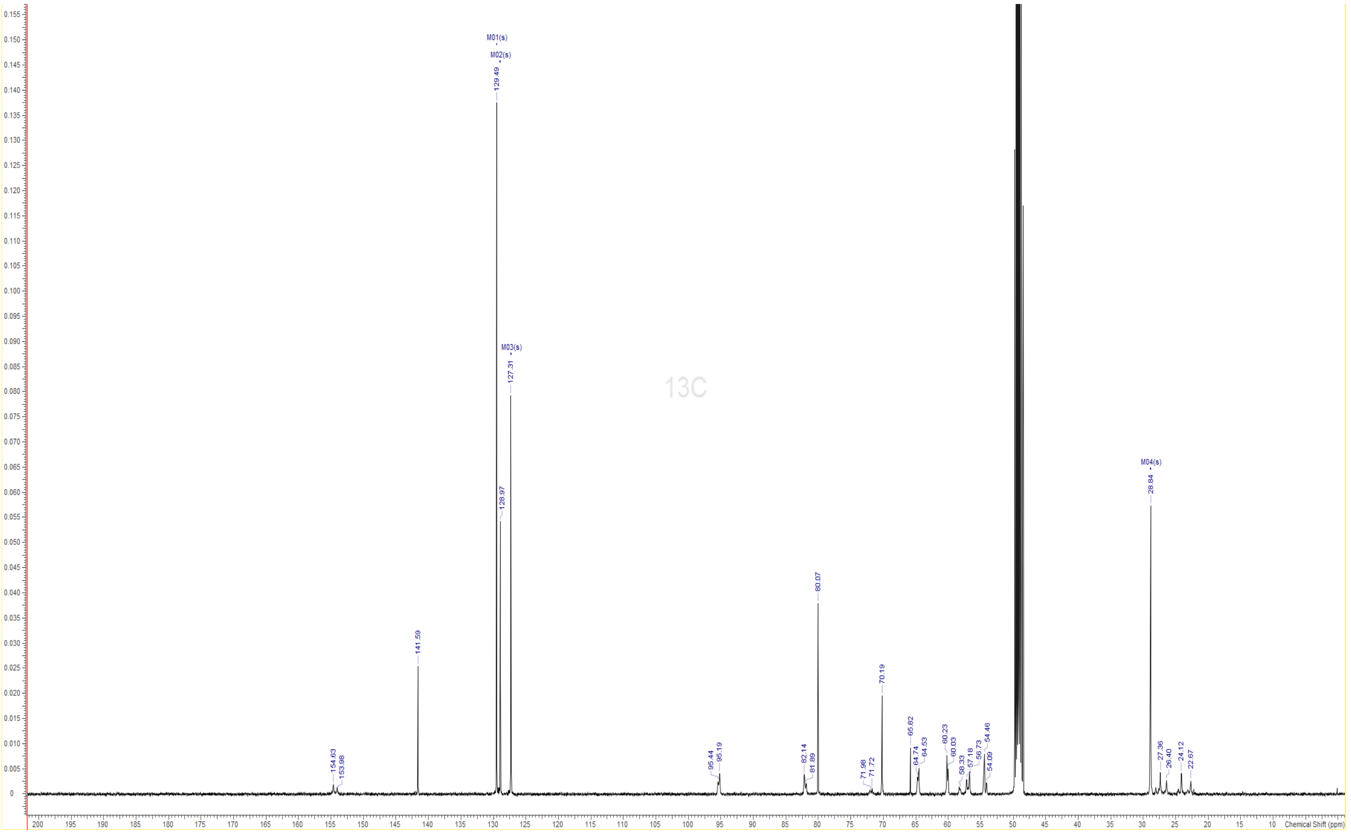

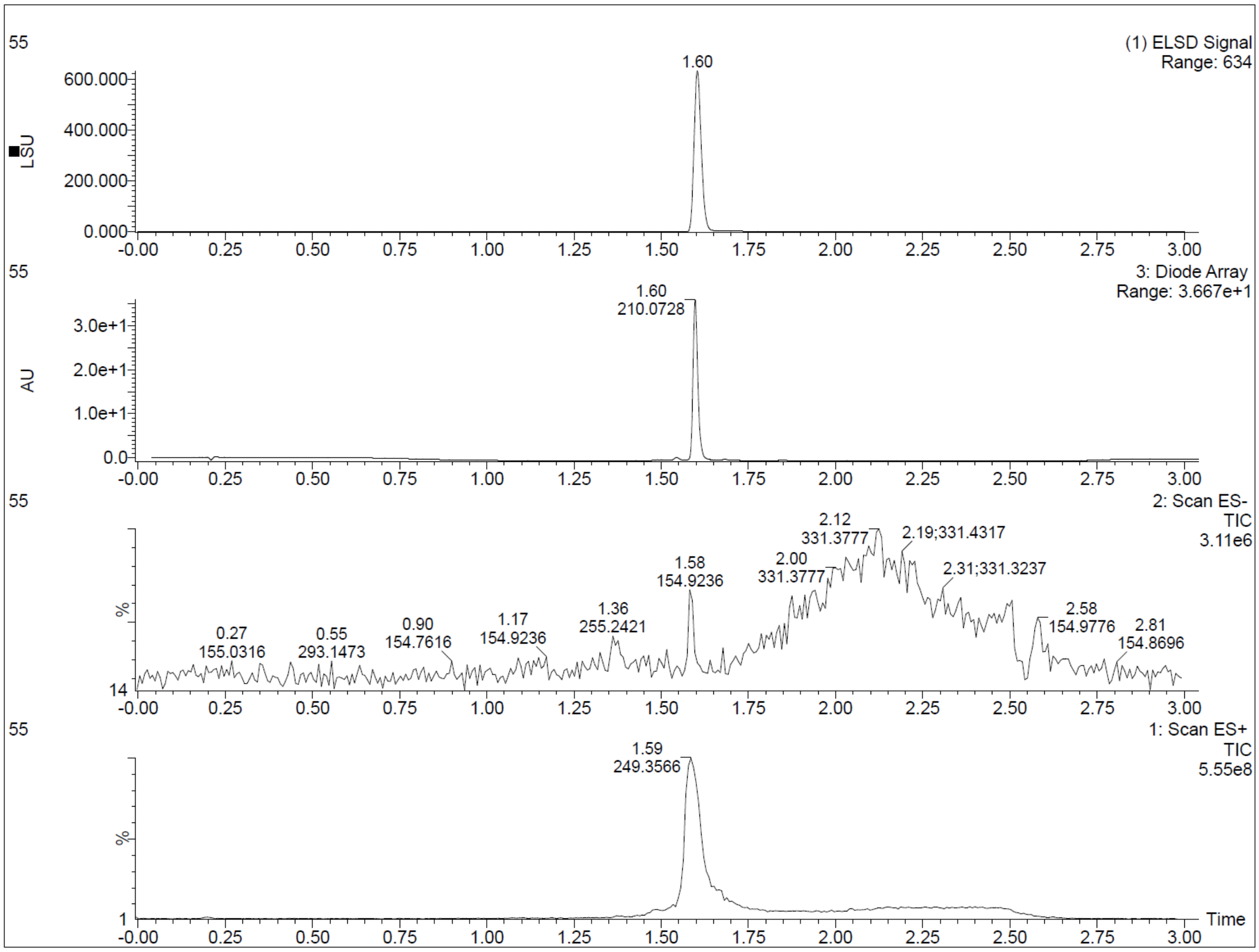
55
(1) ELSD Signal
Range: 634
1.60
LSU
55
3: Diode Array
Range: 3.667e+1
1.60
210.0728
AU
55
2: Scan ES-
TIC
3.11e6
2.12
331.3777
2.19;331.4317
2.00
331.3777
2.31;331.3237
1.58
154.9236
1.36
255.2421
0.27
155.0316
0.55
293.1473
0.90
154.7616
1.17
154.9236
2.58
154.9776
2.81
154.8696
55
1: Scan ES+
TIC
5.55e8
1.59
249.3566
Time

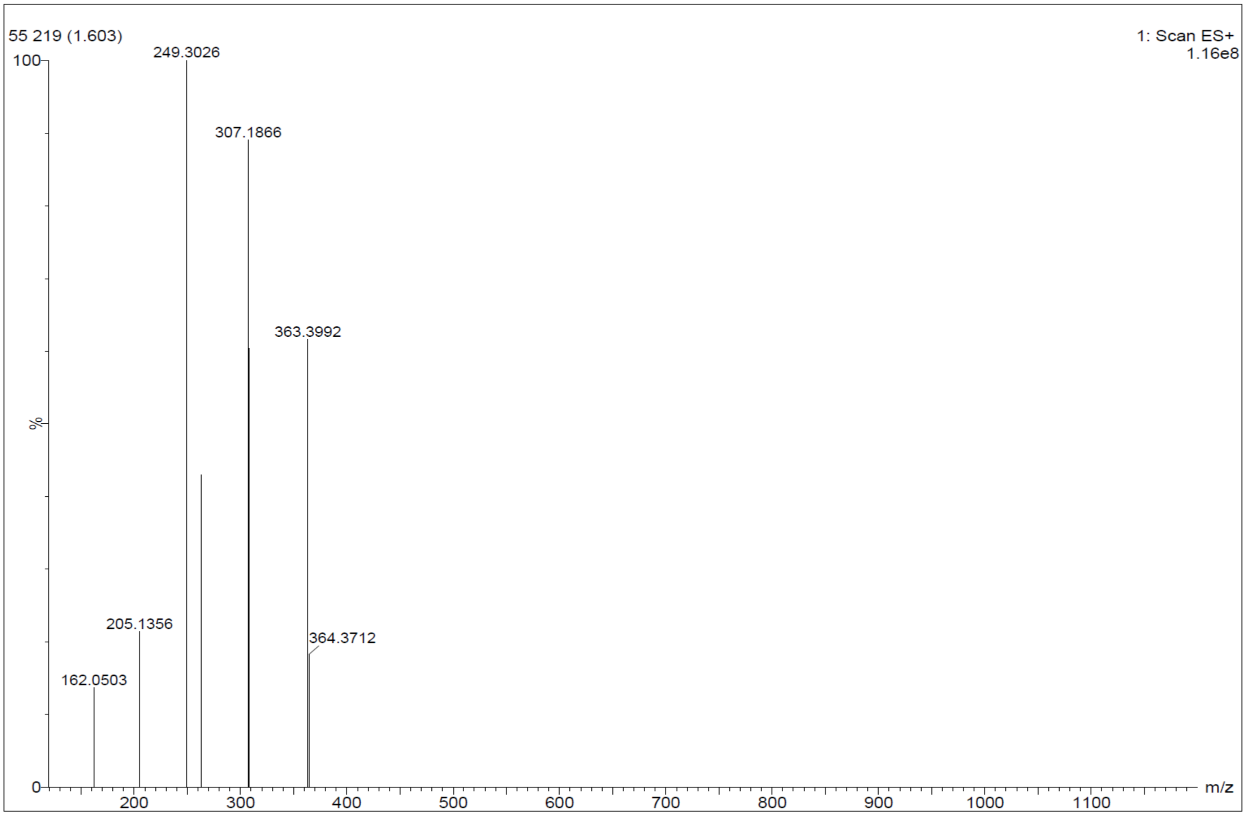
55 219 (1.603)
1: Scan ES+
1.16e8
249.3026
307.1866
363.3992
205.1356
364.3712
162.0503
m/z

tert-Butyl 3-((3R,4S,7S)-hexahydro-2H-furo[3,4-b][1,4]oxazin-3-yl)-1H-indole-1-carboxylate, Free base (4fε)

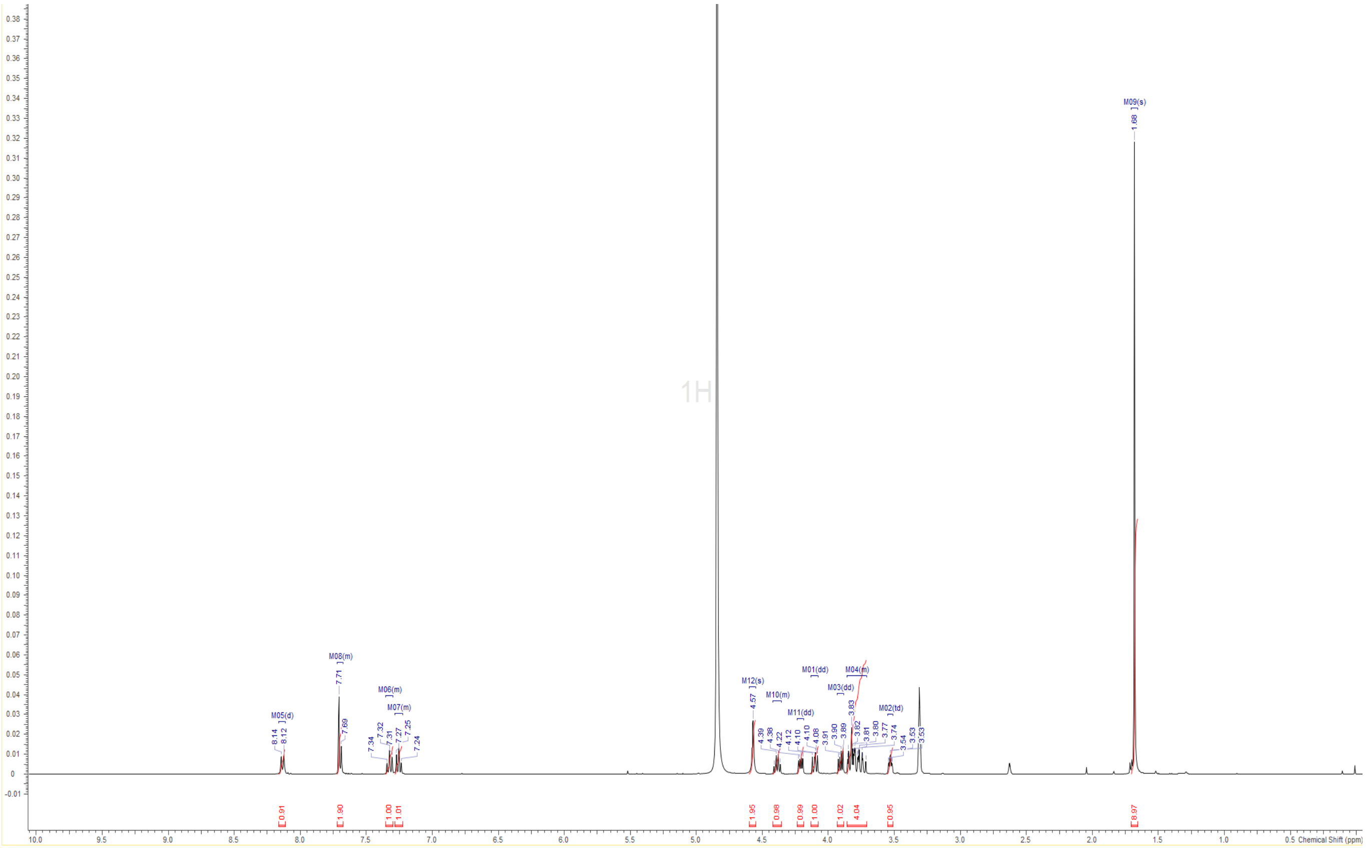

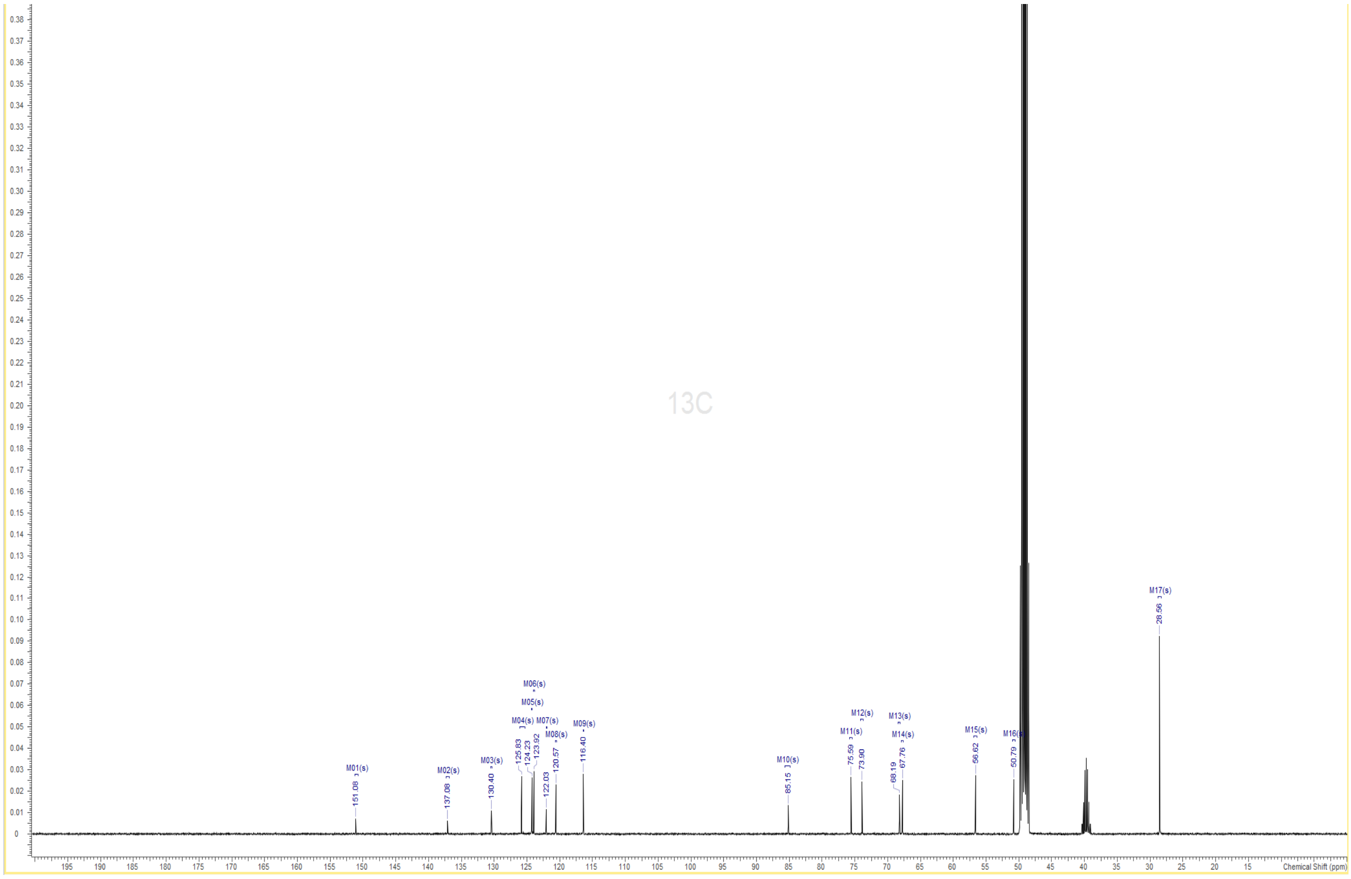

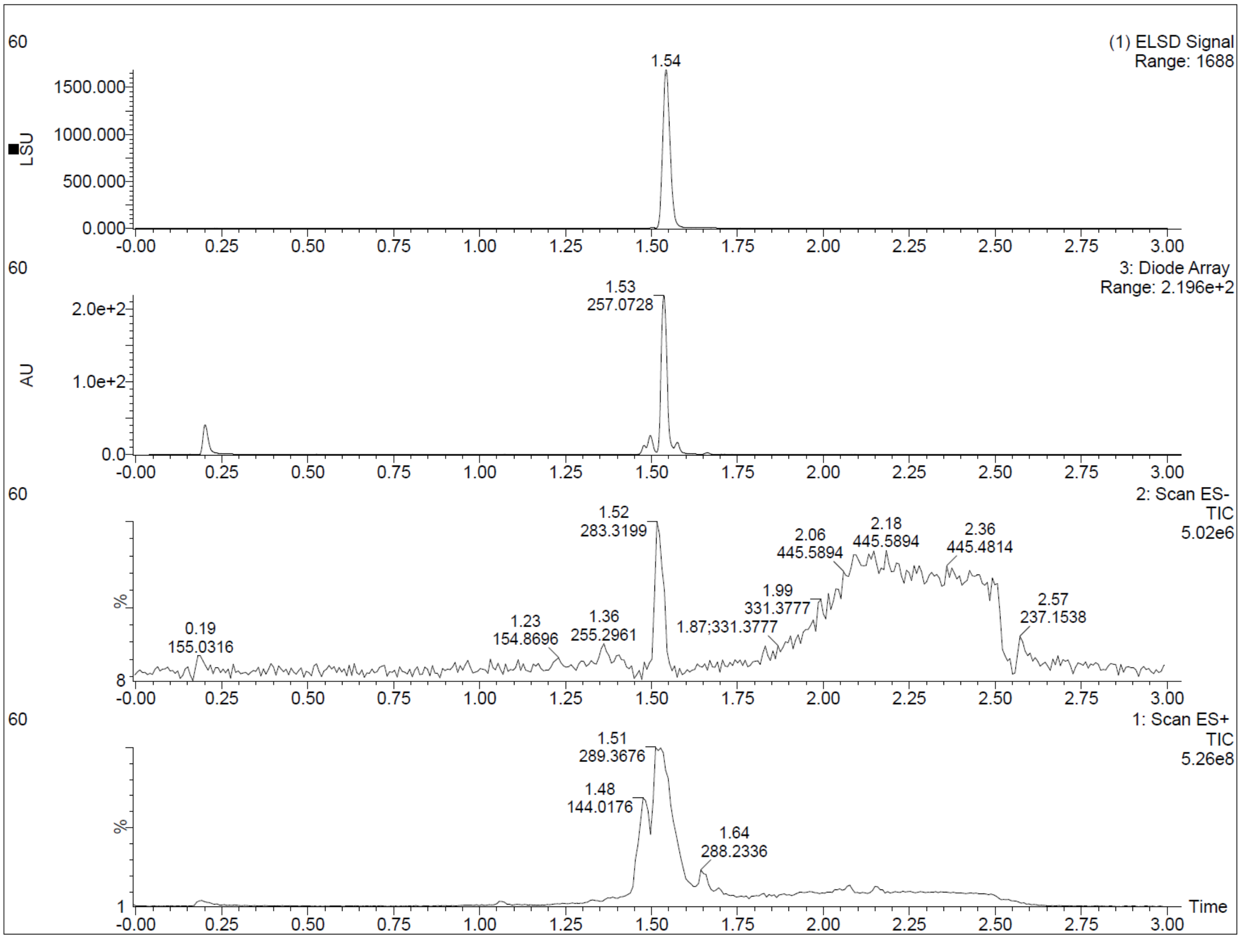

60
(1) ELSD Signal
Range: 1688
1.54
LSU
1500.000
1000.000
500.000
0.000
60
3: Diode Array
Range: 2.196e+2
1.53
257.0728
AU
2.0e+2
1.0e+2
0.0
60
2: Scan ES-
TIC
5.02e6
1.52
283.3199
2.06
445.5894
2.18
445.5894
2.36
445.4814
1.99
331.3777
1.87;331.3777
2.57
237.1538
0.19
155.0316
1.23
154.8696
1.36
255.2961
%
60
1: Scan ES+
TIC
5.26e8
1.51
289.3676
1.48
144.0176
1.64
288.2336
%
Time
-0.00 0.25 0.50 0.75 1.00 1.25 1.50 1.75 2.00 2.25 2.50 2.75 3.00

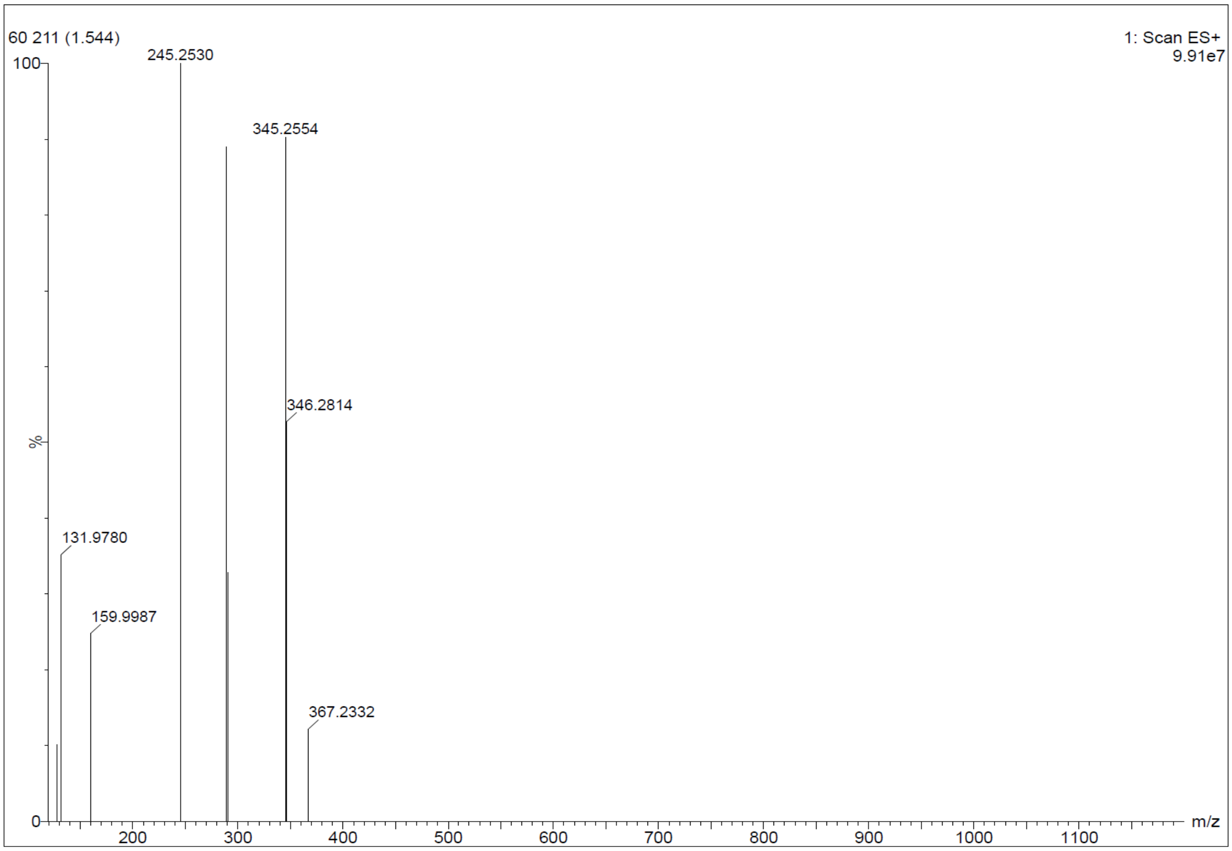

60 211 (1.544)
1: Scan ES+
9.91e7
245.2530
345.2554
346.2814
131.9780
159.9987
367.2332
%
m/z
200 300 400 500 600 700 800 900 1000 1100

(3R,4S,7R)-3-(1-Methyl-1H-pyrazol-5-yl)-6-tosyloctahydropyrrolo[3,4-b][1,4]oxazine, Free base (4hp)

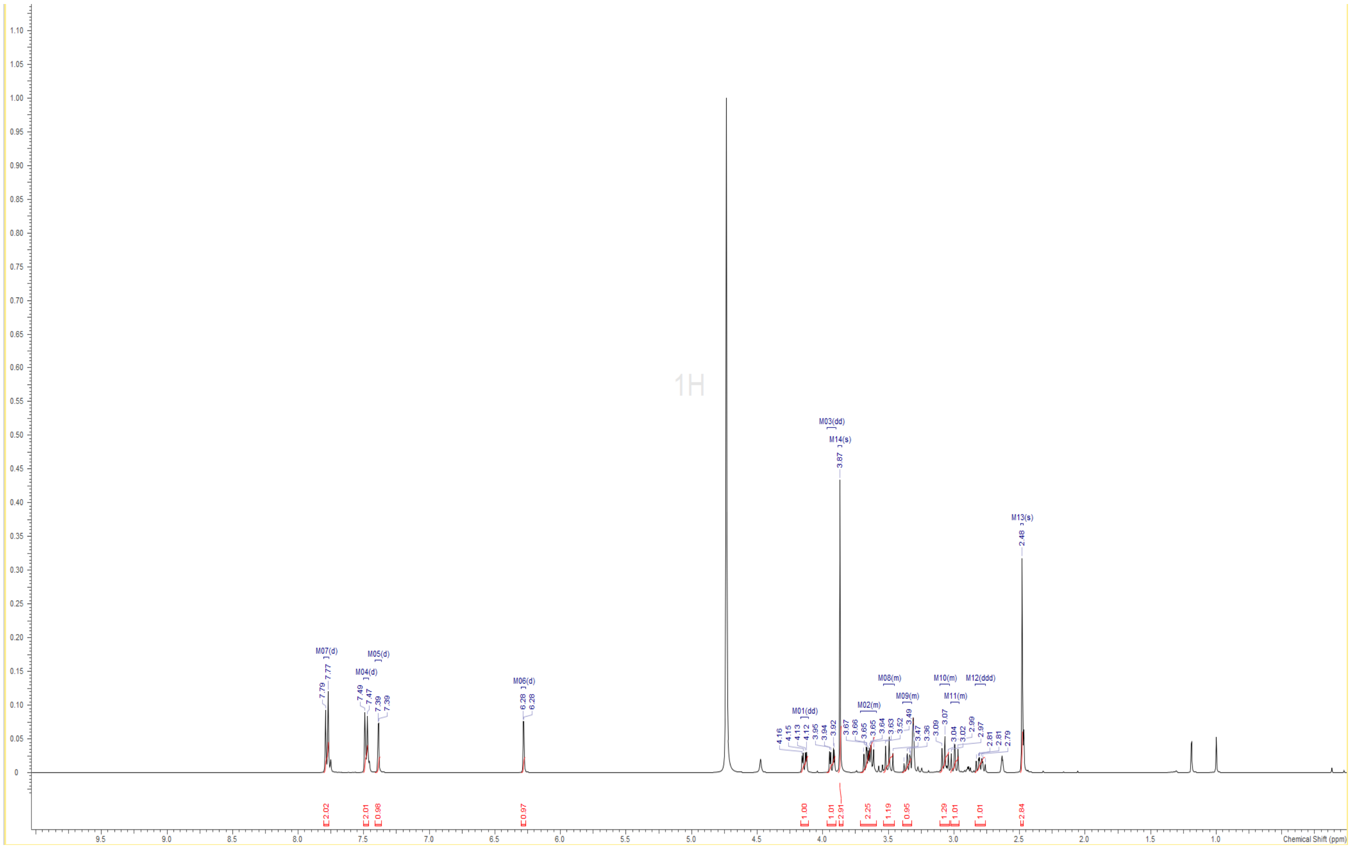

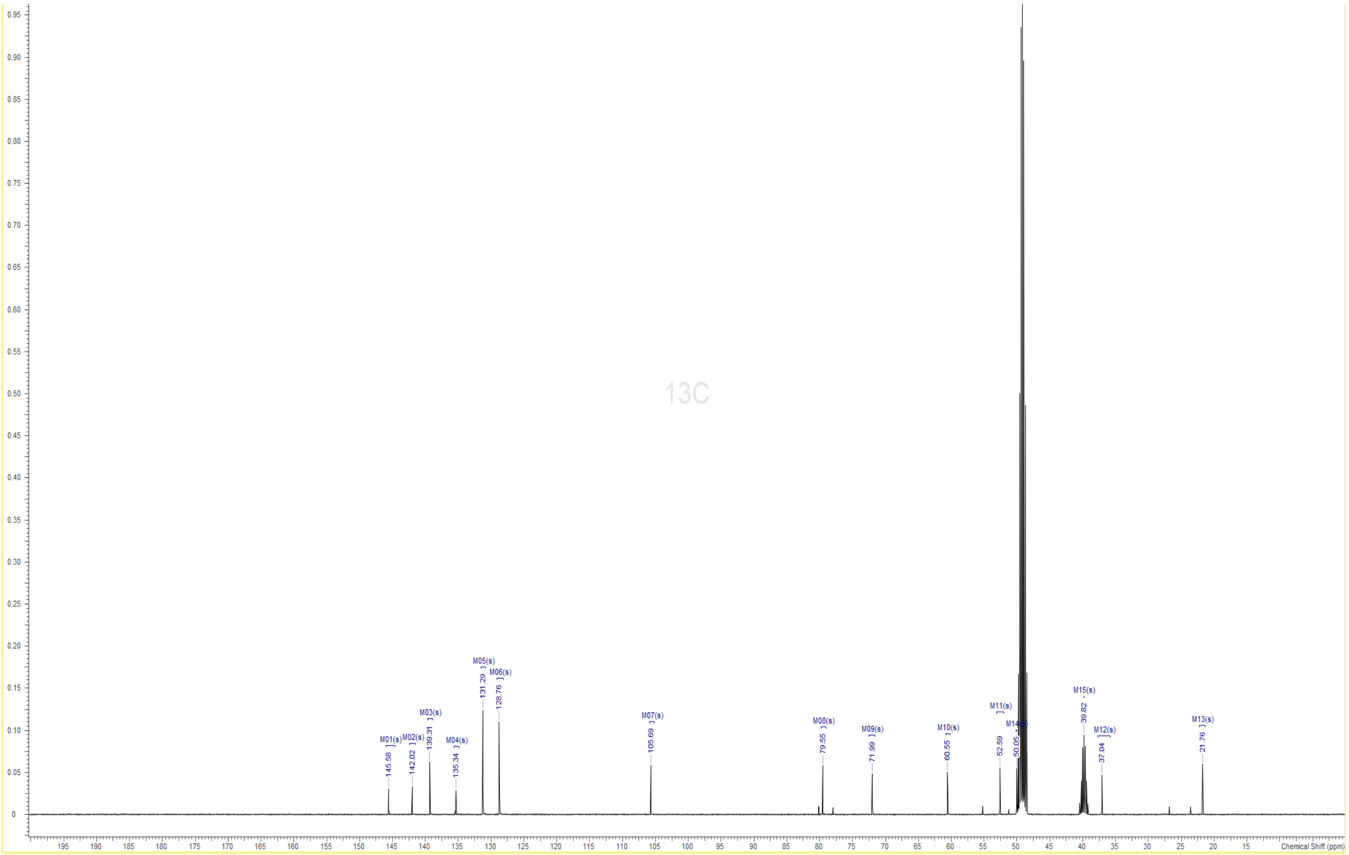

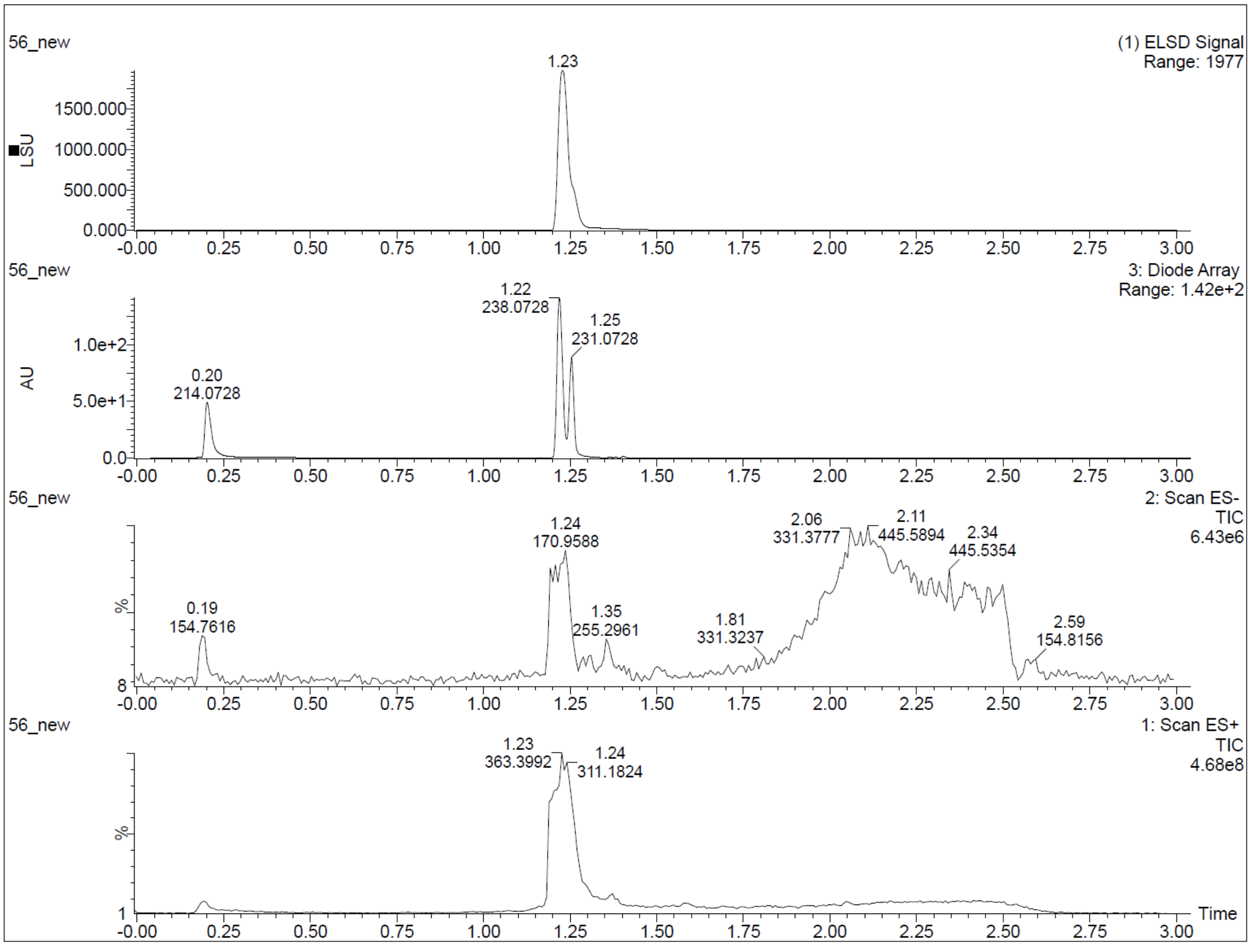
56_new
(1) ELSD Signal
Range: 1977
1.23
LSU
3: Diode Array
Range: 1.42e+2
1.22
238.0728
1.25
231.0728
0.20
214.0728
AU
2: Scan ES-
TIC
6.43e6
1.24
170.9588
2.06
331.3777
2.11
445.5894
2.34
445.5354
0.19
154.7616
1.35
255.2961
1.81
331.3237
2.59
154.8156
1: Scan ES+
TIC
4.68e8
1.23
363.3992
1.24
311.1824
Time

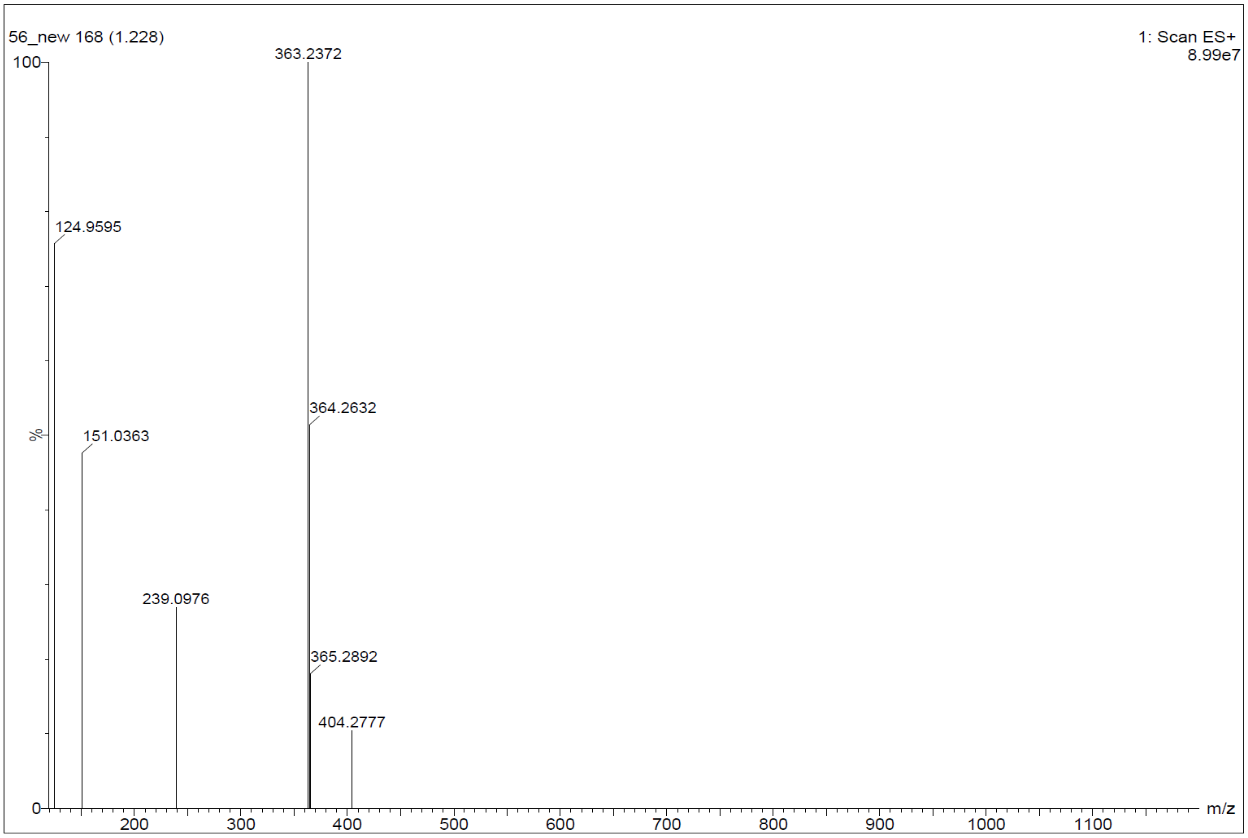
56_new 168 (1.228)
1: Scan ES+
8.99e7
363.2372
124.9595
364.2632
151.0363
239.0976
365.2892
404.2777
m/z

(3R,4S,7S)-3-(1-Methyl-1H-indol-2-yl)-6-tosyloctahydropyrrolo[3,4-b][1,4]oxazine, Free base (4iz)

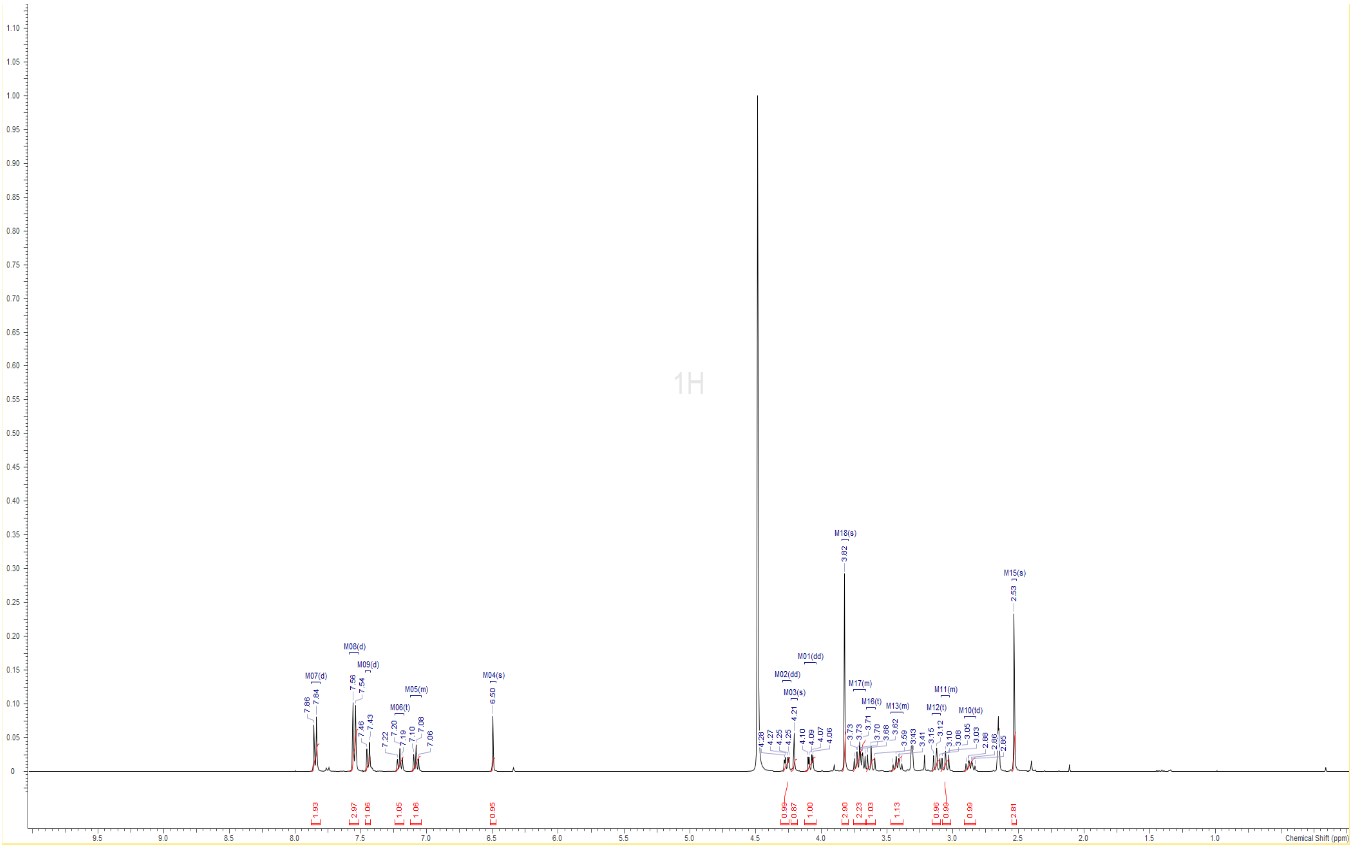

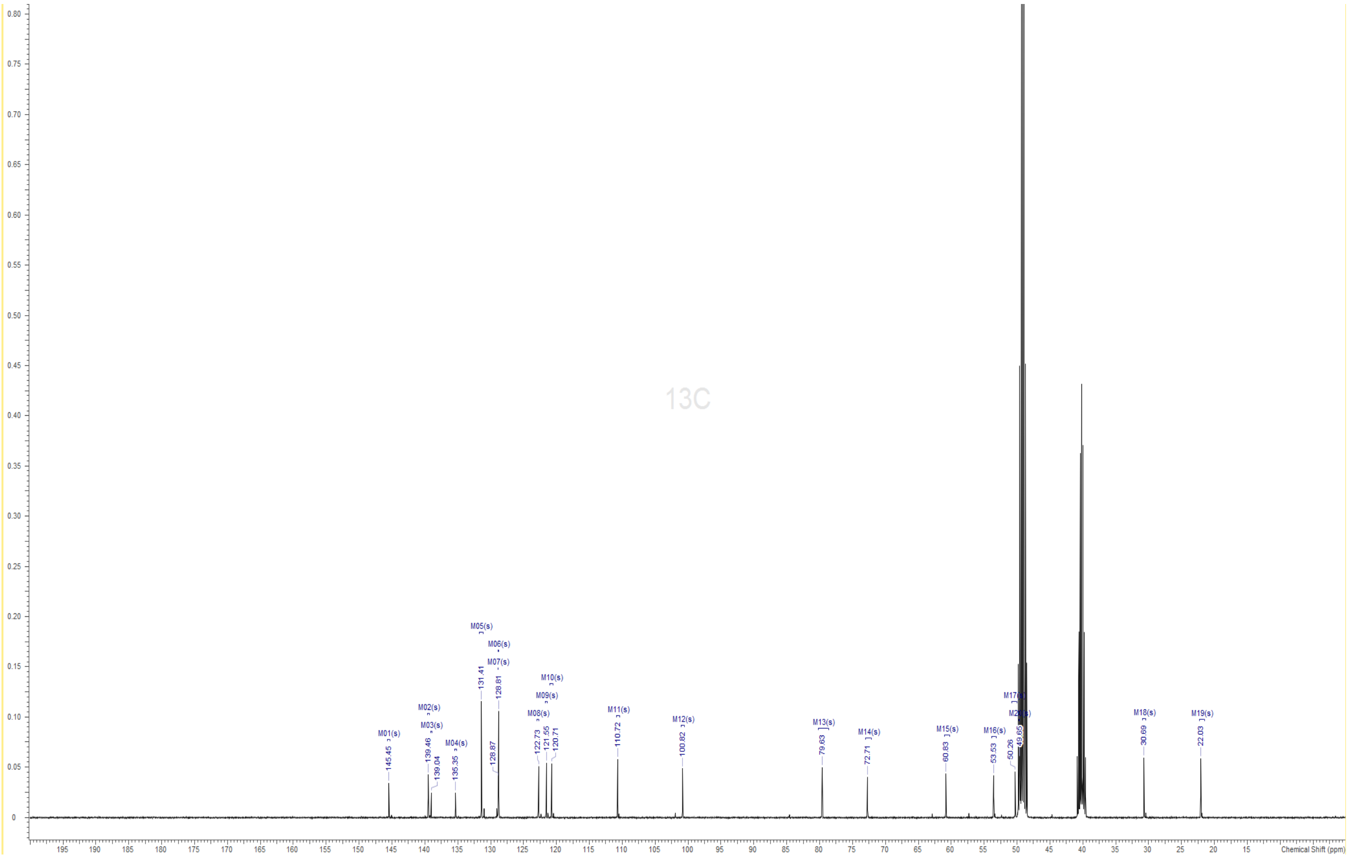

tert-Butyl 4-((3R,6S)-6-methylthiomorpholin-3-yl)indoline-1-carboxylate, Free base (4ll)

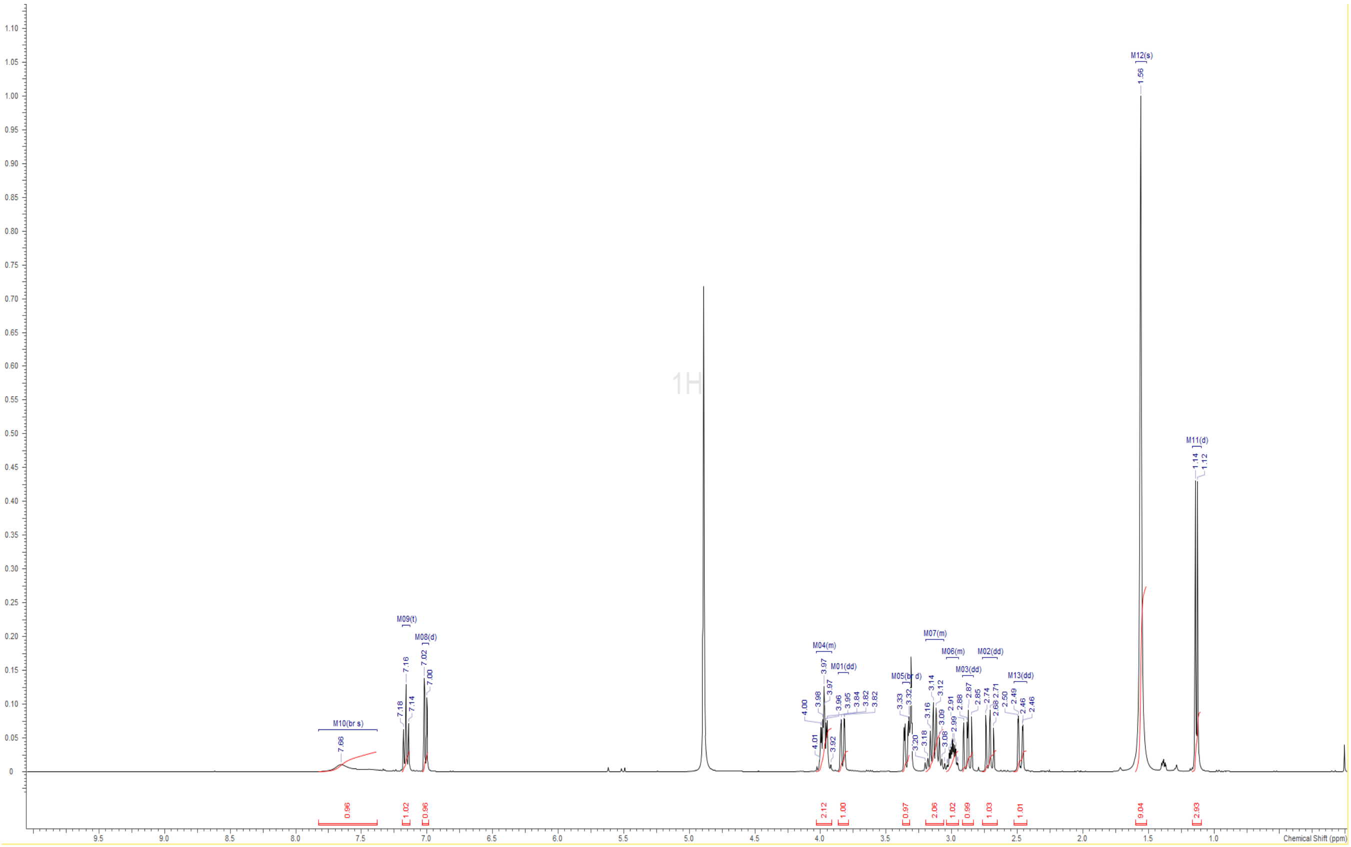

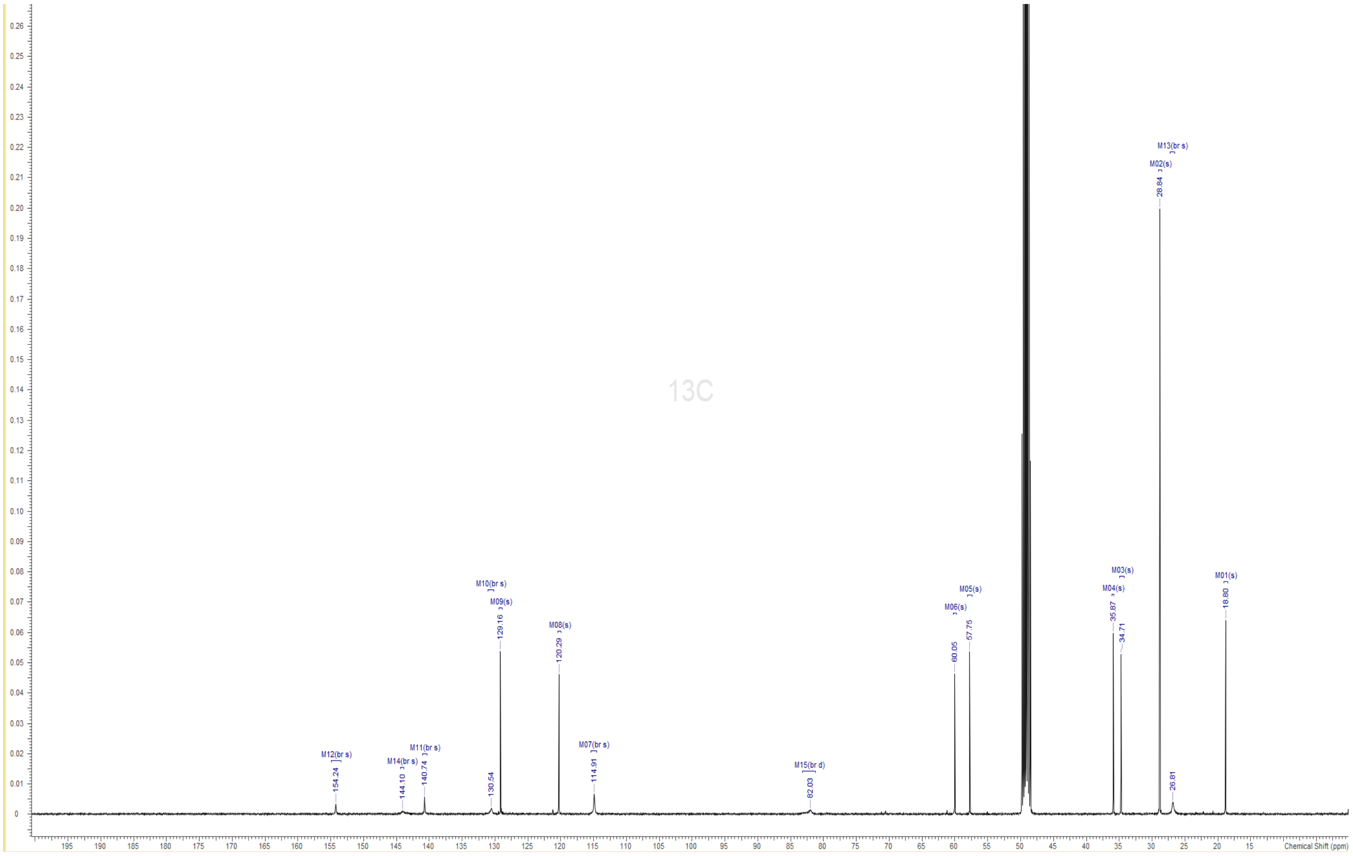

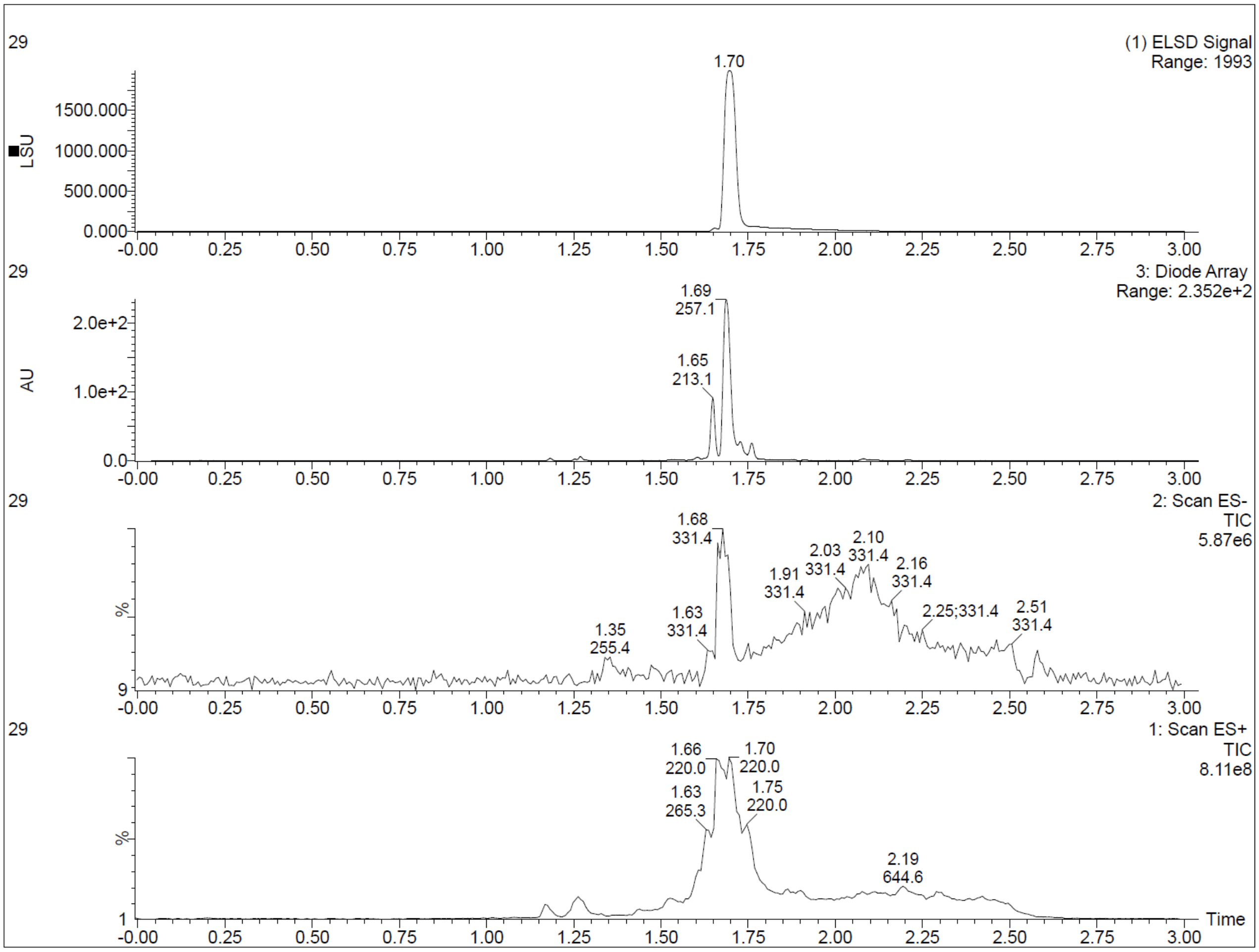

29
(1) ELSD Signal
Range: 1993
1.70
LSU
3: Diode Array
Range: 2.352e+2
1.69
257.1
1.65
213.1
AU
2: Scan ES-
TIC
5.87e6
1.68
331.4
1: Scan ES+
TIC
8.11e8
2.19
644.6
Time

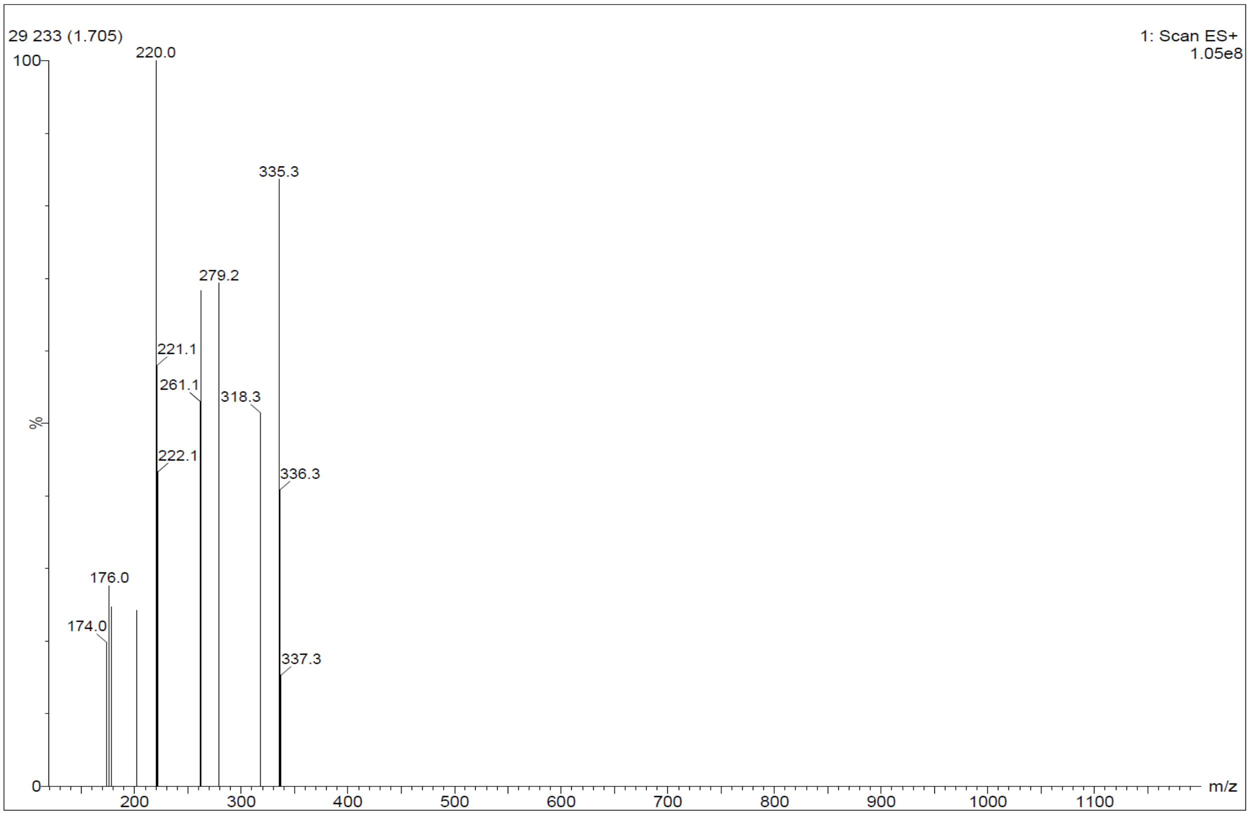

29 233 (1.705)
1: Scan ES+
1.05e8
220.0
335.3
279.2
221.1
261.1
318.3
222.1
336.3
176.0
174.0
337.3
m/z

(2S,5R)-5-(6-(4-Methoxyphenyl)pyridin-2-yl)-2-methylthiomorpholine, Free base (4lγ)

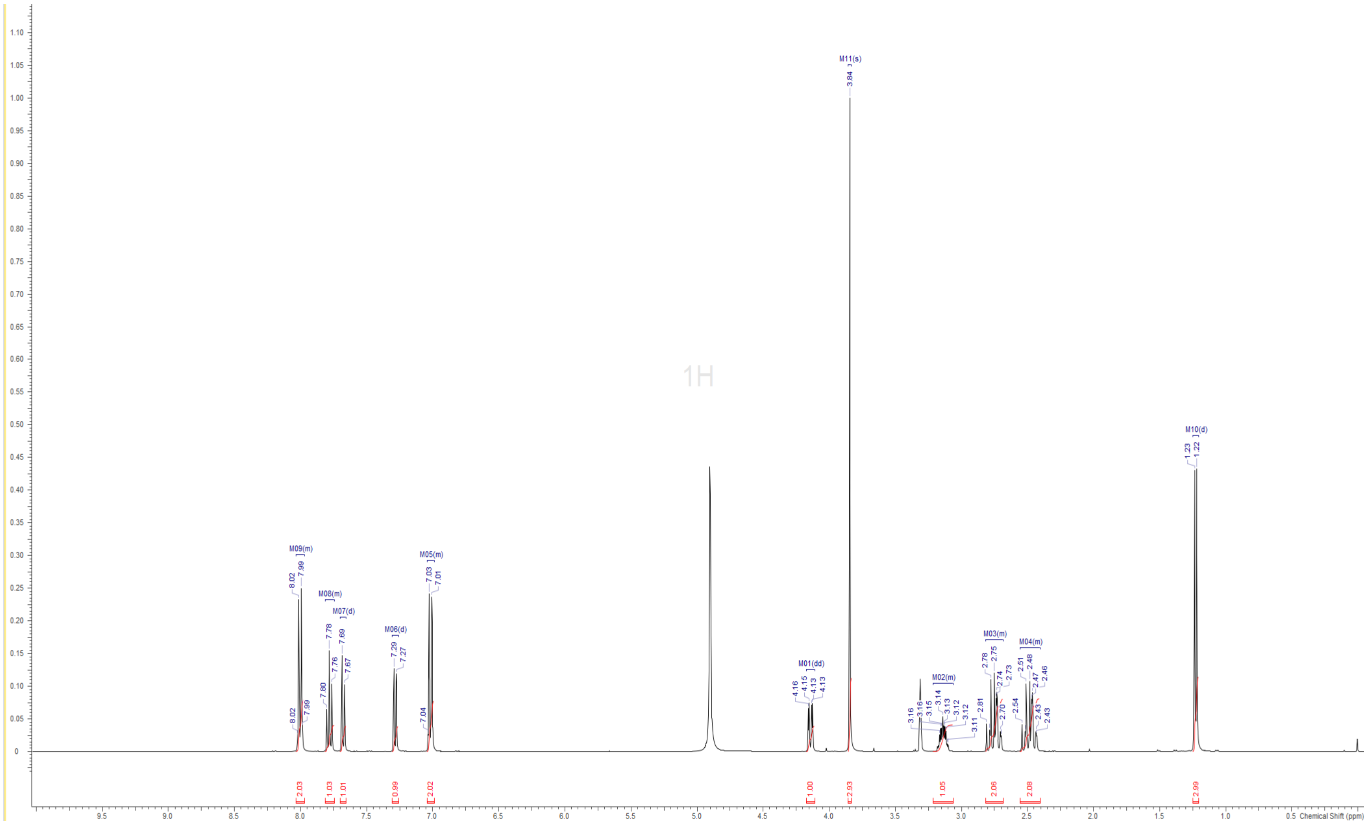

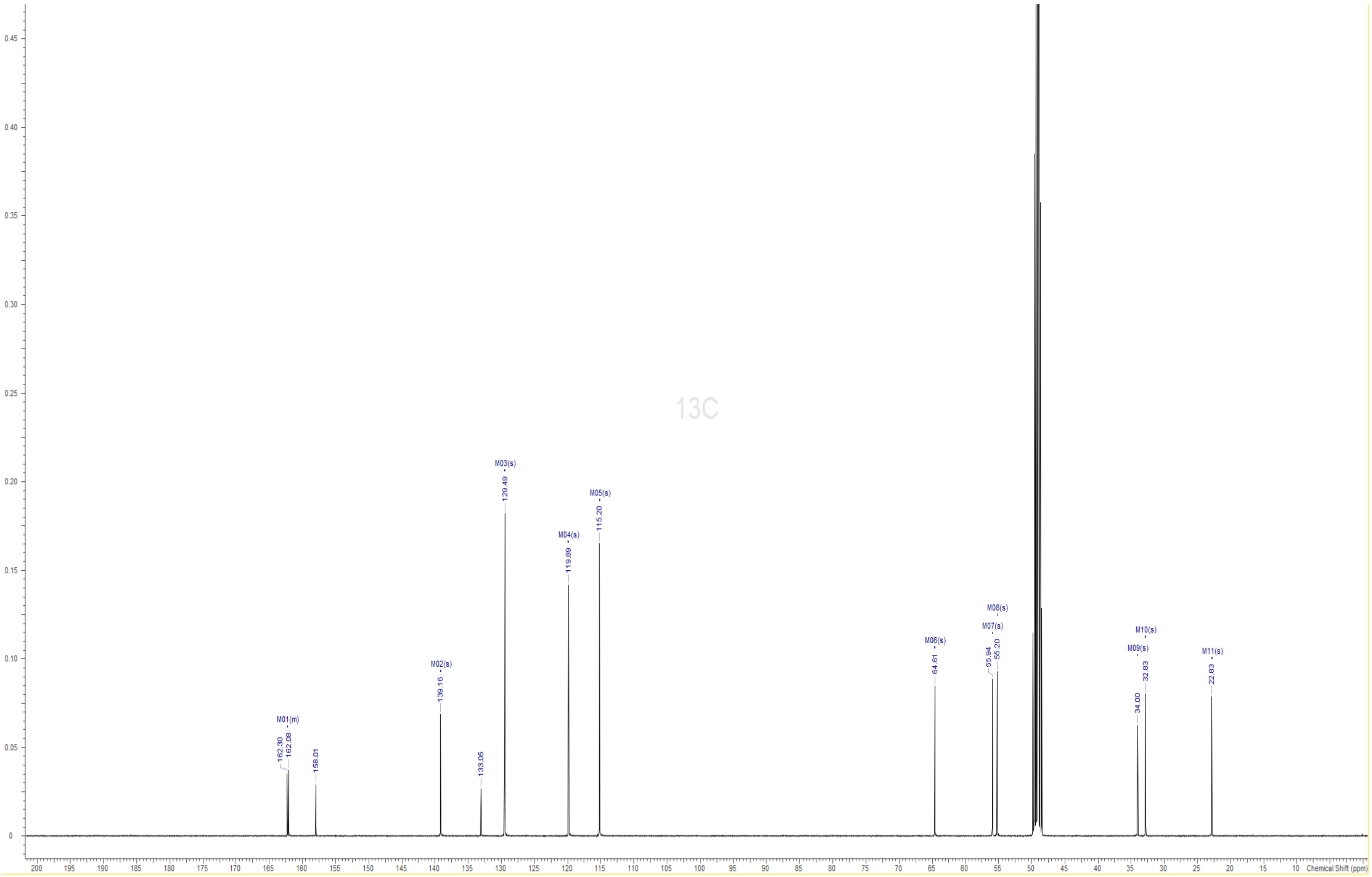

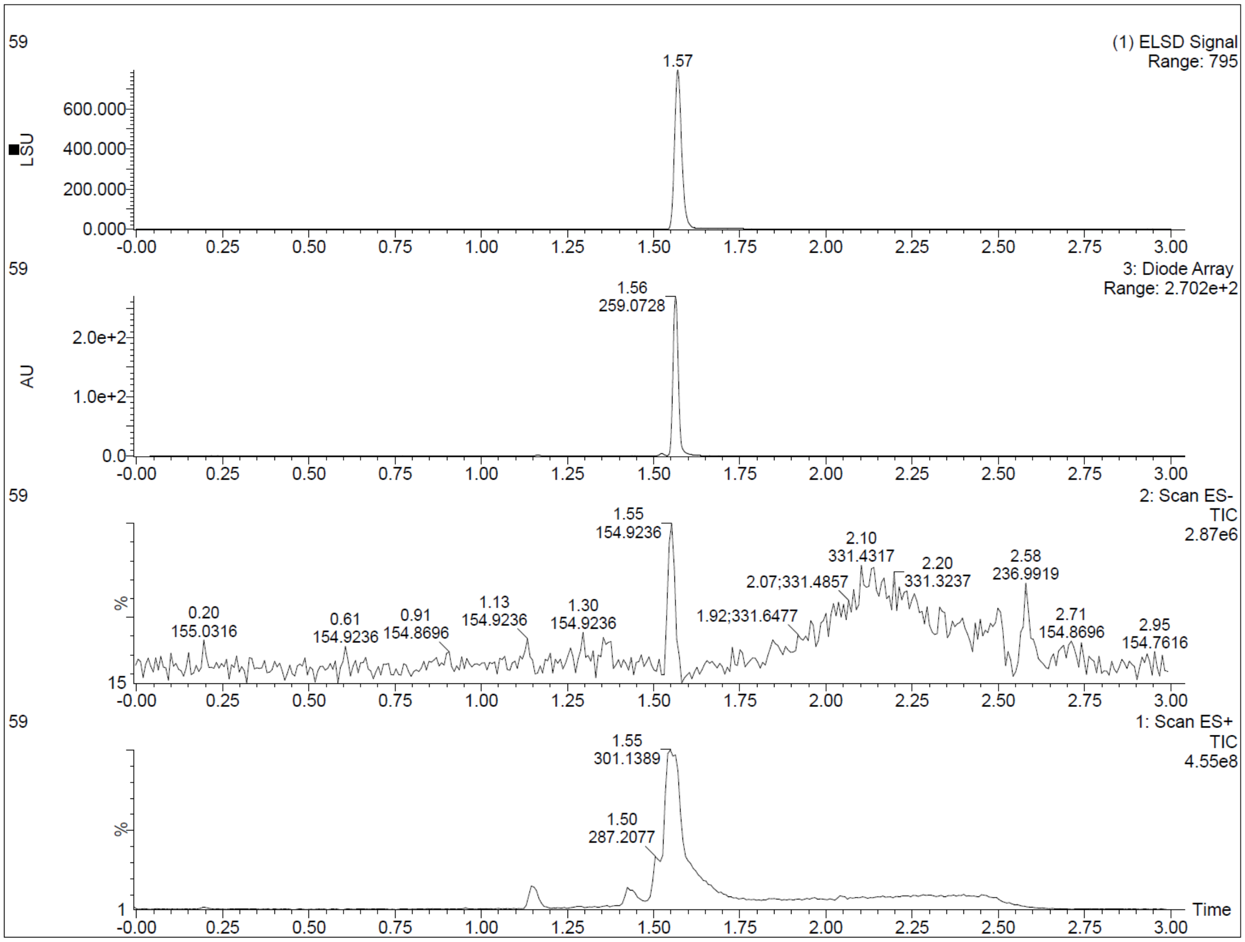
59
(1) ELSD Signal
Range: 795
1.57
LSU
600.000
400.000
200.000
0.000
3: Diode Array
Range: 2.702e+2
1.56
259.0728
AU
2.0e+2
1.0e+2
0.0
2: Scan ES-
TIC
2.87e6
1.55
154.9236
0.20
155.0316
0.61
154.9236
0.91
154.8696
1.13
154.9236
1.30
154.9236
1.92;331.6477
2.07;331.4857
2.10
331.4317
2.20
331.3237
2.58
236.9919
2.71
154.8696
2.95
154.7616
15
1: Scan ES+
TIC
4.55e8
1.55
301.1389
1.50
287.2077
%
1
Time
-0.00
0.25
0.50
0.75
1.00
1.25
1.50
1.75
2.00
2.25
2.50
2.75
3.00

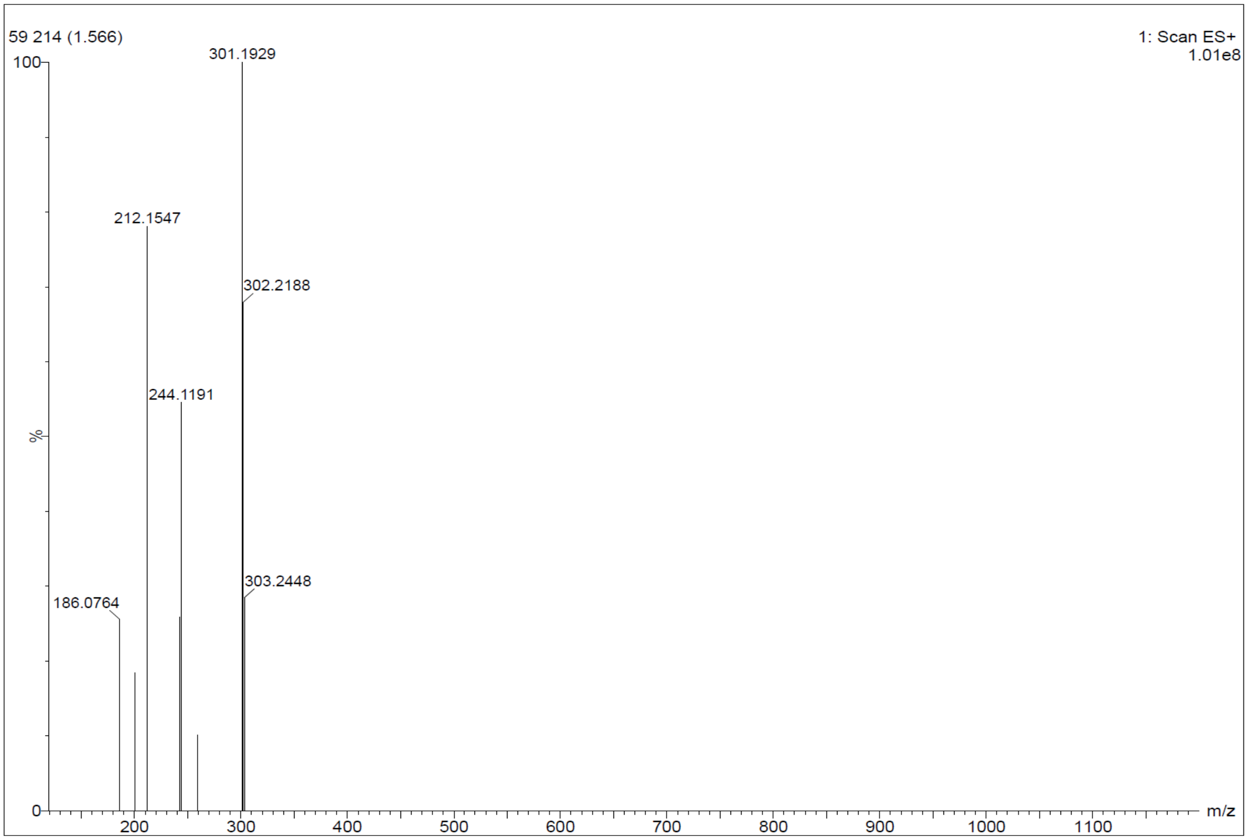
59 214 (1.566)
1: Scan ES+
1.01e8
100
301.1929
212.1547
302.2188
244.1191
%
186.0764
303.2448
0
m/z
200
300
400
500
600
700
800
900
1000
1100

Benzyl 3-(5-bromopyridin-3-yl)piperazine-1-carboxylate, Free base (4mr)

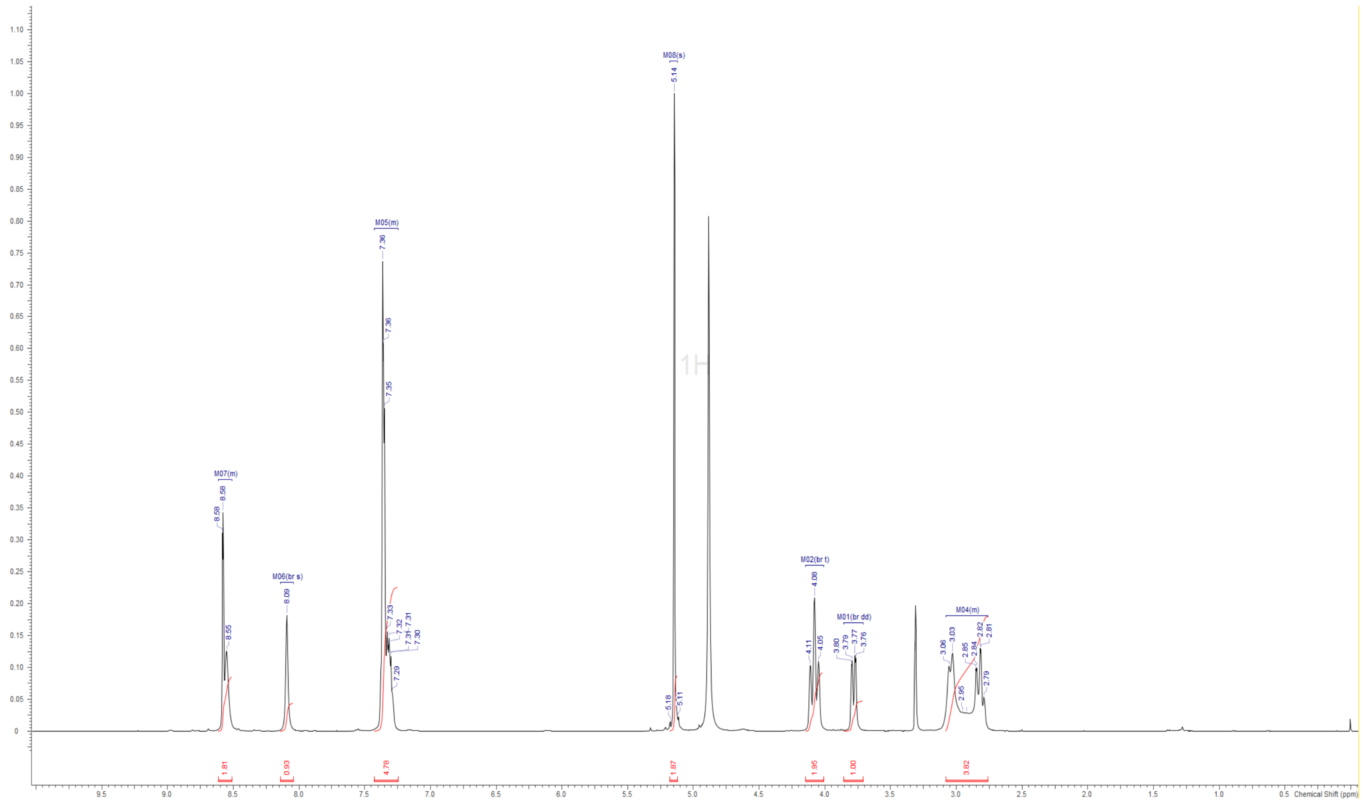

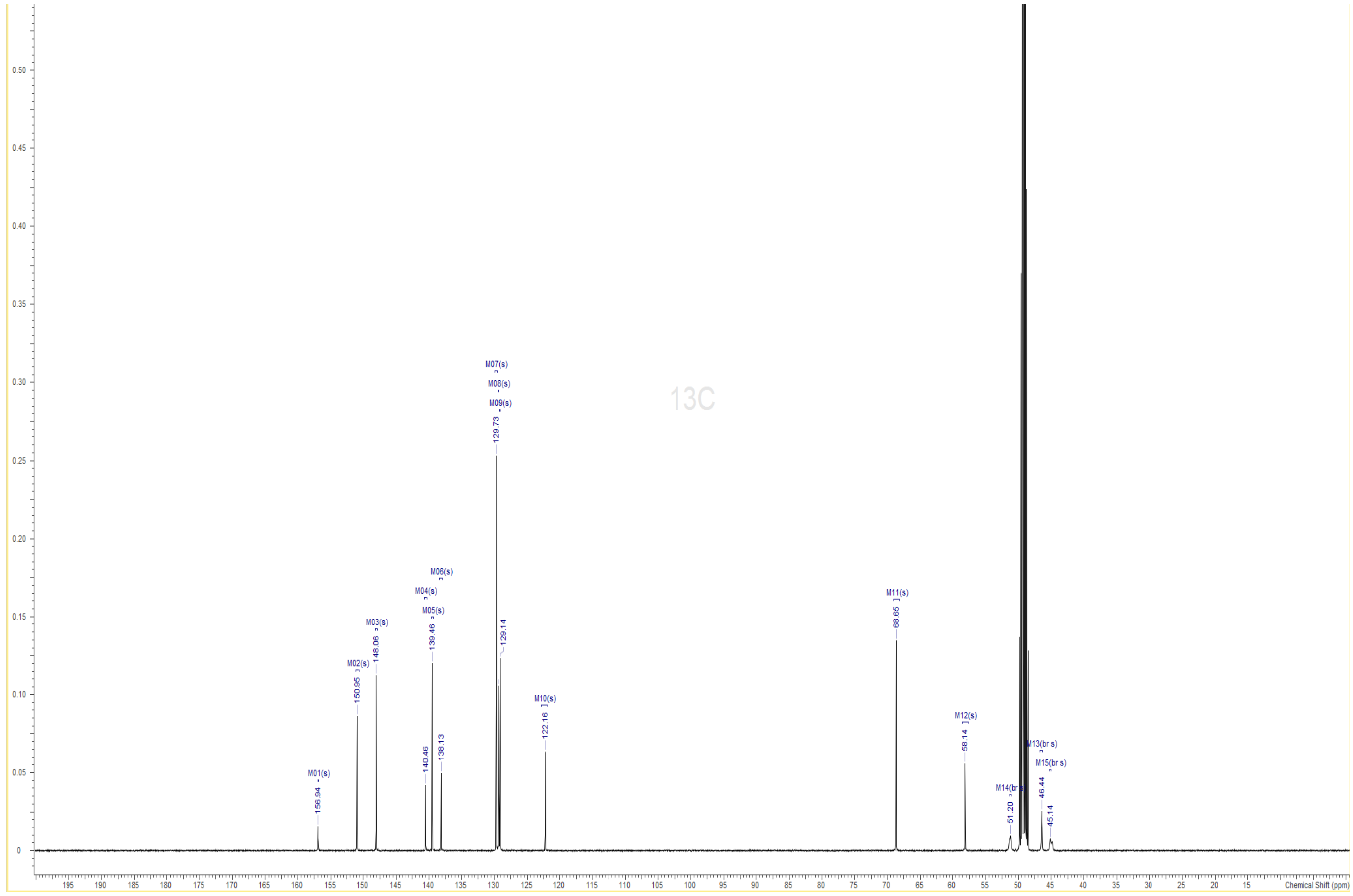

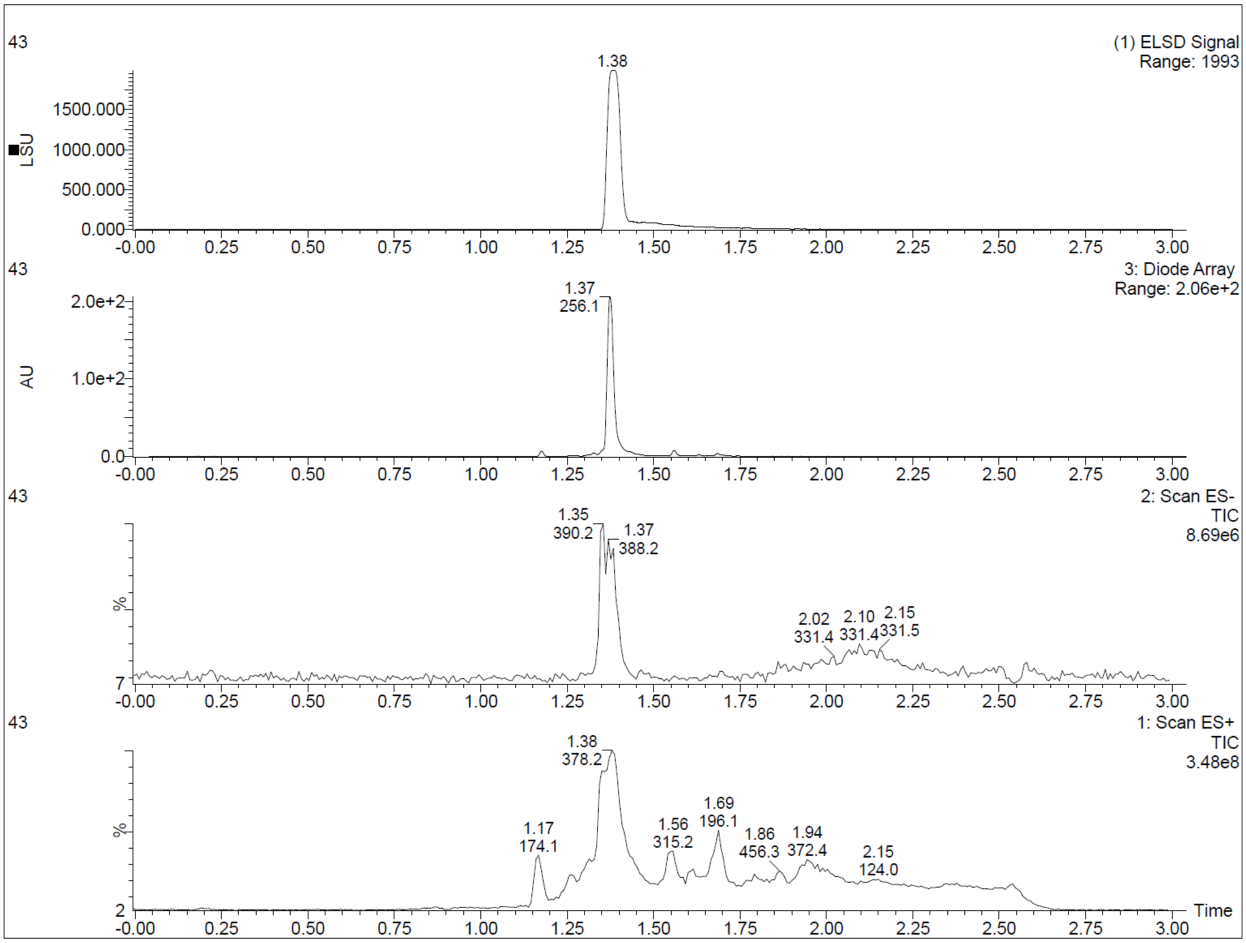
43
(1) ELSD Signal
Range: 1993
1.38
LSU
1500.000
1000.000
500.000
0.000
43
3: Diode Array
Range: 2.06e+2
1.37
256.1
AU
2.0e+2
1.0e+2
0.0
43
2: Scan ES-
TIC
8.69e6
1.35
390.2
1.37
388.2
2.02
331.4
2.10
331.4
2.15
331.5
%
7
43
1: Scan ES+
TIC
3.48e8
1.38
378.2
1.17
174.1
1.56
315.2
1.69
196.1
1.86
456.3
1.94
372.4
2.15
124.0
%
2
Time
-0.00
0.25
0.50
0.75
1.00
1.25
1.50
1.75
2.00
2.25
2.50
2.75
3.00

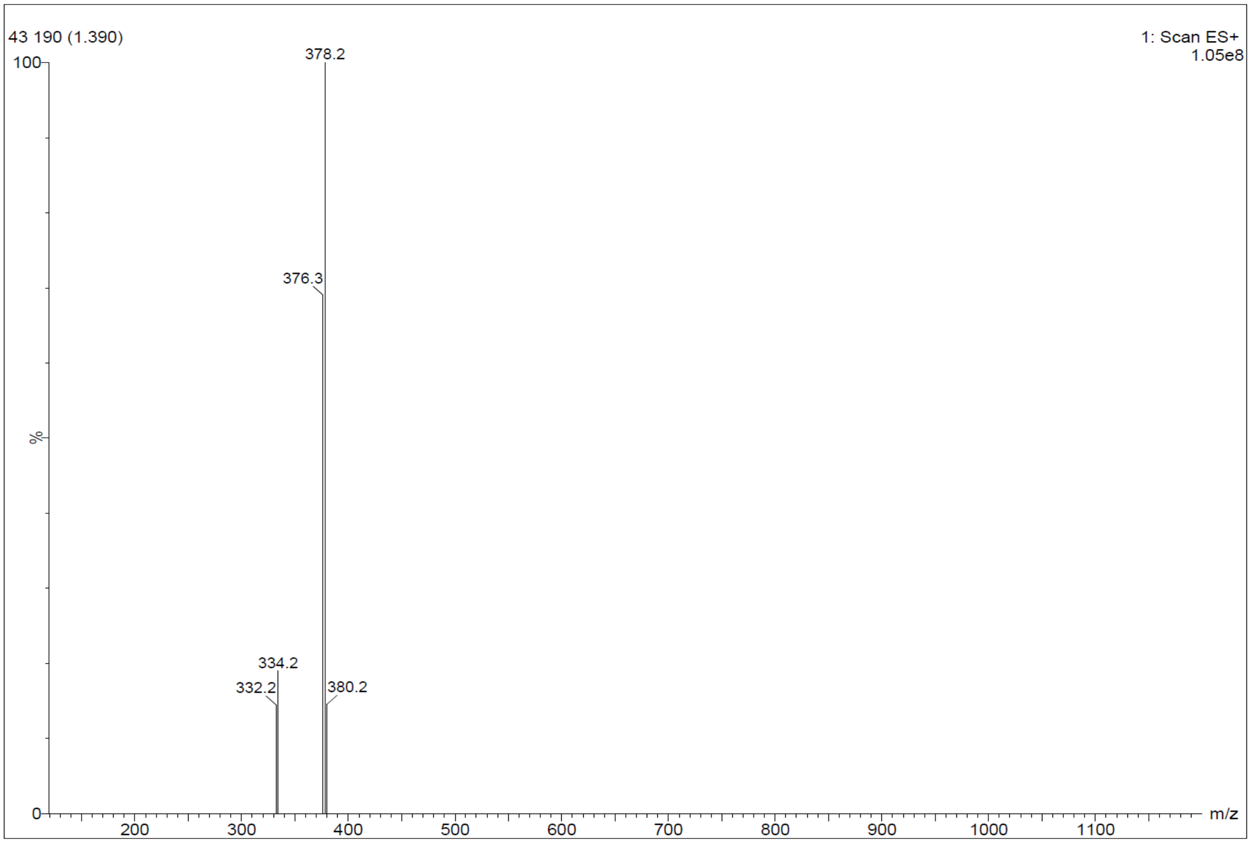
43 190 (1.390)
1: Scan ES+
1.05e8
100
378.2
376.3
%
334.2
332.2
380.2
0
200
300
400
500
600
700
800
900
1000
1100
m/z

Benzyl 3-cyclopropylpiperazine-1-carboxylate, Free base (4mα)

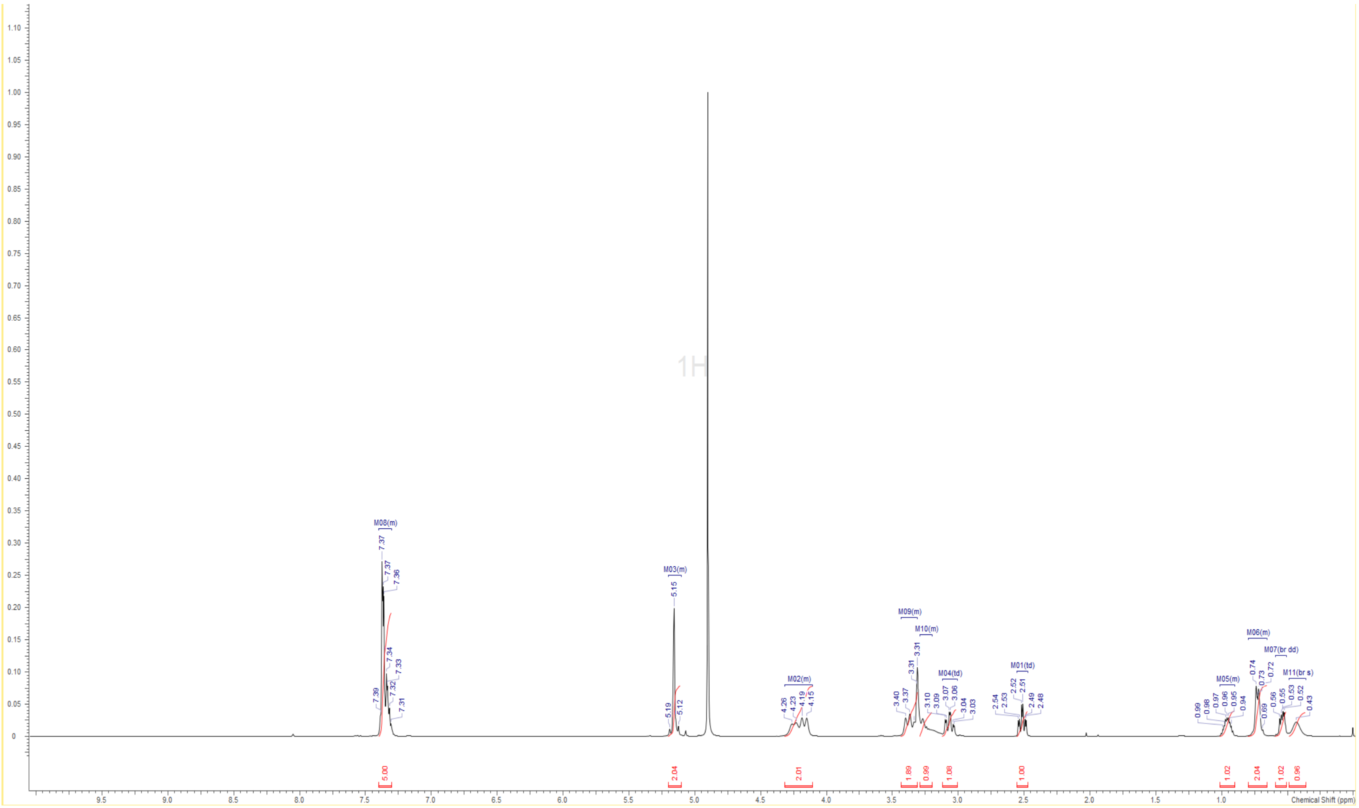

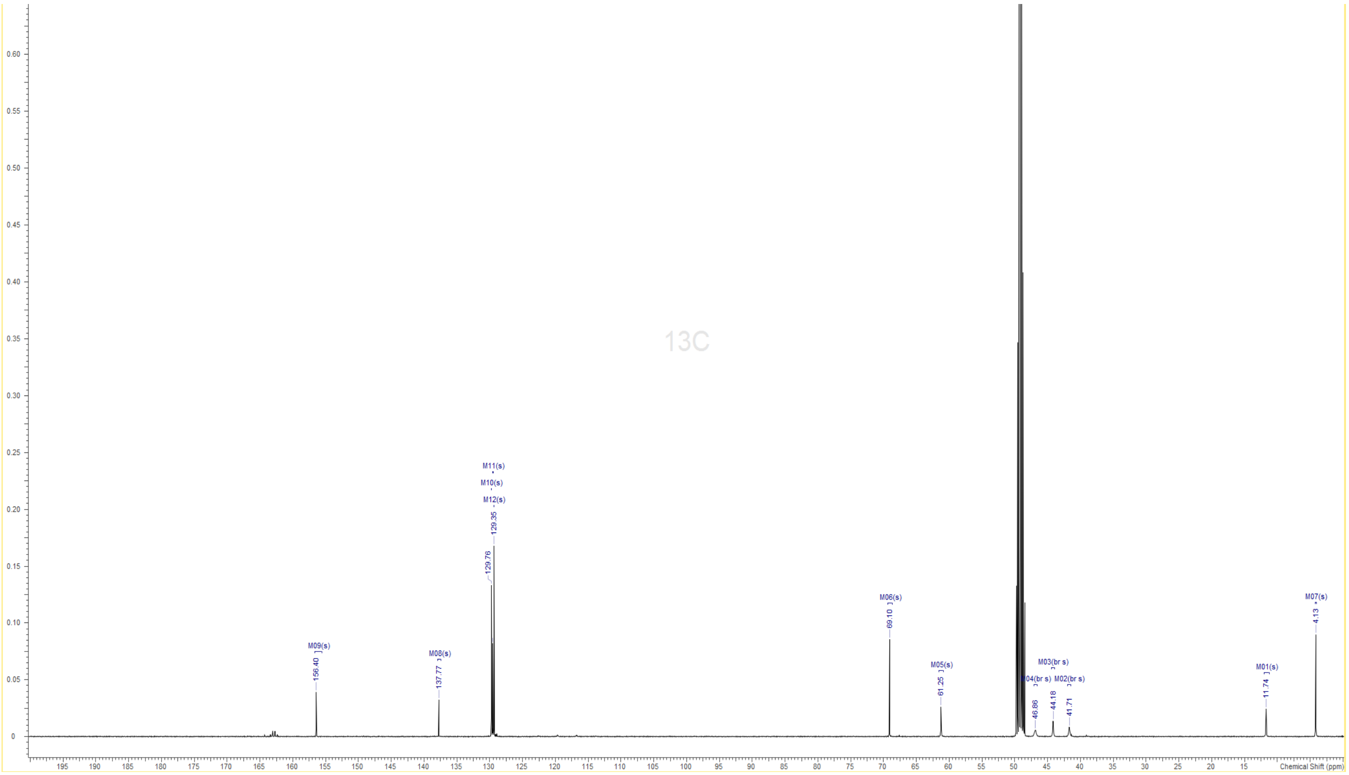

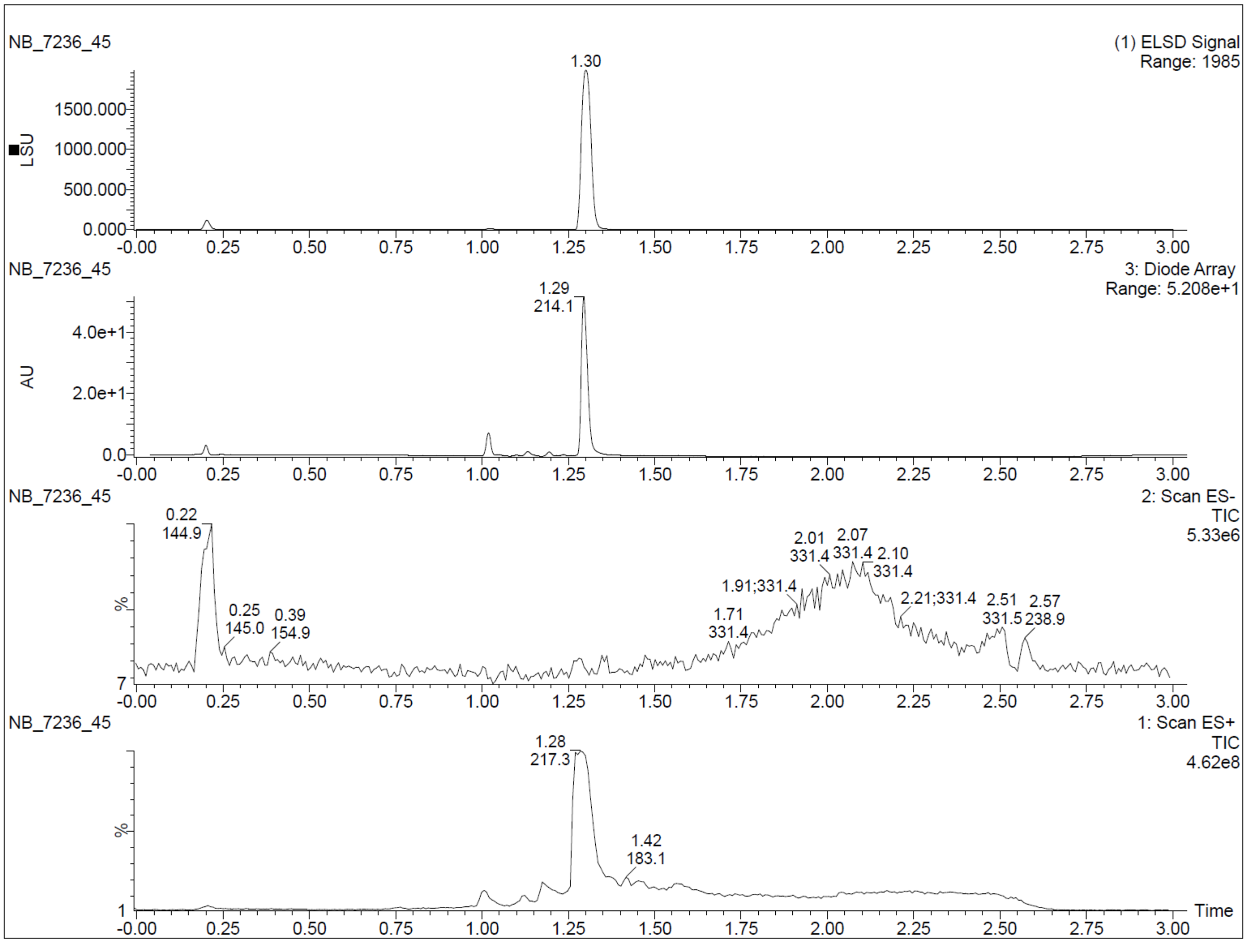

NB_7236_45
(1) ELSD Signal
Range: 1985
1.30
LSU
1500.000
1000.000
500.000
0.000
NB_7236_45
3: Diode Array
Range: 5.208e+1
1.29
214.1
AU
4.0e+1
2.0e+1
0.0
NB_7236_45
2: Scan ES-
TIC
5.33e6
0.22
144.9
0.25
145.0
0.39
154.9
1.71
331.4
1.91;331.4
2.01
331.4
2.07
331.4
2.10
331.4
2.21;331.4
2.51
331.5
2.57
238.9
NB_7236_45
1: Scan ES+
TIC
4.62e8
1.28
217.3
1.42
183.1
Time
-0.00 0.25 0.50 0.75 1.00 1.25 1.50 1.75 2.00 2.25 2.50 2.75 3.00

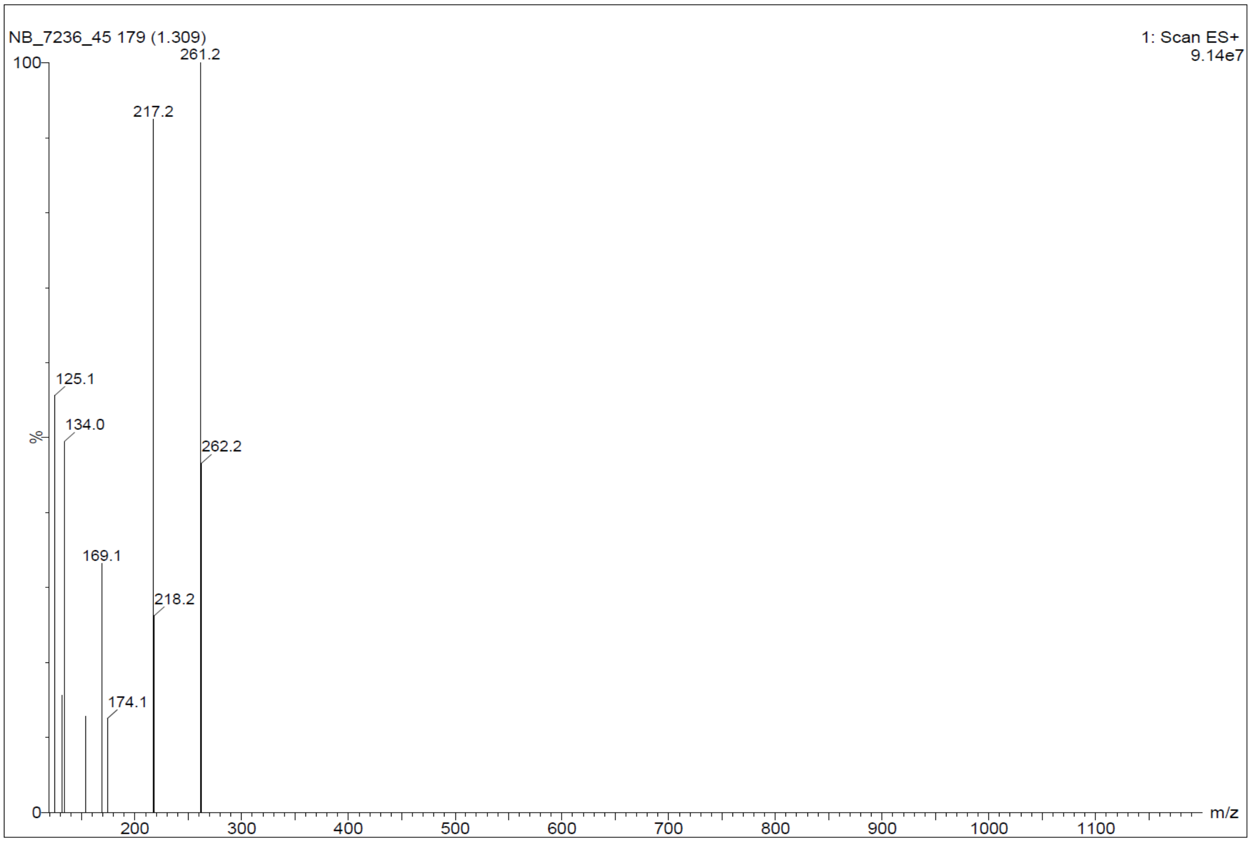

NB_7236_45 179 (1.309)
1: Scan ES+
9.14e7
261.2
217.2
125.1
134.0
262.2
169.1
218.2
174.1
m/z
200 300 400 500 600 700 800 900 1000 1100

Benzyl 3-(3-phenylisoxazol-5-yl)piperazine-1-carboxylate, Free base (4mx)

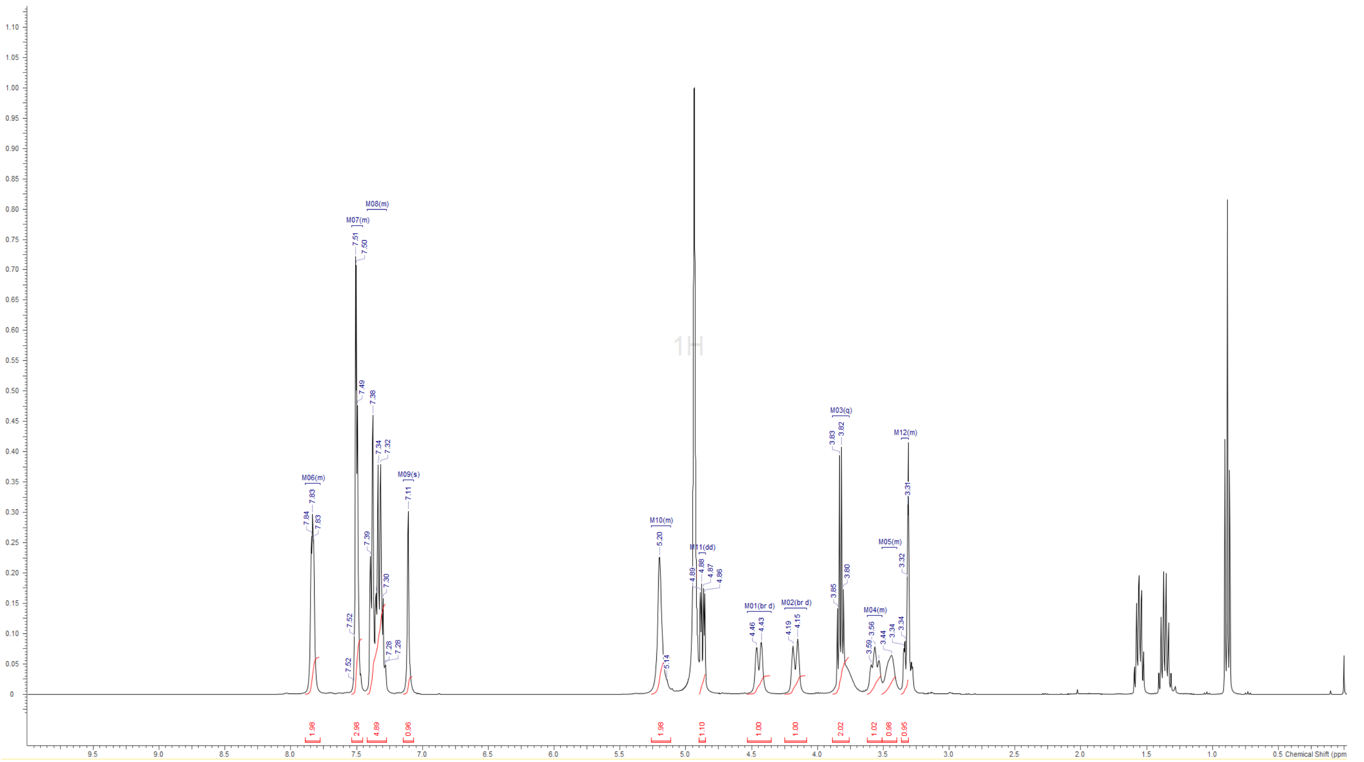

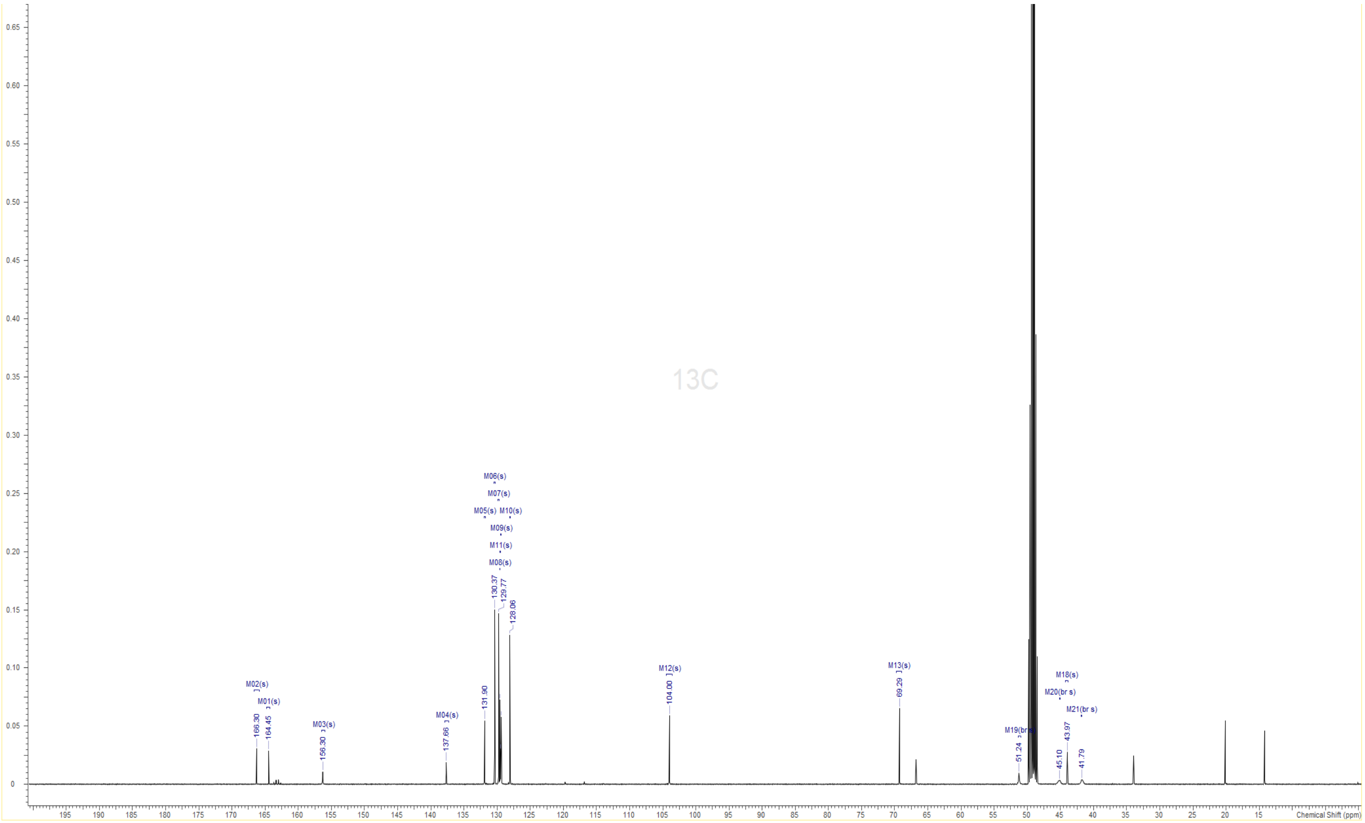

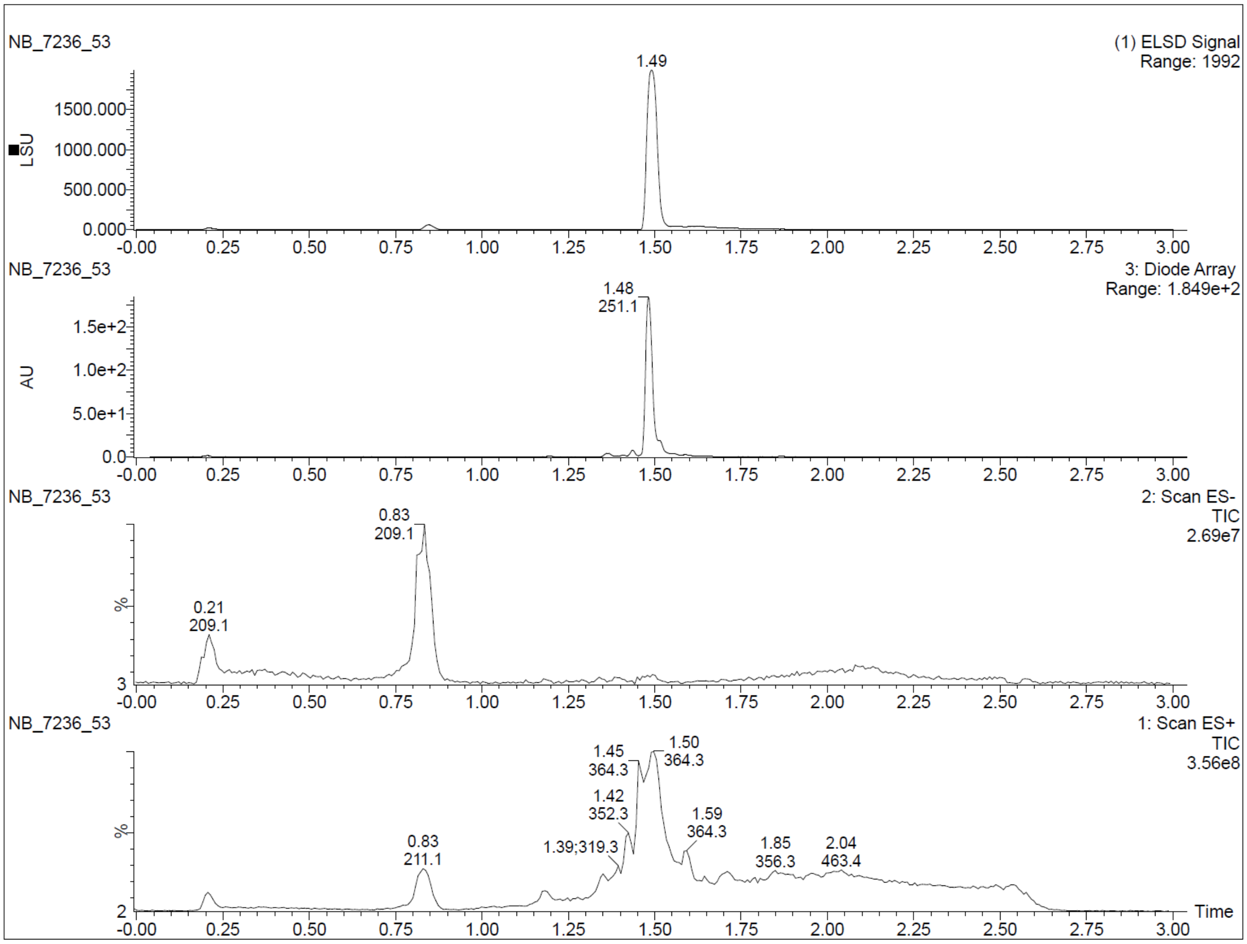
NB_7236_53
(1) ELSD Signal
Range: 1992
1.49
LSU
1500.000
1000.000
500.000
0.000
NB_7236_53
3: Diode Array
Range: 1.849e+2
1.48
251.1
AU
1.5e+2
1.0e+2
5.0e+1
0.0
NB_7236_53
2: Scan ES-
TIC
2.69e7
0.83
209.1
0.21
209.1
%
3
NB_7236_53
1: Scan ES+
TIC
3.56e8
1.45
364.3
1.50
364.3
1.42
352.3
1.59
364.3
1.39;319.3
0.83
211.1
1.85
356.3
2.04
463.4
%
2
Time
-0.00 0.25 0.50 0.75 1.00 1.25 1.50 1.75 2.00 2.25 2.50 2.75 3.00

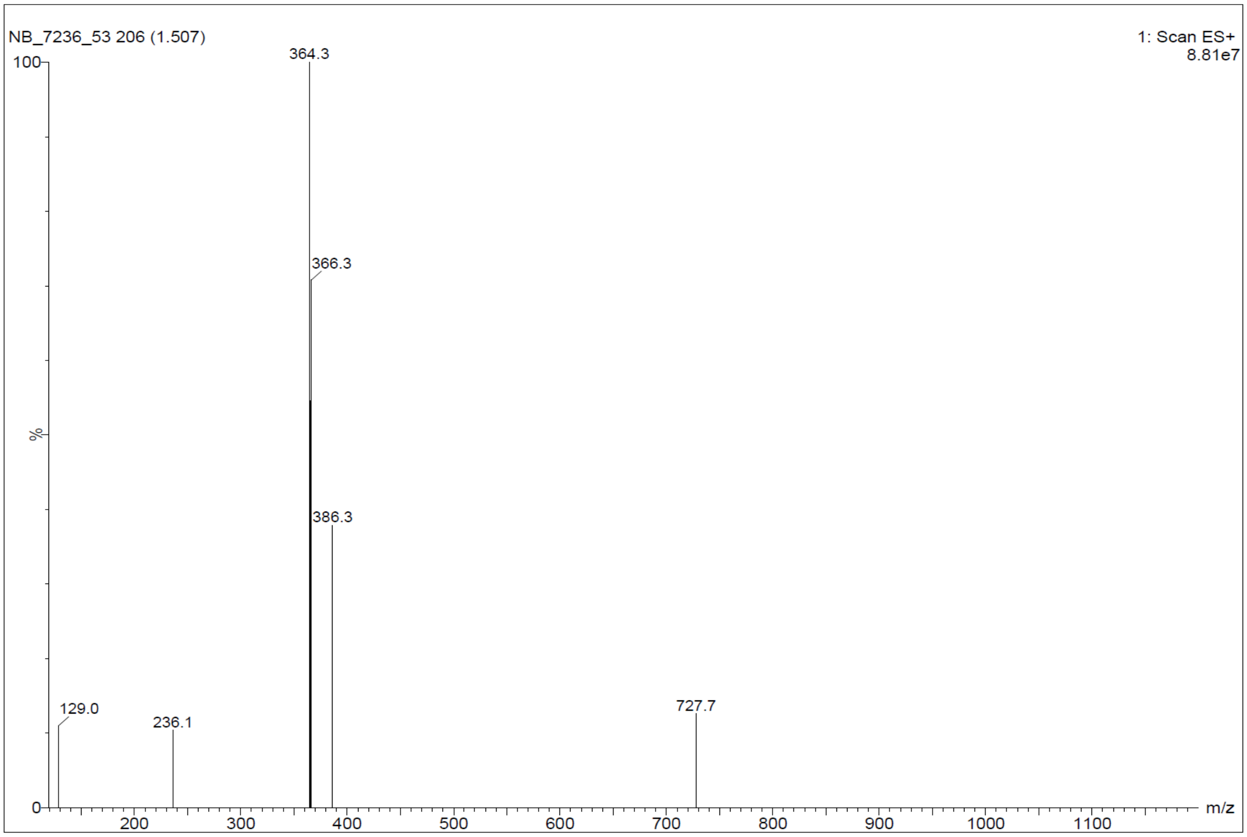
NB_7236_53 206 (1.507)
1: Scan ES+
8.81e7
100
364.3
366.3
%
386.3
129.0
236.1
727.7
0
200 300 400 500 600 700 800 900 1000 1100
m/z

3-(Benzo[b]thiophen-2-yl)-1,4-oxazepane, Free base (4qu)

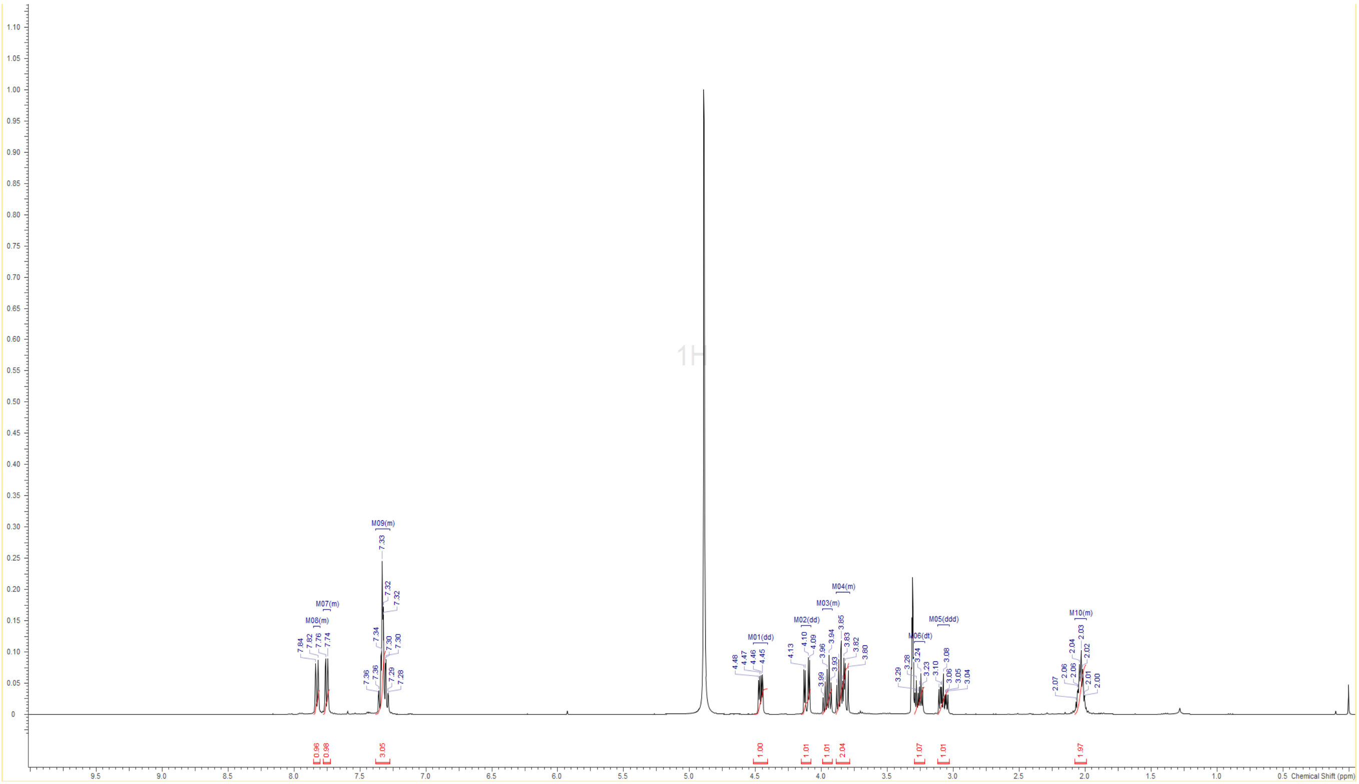

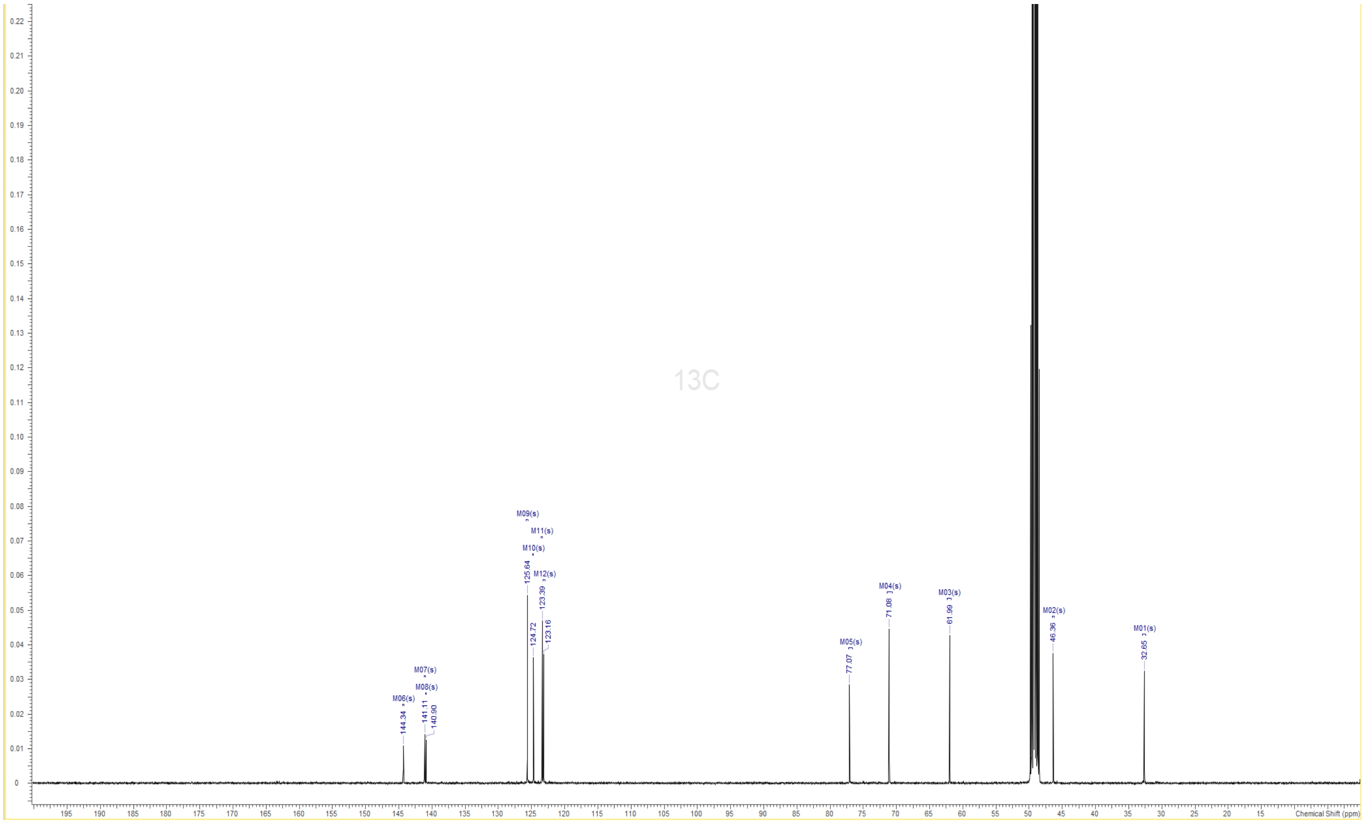

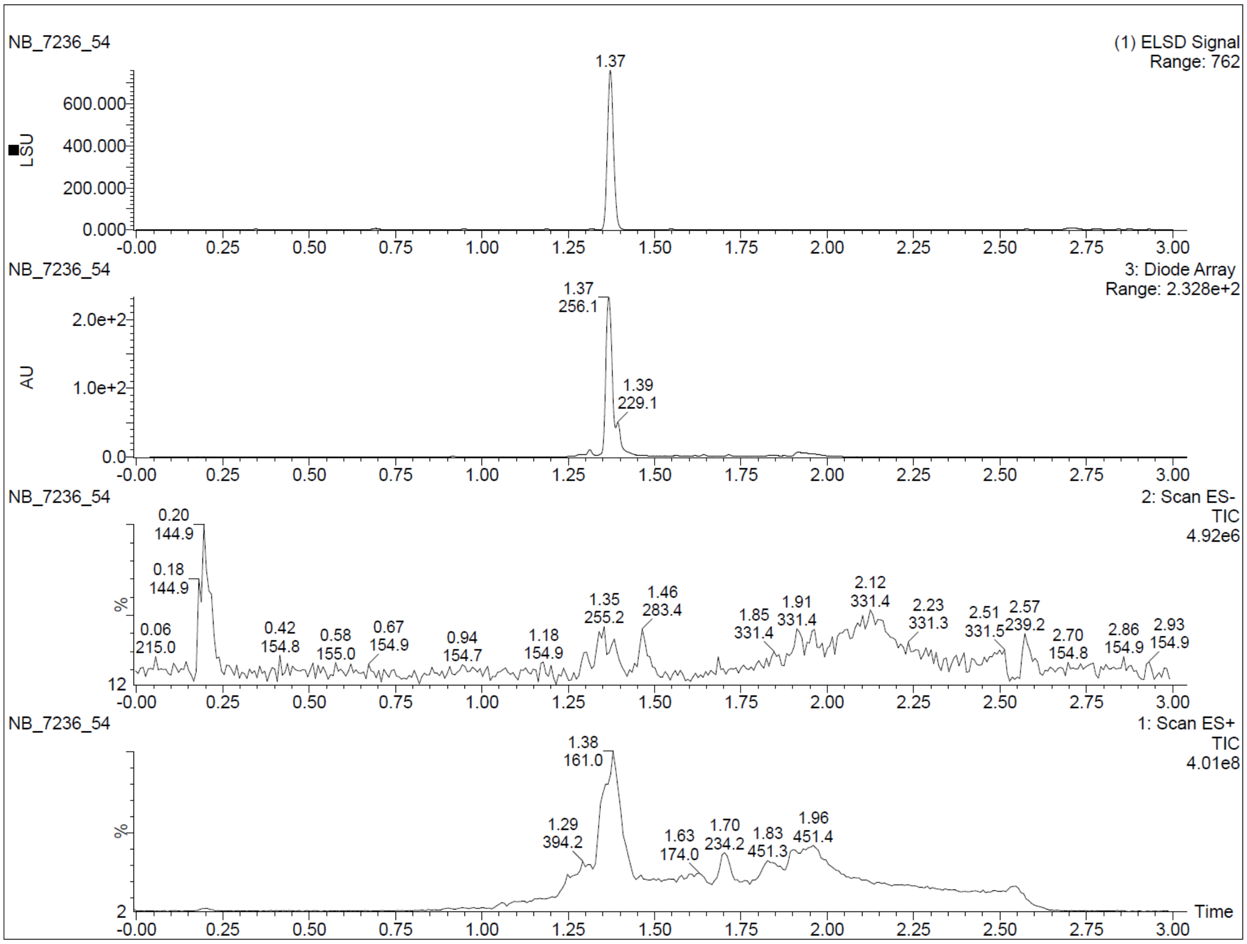
NB_7236_54
(1) ELSD Signal
Range: 762
1.37
LSU
3: Diode Array
Range: 2.328e+2
1.37
256.1
1.39
229.1
AU
2: Scan ES-
TIC
4.92e6
1: Scan ES+
TIC
4.01e8
1.38
161.0
Time

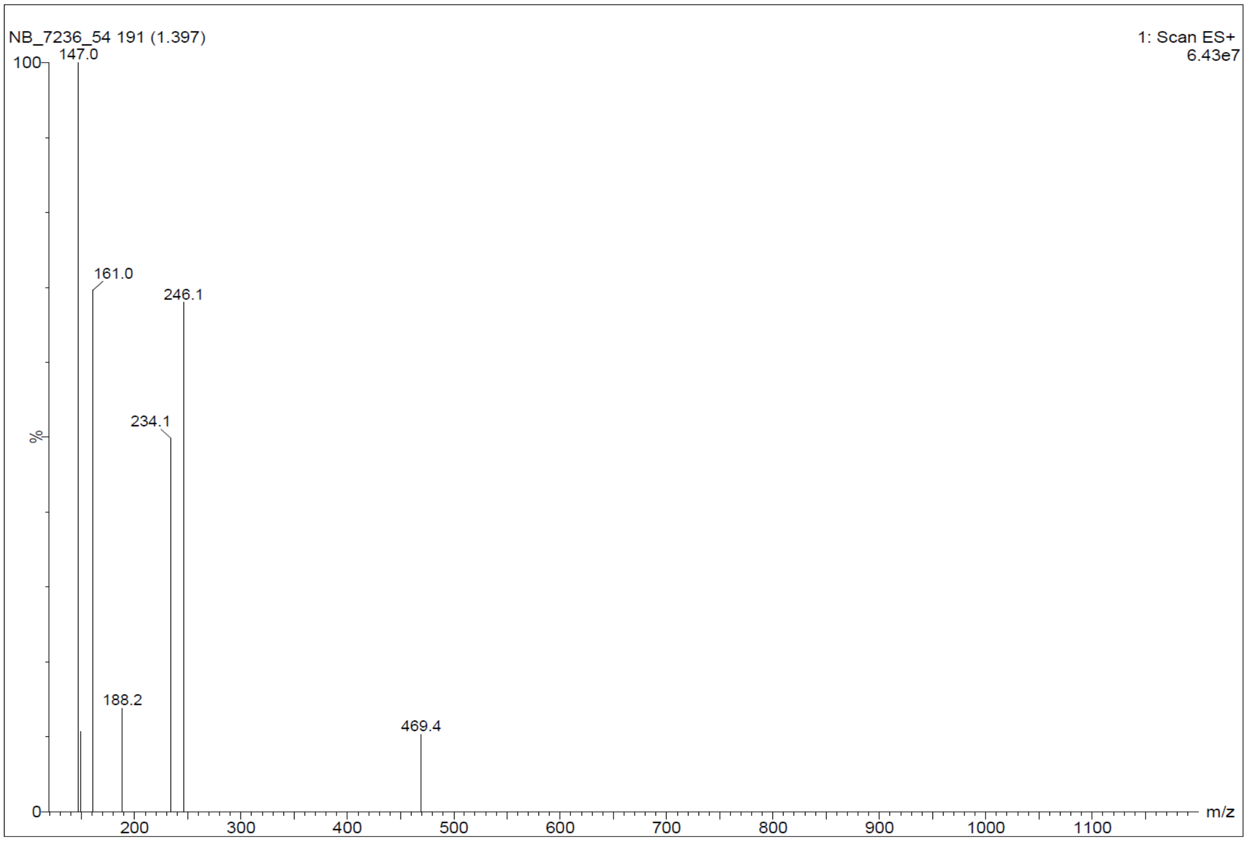
NB_7236_54 191 (1.397)
1: Scan ES+
6.43e7
147.0
161.0
246.1
234.1
188.2
469.4
m/z
